# Nms‐Amides: An Amine Protecting Group with Unique Stability and Selectivity

**DOI:** 10.1002/chem.202301312

**Published:** 2023-06-07

**Authors:** Philipp Spieß, Ana Sirvent, Irmgard Tiefenbrunner, Jules Sargueil, Anthony J. Fernandes, Ana Arroyo‐Bondía, Ricardo Meyrelles, David Just, Alexander Prado‐Roller, Saad Shaaban, Daniel Kaiser, Nuno Maulide

**Affiliations:** ^1^ Institute of Organic Chemistry University of Vienna Währinger Straße 38 1090 Vienna Austria; ^2^ Vienna Doctoral School in Chemistry University of Vienna Währinger Straße 42 1090 Vienna Austria; ^3^ Christian-Doppler Laboratory for Entropy-Oriented Drug Design University of Vienna Josef-Holaubek-Platz 2 1090 Vienna Austria; ^4^ X-ray Structure Analysis Centre University of Vienna Währinger Straße 42 1090 Vienna Austria

**Keywords:** amines, density functional theory calculations, orthogonal deprotection, protecting groups, sulfonamides

## Abstract

*p*‐Toluenesulfonyl (Tosyl) and nitrobenzenesulfonyl (Nosyl) are two of the most common sulfonyl protecting groups for amines in contemporary organic synthesis. While *p*‐toluenesulfonamides are known for their high stability/robustness, their use in multistep synthesis is plagued by difficult removal. Nitrobenzenesulfonamides, on the other hand, are easily cleaved but display limited stability to various reaction conditions. In an effort to resolve this predicament, we herein present a new sulfonamide protecting group, which we term Nms. Initially developed through in silico studies, Nms‐amides overcome these previous limitations and leave no room for compromise. We have investigated the incorporation, robustness and cleavability of this group and found it to be superior to traditional sulfonamide protecting groups in a broad range of case studies.

## Graphical abstract

***p*** **‐Toluenesulfonyl (Tosyl) and nitrobenzenesulfonyl (Nosyl)** are two of the most common sulfonyl protecting groups for amines in contemporary organic synthesis. While *p*‐toluenesulfonamides are known for their high stability and robustness, their use in multistep synthesis is plagued by difficult removal. Nitrobenzenesulfonamides, on the other hand, are easily cleaved but display limited stability to various reaction conditions. In an effort to resolve this conundrum, we herein present Nms‐amides, initially developed through *in silico* studies, which overcome these previous limitations and leave no room for compromise, enabling a range of transformations that are not possible with traditional sulfonamide protecting groups.

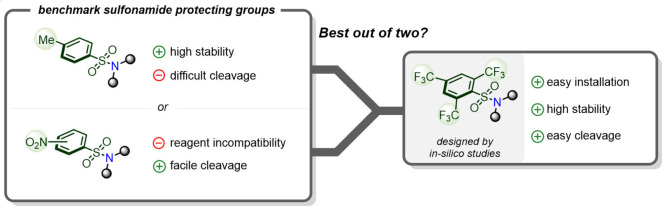

## Introduction

**Scheme 1:**
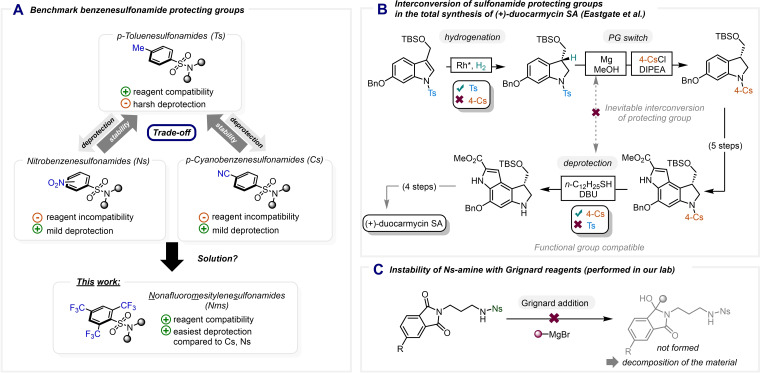
State‐of‐the‐art of benzenesulfonamide protecting groups. A) Comparison between known benzenesulfonamide protecting groups and our new PG. B, C) Selected examples of problems associated with some known benzenesulfonamide protecting groups.

The amine functionality is widely represented in many natural products, pharmaceuticals, agrochemicals and other compounds of importance to the chemical industry.[^1^, ^2^, ^3^] Therefore, considerable research has been devoted to the development of synthetic methods for the preparation of amines.[^4^, ^5^] Due to the high nucleophilicity and polarity of free amines, their temporary transformation into less reactive moieties (“protection”) is a common synthetic device, employed to tame their reactivity within the context of multistep synthesis. As a result, amine protecting groups have emerged as indispensable tools for organic chemists. The key features of any protecting group are 1) ease of installation, 2) robustness under different reaction conditions, and 3) selective cleavage under mild conditions (“deprotection”). The protection of amines as carbamates (Boc, Fmoc), amides, *N*‐alkylated amines (e. g., benzylamines) or as sulfonamides ranks among the most widespread strategies in organic synthesis.^[6]^ Compared to carbamates or amides, NH‐sulfonamides carry a uniquely acidic N−H bond, rendering the sulfonamide anion an attractive reactant in *N*‐alkylations, Mitsunobu reactions and transition metal‐catalyzed protocols.[^7^, ^8^, ^9^, ^10^, ^11^, ^12^] However, while the three key criteria for a synthetically useful protecting group are mostly fulfilled for carbamates and *N*‐alkylated amines, consequently allowing reliable protection of the amine functionality, they are not yet fully met for the arenesulfonamides. *p*‐Toluenesulfonamides (tosylamides), perhaps the most commonly used sulfonamide protecting groups due to unique chemical stability, usually mandate harsh deprotection conditions (reductive[^13^, ^14^, ^15^, ^16^, ^17^, ^18^, ^19^] or acidic[^20^, ^21^]) that are often incompatible with highly functionalized substrates (Scheme 1A). This frequently leads to a redesign of the synthesis, and a range of examples can be found in the literature where protecting group interconversions are needed to avoid a general failure of the synthetic route (cf. Scheme 1B, vide infra).[^8^, ^9^, ^22^] Aiming for a more readily cleavable sulfonamide, Fukuyama and co‐workers pioneered the venerable *o*‐ and *p*‐nitrobenzenesulfonyl (nosyl or Ns) groups.[^7^, ^23^] The electron deficiency of the nosyl group enables its removal under mild conditions, usually employing thiolate nucleophiles capable of nucleophilic aromatic substitution with SO_2_ extrusion.

While the introduction of the nosyl protecting group marked a breakthrough, the presence of the nitro functionality simultaneously renders the nosyl group sensitive to either certain reactant types, such as reducing agents and organometallic reagents, or to photomediated^[24]^ and metal‐catalyzed reactions.[^25^, ^26^] Following this seminal contribution, the 4‐cyanobenzenesulfonamide (4‐Cs or Cs) was described as a protecting group readily cleavable under similar conditions.^[27]^ However, the cyano variant could not solve the main problem of broader reagent compatibility, as shown, for instance, by the total synthesis of (+)‐duocarmycin SA by Eastgate and coworkers (Scheme 1B).^[22]^ The authors had to adjust their synthetic strategy, starting with a Ts‐protected indole, to allow an early hydrogenation. The Ts group then had to be removed immediately after this step and the indole reprotected with the more labile 4‐Cs group, in order to allow milder deprotection at a later stage of the synthesis.

During the development of a target‐oriented synthesis project in our group, we encountered related difficulties with Grignard addition to a phthalimide substrate carrying a nosyl‐protected amine (Scheme 1C). Aiming to circumvent an unwanted protecting group switch (akin to Scheme 1B) during a multi‐step synthetic sequence, we decided to undertake studies to identify an alternative arenesulfonamide. This new protecting group should be easily cleavable (electron‐deficiency required) and not suffer from the problems associated with conventional arenensulfonamide protecting groups (vide supra). Systematic in silico studies, which aided in assessing an array of potential arenesulfonyl groups, led to the identification of 2,4,6‐tris(trifluoromethyl)benzenesulfonyl chloride as a promising reagent for amine protection. Herein we report our results of the exploration of this reagent with regard to its installation, robustness and cleavage, and how it compares to current state‐of‐the‐art sulfonamide protecting groups in a variety of contexts.^[28]^

## Results and Discussion

We envisioned the development of a new sulfonamide protecting group with broader applicability than nosyl (Ns), by substituting the nitro moiety with a more inert electron‐withdrawing group. To examine the feasibility of smooth deprotection of various sulfonamides equipped with suitable electron‐withdrawing groups, we set to computationally investigate the feasibility of nucleophilic aromatic substitution in the presence of thiolate anions for a range of different sulfonamide architectures.

**Figure 1:**
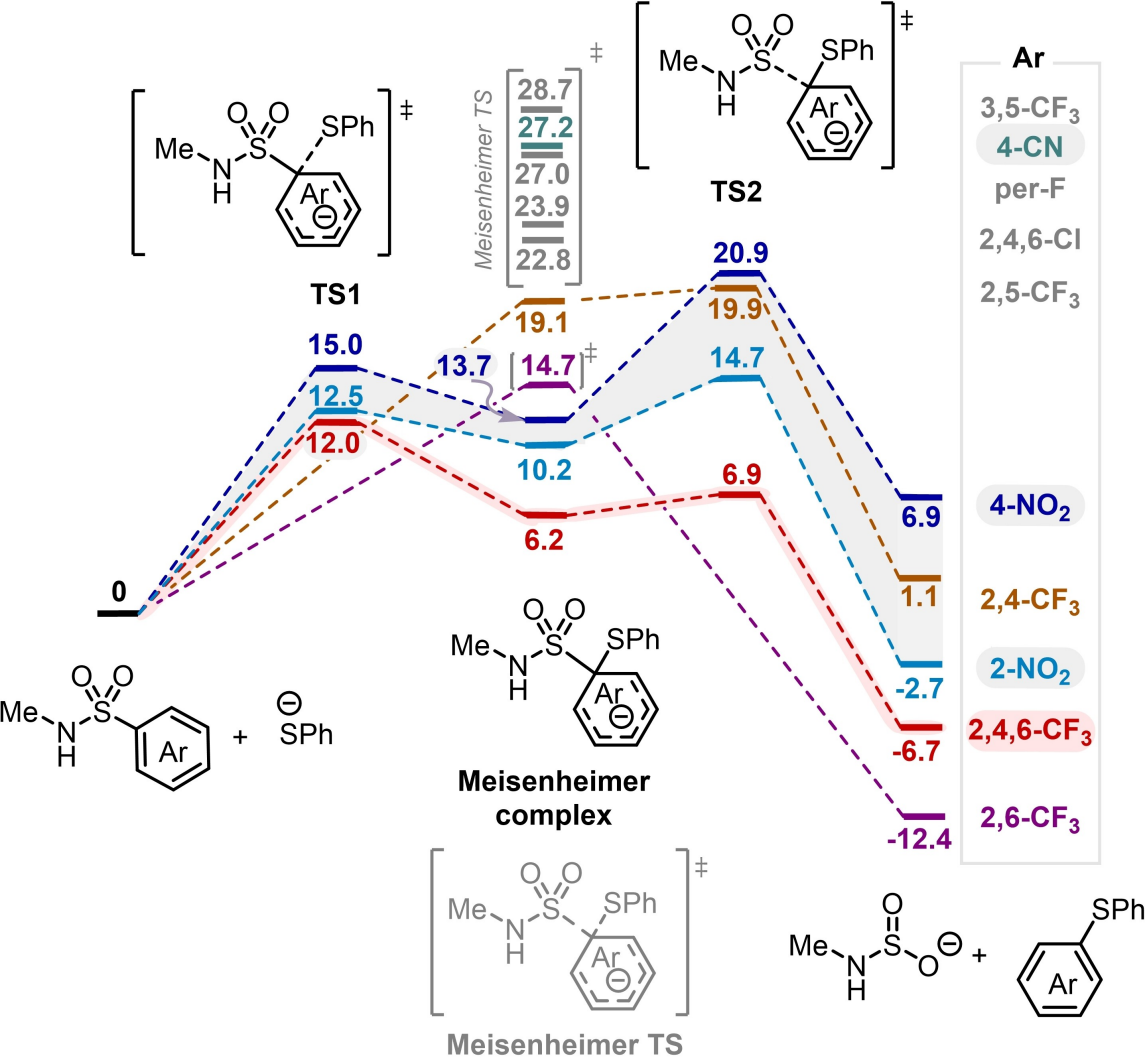
DFT calculations on the deprotection of various sulfonamides. Computed reaction profile for the nucleophilic aromatic substitution of sulfonamides with a phenylthiolate anion as the nucleophile at the PBE0‐D3(BJ)/def2TZVP,SMD//PBE0‐D3(BJ)/def2SVP,SMD level of theory. Relative Gibbs free energies are shown in kcal/mol. The separated reagents serve as reference (0.0 kcal/mol).

For this study, we used quantum chemical calculations at the density functional theory (DFT) level PBE0‐D3(BJ)/def2TZVP,SMD//PBE0‐D3(BJ)/def2SVP,SMD[^29^, ^30^, ^31^, ^32^, ^33^, ^34^, ^35^, ^36^] (see SI for details) to screen a variety of model *N*‐methyl sulfonamides containing fluorine or chlorine atoms attached to the aromatic ring, together with the corresponding *o*‐ and *p*‐nosyl and 4‐cyanobenzenesulfonamides (4‐Cs), and considering a model phenylthiolate anion as the nucleophile (Figure 1). Strikingly, our results showed that, depending on the sulfonamide, the nucleophilic aromatic substitution required for the deprotection process can occur in either a single or two consecutive steps. In the latter case, an initial S−C bond formation between the phenylthiolate and the aromatic ring yields a discrete Meisenheimer complex (MC) intermediate. From this structure, the S−C bond to the arylsulfonamide can cleave to form an aminosulfinate which ultimately converts into the deprotected amine through SO_2_ release.

This *stepwise* profile is common to the 2,4‐bis(trifluoromethyl)benzenesulfonamide (Figure 1, orange), presenting a barrierless first step, to the *o*‐ and *p*‐Ns‐amides (Figure 1, light and dark blue, respectively), for which the S−C bond cleavage (TS2) displays the highest activation barrier, and finally to the 2,4,6‐tris(trifluoromethyl)benzenesulfonamide (Figure 1, red), for which the highest activation barrier is obtained for the first step (formation of the MC). Importantly, this last substrate presents the lowest activation barrier for the aromatic substitution (ΔG^≠^=12.0 kcal/mol), suggesting a facile deprotection process, kinetically favored when compared to the nosyl groups. For all these substrates, the formation of the MC is endergonic (ΔG_MC_ ranges between 19.1 and 6.2 kcal/mol), with again the specific 2,4,6‐tris(trifluoromethyl)benzenesulfonyl substrate presenting the most stable MC intermediate.^[37]^ The aromatic substitution process is not exergonic for all the studied substrates, being thermodynamically favorable especially for the 2,4,6‐tris(trifluoromethyl)benzenesulfonyl and 2,6‐bis(trifluoromethyl)benzenesulfonyl groups. The latter (Figure 1, purple) presents a single step for the aromatic substitution, and therefore the MC is obtained as a transition state rather than an intermediate. The apparent activation barrier for this substrate (ΔG^≠^=14.7 kcal/mol) is still higher than that for the 2,4,6‐tris(trifluoromethyl)benzenesulfonyl group and similar to the obtained barrier for *o*‐Ns. Notably, for the rest of the electron‐deficient arenesulfonamides that we investigated, including the 4‐cyanobenzenesulfonamide, computationally, the Meisenheimer complex‐type structure was again found to be a transition state, but presenting significantly higher activation free energies (ΔG^≠^>22.8 kcal/mol), which suggest a more cumbersome deprotection. The high activation barrier obtained for the 4‐cyanobenzenesulfonamide (ΔG^≠^=27.2 kcal/mol) is in line with the problems observed by Schmidt and coworkers to deprotect this group in primary amines, and the need of a large excess of alkylthiol and base in the deprotection of secondary amines.^[27]^ These computational results suggest that the deprotection of 2,4,6‐tris(trifluoromethyl)benzenesulfonamides should proceed easily and even under milder conditions than those required to remove nosyl groups.

**Scheme 2:**
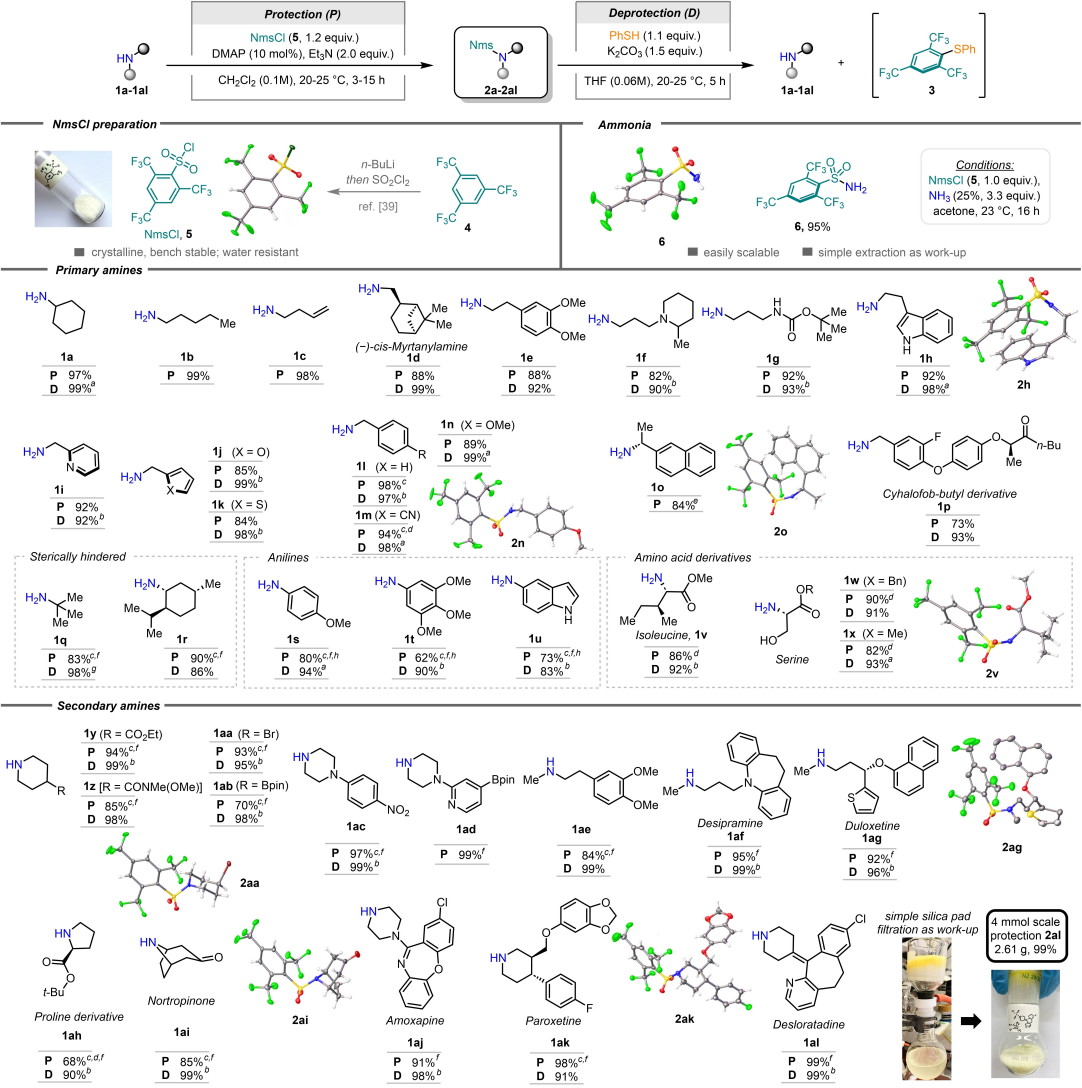
Protection and deprotection of amines with the Nms group. [a] Isolated as the hydrochloride salt. [b] NMR yield using dibromomethane or mesitylene as the internal standard. [c] Addition of NmsCl (**5**) was carried out at 0 °C. [d] From the hydrochloride salt of the amine, 3 equiv. of base were used. [e] 5 mmol scale. [f] 20 mol % of DMAP was used. [g] NMR yield based on the formation of diarylsulfide **3**. [h] 2,6‐lutidine was used as a base.

Following our computational investigation and resulting identification of 2,4,6‐tris(trifluoromethyl)benzenesulfonamides – which we henceforth shall term Nms, derived from the designation “**N**onafluoro**m**esitylene**s**ulfonyl” – as the most promising protecting group, we sought experimental validation of our hypothesis. Synthesis of the corresponding sulfonylating agent (**5**) was achieved by direct lithiation of 1,3,5‐tris(trifluoromethyl)benzene and subsequent addition to SO_2_Cl_2_, following a procedure described in the literature (Scheme 2).[^38^, ^39^] The desired sulfonyl chloride **5** was obtained as a white crystalline solid that proved to be extremely stable under ambient conditions, and even in the presence of water.^[40]^ By treating NmsCl (**5**) with an aqueous solution of ammonia, the primary 2,4,6‐tris(trifluoromethyl)benzenesulfonamide (NmsNH_2_, **6**) could be prepared in excellent yield.

Following a screening of reaction conditions for the formation of secondary and tertiary sulfonamides using sulfonyl chloride **5**,^[41]^ a variety of primary and secondary amines were subjected to protection (Scheme 2). Primary amines were successfully sulfonylated employing triethylamine as the base and catalytic amounts of 4‐dimethylaminopyridine (DMAP). Simple alkyl sulfonamides (**2 a**–**e**), as well as sulfonamides containing different functional groups (**2 f**–**h**, **2 v**–**x**), such as hydroxy, indole or carbamate moieties, were prepared in yields of 82–97 % under these conditions. For benzylamines (**1 i**–**p**), in general, we found that performing the addition of the arenesulfonyl chloride **5** at 0 °C was beneficial.^[42]^ More sterically encumbered primary amines (**1 q**, **1 r**) required a slightly higher loading of DMAP (20 mol %) and increased reaction time to give the corresponding sulfonamides **2 q** and **2 r** in comparable yields. When protecting anilines (**1 s**–**u**), we found that 2,6‐lutidine, replacing triethylamine as the base, led to improved results for the sulfonylation. Finally, a variety of cyclic and acyclic secondary amines were effectively protected in good to excellent yields, under the same conditions found optimal for sterically hindered primary amines. Various functional groups were well tolerated, including halides (**2 aa**, **2 ak**, **2 al**), boronic esters (**2 ab**, **2 ad**), a nitro group (**2 ac**), carbonyls (**2 y**, **2 ai**) and various heterocyclic structures (**2 ag**, **2 aj**, **2 al**). Importantly, sterically hindered amines (**2 ah**, **2 ai**) also provided the desired sulfonamides, albeit in slightly reduced yields compared to unhindered systems. Top‐selling amine‐containing drugs such as desipramine (**1 af**), duloxetine (**1 ag**), amoxapine (**1 aj**), paroxetine (**1 ak**) (all antidepressants) and desloratadine (**1 al**) (antiallergic) could be easily protected, demonstrating the potential suitability of the protection in more challenging settings (**2 af**, **2 ag**, **2 aj**–**2 al**). The protection of desloratadine (**1 al**) was easily scaled up; for purification, short‐path silica gel filtration of the crude material was sufficient to obtain the protected amine (**2 al**) in high purity and quantitative isolated yield. Overall, it is important to highlight that all protections yielded solids, often crystalline (see X‐ray structures in Scheme 2),^[43]^ facilitating the purification and handling of these compounds. This is particularly interesting for industrial processes, as Schmidt *et al*. (Bristol‐Myers Squibb) pointed out for 4‐Cs‐amides.^[27]^

With a plethora of Nms‐amides in hand, we turned our attention to deprotection, for which we chose reaction conditions with nearly stochiometric amounts of thiophenol (1.1 equiv.) and a slight excess of potassium carbonate (1.5 equiv.) at ambient temperature – a milder setup than when more nucleophilic alkyl thiols are used. Additionally, we consider avoiding the use of an excess amount of thiophenol more user‐friendly, as it obviates working with toxic unreacted thiophenol after completion of the reaction. Importantly, however, other thiols, including water‐soluble thiols, were found to perform similarly well.[^41^, ^44^] Overall, an exceptionally smooth deprotection to liberate the free amines **1** in yields above 95 % – on average – was observed, while at the same time showing excellent functional group tolerance (Scheme 2). Notably, the observation and isolation of diarylsulfide **3** lends support to the deprotection mechanism described above (Figure 1).

**Scheme 3:**
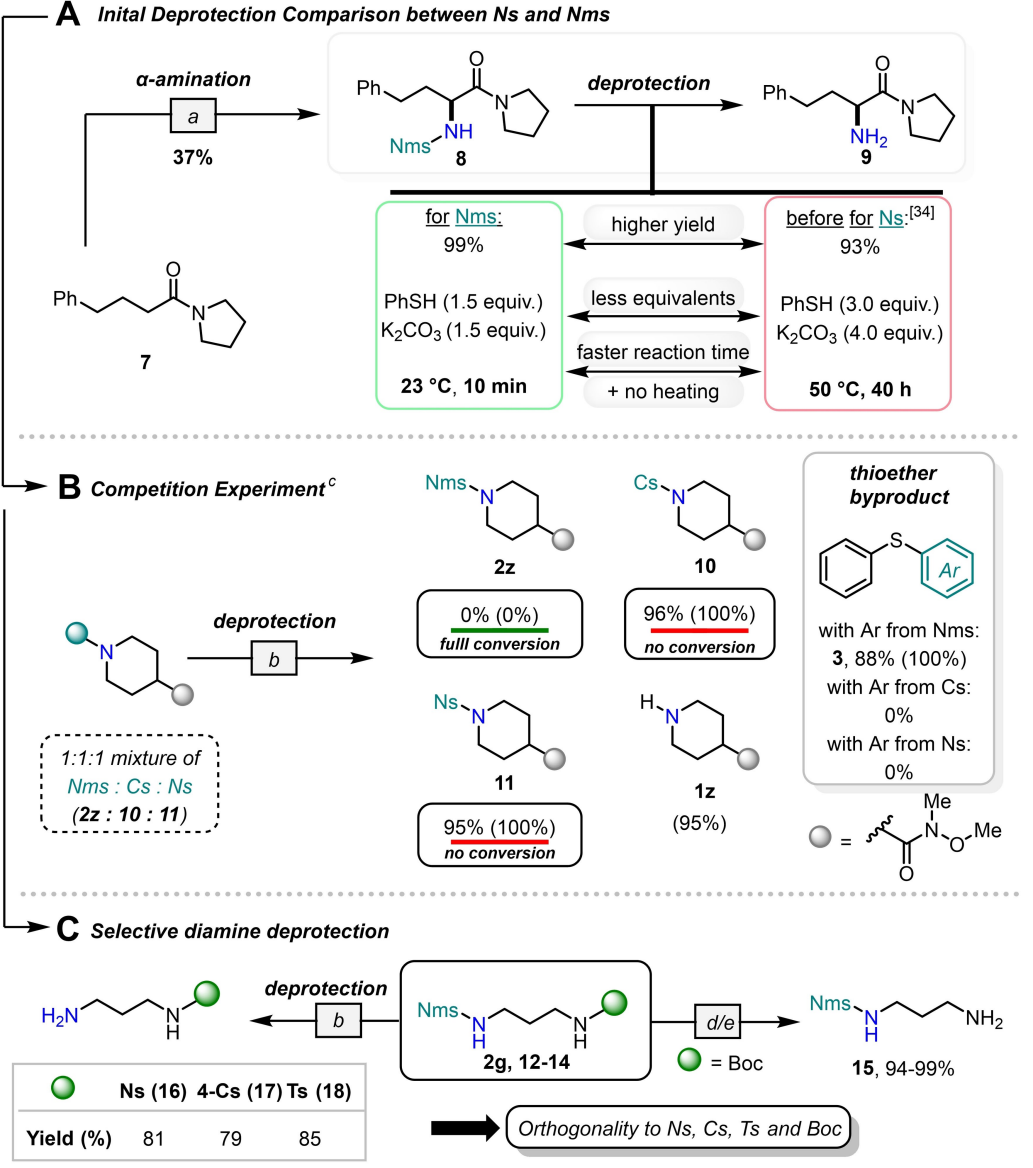
Detailed deprotection studies. Yields in brackets represent NMR yields with CH_2_Br_2_ as the internal standard. [a] Tf_2_O (1.1 equiv.), 2‐I‐pyr (2.2 equiv.), CH_2_Cl_2_ (0.1 M), 0 °C, 15 min, then LNO (1.0 equiv.), 15 min, 0 °C, then **6** (3.0 equiv.) with NaH (3.0 equiv.) in DMF, 20 °C, 3 h. [b] PhSH (1.0 equiv.), K_2_CO_3_ (1.5 equiv.), THF, 23 °C, 16 h. [c] For detailed studies on the kinetics of the deprotection of the Nms group see the Supporting Information. [d] HCl (2 M in Et_2_O, 7 equiv.), MeCN, 20 °C, 2 h, 99 %. [e] TFA (10 equiv.), CH_2_Cl_2_, 20 °C, 2 h, 94 %.

To validate the initially computed easier deprotection, we carried out a detailed study comparing the Nms group with other benzenesulfonamide systems. We first investigated sulfonamide **8**, accessible through our group's amide Umpolung strategy using primary Nms‐amide **6** (vide supra),^[45]^ as we wanted to compare its deprotection with that of the Ns‐protected amine that we had previously studied (Scheme 3A). Cleavage of the Nms group in compound **8** occurred quantitatively at 23 °C in only 10 minutes. For comparison, the earlier deprotection of the Ns‐protected amine required a greater excess of thiol and base, a higher temperature, and a much longer reaction time. To go a step further, we performed a competition experiment by now mixing Nms‐, Ns‐, and Cs‐protected amines (**2 z**, **10, 11**) in a 1 : 1 : 1 ratio. This mixture was subjected to the standard desulfonylation conditions developed for Nms‐amides (Scheme 3B). Surprisingly, the Nms‐amide **2 z** was quantitatively converted to the corresponding free amine (**1 z**), while the Ns‐ and Cs‐amides **10** and **11** remained entirely unaltered. Additionally, we were able to detect and isolate the diarylsulfide by‐product of this reaction (**3**), notably, only the one resulting from Nms deprotection was detected. Remarkably, Nms‐amide **2 z** could even be deprotected under base‐free conditions with an excess of thiophenol.^[41]^ Based on our results, we next enquired whether the Nms group could be used orthogonally to the other benzenesulfonamides (Ns, Cs, Ts). Diamines carrying a combination of Nms with Ns, Cs or Ts (**12**–**14**) groups were easily unravelled (standard deprotection conditions for the removal of Nms) and gave exclusively the desired monosulfonamides (**16**‐**18**) in high yields throughout (Scheme 3C).^[46]^ Finally, we examined the Boc‐Nms protected diamine **2 g**. In this case, the stability of the Nms group allowed the selective cleavage of the Boc group in quantitative yields by either HCl or TFA treatment.

**Scheme 4:**
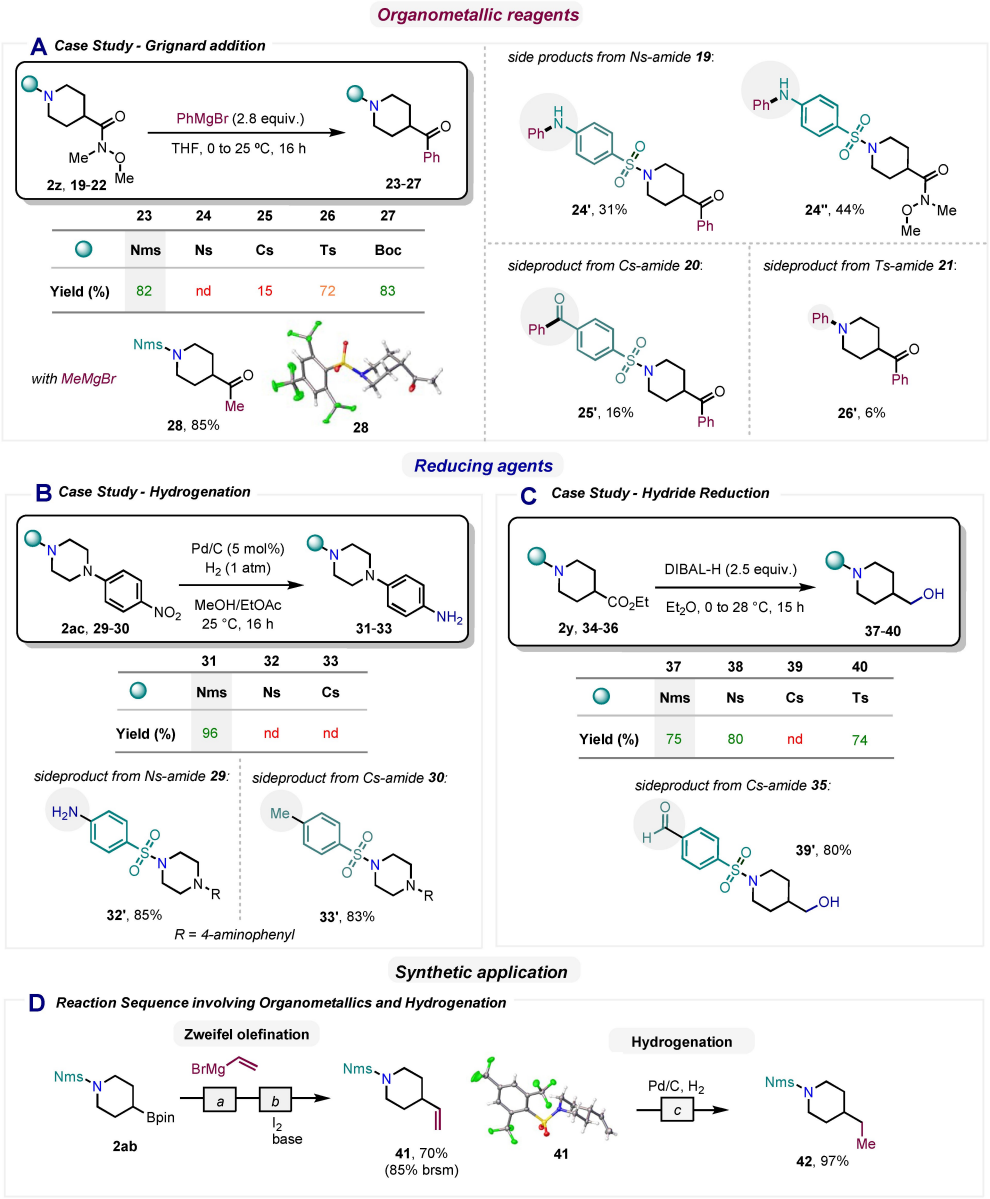
Stability of Nms‐amides. [a] Vinylmagensium bromide (1.2 equiv.), THF/DMSO (1 : 1), 0 °C to 23 °C, 30 min. [b] NaOMe (3 equiv.), I_2_ (1.2 equiv.), 0 °C, 30 min. [c] Pd/C (5 mol %), H_2_ (1 atm), MeOH/EtOAc, 25 °C,16 h.

Next, we investigated the stability of the Nms group under different reaction conditions, while making a comparison with other benchmark benzenesulfonamide protecting groups such as Ns, Cs, and Ts (Scheme 4). In these case studies, we wanted to document the overall stability of different sulfonamides towards some typical reaction conditions. Our first study centered on Grignard reagents, our initial motivation for conducting the research presented herein (vide supra), and on the Weinreb‐Nahm ketone synthesis as a case study reaction. The protected Weinreb amides **2 z** and **19**–**22** were treated with an excess of phenylmagnesium bromide to ensure complete conversion (Scheme 4A). We were pleased to see that the reaction with Nms‐amide **2 z** provided the desired ketone product **23** in 82 % yield, a result comparable to the usually organometallic‐stable, Boc‐protected amine **22**, which gave an 83% yield of **27**. In contrast, when we attempted to perform the same transformation on amines protected with the Ns and Cs groups (**19**, **20**), respectively, we observed the formation of various side products (compounds **24’**, **24’’** and **25’**) resulting from addition of the organomagnesium reagent to the nitro or nitrile functionality. In fact, in the case of the Ns‐amide **19**, the desired ketone **24** was not detected, while ketone **25**, derived from Cs‐amide (**20**), was isolated in only 15 % yield. To our surprise, even the Ts‐amide **21**, known for its high stability, did not react entirely selectively, providing the desired product of Grignard addition (**26**) in 72 % yield alongside a small amount of the undesired aniline (**26’**). To rule out a specific effect of arylmagnesium reagents, the same Nms‐protected Weinreb amide compound (**2 z**) was treated with methylmagnesium bromide, allowing an 85 % yield of the desired alkyl ketone (**28**).

Next, we focused on stability toward reductive conditions. We began by investigating hydrogenation with Pd/C, which had previously proven difficult for Cs‐amides under Rh catalysis (Scheme 1B).^[22]^ We were pleased to see that the hydrogenation of the nitro group of Nms‐amide (**2 ac**) proceeded quantitatively to afford the desired aniline **31** (Scheme 4B). In sharp contrast, the Ns‐ and Cs‐amides **29**–**30** were found to be incompatible with hydrogenation. Consequently, we isolated compounds **32’** and **33’**, which were formed by undesired additional reduction of the nitro and nitrile groups of Ns‐ and Cs‐amides **29** and **30**, respectively. Next, we devoted our attention to ester reduction with the commonly used hydride source DIBAL‐H. While Nms‐ (**2 y**), Ns‐ (**34**) and Ts‐ (**36**) containing substrates were able to give the desired alcohol product with very comparable results, the Cs‐amide (**35**) afforded the nitrile reduction product as the main compound (**39’**) (Scheme 4C). To further highlight the consequences of the above observations, we carried out a two‐step synthetic sequence towards compound **42**, consisting in a Zweifel olefination of Nms‐amide **2 ab** and a subsequent hydrogenation (Scheme 4D). As shown by the results discussed before, this sequence would not be possible in the presence of either the Ns or the Cs groups.

**Scheme 5:**
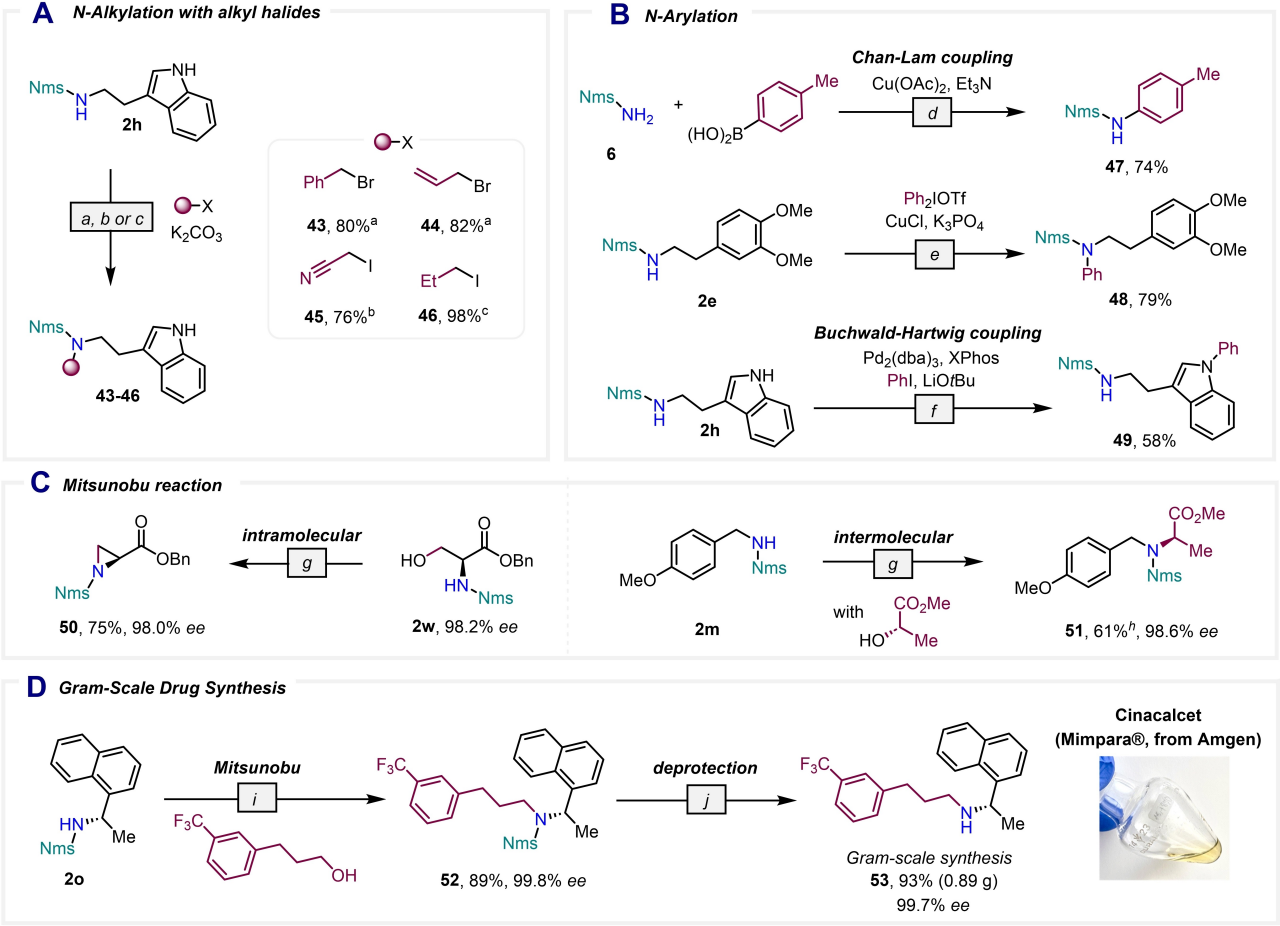
Derivatization of Nms‐amides. [a] Alkyl halide (5.0 equiv.), K_2_CO_3_ (5.0 equiv.), acetone (0.05 M), 23 °C, 30 h. [b] Alkyl halide (1.5 equiv.), K_2_CO_3_ (1.5 equiv.), acetone (0.05 M), 23 °C, 30 h. [c] Alkyl halide (5.0 equiv.), K_2_CO_3_ (5.0 equiv.), acetone (0.05 M), 60 °C, 30 h. [d] Cu(OAc) _2_ (2.0 equiv.), ArB(OH) _2_ (2.0 equiv.), Et_3_N (2.0 equiv.), CH_2_Cl_2_ (0.06 M), 21 °C, 48 h. [e] Ph_2_IOTf (2.0 equiv.), CuCl (20 mol %), K_3_PO_4_ (2.0 equiv.), CH_2_Cl_2_ (0.1 M), 40 °C, 48 h. [f] Pd_2_(dba)_3_ (20 mol %), XPhos (40 mol %), PhI (5.0 equiv.), LiO*t*Bu (2.0 equiv.), THF (0.1 M), 80 °C, 12 h [g] Alcohol (1.0 equiv.), PPh_3_ (2.0 equiv.), DEAD (2.0 equiv.), THF (0.1 M), 22 °C, 48 h. [h] When 1.5 equiv. of the alcohol were used, **51** was obtained in 84 % yield. [i] Alcohol (1.4 equiv.), PPh_3_ (2.0 equiv.), DEAD (2.0 equiv.), THF (0.1 M), 0 to 25 °C, 96 h. [j] PhSH (1.2 equiv.), K_2_CO_3_ (1.5 equiv.), 23 °C, 40 h then 30 °C, 3 h.

Finally, the applicability of the Nms‐amides in common sulfonamide reactions was investigated (Scheme 5). The *N*‐alkylation of Nms‐amide **2 h** with different alkyl halides under mild basic conditions proceeded in excellent yield (Scheme 5A), and the *N*‐arylation of Nms‐amides was also possible (Scheme 5B). For instance, the *N*‐aryl Nms‐amide **47** was prepared in good yield through a Chan‐Evans‐Lam coupling of the primary Nms‐amide **6** and *p*‐tolueneboronic acid, while compound **2 e** was successfully converted into the corresponding *N*‐phenylsulfonamide **48** employing diphenyliodonium triflate as the arylating agent. The Nms group was also found to be stable under Buchwald‐Hartwig coupling conditions, employing LiO*t*Bu at 80 °C, as exemplified in the transformation of Nms‐amide **2 h** into compound **49**. Intra‐ and intermolecular Mitsunobu reactions that enabled access to the Nms‐protected aziridine **50** and the amino ester **51** in good yields are also feasible (Scheme 5C). Following a similar strategy, we prepared the therapeutic agent cinacalcet (**53**), a calcimimetic and P450 inhibitor that is used for the treatment of hyperparathyroidism,[^47^, ^48^, ^49^] in good yield *via* a gram‐scale synthesis where the key step was an intermolecular Mitsunobu reaction of sulfonamide **52** with a primary alcohol (Scheme 5D).

## Conclusion

Guided by an in silico DFT analysis we have identified a new arenesulfonamide‐based amine protecting group. Subsequently, the installation and cleavage of this group were studied in detail, showing a broad scope and wide functional‐group tolerance. In stability tests and comprehensive deprotection studies, the Nms‐amide was found to be superior to benchmark sulfonyl protecting groups (Ts, Ns, Cs), a superiority further underlined by (orthogonal) competition studies and a range of synthetic case studies. We believe that the Nms protecting group will find wide application in multistep synthesis that may have so far suffered from the classical dilemma of stability vs deprotection, often encountered when using traditional arenesulfonamides.

## Supporting Information

Additional references cited within the Supporting Information.[^50^, ^51^, ^52^, ^53^, ^54^, ^55^, ^56^, ^57^, ^58^, ^59^, ^60^, ^61^, ^62^, ^63^, ^64^, ^65^, ^66^, ^67^, ^68^, ^69^, ^70^, ^71^, ^72^]

## Conflict of interest

The authors declare no conflict of interest.

1

## Supporting information

As a service to our authors and readers, this journal provides supporting information supplied by the authors. Such materials are peer reviewed and may be re‐organized for online delivery, but are not copy‐edited or typeset. Technical support issues arising from supporting information (other than missing files) should be addressed to the authors.

Supporting Information

## Data Availability

The data that support the findings of this study are available in the supplementary material of this article.
