# Supplementary material for: Nms‐Amides: An Amine Protecting Group with Unique Stability and Selectivity
Source: Chemistry. 2023 Jun 7;29(41):e202301312. doi: 10.1002/chem.202301312 (PMC10946766; doi:10.1002/chem.202301312)
Supplement: Supplementary file 1 — Supporting Information [file CHEM-29-0-s001.pdf]

Chemistry–A European Journal

Supporting Information

Nms-Amides: An Amine Protecting Group with Unique Stability and Selectivity

Philipp Spieß, Ana Sirvent, Irmgard Tiefenbrunner, Jules Sargueil, Anthony J. Fernandes, Ana Arroyo-Bondía, Ricardo Meyrelles, David Just, Alexander Prado-Roller, Saad Shaaban, Daniel Kaiser, and Nuno Maulide*

Nms-amides: an Amine Protecting Group with Unique Stability and Selectivity

Philipp Spieß,[a,b,‡] Ana Sirvent,[a,c,‡] Irmgard Tiefenbrunner,[a] Jules Sargueil, Anthony J. Fernandes,[a,c] Ana Arroyo-Bondía,[a] Ricardo Meyrelles,[a,b] David Just,[a] Alexander Prado-Roller,[d] Saad Shaaban,[a] Daniel Kaiser,[a] and Nuno Maulide*[a]

E-Mail: nuno.maulide@univie.ac.at, Homepage: http://maulide.univie.ac.at

Contents

1. General Information ... 3
2. Experimental ... 4
2.1. Synthesis of NmsCl (5) ... 4
2.2. Optimization ... 5
2.2.1 Optimization of protection ... 5
2.2.2 Optimization of deprotection ... 7
2.3. General Procedures ... 9
2.3.1. General Procedures for the protection of amines ... 9
2.3.2. General Procedure for the deprotection of sulfonamides ... 10
2.4. Synthesis of starting materials ... 11
2.5. Protection of amines (2a-2al) ... 12
2.5.1. Ammonia surrogate (6) ... 12
2.5.2 Primary amines (2a-2x) ... 13
3.5.3. Secondary amines (2y-2al) ... 26
2.5.4 Scale-up of protection ... 34
2.6. Deprotection of amines (1a-1al) ... 35
2.6.1. Primary amines (1a-1x) ... 35
3.6.2. Secondary amines (1y-1al) ... 40
2.6.3. Diaryl sulfide 3 ... 44
3. Deprotection studies ... 45
3.1 Comparison Study of Nms- and Ns-amides derived from amide Umpolung ... 45
3.1.1 Preparation of compound 8 ... 45
3.1.2 Deprotection of compound 8 ... 45
3.2 Competition experiment (Ns, Cs, Nms) ... 46
3.3 Deprotection followed by in-situ IR and NMR techniques ... 49
3.4. Selective diamine deprotection ... 57
3.4.1 Orthogonal Boc deprotection of sulfonamide 2g ... 57

3.4.2 Orthogonal Nms cleavage in the presence of other sulfonamides 58
4. Stability of Nms-amides 62
4.1 Case study - Grignard addition 62
4.1.1 Preparation of starting materials 62
4.1.2 General procedure 64
4.1.3 Reactions results of different sulfonamides 65
4.1.4 Other Grignard reagent (MeMgBr) 69
4.2 Case study - Hydrogenation 70
4.2.1 Preparation of starting materials 70
4.2.2 General procedure 71
4.2.3 Reactions results of different sulfonamides 72
4.3 Case Study – Hydride Reduction 75
4.3.1 Preparation of starting materials 75
4.3.2 General Procedure 77
4.3.3 Reaction results of different sulfonamides 77
4.4 Reaction Sequence towards **42** 79
5. Derivatization of Nms-amides 81
5.1. *N*-Alkylation 81
5.2. *N*-Arylation 84
5.3. Mitsunobu reaction 86
5.4 Synthesis of Cinacalcet (53) 88
6. DFT calculations 92
6.1. XYZ structures 92
7. References 102
8. NMR Spectra 103
9. X-Ray Crystallographic Data 252

1. General Information

All solvents were distilled from appropriate drying agents prior to use or directly taken from commercial sealed bottles under an atmosphere of argon. All reagents were used as received from commercial suppliers unless otherwise stated. Reaction progress was monitored by thin layer chromatography (TLC) performed on aluminum plates coated with silica gel F254 with 0.2 mm thickness. Chromatograms were visualized by fluorescence quenching with UV light at 254 nm or by staining using potassium permanganate. Flash column chromatography was performed using silica gel 60 (230-400 mesh, Merck and co.). Neat infrared spectra were recorded using a Perkin-Elmer Spectrum 100 FT-IR spectrometer. Wavenumbers (ν_{max}) are reported in cm^{-1}. Mass spectra were obtained using a Finnigan MAT 8200 or (70 eV) or an Agilent 5973 (70 eV) spectrometer, using electrospray ionization (ESI). All ^{1}H, ^{13}C and ^{19}F NMR spectra were recorded using a Bruker AV-400, AV-600 spectrometer or AV-700 spectrometer at 300K. Chemical shifts are given in parts per million (ppm, δ), referenced to the solvent peak of $CDCl_3$ [defined at δ = 7.26 ppm (^{1}H NMR) and δ = 77.16 (^{13}C NMR)], MeOD [defined at δ = 3.31 ppm (^{1}H NMR) and δ = 49.00 (^{13}C NMR)], acetone-d_6 [defined at δ = 2.05 ppm (^{1}H NMR) and δ = 29.84 (^{13}C NMR)], or DMSO-d_6 [defined at δ = 2.50 ppm (^{1}H NMR) and δ = 32.52 (^{13}C NMR)]. Coupling constants are quoted in Hz (*J*). ^{1}H NMR splitting patterns are designated as singlet (s), doublet (d), triplet (t), quartet (q) and quintet (quint) as they appeared in the spectrum. If the appearance of a signal differs from the expected splitting pattern, the observed pattern is designated as apparent (app). Splitting patterns that could not be interpreted or easily visualized are designated as multiplet (m) or broad (br).

2. Experimental

2.1. Synthesis of NmsCl (5)

2,4,6-tris(Trifluoromethyl)sulfonyl chloride **5** was obtained as a white solid following a procedure reported in the literature.[1]

2,4,6-Tris(trifluoromethyl)benzenesulfonyl chloride (5)

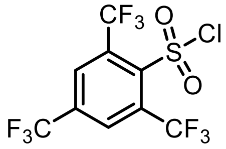

^{1}H NMR (400 MHz, $CDCl_3$): δ 8.43 (s, 2H) ppm.

^{19}F NMR (377 MHz, $CDCl_3$): δ -54.3 (6F), -63.7 (3F) ppm.

^{13}C NMR (101 MHz, $CDCl_3$): δ 145.5, 137.1 (q, *J* = 35.8 Hz), 133.6 (q, *J* = 35.3 Hz), 130.2 (m), 121.6 (q, *J* = 276.9 Hz) ppm.

IR (neat): ν_{max} 1402, 1270, 1154, 1136, 1086, 921, 709, 564, 459 cm^{-1}.

GC-MS (QTOF): exact mass calculated for [M-Cl] ($C_9H_2F_9O_2S$) requires m/z 344.9632, m/z found: 344.9624.

2.2. Optimization

2.2.1 Optimization of protection

Table S1. Optimization of the protection of the non-hindered primary amine **1a**

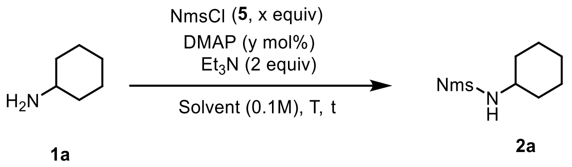

Entry[a]	T (°C)	t (h)	Equiv. of NmsCl	DMAP	Solvent	NMR yield (%)[b]
1	20	0.5	1.1	-	CH_2Cl_2	94
2	40	0.5	1.1	-	CH_2Cl_2	89
3	20	3	1.5	-	CH_2Cl_2	91
4	20	0.5	1.1	10 mol%	CH_2Cl_2	93
5	20	3	1.1	10 mol%	CH_2Cl_2	96 (97)[c]
6	20	3	1.2	10 mol%	CH_2Cl_2	99
7	20	3	1.2	10 mol%	THF	35
8	20	3	1.2	10 mol%	CH_3CN	97
9	20	3	1.2	10 mol%	DMF	88
10	20	3	1.2	10 mol%	Acetone	69
11	20	3	1.2	10 mol%	MeOH	39

[a]Reactions conducted on 0.1 mmol scale. [b]Determined from the ^1H NMR spectrum of the crude using mesitylene as internal standard. [c]Isolated yield after column chromatography.

Table S2. Optimization of the protection of the benzylamine **1m**

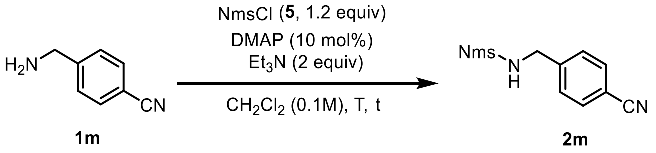

Entry[a]	T (°C)	t (h)	Isolated yield (%)
1	23	3	74
**2**	0 to 23	15	94

[a]Reactions conducted on 0.1 mmol scale.

Table S3. Optimization of the protection of the hindered primary amine **1q**

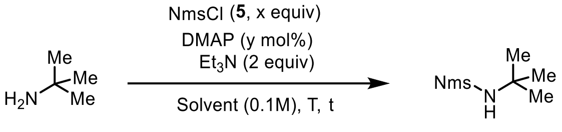

Entry[a]	T (°C)	t (h)	Equiv. of NmsCl	DMAP	Solvent	NMR yield (%)[b]
1	20	3	1.1	-	CH_2Cl_2	21
2	40	3	1.1	-	CH_2Cl_2	9
3	20	3	1.5	-	CH_2Cl_2	23
4	20	3	1.1	10 mol%	CH_2Cl_2	60
5	20	3	1.2	10 mol%	CH_2Cl_2	66
6	20	3	1.1	20 mol%	CH_2Cl_2	67 (78)[c]
7	35	3	1.1	20 mol%	CH_2Cl_2	48
8	20	3	1.2	20 mol%	THF	0
9	0 to 20	24	1.1	20 mol%	CH_2Cl_2	73
10	0 to 20	24	1.2	20 mol%	CH_2Cl_2	77 (83)[c]

[a]Reactions conducted on 0.1 mmol scale. [b]Determined from the ^{1}H NMR spectrum of the crude using mesitylene as internal standard. [c]Isolated yield after column chromatography.

Table S4. Optimization of the protection of the aniline **1s**

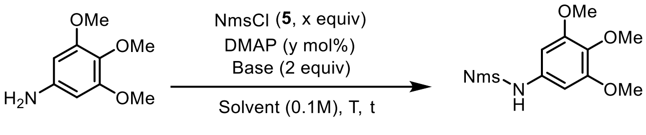

1t **2t**

Entry[a]	T (°C)	t (h)	Equiv. of NmsCl	DMAP	Solvent	Base	NMR yield (%)[b]
1	20	3	1.1	10 mol%	CH_2Cl_2	Et_3N	32
2	20	3	1.1	10 mol%	CH_2Cl_2	2,6-lutidine	37
3	0 to 20	24	1.2	10 mol%	CH_2Cl_2	pyridine	34
4	0 to 20	24	1.2	20 mol%	DMF	-	<5
5	0 to 20	24	1.2	20 mol%	pyridine	-	56
6	0 to 20	24	1.2	20 mol%[c]	CH_2Cl_2	pyridine	37
7	0 to 20	24	1.2	20 mol%	CH_2Cl_2	pyridine	46
8	0 to 20	24	1.2	20 mol%	CH_2Cl_2	2,6-lutidine	56 (62)[d]
9	0 to 20	24	1.2	25 mol%	CH_2Cl_2	Et_3N	56

[a]Reactions conducted on 0.1 mmol scale. [b]Determined from the ^1H NMR spectrum of the crude using mesitylene as internal standard. [c]Imidazole was used instead of DMAP. [d]Isolated yield after column chromatography.

2.2.2 Optimization of deprotection

Table S5. Optimization of the deprotection

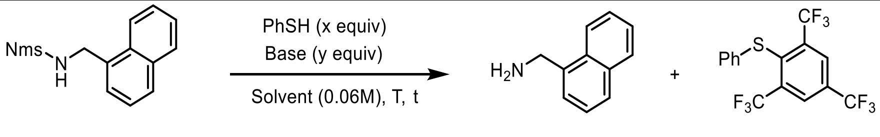

5

Entry[a]	T (°C)	t (h)	Base (equiv.)	PhSH (equiv.)	Solvent	Conversion (%)[b]	NMR yield (%)[b]
1	50	2	K_2CO_3 (4)	3	CH_3CN	100	77
2	50	2	K_2CO_3 (1.5)	1	CH_3CN	99	62
3	24	2	K_2CO_3 (1.5)	1	CH_3CN	94	82
4	24	2	Cs_2CO_3 (1.5)	1	CH_3CN	100	68
5	24	2	K_2CO_3 (1.5)	1	THF	100	85
6	24	26	K_2CO_3 (1.5)	1	1,4-Dioxane	95	67
7	24	26	K_2CO_3 (1.5)	1	Toluene	69	51

[a]Reactions conducted on 0.1 mmol scale. [b]Determined from the ^1H NMR spectrum of the crude using 1,3,5-trimethoxybenzene or mesitylene as internal standard.

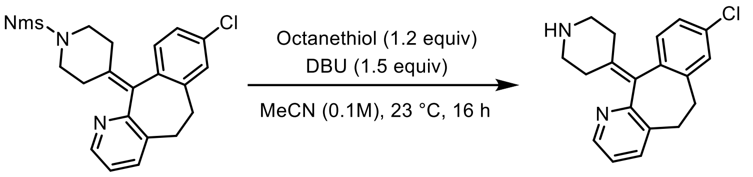

2al **1al**

92% NMR yield

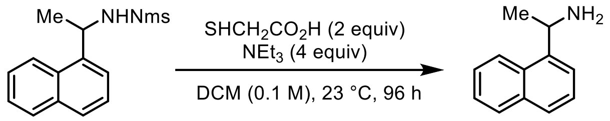

2o **1o**

81% NMR yield

Reactions were conducted on 0.1 mmol scale and the NMR yields were determined from the ^1H NMR of the crude employing mesitylene or CH_2Br_2 as internal standard.

Scheme S1. Deprotection of the Nms group employing other thiols.

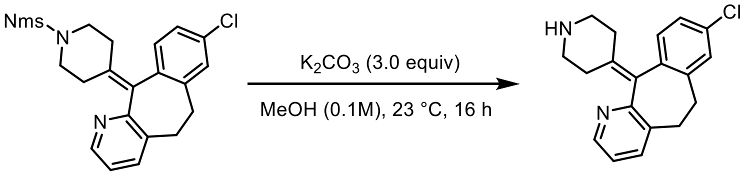

2al **1al**

90% NMR yield

Reaction was conducted on 0.1 mmol scale and the NMR yield was determined from the ^1H NMR of the crude employing CH_2Br_2 as internal standard.

Scheme S2. Alternative conditions for the deprotection of the Nms group.

2.3. General Procedures

2.3.1. General Procedures for the protection of amines

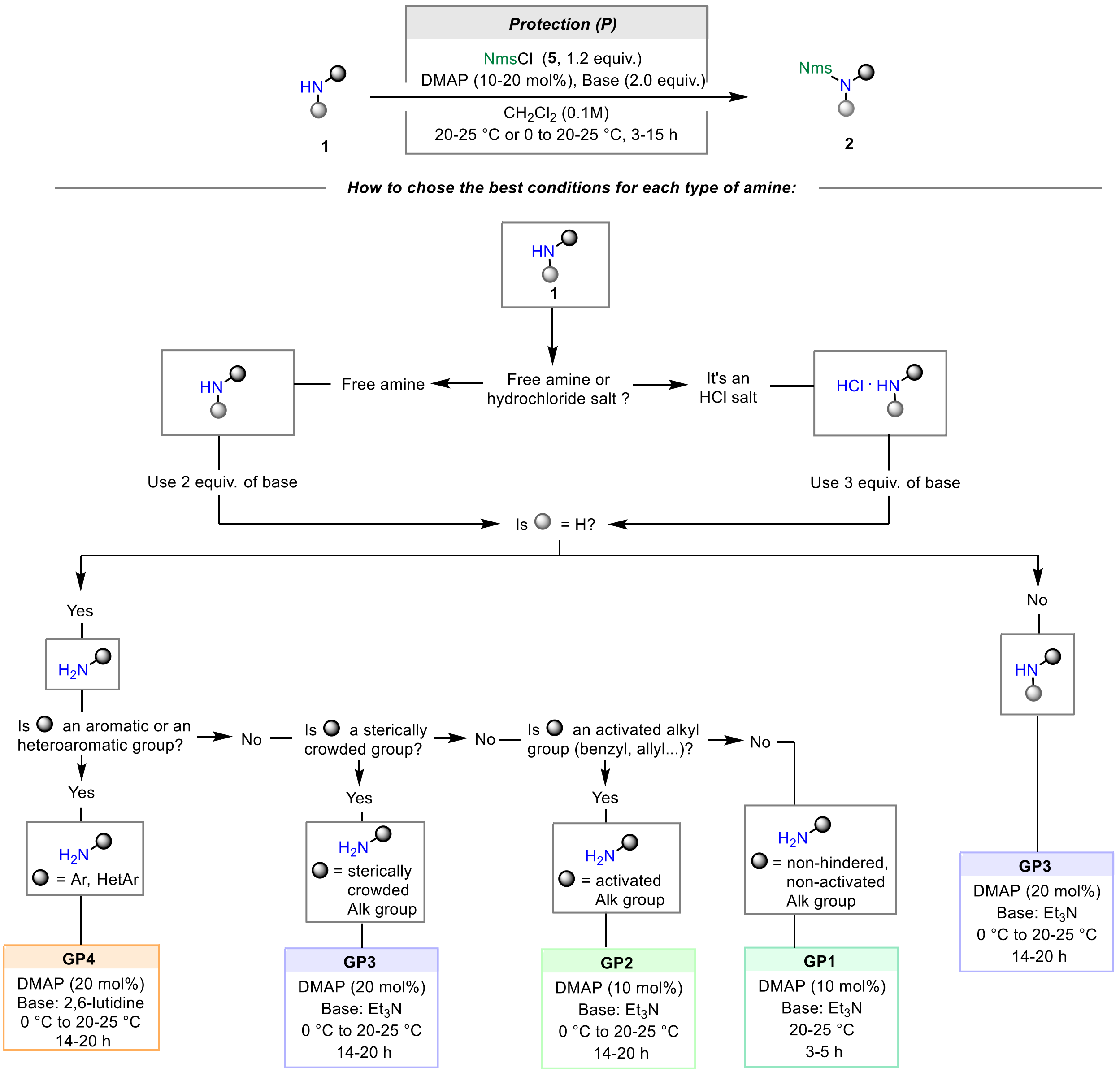

General Procedure 1 (GP1, Protection of non-hindered primary amines):

The corresponding amine **1** (1.0 equiv.) was dissolved in DCM (0.1M) at room temperature (18-25 °C). DMAP (10 mol%), Et_3N (2.0 equiv. for free amines, 3.0 equiv for hydrochloride salts of amines) and the arenesulfonyl chloride **5** (1.2 equiv.) were added sequentially to the solution at the same temperature. The mixture was stirred at room temperature for 3-5 h. The progress of the reaction was monitored by TLC analysis. Then, the solvents were removed under vacuum. The residue was purified by column chromatography (silica gel, heptane:EtOAc).

General Procedure 2 (GP2, Protection of benzylamines):

The corresponding amine **1** (1.0 equiv.) was dissolved in DCM (0.1M) at room temperature (18-25 °C). DMAP (10 mol%), Et_3N (2.0 equiv. for free amines, 3.0 equiv. for hydrochloride salts of amines) were added at this temperature. Then, the mixture was cooled to 0 °C and the

arenesulfonyl chloride **5** (1.2 equiv.) was added in one portion. The reaction was allowed to slowly warm up to room temperature over 15 h (without removing the cooling bath after the addition of **5**). The progress of the reaction was monitored by TLC analysis. Then, the solvents were removed under vacuum. The residue was purified by column chromatography (silica gel, heptane:EtOAc).

General Procedure 3 (GP3, Protection of hindered primary amines and secondary amines):

The corresponding amine **1** (1.0 equiv.) was dissolved in DCM (0.1M) at room temperature (18-25 °C). DMAP (20 mol%) and Et_3N (2.0 equiv. for free amines, 3.0 equiv for hydrochloride salts of amines) were added at this temperature. Then, the mixture was cooled to 0 °C and the arenesulfonyl chloride **5** (1.2 equiv.) was added in one portion. The reaction was allowed to slowly warm up to room temperature over 14-20 h (without removing the cooling bath right after the addition of **5**). The progress of the reaction was monitored by TLC analysis. Then, the solvents were removed under vacuum. The residue was purified by column chromatography (silica gel, heptane:EtOAc).

General Procedure 4 (GP4, Protection of anilines):

The corresponding amine **1** (1.0 equiv.) was dissolved in DCM (0.1M) at room temperature (18-25 °C). DMAP (20 mol%) and 2,6-lutidine (2.0 equiv.) were added at this temperature. Then, the mixture was cooled to 0 °C and the arenesulfonyl chloride **5** (1.2 equiv.) was added in one portion. The reaction was allowed to slowly warm up to room temperature over 14-20 h (without removing the cooling bath right after the addition of **5**). The progress of the reaction was monitored by TLC analysis. Then, the solvents were removed under vacuum. Then, the solvents were removed under vacuum. The residue was purified by column chromatography (silica gel, heptane:EtOAc).

2.3.2. General Procedure for the deprotection of sulfonamides

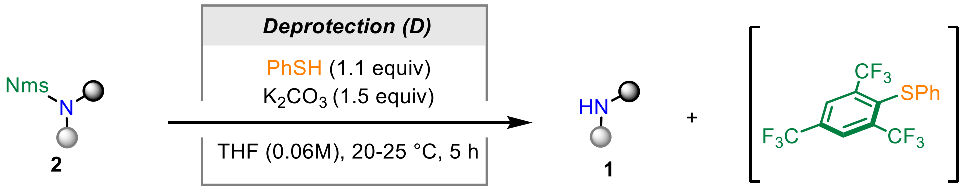

General Procedure 5 (GP5, Deprotection of amines):

Thiophenol (1.0 equiv.) was added to a mixture of the sulfonamide **2** (1.0 equiv.) and K_2CO_3 (1.5 equiv.) in dry THF (0.06M) at room temperature (18-25 °C). The reaction mixture was stirred at the same temperature for 3-12 h. The progress of the reaction was monitored by TLC analysis. Then, the solvents were evaporated and the crude reaction yield determined using CH_2Br_2 or mesitylene as internal standard. For certain amines (non volatile, non-commercial) a further purification was applied to purely isolate the desired unprotected amine. Following work-up techniques have been used:

Purification procedure for amine isolation

via hydrochloride salt (PP1):

The crude mixture was filtered through a short pad of celite using EtOAc as the solvent. The filtrate was then concentrated under reduced pressure and the residue dissolved in DCM (0.1M). Then, HCl (2M in Et_2O, 10.0 equiv.) was added and the mixture stirred at room temperature (18-25 °C) for 30 min. The solvent was again evaporated under reduced pressure and the remaining solid was triturated with EtOAc (x2).

via acid-base extraction (PP2):

DCM and HCl (1M in water) were added to the crude mixture. The layers were separated and the organic layer was extracted with HCl (1M in water, x2). The combined aqueous phases were basified with 1M NaOH until pH 9-10 was reached. Then, the aqueous phase was extracted with DCM (x5) and the organic phases were combined. After drying over $MgSO_4$, filtration and evaporation of solvent the desired free amine was obtained.

via column chromatography (PP3):

The crude material was subjected to column chromatography using silica gel and a suitable eluent gradient [typically DCM to DCM/MeOH (9:1) with 3% concentrated NH_4OH].

2.4. Synthesis of starting materials

Butyl (*R*)-2-(4-(4-(aminomethyl)-2-fluorophenoxy)phenoxy)propanoate (1p)

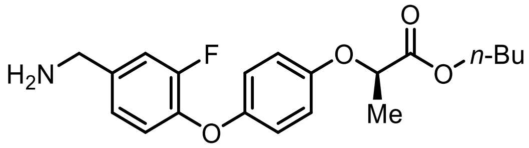

Cyhalofop-butyl (366.5 mg, 1.0 mmol, 1.0 equiv.) and Pd/C (10 % in charcoal, 105.5 mg, 0.10 mmol, 0.1 equiv.) were placed in a Schlenk flask, which was evacuated and backfilled with argon three times. Dry MeOH (20 mL, 0.5M) and a concentrated aqueous solution of HCl (0.32 ml, 3.8 mmol, 3.8 equiv.) were added. A hydrogen balloon was attached, and hydrogen gas was bubbled through the solvent for 1 h. After 2.5 h the mixture was filtered through a pad of celite and washed with MeOH (40 mL). The solvent was removed under reduced pressure. Purification on silica-gel (DCM:EtOAc:MeOH:NEt_3 = 3:1:0.04:0.04 to 1:2:0.06:0.06). Purification on silica-gel (DCM:MeOH:NEt_3 = 100:0:1 to 50:1:0.5). Amine **1p** was obtained as a colourless oil (90.0 mg, 0.25 mmol, 25%).

^{1}H NMR (700 MHz, $CDCl_3$): δ 7.14 (dd, *J* = 11.5, 1.9 Hz, 1H), 6.99 (dd, *J* = 8.3, 0.9 Hz, 1H), 6.94 – 6.87 (m, 3H), 6.85 – 6.81 (m, 2H), 4.68 (q, *J* = 6.8 Hz, 1H), 4.19 – 4.10 (m, 2H), 3.84 (s, 2H), 1.62 – 1.56 (m, 5H), 1.53 (br, 2H), 1.34 – 1.27 (m, 2H), 0.89 (t, *J* = 7.4 Hz, 3H) ppm.

^{13}C NMR (176 MHz, $CDCl_3$): δ 172.4, 154.2 (d, *J* = 248.6 Hz), 153.7, 151.7, 143.4 (d, *J* = 11.5 Hz), 140.0 (d, *J* = 5.6 Hz), 123.0 (d, *J* = 3.4 Hz), 120.9 (d, *J* = 1.3 Hz), 118.9, 116.4, 115.7 (d, *J* = 18.4 Hz), 73.4, 65.2, 45.7, 30.6, 19.1, 18.8, 13.7 ppm.

^{19}F NMR (659 MHz, $CDCl_3$): δ -131.6 ppm.

IR (neat): ν_{max} 2961, 2935, 2873, 1753, 1500, 1275, 1212 cm^{-1}.

HRMS (ESI$^+$): exact mass calculated for $[M+H]^+$ ($C_{20}H_{25}FNO_4^+$) requires *m/z* 362.1762, found *m/z* 362.1761.

The spectroscopic data matched the data reported in the literature for this compound.[2]

2.5. Protection of amines (2a-2al)

2.5.1. Ammonia surrogate (6)

2,4,6-tris(Trifluoromethyl)benzenesulfonamide (6)

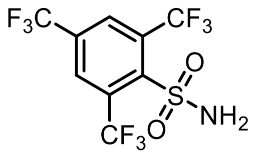

Sulfonyl chloride **5** (761 mg, 2.00 mmol, 1.0 equiv.) was dissolved in acetone (12 mL, 0.17M). Then concentrated ammonia solution (25 mol%, 0.6 mL, 7.7 mmol, 3.8 equiv.) was added. A white precipitate formed immediately. The reaction was continued to stir at room temperature for 16 h. Then the crude material was transferred to a separating funnel with EtOAc. A saturated solution of NH_4Cl was added and the aqueous phase was extracted twice with ethyl acetate. The combined organic phases were dried over $MgSO_4$ and the solvent was evaporated under reduced pressure. The desired primary sulfonamide was obtained as a slightly yellowish solid (687 mg, 1.90 mmol, 95%).

^{1}H NMR (600 MHz, Acetone-d6): δ 8.54 (s, 2H), 7.41 (s, 2H) ppm.

^{13}C NMR (151 MHz, Acetone-d6): δ 148.2, 134.14 (q, *J* = 34.8 Hz), 132.81 (q, *J* = 33.7 Hz), 123.59 (q, *J* = 274.5 Hz), 123.3 (q, *J* = 272.6 Hz) ppm.

^{19}F NMR (376 MHz, Acetone-d6): δ -55.3 (6F), -64.0 (3F) ppm.

IR (neat): ν_{max} **:** 1380, 1288, 1203, 1185, 1162, 1139, 1124, 1081, 913, 708, 685 cm^{-1}.

HRMS (ESI$^+$): exact mass calculated for $[M+H]^+$ ($C_9H_4F_9NNaO_2S^+$) requires m/z 383.9717, found m/z 383.9711.

2.5.2 Primary amines (2a-2x)

***N*-(Cyclohexyl)-2,4,6-tris(trifluoromethyl)benzenesulfonamide (2a)**

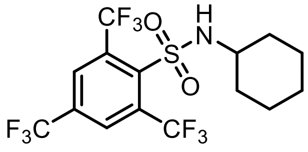

Following GP1 using cyclohexylamine (9.92 µL, 11.4 mg, 0.1 mmol), sulfonamide **2a** was obtained as a white solid (43.1 mg, 0.097 mmol, 97%).

^{1}H NMR (400 MHz, $CDCl_3$): δ 8.29 (s, 2H), 4.80 (d, *J* = 8.3 Hz, 1H), 3.68 – 3.65 (m, 1H), 1.94 – 1.81 (m, 2H), 1.75 – 1.66 (m, 2H), 1.61 – 1.55 (m, 1H), 1.38 – 1.08 (m, 5H) ppm.

^{13}C NMR (151 MHz, $CDCl_3$): δ 146.8, 134.0 (q, *J* = 35.1 Hz), 132.5 (q, *J* = 33.6 Hz), 129.5 (m), 122.4 (q, *J* = 275.0 Hz), 122.0 (q, *J* = 273.5 Hz), 54.5, 34.5, 25.1, 25.0 ppm.

^{19}F NMR (565 MHz, $CDCl_3$): δ -54.6 (6F), -63.6 (3F) ppm.

IR (neat): ν_{max} 3340, 2970, 2857, 1435, 1373, 1275, 1137, 1065, 708 cm^{-1}.

HRMS (ESI$^+$): exact mass calculated for $[M+Na]^+$ ($C_{15}H_{14}F_9NO_2SNa^+$) requires *m/z* 466.0499, found *m/z* 466.0494.

***N*-Pentyl-2,4,6-tris(trifluoromethyl)benzenesulfonamide (2b)**

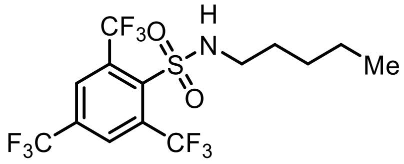

Following GP1 using *n*-amylamine (174 µL, 1.5 mmol), sulfonamide **2b** was obtained as a white solid (640 mg, 1.48 mmol, 99%).

^{1}H NMR (400 MHz, $CDCl_3$): δ 8.29 (s, 2H), 4.99 (t, *J* = 5.1 Hz, 1H), 3.20 (dd, *J* = 13.4, 6.7 Hz, 2H), 1.61 – 1.49 (m, 2H), 1.36 – 1.23 (m, 4H), 0.88 (t, *J* = 6.8 Hz, 3H) ppm.

^{13}C NMR (176 MHz, $CDCl_3$): δ 145.8, 134.1 (q, *J* = 35.1 Hz), 132.6 (q, *J* = 33.7 Hz), 129.5 – 129.3 (m), 122.35 (q, *J* = 275.5 Hz), 44.2, 30.0, 28.7, 22.3, 13.9 ppm.

^{19}F NMR (376 MHz, $CDCl_3$): δ -54.8 (6F), -63.7 (3F) ppm.

IR (neat): ν_{max} 1276, 1194, 1153, 1119 cm^{-1}.

HRMS (ESI$^+$): exact mass calculated for $[M+Na]^+$ ($C_{14}H_{14}F_9NNaO_2S^+$) requires m/z 454.0499, found m/z 454.0502.

N-(But-3-en-1-yl)-2,4,6-tris(trifluoromethyl)benzenesulfonamide (2c)

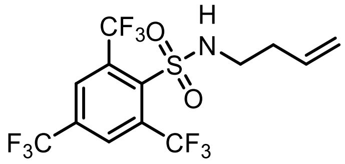

Following GP1 using but-3-en-1-amine hydrochloride (95 mg, 0.88 mmol), sulfonamide **2c** was obtained as a white solid (361 mg, 0.87 mmol, 98%).

^{1}H NMR (700 MHz, CDCl$_3$): δ 8.30 (s, 2H), 5.74 – 5.63 (m, 1H), 5.16 – 5.12 (m, 2H), 5.01 (t, *J* = 5.0 Hz, 1H), 3.30 (q, *J* = 6.4 Hz, 2H), 2.34 (q, *J* = 6.7 Hz, 2H) ppm.

^{13}C NMR (176 MHz, CDCl$_3$): δ 145.7, 134.2 (q, *J* = 35.2 Hz), 133.8, 132.6 (q, *J* = 33.6 Hz), 129.5, 122.3 (q, *J* = 275.5 Hz), 122.0 (q, *J* = 275.3 Hz), 119.1, 42.8, 34.4 ppm.

^{19}F NMR (659 MHz, CDCl$_3$): δ -54.7 (6F), -63.6 (3F) ppm.

IR (neat): ν_{max} 1272, 1181, 1136, 1111, 1066, 917, 708 cm^{-1}.

HRMS (ESI$^+$): exact mass calculated for [M+H]$^+$ ($C_{13}H_{10}F_9NO_2S^+$) requires m/z 438.0181, found m/z 438.0184.

N-(((1*S*,2*R*,5*S*)-6,6-Dimethylbicyclo[3.1.1]heptan-2-yl)methyl)-2,4,6-tris(trifluoromethyl)benzenesulfonamide (2d)

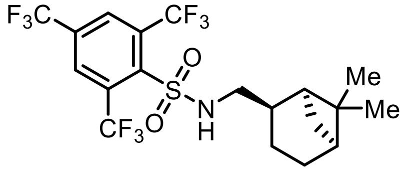

Following GP1 using (-)-*cis*-myrtanylamine (0.1 mmol, 15.3 mg, 16.4 µL), sulfonamide **2d** was obtained as a white solid (43.6 mg, 0.0877 mmol, 88%).

^{1}H NMR (400 MHz, CDCl$_3$): δ 8.29 (s, 2H), 4.93 (t, *J* = 5.3 Hz, 1H), 3.24 – 3.16 (m, 2H), 2.42 – 2.33 (m, 1H), 2.27 – 2.16 (m, 1H), 2.03 – 1.84 (m, 5H), 1.50 – 1.39 (m, 1H), 1.18 (s, 3H), 0.97 (s, 3H), 0.90 (d, *J* = 9.8 Hz, 1H) ppm.

^{13}C NMR (101 MHz, CDCl$_3$): δ 145.9, 134.1 (q, *J* = 35.2 Hz), 132.6 (q, *J* = 33.5 Hz), 129.1 (m), 122.4 (q, *J* = 274.5 Hz), 122.1 (q, *J* = 273.4 Hz), 49.3, 43.5, 41.9, 41.3, 38.7, 33.1, 27.9, 25.9, 23.1, 19.6 ppm.

^{19}F NMR (377 MHz, CDCl$_3$): δ -54.7 (6F), -63.6 (3F) ppm.

IR (neat): ν_{max} 2920, 1428, 1270, 1178, 1145, 1115, 918, 863, 707, 686, 617, 573 cm^{-1}.

HRMS (ESI$^+$): exact mass calculated for [M+H]$^+$ ($C_{19}H_{21}F_9NO_2S^+$) requires m/z 498.1144, m/z found: 498.1149.

N-(3,4-Dimethoxyphenethyl)-2,4,6-tris(trifluoromethyl)benzenesulfonamide (2e)

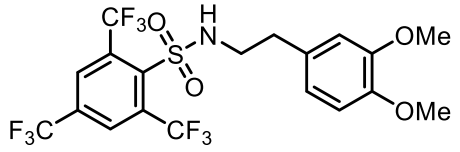

Following GP1 using 3,4-dimethoxybenzeneethanamine (16.9 µL, 18.1 mg, 0.1 mmol), sulfonamide **2e** was obtained as a white solid (46.1 mg, 0.0877 mmol, 88%).

^{1}H NMR (400 MHz, CDCl$_3$): δ 8.27 (s, 2H), 6.78 (d, *J* = 8.0 Hz, 1H), 6.72 – 6.64 (m, 2H), 4.94 (t, *J* = 5.6 Hz, 1H), 3.86 (s, 3H), 3.85 (s, 3H), 3.48 – 3.45 (m, 2H), 2.83 (t, *J* = 6.8 Hz, 2H) ppm.

^{13}C NMR (151 MHz, CDCl$_3$): δ 149.3, 148.2, 145.6, 134.1 (q, *J* = 35.0 Hz), 132.5 (q, *J* = 33.6 Hz), 129.6, 129.4 (m), 122.3 (q, *J* = 275.5 Hz), 122.0 (q, *J* = 273.4 Hz), 120.9, 112.0, 111.6, 56.1, 56.0, 45.3, 36.3 ppm.

^{19}F NMR (659 MHz, CDCl$_3$): δ -54.7 (6F), -63.6 (3F) ppm.

IR (neat): ν_{max} 3374, 2918, 2842, 1516, 1420, 1365, 1177, 1154, 915, 704, 686, 577 cm^{-1}.

HRMS (ESI$^+$): exact mass calculated for [M+Na]$^+$ ($C_{19}H_{16}O_4NSF_9Na^+$) requires *m/z* 548.0548, found *m/z* 548.0549.

N-[3-(2-Methylpiperidin-1-yl)propyl]-2,4,6-tris(trifluoromethyl)benzenesulfonamide (2f)

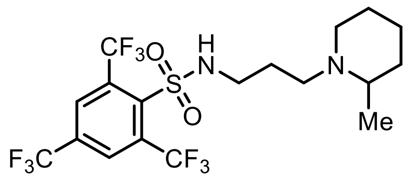

Following an adapted version of GP1 (the eluent for column chromatography purification was 2:1:0.3 heptane:EtOAc:MeOH) using 1-(3-aminopropyl)-2-pipecoline (15.6 mg, 0.1 mmol), sulfonamide **2f** was obtained as a yellow solid (41.1 mg, 0.0821 mmol, 82%).

^{1}H NMR (400 MHz, CDCl$_3$): δ 8.25 (s, 2H), 3.48 – 3.40 (m, 1H), 3.28 – 3.17 (m, 1H), 3.01 – 2.90 (m, 2H), 2.45 – 2.30 (m, 2H), 2.10 – 2.04 (m, 1H), 1.93 – 1.82 (m, 1H), 1.72 – 1.55 (m, 5H), 1.48 – 1.32 (m, 2H), 1.11 (t, *J* = 6.3 Hz, 3H) ppm. *The N-H was not observed in ^{1}H NMR.

^{13}C NMR (151 MHz, CDCl$_3$): δ 146.9, 133.5 (q, *J* = 34.9 Hz), 132.6 (q, *J* = 33.6 Hz), 129.2 (m), 122.5 (q, *J* = 275.0 Hz), 122.2 (q, *J* = 273.0 Hz), 57.1, 54.2, 51.3, 45.3, 33.7, 29.8, 25.1, 24.6, 14.2 ppm.

^{19}F NMR (565 MHz, CDCl$_3$): δ -54.5 (6F), -63.5 (3F) ppm.

IR (neat): ν_{max} 2961, 2929, 2853, 1352, 1280, 1147, 1112, 914, 684, 614 cm^{-1}.

HRMS (ESI$^+$): exact mass calculated for [M+H]$^+$ ($C_{18}H_{22}F_9N_2O_2S^+$) 501.1253, found *m/z* 501.1251.

***tert*-Butyl [3-((2,4,6-tris(trifluoromethyl)phenyl)sulfonamido)propyl]carbamate (2g)**

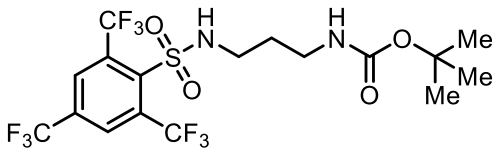

Following GP1 using *N*-Boc-1,3-diaminopropane (34.9 µL, 34.8 mg, 0.2 mmol), sulfonamide **2g** was obtained as a white solid (94.9 mg, 0.183 mmol, 92%).

^{1}H NMR (400 MHz, $CDCl_3$): δ 8.27 (s, 2H), 6.86-6.73 (m, 1H), 4.76 – 4.64 (m, 1H), 3.37 – 3.25 (m, 2H), 3.23 – 3.16 (m, 2H), 1.74 – 1.61 (m, 2H), 1.42 (s, 9H) ppm.

^{13}C NMR (176 MHz, $CDCl_3$): δ 157.5, 146.4, 133.6 (q, *J* = 35.0 Hz), 132.6 (q, *J* = 33.4 Hz), 129.3 (m), 122.3 (q, *J* = 275.5 Hz), 122.1 (q, *J* = 273.5 Hz), 80.2, 40.4, 36.6, 31.8, 28.4 ppm.

^{19}F NMR (659 MHz, $CDCl_3$): δ -54.8 (6F), -63.6 (3F) ppm.

IR (neat): ν_{max} 3345, 2990, 2932, 1685, 1522, 1366, 1156, 956, 740, 707, 615 cm^{-1}.

HRMS (ESI$^+$): exact mass calculated for [M+Na]$^+$ ($C_{17}H_{19}F_9N_2O_4SNa^+$) requires *m/z* 541.0814, found *m/z* 541.0818.

***N*-[2-(1*H*-Indol-3-yl)ethyl]-2,4,6-tris(trifluoromethyl)benzenesulfonamide (2h)**

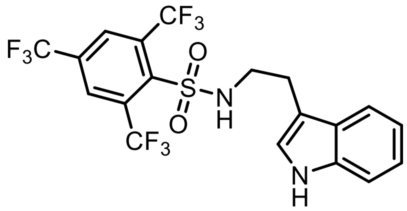

Following GP1 using tryptamine (32.0 mg, 0.2 mmol), sulfonamide **2h** was obtained as a white solid (92.9 mg, 0.184 mmol, 92%).

^{1}H NMR (700 MHz, $CDCl_3$): δ 8.18 (s, 2H), 8.03 (br s, 1H), 7.44 (d, *J* = 7.9 Hz, 1H), 7.33 (d, *J* = 8.2 Hz, 1H), 7.20 – 7.16 (m, 1H), 7.07 – 7.04 (m, 2H), 5.02 – 4.94 (m, 1H), 3.56 (q, *J* = 6.3 Hz, 2H), 3.05 (t, *J* = 6.6 Hz, 2H) ppm.

^{13}C NMR (176 MHz, $CDCl_3$): δ 145.2, 136.5, 134.0 (q, *J* = 35.0 Hz), 132.4 (q, *J* = 33.5 Hz), 129.2 (m), 126.8, 123.0, 122.6, 122.4 (q, *J* = 275.0 Hz), 121.9 (q, *J* = 285.3 Hz), 119.8, 119.8, 118.5, 111.5, 111.4, 111.3, 43.8, 26.2 ppm.

^{19}F NMR (659 MHz, $CDCl_3$): δ -54.7 (6F), -63.5 (3F) ppm.

IR (neat): ν_{max} 3392, 3357, 2955, 1286, 1186, 1142, 933, 818, 644 cm^{-1}.

HRMS (ESI$^+$): exact mass calculated for [M+Na]$^+$ ($C_{19}H_{13}F_9N_2O_2SNa^+$) requires *m/z* 527.0446, found *m/z* 527.0449.

N-(Pyridin-2-ylmethyl)-2,4,6-tris(trifluoromethyl)benzenesulfonamide (2i)

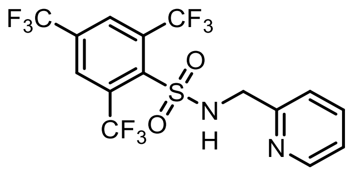

Following GP2 using pyridin-2-ylmethanamine (41.2 µL, 0.4 mmol), sulfonamide **2i** was obtained as a white solid (167 mg, 0.37 mmol, 92%).

^{1}H NMR (400 MHz, CDCl$_3$): δ 8.43 (d, *J* = 4.7 Hz, 1H), 8.28 (s, 2H), 7.66 (td, *J* = 7.7, 1.3 Hz, 1H), 7.24 – 7.17 (m, 2H), 6.87 (s, 1H), 4.52 (s, 2H) ppm.

^{13}C NMR (101 MHz, CDCl$_3$): δ 154.1, 149.0, 145.6, 137.1, 134.0 (q, *J* = 35.0 Hz), 132.7 (q, *J* = 33.6 Hz), 129.6 – 129.1 (m), 123.0, 122.3 (q, *J* = 275.4 Hz), 122.1 (q, *J* = 273.5 Hz), 121.8, 47.4 ppm.

^{19}F NMR (376 MHz, CDCl$_3$): δ -54.8 (6F), -63.6 (3F).

IR (neat): ν_{max} 1357, 1284, 1195, 1177, 1143 cm^{-1}.

HRMS (ESI$^+$): exact mass calculated for [M+H]$^+$ ($C_{15}H_{10}F_9N_2O_2S^+$) requires m/z 453.0314, found m/z 453.0305.

N-(Furan-2-ylmethyl)-2,4,6-tris(trifluoromethyl)benzenesulfonamide (2j)

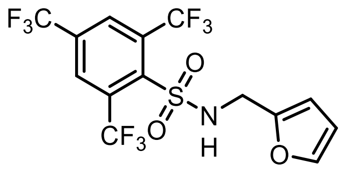

Following GP2 using furan-2-ylmethanamine (17.7 µL, 0.2 mmol), sulfonamide **2j** was obtained as a white solid (74.6 mg, 0.17 mmol, 85%).

^{1}H NMR (400 MHz, CDCl$_3$): δ 8.26 (s, 2H), 7.23 – 7.22 (m, 1H), 6.22 (dd, *J* = 3.2, 1.9 Hz, 1H), 6.18 (d, *J* = 3.2 Hz, 1H), 5.36 (s, 1H), 4.43 (s, 2H) ppm.

^{13}C NMR (101 MHz, CDCl$_3$): δ 149.1, 145.5, 143.0, 134.19 (q, *J* = 35.1 Hz), 132.6 (q, *J* = 33.6 Hz), 129.5 – 129.2 (m), 122.3 (q, *J* = 275.5 Hz), 122.0 (q, *J* = 273.5 Hz), 110.7, 109.0, 40.6 ppm.

^{19}F NMR (376 MHz, CDCl$_3$): δ -54.7 (6F), -63.7 (3F) ppm.

IR (neat): ν_{max} 1357, 1284, 1195, 1177, 1143 cm^{-1}.

HRMS (ESI$^+$): exact mass calculated for [M+Na]$^+$ ($C_{13}H_8F_9NNaO_2S^+$) requires m/z 463.9973, found m/z 463.9962.

N-(Thiophen-2-ylmethyl)-2,4,6-tris(trifluoromethyl)benzenesulfonamide (2k)

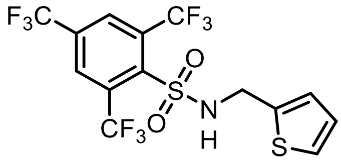

Following GP2 using thiophen-2-ylmethanamine (20.5 µL, 0.2 mmol), sulfonamide **2k** was obtained as a white solid (76.8 mg, 0.17 mmol, 84%).

^{1}H NMR (400 MHz, $CDCl_3$): δ 8.29 (s, 2H), 7.23 (dd, *J* = 5.1, 1.2 Hz, 1H), 6.97 (d, *J* = 3.0 Hz, 1H), 6.92 (dd, *J* = 5.1, 3.5 Hz, 1H), 5.29 (s, 1H), 4.61 (s, 2H) ppm.

^{13}C NMR (101 MHz, $CDCl_3$): δ 145.6, 138.2, 134.3 (q, *J* = 35.2 Hz), 132.6 (q, *J* = 33.6 Hz), 130.0 – 128.9 (m), 127.5, 127.3, 126.6, 122.3 (q, *J* = 275.5 Hz), 42.6 ppm.

^{19}F NMR (376 MHz, $CDCl_3$): δ -54.7 (6F), -63.6 (3F) ppm.

IR (neat): ν_{max} 1715, 1359, 1223, 1197, 1147 cm^{-1}.

HRMS (ESI$^+$): exact mass calculated for $[M+Na]^+$ ($C_{14}H_8F_9NNaO_2S^+$) requires m/z 479.9745, found m/z 479.9738.

N-Benzyl-2,4,6-tris(trifluoromethyl)benzenesulfonamide (2l)

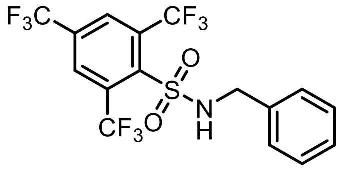

Following GP2 using benzylamine (22.4 µL, 22.0 mg, 0.205 mmol), sulfonamide **2l** was obtained as a white solid (90.5 mg, 0.201 mmol, 98%).

^{1}H NMR (400 MHz, $CDCl_3$): δ 8.29 (s, 2H), 7.37 – 7.26 (m, 5H), 5.22 (s, 1H), 4.41 (d, *J* = 5.3 Hz, 2H) ppm.

^{13}C NMR (151 MHz, $CDCl_3$): δ 145.70, 135.8, 134.3 (q, *J* = 34.2 Hz), 132.6 (q, *J* = 33.6 Hz), 129.4 (m), 129.1, 128.5, 128.2, 122.3 (q, *J* = 275.9 Hz), 122.0 (q, *J* = 273.3 Hz), 122.0 (m), 48.1 ppm.

^{19}F NMR (565 MHz, $CDCl_3$): δ -54.7 (6F), -63.6 (3F) ppm.

IR (neat): ν_{max} 3357, 1357, 1284, 1178, 1138, 1118, 1065, 914, 706, 616 cm^{-1}.

HRMS (ESI$^+$): exact mass calculated for $[M+Na]^+$ ($C_{16}H_{10}F_9NO_2SNa^+$) 474.0186, found *m/z* 474.0184.

N-(4-Cyanobenzyl)-2,4,6-tris(trifluoromethyl)benzenesulfonamide (2m)

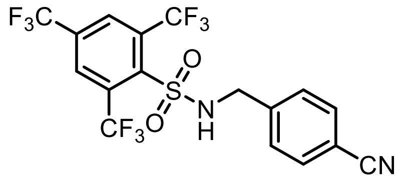

Following GP2 using 4-cyanobenzylamine hydrochloride (16.9 mg, 0.1 mmol), sulfonamide **2m** was obtained as a yellow solid (44.6 mg, 0.094 mmol, 94%).

^{1}H NMR (700 MHz, $CDCl_3$): δ 8.32 (s, 2H), 7.65 (d, *J* = 8.2 Hz, 2H), 7.45 (d, *J* = 8.1 Hz, 2H), 5.38 (s, 1H), 4.48 (s, 2H) ppm.

^{13}C NMR (176 MHz, $CDCl_3$): δ 145.2, 141.5, 134.6 (q, *J* = 35.2 Hz), 132.8, 132.6 (q, *J* = 33.7 Hz), 129.6 (m), 128.5, 122.3 (q, *J* = 275.5 Hz), 121.9 (q, *J* = 273.3 Hz), 47.4 ppm.

^{19}F NMR (659 MHz, $CDCl_3$): δ -54.6 (6F), -63.6 (3F) ppm.

IR (neat): ν_{max} 3289, 1364, 1292, 1275, 1198, 1138, 1083, 915, 868, 710, 687 cm^{-1}.

HRMS (ESI^+): exact mass calculated for $[M+Na]^+$ ($C_{17}H_9F_9N_2O_2SNa^+$) 499.0133, found *m/z* 499.0135.

N-(4-Methoxybenzyl)-2,4,6-tris(trifluoromethyl)benzenesulfonamide (2n)

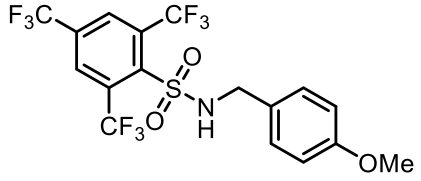

Following GP2 using 4-methoxybenzylamine (260.0 µL, 274.0 mg, 2.0 mmol), sulfonamide **2n** was obtained as a yellow solid (940.0 mg, 1.95 mmol, 98%).

^{1}H NMR (600 MHz, $CDCl_3$): δ 8.29 (s, 2H), 7.20 (d, *J* = 8.4 Hz, 2H), 6.84 (d, *J* = 8.4 Hz, 2H), 5.15 (s, 1H), 4.44 (s, 2H), 3.79 (s, 3H) ppm.

^{13}C NMR (151 MHz, $CDCl_3$): δ 159.8, 145.8, 134.2 (q, *J* = 35.2 Hz), 132.6 (q, *J* = 33.7 Hz), 129.7, 129.4 (m), 127.7, 122.3 (q, *J* = 275.5 Hz), 122.2 (q, *J* = 275.5 Hz), 114.4, 55.4, 47.6 ppm.

^{19}F NMR (565 MHz, $CDCl_3$): δ -54.6 (6F), -63.6 (3F) ppm.

IR (neat): ν_{max} 2967, 1610, 1515, 1355, 1290, 1187, 1141, 868, 684, 668, 464 cm^{-1}.

HRMS (ESI^+): exact mass calculated for $[M+Na]^+$ ($C_{17}H_{12}F_9NO_3SNa^+$) 504.0286, found *m/z* 504.0290.

(*R*)-*N*-(1-(Naphthalen-1-yl)ethyl)-2,4,6-tris(trifluoromethyl)benzenesulfonamide (2o)

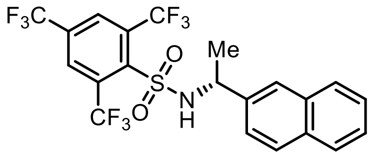

Following GP3 using (*R*)-(+)-1-(1-naphtyl)ethylamine (855.8 mg, 5.0 mmol), sulfonamide **2o** was obtained as a white solid (2.123 g, 4.12 mmol, 84%).

^{1}H NMR (700 MHz, CDCl$_3$): δ 8.08 (s, 2H), 7.96 (dd, *J* = 7.7, 6.2 Hz, 1H), 7.84 – 7.79 (m, 1H), 7.76 – 7.70 (m, 1H), 7.50 – 7.43 (m, 2H), 7.40 – 7.33 (m, 2H), 5.73 (app p, *J* = 7.0 Hz, 1H), 5.41 (d, *J* = 8.1 Hz, 1H), 1.68 (d, *J* = 6.9 Hz, 3H) ppm.

^{13}C NMR (176 MHz, CDCl$_3$): δ 145.9, 136.9, 134.0, 133.9 (q, *J* = 35.0 Hz), 132.3 (q, *J* = 33.5 Hz), 130.03, 129.13, 128.94, 126.8, 126.1, 125.2, 122.8, 122.5, 122.2 (q, *J* = 274.0 Hz), 121.9 (q, *J* = 271.7 Hz), 50.9, 23.2 ppm.

^{19}F NMR (659 MHz, CDCl$_3$): δ -54.5 (6F), -63.6 (3F) ppm.

IR (neat): ν_{max} 3326, 3105, 3055, 2986, 1425, 1366, 1269, 1192, 1138, 1045, 739 cm^{-1}.

HRMS (ESI$^+$): exact mass calculated for [M+Na]$^+$ ($C_{21}H_{14}F_9NO_2SNa^+$) requires *m/z* 538.0494, found *m/z* 538.0494.

Determination of enantiopurity. Method description: Lux-Cellulose 1 (Chiralcel OD-H) 250 x 4.6 mm, particle size 5 µm, solvent system: *n*-heptane+0.1*i*-PrOH/*i*-PrOH 9:1; flow 1 mL/min, 25 °C. Peak area 0.035% (Rt = 8.167 min), 99.965% (Rt = 10.213 min): 99.9% *ee*.

Enantiopure compound:

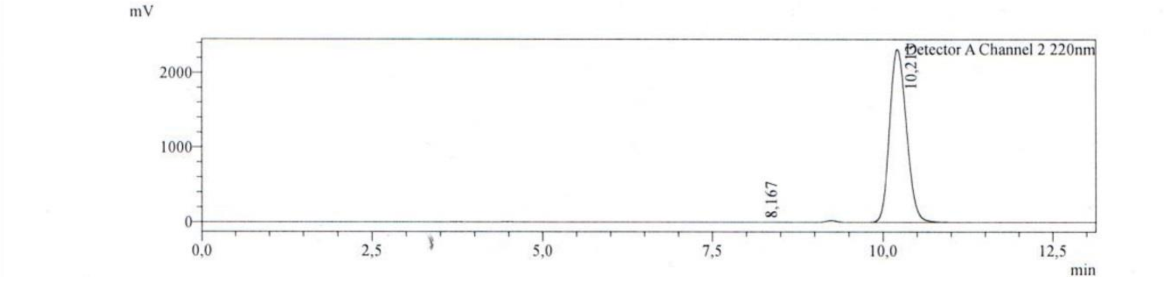

Racemic standard:

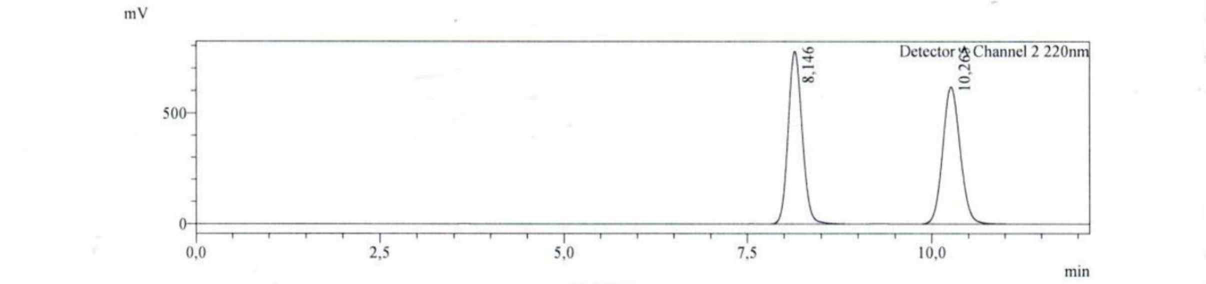

Butyl (*R*)-2-(4-(2-fluoro-4-(((2,4,6-tris(trifluoromethyl)phenyl)sulfonamido)methyl)phenoxy)phenoxy)propanoate (2p)

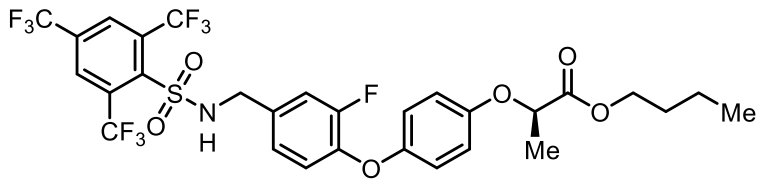

Following an adapted version of GP3 [addition of NmsCl was carried out at rt (20-25 °C)] using amine **1p** (37.3 mg, 0.10 mmol), sulfonamide **2p** was obtained as a yellowish solid (51.3 mg, 0.073 mmol, 73%).

^{1}H NMR (600 MHz, $CDCl_3$): δ 8.31 (s, 2H), 7.11 (dd, *J* = 11.0, 1.6 Hz, 1H), 6.98 (d, *J* = 8.3 Hz, 1H), 6.93 – 6.81 (m, 5H), 5.34 (s, 1H), 4.70 (q, *J* = 6.8 Hz, 1H), 4.35 (s, 2H), 4.21 – 4.11 (m, 2H), 1.64 – 1.55 (m, 5H), 1.31 (app dq, *J* = 14.8, 7.3 Hz, 2H), 0.90 (t, *J* = 7.4 Hz, 3H) ppm.

^{13}C NMR (151 MHz, $CDCl_3$): δ 172.4, 154.2, 153.7 (d, *J* = 251.5 Hz), 150.8, 145.6, 145.3 (d, *J* = 11.0 Hz), 134.4 (q, *J* = 35.2 Hz), 132.6 (q, *J* = 33.7 Hz), 132.0 (d, *J* = 6.1 Hz), 129.5, 124.2 (d, *J* = 3.2 Hz), 122.3 (q, *J* = 275.7 Hz), 122.0 (q, *J* = 273.1), 120.5, 119.6, 116.8 (d, *J* = 18.8 Hz), 116.5, 73.4, 65.3, 47.1, 30.7, 19.1, 18.7, 13.7 ppm.

^{19}F NMR (565 MHz, $CDCl_3$): δ -54.7 (6F), -63.6 (3F), -130.7 (t, *J* = 9.7 Hz, 1F) ppm.

IR (neat): ν_{max} 2963, 2935, 2900, 2876, 1736, 1500, 1271, 1194, 1135 cm^{-1}.

HRMS (ESI$^+$): exact mass calculated for $[M+Na]^+$ ($C_{29}H_{25}F_{10}NO_6SNa^+$) requires *m/z* 728.1141, found *m/z* 728.1135.

***N*-(*tert*-Butyl)-2,4,6-tris(trifluoromethyl)benzenesulfonamide (2q)**

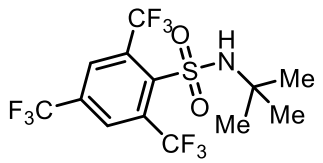

Following GP3 using *tert*-butylamine (19.6 µL, 13.5 mg, 0.185 mmol), sulfonamide **2q** was obtained as a white solid (63.8 mg, 0.152 mmol, 83%).

^{1}H NMR (400 MHz, $CDCl_3$): δ 8.26 (s, 2H), 5.03 (br s, 1H), 1.27 (s, 9H) ppm.

^{13}C NMR (176 MHz, $CDCl_3$): δ 148.1, 133.7 (q, *J* = 35.2 Hz), 132.0 (q, *J* = 33.4 Hz), 129.3 (m), 122.4 (q, *J* = 275.7 Hz), 122.0 (q, *J* = 273.3 Hz), 57.4, 29.7 ppm.

^{19}F NMR (376 MHz, $CDCl_3$): δ -53.9 (6F), -63.5 (3F) ppm.

IR (neat): ν_{max} 3301, 3095, 2977, 1294, 1265, 1131, 1087, 995, 706, 628, 463 cm^{-1}.

HRMS (ESI$^+$): exact mass calculated for $[M+Na]^+$ ($C_{13}H_{12}F_9NO_2SNa^+$) requires *m/z* 440.0337, found *m/z* 440.0322.

N-((1S,2R,5S)-2-Isopropyl-5-methylcyclohexyl)-2,4,6-tris(trifluoromethyl)benzenesulfonamide (2r)

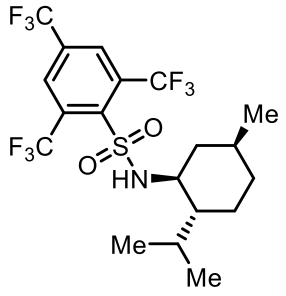

Following GP1 using mentylamine (0.1 mmol, 15.5 mg), sulfonamide **2r** was obtained as a white solid (45.0 mg, 0.090 mmol, 90%).

^{1}H NMR (400 MHz, $CDCl_3$): δ 8.31 (s, 2H), 4.57 (d, *J* = 9.3 Hz, 1H), 3.55 (qd, *J* = 10.7, 4.2 Hz, 1H), 2.06 (heptd, *J* = 6.9, 1.4 Hz, 1H), 1.81 – 1.63 (m, 3H), 1.50 – 1.32 (m, 1H), 1.17 – 0.99 (m, 2H), 0.94 – 0.88 (m, 1H), 0.91 (d, *J* = 7.0 Hz, 3H), 087 – 0.78 (m, 1H), 0.82 (d, *J* = 6.6 Hz, 3H), 0.75 (d, *J* = 6.9 Hz, 3H) ppm.

^{13}C NMR (101 MHz, $CDCl_3$): δ 146.9, 134.0 (q, *J* = 35.2 Hz), 132.5 (q, *J* = 33.4 Hz, 2C), 129.8-129.4 (m, 2C), 122.4 (q, *J* = 275.5 Hz, 2C), 122.1 (q, *J* = 273.6 Hz), 56.68, 48.9, 44.4, 34.2, 32.1, 26.3, 23.8, 22.1, 21.4, 15.5 ppm.

^{19}F NMR (377 MHz, $CDCl_3$): δ -54.7 (6F), -63.6 (3F) ppm.

IR (neat): ν_{max} 3418, 3358, 3111, 2958, 2930, 2873, 1432, 1368, 1271, 1181, 1143, 1119, 1083, 1051, 916, 862, 707, 686, 618 cm^{-1}.

HRMS (ESI$^+$): exact mass calculated for $[M+H]^+$ ($C_{19}H_{23}SO_2NF_9^+$) requires *m/z* 500.1306, found *m/z* 500.1206.

N-(4-Methoxyphenyl)-2,4,6-tris(trifluoromethyl)benzenesulfonamide (2s)

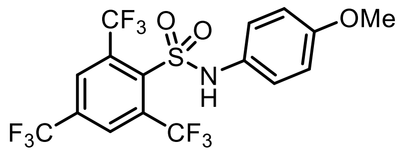

Following GP4 using *p*-anisidine (11.6 µL, 12.3 mg, 0.1 mmol), sulfonamide **2s** was obtained as a white solid (37.5 mg, 0.080 mmol, 80%).

^{1}H NMR (600 MHz, $CDCl_3$): δ 8.31 (s, 2H), 7.03 (d, *J* = 8.9 Hz, 2H), 6.81 (d, *J* = 8.9 Hz, 2H), 3.78 (s, 3H) ppm. *The N-H was not observed in ^{1}H NMR.

^{13}C NMR (176 MHz, $CDCl_3$): δ 159.3, 144.9, 134.5 (q, *J* = 35.0 Hz), 133.3 (q, *J* = 33.5 Hz), 129.6 (m), 127.6, 126.6, 122.2 (q, *J* = 274.7 Hz), 122.0 (q, *J* = 275.8 Hz), 114.7, 55.6 ppm.

^{19}F NMR (659 MHz, $CDCl_3$): δ -54.7 (6F), -63.6 (3F) ppm.

IR (neat): ν_{max} 3326, 3123, 2970, 1513, 1183, 1136, 918, 709, 685, 619, 461 cm^{-1}.

HRMS (ESI⁺): exact mass calculated for $[M+Na]^+$ ($C_{16}H_{10}F_9NO_3SNa^+$) requires *m/z* 490.0129, found *m/z* 490.0134.

2,4,6-Tris(trifluoromethyl)-*N*-(3,4,5-trimethoxyphenyl)benzenesulfonamide (2t)

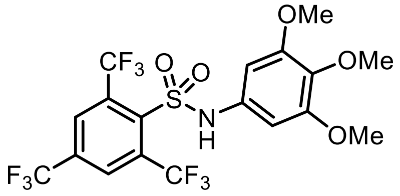

Following GP4 from 3,4,5-trimethoxyaniline (18.3 mg, 0.1 mmol), sulfonamide **2t** was obtained as a yellow solid (32.8 mg, 0.062 mmol, 62%).

^{1}H NMR (400 MHz, $CDCl_3$): δ 8.33 (s, 2H), 6.86 (br s, 1H), 6.32 (s, 2H), 3.80 (s, 3H), 3.75 (s, 6H) ppm.

^{13}C NMR (176 MHz, $CDCl_3$): δ 153.7, 144.7, 137.4, 134.7 (q, *J* = 35.2 Hz), 133.3 (q, *J* = 33.6 Hz), 130.0, 129.7 (m), 122.1 (q, *J* = 276.0 Hz), 121.9 (q, *J* = 273.8 Hz), 101.9, 61.1, 56.3 ppm.

^{19}F NMR (659 MHz, $CDCl_3$): δ -54.5 (6F), -63.6 (3F) ppm.

IR (neat): ν_{max} 3123, 2928, 2849, 1607, 1512, 1364, 1275, 1132, 1010, 985, 706, 565, 462 cm^{-1}.

HRMS (ESI⁺): exact mass calculated for $[M+H]^+$ ($C_{18}H_{15}F_9NO_5S^+$) requires *m/z* 528.0522, found *m/z* 528.0520.

***N*-(1*H*-Indol-5-yl)-2,4,6-tris(trifluoromethyl)benzenesulfonamide (2u)**

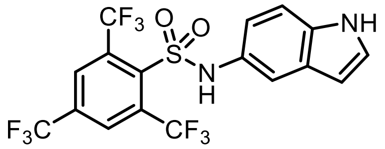

Following GP4 using 5-aminoindole (13.2 mg, 0.1 mmol), sulfonamide **2u** was obtained as a yellow solid (34.9 mg, 0.073 mmol, 73%).

^{1}H NMR (400 MHz, MeOD): δ 8.47 (s, 2H), 7.25 (d, *J* = 8.6 Hz, 1H), 7.22 (d, *J* = 3.1 Hz, 1H), 7.16 (d, *J* = 1.9 Hz, 1H), 6.76 (dd, *J* = 8.6, 2.0 Hz, 1H), 6.34 (d, *J* = 3.1 Hz, 1H) ppm. *The N-H were not observed in ^{1}H NMR.

^{13}C NMR (176 MHz, MeOD): δ 147.4, 136.2, 135.0 (q, *J* = 34.7 Hz), 134.1 (q, *J* = 33.7 Hz), 130.8 (m), 129.5, 126.9, 123.6 (q, *J* = 275.4 Hz), 120.1, 117.6, 112.4, 102.5 ppm.

^{19}F NMR (659 MHz, MeOD): δ -55.8 (6F), -65.0 (3F) ppm.

IR (neat): ν_{max} 3413, 2970, 1369, 1273, 1187, 1142, 965, 707, 638 cm^{-1}.

HRMS (ESI⁺): exact mass calculated for $[M+Na]^+$ ($C_{17}H_9F_9N_2O_2SNa^+$) requires *m/z* 499.0133, found *m/z* 499.0122.

Methyl ((2,4,6-tris(trifluoromethyl)phenyl)sulfonyl)-*L*-isoleucinate (2v)

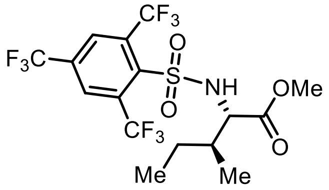

Following an adapted version of GP3 [addition of NmsCl was carried out at rt (20-25 °C)] using *L*-isoleucine methyl ester hydrochloride (148.7 mg, 0.80 mmol), sulfonamide **2v** was obtained as a white solid (338.9 mg, 0.69 mmol, 86%).

^{1}H NMR (700 MHz, $CDCl_3$): δ 8.31 (s, 2H), 5.46 (d, *J* = 9.8 Hz, 1H), 4.35 (dd, *J* = 10.0, 4.3 Hz, 1H), 3.60 (s, 3H), 2.04 – 1.96 (m, 1H), 1.35 (dqd, *J* = 14.8, 7.4, 4.5 Hz, 1H), 1.20 – 1.11 (m, 1H), 1.01 (d, *J* = 6.9 Hz, 3H), 0.92 (t, *J* = 7.4 Hz, 3H) ppm.

^{13}C NMR (176 MHz, $CDCl_3$): δ 171.7, 145.3, 134.3 (q, *J* = 35.2 Hz), 132.7 (q, *J* = 33.4 Hz), 129.6 – 129.4 (m), 122.3 (q, *J* = 275.2 Hz), 122.0 (q, *J* = 274.0 Hz), 61.4, 52.6, 38.3, 24.7, 15.8, 11.7 ppm.

^{19}F NMR (659 MHz, $CDCl_3$): δ -54.8 (6F), -63.6 (3F) ppm.

IR (neat): ν_{max} 3415, 2970, 2939, 2883, 1740, 1435, 1379, 1288, 1191, 1135, 708 cm^{-1}.

HRMS (ESI$^+$): exact mass calculated for $[M+Na]^+$ ($C_{16}H_{16}F_9NO_4SNa^+$) requires *m/z* 512.0554, found *m/z* 512.0538.

Benzyl ((2,4,6-tris(trifluoromethyl)phenyl)sulfonyl)-L-serinate (2w)

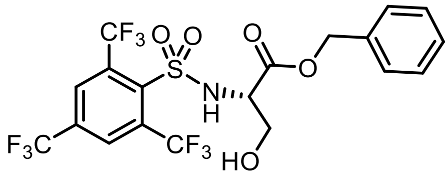

Following an adapted version of GP1 (eluent for the column chromatography was DCM to DCM/MeOH, 98:2) using benzyl-L-serinate hydrochloride (0.1 mmol, 23.2 mg), sulfonamide **2w** was obtained as a yellowish solid (49 mg, 0.0906 mmol, 91%).

^{1}H NMR (400 MHz, $CDCl_3$): δ 8.18 (s, 2H), 7.32 – 7.25 (m, 3H), 7.22 – 7.12 (m, 2H), 6.05 (s, 1H), 5.03 (s, 2H), 4.54 (s, 1H), 4.18 (dd, *J* = 10.9, 3.0 Hz, 1H), 3.97 (dd, *J* = 10.9, 3.2 Hz, 1H), 2.10 (s, 1H) ppm.

^{13}C NMR (101 MHz, $CDCl_3$): δ 169.6, 145.2, 134.6, 134.2 (q, *J* = 35.4 Hz), 132.5 (q, *J* = 33.4 Hz), 130.1 – 128.8 (m), 128.8, 128.4, 122.2 (q, *J* = 276.7 Hz), 122.0 (q, *J* = 274.7 Hz), 68.1, 63.9, 58.5 ppm.

^{19}F NMR (377 MHz, $CDCl_3$): δ -54.8 (6F), -63.5 (3F) ppm.

IR (neat): ν_{max} 3173, 1736, 1372, 1274, 1194, 1133, 1113, 1043, 974, 913, 867, 748, 708, 570, 462, 447, 429 cm^{-1}.

HRMS (ESI$^+$): exact mass calculated for $[M+Na]^+$ ($C_{19}H_{14}F_9NO_5SNa^+$) requires m/z 562.0341, m/z found: 562.0356.

Determination of enantiopurity. Method description: Chiralpak IC 250 x 4.6 mm, particle size 5 µm, solvent system: *n*-heptane+0.1*i*-PrOH/*i*-PrOH 92:8; flow 1 mL/min, 25 °C. Peak area 99.100% (Rt = 5.989 min), 0.900% (Rt = 7.223 min): 98.2% *ee*.

Enantiopure compound:

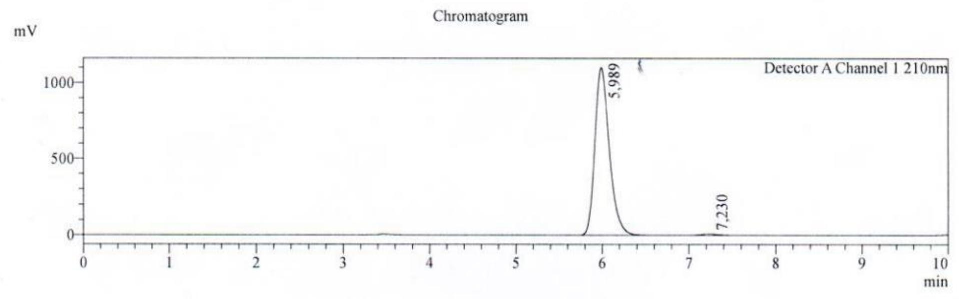

Racemic standard:

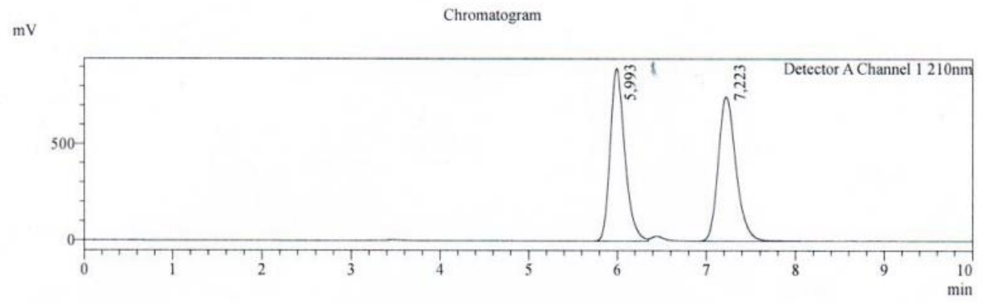

Methyl ((2,4,6-tris(trifluoromethyl)phenyl)sulfonyl)-*L*-serinate (2x)

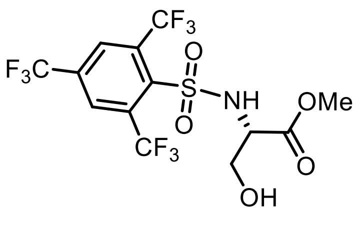

Following an adapted version of GP3 [addition of NmsCl was carried out at rt (20-25 °C)] using *L*-serine methyl ester hydrochloride (15.6 mg, 0.10 mmol), sulfonamide **2x** was obtained as a white solid (38.1 mg, 0.082 mmol, 82%).

^{1}H NMR (700 MHz, $CDCl_3$): δ 8.32 (s, 2H), 5.97 (s, 1H), 4.50 (t, *J* = 2.8 Hz, 1H), 4.14 (d, *J* = 10.5 Hz, 1H), 3.98 (d, *J* = 10.8 Hz, 1H), 3.67 (s, 3H), 1.82 (s, 1H) ppm.

^{13}C NMR (176 MHz, $CDCl_3$): δ 170.1, 145.3, 134.4 (q, *J* = 35.9 Hz), 132.7 (q, *J* = 33.3 Hz), 129.5, 122.3 (q, *J* = 277.7 Hz), 122.0 (q, *J* = 272.3 Hz), 63.8, 58.3, 53.2 ppm.

^{19}F NMR (376 MHz, $CDCl_3$): δ -54.8 (6F), -63.6 (3F) ppm.

IR (neat): ν_{max} 1737, 1369, 1293, 1197, 1142 cm^{-1}.

HRMS (ESI⁺): exact mass calculated for $[M+Na]^+$ ($C_{13}H_{10}F_9NO_5SNa^+$) requires *m/z* 486.0034, found *m/z* 486.0023.

3.5.3. Secondary amines (2y-2al)

Ethyl 1-((2,4,6-tris(trifluoromethyl)phenyl)sulfonyl)piperidine-4-carboxylate (2y)

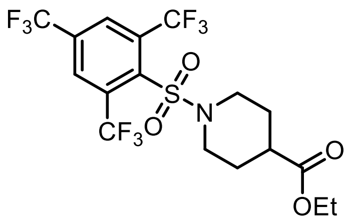

Following GP3 using ethyl isonipecotate (80.2 mg, 78.6 µL, 0.5 mmol), sulfonamide **2y** was obtained as a white solid (206 mg, 0.411 mmol, 82%).

^{1}H NMR (400 MHz, CDCl$_3$): δ 8.25 (s, 2H), 4.21 – 4.01 (m, 2H), 3.73 – 3.56 (m, 2H), 3.10 – 2.96 (m, 2H), 2.61 – 2.48 (m, 1H), 2.05 – 1.78 (m, 4H), 1.25 (t, *J* = 7.1 Hz, 3H) ppm.

^{13}C NMR (101 MHz, CDCl$_3$): δ 173.7, 145.2, 134.2 (q, *J* = 35.2 Hz), 133.1 (q, *J* = 34.0 Hz), 129.6 – 129.1 (m), 126.3-118.1 (q, *J* = 277 Hz), 126.1-117.9 (q, *J* = 275 Hz), 60.9, 46.1, 40.3, 27.9, 14.3 ppm.

^{19}F NMR (377 MHz, CDCl$_3$): δ -55.5 (6F), -63.6 (3F) ppm.

IR (neat): ν_{max} 1714, 1269, 1185, 1154, 1136, 1112, 1086, 1028, 948, 914, 725, 683, 573 cm^{-1}.

HRMS (ESI⁺): exact mass calculated for $[M+H]^+$ ($C_{17}H_{17}F_9NO_4S^+$) requires *m/z* 502.0729, *m/z* found: 502.0734.

***N*-Methoxy-*N*-methyl-1-((2,4,6-tris(trifluoromethyl)phenyl)sulfonyl)piperidine-4-carboxamide (2z)**

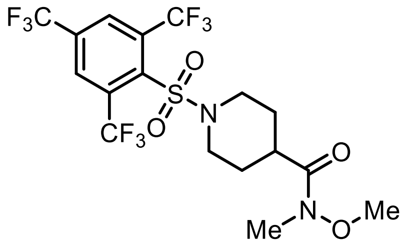

Following GP3 using *N*-methoxy-*N*-methylpiperidine-4-carboxamide (18.9 mg, 0.11 mmol), sulfonamide **2z** was obtained as a white solid (49 mg, 0.0949 mmol, 86%).

^{1}H NMR (400 MHz, CDCl$_3$): δ 8.26 (s, 2H), 3.80 – 3.72 (m, 2H), 3.70 (s, 3H), 3.18 (s, 3H), 3.10 – 2.99 (m, 2H), 2.95 – 2.83 (m, 1H), 1.96 – 1.77 (m, 4H) ppm.

^{13}C NMR (101 MHz, CDCl$_3$): δ 174.9, 145.3, 134.2 (q, J=35.1 Hz), 133.2 (q, J=34.0 Hz), 129.9 – 128.8 (m), 122.2 (q, J=276.7 Hz), 122.0 (q, J=273.6 Hz), 61.7, 46.0, 37.1, 32.4, 27.8 ppm.

^{19}**F NMR (377 MHz, CDCl$_3$):** δ -55.4 (6F), -63.6 (3F) ppm.

IR (neat): ν_{max} 1660, 1355, 1268, 1188, 1152, 1128, 996, 947, 933, 909, 720, 683 cm^{-1}.

HRMS (ESI$^+$): exact mass calculated for [M+H]$^+$ ($C_{17}H_{18}F_9N_2O_4S^+$) requires *m/z* 517.0838, *m/z* found: 517.0845.

4-Bromo-1-((2,4,6-tris(trifluoromethyl)phenyl)sulfonyl)piperidine (2aa)

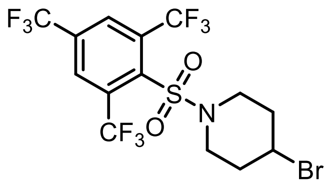

Following GP3 using 4-bromopiperidine hydrobromide (25.0 mg, 0.1 mmol), sulfonamide **2aa** was obtained as a white solid (47.0 mg, 93 µmol, 93%).

^{1}**H NMR (600 MHz, CDCl$_3$):** δ 8.27 (s, 2H), 4.50 (s, 1H), 3.60 – 3.52 (m, 2H), 3.42 – 3.32 (m, 2H), 2.30 – 2.20 (m, 2H), 2.15 – 2.02 (m, 2H) ppm.

^{13}**C NMR (151 MHz, CDCl$_3$):** δ 145.1, 134.3 (q, *J* = 35.1 Hz), 133.1 (q, *J* = 34.1 Hz), 129.4, 122.2 (q, *J* = 275.5 Hz), 48.2, 44.4, 35.1 ppm.

^{19}**F NMR (565 MHz, CDCl$_3$):** δ -55.5 (6F), -63.6 (3F) ppm.

IR (neat): ν_{max} 1350, 1273, 1194, 1174, 1149, 1133cm^{-1}.

HRMS (ESI$^+$): exact mass calculated for [M+H]$^+$ ($C_{14}H_{12}BrF_9NO_2S^+$) requires *m/z* 507.9623 found *m/z* 507.9623.

4-(4,4,5,5-Tetramethyl-1,3,2-dioxaborolan-2-yl)-1-((2,4,6 tris(trifluoromethyl)phenyl)sulfonyl)piperidine (2ab)

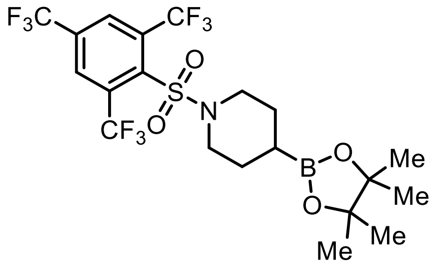

Following GP3 using piperidine-4-boronic acid pinacol ester hydrochloride (58.4 mg, 0.2 mmol), sulfonamide **2ab** was obtained as a white solid (77.6 mg, 0.14 mmol, 70%).

^{1}**H NMR (600 MHz, CDCl$_3$):** δ 8.24 (s, 2H), 3.54 – 3.48 (m, 2H), 3.07 – 3.00 (m, 2H), 1.78 – 1.68 (m, 5H), 1.22 (s, 12H) ppm.

^{13}**C NMR (151 MHz, CDCl$_3$):** δ 145.7, 134.0 (q, *J* = 34.9 Hz), 133.2 (q, *J* = 34.0 Hz), 129.3, 122.2 (q, *J* = 275.7 Hz), 122.1 (q, *J* = 273.4 Hz), 83.6, 47.9, 27.0, 24.9 ppm.

^{19}**F NMR (376 MHz, CDCl$_3$):** δ -55.5 (6F), -63.6 (3F) ppm.

^{11}**B NMR (193 MHz, CDCl$_3$):** δ 33.3 ppm.

IR (neat): ν_{max} 1357, 1286, 1268, 1192, 1158, 1134, 1112, 1081, 937, 725 cm^{-1}.

HRMS (ESI$^+$): exact mass calculated for $[M+H]^+$ ($C_{20}H_{24}BF_9NO_4S^+$) requires *m/z* 556.1370 found *m/z* 556.1386.

1-(4-Nitrophenyl)-4-((2,4,6-tris(trifluoromethyl)phenyl)sulfonyl)piperazine (2ac)

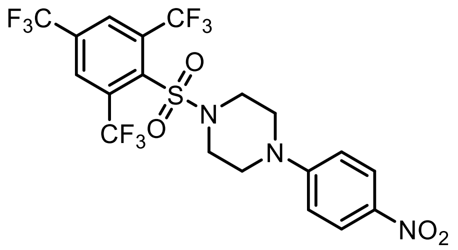

Following an adapted version of GP3 (addition of sulfonyl chloride at 20-25 °C) using 1-(4-nitrophenyl)piperazine (20.7 mg, 0.1 mmol), sulfonamide **2ac** was obtained as a yellow solid (53.4 mg, 97 µmol, 97%).

^{1}H NMR (400 MHz, CDCl$_3$): δ 8.30 (s, 2H), 8.14 (d, *J* = 9.3 Hz, 2H), 6.85 (d, *J* = 9.4 Hz, 2H), 3.62 – 3.40 (m, 8H) ppm.

^{13}C NMR (101 MHz, CDCl$_3$): δ 154.7, 144.6, 139.8, 134.6 (q, *J* = 35.2 Hz), 133.1 (q, *J* = 34.0 Hz), 129.6, 126.0, 122.2 (q, *J* = 276.7 Hz), 121.9 (q, *J* = 274.7 Hz), 113.9, 47.8, 46.3 ppm.

^{19}F NMR (377 MHz, CDCl$_3$): δ -55.4 (6F), -63.6 (3F) ppm.

IR (neat): ν_{max} 1602, 1482, 1355, 1327, 1274, 1191, 1139, 1114, 955, 915, 721, 696 cm^{-1}.

HRMS (ESI$^+$): exact mass calculated for $[M+H]^+$ ($C_{19}H_{15}F_9N_3O_4S^+$) requires m/z 552.0634, m/z found: 552.0634.

1-(4-(4,4,5,5-Tetramethyl-1,3,2-dioxaborolan-2-yl)pyridin-2-yl)-4-((2,4,6-tris(trifluoromethyl)phenyl)sulfonyl)piperazine (2ad)

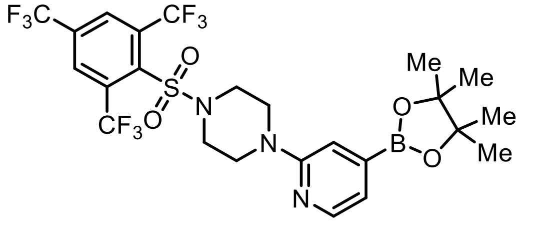

Following an adapted version of GP3 (addition of sulfonyl chloride at 20-25 °C) using 2-(piperazin-1-yl)pyridine-4-boronic acid, pinacol ester (28.9 mg, 0.1 mmol), sulfonamide **2ad** was obtained as a white solid (63.0 mg, 99 µmol, 99%) after an aqueous work-up. For further purification, the material could be subjected to column chromatography (heptane/EtOAc), but a substantial instability of the compound on silica was observed.

^{1}H NMR (400 MHz, CDCl$_3$): δ 8.28 (s, 2H), 8.21 (dd, *J* = 4.8, 0.7 Hz, 1H), 7.03 (s, 1H), 7.02 (d, *J* = 4.9 Hz, 1H), 3.72 – 3.67 (m, 4H), 3.49 – 3.45 (m, 4H), 1.34 (s, 12H) ppm.

^{13}C NMR (101 MHz, CDCl$_3$): δ 158.6, 147.6, 145.1, 134.4 (q, *J* = 35.2 Hz), 133.2 (q, *J* = 34.0 Hz), 129.5, 122.2 (q, *J* = 275.6 Hz), 122.0 (q, *J* = 273.7 Hz), 119.2, 112.9, 84.5, 46.7, 45.7, 25.0 ppm. Note: one carbon is missing.

^{19}F NMR (377 MHz, CDCl$_3$): δ -55.3 (6F), -63.6 (3F) ppm.

IR (neat): ν_{max} 1357, 1268, 1193, 1142, 1111, 1085, 954 cm^{-1}.

HRMS (ESI$^+$): exact mass calculated for [M+H]$^+$ ($C_{24}H_{26}BF_9N_3O_4S^+$) requires m/z 634.1588, found m/z 634.1569.

N-(3,4-Dimethoxyphenethyl)-N-methyl-2,4,6-tris(trifluoromethyl)benzenesulfonamide (2ae)

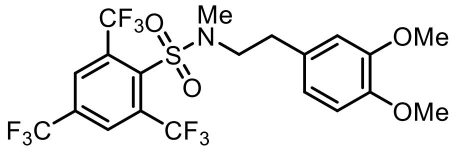

Following GP3 using 2-(3,4-dimethoxyphenyl)-*N*-methylethanamine (**1ae**, 19.4 µL, 20.6 mg, 0.1 mmol), sulfonamide **2ae** was obtained as a white solid (45.3 mg, 0.084 mmol, 84%).

^{1}H NMR (400 MHz, CDCl$_3$): δ 8.26 (s, 2H), 6.79 (d, *J* = 8.2 Hz, 1H), 6.76-6.72 (m, 2H), 3.87 (s, 3H), 3.85 (s, 3H), 3.47-3.40 (m, 2H), 2.97-2.91 (m, 2H), 2.83 (s, 3H) ppm.

^{13}C NMR (176 MHz, CDCl$_3$): δ 149.2, 148.0, 144.9, 134.2 (q, *J* = 35.2 Hz), 133.2 (q, *J* = 33.9 Hz), 130.5, 129.4 (m), 122.2 (q, *J* = 275.5 Hz), 122.0 (q, *J* = 273.5 Hz), 120.9, 112.2, 111.5, 56.1, 56.1, 53.1, 35.2, 34.3 ppm.

^{19}F NMR (565 MHz, CDCl$_3$): δ -55.7 (6F), -63.6 (3F) ppm.

IR (neat): ν_{max} 3080, 2837, 1590, 1348, 1238, 1189, 1159, 1136, 839, 462 cm^{-1}.

HRMS (ESI$^+$): exact mass calculated for [M+Na]$^+$ ($C_{20}H_{18}F_9NO_4SNa^+$) requires *m/z* 562.0705, found *m/z* 562.0704.

N-(3-(10,11-Dihydro-5H-dibenzo[b,f]azepin-5-yl)propyl)-N-methyl-2,4,6-tris(trifluoromethyl)benzenesulfonamide (2af)

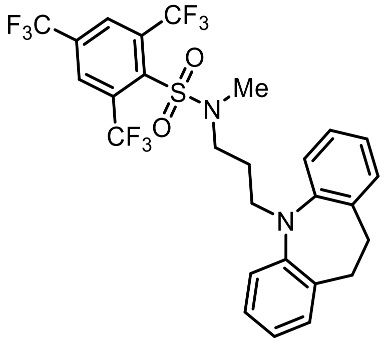

Following an adapted version of GP3 (addition of sulfonyl chloride at 20-25 °C) using desipramine hydrochloride (**1af·HCl**, 30.9 mg, 0.1 mmol), sulfonamide **2af** was obtained as a white solid (56.4 mg, 95 µmol, 95%).

^{1}H NMR (600 MHz, CDCl$_3$): δ 8.24 (s, 2H), 7.16 – 7.12 (m, 2H), 7.11 – 7.05 (m, 4H), 6.95 – 6.91 (m, 2H), 3.79 (t, *J* = 6.5 Hz, 2H), 3.29 – 3.25 (m, 2H), 3.12 (s, 4H), 2.62 (s, 3H), 1.97 – 1.91 (m, 2H) ppm.

^{13}C NMR (151 MHz, CDCl$_3$): δ 147.8, 145.0, 134.3, 134.1 (q, *J* = 35.2 Hz) 133.2 (q, *J* = 34.0 Hz), 130.1, 129.3, 126.7, 123.0, 122.14 (q, *J* = 275.7 Hz), 122.01 (d, *J* = 271.2 Hz), 119.9, 49.3, 47.8, 34.5, 32.2, 25.5 ppm.

^{19}F NMR (376 MHz, CDCl$_3$): δ -55.7 (6F), -63.5 (3F) ppm.

IR (neat): ν_{max} 1356, 1278, 1268, 1197, 1180, 1135, 1109, 1084, 1060, 990, 912, 777 cm^{-1}.

HRMS (ESI$^+$): exact mass calculated for [M+H]$^+$ ($C_{27}H_{24}F_9N_2O_2S^+$) requires m/z 611.1409, found m/z 611.1397.

(*S*)-*N*-Methyl-*N*-(3-(naphthalen-1-yloxy)-3-(thiophen-2-yl)propyl)-2,4,6 tris(trifluoromethyl)benzenesulfonamide (2ag)

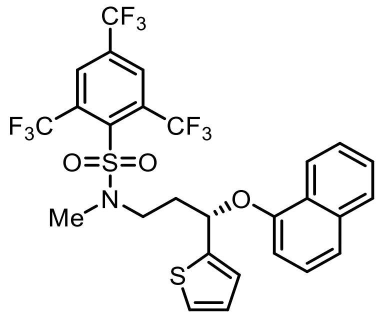

Following an adapted version of GP3 (addition of sulfonyl chloride at 20-25 °C) using duloxetine hydrochloride (**1ag**, 33.4 mg, 0.1 mmol), sulfonamide **2ag** was obtained as a white solid (59.0 mg, 92 µmol, 92%).

^{1}H NMR (400 MHz, CDCl$_3$): δ 8.29 – 8.25 (m, 1H), 8.21 (s, 2H), 7.79 – 7.75 (m, 1H), 7.52 – 7.46 (m, 2H), 7.39 (d, *J* = 8.3 Hz, 1H), 7.26 (d, *J* = 8.0 Hz, 1H), 7.23 – 7.20 (m, 1H), 7.09 – 7.06 (m, 1H), 6.93 (dd, *J* = 5.0, 3.5 Hz, 1H), 6.80 (d, *J* = 7.6 Hz, 1H), 5.68 (dd, *J* = 8.4, 4.3 Hz, 1H), 3.61 – 3.47 (m, 2H), 2.89 (s, 3H), 2.71 – 2.60 (m, 1H), 2.52 – 2.42 (m, 1H) ppm.

^{13}C NMR (176 MHz, CDCl$_3$): δ 152.9, 144.6, 144.1, 134.7, 134.2 (q, *J* = 35.3 Hz), 133.2 (q, *J* = 34.1 Hz), 129.4, 127.7, 126.9, 126.6, 126.0, 125.7, 125.5, 125.2, 125.1, 122.1 (q, J = 275.6 Hz), 121.9 (q, *J* = 273.4 Hz), 121.9, 121.1, 107.0, 73.9, 47.9, 36.9, 35.0 ppm.

^{19}F NMR (659 MHz, CDCl$_3$): δ -55.6 (6F), -63.6 (3F) ppm.

IR (neat): ν_{max} 1269, 1187, 1156, 1137, 1117, 1095, 703 cm^{-1}.

HRMS (ESI$^+$): exact mass calculated for [M+Na]$^+$ ($C_{27}H_{20}F_9NO_3S_2Na^+$) requires m/z 664.0633, found m/z 664.0612.

tert-Butyl ((2,4,6-(tristrifluoromethyl)phenyl)sulfonyl]-L-prolinate (2ah)

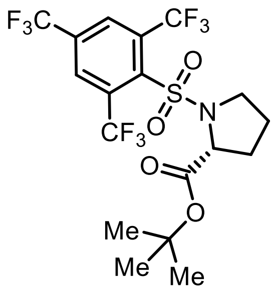

Following GP3 from *L*-proline *tert*-butyl ester hydrochloride (20.8 mg, 0.1 mmol), sulfonamide **2ah** was obtained as a yellow solid (35.2 mg, 0.068 mmol, 68%).

^{1}H NMR (600 MHz, CDCl$_3$): δ 8.29 (s, 2H), 4.58 (dd, *J* = 8.7, 3.6 Hz, 1H), 3.78 – 3.69 (m, 1H), 3.65 – 3.56 (m, 1H), 2.40 – 2.30 (m, 1H), 2.08 – 2.01 (m, 1H), 1.98 – 1.93 (m, 2H), 1.34 (s, 9H) ppm.

^{13}C NMR (151 MHz, CDCl$_3$): δ 170.6, 145.8, 134.0 (q, *J* = 35.1 Hz), 133.1 (q, *J* = 33.6 Hz), 129.4 (m), 122.2 (q, *J* = 274.5 Hz), 122.1 (q, *J* = 273.4 Hz), 82.2, 62.8, 50.1, 31.2, 27.8, 24.8 ppm.

^{19}F NMR (565 MHz, CDCl$_3$): δ -54.8 (6F), -63.6 (3F) ppm.

IR (neat): ν_{max} 2984, 2917, 2850, 1740, 1369, 1151, 1081, 910, 730, 465 cm^{-1}.

HRMS (ESI$^+$): exact mass calculated for [M+Na]$^+$ ($C_{18}H_{18}F_9NO_4SNa^+$) requires *m/z* 538.0705, found *m/z* 538.0704.

8-((2,4,6-Tris(trifluoromethyl)phenyl)sulfonyl)-8-azabicyclo[3.2.1]octan-3-one (2ai)

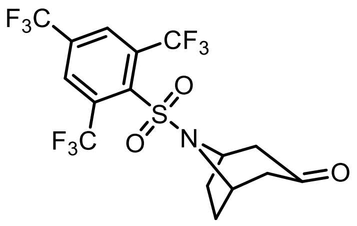

Following GP3 using nortropinone hydrochloride (16.7 mg, 0.1 mmol), sulfonamide **2ai** was obtained as a white solid (40.0 mg, 85 µmol, 85%).

^{1}H NMR (400 MHz, CDCl$_3$): δ 8.34 (s, 1H), 4.65 (s, 1H), 2.88 (dd, *J* = 16.8, 4.3 Hz, 1H), 2.41 (d, *J* = 16.1 Hz, 1H), 2.29 – 2.20 (m, 1H), 1.84 – 1.76 (m, 1H) ppm.

^{13}C NMR (101 MHz, CDCl$_3$): δ 206.3, 145.3, 134.7 (q, *J* = 35.3 Hz), 133.3 (q, *J* = 33.7 Hz), 129.8, 122.2 (q, *J* = 275.7 Hz), 121.9 (q, *J* = 273.7 Hz), 58.2, 49.2, 30.1 ppm.

^{19}F NMR (376 MHz, CDCl$_3$): δ -54.51 (6F), -63.64 (3F) ppm.

IR (neat) ν_{max}: 1717, 1356, 1193, 1149, 1116, 940, 914, 712.

HRMS (ESI$^+$): exact mass calculated for [M+H]$^+$ ($C_{16}H_{12}F_9NO_3SNa^+$) requires m/z 490.0292, found m/z 490.0279 .

2-Chloro-11-(4-((2,4,6-tris(trifluoromethyl)phenyl)sulfonyl)piperazin-1-yl)dibenzo[b,f][1,4]oxazepane (2aj)

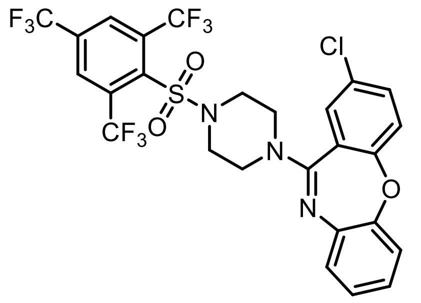

Following an adapted version of GP3 (addition of sulfonyl chloride at 20-25 °C) using amoxapine (**1aj**, 54.0 mg, 172 µmol), sulfonamide **2aj** was obtained as a white solid (102.0 mg, 155 µmol, 91%).

^{1}H NMR (400 MHz, CDCl$_3$): δ 8.30 (s, 2H), 7.41 (dd, *J* = 8.6, 2.6 Hz, 1H), 7.29 (d, *J* = 2.6 Hz, 1H), 7.20 (d, *J* = 8.6 Hz, 1H), 7.15 – 7.07 (m, 3H), 7.05 – 7.00 (m, 1H), 3.73 – 3.42 (m, 8H).

^{13}C NMR (101 MHz, CDCl$_3$): δ 159.5, 158.4, 151.8, 144.8, 139.8, 134.4 (q, J = 35.2 Hz), 133.2 (q, J = 33.9 Hz) 133.1, 130.6, 129.5, 128.9, 127.2, 126.0, 125.2, 124.7, 123.0, 122.2 (q, J = 275.7 Hz), 122.0 (q, J = 273.6 Hz), 120.3, 47.6, 46.5 ppm.

^{19}F NMR (377 MHz, CDCl$_3$): δ -55.2 (6F), -63.6 (3F) ppm.

IR (neat): ν_{max} 1611, 1364, 1289, 1271, 1207, 1192, 1155, 1106, 1082, 946, 917, 728 cm^{-1}.

HRMS (ESI$^+$): exact mass calculated for [M+H]$^+$ ($C_{26}H_{18}ClF_9N_3O_3S^+$) requires m/z 658.0608, found m/z 658.0604.

(3*S*,4*R*)-3-((Benzo[d][1,3]dioxol-5-yloxy)methyl)-4-(4-fluorophenyl)-1-((2,4,6-tris(trifluoromethyl)phenyl)sulfonyl)piperidine (2ak)

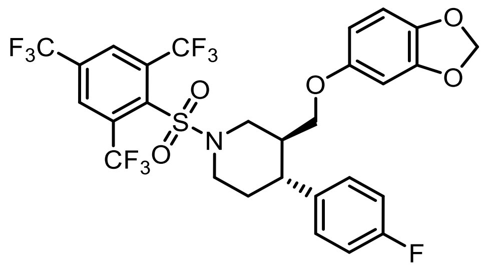

Following an adapted version of GP3 (addition of sulfonyl chloride at 20-25 °C) using paroxetine hydrochloride hemihydrate (**1ak·HCl**, 32.9 mg, 88 µmol), sulfonamide **2ak** was obtained as a white solid (57.8 mg, 86 µmol, 98%).

^{1}H NMR (700 MHz, CDCl$_3$): δ 8.29 (s, 2H), 7.17 – 7.14 (m, 2H), 6.98 (t, *J* = 8.6 Hz, 2H), 6.61 (d, *J* = 8.5 Hz, 1H), 6.33 (d, *J* = 2.5 Hz, 1H), 6.12 (dd, *J* = 8.5, 2.5 Hz, 1H), 5.89 – 5.87 (m, 2H), 3.97 – 3.90 (m, 2H), 3.59 (dd, *J* = 9.6, 2.6 Hz, 1H), 3.45 (dd, *J* = 9.6, 5.5 Hz, 1H), 3.11 – 3.03 (m, 2H), 2.85 (td, *J* = 12.1, 3.9 Hz, 1H), 2.32 – 2.23 (m, 1H), 2.07 – 1.97 (m, 1H), 1.89 – 1.85 (m, 1H).

^{13}C NMR (151 MHz, CDCl$_3$): δ 161.9 (d, *J* = 245.3 Hz), 154.1, 148.4, 145.3, 142.0, 138.26 (d, *J* = 3.1 Hz), 134.3 (q, *J* = 35.1 Hz), 133.2 (q, *J* = 34.0 Hz), 129.5, 129.0 (d, *J* = 7.6 Hz), 122.3 (q, *J* =

275.6 Hz), 122.0 (q, *J* = 275.8 Hz), 115.8 (d, *J* = 21.2 Hz), 108.0, 105.8, 101.3, 98.1, 68.3, 49.9, 47.5, 43.3, 42.0, 33.9 ppm.

^{19}F NMR (659 MHz, $CDCl_3$): δ -55.5 (6F), -63.5 (3F), -115.6 (1F) ppm

IR (neat): ν_{max} 1515, 1270, 1197, 1177, 1156, 1138, 1109, 1067, 1027, 911, 727, 711, 684 cm^{-1}.

HRMS (ESI$^+$): exact mass calculated for $[M+H]^+$ ($C_{28}H_{22}F_{10}NO_5S^+$) requires m/z 674.1054, found m/z 674.1051.

8-Chloro-11-(1-((2,4,6-tris(trifluoromethyl)phenyl)sulfonyl)piperidin-4-ylidene)-6,11-dihydro-5H-benzo[5,6]cyclohepta[1,2-b]pyridine (2al)

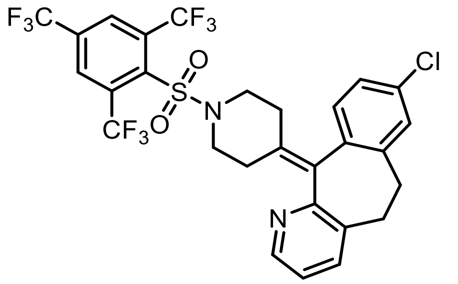

Following an adapted version of GP3 (addition of sulfonyl chloride at 20-25 °C) using desloratadine (**1al**, 31.1 mg, 0.1 mmol), sulfonamide **2al** was obtained as a white solid (65.0 mg, 99 µmol, 99%).

^{1}H NMR (400 MHz, $CDCl_3$): δ 8.38 (dd, *J* = 4.7, 1.5 Hz, 1H), 8.25 (s, 2H), 7.43 (dd, *J* = 7.7, 1.5 Hz, 1H), 7.18 – 7.16 (m, 1H), 7.14 – 7.07 (m, 3H), 3.59 – 3.51 (m, 1H), 3.49 – 3.41 (m, 1H), 3.39 – 3.30 (m, 2H), 3.29 – 3.20 (m, 2H), 2.91 – 2.76 (m, 2H), 2.72 – 2.63 (m, 1H), 2.59 – 2.51 (m, 1H), 2.45 – 2.37 (m, 2H) ppm.

^{13}C NMR (101 MHz, $CDCl_3$): δ 156.7, 146.9, 145.3, 139.7, 137.8, 137.4, 135.7, 135.5, 134.2 (q, *J* = 35.1 Hz), 133.5, 133.3, 133.2 (d, *J* = 34.0 Hz), 130.5, 129.3 ,129.2, 126.4, 122.6, 122.2 (q, *J* = 275.7 Hz), 122.0 (q, *J* = 273.5 Hz), 48.1, 48.0, 31.8, 31.6, 31.1, 30.9 ppm.

^{19}F NMR (376 MHz, $CDCl_3$): δ -55.4 (6F), -63.6 (3F) ppm.

IR (neat): ν_{max} 1364, 1270, 1195, 1150, 1135, 1145, 1083, 941, 909, 736, 718, 684, 572 cm^{-1}.

HRMS (ESI$^+$): exact mass calculated for $[M+H]^+$ ($C_{28}H_{21}ClF_9N_2O_2S^+$) requires m/z 655.0863, found m/z 655.0880.

2.5.4 Scale-up of protection

Protection of amine **1al** was also performed at a scale of 4.0 mmol (16 h reaction time) following an adapted version of GP3 (addition of sulfonyl chloride at 20-25 °C). The crude material obtained after evaporation of the reaction solvent was passed through a simple and short silica gel pad (see below) using EtOAc. After concentration, the desired amine was obtained in excellent yield (99%, 2.61 g, 3.98 mmol).

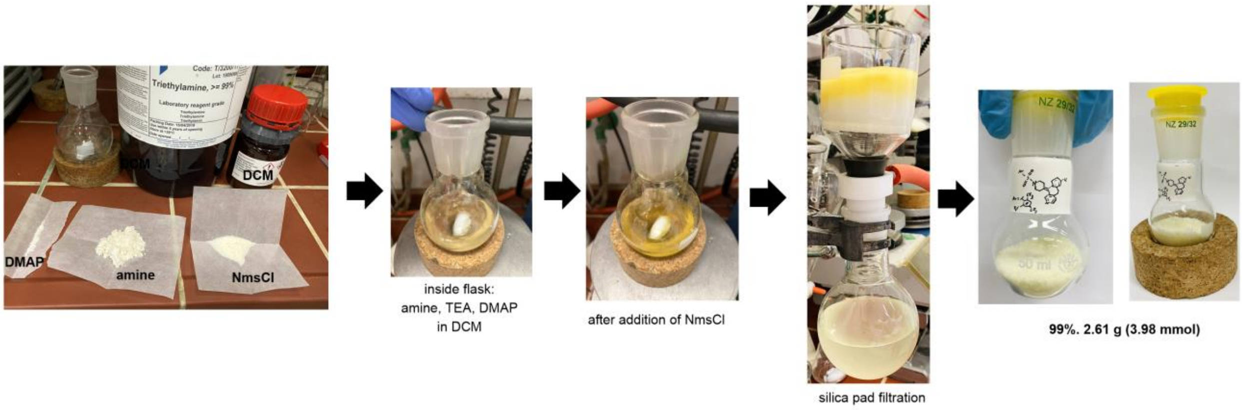

2.6. Deprotection of amines (1a-1al)

2.6.1. Primary amines (1a-1x)

Cyclohexylamine hydrochloride (1a·HCl) - CAS number: 4998-76-9

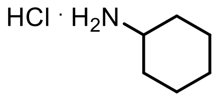

Following GP5 (reaction time: 5 h) using sulfonamide **2a** (88.7 mg, 0.2 mmol), compound **1a·HCl** was obtained as a white solid (27.0 mg, 0.199 mmol, 99%) after converting the free amine **1a** to the corresponding hydrochloride salt (PP1).

^{1}H NMR (400 MHz, MeOD): δ 3.11 – 3.01 (m, 1H), 2.01 – 1.95 (m, 2H), 1.85 – 1.78 (m, 2H), 1.74 – 1.69 (m, 2H), 1.49 – 1.18 (m, 5H) ppm.

^{13}C NMR (151 MHz, MeOD): δ 51.6, 31.9, 25.9, 25.3 ppm.

((1*S*,2*R*,5*S*)-6,6-Dimethylbicyclo[3.1.1]heptan-2-yl)methanamine (1d) - CAS number: 73522-42-6

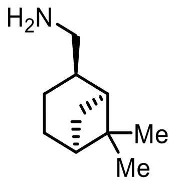

Following GP5 (reaction time: 5 h) using sulfonamide **2d** (49.7 mg, 0.10 mmol), amine **1d** was observed in a 99% NMR yield (CH_2Br_2 as internal standard).

2-(3,4-Dimethoxyphenyl)ethan-1-amine (1e) - CAS number: 120-20-7

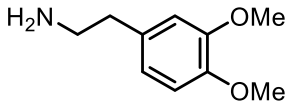

Following GP5 (reaction time: 5 h) using sulfonamide **2e** (52.5 mg, 0.1 mmol), amine **1e** was obtained as a yellow oil (16.7 mg, 0.0921 mmol, 92%) after acid-base extraction (PP2).

^{1}H NMR (400MHz, $CDCl_3$): δ 6.80 (d, *J* = 7.9 Hz, 1H), 6.77 – 6.71 (m, 2H), 3.87 (s, 3H), 3.85 (s, 3H), 2.94 (t, *J* = 6.4 Hz, 2H), 2.69 (t, *J* = 6.8 Hz, 2H), 1.31 (br s, 2 H) ppm.

^{13}C NMR (101 MHz, $CDCl_3$): δ 149.0, 147.6, 132.5, 120.8, 112.2, 111.4, 56.1, 56.0, 43.8, 39.7 ppm.

1-(3-Aminopropyl)-2-pipecoline (1f) - CAS number: 25560-00-3

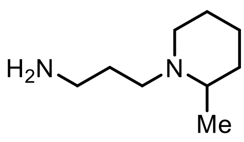

Following GP5 (reaction time: 5 h) using sulfonamide **2f** (75.1 mg, 0.15 mmol), amine **1f** was observed in a 90% NMR yield (mesitylene as internal standard).

***tert*-Butyl (3-aminopropyl)carbamate (1g) - CAS number: 75178-96-0**

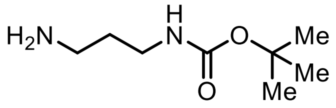

Following GP5 (reaction time: 5 h) using sulfonamide **2g** (36.8 mg, 0.07 mmol), amine **1g** was observed in a 93% NMR yield (mesitylene as internal standard).

Tryptamine hydrochloride (1h·HCl) - CAS number: 343-94-2

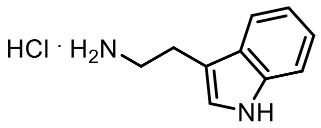

Following GP5 (reaction time: 5 h) using sulfonamide **2h** (75.7 mg, 0.15 mmol), compound **1h·HCl** was obtained as a white solid (29.0 mg, 0.147 mmol, 98%) after converting the free amine **1h** to the corresponding hydrochloride salt (PP1).

^{1}H NMR (400 MHz, MeOD): δ 7.57 (d, *J* = 7.9 Hz, 1H), 7.38 (d, *J* = 8.1 Hz, 1H), 7.18 (s, 1H), 7.13 (t, *J* = 7.5 Hz, 1H), 7.05 (t, *J* = 7.4 Hz, 1H), 4.22 (s, 2H), 3.24 (t, *J* = 7.2 Hz, 2H), 3.13 (t, *J* = 7.3 Hz, 2H) ppm. *One of the N-H was not observed in ^{1}H NMR.

^{13}C NMR (151 MHz, MeOD): δ 138.5, 128.2, 124.3, 122.7, 120.0, 118.9, 112.5, 110.2, 41.2, 24.5 ppm.

Pyridin-2-ylmethanamine (2i) - CAS number: **3731-51-9**

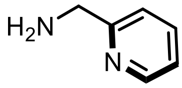

Following GP5 using sulfonamide **2i** (45.2 mg, 0.1 mmol), amine **1i** was observed in a 92% NMR yield (CH_2Br_2 as internal standard).

Furan-2-ylmethanamine (1j) - CAS number: 617-89-0

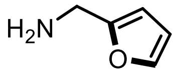

Following GP5 using sulfonamide **2j** (22.1 mg, 0.05 mmol), amine **1j** was observed in a 99% NMR yield (CH_2Br_2 as internal standard).

Thiophen-2-ylmethanamine (1k) - CAS number: 27757-85-3

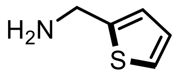

Following GP5 using sulfonamide **2k** (22.9 mg, 0.05 mmol), amine **1k** was observed in a 98% NMR yield (CH_2Br_2 as internal standard).

Benzylamine (1l) - CAS number: 100-46-9

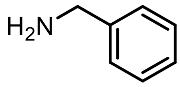

Following GP5 (reaction time: 5 h) using sulfonamide **2l** (13.5 mg, 0.03 mmol), amine **1l** was observed in a 97% NMR yield (CH_2Br_2 as internal standard).

4-(Aminomethyl)benzonitrile hydrochloride (1m·HCl) - CAS number: 15996-76-6

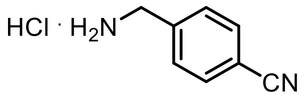

Following GP5 (reaction time: 5 h) using sulfonamide **2m** (89.2 mg, 0.187 mmol), compound **1m·HCl** was obtained as a white solid (31.0 mg, 0.184 mmol, 98%) after converting the free amine **1m** to the corresponding hydrochloride salt (PP1).

^{1}H NMR (400 MHz, MeOD): δ 7.82 (d, *J* = 8.3 Hz, 2H), 7.66 (d, *J* = 8.6 Hz, 2H), 4.22 (s, 2H) ppm.

^{13}C NMR (151 MHz, MeOD): δ 139.7, 134.0, 130.9, 119.1, 114.0, 43.7 ppm.

(4-Methoxyphenyl)methanamine hydrochloride (1n·HCl) - CAS number: 17061-61-9

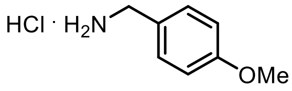

Following GP5 (reaction time: 5 h) using sulfonamide **1n** (79.4 mg, 0.165 mmol), compound **1n·HCl** was obtained as a white solid (28.5 mg, 0.164 mmol, 99%) after converting the free amine **1n** to the corresponding hydrochloride salt (PP1).

^{1}H NMR (600 MHz, MeOD): δ 7.40 (d, *J* = 8.6 Hz, 2H), 6.98 (d, *J* = 8.6 Hz, 2H), 4.05 (s, 2H), 3.81 (s, 3H) ppm.

^{13}C NMR (151 MHz, MeOD): δ 161.8, 131.6, 126.3, 115.5, 55.8, 43.9 ppm.

Butyl (*R*)-2-(4-(4-(aminomethyl)-2-fluorophenoxy)phenoxy)propanoate (1p)

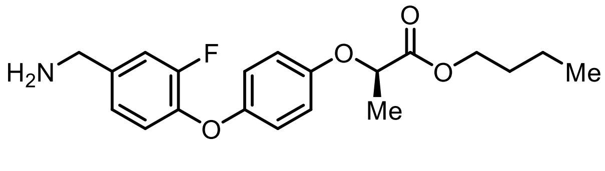

Following an adapted version of GP5 (reaction time: 20 h) using sulfonamide **2p** (51.0 mg, 0.072 mmol), amine **1p** was observed in a 93% NMR yield (mesitylene as internal standard).

***tert*-Butylamine (1q) - CAS number: 75-64-9**

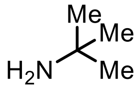

Following GP5 (THF-d8 as solvent, reaction time: 5 h) using sulfonamide **2q** (41.7 mg, 0.10 mmol), amine **1q** was observed in a 24% NMR yield (mesitylene as internal standard). The diarylsulfide side-product **3** was formed in 98% yield (boiling point of *tert*-butylamine: 45 °C/760 mmHg).

Mentylamine (1r)

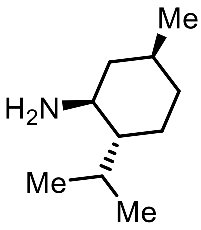

Following GP5 using sulfonamide **2r** (28.8 mg, 58 µmol), amine **1r** was obtained as a pale-yellow oil (6.7 mg, 0.043 mmol, 86%) after column chromatography (PP3).

^{1}H NMR (400 MHz, CDCl$_3$): δ 2.49 (ddd, *J* = 11.0, 9.8, 3.9 Hz, 1H), 2.10 (dtd, *J* = 13.9, 6.9, 2.6 Hz, 1H), 1.79 (dtd, *J* = 12.4, 3.7, 2.3 Hz, 1H), 1.67 (ddd, *J* = 12.2, 5.5, 3.1 Hz, 1H), 1.62 – 1.53 (m, 1H), 1.45 – 1.34 (m, 1H), 1.14 (s, 2H), 0.98 – 0.78 (m, 4H), 0.92 – 0.89 (m, 3H), 0.87 (d, *J* = 6.6 Hz, 3H), 0.76 (d, *J* = 7.0 Hz, 3H) ppm.

^{13}C NMR (101 MHz, CDCl$_3$): δ 52.2, 46.3, 40.3, 34.0, 31.8, 25.9, 23.0, 22.1, 21.2, 15.5 ppm.

The spectroscopic data matched the data reported in the literature for this compound.[3]

***p*-Methoxyaniline hydrochloride (1s·HCl) - CAS number: 20265-97-8**

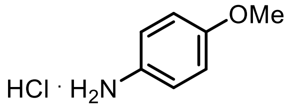

Following GP5 (reaction time: 5 h) using sulfonamide **2s** (46.7 mg, 0.10 mmol), compound **1s·HCl** was obtained as a white solid (15.0 mg, 0.094 mmol, 94%) after converting the free amine **1s** to the corresponding hydrochloride salt (PP1).

^{1}H NMR (400 MHz, MeOD): δ 7.34 (d, *J* = 8.9 Hz, 2H), 7.06 (d, *J* = 8.9 Hz, 2H), 3.83 (s, 3H) ppm.

^{13}C NMR (151 MHz, MeOD): δ 161.5, 125.2, 124.2, 116.1, 56.1 ppm.

3,4,5-Trimethoxyaniline (1t) - CAS number: 24313-88-0

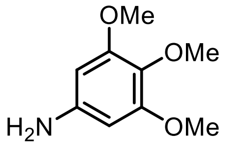

Following GP5 (reaction time: 15 h) using sulfonamide **2t** (26.4 mg, 0.05 mmol), amine **1t** was observed in 90% NMR yield (mesitylene as internal standard).

1*H*-Indol-5-amine (1u) - CAS number: 5192-03-0

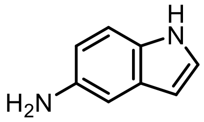

Following GP5 (reaction time: 15 h) using sulfonamide **2u** (23.8 mg, 0.05 mmol), amine **1u** was observed in 83% NMR yield (mesitylene as internal standard).

Methyl *L*-isoleucinate (1v) - CAS number: 2577-46-0

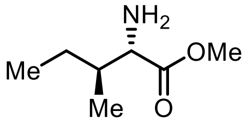

Following GP5 (reaction time: 5 h) using sulfonamide **2v** (24.5 mg, 0.05 mmol), amine **1v** was observed in a 92% NMR yield (mesitylene as internal standard).

Benzyl L-serinate (1w) - CAS number: 4726-96-9

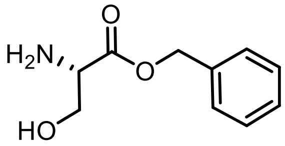

Following GP5 using sulfonamide **2w** (100 mg, 0.185 mmol), amine **1w** was observed in a 91% NMR yield (CH_2Br_2 as internal standard).

Methyl *L*-serinate hydrochloride (1x·HCl) - CAS number: 5680-80-8

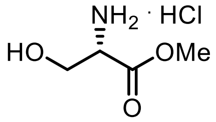

Following GP5 (reaction time: 5 h) using sulfonamide **2x** (23.2 mg, 0.05 mmol), compound **1x·HCl** was obtained as a white solid (7.2 mg, 0.046 mmol, 93%) after converting the free amine **1x** to the corresponding hydrochloride salt (PP1).

^{1}H NMR (400 MHz, MeOD): δ 4.13 (d, *J* = 4.0 Hz, 1H), 4.01 (dd, *J* = 11.8, 4.5 Hz, 2H), 3.13 (dd, *J* = 11.8, 3.5 Hz, 1H), 3.85 (s, 3H) ppm.

^{13}C NMR (151 MHz, MeOD): δ 169.4, 60.7, 56.1, 53.7 ppm.

3.6.2. Secondary amines (1y-1al)

Ethyl isonipecotate (1y) - CAS number: 1126-09-6

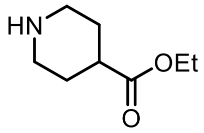

Following GP5 (reaction time: 15 h, 1.5 equiv of PhSH) using sulfonamide **2y** (23.8 mg, 0.05 mmol), amine **1y** was observed in 99% NMR yield (mesitylene as internal standard).

***N*-Methoxy-*N*-methylpiperidine-4-carboxamide (1z) – CAS 160809-37-0**

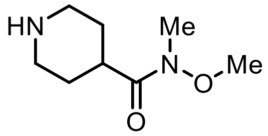

Following GP5 using sulfonamide **2z** (51.6 mg, 0.1 mmol), amine **1z** was isolated using column chromatography (PP3) as a yellow solid (17.2 mg, 0.1 mmol, 99%).

^{1}H NMR (600 MHz, MeOD): δ 3.76 (s, 3H), 3.20 (s, 3H), 3.16 – 3.07 (m, 2H), 2.96 (s, 1H), 2.71 (td, *J* = 12.6, 2.6 Hz, 2H), 1.81 – 1.59 (m, 4H) ppm.

^{13}C NMR (151 MHz, MeOD): δ 177.6, 62.2, 46.0, 39.2, 32.4, 29.3 ppm.

4-Bromopiperidine (1aa) - CAS number: 54288-70-9 (for HBr salt)

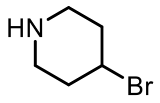

Following GP5 using sulfonamide **2aa** (25.4 mg, 0.05 mmol), amine **1aa** was observed in a 95% NMR yield (CH_2Br_2 as internal standard).

4-(4,4,5,5-Tetramethyl-1,3,2-dioxaborolan-2-yl)piperidine (1ab) - CAS number: 1087160-40-4 (for HBr salt)

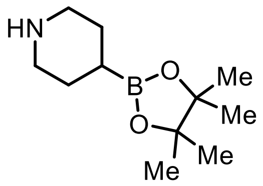

Following GP5 using sulfonamide **2ab** (55.5 mg, 0.1 mmol), amine **1ab** was observed in a 98% NMR yield (CH_2Br_2 as internal standard).

1-(4-Nitrophenyl)piperazine (1ad) - CAS number: 6269-89-2

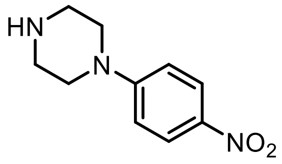

Following GP5 using sulfonamide **2ad** (55.1 mg, 0.1 mol), amine **1ad** was observed in a 99% NMR yield (CH_2Br_2 as internal standard).

3,4-Dimethoxy-*N*-methyl-benzeneethanamine (1ae) - CAS number: 3490-06-0

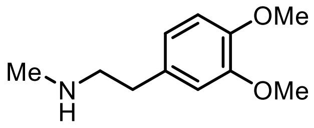

Following GP5 (reaction time: 5 h) using sulfonamide **2ae** (64.7 mg, 0.12 mmol), amine **1ae** was observed in a 99% NMR yield (mesitylene as internal standard).

Desipramine (1af) - CAS number: 50-47-5

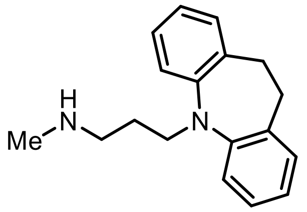

Following GP5 using sulfonamide **2af** (31.6 mg, 0.05 mmol), amine **1af** was observed in a 99% NMR yield (CH_2Br_2 as internal standard).

Duloxetine (1ag) - CAS number: 136434-34-9 (for HCl salt)

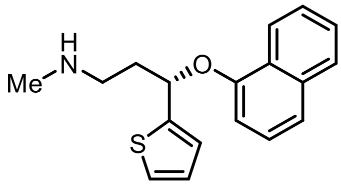

Following GP5 using sulfonamide **2ag** (64.2 mg, 0.1 mmol), amine **1ag** was observed in a 96% NMR yield (CH_2Br_2 as internal standard).

***L*-Proline *tert*-butyl ester (1ah) - CAS number: 2812-46-6**

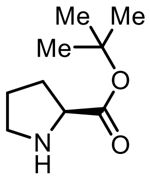

Following GP5 (reaction time: 5 h) using sulfonamide **2ah** (51.5 mg, 0.10 mmol), amine **1ah** was observed in a 99% NMR yield (mesitylene as internal standard).

Nortopinone (1ai) – CAS number: 25602-68-0 (for HCl salt)

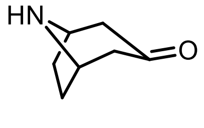

Following GP5 using sulfonamide **2ai** (18.5 mg, 0.04 mmol), amine **1ai** was observed in a 99% NMR yield (CH_2Br_2 as internal standard).

Amoxapine (1aj) - CAS number: 14028-44-5

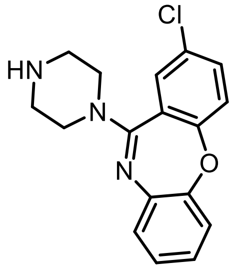

Following GP5 using sulfonamide **1aj** (32.7 mg, 0.05 mmol), amine **1aj** was observed in a 98% NMR yield (CH_2Br_2 as internal standard).

Paroxetine (1ak) - CAS number: 110429-35-1 (HCl salt)

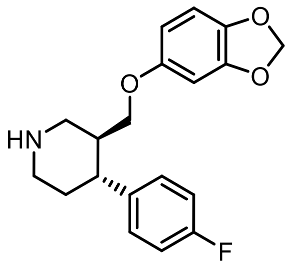

Following GP5 using sulfonamide **2ak** (35.7 mg, 0.053 mmol), amine **1ak** was obtained as a colorless oil (15.9 mg, 0.048 mmol, 91%) after flash column chromatography (PP3).

^{1}H NMR (700 MHz, CDCl$_3$): δ 7.18 (dd, *J* = 8.5, 5.4 Hz, 2H), 6.98 (t, *J* = 8.6 Hz, 2H), 6.62 (d, *J* = 8.5 Hz, 1H), 6.33 (d, *J* = 2.5 Hz, 1H), 6.12 (dd, *J* = 8.5, 2.5 Hz, 1H), 5.87 (d, *J* = 3.7 Hz, 2H), 5.64 – 5.25 (br, 1H), 3.59 – 3.54 (m, 2H), 3.47 – 3.39 (m, 2H), 2.93 – 2.86 (m, 2H), 2.74 (td, *J* = 11.9, 3.8 Hz, 1H), 2.37 – 2.31 (m, 1H), 2.07 – 2.00 (m, 1H), 1.94 – 1.89 (m, 1H) ppm.

^{13}C NMR (176 MHz, CDCl$_3$): δ 161.85 (d, *J* = 245.1 Hz), 154.2, 148.3, 141.9, 138.68 (d, *J* = 3.0 Hz), 129.00 (d, *J* = 7.5 Hz), 115.76 (d, *J* = 21.2 Hz), 108.0, 105.7, 101.3, 98.1, 68.7, 48.8, 46.0, 43.3, 41.4, 33.0 ppm.

^{19}F NMR (659 MHz, CDCl$_3$): δ -115.8 (1F) ppm.

HRMS (ESI$^+$): exact mass calculated for $[M+H]^+$ ($C_{19}H_{21}FNO_3^+$) requires m/z 330.1500, found m/z 330.1500.

Desloratadine (1al) - CAS number: 100643-71-8

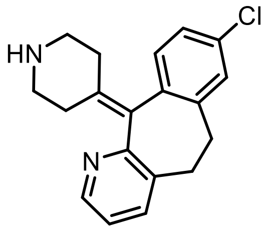

Following GP5 using sulfonamide **2al** (32.7 mg, 0.05 mmol), amine **1al** was observed in a 99% NMR yield (CH_2Br_2 as internal standard).

2.6.3. Diaryl sulfide 3

Phenyl(2,4,6-tris(trifluoromethyl)phenyl)sulfane (3)

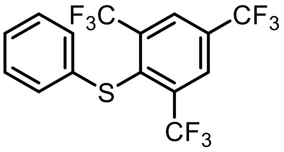

^{1}H NMR (700 MHz, $CDCl_3$): δ 8.27 (s, 2H), 7.24 – 7.21 (m, 2H), 7.16 (ddd, *J* = 7.4, 3.9, 1.1 Hz, 1H), 6.94 – 6.91 (m, 2H) ppm.

^{13}C NMR (176 MHz, $CDCl_3$): δ 139.14 (q, *J* = 30.8 Hz), 137.0, 135.9, 132.6 (q, *J* = 34.5 Hz), 129.2, 128.0, 127.2, 126.5, 122.56 (q, *J* = 273.2 Hz), 122.30 (q, *J* = 275.3 Hz) ppm.

^{19}F NMR (659 MHz, $CDCl_3$): δ -59.9 (6F), -63.3 (3F) ppm.

IR (neat): ν_{max} 1292, 1267, 1194, 1133, 1116, 1094, 916, 736, 684 cm^{-1}.

HRMS (ESI$^+$): exact mass calculated for $[M+H]^+$ ($C_{15}H_8F_9S^+$) requires m/z 391.0198, found m/z 391.0198.

3. Deprotection studies

3.1 Comparison Study of Nms- and Ns-amides derived from amide Umpolung

3.1.1 Preparation of compound 8

***N*-(1-Oxo-4-phenyl-1-(pyrrolidin-1-yl)butan-2-yl)-2,4,6-tris(trifluoromethyl)benzenesulfonamide (8)**

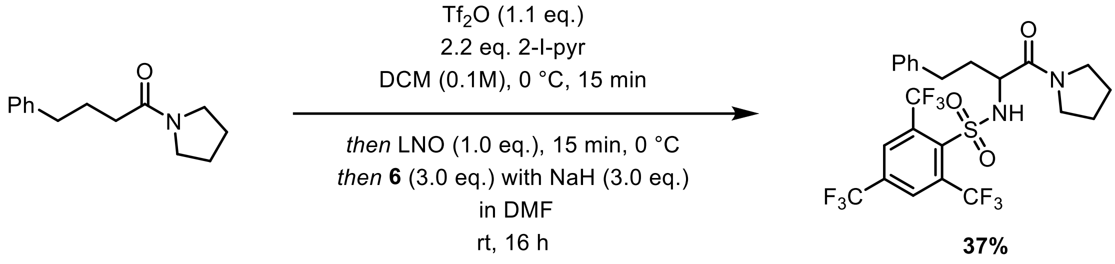

The preparation of this compound was carried out (0.2 mmol scale) adapting a procedure for the same transformation with $NsNH_2$ from the literature.[4] Compound **8** was obtained as a colorless oil (27.2 mg, 0.066 mmol, 37%).

^{1}H NMR (600 MHz, $CDCl_3$): δ 8.29 (s, 2H), 7.29 (t, *J* = 7.6 Hz, 2H), 7.22 – 7.19 (m, 3H), 6.04 (d, *J* = 9.1 Hz, 1H), 4.42 (td, *J* = 9.1, 3.5 Hz, 1H), 3.27 – 3.17 (m, 3H), 3.06 – 3.00 (m, 1H), 2.87 – 2.82 (m, 1H), 2.78 – 2.71 (m, 1H), 2.02 – 1.76 (m, 6H) ppm.

^{13}C NMR (151 MHz, $CDCl_3$): δ 168.7, 145.4, 140.4, 134.2 (q, *J* = 35.2 Hz), 132.7 (q, *J* = 33.2 Hz), 129.4, 128.7 (q, *J* = 1.8 Hz), 126.5, 122.3 (q, *J* = 275.2 Hz), 122.0 (q, *J* = 273.5 Hz), 55.5, 46.2, 45.9, 35.4, 31.6, 26.1, 24.0 ppm.

^{19}F NMR (565 MHz, $CDCl_3$): δ -54.75 (6F), -63.61 (3F) ppm.

IR (neat): ν_{max} 1633, 1275, 1191, 1133, 1115, 1080, 913, 703 cm^{-1}.

HRMS (ESI$^+$): exact mass calculated for $[M+H]^+$ ($C_{23}H_{22}F_9N_2O_3S^+$) requires m/z 577.1202, found m/z 577.1202.

3.1.2 Deprotection of compound 8

2-amino-4-phenyl-1-(pyrrolidin-1-yl)butan-1-one (9)

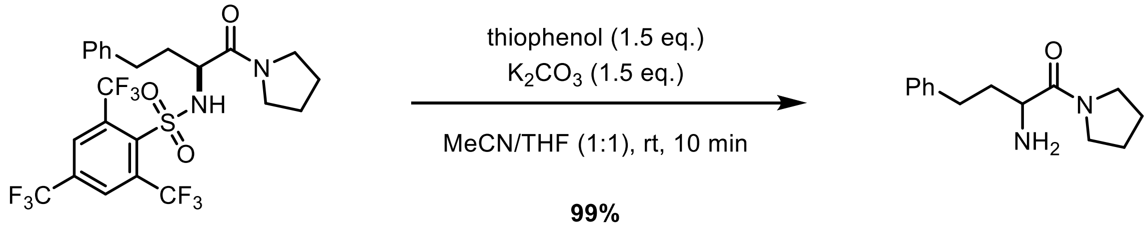

Following GP5 using sulfonamide **8** (18.3 mg, 31.7 µmol) and slightly higher amounts of thiophenol (1.5 equiv. instead of 1.0 equiv.). The title compound was obtained after column chromatography (DCM to DCM/MeOH (9:1) with 3% NH_3) as colorless oil (7.3 mg, 31 µmol, 99%) which agrees in all NMR data with a previous report.[4]

^{1}H NMR (400 MHz, $CDCl_3$): δ 7.30 – 7.26 (m, 2H), 7.22 – 7.16 (m, 3H), 3.54 – 3.38 (m, 3H), 3.30 (dt, *J* = 10.2, 6.6 Hz, 1H), 3.14 (dt, *J* = 10.2, 6.7 Hz, 1H), 2.85 – 2.66 (m, 2H), 1.93 – 1.75 (m, 8H) ppm.

^{13}C NMR (101 MHz, $CDCl_3$): δ 174.1, 141.7, 128.6 (2C), 126.1, 52.4, 46.1, 46.0, 36.9, 32.2, 26.2, 24.2 ppm.

3.2 Competition experiment (Ns, Cs, Nms)

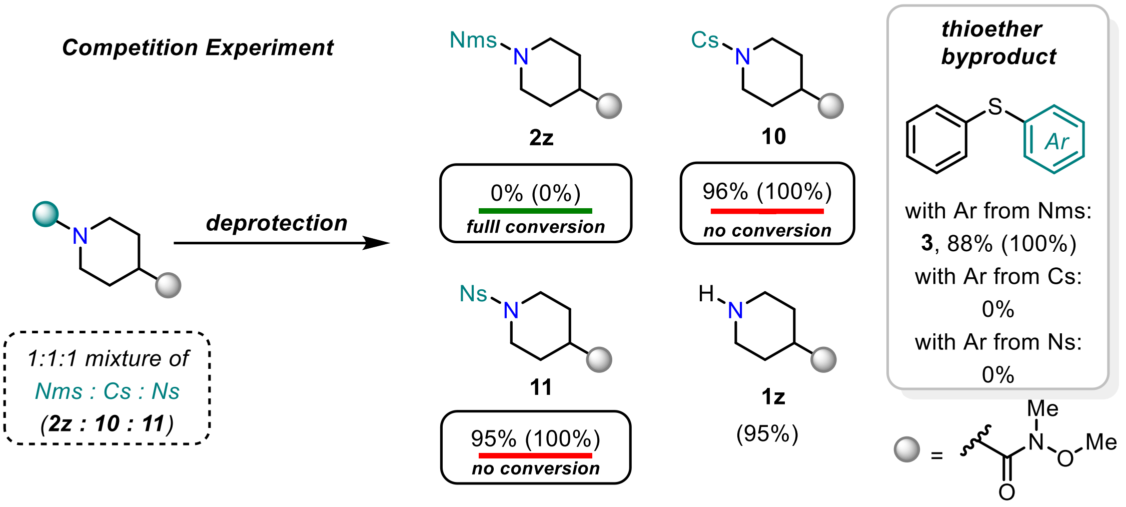

To a mixture of sulfonamide **2z** (51.6 mg, 0.1 mmol, 1.0 equiv.), **11** (35.7 mg, 0.1 mmol, 1.0 equiv.) and **10** (33.7 mg, 0.1 mmol, 1.0 equiv.) dissolved in THF (2 mL) was added K_2CO_3 (20.7 mg, 0.15 mmol, 1.5 equiv.), followed by dropwise addition of thiophenol (10.3 µL, 11 mg, 0.1 mmol, 1.0 equiv.). The resulting mixture was allowed to stir at room temperature for 16 h, before evaporating carefully the reaction solvent (25°C, 150 mbar, rotavapor). The crude material was analyzed by ^{1}H NMR (see NMR data below) with the addition of dibromomethane as an internal standard, demonstrating complete conversion of sulfonamide **2z** and completely unreacted sulfonamides for the corresponding Ns-protected **11** and Cs-protected amines **10**. Also, the deprotected piperidine was found in an 95% NMR yield. In addition, the previously isolated thioether cleavage product **3** of sulfonamide **2z** was found in a quantitative NMR yield. For a further verification, the crude material was subjected to column chromatography which allowed the almost complete recovery of the Ns-amide **11** (34.0 mg, 0.05 mmol, 95%) and Cs-amide **10** (32.5 mg, 0.096 mmol, 96%), together with the isolation of major amounts of thioether **3** (34.3 mg, 0.088mmol, 88%).

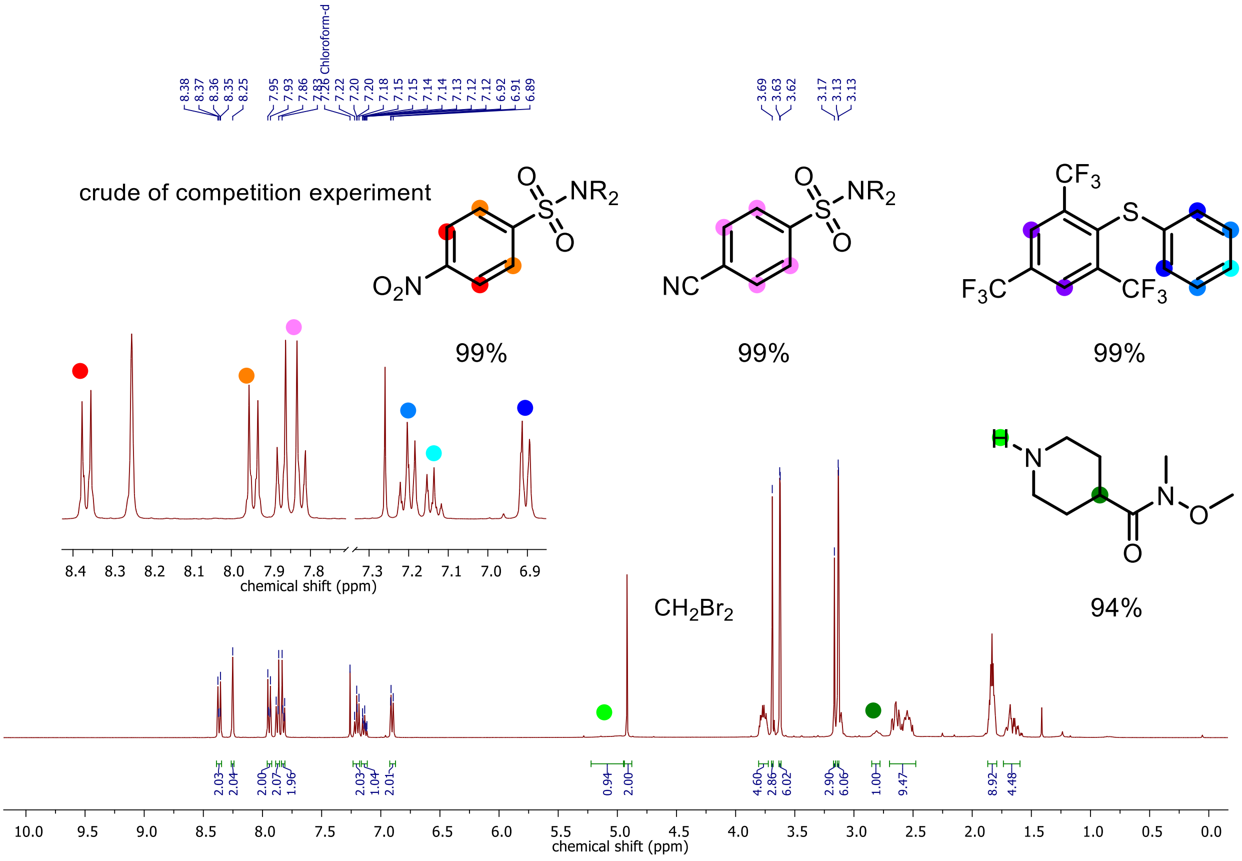
crude of competition experiment
99%
99%
99%
94%
CH_2Br_2
chemical shift (ppm)

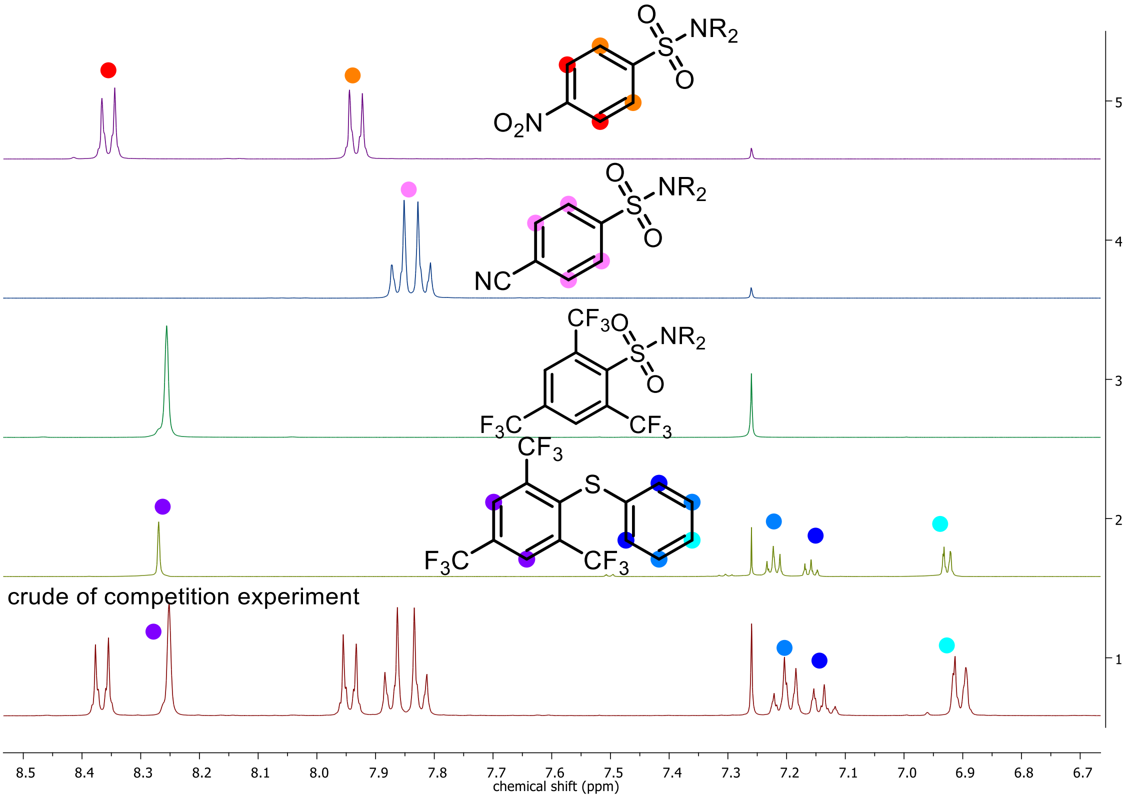
crude of competition experiment
chemical shift (ppm)

^{19}F (crude of competition experiment)

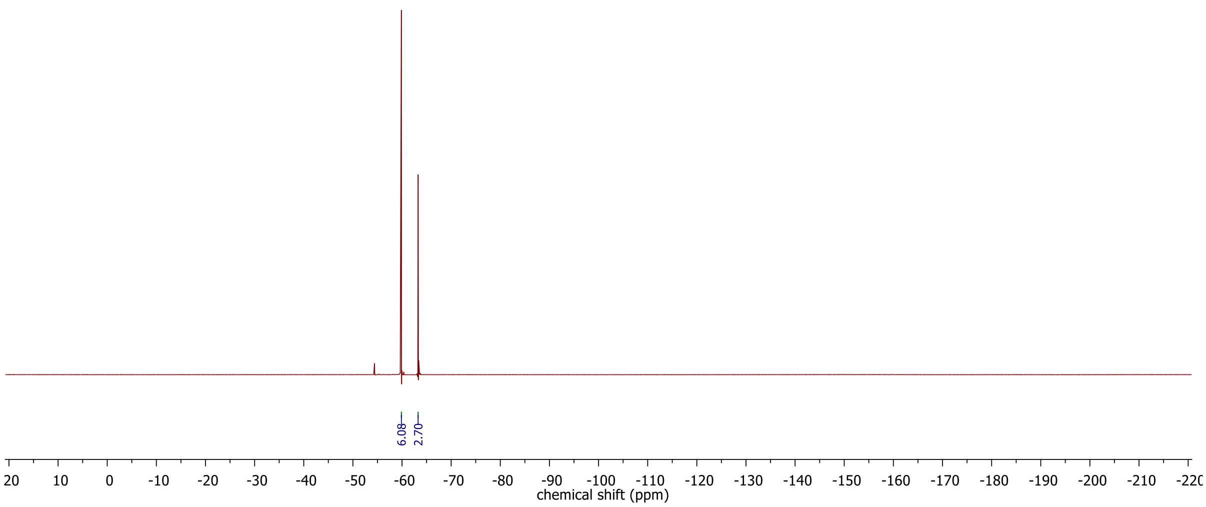

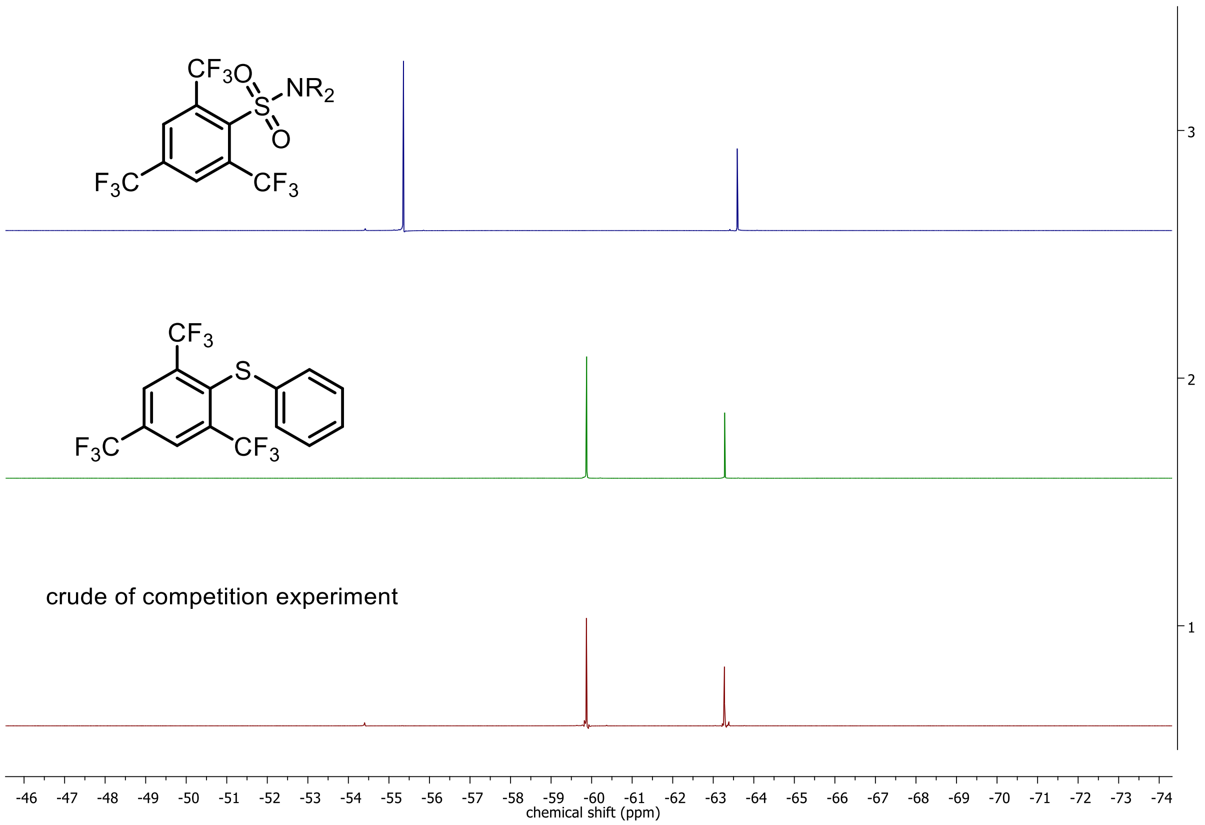

3.3 Deprotection followed by in-situ IR and NMR techniques

To gain further insight into the different deprotection reaction times of sulfonamides **2z** and **11**, we performed a kinetic study using an in-situ IR instrument (Mettler Toledo, ReactIR 702L). The sulfonamide band of compound **2z** was followed to determine the consumption/conversion rate. An IR band of the released amine was also detected (see figure below for an explanation of the exact IR band), but was much less intense and therefore less suitable for tracking the conversion rate. While sulfonamide **2z** reacted rapidly (99% conversion in 2 hours), sulfonamide **11** showed no conversion and was therefore heated at 40 °C for 4 days. The final result of this deprotection reaction was analyzed only by NMR and the addition of an internal NMR standard (15% conversion).

Analyzed Reaction:

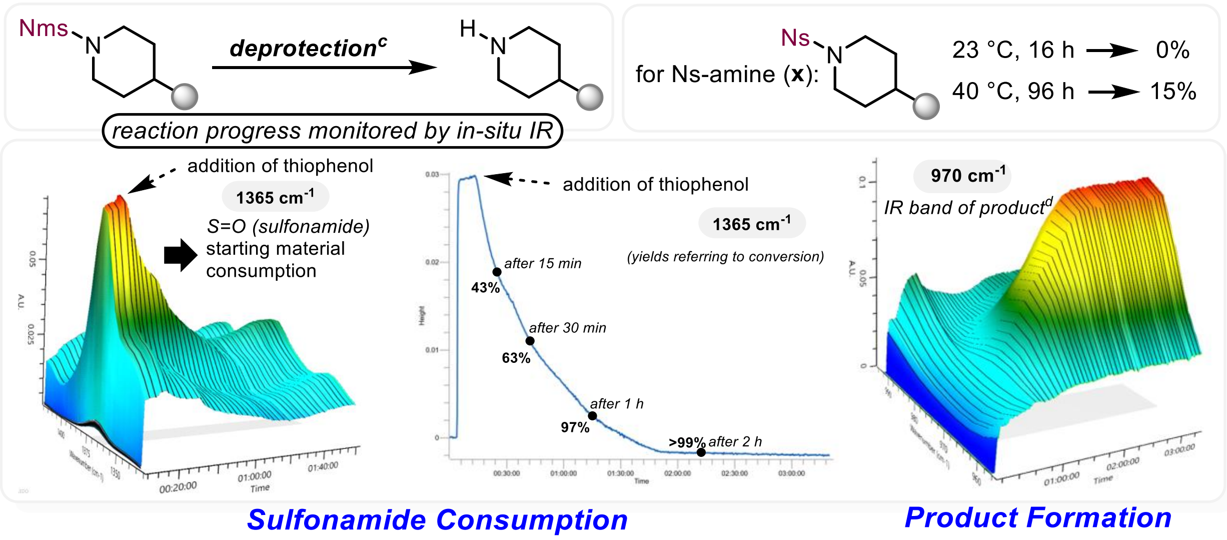

[c]PhSH (1.0 equiv.), K_2CO_3 (1.5 equiv.), THF, 23 °C, 16 h.

Set-up:

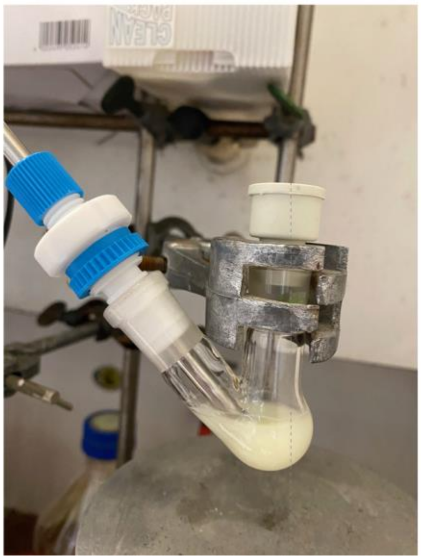

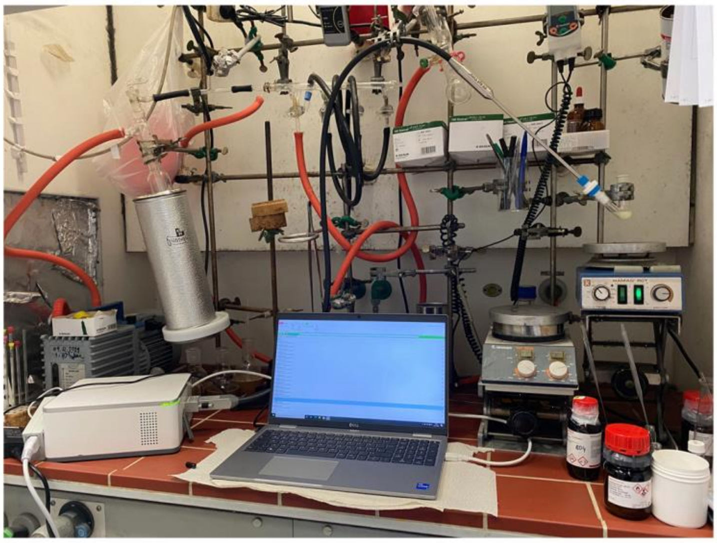

The *in-situ* IR experiments performed to study the kinetics of the deprotection were assisted by the calculation of the IR spectra of the protected amine, **2z**, and the deprotected product, **1z**.

The computed IR spectrum for the protected amine is shown in Figure S3. The highlighted vibrational mode at a computed 1358 cm^{-1} (matching an experimentally observed 1365 cm^{-1}) mainly involves the asymmetric stretching of the S–O bonds, with a smaller contribution of the S–N bond stretching. In reactional conditions, with the removal of the protecting group, this vibrational mode stops being visible, since the S–N bond is cleaved.

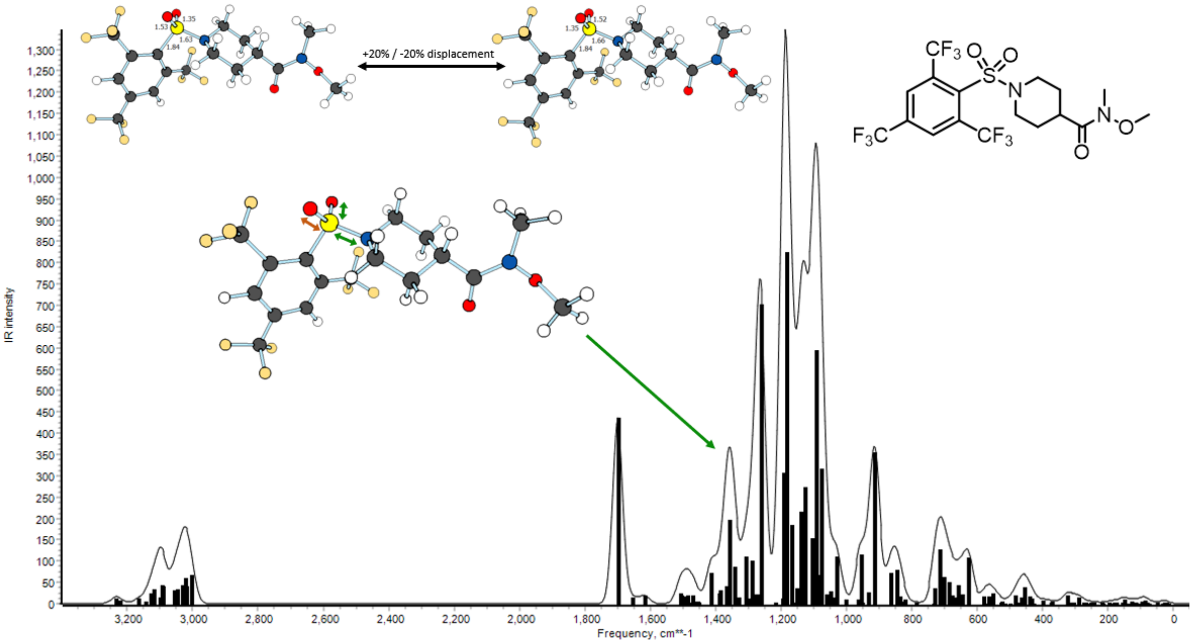

Figure S3. Computed IR spectra of the protected amine with a highlighted vibrational mode. Distances are shown in Å. Geometric parameters are shown for the structures obtained by perturbing the equilibrium geometry by +20% and -20% of the force constant of the selected vibrational mode. The coloured arrows represent the geometry displacement of the vibration: Asymmetric stretching of the two S–O bonds (one represented in orange and another in green) and symmetric stretching of one S–O the S–N bonds, represented in green.

The computed IR spectrum for the deprotected amine is shown in Figure S4. The highlighted vibrational mode at a computed 1033 cm^{-1} (matching an experimentally observed 985 cm^{-1}) was used to characterize the formation of this compound in the *in-situ* experiment. This vibrational mode mainly involves the N–O bond stretching (0.12 Å at a 20% geometry displacement) and smaller contributions of C–N, C–C and C–C_{CO} bond stretches and the C–N–C angle bending. The deprotection of **2z** (protected amine) to form a secondary amine enables the bending of this angle, and therefore the selected vibrational mode is a viable option to track the formation of the product in the *in-situ* IR studies.

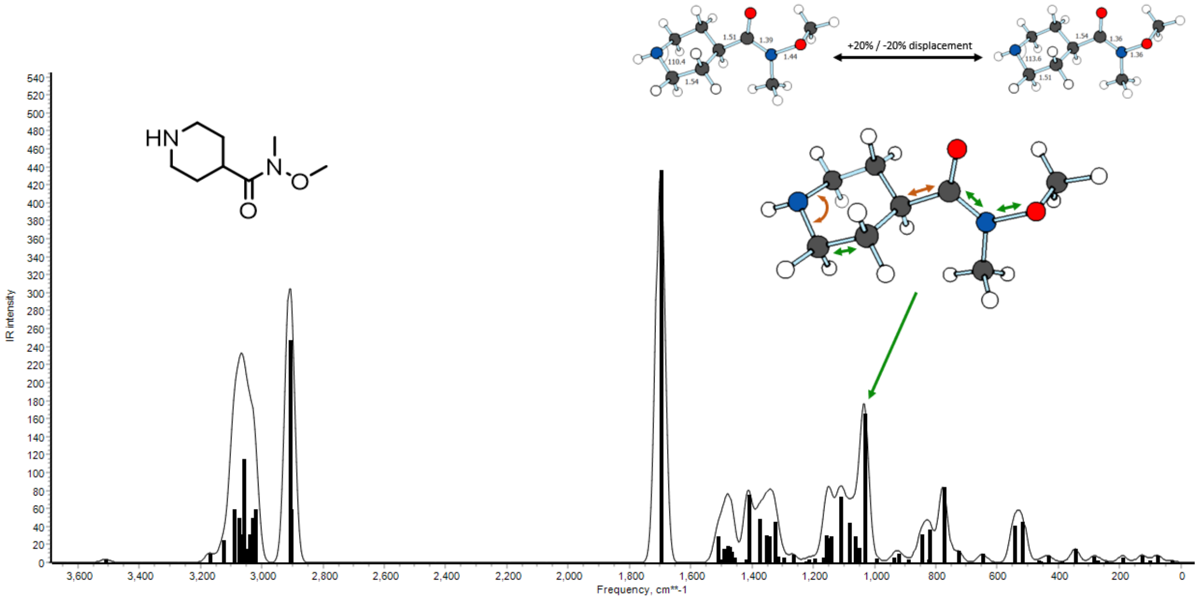

Figure S4. Computed IR spectra of the deprotected amine with a highlighted vibrational mode. Distances are shown in Å and the angle in °. Geometric parameters are shown for the structures obtained by perturbing the equilibrium geometry by +20% and -20% of the force constant of the selected vibrational mode. The coloured arrows represent the geometry displacement of the vibration: As the C–C, C–N and N–O bonds, represented in green, increase in length, the C–C_{CO} and the C–N–C angle, both represented in orange, are compressed together.

IR spectra of starting sulfonamide, amine and thioether:

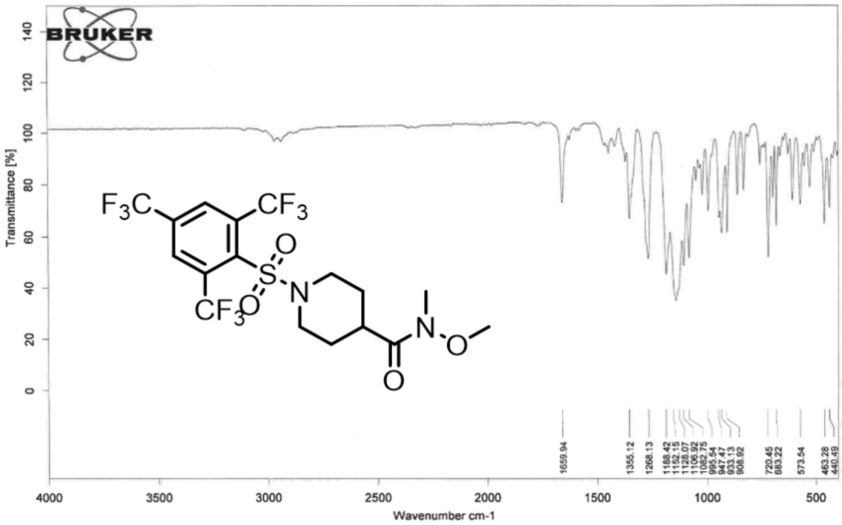

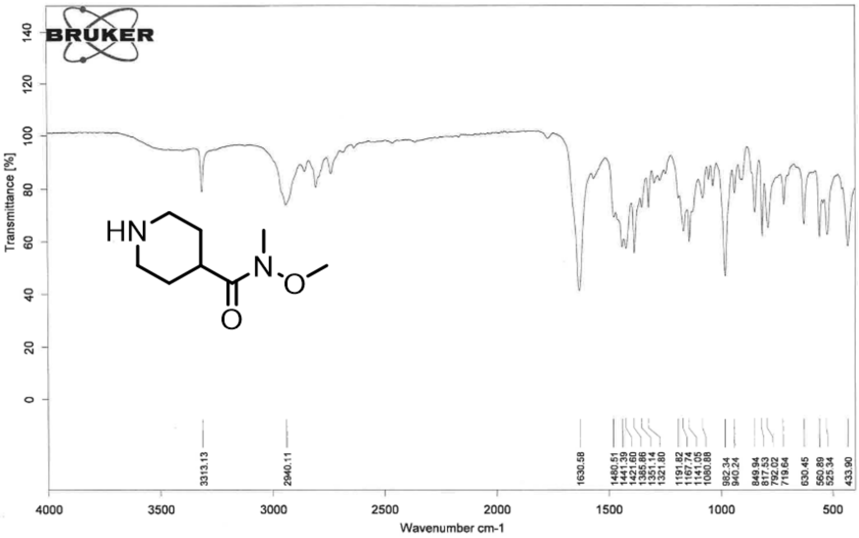

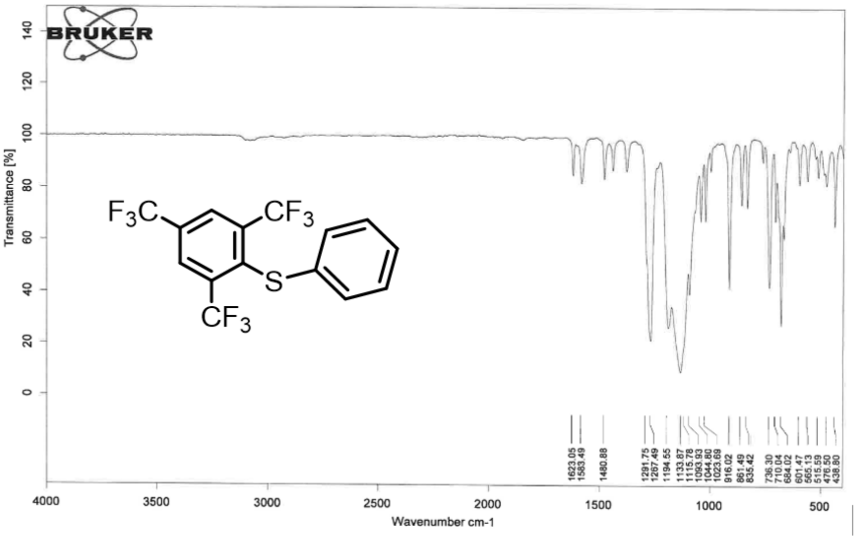

We also monitored the deprotection of sulfonamide **2z** employing *in situ* NMR techniques.

The deprotection of Nms amide **2z** under the standard conditions was studied by *in situ* ^{1}H NMR employing THF-d8 as solvent.

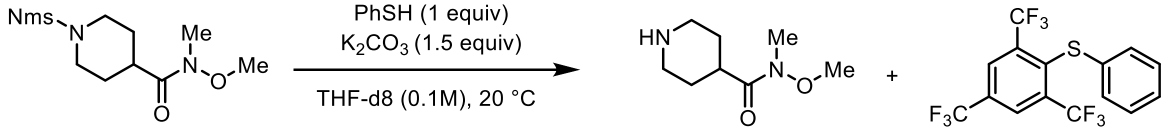

2z **1z** **5**

The consumption of sulfonamide **2z** was difficult to follow as all the signals overlapped with signals from the products **1z** and **5**. However, one of the signals from the diarylsulfide **5** [6.94 – 6.91 (m, 2H)] could be easily followed, which allowed to monitor the deprotection kinetics. The deprotection occurred relatively fast, as after 2 h, 90% of compound **5** was already detected. Quantitative formation of **5** was observed after 15 h.

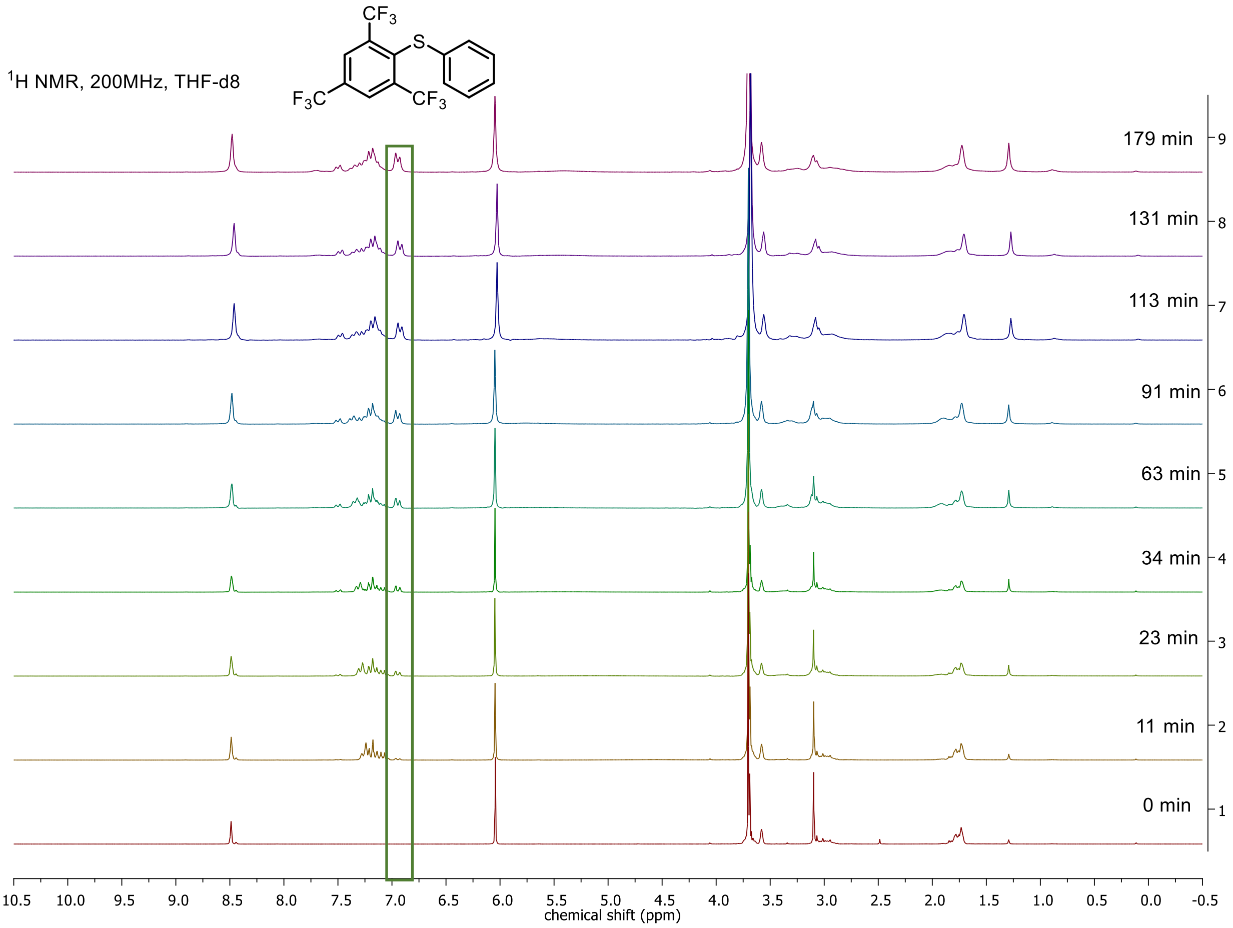

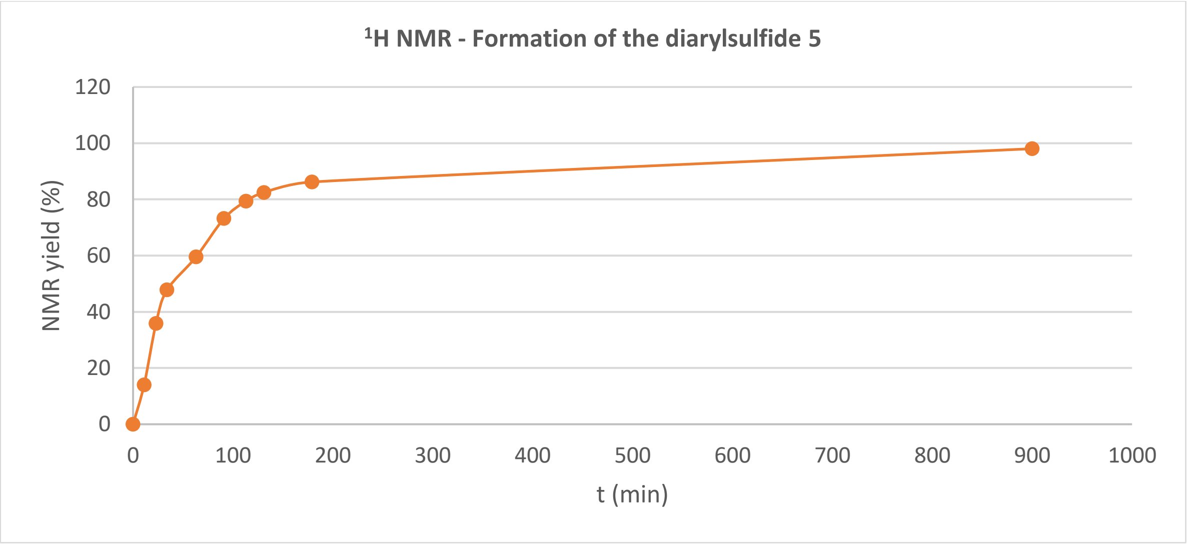

The deprotection of Nms amide **2z** was also possible in the absence of base. Employing 1.0 equiv. of PhSH, we detected 92% NMR yield of the amine **1z** after stirring the reaction at 20 °C for 16 h.

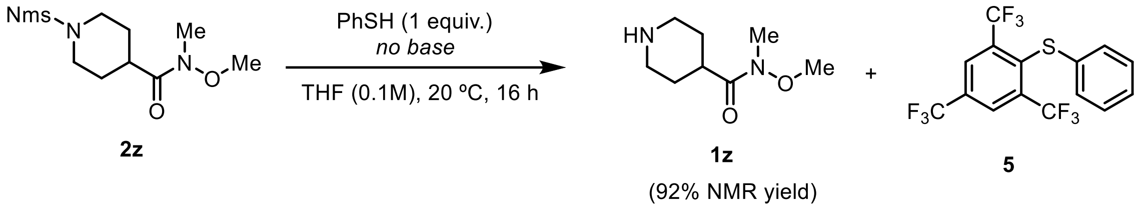

To gain more insight into the process, we decided to monitor the reaction by *in situ* ^{19}F NMR employing THF-d$_8$ as solvent and 1-bromo-2,6-di(trifluoromethyl)benzene as internal standard.

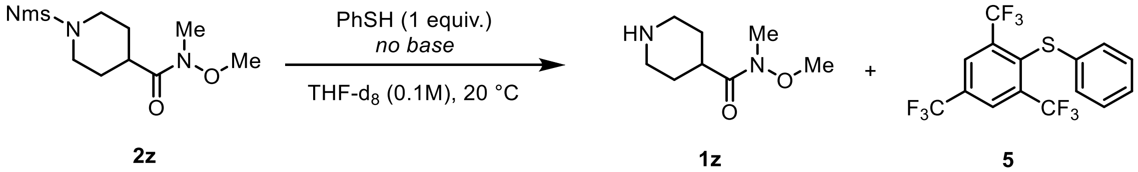

In this case, compound **2z** remained unaltered for 3 h, and then the reaction started to proceed. After this time, signals from compound **5** appeared and almost full conversion of **4s** was observed after 6 h of reaction. No signals matching the Meisenheimer complex could be detected during the experiment.

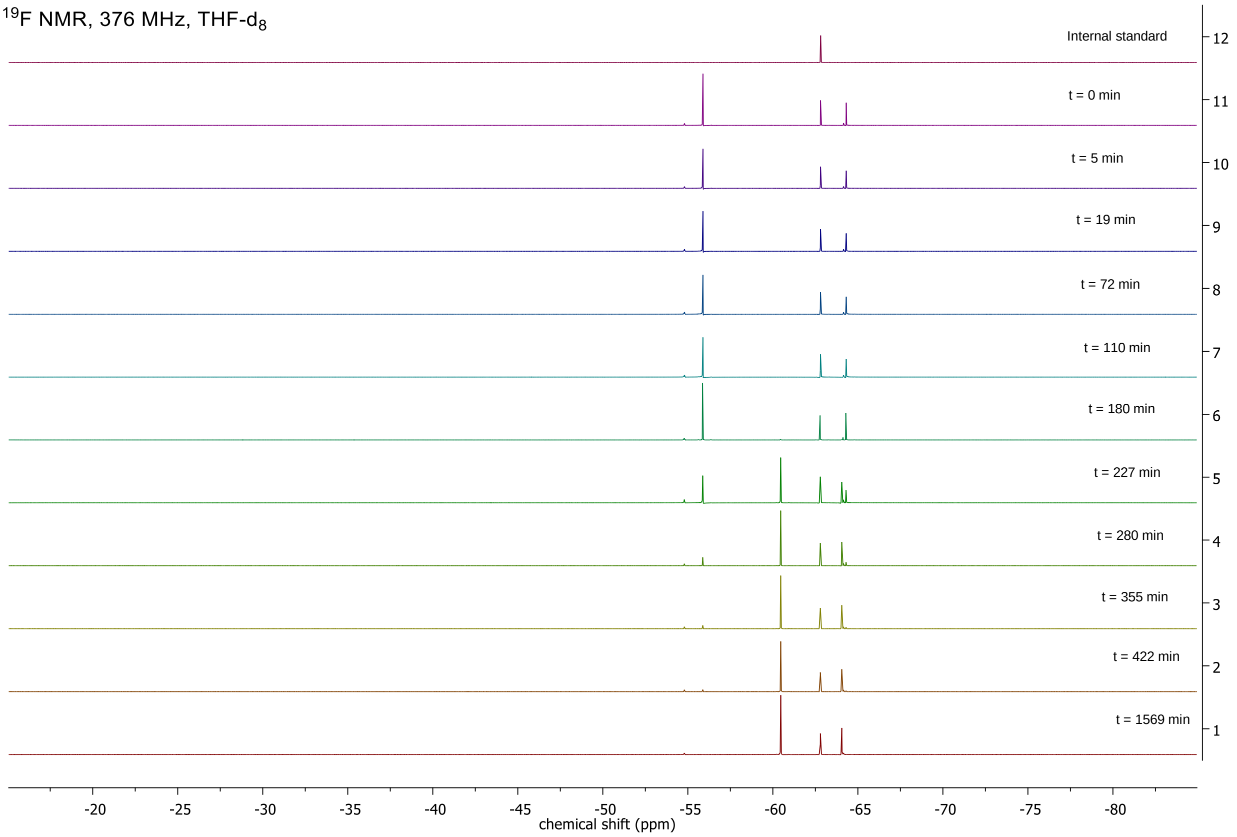
19F NMR, 376 MHz, THF-d8
Internal standard
t = 0 min
t = 5 min
t = 19 min
t = 72 min
t = 110 min
t = 180 min
t = 227 min
t = 280 min
t = 355 min
t = 422 min
t = 1569 min
chemical shift (ppm)

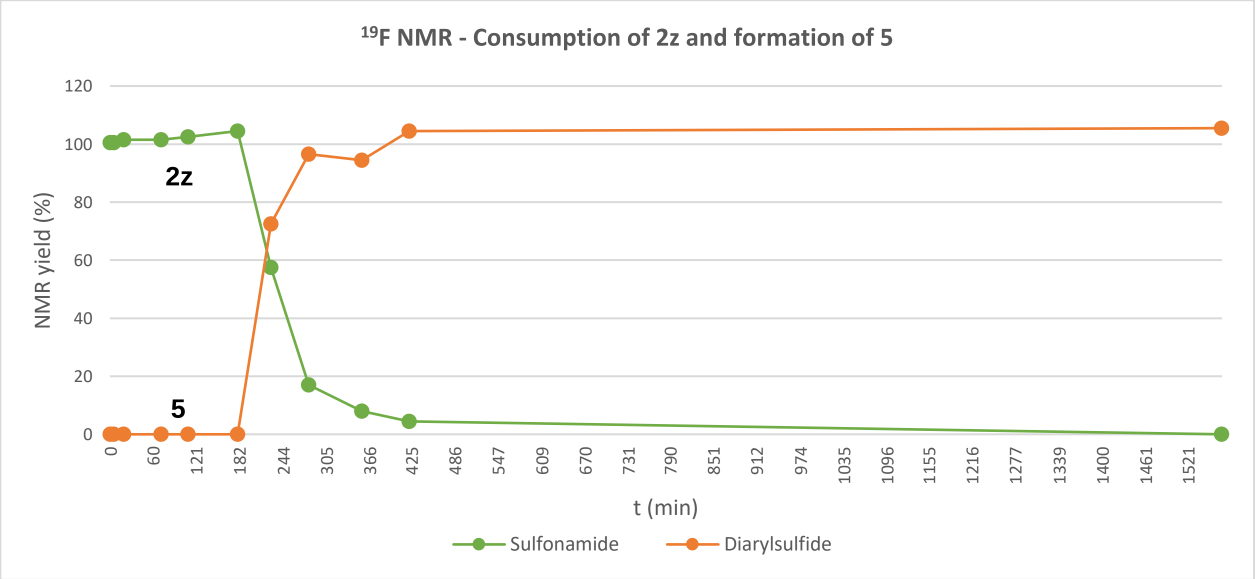
19F NMR - Consumption of 2z and formation of 5
NMR yield (%)
2z
5
t (min)
Sulfonamide
Diarylsulfide

3.4. Selective diamine deprotection

3.4.1 Orthogonal Boc deprotection of sulfonamide 2g

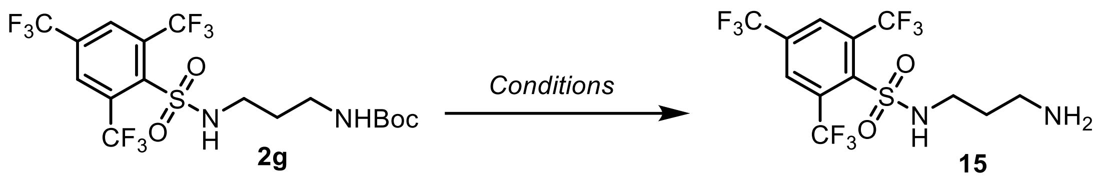

Procedure A: deprotection with HCl

Sulfonamide **2g** (53.0 mg, 0.10 mmol, 1.0 equiv.) was dissolved in dry MeCN (1.0 ml, 0.1 M). HCl (2 M in Et_2O, 0.33 ml, 0.66 mmol, 6.5 equiv.) was added and the mixture was stirred at room temperature for 2 h (after a few minutes a white solid formed). The solvent was removed under reduced pressure. A saturated aqueous solution of $NaHCO_3$ (10 ml) was added, followed by extraction with DCM (4 × 10 ml due to low solubility of the product). The combined organic layers were dried over $MgSO_4$ and concentrated under reduced pressure. Sulfonamide 15 was obtained as a white solid (43.5 mg, 0.10 mmol, >99%).

Procedure B: deprotection with TFA

Sulfonamide **2g** (397 mg, 0.77 mmol, 1.0 equiv.) was dissolved in dry DCM (0.1 M). TFA (0.57 ml, 7.7 mmol, 10 equiv.) was added. The mixture was stirred at room temperature for 2 h. Then it was neutralized with a saturated aqueous solution of $NaHCO_3$ at 0 °C. DCM was added and the aqueous layer was extracted (5 ×). The combined organic layers were dried over $MgSO_4$, filtered and concentrated under reduced pressure. Sulfonamide 15 was obtained as a white solid (315.9 mg, 0.76 mmol, 99%).

***N*-(3-Aminopropyl)-2,4,6-tris(trifluoromethyl)benzenesulfonamide (15)**

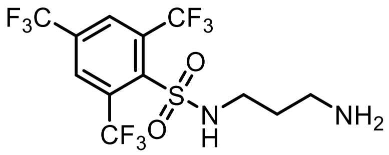

^{1}H NMR (700 MHz, MeOD): δ 8.44 (s, 2H), 3.09 (t, *J* = 6.8 Hz, 2H), 2.74 (t, *J* = 6.9 Hz, 2H), 1.71 (p, *J* = 6.8 Hz, 2H) ppm.

^{13}C NMR (176 MHz, MeOD): δ 148.5, 134.4 (q, *J* = 34.8 Hz), 133.5 (q, *J* = 33.6), 130.5, 123.8 (q, *J* = 274.4), 123.7 (q, *J* =272.4 Hz), 42.6, 39.6, 33.1 ppm.

^{19}F NMR (659 MHz, MeOD): δ -55.9 (6F), -65.0 (3F) ppm.

IR (neat): ν_{max} 3376, 1334, 1291, 1205, 1184, 1149 cm^{-1}.

HRMS (ESI$^+$): exact mass calculated for $[M+H]^+$ ($C_{12}H_{12}F_9N_2O_2S^+$) requires *m/z* 419.0470, found *m/z* 419.0471.

3.4.2 Orthogonal Nms cleavage in the presence of other sulfonamides

3.4.2.1 Preparation of starting materials

For this study following compounds **12-14** have been prepared using the below described general procedure.

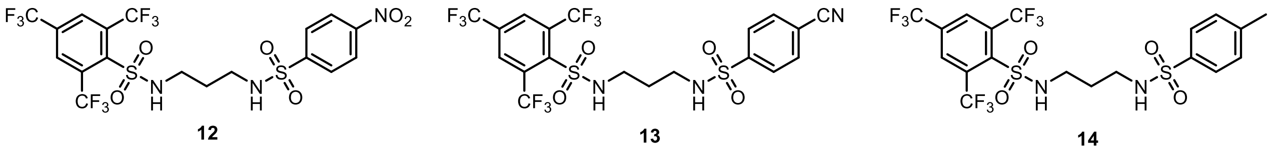

General Procedure:

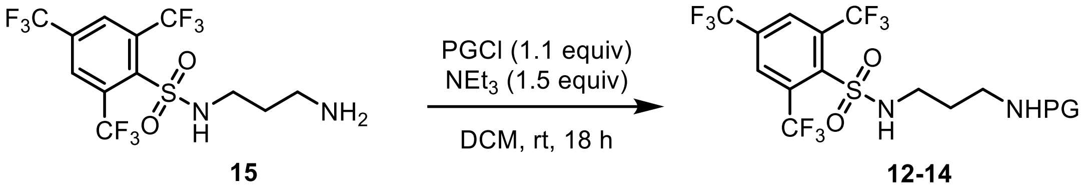

Sulfonamide **15** (1.0 equiv.) was suspended in dry DCM (0.1 M). Et_3N (1.5 equiv.) was added, followed by the corresponding sulfonyl chloride (1.1 equiv.). The reaction was run for 18 h, after which H_2O was added. The aqueous layer was extracted with DCM (3x). The combined organic layers were washed with brine, dried over $MgSO_4$, filtered and concentrated under reduced pressure. The residue was purified by column chromatography (silica gel, heptane:EtOAc).

N-(3-((4-Nitrophenyl)sulfonamido)propyl)-2,4,6-tris(trifluoromethyl)benzenesulfonamide (12)

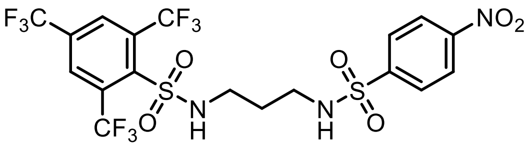

Following the general procedure using sulfonamide **15** (83.9 mg, 0.20 mmol), sulfonamide **12** was obtained as a white solid (97.7 mg, 0.16 mmol, 81%).

^{1}H NMR (700 MHz, d_6-DMSO): δ 8.57 (s, 2H), 8.43 – 8.37 (m, 2H), 8.35 (br, 1H), 8.01 – 7.99 (m, 2H), 7.97 (t, *J* = 5.7 Hz, 1H), 2.96 – 2.85 (m, 2H), 2.79 (dt, *J* = 6.7, 6.2 Hz, 2H), 1.63 – 1.54 (m, 2H) ppm.

^{13}C NMR (176 MHz, d_6-DMSO): δ 149.5, 146.0, 145.7, 132.5 (q, *J* = 34.7 Hz), 131.0 (q, *J* = 32.9 Hz), 130.1, 128.0, 124.6, 122.2 (q, *J* = 275.5 Hz), 122.0 (q, *J* = 273.3 Hz), 40.5, 40.1, 29.6 ppm.

^{19}F NMR (659 MHz, d_6-DMSO): δ -53.4 (6F), -61.9 (3F) ppm.

IR (neat): ν_{max} 3321, 1533, 1372, 1290, 1196, 1159 cm^{-1}.

HRMS (ESI$^+$): exact mass calculated for $[M+H]^+$ ($C_{18}H_{15}F_9N_3O_6S_2^+$) requires *m/z* 604.0253, found *m/z* 604.0256.

N-(3-((4-Cyanophenyl)sulfonamido)propyl)-2,4,6-tris(trifluoromethyl)benzenesulfonamide (13)

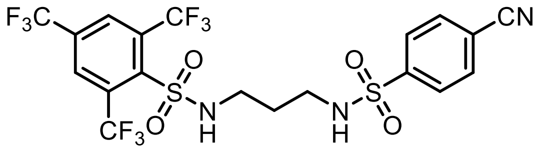

Following the general procedure using sulfonamide **15** (124.7 mg, 0.30 mmol), sulfonamide **13** was obtained as a white solid (155.1 mg, 0.27 mmol, 89%).

^{1}H NMR (700 MHz, MeOD): δ 8.47 (s, 2H), 7.99 – 7.94 (m, 2H), 7.94 – 7.90 (m, 2H), 3.07 (t, *J* = 7.0 Hz, 2H), 2.92 (t, *J* = 6.9 Hz, 2H), 1.70 (app p, *J* = 6.9 Hz, 2H) ppm.

^{13}C NMR (176 MHz, MeOD): δ 147.4, 146.2, 134.8 (q, *J* = 35.2 Hz), 134.3, 133.5 (q, *J* = 33.8 Hz), 130.8, 128.7, 123.8 (q, *J* = 275 Hz), 123.6 (q, *J* = 272 Hz), 118.5, 117.2, 41.8, 41.4, 31.4 ppm.

^{19}F NMR (659 MHz, MeOD): δ -55.9 (6F), -65.0 (3F) ppm.

IR (neat): ν_{max} 3363, 3275, 2234, 1286, 1183, 1157 cm^{-1}.

HRMS (ESI$^+$): exact mass calculated for $[M+H]^+$ ($C_{19}H_{15}F_9N_3O_4S_2^+$) requires *m/z* 584.0355, found *m/z* 584.0354.

N-(3-((4-Methylphenyl)sulfonamido)propyl)-2,4,6-tris(trifluoromethyl)benzenesulfonamide (14)

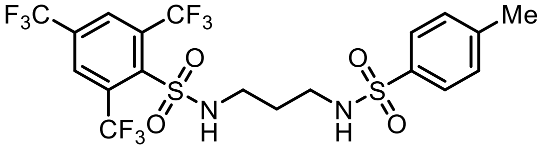

Following the general procedure using sulfonamide **15** (209.2 mg, 0.50 mmol), sulfonamide **14** was obtained as a white solid (258 mg, 0.45 mmol, 90%).

^{1}H NMR (600 MHz, MeOD): δ 8.46 (s, 2H), 7.69 (d, *J* = 8.3 Hz, 2H), 7.36 (d, *J* = 8.0 Hz, 2H), 3.08 (t, *J* = 7.0 Hz, 2H), 2.84 (t, *J* = 6.9 Hz, 2H), 2.42 (s, 3H), 1.68 (app p, *J* = 6.9 Hz, 2H) ppm. *The N-H were not observed in ^{1}H NMR.

^{13}C NMR (176 MHz, MeOD): δ 147.5, 144.7, 138.7, 134.8 (q, *J* = 34.8 Hz), 133.5 (q, *J* = 33.7 Hz), 130.9 – 130.6, 128.1, 128.0, 123.7 (q, *J* = 274.0 Hz), 123.6 (q, *J* = 274.0 Hz), 41.9, 41.3, 31.4, 21.4 ppm.

^{19}F NMR (565 MHz, MeOD): δ -55.9 (6F), -65.0 (3F) ppm.

IR (neat): ν_{max} 3326, 3271, 1290, 1197, 1155 cm^{-1}.

HRMS (ESI⁺): exact mass calculated for $[M+H]^+$ ($C_{19}H_{18}F_9N_2O_4S_2^+$) requires *m/z* 573.0559, found *m/z* 573.0558.

3.4.2.2. Deprotections

N-(3-Aminopropyl)-4-nitrobenzenesulfonamide (16)

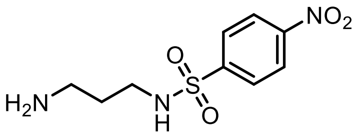

Following an adapted version of GP5 (18 h reaction time) using sulfonamide **12** (60.3 mg, 0.10 mmol), amine **16** was obtained as a white solid (21.0 mg, 0.081 mmol, 81%) after acid-base extraction (PP2).

^{1}H NMR (600 MHz, MeOD): δ 8.42 (d, *J* = 8.7 Hz, 2H), 8.10 (d, *J* = 8.7 Hz, 2H), 3.02 (t, *J* = 6.6 Hz, 2H), 2.97 (t, *J* = 7.7 Hz, 2H), 1.88 – 1.81 (m, 2H) ppm.

^{13}C NMR (151 MHz, MeOD): δ 151.5, 147.6, 129.4, 125.5, 41.1, 38.3, 29.5 ppm.

IR (neat): ν_{max} 1529, 1351, 1273, 1199, 1159 cm^{-1}.

HRMS (ESI⁺): exact mass calculated for $[M+H]^+$ ($C_9H_{14}N_3O_4S^+$) requires *m/z* 260.0700, found *m/z* 260.0701.

N-(3-Aminopropyl)-4-cyanobenzenesulfonamide (17)

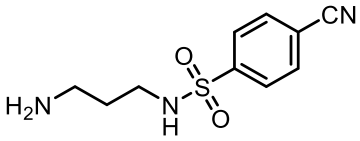

Following an adapted version of GP5 (18 h reaction time) using sulfonamide **13** (58.6 mg, 0.10 mmol), amine **17** was obtained as a white solid (18.9 mg, 0.079 mmol, 79%) after acid-base extraction (PP2).

^{1}H NMR (700 MHz, $CDCl_3$): δ 7.98 (d, *J* = 8.4 Hz, 2H), 7.81 (d, *J* = 8.4 Hz, 2H), 3.13 (t, *J* = 5.8 Hz, 2H), 2.85 (br s, 2H), 1.62 (dt, *J* = 11.3, 5.7 Hz, 2H) ppm.

^{13}C NMR (176 MHz, $CDCl_3$): δ 144.9, 133.0, 127.8, 117.6, 116.2, 43.9, 41.5, 30.1 ppm.

IR (neat): ν_{max} 3371, 3308, 2940, 2873, 2849, 2234, 1323, 1155, 1091, 632 cm^{-1}.

HRMS (ESI⁺): exact mass calculated for $[M+H]^+$ ($C_{10}H_{14}N_3O_2S^+$) requires *m/z* 240.0801, found *m/z* 240.0800.

***N*-(3-Aminopropyl)-4-methylbenzenesulfonamide (18)**

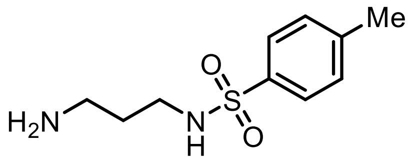

Following an adapted version of GP5 (18 h reaction time) using sulfonamide **14** (57.1 mg, 0.10 mmol), amine **18** was obtained as a white solid (19.3 mg, 0.08 mmol, 85%) after acid-base extraction (PP2).

^{1}H NMR (400 MHz, $CDCl_3$): δ 7.74 (d, *J* = 8.1 Hz, 2H), 7.29 (d, *J* = 8.0 Hz, 2H), 3.05 (t, *J* = 6.1 Hz, 2H), 2.78 (br s, 2H), 2.42 (s, 3H), 1.65 – 1.50 (m, 2H) ppm.

The spectroscopic data matched the data reported in the literature for this compound.[5]

4. Stability of Nms-amides

4.1 Case study - Grignard addition

4.1.1 Preparation of starting materials

Following the general procedure GP3, the sulfonamides shown below were prepared from the corresponding Weinreb amide amine (CAS: 160809-37-0). The Boc-protected amine was purchased from Sigma Aldrich (CAS: 139290-70-3).

For the preparation of sulfonamide **2z**, see previous protection section.

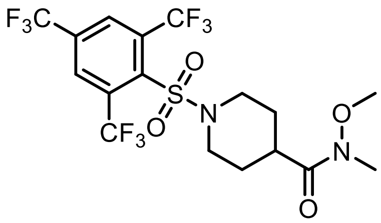

2z

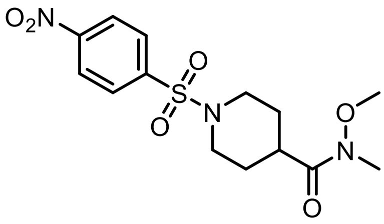

19

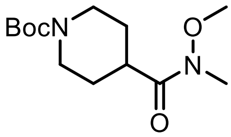

22

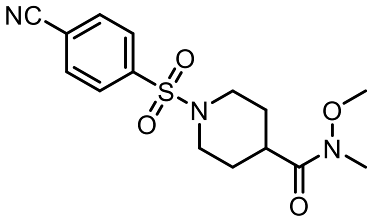

20

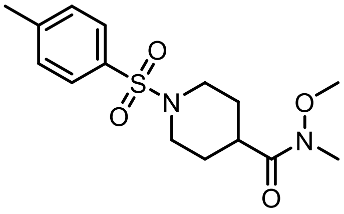

21

N-methoxy-N-methyl-1-((4-nitrophenyl)sulfonyl)piperidine-4-carboxamide (19)

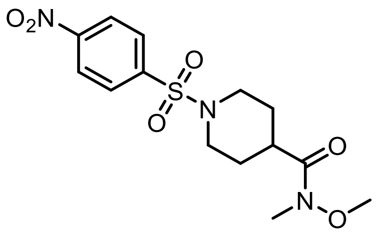

Following GP3 using *N*-methoxy-*N*-methylpiperidine-4-carboxamide hydrochloride (209 mg, 1.0 mmol), sulfonamide **19** was obtained as a white solid (209 mg, 0.585 mmol, 59%)

^{1}H NMR (400 MHz, CDCl$_3$): δ 8.36 (d, *J* = 8.8 Hz, 2H), 7.93 (d, *J* = 8.8 Hz, 2H), 3.84 – 3.70 (m, 2H), 3.62 (s, 3H), 3.12 (s, 3H), 2.70 – 2.59 (m, 1H), 2.59 – 2.44 (m, 2H), 1.93 – 1.72 (m, 4H) ppm.

^{13}C NMR (101 MHz, CDCl$_3$): δ 174.6, 150.2, 142.6, 128.8, 124.4, 61.6, 45.5, 36.7, 32.2, 27.5 ppm.

IR (neat): ν_{max} 1665, 1527, 1347, 1163, 929, 742, 598, 577 cm^{-1}.

HRMS (ESI$^+$): exact mass calculated for [M+H]$^+$ ($C_{14}H_{20}N_3O_6S^+$) requires m/z 358.1067, m/z found: 358.1065.

1-((4-Cyanophenyl)sulfonyl)-N-methoxy-N-methylpiperidine-4-carboxamide (20)

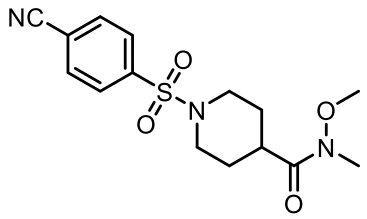

Following GP3 using *N*-methoxy-*N*-methylpiperidine-4-carboxamide hydrochloride (209 mg, 1.0 mmol), sulfonamide **20** was obtained as a white solid (184 mg, 0.545 mmol, 55%).

^{1}H NMR (400 MHz, CDCl$_3$): δ 7.90 – 7.78 (m, 4H), 3.75 (m, 2H), 3.62 (s, 3H), 3.12 (s, 3H), 2.70 – 2.56 (m, 1H), 2.56 – 2.46 (m, 2H), 1.89 – 1.72 (m, 4H) ppm.

^{13}C NMR (101 MHz, CDCl$_3$): δ 174.7, 141.0, 133.0, 128.2, 117.4, 116.5, 61.6, 45.5, 36.7, 32.2, 27.4 ppm.

IR (neat): ν_{max} 2229, 1644, 1345, 1161, 920, 727, 587, 513 cm^{-1}.

HRMS (ESI$^+$): exact mass calculated for [M+H]$^+$ ($C_{15}H_{20}N_3O_4S^+$) requires m/z 338.1169, m/z found: 338.1168.

***N*-Methoxy-*N*-methyl-1-tosylpiperidine-4-carboxamide (21)**

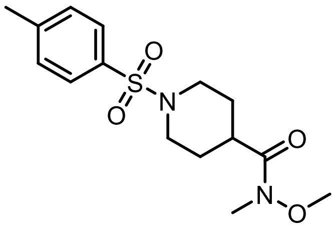

Following GP3 using *N*-methoxy-*N*-methylpiperidine-4-carboxamide hydrochloride (209 mg, 1.0 mmol), sulfonamide **21** was obtained as a as a white solid (235 mg, 0.72 mmol, 72%)

^{1}H NMR (400 MHz, CDCl$_3$): δ 7.63 (d, *J* = 8.2 Hz, 2H), 7.33 (d, *J* = 8.0 Hz, 2H), 3.82 – 3.72 (m, 2H), 3.63 (s, 3H), 3.14 (s, 3H), 2.67 – 2.54 (m, 1H), 2.43 (s, 3H), 2.41 – 2.29 (m, 2H), 1.92 – 1.73 (m, 4H) ppm.

^{13}C NMR (101 MHz, CDCl$_3$): δ 174.8, 143.5, 132.9, 129.6, 127.5, 61.4, 45.5, 37.0, 32.1, 27.4, 21.4 ppm.

IR (neat): ν_{max} 1649, 1348, 1326, 1160, 999, 929, 727, 650, 583, 546 cm^{-1}.

HRMS (ESI$^+$): exact mass calculated for [M+H]$^+$ ($C_{15}H_{23}N_2O_4S^+$) requires m/z 327.1373, m/z found: 327.1370.

4.1.2 General procedure

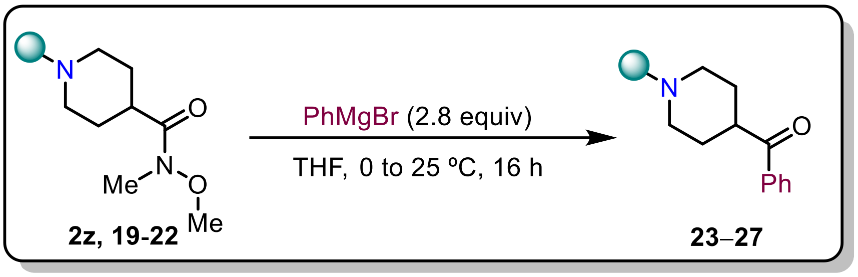

	23	24	25	26	27
	**Nms**	**4-Ns**	**4-Cs**	**Ts**	**Boc**
**Yield (%)**	82	nd	15	72	83

To a stirred solution of the corresponding Weinreb amide (0.1-0.2 mmol, 1.0 equiv.) in THF (0.1 M) at 0 °C, was added dropwise a solution of phenylmagnesium bromide (0.1-0.2 mL, 2.8 equiv., 2.8M in Et$_2$O). The resulting mixture was stirred for 16 h without removal of the ice-bath. The solution was quenched using a saturated solution of NH_4Cl and the aq. phase extracted twice with DCM. The combined organic phases were concentrated and the crude material analyzed by NMR spectroscopy and GCMS analysis. For further purification and identification of potential side products, the crude material was subjected to column chromatography.

4.1.3 Reactions results of different sulfonamides

With Nms

Phenyl(1-((2,4,6-tris(trifluoromethyl)phenyl)sulfonyl)piperidin-4-yl)methanone (23)

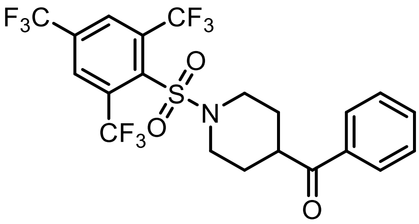

Following the general procedure using **2z** (51.6 mg, 0,.1 mmol, 1.0 equiv.), the title compound was obtained after column chromatography (heptane/EtOAc) as a white solid (43.7 mg, 82 µmol, 82%).

^{1}H NMR (400 MHz, CDCl$_3$): δ 8.27 (s, 2H), 7.92 (d, *J* = 7.4 Hz, 2H), 7.58 (t, *J* = 6.8 Hz, 1H), 7.48 (t, *J* = 7.2 Hz, 2H), 3.76 (d, *J* = 13.6 Hz, 2H), 3.56 – 3.47 (m, 1H), 3.21 – 3.10 (m, 2H), 2.02 – 1.92 (m, 4H) ppm.

^{13}C NMR (151 MHz, CDCl$_3$): δ 201.2, 145.2, 135.7, 134.2 (q, *J* = 35.6 Hz), 133.4, 133.2 (q, *J* = 34.0 Hz), 129.4, 129.0, 128.4, 122.2 (q, *J* = 275.7 Hz), 122.01 (q, *J* = 273.8 Hz), 46.1, 42.3, 28.2 ppm.

^{19}F NMR (377 MHz, CDCl$_3$): δ -55.37 (6F), -63.56 (3F) ppm.

IR (neat): ν_{max} 1668, 1356, 1191, 1181, 1153, 1135, 1109, 1084, 1057, 944, 912, 862, 723, 711, 683, 665 cm^{-1}.

HRMS (ESI$^+$): exact mass calculated for [M+H]$^+$ ($C_{21}H_{17}F_9NO_3S^+$) requires m/z 534.0780, found m/z 534.0776.

With Ns

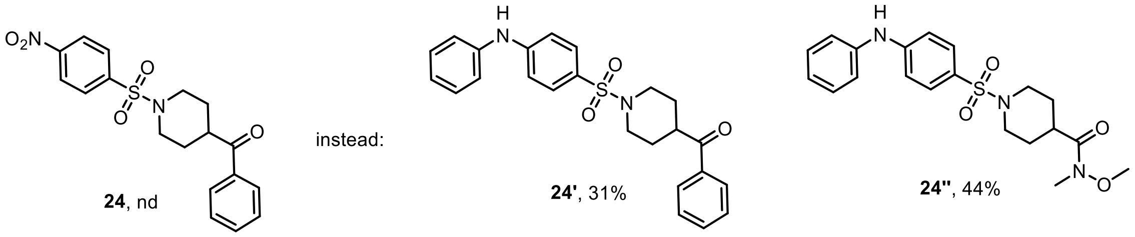

Following the general procedure using **19** (35.8 mg, 0,.1 mmol, 1.0 equiv.), the desired ketone was not obtained after column chromatography (heptane/EtOAc). Instead, two side products, **24’** as white solid (12.9 mg, 31 µmol, 31%; quickly turning to a red solid while standing at air) and **24’’** as a red liquid (17.9 mg, 44 µmol, 44%), were isolated.

Analytical data for **24’**: **Phenyl(1-((4-(phenylamino)phenyl)sulfonyl)piperidin-4-yl)methanone**

^{1}H NMR (600 MHz, CDCl$_3$): δ 7.87 (d, *J* = 7.4 Hz, 2H), 7.62 (d, *J* = 8.5 Hz, 2H), 7.55 (d, *J* = 7.3 Hz, 1H), 7.45 (s, 2H), 7.37 (t, *J* = 7.8 Hz, 2H), 7.20 (d, *J* = 7.7 Hz, 2H), 7.12 (t, *J* = 7.4 Hz, 1H), 7.05 (d, *J*

= 8.6 Hz, 2H), 6.13 (s, 1H), 3.76 (d, *J* = 11.8 Hz, 2H), 3.23 – 3.20 (m, 1H), 2.56 (td, *J* = 11.5, 2.6 Hz, 2H), 1.97 – 1.87 (m, 4H) ppm.

^{13}C NMR (151 MHz, CDCl$_3$): δ 201.4, 148.1, 140.2, 135.5, 133.1, 129.7, 129.5, 128.7, 128.1, 125.5, 123.7, 120.9, 114.5, 45.5, 42.3, 27.8 ppm.

IR (neat): ν_{max} 3354, 1710, 1679, 1584, 1336, 1321, 1152, 1093, 924, 733, 698, 585, 566 cm^{-1}.

HRMS (ESI$^+$): exact mass calculated for [M+H]$^+$ ($C_{24}H_{25}N_2O_3S^+$) requires m/z 421.1580, found m/z 421.1573.

Analytical data for **24''**: ***N*-Methoxy-*N*-methyl-1-((4-(phenylamino)phenyl)sulfonyl)piperidine-4-carboxamide**

^{1}H NMR (600 MHz, CDCl$_3$): δ 7.60 (d, *J* = 8.1 Hz, 2H), 7.36 (t, *J* = 7.6 Hz, 2H), 7.19 (d, *J* = 7.8 Hz, 2H), 7.11 (t, *J* = 7.3 Hz, 1H), 7.03 (d, *J* = 8.3 Hz, 2H), 6.15 (s, 1H), 3.79 – 3.74 (m, 2H), 3.65 (s, 3H), 3.15 (s, 3H), 2.61 (s, 1H), 2.46 – 2.39 (m, 2H), 1.87 – 1.79 (m, 4H) ppm.

^{13}C NMR (151 MHz, CDCl$_3$): δ 175.1, 148.1, 140.2, 129.7, 129.5, 125.5, 123.7, 120.9, 114.5, 61.5, 45.5, 37.1, 32.2, 27.4 ppm.

IR (neat): ν_{max} 2924, 2853, 1680, 1582, 1157, 925, 733 cm^{-1}.

HRMS (ESI$^+$): exact mass calculated for [M+H]$^+$ ($C_{20}H_{26}N_3O_4S^+$) requires m/z 404.1639, found m/z 404.1636.

With Cs

4-((4-Benzoylpiperidin-1-yl)sulfonyl)benzonitrile (25) and (1-((4-benzoylphenyl)sulfonyl)piperidin-4-yl)(phenyl)methanone (25')

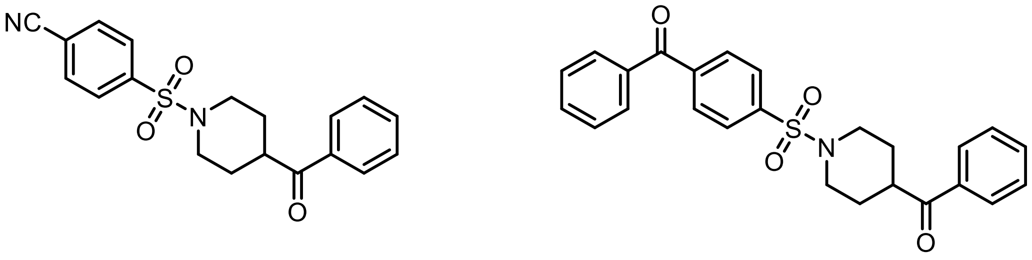

25 **25'**

Following the general procedure using **20** (33.7 mg, 0.1 mmol, 1.0 equiv.), the desired compound (**25**) was obtained after column chromatography (heptane/EtOAc) as a white solid (5.4 mg, 15 µmol, 15%). In addition, side product **25'** was isolated as a colorless oil (6.7 mg, 15 µmol, 15%).

<u>Analytical data for **25**:</u>

^{1}H NMR (400 MHz, CDCl$_3$): δ 7.93 – 7.87 (m, 2H), 7.87 – 7.83 (m, 4H), 7.60 – 7.53 (m, 1H), 7.45 (t, *J* = 7.6 Hz, 2H), 3.77 (dt, *J* = 11.9, 3.8 Hz, 2H), 3.32 – 3.21 (m, 1H), 2.67 (td, *J* = 11.6, 3.2 Hz, 2H), 2.02 – 1.83 (m, 4H) ppm.

^{13}C NMR (101 MHz, CDCl$_3$): δ 201.2, 141.1, 135.6, 133.6, 133.1, 129.0, 128.3, 117.4, 116.8, 45.5, 42.0, 27.9 ppm.

IR (neat): ν_{max} 1680, 1358, 1342, 1282, 1164, 926, 727 cm^{-1}.

HRMS (ESI⁺): exact mass calculated for $[M+H]^+$ ($C_{19}H_{19}N_2O_3S^+$) requires m/z 355.1111, found m/z 355.1109.

Analytical data for **25'**:

^{1}H NMR (400 MHz, $CDCl_3$): δ 7.97 – 7.88 (m, *J* = 8.4 Hz, 4H), 7.88 – 7.80 (m, 4H), 7.66 (t, *J* = 7.4 Hz, 1H), 7.59 – 7.51 (m, *J* = 11.1, 7.5 Hz, 3H), 7.45 (t, *J* = 7.7 Hz, 2H), 3.83 (dd, *J* = 8.1, 4.0 Hz, 2H), 3.28 – 3.21 (m, 1H), 2.72 – 2.61 (m, 2H), 2.02 – 1.86 (m, 4H) ppm.

^{13}C NMR (101 MHz, $CDCl_3$): δ 201.4, 195.5, 141.6, 139.8, 136.6, 135.7, 133.5, 130.5, 130.3, 129.0, 128.8, 128.3, 127.8, 45.7, 42.3, 28.0 ppm.

IR (neat): ν_{max} 1682, 1660, 1276, 1167, 923, 738, 700 cm^{-1}.

HRMS (ESI⁺): exact mass calculated for $[M+H]^+$ ($C_{25}H_{24}NO_4S^+$) requires m/z 434.1421, found m/z 434.1417.

With Ts

Phenyl(1-tosylpiperidin-4-yl)methanone (26) and phenyl(1-phenylpiperidin-4-yl)methanone (26')

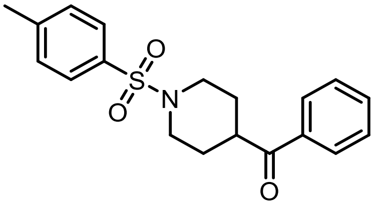

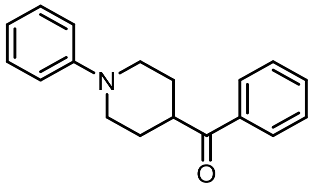

26 **26'**

Following the general procedure using **22** (32.6 mg, 0.1 mmol, 1.0 equiv.), the title compound was obtained after column chromatography (heptane/EtOAc) as a white solid (24.7 mg, 72 µmol, 72%). In addition, side product **26'** was isolated as a white solid (1.6 mg, 6 µmol, 6%).

Analytical data for **26**:

^{1}H NMR (400 MHz, $CDCl_3$): δ 7.84 (d, J = 7.4 Hz, 2H), 7.66 (d, J = 8.2 Hz, 2H), 7.54 (t, J = 7.4 Hz, 1H), 7.43 (t, J = 7.8 Hz, 2H), 7.34 (d, J = 8.4 Hz, 2H), 3.77 (dt, J = 11.6, 3.4 Hz, 2H), 3.24 – 3.14 (m, 1H), 2.58 – 2.49 (m, 2H), 2.45 (s, 3H), 1.98 – 1.84 (m, 4H) ppm.

^{13}C NMR (101 MHz, $CDCl_3$): δ 201.5, 143.7, 135.7, 133.4, 133.3, 129.8, 128.9, 128.3, 127.9, 45.7, 42.4, 28.0, 21.7 ppm.

IR (neat): ν_{max} 1674, 1353, 1336, 1284, 1161, 1141, 1091, 925, 723, 696, 582, 547 cm^{-1}.

HRMS (ESI⁺): exact mass calculated for $[M+Na]^+$ ($C_{19}H_{21}NNaO_3S^+$) requires m/z 366.1140, found m/z 3666.1133.

Analytical data for **26'**:

^{1}H NMR (600 MHz, CDCl$_3$): δ 7.98 – 7.96 (m, 2H), 7.59 – 7.56 (m, 1H), 7.49 (t, *J* = 7.7 Hz, 2H), 7.29 – 7.26 (m, *J* = 7.0, 1.6 Hz, 2H), 6.97 (d, *J* = 7.9 Hz, 2H), 6.86 (t, *J* = 7.3 Hz, 1H), 3.77 (dt, *J* = 5.9, 2.7 Hz, 2H), 3.39 (dt, *J* = 15.1, 5.4 Hz, 1H), 3.42 – 3.36 (m, J = 15.1, 5.4 Hz, 1H), 2.88 (td, *J* = 12.0, 3.4 Hz, 2H), 2.03 – 1.94 (m, 4H) ppm.

^{13}C NMR (151 MHz, CDCl$_3$): δ 202.6, 151.7, 136.2, 133.2, 129.3, 128.9, 128.4, 119.8, 116.8, 49.6, 43.7, 28.8 ppm.

IR (neat): ν_{max} 1667, 1596, 1502, 1447, 1389, 1296, 1239, 980, 701 cm^{-1}.

HRMS (ESI$^+$): exact mass calculated for $[M+H]^+$ ($C_{18}H_{20}NO^+$) requires m/z 266.1539, found m/z 266.1541.

With Boc

tert-Butyl 4-benzoylpiperidine-1-carboxylate (27)

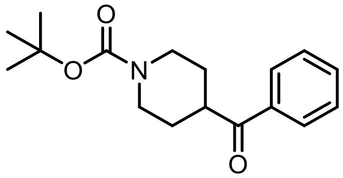

Following the general procedure using **22** (54.5 mg, 0.2 mmol, 1.0 equiv.), the title compound was obtained after column chromatography (heptane/EtOAc) as colorless oil (47.8 mg, 165 µmol, 83%).

^{1}H NMR (400 MHz, CDCl$_3$): δ 7.97 – 7.91 (m, 2H), 7.57 (dd, J = 8.3, 6.4 Hz, 1H), 7.47 (t, J = 7.7 Hz, 2H), 4.15 (s, 2H), 3.40 (tt, J = 11.1, 3.7 Hz, 1H), 2.90 (t, J = 12.1 Hz, 2H), 1.90 – 1.80 (m, J = 12.1 Hz, 2H), 1.78 – 1.65 (m, J = 21.2, 7.8 Hz, 2H), 1.46 (s, 9H) ppm.

^{13}C NMR (101 MHz, CDCl$_3$): δ 202.2, 154.9, 136.0, 133.2, 128.9, 128.4, 79.8, 43.6, 28.6 ppm.

Spectral data was found in agreement with the literature.[6]

4.1.4 Other Grignard reagent (MeMgBr)

1-(1-((2,4,6-Tris(trifluoromethyl)phenyl)sulfonyl)piperidin-4-yl)ethan-1-one (28)

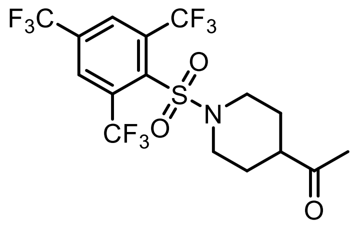

Following the general procedure using **2z** (51.6 mg, 0,.1 mmol, 1.0 equiv.) and methylmagnesium bromide (0.1 mL, 2.8M in Et_2O, 0.28 mmol, 0.28 equiv.), the title compound was obtained after column chromatography (heptane/EtOAc) as a white solid (40.0 mg, 85 µmol, 85%).

^{1}H NMR (400 MHz, $CDCl_3$): δ 8.26 (s, 2H), 3.71 (dt, *J* = 13.8, 3.4 Hz, 2H), 3.05 – 2.95 (m, 2H), 2.59 – 2.49 (m, *J* = 10.8, 3.9 Hz, 1H), 2.16 (s, 3H), 1.98 – 1.89 (m, 2H), 1.84 – 1.73 (m, 2H) ppm.

^{13}C NMR (101 MHz, $CDCl_3$): δ 209.1, 145.1, 134.3 (q, *J* = 35.2 Hz), 133.1 (q, *J* = 33.9 Hz), 129.4, 122.2 (q, *J* = 275.7 Hz), 122.0 (q, *J* = 273.5 Hz), 48.2, 46.2, 27.8, 27.4 ppm.

^{19}F NMR (376 MHz, $CDCl_3$): δ -55.47 (6F), -63.61 (3F) ppm.

IR (neat): ν_{max} 1708, 1349, 1296, 1272, 1171, 944, 917, 721 cm^{-1}.

HRMS (ESI^{+}): exact mass calculated for $[M+H]^{+}$ ($C_{16}H_{15}F_9NO_3S^{+}$) requires m/z 472.0623, found m/z 472.0622.

4.2 Case study - Hydrogenation

4.2.1 Preparation of starting materials

Following the general procedure GP3, below shown sulfonamides were prepared for this comparative reductive study. For the preparation of sulfonamide **2ac**, see previous description.

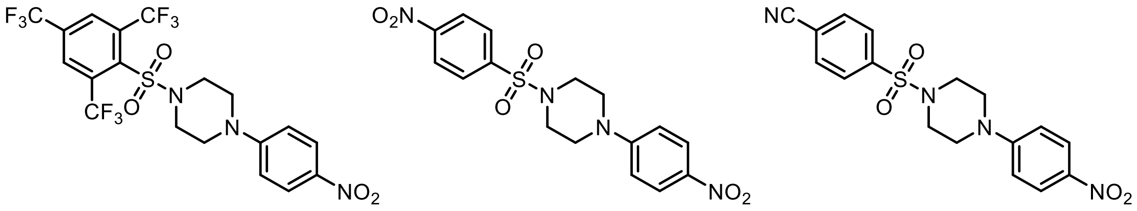

2ac **29** **30**

1-(4-Nitrophenyl)-4-((4-nitrophenyl)sulfonyl)piperazine (29)

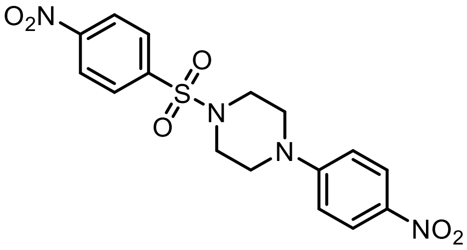

Following GP3 using 1-(4-nitrophenyl)piperazine (207 mg, 1 mmol) provided the titled compound as a yellow solid (262 mg, 0.668 mmol, 67%).

^{1}H NMR (400 MHz, CDCl$_3$): δ 8.41 (d, *J* = 8.9 Hz, 2H), 8.11 (d, *J* = 9.4 Hz, 2H), 7.98 (d, *J* = 8.9 Hz, 2H), 6.80 (d, *J* = 9.4 Hz, 2H), 3.55 – 3.49 (m, 4H), 3.28 – 3.21 (m, 4H) ppm.

^{13}C NMR (151 MHz, CDCl$_3$): δ 154.2, 150.6, 141.7, 139.9, 129.1, 126.1, 124.7, 114.0, 47.3, 45.6 ppm.

IR (neat): ν_{max} 1519, 1346, 1327, 1309, 1248, 1165, 1111, 950, 752, 600, 575 cm^{-1}.

HRMS (ESI$^+$): exact mass calculated for $[M+H]^+$ ($C_{16}H_{17}N_4O_6S^+$) requires m/z 393.0863, m/z found: 393.0863.

4-((4-(4-Nitrophenyl)piperazin-1-yl)sulfonyl)benzonitrile (30)

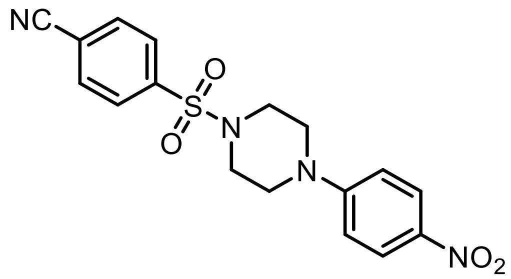

Following GP3 using 1-(4-nitrophenyl)piperazine (207 mg, 1.00 mmol), sulfonamide **30** was obtained as a yellow solid (375 mg, 1.00 mmol, 100%).

^{1}H NMR (400 MHz, $CDCl_3$): δ 8.12 (d, *J* = 9.3 Hz, 2H), 7.93 – 7.85 (m, 4H), 6.80 (d, *J* = 9.3 Hz, 2H), 3.53 – 3.47 (m, 4H), 3.26 – 3.17 (m, 4H) ppm.

^{13}C NMR (101 MHz, $CDCl_3$): δ 154.2, 140.1, 139.9, 133.2, 128.5, 126.1, 117.3, 117.2,113.9, 47.3, 45.6 ppm.

IR (neat): ν_{max} 1590, 1488, 1363, 1322, 1275, 1166, 954, 835, 619 cm^{-1}.

HRMS (ESI^{+}): exact mass calculated for $[M+H]^{+}$ ($C_{17}H_{16}N_4NaO_4S^{+}$) requires m/z 395.0790, m/z found: 395.0785.

4.2.2 General procedure

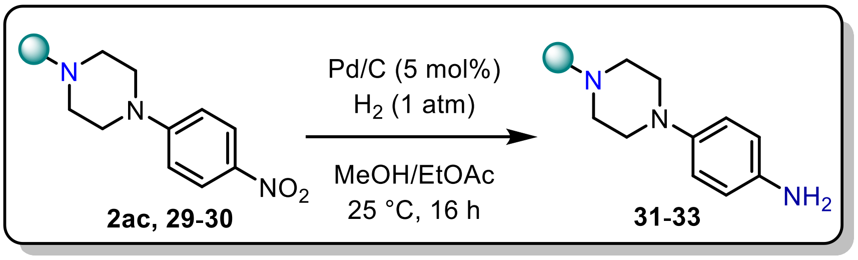

	31	32	33
	**Nms**	**Ns**	**4-Cs**
**Yield (%)**	96	nd	nd

A flame-dried Schlenk flask under argon atmosphere was loaded with Pd/C (53.2 mg, 10 % in charcoal, 0.5 equiv.) and the corresponding sulfonamide (1.0 equiv.). Subsequently, dry EtOAc (1 mL) and MeOH (1 mL) were added. The mixture was adjusted under H_2 atmosphere by blowing H_2 gas with a H_2 balloon for 30 min. The reaction was stirred for further 16 h. The crude material was passed through a Celite pad using MeOH and acetone as transfer solvent. The filtrate was concentrated and analyzed by NMR spectroscopy.

4.2.3 Reactions results of different sulfonamides

With Nms

4-(4-((2,4,6-tris(Trifluoromethyl)phenyl)sulfonyl)piperazin-1-yl)aniline (31)

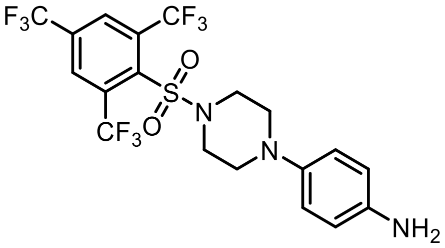

Following the general procedure using **2ac** (55.1 mg, 0.1 mmol, 1.0 equiv.), the title compound was obtained as a white solid (50.0 mg, 0.096 mmol, 96%).

^{1}H NMR (600 MHz, Acetone-d6): δ 8.58 (s, 2H), 6.78 (d, *J* = 8.6 Hz, 2H), 6.61 (d, *J* = 8.5 Hz, 2H), 4.27 (s, 2H), 3.49 – 3.46 (m, 4H), 3.06 – 3.03 (m, 4H) ppm.

^{13}C NMR (151 MHz, Acetone-d6): δ 145.7, 144.0, 143.9, 134.7 (d, *J* = 35.0 Hz), 133.4 (d, *J* = 34.0 Hz), 131.0, 120.3, 116.0.

^{19}F NMR (565 MHz, Acetone-d6): δ -55.55 (6F), -64.07 (3F) ppm.

IR (neat): ν_{max} 1518, 1345, 1282, 1268, 1192, 1154, 1137, 1110, 954, 914, 725, 536 cm^{-1}.

HRMS (ESI$^+$): exact mass calculated for $[M+H]^+$ ($C_{19}H_{17}F_9N_3O_2S^+$) requires m/z 522.0892, found m/z 522.0883.

With Ns

Following the general procedure using **29** (39.2 mg, 0.1 mmol, 1.0 equiv.), a white solid (28.2 mg, 0.085 mmol, 85%) was obtained after Celite filtration of the crude material, which represented the diamine detected by NMR and mass spectroscopy (see below) with some minor impurities of unknown structure.

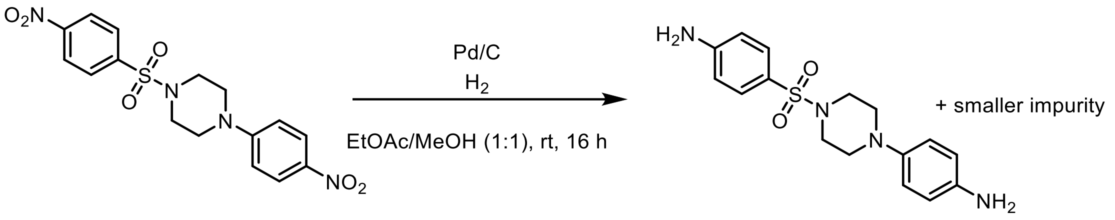

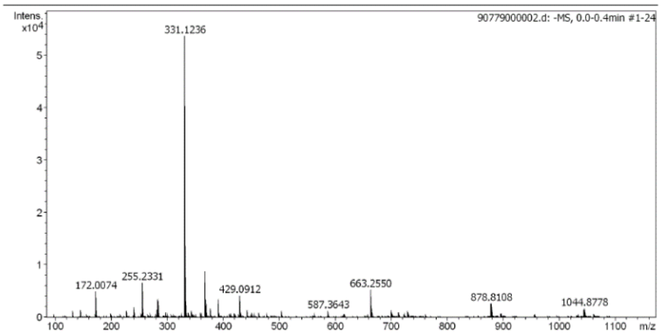

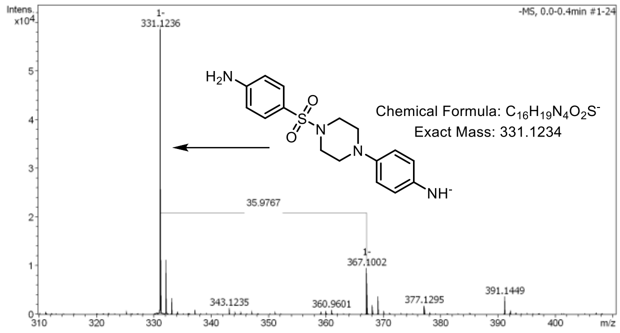

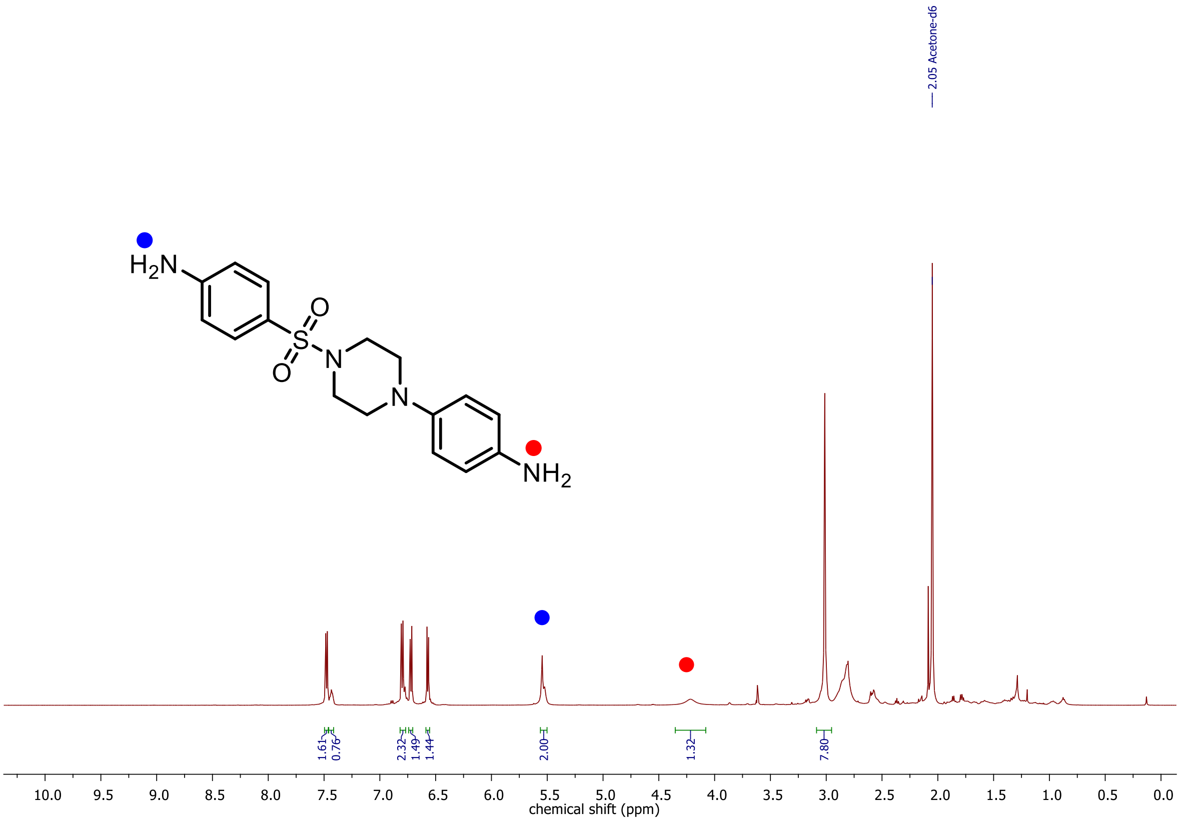

With Cs

4-(4-Tosylpiperazin-1-yl)aniline (33')

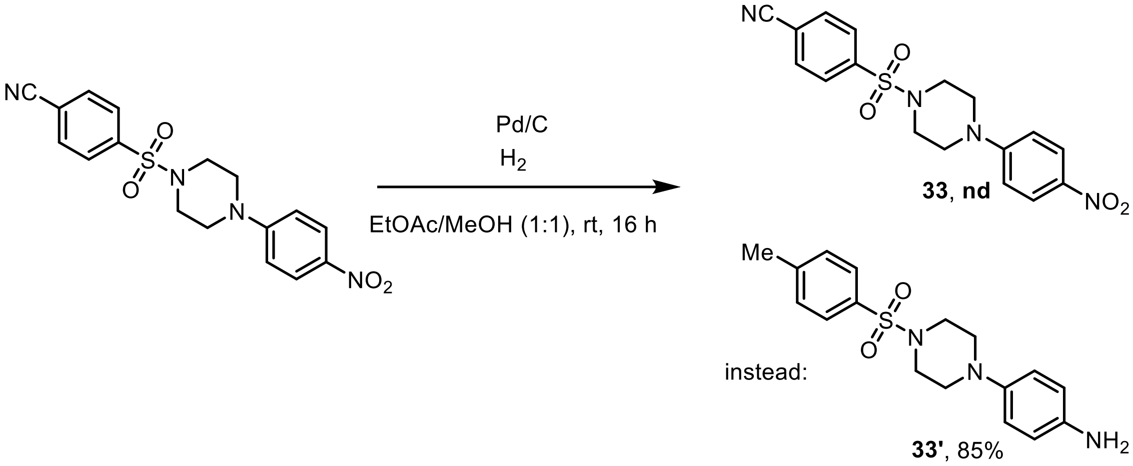

Following the general procedure using **30** as starting material, Ts-sulfonamide **33'** (28.2 mg, 0.085 mmol, 85%) rather than the desired Cs-sulfonamide **33** was obtained.

Analytical data for **33'**:

^{1}H NMR (700 MHz, Acetone-d$_6$): δ 7.69 (d, *J* = 8.3 Hz, 2H), 7.48 (d, *J* = 8.0 Hz, 2H), 6.73 – 6.69 (m, 2H), 6.59 – 6.54 (m, 2H), 4.24 (s, 2H), 3.09 – 3.04 (m, 4H), 3.04 – 3.01 (m, 4H) ppm.

^{13}C NMR (176 MHz, Acetone-d$_6$): δ 144.7, 143.9, 143.7, 133.8, 130.6, 128.8, 120.2, 115.9, 51.7, 47.4, 21.4 ppm.

IR (neat): ν_{max} 1515, 1321, 1157, 1120, 959, 829, 815, 728, 653 cm^{-1}.

HRMS (ESI$^+$): exact mass calculated for [M-H]$^-$ ($C_{17}H_{20}N_3O_2S^-$) requires m/z 330.1282, found m/z 330.1284.

4.3 Case Study – Hydride Reduction

4.3.1 Preparation of starting materials

For the preparation of sulfonamide **2y**, see previous protection section. Compounds **34-36** were prepared following this general procedure:

Ethyl isonipecotate (1.0 equiv.) was dissolved in THF (0.3 M) at room temperature (18-25 °C). Et_3N (2.0 equiv.) and the corresponding arenesulfonyl chloride (1.2 equiv.) were added sequentially to the solution at 0 °C. The mixture was stirred at 0 °C for 15 min, then at room temperature (23-25 °C) for 4 h. The progress of the reaction was monitored by TLC analysis. Then, the solvents were removed under vacuum. The residue was purified by column chromatography (silica gel, heptane:EtOAc).

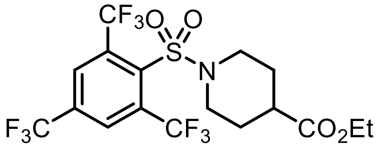

2y

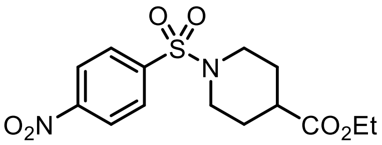

34

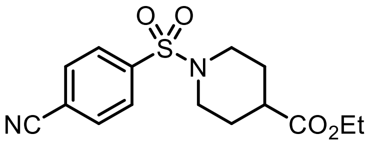

35

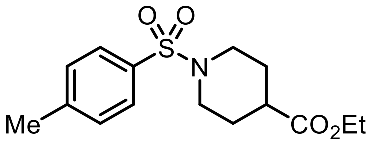

36

Ethyl 1-((4-nitrophenyl)sulfonyl)piperidine-4-carboxylate (34)

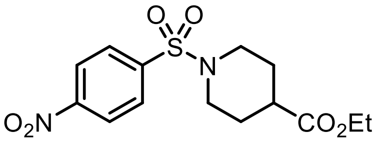

Following the general procedure using ethyl isonipecotate (78.6 µL, 80.2 mg, 0.5 mmol) and *p*-nitrobenzenesulfonyl chloride (133.0 mg, 0.6 mmol), sulfonamide **34** was obtained as a white solid (163.9 mg, 0.479 mmol, 96%).

^{1}H NMR (400 MHz, $CDCl_3$): δ 8.38 (d, *J* = 8.8 Hz, 2H), 7.95 (d, *J* = 8.8 Hz, 2H), 4.11 (q, *J* = 7.1 Hz, 2H), 3.64 (dt, *J* = 11.8, 3.9 Hz, 2H), 2.68 – 2.58 (m, 2H), 2.34 – 2.26 (m, 1H), 2.06 – 1.95 (m, 2H), 1.91 – 1.77 (m, 2H), 1.22 (t, *J* = 7.1 Hz, 3H) ppm.

^{13}C NMR (176 MHz, $CDCl_3$): δ 173.6, 150.3, 142.7, 128.8, 124.5, 60.9, 45.4, 39.9, 27.5, 14.3 ppm.

All NMR data was in accordance with the literature.[7]

Ethyl 1-((4-cyanophenyl)sulfonyl)piperidine-4-carboxylate (35)

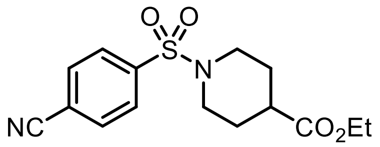

Following the general procedure using ethyl isonipecotate (78.6 µL, 80.2 mg, 0.5 mmol) and *p*-cyanobenzenesulfonyl chloride (121.0 mg, 0.6 mmol), sulfonamide **35** was obtained as a white solid (102.7 mg, 0.319 mmol, 64%).

^{1}H NMR (400 MHz, $CDCl_3$): δ 7.87 (d, *J* = 8.6 Hz, 2H), 7.83 (d, *J* = 8.6 Hz, 2H), 4.11 (q, *J* = 7.1 Hz, 2H), 3.67 – 3.57 (m, 2H), 2.60 (td, *J* = 12.0, 3.0 Hz, 2H), 2.33 – 2.26 (m, 1H), 2.02 – 1.96 (m, 2H), 1.87 – 1.77 (m, 2H), 1.22 (t, *J* = 7.1 Hz, 3H) ppm.

^{13}C NMR (176 MHz, $CDCl_3$): δ 173.7, 141.1, 133.1, 128.7, 117.4, 116.7, 60.9, 45.4, 39.9, 27.5, 14.3 ppm.

IR (neat): ν_{max} 2925, 2236, 1724, 1351, 1160, 925, 583 cm^{-1}.

HRMS (ESI$^+$): exact mass calculated for $[M+Na]^+$ ($C_{15}H_{18}N_2O_4SNa^+$) requires *m/z* 345.0879, found *m/z* 345.0877.

Ethyl 1-tosylpiperidine-4-carboxylate (36)

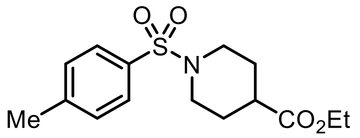

Following the general procedure (with 1.5 equiv. of sulfonyl chloride and 1.5 equiv. of Et_3N) using ethyl isonipecotate (78.6 µL, 80.2 mg, 0.5 mmol) and *p*-toluenebenzenesulfonyl chloride (143.0 mg, 0.75 mmol), sulfonamide **36** was obtained as a white solid (126.9 mg, 0.408 mmol, 81%).

^{1}H NMR (400 MHz, $CDCl_3$): δ 7.64 (d, *J* = 8.3 Hz, 2H), 7.32 (d, *J* = 8.0 Hz, 2H), 4.10 (q, *J* = 7.1 Hz, 2H), 3.62 (dt, *J* = 11.9, 3.7 Hz, 2H), 2.52 – 2.44 (m, 2H), 2.43 (s, 3H), 2.28 – 2.26 (m, 1H), 1.99 – 1.92 (m, 2H), 1.87 – 1.75 (m, 2H), 1.22 (t, *J* = 7.1 Hz, 3H) ppm.

^{13}C NMR (176 MHz, $CDCl_3$): δ 174.0, 143.7, 133.3, 129.8, 127.8, 60.8, 45.5, 40.2, 27.6, 21.7, 14.3 ppm.

IR (neat): ν_{max} 2845, 1719, 1594, 1156, 930, 709, 542 cm^{-1}.

HRMS (ESI$^+$): exact mass calculated for $[M+Na]^+$ ($C_{15}H_{21}NO_4SNa^+$) requires *m/z* 334.1083, found *m/z* 334.1081.

4.3.2 General Procedure

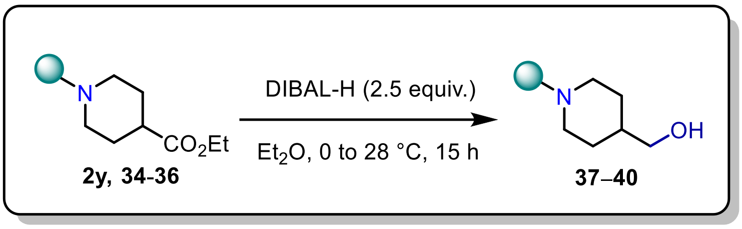

	37	38	39	40
	Nms	Ns	4-Cs	Ts
Yield (%)	75	80	nd	74

The corresponding ester (1.0 equiv.) was dissolved in dry Et_2O (0.1 M). The mixture was cooled to 0 °C and then DIBAL (1M in hexanes, 2.5 equiv.) was added dropwise. The reaction was stirred at 0 °C for 10 min, then at room temperature (23-25 °C) for 2 h. A sat., aq. solution of the Rochelle salt (1 mL for 0.1 mmol of ester) and EtOAc (1 mL for 0.1 mmol of ester) were added to the mixture. The layers were separated and the aqueous layer was extracted with EtOAc (2 x 1 mL for 0.1 mmol of ester). The combined organic layers were dried over $MgSO_4$, filtered and the solvents were removed under vacuum. The residue was purified by column chromatography (silica gel, hexane:ethyl acetate).

4.3.3 Reaction results of different sulfonamides

With Nms

[1-((2,4,6-Tris(Trifluoromethyl)phenyl)sulfonyl)piperidin-4-yl]methanol (37)

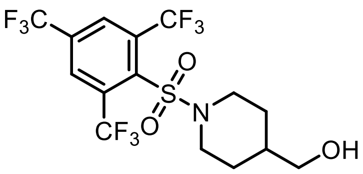

Following the general procedure using sulfonamide **2y** (30.1 mg, 0.06 mmol), sulfonamide **37** was obtained as a white solid (20.7 mg, 0.045 mmol, 75%).

^{1}H NMR (400 MHz, $CDCl_3$): δ 8.25 (s, 2H), 3.80 – 3.74 (m, 2H), 3.52 (d, *J* = 6.1 Hz, 2H), 3.00 – 2.85 (m, 2H), 1.84 – 1.68 (m, 3H), 1.55 – 1.35 (m, 2H) ppm. *The O-H was not observed in ^{1}H NMR.

^{13}C NMR (151 MHz, $CDCl_3$): δ 145.5, 134.1 (q, *J* = 35.2 Hz), 133.4 (q, *J* = 33.9 Hz), 129.3 (m), 122.2 (q, *J* = 275.6 Hz), 122.3 (q, *J* = 273.3 Hz), 67.4, 46.9, 38.3, 28.7 ppm.

^{19}F NMR (565 MHz, $CDCl_3$): δ -55.5 (6F), -63.6 (3F) ppm.

IR (neat): ν_{max} 3353, 2916, 1338, 1283, 1181, 1144, 1135, 1029, 921, 722 cm^{-1}.

HRMS (ESI$^+$): exact mass calculated for $[M+Na]^+$ ($C_{15}H_{14}F_9NO_3SNa^+$) requires *m/z* 482.0442, found *m/z* 482.0443.

With Ns

[1-((4-Nitrophenyl)sulfonyl)piperidin-4-yl]methanol (38)

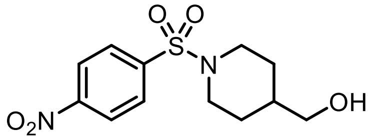

Following GP7 using sulfonamide **34** (34.2 mg, 0.10 mmol), sulfonamide **38** was obtained as a white solid (24.1 mg, 0.080 mmol, 80%).

^{1}H NMR (400 MHz, $CDCl_3$): δ 8.46 – 8.32 (m, 2H), 8.02 – 7.90 (m, 2H), 3.88 (d, *J* = 11.7 Hz, 2H), 3.64 – 3.38 (m, 2H), 2.35 (td, *J* = 11.8, 2.4 Hz, 2H), 1.83 (dd, *J* = 12.8, 2.2 Hz, 2H), 1.52 – 1.22 (m, 4H) ppm.

^{13}C NMR (151 MHz, $CDCl_3$): δ 150.3, 142.8, 128.9, 124.5, 67.0, 46.2, 37.9, 28.2 ppm.

IR (neat): ν_{max} 3336, 2918, 2851, 1530, 1343, 1162, 926, 753, 739, 596, 463 cm^{-1}.

HRMS (ESI$^+$): exact mass calculated for $[M+Na]^+$ ($C_{12}H_{16}N_2O_5SNa^+$) requires *m/z* 323.0672, found *m/z* 323.0673.

With Cs

4-[(4-(Hydroxymethyl)piperidin-1-yl)sulfonyl]benzaldehyde (39')

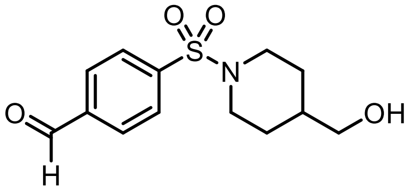

Following the general procedure using sulfonamide **35** (32.2 mg, 0.10 mmol), the undesired sulfonamide **39'** was obtained as a yellow wax (23.0 mg, 0.080 mmol, 80%) with some impurities that could not be removed by column chromatography.

^{1}H NMR (400 MHz, $CDCl_3$): δ 10.11 (s, 1H), 8.10 – 8.01 (m, 2H), 7.93 (d, *J* = 8.2 Hz, 2H), 4.10 – 4.00 (m, 2H), 3.87 (d, *J* = 11.6 Hz, 2H), 3.48 (d, *J* = 6.1 Hz, 2H), 2.81 (br s, 1H), 2.32 (t, *J* = 11.0 Hz, 2H), 2.07 – 1.95 (m, 1H), 1.85 – 1.75 (m, 2H) ppm.

^{13}C NMR (151 MHz, $CDCl_3$): δ 191.0, 142.0, 139.0, 128.4, 67.1, 46.2, 38.0, 28.2 ppm.

IR (neat): ν_{max} 3399, 2925, 2854, 1706, 1338, 1199, 931, 829, 727, 625 cm^{-1}.

HRMS (ESI$^+$): exact mass calculated for $[M+Na]^+$ ($C_{13}H_{17}NO_4SNa^+$) requires *m/z* 306.0770, found *m/z* 306.0766.

With Ts

[1-((4-Methylphenyl)sulfonyl)piperidin-4-yl]methanol (40)

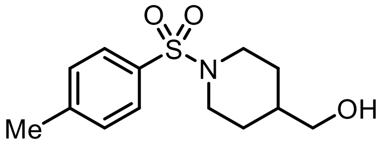

Following GP7 using sulfonamide **36** (31.1 mg, 0.10 mmol), sulfonamide **40** was obtained as a white solid (19.1 mg, 0.074 mmol, 74%).

^{1}H NMR (400 MHz, CDCl$_3$): δ 7.63 (d, *J* = 8.2 Hz, 2H), 7.31 (d, *J* = 8.0 Hz, 2H), 3.80 (d, *J* = 11.8 Hz, 2H), 3.46 (d, *J* = 6.0 Hz, 2H), 2.43 (s, 3H), 2.29 – 2.17 (m, 2H), 1.84 – 1.76 (m, 2H), 1.51 – 1.22 (m, 4H) ppm.

^{13}C NMR (151 MHz, CDCl$_3$): δ 147.6, 133.3, 129.7, 127.8, 67.2, 46.2, 38.0, 28.2, 21.6 ppm.

IR (neat): ν_{max} 3302, 2924, 2919, 1361, 1286, 1161, 959, 811, 549 cm^{-1}.

HRMS (ESI$^+$): exact mass calculated for [M+Na]$^+$ ($C_{13}H_{19}NO_3SNa^+$) requires *m/z* 292.0977, found *m/z* 292.0979.

4.4 Reaction Sequence towards 42

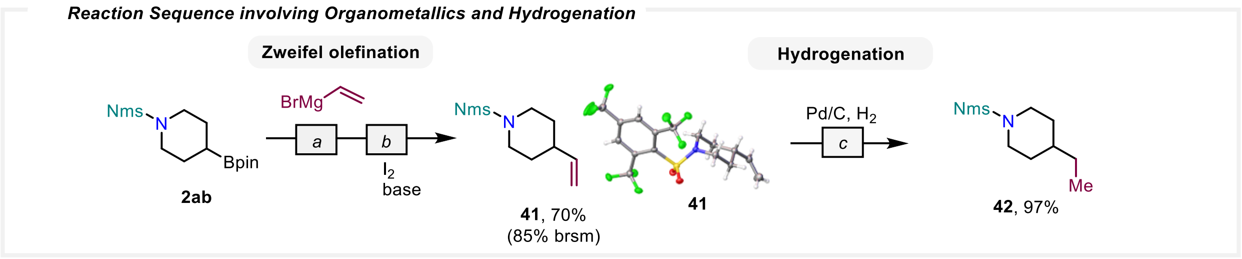

1-((2,4,6-tris(Trifluoromethyl)phenyl)sulfonyl)-4-vinylpiperidine (41)

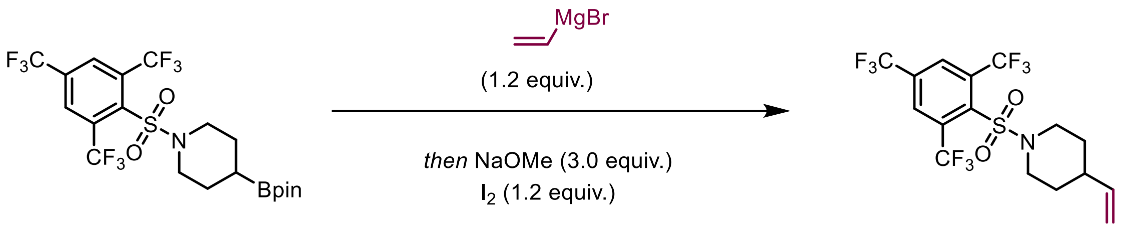

An adapted protocol from literature was used.[8]

A flame-dried Schlenk flask was loaded with sulfonamide **2ab** (55.5 mg, 0.1 mmol, 1.0 equiv.). Then, THF/DMSO (1:1, 0.5 mL each) was then added. The solution was cooled to 0 °C, then the vinylmagnesium bromide (1 M in THF, 0.12 mL, 0.12 mmol, 1.2 equiv.) was added dropwise. After addition, the ice bath was removed and the mixture was stirred for an additional 30 min at room temperature. The solution was then cooed again to 0 °C, and a suspension of NaOMe (16.2 mg

in 0.3 mL MeOH (1 M), 0.3 mmol, 3.0 equiv.) was added in one portion followed, by a dropwise addition of I_2 (30.5 mg, in 0.24 mL MeOH (0.5 M), 0.24 mL, 0.12 mmol, 1.2 equiv.). The resulting mixture was stirred at 0 °C for 30 min before adding saturated aqueous NaS_2O_3 to quench the reaction. The aqueous phase was extracted twice with DCM, the organic phases combined and dried over $MgSO_4$. After evaporation of the solvent, the crude material was subjected to column chromatography to afford the titled olefin **41** as a white solid (32.0 mg, 70 µmol, 70%). In addition, the remaining unreacted starting material **2ab** was isolated pure (8.4 mg, 12 µmol, 15%).

^{1}H NMR (400 MHz, CDCl$_3$): δ 8.26 (s, 2H), 5.76 (ddd, *J* = 17.1, 10.4, 6.4 Hz, 1H), 5.07 – 4.95 (m, 2H), 3.73 (d, *J* = 13.7 Hz, 2H), 3.04 – 2.86 (m, 2H), 2.27 – 2.15 (m, 1H), 1.79 – 1.70 (m, 2H), 1.61 – 1.50 (m, 2H) ppm.

^{13}C NMR (101 MHz, CDCl$_3$): δ 145.5, 141.6, 134.10 (q, *J* = 35.1 Hz), 133.15 (q, *J* = 34.0 Hz), 129.4, 122.23 (q, *J* = 275.7 Hz), 122.0 (d, *J* = 273.4 Hz).113.9, 46.9, 39.2, 31.4 ppm.

^{19}F NMR (376 MHz, CDCl$_3$): δ -55.48 (6F), -63.59 (3F) ppm.

IR (neat): ν_{max} 1356, 1283, 1268, 1189, 1148, 1133, 1113, 936, 863, 722, 684, 609 cm^{-1}.

HRMS (ESI$^+$): exact mass calculated for $[M+H]^+$ ($C_{16}H_{15}F_9NO_2S^+$) requires m/z 456.0674 found m/z 456.0675.

4-Ethyl-1-((2,4,6-tris(trifluoromethyl)phenyl)sulfonyl)piperidine (42)

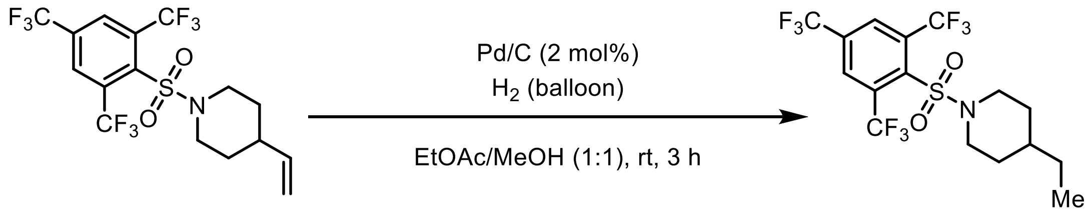

A flame-dried Schlenk flask under argon atmosphere was loaded with Pd/C (12.3 mg, 12 µmol, 0.2 equiv., 10% on carbon) and olefin **41** (26.4 mg, 58 µmol, 1.0 equiv.). Then, dry MeOH (2 mL) and EtOAc (2 mL) were added. The reaction mixture was degassed with a H_2 balloon for 30 min and then kept under a H_2 atmosphere using a H_2 balloon. The reaction was stirred for 3 h, before it was passed through a Celite pad using EtOAc. The filtrate was concentrated to give the title compound as a white solid (25.6 mg, 56 µmol, 97%).

^{1}H NMR (400 MHz, CDCl$_3$): δ 8.25 (s, 2H), 3.77 – 3.63 (m, 2H), 2.88 (app t, *J* = 12.6 Hz, 2H), 1.76 – 1.67 (m, 2H), 1.42 – 1.29 (m, 5H), 0.89 (t, *J* = 7.3 Hz, 3H) ppm.

^{13}C NMR (101 MHz, CDCl$_3$): δ 145.7, 134.0 (q, *J* = 35.1 Hz), 133.2 (q, *J* = 33.9 Hz), 129.3, 122.2 (q, *J* = 275.6 Hz), 122.1 (q, *J* = 273.4 Hz), 47.4, 37.3, 31.8, 29.1, 11.1 ppm.

^{19}F NMR (376 MHz, CDCl$_3$): δ -55.47 (6F), -63.57 (3F) ppm.

IR (neat): ν_{max} 1280, 1192, 1167, 1143, 1115, 1087, 937, 911, 723 cm^{-1}.

HRMS (ESI$^+$): exact mass calculated for $[M+H]^+$ ($C_{16}H_{17}F_9NO_2S^+$) requires *m/z* 458.0831 found *m/z* 458.0824.

5. Derivatization of Nms-amides

5.1. *N*-Alkylation

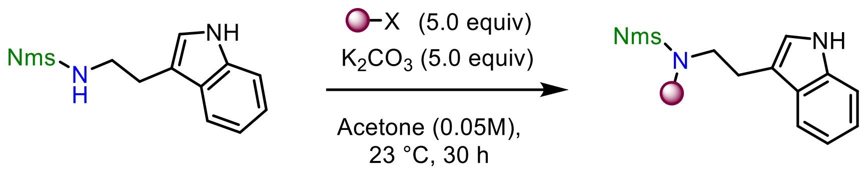

General procedure for the *N*-alkylation of Nms amide 2h:

The corresponding alkyl halide (5.0 equiv.) was added to a mixture of the sulfonamide **2h** (1.0 equiv.) and K_2CO_3 (5.0 equiv.) in dry acetone (0.1 M) at room temperature (18-25 °C). The reaction mixture was stirred at the same temperature for 48h. The progress of the reaction was monitored by TLC analysis. Then, H_2O was added, the phases were separated and the aqueous phase was extracted with EtOAc (x2). The combined organic layers were combined, dried over $MgSO_4$ and filtered. The solvents were evaporated under reduced pressure. The residue was purified by column chromatography (silica gel, heptane:EtOAc 50:1 to 10:1).

***N*-[2-(1*H*-Indol-3-yl)ethyl]-*N*-benzyl-2,4,6-tris(trifluoromethyl)benzenesulfonamide (43)**

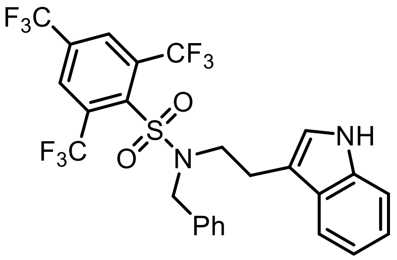

Following the general procedure using sulfonamide **2h** (50.4 mg, 0.10 mmol) and benzyl bromide (59.5 µL, 85.5 mg, 0.5 mmol), sulfonamide **43** was obtained as a yellow solid (47.8 mg, 0.080 mmol, 80%).

^{1}H NMR (400 MHz, CDCl$_3$): δ 8.04 (s, 2H), 7.83 (br s, 1H), 7.44 (d, *J* = 6.8 Hz, 2H), 7.40 – 7.30 (m, 3H), 7.24 (d, *J* = 8.3 Hz, 2H), 7.19 (d, *J* = 8.1 Hz, 1H), 7.14 – 7.10 (m, 1H), 6.99 (dd, *J* = 11.0, 4.0 Hz, 1H), 6.86 (d, *J* = 2.2 Hz, 1H), 4.70 (s, 1H), 3.53 – 3.42 (m, 2H), 2.86 – 2.77 (m, 2H) ppm.

^{13}C NMR (176 MHz, CDCl$_3$): δ 144.8, 136.0, 135.5, 133.8 (q, *J* = 34.8 Hz), 132.8 (q, *J* = 33.3 Hz), 129.1, 129.0, 128.4, 126.7, 122.5 (q, *J* = 275.8 Hz), 122.3, 121.8 (q, *J* = 273.3 Hz), 119.7, 118.5, 112.1, 111.3, 52.3, 48.0, 23.6 ppm.

^{19}F NMR (659 MHz, CDCl$_3$): δ -54.8 (6F), -63.5 (3F) ppm.

IR (neat): ν_{max} 3412, 2922, 1359, 1271, 1191, 1136, 769, 746, 541 cm^{-1}.

HRMS (ESI$^+$): exact mass calculated for [M+Na]$^+$ ($C_{26}H_{19}F_9N_2O_2SNa^+$) requires *m/z* 617.0916, found *m/z* 617.0912.

N-[2-(1H-Indol-3-yl)ethyl]-N-allyl-2,4,6-tris(trifluoromethyl)benzenesulfonamide (44)

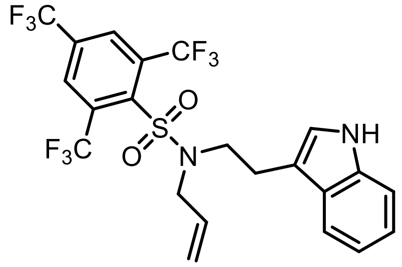

Following the general procedure using sulfonamide **2h** (50.4 mg, 0.10 mmol) and allyl bromide (43.3 µL, 60.5 mg, 0.5 mmol), sulfonamide **44** was obtained as a yellow solid (44.6 mg, 0.082 mmol, 82%).

^{1}H NMR (400 MHz, $CDCl_3$): δ 8.14 (s, 2H), 7.91 (br s, 1H), 7.47 (d, J = 7.9 Hz, 1H), 7.27 (d, J = 7.5 Hz, 1H), 7.21 – 7.12 (m, 1H), 7.08 – 7.04 (m, 1H), 6.92 (d, J = 2.2 Hz, 1H), 5.92 (ddt, J = 16.9, 10.1, 6.7 Hz, 1H), 5.65 – 5.24 (m, 2H), 4.02 (d, J = 6.7 Hz, 2H), 3.54 (dd, J = 9.0, 6.6 Hz, 2H), 3.06 (dd, J = 8.9, 6.6 Hz, 2H) ppm.

^{13}C NMR (176 MHz, $CDCl_3$): δ 144.9, 136.2, 135.5, 134.0 (q, J = 35.9 Hz), 133.1 (q, J = 33.6 Hz), 133.0, 129.2 (m), 126.9, 122.4, 122.3, 122.1 (q, J = 275.8 Hz), 122.0 (q, J = 273.9 Hz), 120.4, 119.7, 118.7, 112.3, 111.3, 51.2, 48.4, 24.0 ppm.

^{19}F NMR (659 MHz, $CDCl_3$): δ -54.9 (6F), -63.5 (3F) ppm.

IR (neat): ν_{max} 3423, 2917, 2849, 1357. 1283, 1137, 909, 745 cm^{-1}.

HRMS (ESI$^+$): exact mass calculated for $[M+Na]^+$ ($C_{22}H_{17}F_9N_2O_2SNa^+$) requires m/z 567.0759, found m/z 567.0760.

N-[2-(1H-Indol-3-yl)ethyl]-N-(cyanomethyl)-2,4,6-tris(trifluoromethyl)benzenesulfonamide (45)

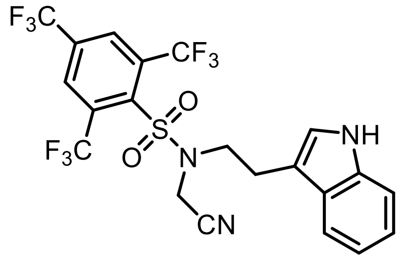

Following an adapted version of the general procedure (1.5 equiv. of iodoacetonitrile, 1.5 equiv. of K_2CO_3) using sulfonamide **2h** (50.4 mg, 0.10 mmol) and iodoacetonitrile (43.3 µL, 60.5 mg, 0.5 mmol), sulfonamide **45** was obtained as a yellow solid (39.9 mg, 0.073 mmol, 73%).

^{1}H NMR (400 MHz, $CDCl_3$): δ 8.14 (s, 2H), 7.95 (br s, 1H), 7.47 (d, J = 7.8 Hz, 1H), 7.27 (m, 1H), 7.16 (t, J = 7.3, 1H), 7.07 (t, J = 7.5 Hz, 1H), 7.01 (d, J = 2.2 Hz, 1H), 4.32 (s, 2H), 3.72 – 3.67 (m, 2H), 3.20 – 3.15 (m, 2H) ppm.

^{13}C NMR (176 MHz, CDCl$_3$): δ 142.8, 136.2, 134.9 (q, *J* = 35.7 Hz), 133.1 (q, *J* = 33.7 Hz), 129.4 (m), 126.6, 122.7, 122.6, 121.9 (q, *J* = 275.6 Hz), 121.8 (q, *J* = 273.4 Hz), 120.2, 118.4, 114.3, 111.5, 111.1, 49.6, 35.6, 23.6 ppm.

^{19}F NMR (659 MHz, CDCl$_3$): δ -55.0 (6F), -63.5 (3F) ppm.

IR (neat): ν_{max} 3409, 2360, 1361, 1285, 1191, 1179, 1144, 758 cm^{-1}.

HRMS (ESI$^+$): exact mass calculated for [M+Na]$^+$ ($C_{21}H_{14}F_9N_3O_2SNa^+$) requires *m/z* 566.0555, found *m/z* 566.0549.

***N*-[2-(1*H*-Indol-3-yl)ethyl]-*N*-propyl-2,4,6-tris(trifluoromethyl)benzenesulfonamide (46)**

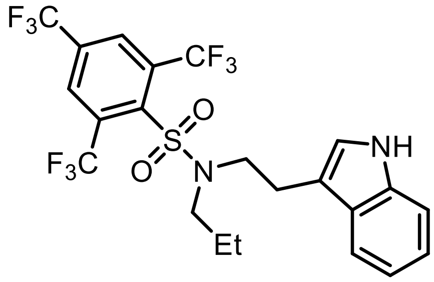

Following an adapted version of the general procedure (60 °C) using sulfonamide **2h** (50.4 mg, 0.10 mmol) and iodopropane (48.8 µL, 85.0 mg, 0.5 mmol), sulfonamide **46** was obtained as a yellow solid (53.6 mg, 0.0981 mmol, 98%).

^{1}H NMR (400 MHz, CDCl$_3$): δ 8.11 (s, 2H), 7.91 (br s, 1H), 7.44 (d, *J* = 7.9 Hz, 1H), 7.25 (d, *J* = 6.6 Hz, 1H), 7.15 (t, *J* = 7.6, 1H), 7.05 (t, *J* = 7.5 Hz, 1H), 6.95 (d, *J* = 2.2 Hz, 1H), 3.55 – 3.51 (m, 2H), 3.46 – 3.36 (m, 2H), 3.11 – 2.97 (m, 2H), 1.80 – 1.70 (m, 2H), 0.97 (t, *J* = 7.4 Hz, 3H) ppm.

^{13}C NMR (151 MHz, CDCl$_3$): 145.1, 136.2, 133.8 (q, *J* = 35.0 Hz), 133.0 (q, *J* = 33.6 Hz), 129.1 (m), 126.8, 122.4, 122.3, 122.1 (q, *J* = 275.8 Hz), 122.0 (q, *J* = 273.4 Hz), 119.7, 118.5, 112.2, 111.3, 50.1, 47.9, 24.3, 21.5, 11.4 ppm.

^{19}F NMR (376 MHz, CDCl$_3$): δ -54.9 (6F), -63.5 (3F) ppm.

IR (neat): ν_{max} 3414, 1372, 1190, 1171, 911, 750 cm^{-1}.

HRMS (ESI$^+$): exact mass calculated for [M+Na]$^+$ ($C_{22}H_{19}F_9N_2O_2SNa^+$) requires *m/z* 569.0916, found *m/z* 569.0896.

5.2. *N*-Arylation

_N_-(_p_-Tolyl)-2,4,6-tris(trifluoromethyl)benzenesulfonamide (47)

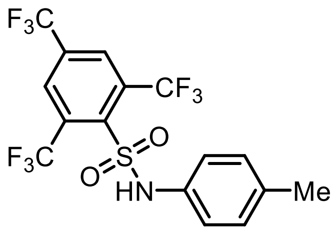

Et_3N (2.0 equiv.) was added to a mixture of sulfonamide **6** (0.10 mmol, 1.0 equiv.), arylboronic acid (2.0 equiv.), $Cu(OAc)_2$ (1.0 equiv.) and 3Å MS (11 mg/0.1 mmol **6**) in dry DCM (0.06 M) at room temperature (18-25 °C). The reaction mixture was stirred at the same temperature for 48 h. The progress of the reaction was monitored by TLC analysis. The solvents were evaporated under reduced pressure. The residue was purified by column chromatography (silica gel, heptane:EtOAc 50:1 to 20:1). Sulfonamide **47** was obtained as a yellow solid (33.2 mg, 0.074 mmol, 74%).

^{1}H NMR (400 MHz, $CDCl_3$): δ 8.31 (s, 2H), 7.09 (d, *J* = 8.2 Hz, 2H), 6.96 (d, *J* = 8.3 Hz, 2H), 6.87 (br s, 1H), 2.31 (s, 3H) ppm.

^{13}C NMR (151 MHz, $CDCl_3$): δ 144.9, 137.5, 134.5 (q, *J* = 35.2 Hz), 133.2 (q, *J* = 33.7 Hz), 131.7, 130.1, 129.6 (m), 124.6, 122.1 (q, *J* = 275.9 Hz), 122.0 (q, *J* = 273.6 Hz), 21.1 ppm.

^{19}F NMR (565 MHz, $CDCl_3$): δ -54.6 (6F), -63.5 (3F) ppm.

IR (neat): ν_{max} 3362, 1509, 1365, 1273, 1187, 1157, 1081, 919, 708, 685, 644, 612, 491 cm^{-1}.

HRMS (ESI$^+$): exact mass calculated for $[M+Na]^+$ ($C_{16}H_{10}F_9NO_2SNa^+$) requires *m/z* 474.0181, found *m/z* 474.0171.

_N_-(3,4-Dimethoxyphenethyl)-_N_-phenyl-2,4,6-tris(trifluoromethyl)benzenesulfonamide (48)

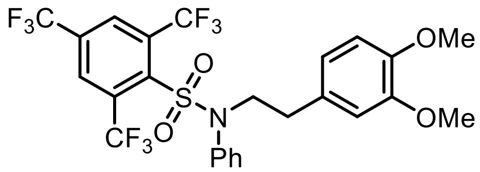

A mixture of sulfonamide **2e** (0.10 mmol, 1.0 equiv.), CuCl (20 mol%), diaryliodonium salt (2.0 equiv.) and K_3PO_4 (2.0 equiv.) in dry DCM (0.1 M) at room temperature (18-25 °C). The reaction mixture was stirred at 40 °C for 48h. The progress of the reaction was monitored by TLC analysis. The solvents were evaporated under reduced pressure. The residue was purified by column chromatography (silica gel, heptane:EtOAc 50:1 to 5:1). Sulfonamide **48** was obtained as a white solid (47.7 mg, 0.079 mmol, 79%).

^{1}H NMR (400 MHz, CDCl$_3$): δ 8.29 (s, 2H), 7.39 – 7.31 (m, 3H), 7.21 – 7.16 (m, 2H), 6.77 (d, *J* = 7.9 Hz, 1H), 6.65 (d, *J* = 8.1 Hz, 2H), 4.02 – 3.89 (m, 2H), 3.85 (s, 6H), 2.79 (m, 2H) ppm.

^{13}C NMR (151 MHz, CDCl$_3$): δ 149.1, 148.0, 144.0, 137.2, 134.5 (q, *J* = 35.2 Hz), 133.9 (q, *J* = 33.6 Hz), 130.2, 130.1, 129.7, 129.6 (m), 129.1, 122.0 (q, *J* = 273.6 Hz), 121.8 (q, *J* = 276.2 Hz), 112.2, 111.5, 56.0, 54.8, 34.9 ppm.

^{19}F NMR (565 MHz, CDCl$_3$): δ -54.8 (6F), -63.7 (3F) ppm.

IR (neat): ν_{max} 3050, 1518, 1377, 1287, 1195, 1137, 916, 902, 727, 684 cm^{-1}.

HRMS (ESI$^+$): exact mass calculated for [M+Na]$^+$ ($C_{25}H_{20}F_9NO_4SNa^+$) requires *m/z* 624.0862, found *m/z* 624.0863.

N-(2-(1-Phenyl-1*H*-indol-3-yl)ethyl)-2,4,6-tris(trifluoromethyl)benzenesulfonamide (49)

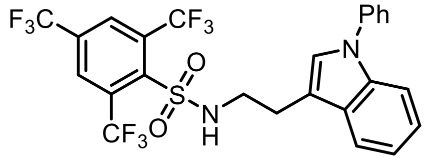

Procedure adapted from the literature:[9]

A mixture of XPhos (0.04 mmol, 40 mol%), $Pd_2(dba)_3$ (0.02 mmol, 20 mol%), sulfonamide **2h** (0.1 mmol, 1.0 equiv.), bromobenzene (0.5 mmol, 5.0 equiv.), LiO*t*Bu (0.2 mmol, 2.0 equiv.) and dry THF (0.1 M) was stirred at 80 °C for 12 h. Then, the mixture was cooled down and was filtered through a short plug of silica gel. The solvents were evaporated under reduced pressure and the residue was purified by column chromatography (silica gel, heptane:EtOAc 20:1 to 5:1). Sulfonamide **49** was obtained as a yellow solid (33.5 mg, 0.0577 mmol, 58%). 35% of sulfonamide **2h** (17.5 mg, 0.0347 mmol) was recovered after purification of the crude.

^{1}H NMR (600 MHz, CDCl$_3$): δ 8.22 (s, 2H), 7.55 – 7.50 (m, 4H), 7.48 – 7.46 (m, 2H), 7.39 – 7.35 (m, 1H), 7.24 – 7.21 (m, 2H), 7.12 – 7.10 (m, 1H), 5.08 (t, *J* = 5.4 Hz, 1H), 3.60 (app q, *J* = 6.5 Hz, 2H), 3.12 (t, *J* = 6.7 Hz, 2H) ppm.

^{13}C NMR (151 MHz, CDCl$_3$): 145.2, 139.5, 136.5, 133.9 (q, *J* = 35.0 Hz), 132.4 (q, *J* = 33.5 Hz), 129.8, 129.4 (m), 128.2, 126.7, 126.6, 124.3, 123.0, 121.8 (q, *J* = 275.8 Hz), 121.5, 120.4, 118.8, 112.3, 111.0, 43.9, 26.2 ppm.

^{19}F NMR (376 MHz, CDCl$_3$): δ -54.7 (6F), -63.5 (3F) ppm.

IR (neat): ν_{max} 2958, 2920, 2851, 1287, 1192, 915, 740, 574, 458 cm^{-1}.

HRMS (ESI$^+$): exact mass calculated for [M+Na]$^+$ ($C_{25}H_{17}F_9N_2O_2S^+$) requires *m/z* 603.0759, found *m/z* 603.0749.

5.3. Mitsunobu reaction

General Procedure for the intermolecular Mitsunobu reaction:

The corresponding sulfonamide **2** (1.0 equiv.) and PPh_3 (2.0 equiv.) were dissolved in dry THF (0.1 M) in a flame-dried Schlenk flask under an atmosphere of argon. The corresponding alcohol (1.0 equiv.) was then added. The mixture was cooled to 0 °C and DEAD (2.0 equiv.) was added dropwise. After stirring the reaction for 10 min at 0 °C the cooling bath was removed, and the reaction was continued for 48 h. The solvent was removed under reduced pressure and the crude was purified by column chromatography (silica gel, heptane/EtOAc, 50:1 to 9:1).

Benzyl (*S*)-1-((2,4,6-tris(trifluoromethyl)phenyl)sulfonyl)aziridine-2-carboxylate (50)

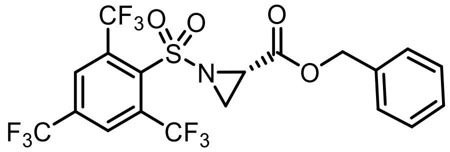

Following the general procedure using sulfonamide **2w** (140.0 mg, 0.26 mmol), sulfonamide **50** was obtained as a white solid (101.0 mg, 0.194 mmol, 75%).

^{1}H NMR (400 MHz, $CDCl_3$): δ 8.34 (s, 2H), 7.38 – 7.33 (m, *J* = 2.4 Hz, 5H), 5.22 (s, 2H), 3.82 (dd, *J* = 7.0, 4.5 Hz, 1H), 3.23 (d, *J* = 7.0 Hz, 1H), 2.84 (d, *J* = 4.5 Hz, 1H) ppm.

^{13}C NMR (101 MHz, $CDCl_3$): δ 166.1, 142.2, 135.5 (q, *J* = 35.3 Hz), 134.8, 133.9 (q, *J* = 34.4 Hz), 129.7 – 129.4 (m), 128.9, 128.8, 128.6, 121.9 (q, *J* = 273.6 Hz), 121.9 (q, *J* = 276.0 Hz), 68.1, 38.1, 35.3 ppm.

^{19}F NMR (377 MHz, $CDCl_3$): δ -54.59 (6F), -63.68 (3F) ppm.

IR (neat): ν_{max} 1745, 1374, 1270, 1176, 1137, 1087, 913, 864, 717, 684 cm^{-1}.

HRMS (ESI$^+$): exact mass calculated for $[M+Na]^+$ ($C_{19}H_{12}F_9NO_4SNa^+$) requires m/z 544.0236, m/z found: 544.0236.

Determination of enantiopurity. Method description: Chiralpak IC 250 x 4.6 mm, particle size 5 µm, solvent system: *n*-heptane+0.1*i*-PrOH/*i*-PrOH 98:2; flow 1 mL/min, 25 °C. Peak area 99.017% (Rt = 6.571 min), 0.983% (Rt = 7.870 min): 98.0% *ee*.

Enantiopure compound:

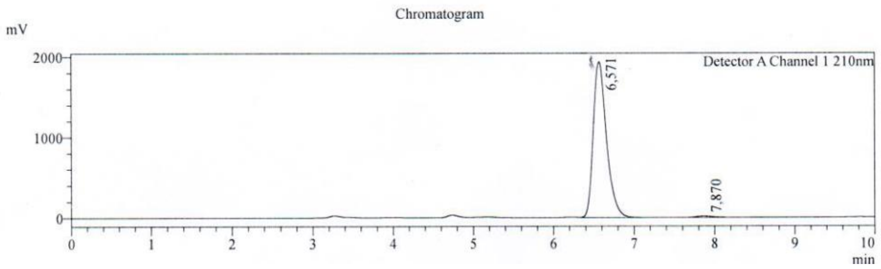

Racemic standard:

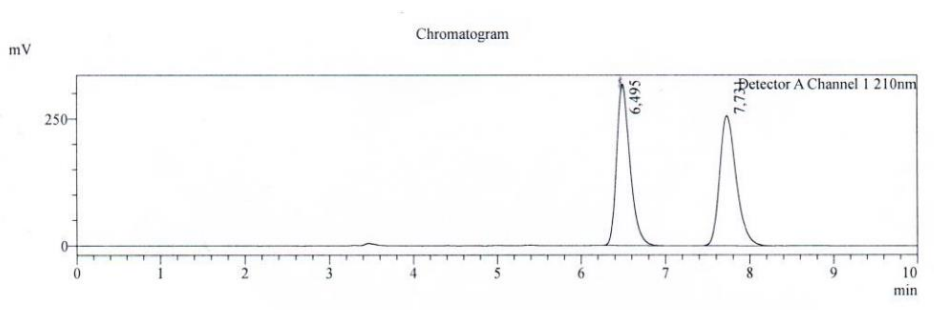

Methyl *N*-(4-methoxybenzyl)-*N*-((2,4,6-tris(trifluoromethyl)phenyl)sulfonyl)-*D*-alaninate (51)

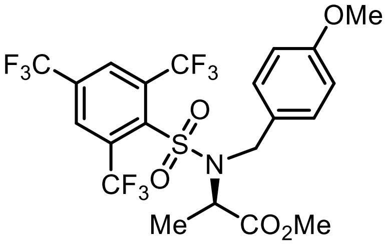

Following the general procedure using sulfonamide **2n** (120.0 mg, 0.25 mmol) and methyl (*S*)-(-)-lactate (28.6 µL, 31.2 mg, 0.3 mmol), sulfonamide **51** was obtained as a yellow solid (86.6 mg, 0.153 mmol, 61%).

^{1}H NMR (400 MHz, $CDCl_3$): δ 7.95 (s, 2H), 7.07 (d, *J* = 8.6 Hz, 2H), 6.51 (d, *J* = 8.7 Hz, 2H), 4.96 (q, *J* = 7.3 Hz, 1H), 4.72 (d, *J* = 14.5 Hz, 1H), 4.25 (d, *J* = 14.5 Hz, 1H), 3.75 (s, 3H), 3.67 (s, 3H), 1.59 (d, *J* = 7.3 Hz, 3H) ppm.

^{13}C NMR (151 MHz, $CDCl_3$): δ 191.0, 171.7, 159.2, 146.0, 133.9 (q, *J* = 35.1 Hz), 132.6 (q, *J* = 33.8 Hz), 131.7, 129.0 (m), 125.7, 122.0 (q, *J* = 275.7 Hz), 121.9 (q, *J* = 273.4 Hz), 56.9, 55.1, 52.7, 49.3, 16.4 ppm.

^{19}F NMR (565 MHz, $CDCl_3$): δ -54.5 (6F), -63.8 (3F) ppm.

IR (neat): ν_{max} 1747, 1714, 1515, 1361, 1193, 1149, 862 cm^{-1}.

HRMS (ESI$^+$): exact mass calculated for $[M+Na]^+$ ($C_{21}H_{18}F_9NO_5SNa^+$) requires *m/z* 590.0654, found *m/z* 590.0656.

Determination of enantiopurity. Method description: Chiralcel IC 250 x 4.6 mm, particle size 5 µm, solvent system: *n*-heptane+0.1*i*-PrOH/MTBE/*i*-PrOH 85:10:5; flow 1 mL/min, 25 °C. Peak area 99.311% (Rt = 5.227 min), 0.689% (Rt = 7.746 min): 98.6% *ee*.

Enantiopure compound:

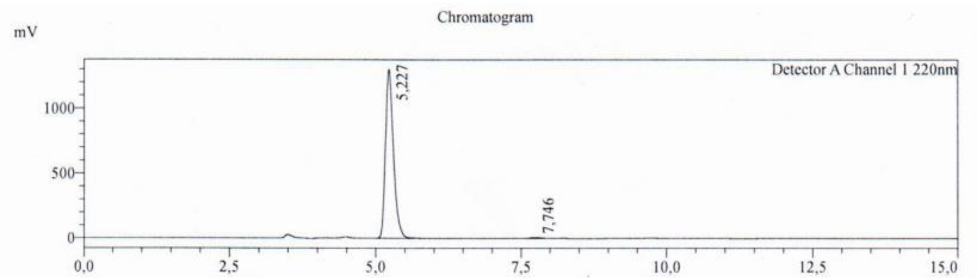

Racemic standard:

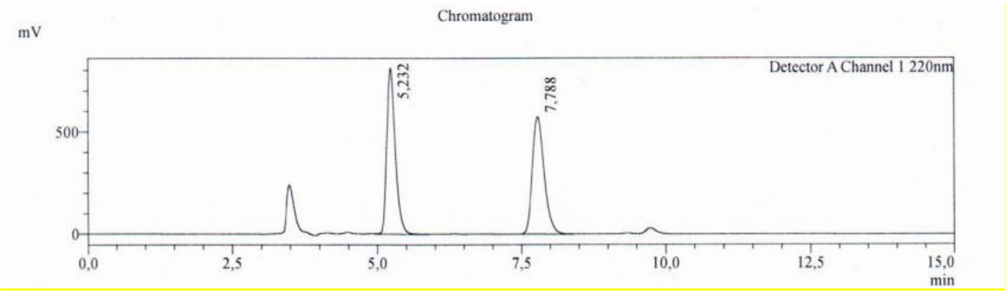

5.4 Synthesis of Cinacalcet (53)

(*R*)-*N*-(1-(naphthalen-1-yl)ethyl)-2,4,6-tris(trifluoromethyl)-*N*-(3-(3-(trifluoromethyl)phenyl)propyl)benzenesulfonamide (52)

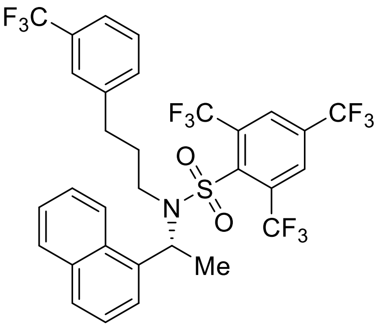

Following an adapted version of the general procedure (1.4 equiv. of the alcohol, 96 h reaction time) using sulfonamide **2o** (1.55 g, 3.0 mmol) and 3-(3'-trifluoromethylphenyl)propanol (0.84 g, 4.1 mmol), sulfonamide **52** was obtained as a yellow solid (1.90 g, 2.7 mmol, 89%).

^{1}H NMR (600 MHz, $CDCl_3$): δ 8.12 (s, 2H), 8.01 (d, *J* = 8.3 Hz, 1H), 7.81 (d, *J* = 7.9 Hz, 1H), 7.73 (d, *J* = 8.1 Hz, 1H), 7.52 – 7.43 (m, 3H), 7.41 (d, *J* = 7.7 Hz, 1H), 7.32 (t, *J* = 7.8 Hz, 1H), 7.31 (t, *J* = 7.8 Hz, 1H), 7.11 (s, 1H), 7.07 (d, *J* = 7.6 Hz, 1H), 5.84 (q, *J* = 6.9 Hz, 1H), 3.62 – 3.48 (m, 2H), 2.54 – 2.41 (m, 2H), 1.69 – 1.62 (m, 1H), 1.59 (d, *J* = 6.9 Hz, 3H), 1.54 – 1.44 (m, 1H) ppm.

^{13}C NMR (151 MHz, $CDCl_3$): δ 146.2, 141.9, 135.1, 134.0 (q, *J* = 35.0 Hz), 133.8, 133.2 (q, *J* = 33.2 Hz), 131.7, 131.0, 130.7 (q, *J* = 32.0 Hz), 129.3, 129.1, 128.9, 126.9, 126.1, 125.4, 125.04, 124.98 (q, *J* = 3.7 Hz), 124.8, 124.3 (q, *J* = 272.3 Hz), 123.0 (q, J = 3.9 Hz), 122.8, 122.1 (q, *J* = 276.2 Hz), 121.9 (q, *J* = 274.1 Hz), 53.1, 47.5, 33.1, 32.4, 19.8 ppm.

^{19}F NMR (565 MHz, $CDCl_3$): δ -54.5 (6F), -62.5 (3F), -63.6 (3F) ppm.

IR (neat): ν_{max} 1365, 1328, 1270, 1197, 1159, 1113, 1074, 703 cm^{-1}.

HRMS (ESI$^+$): exact mass calculated for $[M+Na]^+$ $(C_{31}H_{23}F_{12}NO_2SNa)^+$ requires *m/z* 724.1150, found *m/z* 724.1151.

Determination of enantiopurity. Method description: Lux-Cellulose 1 (Chiralcel OD-H) 250 x 4.6 mm, particle size 5 µm, solvent system: *n*-heptane+0.1*i*-PrOH/*i*-PrOH 98:2; flow 1 mL/min, 25 °C. Peak area 0.089% (Rt = 8.732 min), 99.911% (Rt = 10.046 min): 99.8% *ee*.

Enantiopure compound:

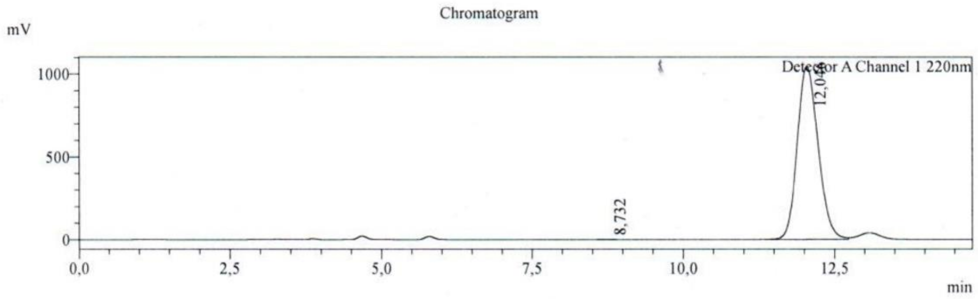

Racemic standard:

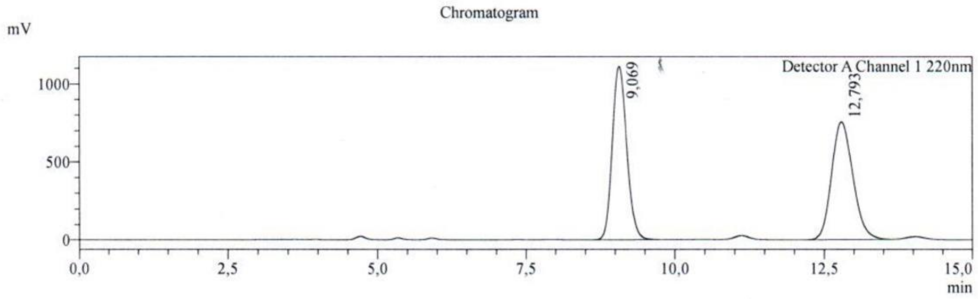

(*R*)-*N*-(1-(Naphthalen-1-yl)ethyl)-3-(3-(trifluoromethyl)phenyl)propan-1-amine (Cinalcacet) (53) – CAS number: 226256-56-0

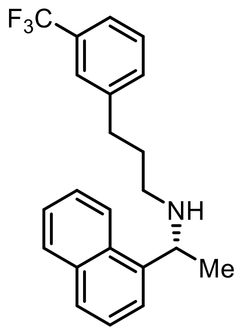

An adapted version of GP5 (0.1 M in THF) using sulfonamide **52** (1.878 g, 2.7 mmol) was followed. After 40 h additional thiophenol (0.050 ml, 0.49 mmol, 0.2 equiv.) was added. The reaction was stirred at room temperature for 4 h, followed by heating to 30 °C for 3 h. The solvent was removed under reduced pressure and the residue was purified by column chromatography (silica gel, DCM then DMA (DCM/MeOH/NH_3 (aq. 25%) = 10:1:0.1)). Cinacalcet **53** was obtained as a yellowish oil (892 mg, 2.5 mmol, 93%). The spectroscopic data matched the data reported in the literature for this compound.[10]

^{1}H NMR (700 MHz, $CDCl_3$): δ 8.20 (d, *J* = 8.4 Hz, 1H), 7.89 (d, *J* = 8.0 Hz, 1H), 7.76 (d, *J* = 8.1 Hz, 1H), 7.66 (d, *J* = 7.1 Hz, 1H), 7.52 (ddd, *J* = 8.4, 6.8, 1.4 Hz, 1H), 7.51 – 7.48 (m, 2H), 7.44 – 7.42 (m, 2H), 7.35 (t, *J* = 7.6 Hz, 1H), 7.31 (d, *J* = 7.6 Hz, 1H), 4.64 (q, *J* = 6.5 Hz, 1H), 2.74 (dt, *J* = 15.2, 7.7 Hz, 1H), 2.70 – 2.64 (m, 2H), 2.63 – 2.58 (m, 1H), 1.89 – 1.82 (m, 2H), 1.51 (d, *J* = 6.6 Hz, 3H) ppm.

^{13}C NMR (176 MHz, $CDCl_3$): δ 143.2, 141.2, 134.1, 131.9, 131.4, 130.7 (q, *J* = 31.9 Hz), 129.1, 128.8, 127.4, 125.9, 125.8, 125.5, 125.2 (q, *J* = 3.7 Hz), 124.0 (q, *J* = 272.3 Hz), 123.0, 122.81, 122.77 (q, *J* = 3.8 Hz), 53.9, 47.4, 33.6, 32.0, 23.7 ppm.

^{19}F NMR (659 MHz, $CDCl_3$): δ -62.5 (3F) ppm.

IR (neat): ν_{max} 3063, 2970, 2929, 2861, 1329, 1161, 1121, 779 cm^{-1}.

HRMS (ESI$^+$): exact mass calculated for $[M+H]^+$ $(C_{22}H_{23}F_3N)^+$ requires *m/z* 358.1777, found *m/z* 358.1773.

Determination of enantiopurity. Method description: Lux-3 Cellulose-3 250 x 4.6 mm, particle size 3 µm, solvent system: *n*-heptane/*i*-PrOH/DEA 98:2:0.1; flow 0.7 mL/min, 25 °C. Peak area 99.840% (Rt = 15.747 min), 0.160% (Rt = 18.201 min): 99.7% *ee*.

Enantiopure compound:

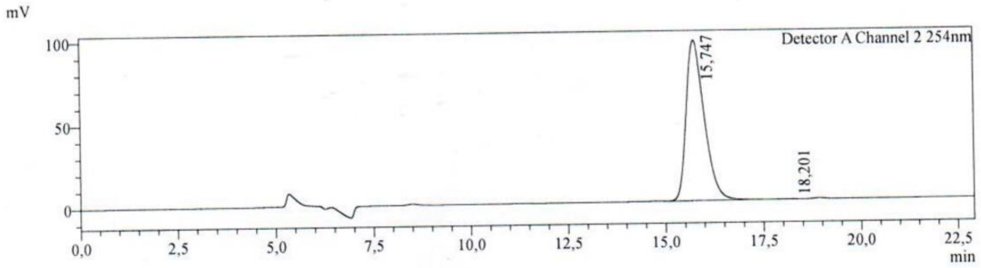

Racemic standard:

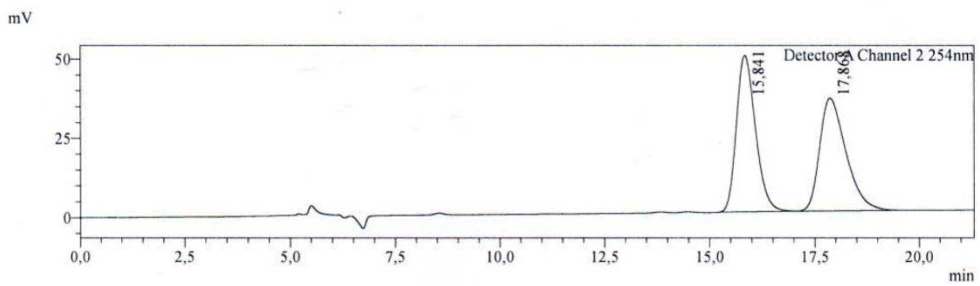

6. DFT calculations

The conformational space of all molecules has been initially searched using meta-dynamics simulations based on semiempirical tight-binding quantum chemical calculations as implemented in CREST.[11,12]

Structures located with CREST have then been subjected to PBE0-D3BJ/def2-SVP[13–19] single point calculation. Structures within a window of 6 kcal mol^{-1} to the structure with the lowest energy have been subjected to geometry optimization at the same level of theory. The nature of all stationary points (minima and transition states) was verified through the computation of the vibrational frequencies. The thermal corrections to the Gibbs free energies were combined with the single point energies calculated at the PBE0-D3BJ/def2-TZVP level of theory to yield Gibbs free energies ("G_{298}") at 298.15 K. All energies are reported in kcal mol^{-1}. The energy profiles were constructed using the most stable conformation (the global minimum) of each intermediate and transition state.

The DFT calculations have been performed with the Gaussian 16 program package.[20] The polarizable continuum model (PCM) with SMD parameters[21,22] of acetonitrile was applied to consider solvent effects for both geometries and energies. The same method was applied for the simulation of the IR spectra at the B3LYP-D3BJ/Def2-TZVP,SMD[23] level of theory.

Free energies in solution have been corrected to a reference state of 1 mol l^{-1} at 298.15 K through the addition of RTln(24.46) = +7.925 kJ mol^{-1} to the gas phase (1 atm) free energies.

6.1. XYZ structures

Cartesian coordinates of the most stable (ΔG_{298}) conformations, computed at the PBE0-D3BJ/def2-TZVP-SMD//PBE0-D3BJ/def2-SVP-SMD level of theory.

```
41
SM_2,4,6-CF3
C        -3.397689000      2.372073000      0.405909000
H        -2.992186000      3.342944000      0.086303000
H        -4.095881000      2.537605000      1.243383000
N        -2.298546000      1.510975000      0.786803000
H        -1.711704000      1.861227000      1.598043000
S        -2.365268000     -0.088620000      0.638459000
O        -2.172449000     -0.698601000      1.951755000
O        -3.511406000     -0.463161000     -0.183454000
H        -3.953858000      1.932351000     -0.430136000
C        -0.839208000     -0.556333000     -0.261097000
C        -0.253438000     -1.810806000      0.009557000
C        -0.182301000      0.307646000     -1.156626000
C         1.019680000     -2.104709000     -0.471804000
C         1.100900000      0.001975000     -1.611223000
C         1.716463000     -1.178805000     -1.234499000
H         1.618347000      0.698830000     -2.269792000
S        -0.572321000      2.962500000      2.955652000
C         0.846737000      2.261999000      2.213308000
C         1.111396000      0.871705000      2.271207000
C         1.797056000      3.054369000      1.526715000
C         2.258072000      0.320865000      1.703099000
H         0.385957000      0.223648000      2.770344000
C         2.939587000      2.499238000      0.953165000
H         1.615825000      4.130454000      1.450903000
C         3.187878000      1.126760000      1.039639000
H         2.426023000     -0.757749000      1.773445000
H         3.648155000      3.150070000      0.430977000
H         4.082385000      0.690472000      0.588844000
C        -0.782839000      1.563363000     -1.764425000
F        -2.092712000      1.439370000     -1.973139000
F        -0.567830000      2.666575000     -1.049306000
F        -0.233807000      1.790210000     -2.966685000
C         3.123234000     -1.491378000     -1.663441000
F         3.160515000     -2.513837000     -2.524746000
F         3.880660000     -1.840380000     -0.616248000
F         3.711749000     -0.450750000     -2.252572000
H         1.474947000     -3.067329000     -0.235254000
C        -0.911866000     -2.945931000      0.780293000
F        -0.659040000     -2.906874000      2.086943000
F        -2.228279000     -3.001222000      0.597388000
F        -0.428559000     -4.120747000      0.344108000
```

```
41
TS1_2,4,6-CF3
C         4.244015000     -0.762068000     -1.075814000
H         5.166361000     -0.459904000     -1.592793000
H         3.565540000     -1.249257000     -1.793698000
N         3.668370000      0.418491000     -0.461178000
H         3.454292000      1.200815000     -1.077227000
S         2.628801000      0.320249000      0.803075000
O         2.995012000     -0.877523000      1.553640000
O         2.670999000      1.603978000      1.499825000
H         4.511631000     -1.477691000     -0.288625000
C         0.847964000      0.142055000      0.266207000
C         0.177413000     -1.037775000      0.747999000
C         0.090185000      1.365992000      0.365566000
C        -1.140094000     -0.986335000      1.187868000
C        -1.220214000      1.375107000      0.801111000
C        -1.850708000      0.207198000      1.244678000
H        -1.771531000      2.318833000      0.802968000
S         1.130482000     -0.113454000     -2.113229000
C        -0.571159000     -0.447181000     -2.308869000
C        -1.063135000     -1.765749000     -2.239753000
C        -1.503273000      0.597615000     -2.475650000
C        -2.430518000     -2.025512000     -2.306486000
H        -0.353240000     -2.587142000     -2.127280000
C        -2.869208000      0.334692000     -2.541632000
H        -1.137592000      1.623876000     -2.539472000
C        -3.341808000     -0.976697000     -2.446243000
H        -2.788260000     -3.057010000     -2.240208000
H        -3.574075000      1.162956000     -2.657224000
H        -4.415019000     -1.180291000     -2.487897000
C         0.598965000      2.704829000     -0.107936000
F         0.748489000      3.580470000      0.896447000
F         1.753781000      2.665471000     -0.774803000
F        -0.289172000      3.282419000     -0.946692000
C        -3.270346000      0.252669000      1.684459000
F        -4.124480000      0.471591000      0.662682000
F        -3.500232000      1.245254000      2.561970000
F        -3.662596000     -0.886255000      2.267669000
H        -1.625481000     -1.912444000      1.500045000
C         0.767264000     -2.425901000      0.720153000
F         1.781596000     -2.576688000     -0.127778000
F         1.176566000     -2.852167000      1.926263000
F        -0.167260000     -3.328942000      0.342687000
```

```
41
MC_2,4,6-CF3
C       -4.239430000     -1.002683000      0.891748000
N       -3.201834000     -1.912397000      0.455863000
S       -1.633801000     -1.773393000      1.007510000
O       -0.994233000     -3.066290000      0.731195000
O       -1.735080000     -1.336937000      2.408433000
C       -0.513364000     -0.390668000      0.023337000
C        0.113741000      0.443670000      1.064430000
C        1.463547000      0.372464000      1.339912000
C        2.340378000     -0.453240000      0.626600000
C        1.812741000     -1.189787000     -0.444156000
C        0.475220000     -1.156029000     -0.760759000
C        0.044395000     -1.884792000     -1.990115000
F       -1.225932000     -2.319662000     -1.951034000
F        0.808607000     -2.960495000     -2.237179000
F        0.129532000     -1.123283000     -3.107494000
H        2.480725000     -1.795625000     -1.061106000
C        3.793047000     -0.446432000      0.898511000
F        4.368541000     -1.637935000      0.652661000
F        4.486322000      0.442348000      0.143872000
F        4.076867000     -0.127822000      2.173190000
H        1.860996000      0.996983000      2.144629000
C       -0.646635000      1.434523000      1.889757000
F       -0.377087000      1.310498000      3.204139000
F       -0.297521000      2.713542000      1.593419000
F       -1.972608000      1.382726000      1.762111000
S       -1.802669000      0.468205000     -1.041720000
C       -0.986413000      1.997481000     -1.397150000
C       -1.724670000      3.180515000     -1.266956000
C       -1.135409000      4.408001000     -1.565516000
C        0.199438000      4.467617000     -1.967517000
C        0.936865000      3.288753000     -2.090798000
C        0.347097000      2.054609000     -1.824465000
H        0.920154000      1.134230000     -1.947276000
H        1.981550000      3.326131000     -2.410782000
H        0.665278000      5.432033000     -2.185258000
H       -1.720436000      5.325886000     -1.464087000
H       -2.758163000      3.136190000     -0.914392000
H       -3.228264000     -2.200898000     -0.521143000
H       -4.246483000     -0.043185000      0.347647000
H       -4.103227000     -0.800649000      1.962040000
H       -5.214978000     -1.494533000      0.759406000
```

```
41
TS2_2,4,6-CF3
C       -1.900403000     -3.286533000      0.541890000
H       -2.231930000     -3.634594000     -0.445302000
H       -2.337106000     -2.287409000      0.720710000
N       -0.456543000     -3.336935000      0.605062000
H       -0.052879000     -3.202588000      1.529954000
S        0.466424000     -2.568325000     -0.576815000
O        1.809960000     -3.176685000     -0.483346000
O       -0.313546000     -2.763792000     -1.817811000
H       -2.301513000     -3.975966000      1.299822000
C        0.434043000     -0.422320000     -0.127902000
C       -0.367333000      0.134110000     -1.214778000
C       -0.179565000     -0.291881000      1.196174000
C       -1.687180000      0.481496000     -1.023707000
C       -1.501212000      0.071639000      1.341045000
C       -2.290404000      0.427932000      0.240026000
H       -1.934051000      0.101919000      2.342672000
S        2.240239000     -0.157978000     -0.208735000
C        2.422486000      1.585681000      0.049114000
C        1.387888000      2.484041000      0.339712000
C        3.738638000      2.065417000     -0.046996000
C        1.674390000      3.836361000      0.526820000
H        0.357660000      2.138780000      0.421668000
C        4.012500000      3.416977000      0.144740000
H        4.554622000      1.373440000     -0.275767000
C        2.981283000      4.312924000      0.432346000
H        0.855180000      4.524837000      0.751452000
H        5.043930000      3.770715000      0.065524000
H        3.195680000      5.374024000      0.581019000
C        0.609700000     -0.584092000      2.429170000
F        1.351265000     -1.707218000      2.322257000
F        1.476067000      0.390505000      2.766169000
F       -0.177356000     -0.757970000      3.500891000
C       -3.730956000      0.724842000      0.419121000
F       -4.501631000     -0.388786000      0.469061000
F       -4.232158000      1.468650000     -0.578362000
F       -3.983929000      1.380052000      1.566082000
H       -2.268634000      0.833035000     -1.877639000
C        0.219958000      0.342583000     -2.575699000
F       -0.731503000      0.412279000     -3.521842000
F        0.907655000      1.504546000     -2.670957000
F        1.071242000     -0.612350000     -2.961769000
```

```
41
P_2,4,6-CF3
C        0.252131000      4.341714000      0.096142000
H        0.910492000      4.696850000     -0.714038000
H        0.202598000      5.148019000      0.848640000
N        0.803380000      3.144423000      0.691241000
H        0.861923000      2.397274000     -0.005013000
S       -0.208517000      2.448943000      1.971706000
O       -1.612722000      2.483436000      1.401780000
O        0.365220000      1.044833000      1.973606000
H       -0.766844000      4.212806000     -0.316820000
C        0.161220000     -1.080229000     -0.811987000
C       -0.152305000     -1.909356000      0.278138000
C       -0.688316000     -0.001148000     -1.104914000
C       -1.279995000     -1.655172000      1.053950000
C       -1.799996000      0.260633000     -0.308381000
C       -2.097482000     -0.574267000      0.758708000
H       -2.433181000      1.117631000     -0.516354000
S        1.565714000     -1.443922000     -1.850539000
C        2.860305000     -0.487130000     -1.097979000
C        2.689413000      0.266398000      0.066286000
C        4.101849000     -0.515780000     -1.747160000
C        3.767827000      0.998439000      0.564228000
H        1.731328000      0.322427000      0.600381000
C        5.170184000      0.213167000     -1.228776000
H        4.234592000     -1.103434000     -2.660177000
C        5.008688000      0.977503000     -0.071626000
H        3.613338000      1.602544000      1.462317000
H        6.135462000      0.185482000     -1.741201000
H        5.845932000      1.554666000      0.328574000
C       -0.426364000      0.922585000     -2.271920000
F        0.775089000      1.512794000     -2.201372000
F       -0.482599000      0.284851000     -3.444755000
F       -1.326978000      1.908851000     -2.326851000
C       -3.288768000     -0.306336000      1.633783000
F       -4.030731000     -1.419415000      1.779149000
F       -4.091009000      0.631981000      1.134748000
F       -2.937589000      0.070182000      2.865495000
H       -1.514477000     -2.307157000      1.896115000
C        0.708769000     -3.095861000      0.644305000
F        0.245447000     -3.716189000      1.734586000
F        0.749826000     -4.012376000     -0.327746000
F        1.970138000     -2.742737000      0.913395000
```

```
34
SM_4-NO2
C       -2.804546000     -1.714626000     -1.592683000
N       -2.373129000     -2.004706000     -0.239276000
S       -0.941604000     -2.762039000     -0.012863000
O       -0.903299000     -3.230493000      1.368587000
O       -0.749045000     -3.704780000     -1.114307000
C        0.297829000     -1.488022000     -0.162832000
C        0.779522000     -1.142582000     -1.427430000
C        1.642704000     -0.059916000     -1.549159000
C        2.009502000      0.631548000     -0.397868000
C        1.562214000      0.271157000      0.869741000
C        0.687052000     -0.801887000      0.986613000
H        0.287638000     -1.093694000      1.957593000
H        1.888248000      0.834303000      1.743018000
N        2.888774000      1.794852000     -0.525613000
O        3.281584000      2.322591000      0.492913000
O        3.176935000      2.166836000     -1.643558000
H        2.026173000      0.254428000     -2.519624000
H        0.480765000     -1.716108000     -2.306414000
H       -2.483523000     -1.200879000      0.438966000
H       -3.775508000     -1.203964000     -1.524064000
H       -2.941440000     -2.645282000     -2.159504000
H       -2.114131000     -1.057275000     -2.151512000
S       -2.572417000      0.544770000      1.702148000
C       -1.593680000      1.667070000      0.794257000
C       -0.740928000      2.592502000      1.444915000
C        0.058659000      3.483920000      0.733240000
C        0.049232000      3.494252000     -0.664325000
C       -0.790883000      2.597779000     -1.330489000
C       -1.597752000      1.710708000     -0.620946000
H       -2.252751000      1.026114000     -1.164933000
H       -0.817819000      2.585999000     -2.424739000
H        0.687953000      4.182661000     -1.223164000
H        0.709910000      4.173131000      1.279350000
H       -0.710009000      2.587532000      2.538389000
```

```
34
TS1_4-NO2
C       -3.281358000     -1.951326000      1.455339000
H       -3.521360000     -1.280717000      2.291044000
H       -2.264932000     -2.353709000      1.601176000
N       -3.446112000     -1.229670000      0.209302000
H       -3.264471000     -1.771313000     -0.635632000
S       -2.868571000      0.311076000      0.048025000
O       -3.305973000      0.753249000     -1.273771000
O       -3.279505000      1.045014000      1.243795000
H       -3.998443000     -2.785259000      1.472292000
C       -1.054147000      0.177910000      0.040783000
C       -0.490010000     -0.056995000      1.355753000
C       -0.552754000     -0.668673000     -1.027577000
C        0.634336000     -0.821829000      1.512554000
H       -0.940098000      0.455105000      2.208056000
C        0.571365000     -1.427675000     -0.851969000
H       -1.045941000     -0.620694000     -2.001157000
C        1.208134000     -1.498147000      0.411285000
H        1.095267000     -0.937218000      2.494716000
H        0.986659000     -2.005808000     -1.678925000
S       -0.659767000      2.225746000     -0.516597000
C        1.077581000      2.074413000     -0.349314000
C        1.705715000      2.286635000      0.890902000
C        1.864879000      1.674603000     -1.444649000
C        3.077459000      2.085880000      1.034480000
H        1.101990000      2.608262000      1.742452000
C        3.235329000      1.473309000     -1.297167000
H        1.383136000      1.516306000     -2.412273000
C        3.844272000      1.669049000     -0.055241000
H        3.550490000      2.249040000      2.006756000
H        3.832392000      1.154635000     -2.156007000
H        4.918848000      1.504876000      0.060798000
N        2.382114000     -2.251336000      0.573671000
O        2.859806000     -2.833441000     -0.403725000
O        2.907552000     -2.309861000      1.688430000
```

```
34
MC_4-NO2
C       -2.167111000      2.875132000      1.568638000
N       -1.204686000      3.280116000      0.563179000
S        0.348600000      2.723501000      0.602522000
O        1.050945000      3.435423000     -0.464691000
O        0.809646000      2.838833000      1.986256000
C        0.280959000      0.937942000      0.143549000
C       -0.472568000      0.746582000     -1.104251000
C       -1.364158000     -0.274663000     -1.249767000
C       -1.673267000     -1.142274000     -0.168366000
C       -1.079071000     -0.894615000      1.095905000
C       -0.184507000      0.120231000      1.270237000
H        0.260273000      0.310948000      2.248698000
H       -1.367580000     -1.527240000      1.937181000
N       -2.575011000     -2.194319000     -0.336910000
O       -2.838939000     -2.924541000      0.626456000
```

```
O       -3.089803000     -2.376312000     -1.447352000
H       -1.867287000     -0.437570000     -2.204489000
H       -0.240694000      1.406645000     -1.943837000
S        2.229783000      0.635312000     -0.200601000
C        2.136809000     -1.116626000     -0.371824000
C        1.896966000     -1.687309000     -1.631460000
C        1.774599000     -3.069263000     -1.765811000
C        1.893852000     -3.895577000     -0.646716000
C        2.146502000     -3.336471000      0.607022000
C        2.266864000     -1.954532000      0.746103000
H        2.462379000     -1.511683000      1.725078000
H        2.246575000     -3.980537000      1.484716000
H        1.795384000     -4.979069000     -0.753313000
H        1.582630000     -3.503687000     -2.750507000
H        1.803401000     -1.035302000     -2.502830000
H       -1.555816000      3.308792000     -0.393907000
H       -2.971655000      3.624693000      1.605675000
H       -2.612294000      1.885013000      1.377457000
H       -1.673189000      2.859923000      2.549106000
```

```
34
TS2_4-NO2
C       -1.387749000     -3.530405000      1.963067000
H       -2.324521000     -3.088796000      2.330056000
H       -0.539709000     -3.018366000      2.453856000
N       -1.371312000     -3.456664000      0.515800000
H       -0.524611000     -3.839681000      0.093293000
S       -1.703079000     -1.948397000     -0.200181000
O       -1.698862000     -2.234330000     -1.650125000
O       -2.969621000     -1.508552000      0.422484000
H       -1.370047000     -4.588810000      2.264330000
C       -0.246274000     -0.338248000      0.143718000
C       -0.166608000      0.312936000     -1.148635000
C       -0.802694000      0.450927000      1.223030000
C       -0.746021000      1.534313000     -1.364216000
H        0.342230000     -0.208689000     -1.960973000
C       -1.374334000      1.672874000      0.990381000
H       -0.776887000      0.041186000      2.235309000
C       -1.380170000      2.228986000     -0.309231000
H       -0.704733000      2.002855000     -2.348812000
H       -1.811804000      2.247519000      1.808235000
S        1.145522000     -1.445938000      0.550726000
C        2.558982000     -0.392503000      0.353078000
C        3.739748000     -0.983696000     -0.116615000
C        2.551067000      0.969640000      0.683351000
C        4.900788000     -0.222654000     -0.243765000
H        3.744598000     -2.042133000     -0.390592000
C        3.711508000      1.726489000      0.530147000
H        1.639202000      1.436404000      1.060421000
C        4.890524000      1.136310000      0.072221000
H        5.816470000     -0.695979000     -0.607879000
H        3.692986000      2.790072000      0.782557000
H        5.798226000      1.734493000     -0.039448000
N       -1.964111000      3.490042000     -0.539673000
O       -2.492131000      4.083990000      0.401908000
O       -1.935104000      3.962738000     -1.677150000
```

```
34
P_4-NO2
C       -3.642225000     -0.863807000     -1.507652000
H       -3.112110000     -1.799695000     -1.755980000
H       -3.268455000     -0.084807000     -2.199762000
N       -3.445333000     -0.556109000     -0.106940000
H       -3.870280000      0.345994000      0.131238000
S       -1.743819000     -0.337327000      0.310847000
O       -1.226874000      0.631656000     -0.732334000
O       -1.930704000      0.337427000      1.656918000
H       -4.711935000     -1.031665000     -1.715651000
C        1.879454000     -0.292347000      2.050660000
C        1.387129000     -1.536296000      1.649076000
C        2.147642000      0.703522000      1.096893000
C        1.111613000     -1.774827000      0.305635000
H        1.174185000     -2.306716000      2.393165000
C        1.867487000      0.473636000     -0.238931000
H        2.548470000      1.669283000      1.411151000
C        1.341046000     -0.760288000     -0.615412000
H        0.688853000     -2.722564000     -0.024980000
H        2.030203000      1.242267000     -0.993695000
S        2.170205000     -0.008188000      3.774462000
C        1.412839000      1.586223000      4.004397000
C        2.056534000      2.507790000      4.836925000
C        0.173869000      1.895976000      3.425562000
C        1.460194000      3.742696000      5.095648000
H        3.027649000      2.263242000      5.275232000
C       -0.396362000      3.142467000      3.676461000
H       -0.361657000      1.186156000      2.778744000
C        0.236259000      4.066718000      4.511506000
H        1.966895000      4.459300000      5.747341000
H       -1.358041000      3.384525000      3.215390000
H       -0.224522000      5.038697000      4.705320000
N        0.955309000     -0.967722000     -2.001307000
O        1.259386000     -0.118203000     -2.813522000
O        0.360855000     -1.990466000     -2.279754000
```

```
36
SM_2,4-NO2
C       -3.430093000     -1.205688000      1.971659000
H       -3.380065000     -2.209107000      1.531234000
H       -4.469453000     -1.016475000      2.282795000
N       -3.046808000     -0.221967000      0.984237000
H       -2.847151000      0.764480000      1.313534000
S       -2.243619000     -0.575887000     -0.374552000
O       -2.286177000     -2.015508000     -0.619057000
O       -2.693783000      0.338311000     -1.418938000
H       -2.782018000     -1.183609000      2.860881000
C       -0.525036000     -0.103872000     -0.104570000
C        0.452901000     -0.941360000      0.440318000
C       -0.168255000      1.188544000     -0.483459000
C        1.761900000     -0.518844000      0.611095000
C        1.124900000      1.656124000     -0.271516000
H       -0.922928000      1.833756000     -0.930094000
C        2.064246000      0.793412000      0.276869000
H        2.518605000     -1.177373000      1.035758000
H        1.404913000      2.675792000     -0.532847000
S       -2.132526000      2.688758000      1.908155000
C       -0.570130000      2.285012000      2.567106000
C       -0.276341000      1.012477000      3.113639000
C        0.488978000      3.225673000      2.565030000
C        0.987515000      0.703012000      3.609220000
H       -1.058803000      0.250029000      3.125483000
C        1.750712000      2.914857000      3.067457000
H        0.299323000      4.217780000      2.145045000
C        2.018169000      1.647338000      3.591863000
H        1.172622000     -0.300648000      4.004929000
H        2.540988000      3.671133000      3.036817000
H        3.011287000      1.397900000      3.973524000
N        3.418554000      1.284156000      0.539434000
O        3.666994000      2.432026000      0.243482000
O        4.210624000      0.513751000      1.037493000
N        0.151219000     -2.287116000      0.930226000
O        0.848961000     -3.190978000      0.531136000
O       -0.737977000     -2.391302000      1.742079000
```

```
36
TS1_2,4-NO2
C       -3.747140000     -1.602711000      1.135639000
H       -3.396872000     -2.575155000      0.770591000
H       -4.843657000     -1.637818000      1.224292000
N       -3.411475000     -0.580681000      0.168486000
H       -3.561345000      0.377430000      0.488207000
S       -2.099297000     -0.620579000     -0.800843000
O       -1.825899000     -2.010559000     -1.153677000
O       -2.385920000      0.351369000     -1.853698000
H       -3.301733000     -1.399270000      2.119595000
C       -0.576568000      0.030721000     -0.044373000
C        0.393474000     -0.772079000      0.580295000
C       -0.186525000      1.304950000     -0.477942000
C        1.700884000     -0.333753000      0.762025000
C        1.089954000      1.785643000     -0.244983000
H       -0.911838000      1.917533000     -1.011441000
C        2.034212000      0.942627000      0.351841000
H        2.435548000     -0.979333000      1.239252000
H        1.377385000      2.788505000     -0.558509000
S       -2.073316000      1.471400000      2.272998000
C       -0.442658000      1.615548000      2.833309000
C        0.203954000      0.554305000      3.516926000
C        0.321730000      2.787868000      2.609390000
C        1.529569000      0.653012000      3.924297000
H       -0.362399000     -0.362081000      3.702736000
C        1.647677000      2.883689000      3.023731000
H       -0.154669000      3.627120000      2.095784000
C        2.269643000      1.812794000      3.669578000
H        1.999944000     -0.193112000      4.434628000
H        2.209827000      3.800098000      2.821741000
H        3.316206000      1.881840000      3.976866000
N        3.387869000      1.419757000      0.569118000
O        3.633030000      2.572860000      0.272649000
O        4.207019000      0.640899000      1.017028000
N        0.082881000     -2.078695000      1.131893000
O        0.975416000     -2.901003000      1.184024000
O       -1.042235000     -2.259086000      1.542776000
```

```
36
MC_2,4-NO2
C       -4.341269000     -1.173541000      0.668197000
N       -3.619799000      0.039631000      0.345672000
S       -2.335145000      0.561876000      1.226608000
O       -2.288053000      2.021135000      1.185985000
O       -2.447463000     -0.102664000      2.529410000
C       -0.701991000     -0.013647000      0.443039000
C       -0.094040000      1.014634000     -0.432030000
C        1.219569000      1.436225000     -0.301513000
C        2.007579000      0.985419000      0.742828000
C        1.456911000      0.077510000      1.701962000
C        0.187263000     -0.370526000      1.578106000
H       -0.227294000     -1.067232000      2.307195000
H        2.080135000     -0.249411000      2.535042000
N        3.348965000      1.423289000      0.856695000
O        3.797064000      2.200926000      0.019516000
O        4.019384000      1.003827000      1.795904000
H        1.621580000      2.134543000     -1.033850000
N       -0.835610000      1.510797000     -1.525696000
O       -2.016650000      1.176012000     -1.624287000
O       -0.301834000      2.250925000     -2.344362000
S       -1.183333000     -1.598393000     -0.503143000
C        0.456179000     -2.165240000     -0.865903000
C        1.150912000     -1.637232000     -1.962626000
C        2.450745000     -2.061794000     -2.233618000
C        3.056853000     -3.023931000     -1.424233000
C        2.360660000     -3.564543000     -0.342315000
C        1.064730000     -3.135129000     -0.059365000
H        0.518199000     -3.545832000      0.792519000
H        2.831229000     -4.321058000      0.290927000
H        4.075354000     -3.356729000     -1.640317000
H        2.991427000     -1.640202000     -3.084875000
H        0.671188000     -0.889302000     -2.598028000
H       -3.513633000      0.296906000     -0.634802000
H       -5.335327000     -1.111351000      0.202024000
H       -3.842475000     -2.092435000      0.317132000
H       -4.477190000     -1.238404000      1.754681000
```

```
36
TS2_2,4-NO2
C        1.355315000      3.622389000      0.133258000
H        1.461695000      3.163405000     -0.867349000
H        2.349556000      3.663023000      0.599495000
N        0.441394000      2.925286000      1.015184000
H       -0.524581000      2.981438000      0.685836000
S        0.786353000      1.266420000      1.320320000
O        2.259685000      1.240780000      1.532295000
O       -0.066799000      0.953017000      2.502174000
H        0.993978000      4.654353000      0.005810000
```

```
C       0.227393000     -0.297144000     -0.404106000
C       1.561758000     -0.551948000     -0.900852000
C      -0.316316000     -1.356958000      0.412276000
C       2.333408000     -1.607598000     -0.433051000
C       0.437213000     -2.407141000      0.848047000
H      -1.358017000     -1.294790000      0.722049000
C       1.788783000     -2.527088000      0.442122000
H       3.362556000     -1.701986000     -0.774987000
H       0.003257000     -3.171010000      1.493673000
S      -0.910063000      0.705796000     -1.372986000
C      -2.501897000      0.310073000     -0.672485000
C      -3.445036000     -0.310312000     -1.498421000
C      -2.862854000      0.712406000      0.621154000
C      -4.744210000     -0.524864000     -1.036325000
H      -3.157856000     -0.625831000     -2.504554000
C      -4.156452000      0.476785000      1.081514000
H      -2.119928000      1.165139000      1.283484000
C      -5.100927000     -0.133986000      0.253462000
H      -5.477272000     -1.007344000     -1.687875000
H      -4.430413000      0.779419000      2.095542000
H      -6.116955000     -0.307264000      0.617173000
N       2.588931000     -3.617628000      0.911995000
O       2.073558000     -4.418480000      1.677540000
O       3.747001000     -3.700825000      0.532321000
N       2.200307000      0.369875000     -1.775286000
O       3.357238000      0.183035000     -2.119589000
O       1.541587000      1.335529000     -2.154025000
```

```
36
P_2,4-NO2
C      -3.482640000     -0.231011000      1.395004000
H      -2.788492000     -0.444169000      2.224195000
H      -3.662691000     -1.185608000      0.865754000
N      -2.934590000      0.810347000      0.552294000
H      -3.576692000      1.035661000     -0.214204000
S      -1.446187000      0.354554000     -0.325500000
O      -1.637653000     -1.115772000     -0.643555000
O      -1.633783000      1.231930000     -1.537823000
H      -4.433630000      0.107299000      1.839163000
C       0.966112000     -0.627847000      1.501833000
C       0.885924000     -1.695196000      0.580941000
C       1.839432000      0.431079000      1.175786000
C       1.580685000     -1.680615000     -0.620837000
C       2.554358000      0.448566000     -0.004072000
H       1.946940000      1.265177000      1.868829000
C       2.405264000     -0.609523000     -0.904633000
H       1.460225000     -2.504821000     -1.320713000
H       3.222977000      1.276497000     -0.239949000
S       0.040955000     -0.605859000      2.997477000
C       0.218793000      1.095571000      3.501114000
C       1.119197000      1.431854000      4.515823000
C      -0.619120000      2.067197000      2.939726000
C       1.194577000      2.751926000      4.960689000
H       1.762486000      0.662315000      4.949562000
C      -0.531446000      3.383848000      3.388215000
H      -1.330328000      1.793089000      2.152631000
C       0.372741000      3.727174000      4.395533000
H       1.899613000      3.017758000      5.752282000
H      -1.180372000      4.146924000      2.950629000
H       0.433900000      4.761079000      4.744848000
N       3.133920000     -0.593093000     -2.163931000
O       3.848291000      0.362360000     -2.390157000
O       2.987938000     -1.531994000     -2.919775000
N       0.048195000     -2.854565000      0.823120000
O      -0.051095000     -3.693161000     -0.043928000
O      -0.482435000     -2.940382000      1.914996000
```

```
34
SM_2-NO2
C      -1.001077000      1.348745000     -2.350865000
N      -1.514252000     -0.006071000     -2.456590000
S      -0.641470000     -1.227979000     -1.794512000
O      -1.331243000     -2.477207000     -2.084751000
O       0.741195000     -1.036701000     -2.216677000
C      -0.649232000     -1.019563000     -0.008413000
C       0.530072000     -0.573881000      0.581077000
C       0.636945000     -0.459883000      1.969446000
C      -0.434917000     -0.825940000      2.778354000
C      -1.616363000     -1.294139000      2.207077000
C      -1.722657000     -1.371238000      0.823889000
N      -3.014719000     -1.802993000      0.294721000
O      -3.447604000     -1.233147000     -0.685950000
O      -3.598147000     -2.677975000      0.890631000
H      -2.468122000     -1.585757000      2.823389000
H      -0.358609000     -0.748512000      3.865229000
H       1.582105000     -0.099274000      2.403121000
H       1.378916000     -0.327050000     -0.059501000
H      -2.497484000     -0.116798000     -2.204661000
H      -1.003917000      1.736949000     -1.317139000
H       0.022790000      1.383131000     -2.743152000
H      -1.632365000      1.999085000     -2.971542000
S       3.908601000      0.905755000      2.647805000
C       3.308597000      1.618458000      1.181900000
C       2.252912000      2.567412000      1.181855000
C       1.752267000      3.116576000      0.003757000
C       2.281858000      2.753435000     -1.238842000
C       3.328231000      1.825226000     -1.270058000
C       3.828841000      1.273880000     -0.093217000
H       4.642512000      0.543614000     -0.135965000
H       3.759325000      1.522415000     -2.229816000
H       1.889057000      3.185521000     -2.162624000
H       0.933036000      3.841172000      0.055817000
H       1.825641000      2.862241000      2.144919000
```

```
34
TS1_2-NO2
C      -3.184764000      1.924340000      1.386155000
N      -2.870439000      0.546729000      1.061295000
S      -2.410436000      0.140746000     -0.468339000
O      -2.924938000     -1.190984000     -0.783512000
O      -2.833477000      1.261764000     -1.316774000
```

```
C      -0.574271000     -0.007713000     -0.641753000
C       0.035943000     -1.282759000     -0.393549000
C       1.098795000     -1.767530000     -1.183335000
C       1.542930000     -1.066126000     -2.277911000
C       0.903711000      0.158345000     -2.600209000
C      -0.132997000      0.644123000     -1.844996000
H      -0.638897000      1.568653000     -2.124646000
H       1.223643000      0.721593000     -3.481448000
H       1.551428000     -2.713116000     -0.883601000
N      -0.329606000     -2.038322000      0.743589000
O      -1.169947000     -1.570801000      1.512317000
O       0.198859000     -3.126287000      0.947710000
S       0.060545000      1.480155000      1.047522000
C       1.741668000      1.062343000      0.841561000
C       2.572048000      1.779217000     -0.041664000
C       3.886158000      1.376009000     -0.269192000
C       4.399911000      0.249215000      0.377332000
C       3.595427000     -0.457695000      1.274703000
C       2.282422000     -0.056227000      1.506727000
H       1.649879000     -0.611956000      2.203324000
H       3.992682000     -1.335543000      1.792085000
H       5.428566000     -0.070475000      0.191005000
H       4.512978000      1.940533000     -0.965304000
H       2.165393000      2.654285000     -0.554338000
H      -2.294817000      0.032142000      1.728978000
H      -3.533341000      1.942058000      2.428725000
H      -2.331007000      2.616181000      1.292063000
H      -4.001967000      2.283073000      0.747450000
H       2.366225000     -1.443754000     -2.887099000
```

```
34
MC_2-NO2
C      -3.695668000      0.081132000      1.514274000
N      -3.134930000     -0.370708000      0.259351000
S      -2.247264000      0.634652000     -0.704311000
O      -2.476713000      0.281489000     -2.104145000
O      -2.586940000      1.997650000     -0.270565000
C      -0.380113000      0.388528000     -0.409937000
C       0.239239000     -0.567714000     -1.338629000
C       1.403601000     -0.258529000     -2.079426000
C       1.961781000      0.991831000     -2.055369000
C       1.332181000      1.993550000     -1.252571000
C       0.228767000      1.730794000     -0.502448000
H      -0.234619000      2.510509000      0.103567000
H       1.743846000      3.007535000     -1.236928000
H       1.828118000     -1.067865000     -2.675222000
N      -0.264698000     -1.861442000     -1.413829000
O      -1.308278000     -2.130165000     -0.796467000
O       0.316858000     -2.724641000     -2.079281000
S      -0.338433000     -0.197770000      1.452611000
C       1.416600000     -0.156890000      1.655758000
C       2.196008000     -1.237167000      1.216503000
C       3.582779000     -1.199803000      1.349903000
C       4.202089000     -0.093298000      1.933798000
C       3.430224000      0.978094000      2.385238000
C       2.043274000      0.950203000      2.243659000
H       1.433753000      1.789790000      2.585274000
H       3.910797000      1.845720000      2.844982000
H       5.289735000     -0.067662000      2.040206000
H       4.183374000     -2.042308000      0.997047000
H       1.707904000     -2.102344000      0.762224000
H      -2.739524000     -1.311135000      0.213576000
H      -4.194221000      1.047375000      1.366846000
H      -4.454653000     -0.650736000      1.828045000
H      -2.954272000      0.187206000      2.325056000
H       2.856260000      1.222827000     -2.636576000
```

```
34
TS2_2-NO2
C      -3.590867000     -1.862592000      0.550820000
N      -3.448388000     -0.423761000      0.602128000
S      -1.975808000      0.226209000      1.219441000
O      -2.333119000      1.623803000      1.576799000
O      -1.632027000     -0.681713000      2.351672000
C      -0.155076000      0.385788000     -0.206024000
C      -0.228166000      1.775440000     -0.573707000
C       0.644918000      2.744035000     -0.048400000
C       1.643129000      2.385013000      0.827619000
C       1.763921000      1.019489000      1.191799000
C       0.931087000      0.057198000      0.679747000
H       1.057842000     -0.983353000      0.978073000
H       2.550109000      0.715576000      1.889059000
H       0.489645000      3.779073000     -0.356855000
N      -1.321139000      2.200561000     -1.348066000
O      -2.178503000      1.352730000     -1.626820000
O      -1.412097000      3.368000000     -1.714175000
S      -0.667927000     -0.863741000     -1.438048000
C       0.535565000     -2.145269000     -1.156354000
C       1.804870000     -2.065496000     -1.742648000
C       2.729308000     -3.090320000     -1.547917000
C       2.388662000     -4.204107000     -0.777893000
C       1.121880000     -4.290072000     -0.198946000
C       0.196215000     -3.263222000     -0.385539000
H      -0.790707000     -3.314199000      0.080552000
H       0.852413000     -5.159936000      0.405689000
H       3.113551000     -5.008731000     -0.629891000
H       3.720074000     -3.021390000     -2.004632000
H       2.063178000     -1.193964000     -2.349247000
H      -3.582187000      0.021398000     -0.307321000
H      -4.650872000     -2.114662000      0.383114000
H      -2.989896000     -2.351816000     -0.237308000
H      -3.295130000     -2.281848000      1.522286000
H       2.325959000      3.132634000      1.235584000
```

```
34
P_2-NO2
C       2.627250000      2.585289000     -0.122326000
N       1.445471000      2.086291000      0.544754000
S       1.779586000      0.752187000      1.683739000
O       2.678899000      1.453663000      2.667178000
O       0.378029000      0.539520000      2.219362000
```

```
C       -0.156676000     -1.127209000     -0.741522000
C        1.215882000     -1.429419000     -0.839641000
C        1.774617000     -2.549528000     -0.219045000
C        0.969090000     -3.417331000      0.496153000
C       -0.400788000     -3.150091000      0.590121000
C       -0.950351000     -2.028668000     -0.013840000
H       -2.020340000     -1.839199000      0.079868000
H       -1.055034000     -3.829569000      1.141792000
H        2.847745000     -2.713155000     -0.317329000
N        2.118672000     -0.570902000     -1.574240000
O        1.628542000      0.222931000     -2.359520000
O        3.312558000     -0.674452000     -1.378654000
S       -0.867588000      0.308037000     -1.491711000
C       -2.381529000      0.494716000     -0.569232000
C       -3.596159000      0.407168000     -1.254781000
C       -4.790128000      0.659200000     -0.576986000
C       -4.766646000      0.987480000      0.777850000
C       -3.547487000      1.068868000      1.456332000
C       -2.346036000      0.834149000      0.790334000
H       -1.380466000      0.883856000      1.318391000
H       -3.531681000      1.327173000      2.518821000
H       -5.702248000      1.181428000      1.308941000
H       -5.741050000      0.592324000     -1.111660000
H       -3.607207000      0.140157000     -2.314579000
H        0.775074000      1.715841000     -0.135595000
H        2.380221000      3.471574000     -0.731762000
H        3.354633000      2.899774000      0.642391000
H        3.138843000      1.860117000     -0.790013000
H        1.399892000     -4.298989000      0.974446000
```

```
35
SM_4-CF3
C        3.098666000      1.837056000     -1.330528000
N        3.111733000      1.258884000     -0.002104000
S        3.080901000     -0.367460000      0.149803000
O        3.360406000     -0.679562000      1.547982000
O        3.914254000     -0.934702000     -0.910607000
C        1.398843000     -0.867082000     -0.168783000
C        0.994980000     -1.131265000     -1.478339000
C       -0.340175000     -1.431084000     -1.725710000
C       -1.248293000     -1.475227000     -0.664685000
C       -0.831311000     -1.229465000      0.643519000
C        0.500954000     -0.916820000      0.895269000
H        0.841726000     -0.691767000      1.906526000
H       -1.546530000     -1.267730000      1.466394000
C       -2.682844000     -1.811786000     -0.955411000
F       -3.488486000     -1.548819000      0.076344000
F       -2.835872000     -3.111854000     -1.249121000
F       -3.148603000     -1.127433000     -2.008624000
H       -0.673832000     -1.628082000     -2.746887000
H        1.721182000     -1.106917000     -2.292712000
H        2.456906000      1.722945000      0.687265000
H        2.210032000      1.563880000     -1.928617000
H        3.101818000      2.929879000     -1.211811000
H        3.999884000      1.545369000     -1.886913000
S        1.027816000      2.696453000      1.954031000
C       -0.445049000      2.375171000      1.076175000
C       -1.663028000      2.122511000      1.754063000
C       -2.845424000      1.852953000      1.068317000
C       -2.870937000      1.818657000     -0.328818000
C       -1.683578000      2.070097000     -1.021904000
C       -0.501667000      2.349373000     -0.338750000
H        0.411031000      2.551819000     -0.904157000
H       -1.675634000      2.046271000     -2.116249000
H       -3.794708000      1.591932000     -0.866322000
H       -3.760929000      1.656957000      1.635481000
H       -1.658618000      2.133521000      2.847969000
```

```
35
MC_4-CF3
C        2.018012000      2.128993000      1.312381000
H        1.904476000      1.512730000      2.214551000
H        1.041350000      2.574205000      1.057798000
N        2.584591000      1.313666000      0.255424000
H        2.666762000      1.791576000     -0.641514000
S        2.032985000     -0.240605000      0.005964000
O        2.874400000     -0.748604000     -1.085411000
O        2.070849000     -0.899241000      1.319731000
H        2.724140000      2.940692000      1.546040000
C        0.210640000     -0.069146000     -0.558688000
C       -0.645792000      0.098472000      0.623564000
C        0.122082000      1.044946000     -1.519214000
C       -1.434221000      1.204126000      0.801882000
H       -0.638646000     -0.698237000      1.370280000
C       -0.670462000      2.140852000     -1.287914000
H        0.711365000      0.975134000     -2.438066000
C       -1.477947000      2.265293000     -0.134929000
H       -2.049211000      1.263973000      1.705682000
H       -0.678839000      2.941105000     -2.034871000
S       -0.115787000     -1.694464000     -1.535921000
C        0.089518000     -2.987462000     -0.341779000
C       -1.001456000     -3.402669000      0.435852000
C        1.329001000     -3.624621000     -0.183233000
C       -0.851085000     -4.432988000      1.364324000
H       -1.969450000     -2.914178000      0.302337000
C        1.471392000     -4.659583000      0.738020000
H        2.179267000     -3.283649000     -0.777086000
C        0.384048000     -5.064296000      1.516018000
H       -1.706444000     -4.747629000      1.968250000
H        2.440588000     -5.152420000      0.853753000
H        0.499743000     -5.875441000      2.239702000
C       -2.188670000      3.510597000      0.166502000
F       -1.460440000      4.414004000      0.895411000
F       -2.557800000      4.192288000     -0.937213000
F       -3.306613000      3.320390000      0.899280000
```

```
35
TS2_4-CF3
C       -1.527032000      3.923297000      0.477564000
H       -2.606409000      4.100098000      0.355533000
H       -1.019792000      4.899954000      0.565852000
N       -1.065659000      3.120155000     -0.637161000
H       -1.263413000      3.539115000     -1.546413000
S        0.574258000      2.671069000     -0.666858000
O        0.911279000      2.708271000     -2.110363000
O        1.328614000      3.564963000      0.248199000
H       -1.386941000      3.378269000      1.421555000
C        0.436434000      0.664981000      0.011615000
C       -0.371188000      0.626739000      1.211932000
C       -0.101306000     -0.069857000     -1.113895000
C       -1.641255000      0.092052000      1.210440000
H        0.037198000      1.065795000      2.125569000
C       -1.374283000     -0.591999000     -1.087477000
H        0.514170000     -0.166746000     -2.010786000
C       -2.192516000     -0.502308000      0.058219000
H       -2.222883000      0.114406000      2.136988000
H       -1.748716000     -1.108437000     -1.976750000
S        2.274097000      0.480262000      0.287258000
C        2.381420000     -1.291889000      0.307118000
C        3.011810000     -1.959959000     -0.749613000
C        1.847210000     -2.032539000      1.370638000
C        3.107953000     -3.351738000     -0.742216000
H        3.421625000     -1.381952000     -1.581453000
C        1.927904000     -3.424060000      1.363512000
H        1.366371000     -1.512231000      2.201837000
C        2.560888000     -4.086558000      0.309979000
H        3.604938000     -3.864264000     -1.570103000
H        1.501103000     -3.994665000      2.192649000
H        2.629026000     -5.177498000      0.310686000
C       -3.515609000     -1.150540000      0.094608000
F       -3.483874000     -2.464808000      0.447080000
F       -4.353256000     -0.575744000      0.979304000
F       -4.138582000     -1.131313000     -1.100672000
```

```
35
P_4-CF3
C        0.428973000      1.713352000      1.045006000
C       -0.107770000      0.458305000      0.733866000
C       -1.389502000      0.365505000      0.203723000
C       -2.153081000      1.520789000      0.013181000
C       -1.631276000      2.771453000      0.348506000
C       -0.338198000      2.870094000      0.855223000
H        0.079224000      3.852342000      1.090903000
H       -2.225315000      3.675630000      0.200937000
C       -3.516507000      1.397616000     -0.592172000
F       -4.210937000      0.380471000     -0.062368000
F       -3.459735000      1.163402000     -1.914894000
F       -4.253377000      2.502940000     -0.430661000
H       -1.752865000     -0.641620000     -0.052195000
H        0.440059000     -0.480365000      0.889794000
S        2.052607000      1.898258000      1.737299000
C        2.941978000      0.565464000      0.964911000
C        3.585585000     -0.380863000      1.767264000
C        4.327889000     -1.402984000      1.173823000
C        4.420699000     -1.487876000     -0.214631000
C        3.775984000     -0.540523000     -1.014054000
C        3.045912000      0.490780000     -0.429679000
H        2.547204000      1.235741000     -1.054426000
H        3.846557000     -0.601901000     -2.103078000
H        4.995031000     -2.294245000     -0.677439000
H        4.826364000     -2.143546000      1.804452000
H        3.492827000     -0.324006000      2.854520000
C        0.485099000     -2.594027000     -2.774031000
N        0.771723000     -2.239286000     -1.402649000
H        1.756119000     -2.402084000     -1.180894000
S       -0.140396000     -3.192409000     -0.213006000
O        0.476986000     -2.640407000      1.052442000
O       -1.544092000     -2.683553000     -0.466165000
H        0.709474000     -3.648241000     -3.043627000
H        1.055439000     -1.948771000     -3.462805000
H       -0.585486000     -2.426000000     -2.970922000
```

```
38
SM_3,5-CF3
C        3.663655000      1.266489000      0.847268000
N        3.393036000     -0.146549000      0.665892000
S        2.937549000     -0.681414000     -0.809568000
O        3.605321000      0.144298000     -1.815407000
O        3.095738000     -2.131822000     -0.826074000
C        1.186281000     -0.361726000     -0.931009000
C        0.292945000     -1.381295000     -0.613104000
C       -1.072337000     -1.106212000     -0.639908000
C       -1.535336000      0.173612000     -0.944355000
C       -0.622956000      1.179811000     -1.246433000
C        0.747107000      0.916936000     -1.258106000
H        1.465066000      1.698708000     -1.512765000
C       -1.103036000      2.568877000     -1.558538000
F       -0.329889000      3.495919000     -0.979746000
F       -1.084634000      2.820923000     -2.874365000
F       -2.353362000      2.774295000     -1.137464000
H       -2.604711000      0.387255000     -0.935283000
C       -2.057926000     -2.212546000     -0.387948000
F       -2.337871000     -2.881214000     -1.517348000
F       -1.595234000     -3.112410000      0.484162000
F       -3.220795000     -1.755157000      0.085212000
H        0.668711000     -2.369326000     -0.347607000
H        2.784837000     -0.582209000      1.414137000
H        2.797138000      1.920071000      0.641525000
H        4.501111000      1.581052000      0.210368000
H        3.954128000      1.411771000      1.897440000
S        1.347429000     -1.385569000      2.785502000
C        0.037899000     -0.246561000      2.616045000
C       -1.311730000     -0.657952000      2.741179000
C       -2.369566000      0.235608000      2.592266000
C       -2.133882000      1.583086000      2.304284000
C       -0.810406000      2.017188000      2.187519000
C        0.249755000      1.127744000      2.347785000
H        1.275349000      1.493854000      2.262927000
H       -0.597883000      3.066627000      1.961693000
H       -2.964447000      2.281094000      2.172266000
H       -3.396227000     -0.130889000      2.688197000
H       -1.514964000     -1.712343000      2.946447000
```

```
38
TS_3,5-CF3
C       -2.513814000      0.427881000      2.689091000
H       -1.409663000      0.399226000      2.728435000
H       -2.895136000     -0.597207000      2.794067000
N       -3.054787000      1.006653000      1.475104000
H       -2.746622000      1.965387000      1.307766000
S       -2.931790000      0.130315000      0.038428000
O       -3.388025000     -1.235168000      0.375913000
O       -3.710559000      0.918622000     -0.940439000
H       -2.873523000      1.013792000      3.548653000
C       -0.881970000      0.101479000     -0.416462000
C       -0.285313000      1.306040000      0.114328000
C       -0.349375000     -1.129994000      0.129340000
C        0.585899000      1.262054000      1.189562000
H       -0.564367000      2.265456000     -0.326906000
C        0.533036000     -1.121798000      1.188863000
H       -0.681738000     -2.074574000     -0.301937000
C        1.008653000      0.064260000      1.778505000
S       -1.110875000      0.089376000     -2.281282000
C        0.554846000     -0.331934000     -2.729541000
C        1.582456000      0.616550000     -2.631508000
C        0.851428000     -1.624740000     -3.178592000
C        2.890424000      0.267956000     -2.964923000
H        1.351741000      1.627705000     -2.288966000
C        2.158940000     -1.964261000     -3.526861000
H        0.050658000     -2.364906000     -3.249249000
C        3.181075000     -1.021061000     -3.415626000
H        3.687273000      1.011517000     -2.878883000
H        2.380473000     -2.975229000     -3.878984000
H        4.206482000     -1.289945000     -3.682183000
H        1.713415000      0.050564000      2.608653000
C        1.026016000      2.567085000      1.779146000
C        0.985906000     -2.422678000      1.778054000
F        2.130219000      2.455170000      2.527592000
F        1.280534000      3.496122000      0.846158000
F        0.076109000      3.090005000      2.579555000
F        2.315896000     -2.461161000      1.960041000
F        0.668152000     -3.482736000      1.026740000
F        0.438896000     -2.633559000      2.989655000


38
P_3,5-CF3
C       -3.528466000      2.731553000      0.954742000
H       -3.741445000      2.232840000     -0.006516000
H       -2.950277000      3.647873000      0.727548000
N       -2.847666000      1.807296000      1.835902000
H       -2.593772000      2.262177000      2.718934000
S       -1.293710000      1.251384000      1.189957000
O       -0.577558000      2.521505000      0.787241000
O       -0.716212000      0.672011000      2.468893000
H       -4.497946000      3.030287000      1.386537000
C        0.839907000     -1.461682000     -0.154130000
C        0.096210000     -0.662970000     -1.028829000
C        2.062205000     -0.997427000      0.337067000
C        0.579764000      0.589199000     -1.397960000
H       -0.863571000     -1.013876000     -1.411774000
C        2.536647000      0.253486000     -0.055991000
H        2.636668000     -1.606368000      1.038661000
C        1.796565000      1.060415000     -0.915763000
S        0.330687000     -3.092143000      0.320195000
C       -1.440482000     -2.947284000      0.384774000
C       -2.218934000     -3.847364000     -0.350349000
C       -2.046730000     -1.984387000      1.200410000
C       -3.610399000     -3.783895000     -0.267643000
H       -1.735945000     -4.589015000     -0.991486000
C       -3.437615000     -1.911180000      1.248961000
H       -1.440989000     -1.272695000      1.774353000
C       -4.221616000     -2.811519000      0.524658000
H       -4.217982000     -4.489421000     -0.840239000
H       -3.904252000     -1.132814000      1.858193000
H       -5.311974000     -2.752678000      0.574545000
H        2.154869000      2.051472000     -1.193368000
C       -0.224519000      1.423635000     -2.353592000
C        3.871400000      0.723821000      0.445030000
F       -1.535425000      1.365221000     -2.104782000
F        0.134168000      2.707194000     -2.345472000
F       -0.064320000      0.989337000     -3.618974000
F        4.875909000      0.276064000     -0.324240000
F        4.121591000      0.294839000      1.687066000
F        3.962855000      2.057298000      0.458646000


38
SM_2,5-CF3
C        0.928987000     -2.942388000     -2.182287000
N        1.303119000     -1.542700000     -2.127295000
S        2.161567000     -0.952581000     -0.895286000
O        2.950994000     -2.046936000     -0.335149000
O        2.817668000      0.274861000     -1.339913000
C        0.971242000     -0.450345000      0.374535000
C       -0.354008000     -0.835497000      0.189946000
C       -1.323472000     -0.493879000      1.128331000
C       -0.982140000      0.248705000      2.254549000
C        0.337677000      0.645372000      2.432824000
C        1.324657000      0.300873000      1.508168000
C        2.706090000      0.870270000      1.740506000
F        3.682168000      0.024564000      1.419341000
F        2.891944000      1.992217000      1.035498000
F        2.883815000      1.194314000      3.026582000
H        0.604953000      1.241829000      3.305735000
H       -1.739775000      0.530203000      2.988252000
C       -2.743372000     -0.957334000      0.953024000
F       -3.015741000     -1.993584000      1.758849000
F       -2.998864000     -1.356056000     -0.294252000
F       -3.620982000      0.004772000      1.256481000
H       -0.646699000     -1.370864000     -0.712959000
H        0.664375000     -0.854394000     -2.621745000
H        0.144431000     -3.032111000     -2.947091000
H        0.520167000     -3.326647000     -1.233391000
H        1.778611000     -3.576032000     -2.476809000
S       -0.920105000      0.187865000     -3.530824000
C       -1.216415000      1.323454000     -2.234772000
C       -2.530941000      1.629467000     -1.810869000
C       -2.776773000      2.515978000     -0.764272000
C       -1.721407000      3.139093000     -0.092338000
C       -0.413560000      2.857234000     -0.497574000
C       -0.165568000      1.977580000     -1.549055000
H        0.866522000      1.757307000     -1.832857000
H        0.432326000      3.321294000      0.019129000
H       -1.914055000      3.826946000      0.734988000
H       -3.809644000      2.715027000     -0.462125000
H       -3.367215000      1.136276000     -2.314505000


38
TS_2,5-CF3
C        2.652494000     -3.416535000     -0.753658000
H        2.156170000     -4.097147000     -0.049509000
H        3.591474000     -3.889653000     -1.080178000
N        2.971241000     -2.185828000     -0.064453000
H        3.460636000     -1.483027000     -0.616174000
S        1.865413000     -1.460869000      0.978466000
O        1.114567000     -2.585839000      1.573004000
O        2.664923000     -0.611340000      1.876642000
H        1.999162000     -3.285712000     -1.634406000
C        0.478281000     -0.188427000      0.116101000
C       -0.769151000     -0.511441000      0.790712000
C        0.905365000      1.189032000      0.298686000
C       -1.423937000      0.392869000      1.589295000
H       -1.175094000     -1.511890000      0.643802000
C        0.208137000      2.070932000      1.126492000
C       -0.951174000      1.709178000      1.796317000
H        0.592971000      3.088781000      1.234333000
S        0.525011000     -0.878024000     -1.645935000
C       -1.051994000     -0.288486000     -2.202676000
C       -2.100535000     -1.195945000     -2.397009000
C       -1.264976000      1.077091000     -2.435318000
C       -3.349406000     -0.742228000     -2.821067000
H       -1.933038000     -2.258850000     -2.206987000
C       -2.520986000      1.526522000     -2.839921000
H       -0.443725000      1.781475000     -2.292919000
C       -3.563915000      0.619496000     -3.036669000
H       -4.162157000     -1.457388000     -2.972728000
H       -2.683366000      2.594036000     -3.010866000
H       -4.545707000      0.975080000     -3.359780000
H       -1.488988000      2.415409000      2.429648000
C        2.091403000      1.701953000     -0.429747000
C       -2.718920000      0.002641000      2.233978000
F        1.802085000      2.241339000     -1.644201000
F        2.727500000      2.679661000      0.237761000
F        3.011824000      0.751733000     -0.685587000
F       -3.037555000     -1.283693000      2.053160000
F       -3.748759000      0.724275000      1.756764000
F       -2.700351000      0.214521000      3.560634000


38
P_2,5-CF3
C       -0.806823000     -1.760580000      2.998534000
H       -0.878369000     -1.816587000      4.099007000
H       -1.098250000     -2.749487000      2.607658000
N        0.553510000     -1.452222000      2.617010000
H        0.639239000     -1.358443000      1.600674000
S        1.124388000      0.107767000      3.227749000
O        2.297277000      0.304696000      2.293315000
O       -0.006730000      1.074010000      2.910403000
H       -1.553925000     -1.019691000      2.656395000
C       -0.974560000      0.116043000     -0.893490000
C        0.321739000     -0.335564000     -1.169230000
C       -1.944364000     -0.826037000     -0.498353000
C        0.640067000     -1.681495000     -1.045479000
H        1.088787000      0.375322000     -1.475803000
C       -1.611789000     -2.178267000     -0.397604000
C       -0.323569000     -2.617768000     -0.666632000
H       -2.373156000     -2.894406000     -0.086893000
S       -1.405280000      1.825594000     -1.092159000
C        0.173306000      2.628143000     -0.925600000
C        0.666539000      3.379888000     -1.996403000
C        0.882528000      2.537155000      0.276742000
C        1.883285000      4.049588000     -1.860676000
H        0.105451000      3.432710000     -2.932704000
C        2.112401000      3.182686000      0.384353000
H        0.500737000      1.947891000      1.123756000
C        2.610555000      3.945312000     -0.674112000
H        2.270322000      4.641774000     -2.693888000
H        2.679552000      3.071155000      1.311742000
H        3.571784000      4.457050000     -0.577215000
H       -0.070513000     -3.675061000     -0.568920000
C       -3.337504000     -0.397535000     -0.118936000
C        2.048855000     -2.149666000     -1.279513000
F       -4.138082000     -1.438578000      0.113987000
F       -3.339966000      0.351054000      0.992842000
F       -3.918522000      0.334527000     -1.079541000
F        2.096028000     -3.137090000     -2.182669000
F        2.591380000     -2.634203000     -0.153736000
F        2.849235000     -1.175220000     -1.714400000


38
SM_2,4-CF3
C       -3.321980000      1.056019000      1.267304000
N       -3.017121000      1.160107000     -0.143105000
S       -2.766324000     -0.158788000     -1.057482000
O       -2.878165000      0.257968000     -2.451273000
O       -3.605932000     -1.245024000     -0.565658000
C       -1.020679000     -0.598536000     -0.852177000
C       -0.167555000      0.131055000     -1.679744000
C        1.212155000     -0.022333000     -1.600776000
C        1.743947000     -0.912015000     -0.675986000
C        0.901997000     -1.649960000      0.153782000
C       -0.482925000     -1.508577000      0.079386000
C       -1.306990000     -2.406211000      0.979344000
F       -1.995300000     -3.313061000      0.286060000
F       -2.166073000     -1.729027000      1.743480000
F       -0.521136000     -3.092288000      1.821639000
```

H	1.331291000	-2.350036000	0.870355000
C	3.227744000	-1.130553000	-0.580080000
F	3.638319000	-1.205735000	0.691064000
F	3.590638000	-2.280669000	-1.167410000
F	3.922803000	-0.156509000	-1.170039000
H	1.862913000	0.559756000	-2.253858000
H	-0.596479000	0.850816000	-2.376707000
H	-2.386360000	1.966900000	-0.419894000
H	-2.471249000	0.724715000	1.885520000
H	-4.161407000	0.365768000	1.425005000
H	-3.624839000	2.056221000	1.609046000
S	-1.015396000	3.532374000	-0.856907000
C	0.390202000	2.949954000	-0.003925000
C	0.301082000	2.193670000	1.189547000
C	1.434692000	1.711852000	1.840247000
C	2.714005000	1.975940000	1.343312000
C	2.829991000	2.736489000	0.176468000
C	1.697566000	3.209947000	-0.482024000
H	1.805126000	3.791805000	-1.402080000
H	3.819989000	2.955317000	-0.235725000
H	3.601754000	1.591294000	1.850902000
H	1.315250000	1.119460000	2.752970000
H	-0.687048000	1.982840000	1.604421000

38

MC_2,4-CF3

C	-3.779023000	1.922092000	0.121684000
H	-4.155533000	1.075624000	-0.467440000
H	-3.070864000	2.494205000	-0.499174000
N	-3.187142000	1.409309000	1.335639000
H	-2.700735000	2.060647000	1.947765000
S	-2.670883000	-0.147632000	1.508613000
O	-2.612607000	-0.373686000	2.954831000
O	-3.553751000	-0.963212000	0.669057000
H	-4.636504000	2.567356000	0.370963000
C	-0.926014000	-0.278083000	0.745474000
C	-0.687368000	-1.580235000	0.104267000
C	-0.730186000	0.844306000	-0.182034000
C	-0.166214000	-1.681919000	-1.177510000
C	-0.228434000	0.701929000	-1.443751000
C	0.072620000	-0.572520000	-1.986574000
H	-0.060932000	1.600930000	-2.044443000
S	0.160422000	0.025145000	2.381059000
C	1.669847000	0.361708000	1.522265000
C	2.098845000	1.684758000	1.343736000
C	2.426074000	-0.681621000	0.967830000
C	3.255675000	1.960093000	0.615279000
H	1.510707000	2.498815000	1.774358000
C	3.570755000	-0.401538000	0.222636000
H	2.098526000	-1.712131000	1.111845000
C	3.988103000	0.918581000	0.043672000
H	3.579308000	2.995999000	0.482683000
H	4.142261000	-1.221730000	-0.220207000
H	4.887282000	1.135769000	-0.538856000
C	0.738897000	-0.698193000	-3.293563000
F	0.619049000	-1.926054000	-3.828104000
F	2.078020000	-0.456676000	-3.248458000
F	0.267609000	0.174368000	-4.208507000
H	0.058972000	-2.680065000	-1.562828000
C	-0.803042000	-2.850025000	0.867003000
F	0.384815000	-3.304310000	1.354461000
F	-1.610555000	-2.769487000	1.929154000
F	-1.261987000	-3.864849000	0.105573000
H	-0.917823000	1.846190000	0.207612000

38

TS2_2,4-CF3

C	-4.198025000	1.881961000	0.869866000
H	-4.318606000	0.789384000	0.979168000
H	-5.145211000	2.302102000	0.497446000
N	-3.133756000	2.278045000	-0.026304000
H	-3.227342000	1.941979000	-0.984018000
S	-1.517699000	2.176512000	0.467802000
O	-0.784422000	3.088310000	-0.429837000
O	-1.549738000	2.504641000	1.909860000
H	-4.002815000	2.311308000	1.861448000
C	-0.539589000	0.303460000	0.266355000
C	0.276871000	0.365991000	-0.934256000
C	0.219883000	0.180016000	1.500976000
C	1.661531000	0.451299000	-0.868563000
C	1.585003000	0.259151000	1.531286000
C	2.343889000	0.415845000	0.344537000
H	2.097270000	0.177109000	2.494829000
S	-2.056467000	-0.774679000	0.231937000
C	-1.339569000	-2.330575000	0.697975000
C	-1.747443000	-2.939976000	1.890453000
C	-0.386612000	-2.961287000	-0.113463000
C	-1.209821000	-4.171272000	2.265871000
H	-2.483054000	-2.440967000	2.526122000
C	0.164196000	-4.179377000	0.280194000
H	-0.081387000	-2.494176000	-1.051217000
C	-0.248307000	-4.789629000	1.466395000
H	-1.536751000	-4.642740000	3.196382000
H	0.914248000	-4.661404000	-0.352439000
H	0.180235000	-5.749110000	1.767086000
C	3.818627000	0.477782000	0.415417000
F	4.392392000	0.641036000	-0.786086000
F	4.371886000	-0.635583000	0.951287000
F	4.266986000	1.489147000	1.193523000
H	2.224738000	0.531004000	-1.800617000
C	-0.364088000	0.351535000	-2.274630000
F	0.382087000	0.960217000	-3.210211000
F	-0.589018000	-0.896936000	-2.758557000
F	-1.568195000	0.954248000	-2.294128000
H	-0.343524000	0.040756000	2.425119000

38

P_2,4-CF3

C	-2.792345000	3.441942000	1.489097000
H	-3.384310000	4.171458000	2.067111000
H	-3.472324000	2.610516000	1.206535000
N	-1.611535000	3.046148000	2.226599000
H	-1.877862000	2.522401000	3.063507000
S	-0.583515000	1.923331000	1.292489000
O	-0.056293000	2.859605000	0.231665000
O	0.446477000	1.612292000	2.361502000
H	-2.476455000	3.934354000	0.555648000
C	0.685727000	-0.941643000	-0.389502000
C	-0.496105000	-1.575209000	0.048348000
C	0.586689000	0.079118000	-1.341161000
C	-1.730587000	-1.170006000	-0.450813000
C	-0.646172000	0.480944000	-1.830325000
C	-1.810025000	-0.144615000	-1.386316000
H	-0.696018000	1.290885000	-2.560615000
S	2.261950000	-1.478951000	0.216902000
C	3.291512000	-0.052102000	-0.043817000
C	3.033366000	1.144834000	0.635949000
C	4.405218000	-0.175690000	-0.879964000
C	3.895718000	2.223517000	0.449262000
H	2.149864000	1.244909000	1.283078000
C	5.271707000	0.906809000	-1.038132000
H	4.589663000	-1.114133000	-1.408702000
C	5.015205000	2.108403000	-0.378281000
H	3.686513000	3.165956000	0.963031000
H	6.144029000	0.810890000	-1.689658000
H	5.689109000	2.958934000	-0.510305000
C	-3.136888000	0.328129000	-1.893595000
F	-4.138735000	-0.482131000	-1.539698000
F	-3.154524000	0.419552000	-3.231039000
F	-3.443717000	1.551363000	-1.430820000
H	-2.639008000	-1.657642000	-0.097265000
C	-0.465206000	-2.654377000	1.095445000
F	-1.666269000	-3.207086000	1.283369000
F	0.372706000	-3.649819000	0.767500000
F	-0.062780000	-2.190501000	2.285631000
H	1.489844000	0.577655000	-1.693728000

38

SM_2,6-CF3

C	0.424607000	2.716798000	4.130663000
H	1.102820000	3.328665000	3.516753000
H	0.938544000	2.462693000	5.068637000
N	0.062277000	1.458213000	3.510641000
H	0.652602000	0.628003000	3.801395000
S	-0.491947000	1.427089000	1.979630000
O	-1.769449000	0.733271000	1.862375000
O	-0.454257000	2.785347000	1.446614000
H	-0.463604000	3.317496000	4.378559000
C	0.707198000	0.351188000	1.136106000
C	0.590751000	-1.051233000	1.269961000
C	1.774535000	0.879387000	0.377372000
C	1.489604000	-1.883237000	0.596750000
C	2.643927000	0.014764000	-0.293739000
C	2.500428000	-1.359050000	-0.191602000
H	3.453842000	0.427105000	-0.893153000
S	2.072027000	-0.791168000	4.502093000
C	3.434305000	-0.383820000	3.494090000
C	3.769096000	0.953805000	3.173816000
C	4.271196000	-1.389194000	2.950605000
C	4.850595000	1.261505000	2.352174000
H	3.153746000	1.759427000	3.580535000
C	5.356328000	-1.078233000	2.134780000
H	4.041272000	-2.433998000	3.178752000
C	5.655689000	0.250606000	1.819358000
H	5.063475000	2.309123000	2.119168000
H	5.973690000	-1.887456000	1.732117000
H	6.503621000	0.493890000	1.173973000
C	2.088107000	2.358823000	0.204195000
F	1.216985000	2.981788000	-0.588955000
F	2.160554000	3.016672000	1.361572000
F	3.288680000	2.520341000	-0.375361000
H	1.393890000	-2.963865000	0.695637000
C	-0.497841000	-1.804685000	2.028996000
F	-0.841262000	-1.283950000	3.199393000
F	-1.604821000	-1.928203000	1.287257000
F	-0.092860000	-3.057821000	2.291769000
H	3.189408000	-2.023891000	-0.715562000

38

TS_2,6-CF3

C	1.984736000	3.118290000	1.297434000
H	1.216986000	3.775922000	0.868404000
H	2.538992000	2.631416000	0.477159000
N	1.352654000	2.183974000	2.202853000
H	1.958491000	1.592936000	2.765523000
S	-0.090110000	1.474421000	1.826426000
O	-0.481061000	0.704954000	3.000809000
O	-0.938050000	2.574041000	1.365903000
H	2.684401000	3.743131000	1.871515000
C	0.211029000	0.389656000	0.316421000
C	-0.488099000	0.830804000	-0.870364000
C	1.585043000	0.006383000	0.086931000
C	0.116939000	0.799355000	-2.124026000
C	2.148740000	-0.005171000	-1.184860000
C	1.438974000	0.405419000	-2.309279000
H	3.181638000	-0.348343000	-1.293232000
S	-0.848819000	-1.524291000	1.150051000
C	-0.750363000	-2.332488000	-0.397613000
C	0.413776000	-3.028455000	-0.782907000
C	-1.810696000	-2.265532000	-1.323443000
C	0.521685000	-3.605767000	-2.046458000
H	1.240950000	-3.100431000	-0.074855000
C	-1.702773000	-2.846622000	-2.585482000
H	-2.724831000	-1.741717000	-1.037793000
C	-0.532193000	-3.511149000	-2.958601000
H	1.439702000	-4.132041000	-2.323953000
H	-2.538385000	-2.773574000	-3.287673000
H	-0.445205000	-3.960788000	-3.951299000
C	2.493074000	-0.503675000	1.164309000
F	3.594747000	0.270989000	1.301972000
F	1.937024000	-0.579592000	2.374785000
F	2.970424000	-1.734407000	0.889817000
H	-0.477334000	1.099068000	-2.991047000

C	-1.934872000	1.249776000	-0.897866000
F	-2.641831000	0.903195000	0.174198000
F	-2.098773000	2.573457000	-1.080905000
F	-2.573477000	0.690211000	-1.955835000
H	1.894524000	0.401504000	-3.300201000

38
P_2,6-CF3

C	2.034599000	4.896942000	0.992506000
H	2.549136000	5.865942000	0.870153000
H	2.022871000	4.673228000	2.071981000
N	2.759417000	3.868956000	0.278031000
H	2.290538000	2.962430000	0.368423000
S	2.845984000	4.150864000	-1.465381000
O	3.291651000	2.770288000	-1.904463000
O	1.410892000	4.434984000	-1.877319000
H	0.988888000	5.036540000	0.655671000
C	-0.565979000	-0.948620000	-0.646179000
C	-1.602716000	0.001330000	-0.653939000
C	0.734474000	-0.533150000	-0.986342000
C	-1.335369000	1.333726000	-0.975920000
C	0.989070000	0.800950000	-1.303753000
C	-0.043707000	1.733836000	-1.295916000
H	1.994563000	1.164770000	-1.556520000
S	-0.904412000	-2.658400000	-0.265554000
C	-0.663698000	-2.723257000	1.491734000
C	-0.304240000	-1.622244000	2.274442000
C	-0.856136000	-3.974396000	2.092851000
C	-0.140139000	-1.778790000	3.650320000
H	-0.150575000	-0.642694000	1.818223000
C	-0.689279000	-4.116935000	3.468372000
H	-1.136224000	-4.839518000	1.485049000
C	-0.330708000	-3.021053000	4.254797000
H	0.141216000	-0.911958000	4.254125000
H	-0.841820000	-5.097456000	3.926921000
H	-0.199976000	-3.135440000	5.333504000
C	1.888742000	-1.507927000	-1.019461000
F	3.033355000	-0.898848000	-1.341541000
F	2.092306000	-2.099065000	0.164647000
F	1.706036000	-2.479289000	-1.920807000
H	-2.150238000	2.059140000	-0.974574000
C	-3.025605000	-0.383447000	-0.320140000
F	-3.138484000	-0.905314000	0.906983000
F	-3.841000000	0.675256000	-0.358768000
F	-3.522164000	-1.279043000	-1.179937000
H	0.209102000	2.776140000	-1.542767000

32
SM_2,4,6-Cl

C	2.889376000	-1.757780000	1.941240000
N	2.128698000	-1.297445000	0.805310000
S	2.535974000	0.042970000	-0.003485000
O	3.145016000	-0.249234000	-1.294609000
O	3.268171000	0.931507000	0.897232000
C	0.904741000	0.768582000	-0.296302000
C	0.347332000	0.985103000	-1.570116000
C	-0.919858000	1.551199000	-1.721066000
C	-1.647070000	1.911309000	-0.596242000
C	-1.135655000	1.717806000	0.680692000
C	0.124848000	1.146654000	0.816453000
Cl	0.665393000	0.917706000	2.442283000
H	-1.709787000	1.991893000	1.565321000
Cl	-3.222886000	2.592947000	-0.784104000
H	-1.327552000	1.699148000	-2.721035000
Cl	1.130133000	0.587004000	-3.059188000
H	1.568142000	-1.985383000	0.230587000
H	3.889125000	-2.144075000	1.675059000
H	2.321601000	-2.573267000	2.412073000
H	3.009246000	-0.951827000	2.679596000
S	0.147321000	-3.102590000	-0.846741000
C	-1.194681000	-2.283818000	-0.089297000
C	-1.286078000	-2.118501000	1.314151000
C	-2.365658000	-1.468379000	1.908093000
C	-3.410382000	-0.958817000	1.131909000
C	-3.346786000	-1.112422000	-0.256233000
C	-2.264675000	-1.754812000	-0.852907000
H	-2.222630000	-1.856209000	-1.941343000
H	-4.146752000	-0.712247000	-0.886558000
H	-4.254738000	-0.444846000	1.598362000
H	-2.388550000	-1.354906000	2.996520000
H	-0.481529000	-2.513979000	1.940071000

32
TS_2,4,6-Cl

C	3.089199000	2.546350000	-1.193066000
H	2.105296000	3.043854000	-1.207742000
H	3.728223000	2.995693000	-1.967989000
N	3.021386000	1.118873000	-1.426478000
H	2.605010000	0.849412000	-2.316903000
S	2.518261000	0.093513000	-0.209145000
O	2.834919000	-1.252920000	-0.699763000
O	3.161485000	0.600573000	1.012063000
H	3.561780000	2.721660000	-0.217634000
C	0.537508000	0.065190000	-0.016874000
C	-0.147506000	0.079639000	-1.305281000
C	0.180328000	-1.148346000	0.713369000
C	-1.004262000	-0.904322000	-1.766104000
C	-0.670173000	-2.137396000	0.262291000
C	-1.268609000	-2.035480000	-0.995305000
H	-1.481066000	-0.771492000	-2.739230000
H	-0.871460000	-2.994421000	0.908087000
S	0.320905000	1.705695000	1.020770000
C	-1.446475000	1.712783000	1.025925000
C	-2.160774000	2.599644000	0.208790000
C	-2.149178000	0.813666000	1.842735000
C	-3.555009000	2.587325000	0.209517000
H	-1.613345000	3.289280000	-0.437141000
C	-3.543346000	0.792470000	1.825894000
H	-1.591604000	0.135215000	2.492062000
C	-4.249205000	1.679463000	1.011542000
H	-4.103209000	3.285524000	-0.428814000
H	-4.081670000	0.083102000	2.460312000
H	-5.342263000	1.665249000	1.003687000
Cl	0.870623000	-1.346474000	2.306527000
Cl	-2.350788000	-3.278542000	-1.573361000
Cl	0.024389000	1.496009000	-2.329814000

32
P_2,4,6-Cl

C	1.419195000	-1.391526000	-3.348443000
H	0.769158000	-2.210803000	-3.693345000
H	2.459504000	-1.648393000	-3.642129000
N	1.215028000	-1.196109000	-1.930777000
H	1.835110000	-0.465948000	-1.577127000
S	1.594611000	-2.633341000	-0.951476000
O	1.333462000	-2.058238000	0.423432000
O	0.513383000	-3.581617000	-1.420541000
H	1.133979000	-0.482808000	-3.904439000
C	-0.274211000	1.302735000	1.017922000
C	0.872761000	0.979496000	1.762166000
C	-0.071687000	1.833556000	-0.269919000
C	2.157440000	1.161992000	1.257808000
C	1.199829000	2.024058000	-0.800384000
C	2.302494000	1.685080000	-0.020902000
H	3.027317000	0.884869000	1.852776000
H	1.324189000	2.428462000	-1.805112000
S	-1.893933000	1.094019000	1.693391000
C	-2.535671000	-0.194212000	0.644780000
C	-1.713319000	-1.205988000	0.142031000
C	-3.906080000	-0.186835000	0.360559000
C	-2.263418000	-2.195780000	-0.672142000
H	-0.635206000	-1.252500000	0.353694000
C	-4.449591000	-1.199940000	-0.429275000
H	-4.543487000	0.614898000	0.743280000
C	-3.630300000	-2.200962000	-0.955959000
H	-1.567167000	-2.945876000	-1.069405000
H	-5.520712000	-1.192393000	-0.648927000
H	-4.060577000	-2.980932000	-1.590166000
Cl	-1.424622000	2.258921000	-1.255208000
Cl	3.892499000	1.888667000	-0.676757000
Cl	0.722036000	0.316628000	3.349384000

32
SM_per-F

C	2.670138000	0.838149000	2.650229000
H	2.699369000	0.448862000	3.676295000
H	1.834242000	1.551419000	2.563584000
N	2.606868000	-0.257572000	1.701074000
H	2.730157000	0.032256000	0.688633000
S	1.429571000	-1.374927000	1.825739000
O	1.829846000	-2.545207000	1.062234000
O	1.064249000	-1.521403000	3.229629000
H	3.605772000	1.382362000	2.458304000
C	0.005434000	-0.669084000	0.973858000
C	-0.304113000	-0.998520000	-0.349559000
C	-0.760348000	0.317632000	1.602105000
C	-1.350669000	-0.363969000	-1.011908000
C	-1.805772000	0.952125000	0.944610000
C	-2.104319000	0.607338000	-0.367357000
S	2.713005000	0.709941000	-1.332759000
C	1.341364000	1.788153000	-1.353055000
C	0.478506000	1.851392000	-2.475140000
C	1.025569000	2.645527000	-0.271934000
C	-0.625413000	2.699011000	-2.506276000
H	0.687575000	1.196977000	-3.326157000
C	-0.085149000	3.486727000	-0.302009000
H	1.674243000	2.640117000	0.607546000
C	-0.925486000	3.523577000	-1.417369000
H	-1.271431000	2.704895000	-3.389730000
H	-0.299961000	4.119834000	0.564578000
H	-1.801155000	4.177102000	-1.436034000
F	-0.501419000	0.708686000	2.841169000
F	-2.495842000	1.905256000	1.550194000
F	-3.091659000	1.206846000	-1.002290000
F	-1.620256000	-0.673699000	-2.270241000
F	0.353959000	-1.912866000	-1.032435000

32
TS_per-F

C	2.096395000	-0.588820000	-3.416928000
H	1.079940000	-0.163441000	-3.446116000
H	2.038587000	-1.660255000	-3.651758000
N	2.764841000	-0.426109000	-2.141268000
H	2.898559000	0.543116000	-1.852188000
S	2.249966000	-1.343591000	-0.831235000
O	2.099353000	-2.719698000	-1.343215000
O	3.241630000	-1.070800000	0.221982000
H	2.704990000	-0.102839000	-4.194152000
C	0.491966000	-0.565920000	-0.075583000
C	0.480544000	-0.828355000	1.336139000
C	0.388173000	0.841474000	-0.345613000
C	0.394624000	0.142136000	2.313059000
C	0.299178000	1.817812000	0.629383000
C	0.315742000	1.496018000	1.985159000
S	-0.723740000	-1.663811000	-1.099951000
C	-2.197807000	-0.844100000	-0.563296000
C	-2.699970000	-1.074028000	0.726086000
C	-2.848319000	0.068810000	-1.403746000
C	-3.832828000	-0.391744000	1.168080000
H	-2.194714000	-1.790421000	1.378077000
C	-3.989115000	0.737871000	-0.962069000
H	-2.447264000	0.258152000	-2.402144000
C	-4.480494000	0.512830000	0.325110000
H	-4.214697000	-0.572151000	2.176483000
H	-4.492204000	1.446902000	-1.624945000
H	-5.370599000	1.044003000	0.672159000
F	0.283776000	1.238616000	-1.632465000
F	0.187855000	3.096820000	0.262447000
F	0.253733000	2.445188000	2.933658000
F	0.385819000	-0.220758000	3.598109000
F	0.526223000	-2.117223000	1.722605000

32
P_per-F

C	-3.136679000	-1.039129000	-0.272183000
H	-2.391566000	-1.624474000	0.293298000
H	-2.944998000	-1.224157000	-1.346591000
N	-3.046317000	0.353475000	0.115341000
H	-3.656564000	0.935485000	-0.468189000
S	-1.435030000	1.018859000	-0.153101000
O	-1.116836000	0.661797000	-1.592341000
O	-1.751200000	2.494916000	-0.010672000
H	-4.130186000	-1.445217000	-0.019640000
C	1.881533000	0.497240000	1.448601000
C	1.168795000	-0.654439000	1.787404000
C	2.103829000	0.755890000	0.093142000
C	0.669397000	-1.502856000	0.807996000
C	1.634589000	-0.102103000	-0.894657000
C	0.913521000	-1.233686000	-0.533868000
S	2.535261000	1.566184000	2.691741000
C	1.034433000	2.134312000	3.469698000
C	1.015101000	2.252901000	4.863080000
C	-0.088005000	2.486126000	2.715442000
C	-0.134992000	2.723299000	5.497943000
H	1.890717000	1.965552000	5.451030000
C	-1.241683000	2.921385000	3.363131000
H	-0.100390000	2.422705000	1.625276000
C	-1.269798000	3.047622000	4.753005000
H	-0.147092000	2.816126000	6.587071000
H	-2.114939000	3.156591000	2.749615000
H	-2.175096000	3.395550000	5.257008000
F	2.758019000	1.844802000	-0.283171000
F	1.854737000	0.164233000	-2.170092000
F	0.434073000	-2.040227000	-1.460452000
F	-0.048338000	-2.567425000	1.143582000
F	0.921455000	-0.938659000	3.056629000

33
SM_4-CN

C	-2.691302000	-0.013667000	2.024230000
H	-2.752323000	-0.918881000	2.644747000
H	-3.553169000	0.626086000	2.257998000
N	-2.732994000	-0.436231000	0.638896000
H	-2.133579000	-1.278596000	0.411117000
S	-2.631471000	0.694332000	-0.534677000
O	-2.967785000	0.045718000	-1.797784000
O	-3.373847000	1.874469000	-0.092131000
H	-1.766223000	0.524687000	2.300303000
C	-0.909047000	1.152615000	-0.630586000
C	-0.067193000	0.418426000	-1.465611000
C	-0.426782000	2.184211000	0.175616000
C	1.289187000	0.714205000	-1.488969000
C	0.932088000	2.474963000	0.163430000
C	1.788768000	1.735887000	-0.666358000
H	1.333092000	3.271438000	0.792593000
S	-0.807025000	-2.912821000	0.031607000
C	0.712464000	-2.209698000	0.520762000
C	1.916625000	-2.520348000	-0.157231000
C	0.821494000	-1.302860000	1.603115000
C	3.135402000	-1.953320000	0.209091000
H	1.872315000	-3.218421000	-0.998337000
C	2.040217000	-0.731104000	1.962598000
H	-0.079214000	-1.046467000	2.165769000
C	3.213125000	-1.046645000	1.270387000
H	4.038896000	-2.218899000	-0.348924000
H	2.073313000	-0.026137000	2.799345000
H	4.167388000	-0.593541000	1.550410000
H	1.966760000	0.151536000	-2.132734000
H	-1.112441000	2.757843000	0.801500000
H	-0.473419000	-0.391540000	-2.072355000
C	3.192331000	2.015732000	-0.663901000
N	4.330682000	2.237343000	-0.658020000

33
TS_4-CN

C	-3.531857000	1.519232000	-0.442881000
H	-4.415093000	2.113288000	-0.160610000
H	-3.678233000	1.164915000	-1.472684000
N	-3.440196000	0.370620000	0.433978000
H	-3.269595000	0.611427000	1.410976000
S	-2.417849000	-0.910734000	-0.003372000
O	-2.737477000	-1.966987000	0.986632000
O	-2.729562000	-1.170274000	-1.429366000
H	-2.648830000	2.183435000	-0.421151000
C	-0.353813000	-0.150384000	0.197702000
C	-0.002048000	0.340176000	-1.115680000
C	-0.391484000	0.837454000	1.253850000
C	0.115177000	1.682653000	-1.367512000
C	-0.278022000	2.178216000	0.982761000
C	-0.045637000	2.648580000	-0.337211000
H	-0.340336000	2.898353000	1.803291000
S	0.321600000	-1.797432000	0.640525000
C	2.054737000	-1.575725000	0.333620000
C	2.767853000	-2.682993000	-0.148082000
C	2.738628000	-0.378996000	0.592833000
C	4.143631000	-2.597285000	-0.356472000
H	2.237372000	-3.613588000	-0.367940000
C	4.110383000	-0.296204000	0.359230000
H	2.197171000	0.486448000	0.979375000
C	4.820580000	-1.402210000	-0.110474000
H	4.686325000	-3.470294000	-0.728861000
H	4.630682000	0.644622000	0.558496000
H	5.897045000	-1.332219000	-0.285594000
H	0.354085000	2.018535000	-2.380351000
H	-0.531669000	0.495371000	2.282358000
H	0.145961000	-0.384948000	-1.918420000
C	0.079612000	4.028710000	-0.606345000
N	0.176930000	5.170675000	-0.829922000

33
P_4-CN

C	1.716879000	-3.114646000	-2.727645000
H	2.810207000	-3.277973000	-2.835671000
H	1.442507000	-2.274879000	-3.384666000
N	1.276749000	-2.817555000	-1.380997000
H	1.537544000	-3.573827000	-0.743914000
S	2.045970000	-1.356639000	-0.707965000
O	1.440751000	-0.300724000	-1.608986000
O	1.438520000	-1.377365000	0.677537000
H	1.208621000	-4.016185000	-3.106881000
C	-0.555814000	2.026077000	1.518219000
C	-0.228993000	3.148161000	2.300388000
C	0.235398000	1.697930000	0.411615000
C	0.867964000	3.932513000	1.979543000
C	1.335456000	2.481051000	0.090633000
C	1.660464000	3.604803000	0.865555000
H	1.952222000	2.179612000	-0.758172000
S	-1.941946000	1.081037000	2.077121000
C	-2.448759000	0.095226000	0.684022000
C	-1.658133000	-0.943169000	0.178145000
C	-3.722864000	0.338354000	0.155180000
C	-2.146526000	-1.713996000	-0.877471000
H	-0.644989000	-1.138740000	0.558630000
C	-4.208570000	-0.458209000	-0.880800000
H	-4.330207000	1.153727000	0.555848000
C	-3.418074000	-1.483182000	-1.402333000
H	-1.501220000	-2.495292000	-1.288736000
H	-5.204993000	-0.267728000	-1.287810000
H	-3.793976000	-2.100642000	-2.222446000
H	1.112910000	4.805763000	2.587455000
H	0.062827000	0.812697000	-0.202077000
H	-0.844858000	3.413441000	3.163784000
C	2.793136000	4.406515000	0.530270000
N	3.714238000	5.057945000	0.255942000

7
thioacid

S	-0.690108000	0.911067000	-0.735626000
C	-0.112822000	-0.260518000	0.402960000
C	-0.731105000	-0.184695000	1.798057000
O	0.738952000	-1.122225000	0.190724000
H	-0.474986000	0.775736000	2.273638000
H	-0.361320000	-1.009411000	2.428832000
H	-1.829615000	-0.225264000	1.740049000

36
SM NMs + thioacid

C	-3.770028000	-0.020797000	0.797988000
N	-3.157306000	-1.216437000	0.253176000
S	-2.035163000	-2.032162000	1.081318000
O	-1.889867000	-3.362278000	0.508071000
O	-2.351295000	-1.934060000	2.499519000
C	-0.409871000	-1.225548000	0.817416000
C	0.128247000	-0.304373000	1.742944000
C	1.381372000	0.267086000	1.508665000
C	2.107714000	-0.052502000	0.376227000
C	1.580959000	-0.937596000	-0.550347000
C	0.327584000	-1.511727000	-0.354740000
C	-0.081509000	-2.465234000	-1.473677000
F	-1.356309000	-2.390136000	-1.826243000
F	0.200370000	-3.733235000	-1.163197000
F	0.618870000	-2.195744000	-2.587706000
H	2.147584000	-1.177066000	-1.449466000
C	3.447850000	0.577975000	0.115512000
F	4.345392000	-0.329804000	-0.281118000
F	3.373850000	1.498690000	-0.853093000
F	3.938474000	1.182087000	1.198573000
H	1.797269000	0.968634000	2.228801000
C	-0.519043000	0.147719000	3.048945000
F	-0.517591000	-0.803238000	3.979260000
F	0.166224000	1.174954000	3.578234000
F	-1.761309000	0.597960000	2.887498000
S	-1.382465000	1.034751000	-1.868458000
C	-2.588355000	0.237889000	-2.795618000
C	-2.655271000	0.570919000	-4.274752000
O	-3.389227000	-0.610130000	-2.367914000
H	-2.578135000	1.653961000	-4.445646000
H	-3.054675000	-1.155414000	-0.793537000
H	-3.149093000	0.874970000	0.634377000
H	-3.973455000	-0.149516000	1.867600000
H	-4.727133000	0.123592000	0.276160000
H	-3.585515000	0.180702000	-4.715639000
H	-1.799560000	0.100074000	-4.786604000

36
TS NMs + thioacid

C	-4.205280000	-0.800818000	0.920780000
N	-3.264200000	-1.736171000	0.336140000
S	-1.734958000	-1.887101000	0.908180000
O	-1.252284000	-3.211800000	0.513515000
O	-1.755950000	-1.568332000	2.338476000
C	-0.510593000	-0.658755000	0.083341000
C	0.063855000	0.241980000	1.086305000
C	1.428346000	0.371122000	1.276387000
C	2.359053000	-0.367985000	0.547280000
C	1.864930000	-1.229585000	-0.440121000
C	0.515831000	-1.390305000	-0.663659000
C	0.164085000	-2.246305000	-1.840381000
F	-1.147511000	-2.374575000	-2.045113000
F	0.681212000	-3.482696000	-1.761673000
F	0.663208000	-1.728045000	-2.987727000
H	2.566585000	-1.797433000	-1.057502000
C	3.816776000	-0.187308000	0.742367000
F	4.503358000	-1.325899000	0.543389000
F	4.365051000	0.714388000	-0.104000000
F	4.117819000	0.245256000	1.977064000
H	1.782401000	1.071075000	2.037734000
C	-0.767941000	1.166972000	1.922759000
F	-0.565407000	0.999491000	3.241463000
F	-0.445923000	2.461580000	1.685338000
F	-2.081141000	1.076014000	1.713141000
S	-1.509955000	0.608012000	-1.218170000
C	-2.781817000	0.072497000	-2.332899000

C	-2.963826000	1.057476000	-3.456953000
O	-3.436484000	-0.949590000	-2.276859000
H	-2.668671000	2.079075000	-3.182772000
H	-3.279168000	-1.740648000	-0.697946000
H	-4.107778000	0.222253000	0.523360000
H	-4.064022000	-0.772670000	2.008287000
H	-5.223315000	-1.164908000	0.716232000
H	-4.013239000	1.035850000	-3.784243000
H	-2.329229000	0.730361000	-4.297468000

36
P NMs + thioacid

C	-2.446575000	-1.197863000	2.154119000
N	-2.130923000	-2.004519000	0.999373000
S	-0.788252000	-3.128920000	1.274874000
O	-0.550835000	-3.570813000	-0.154680000
O	-1.456467000	-4.183004000	2.117660000
C	-0.149521000	0.488457000	-1.149196000
C	-0.129219000	1.041430000	0.144421000
C	0.600251000	0.429287000	1.156872000
C	1.306026000	-0.741253000	0.899201000
C	1.288024000	-1.306015000	-0.369451000
C	0.568085000	-0.693151000	-1.390898000
C	0.556456000	-1.362986000	-2.746070000
F	-0.678762000	-1.696610000	-3.133638000
F	1.280495000	-2.481848000	-2.748667000
F	1.068076000	-0.571889000	-3.697037000
H	1.810871000	-2.239723000	-0.561248000
C	2.139389000	-1.343137000	1.995917000
F	2.493266000	-2.601074000	1.739563000
F	3.277191000	-0.646100000	2.161520000
F	1.511275000	-1.322871000	3.173950000
H	0.610039000	0.865323000	2.155916000
C	-0.908889000	2.290055000	0.487290000
F	-0.758676000	2.620829000	1.772291000
F	-0.519169000	3.345942000	-0.232047000
F	-2.221608000	2.130356000	0.282730000
S	-1.019115000	1.308651000	-2.467094000
C	-2.642104000	0.521037000	-2.329164000
C	-3.565136000	0.968804000	-3.416242000
O	-2.905073000	-0.279874000	-1.478060000
H	-3.368335000	2.003813000	-3.728599000
H	-1.878592000	-1.420602000	0.200262000
H	-1.585434000	-0.648964000	2.593669000
H	-2.860662000	-1.835251000	2.952926000
H	-3.213110000	-0.448003000	1.897407000
H	-4.603755000	0.860993000	-3.075660000
H	-3.408238000	0.309907000	-4.286427000

50
2z (protected amine)

N	-0.963450000	1.135059000	0.243664000
S	0.457262000	1.936333000	0.445585000
O	0.693375000	2.772761000	-0.701867000
O	0.499308000	2.510785000	1.762442000
C	1.728765000	0.608232000	0.340784000
C	1.524110000	-0.687286000	0.853907000
C	2.941703000	0.866013000	-0.333948000
C	2.401864000	-1.717148000	0.526835000
C	3.805565000	-0.180680000	-0.642246000
C	3.513064000	-1.474827000	-0.257023000
H	4.717946000	0.024857000	-1.180472000
H	2.213666000	-2.713375000	0.899214000
C	-2.454210000	-0.184559000	-1.227292000
C	-3.673483000	0.311421000	-0.445316000
C	-3.256372000	0.618177000	1.003738000
C	-2.108761000	1.619461000	1.053789000
C	-1.310339000	0.820806000	-1.163686000
H	-2.710867000	-0.331343000	-2.278939000
H	-2.134869000	-1.147776000	-0.832709000
H	-4.017976000	1.237936000	-0.907898000
H	-4.091078000	1.041310000	1.567086000
H	-2.962417000	-0.308738000	1.499784000
H	-1.774476000	1.757996000	2.077268000
H	-2.447684000	2.589962000	0.672308000
H	-0.430909000	0.408015000	-1.656603000
H	-1.583315000	1.741113000	-1.690140000
C	-4.791674000	-0.729783000	-0.464053000
O	-4.549411000	-1.927824000	-0.463433000
N	-6.077425000	-0.257304000	-0.512817000
C	-6.575803000	1.068545000	-0.198475000
H	-6.899973000	1.123920000	0.844260000
H	-5.808612000	1.815724000	-0.373626000
H	-7.425171000	1.291705000	-0.844862000
O	-7.081701000	-1.218826000	-0.343826000
C	-7.515942000	-1.728554000	-1.608973000
H	-7.890377000	-0.925183000	-2.249378000
H	-6.709470000	-2.265116000	-2.111106000
H	-8.327770000	-2.418451000	-1.378843000
C	3.476419000	2.247254000	-0.718506000
F	4.830975000	2.215514000	-0.766453000
F	3.080428000	2.657726000	-1.934395000
F	3.173748000	3.195982000	0.179547000
C	0.420795000	-1.117753000	1.815862000
F	-0.622235000	-1.689862000	1.183844000
F	0.899829000	-2.057521000	2.665728000
F	-0.046743000	-0.133659000	2.590501000
C	4.404798000	-2.619674000	-0.665895000
F	5.638937000	-2.210181000	-1.007010000
F	4.542534000	-3.529240000	0.318190000
F	3.900048000	-3.277783000	-1.734862000

28
1z (deprotected amine)

N	3.486552000	-0.071496000	0.199896000
C	1.458555000	-1.289529000	-0.436906000
C	0.617937000	-0.024883000	-0.214273000
C	1.321174000	0.905922000	0.795703000
C	2.758058000	1.184849000	0.362745000
C	2.888061000	-0.923770000	-0.827085000
H	1.009637000	-1.905310000	-1.220592000
H	1.470611000	-1.882526000	0.478713000
H	0.551358000	0.500525000	-1.168925000
H	0.778501000	1.849543000	0.891833000
H	1.327208000	0.427377000	1.778852000
H	3.264390000	1.787491000	1.119864000
H	2.741532000	1.775846000	-0.569825000
H	3.491021000	-1.829265000	-0.922452000
H	2.879406000	-0.431254000	-1.815324000
C	-0.783702000	-0.374884000	0.279245000
O	-0.988310000	-1.331350000	1.012102000
N	-1.814223000	0.417003000	-0.165642000
C	-1.756299000	1.754852000	-0.724335000
H	-1.878415000	2.508557000	0.058587000
H	-0.809774000	1.917353000	-1.229916000
H	-2.558803000	1.870504000	-1.453768000
O	-3.055096000	0.193243000	0.447518000
C	-3.829203000	-0.746892000	-0.303053000
H	-3.989427000	-0.396930000	-1.326714000
H	-3.351510000	-1.727898000	-0.311844000
H	-4.787141000	-0.810876000	0.213231000
H	4.454865000	0.119180000	-0.030709000

7. References

[1] Y. Kosugi, M. Akakura, K. Ishihara, *Tetrahedron* **2007**, *63*, 6191–6203.
[2] V. G. Chandrashekhar, W. Baumann, M. Beller, R. V. Jagadeesh, *Science* **2022**, *376*, 1433–1441.
[3] M. C. Schopohl, K. Bergander, O. Kataeva, R. Fröhlich, S. R. Waldvogel, *Synthesis* **2003**, *2003*, 2689–2694.
[4] C. R. Gonçalves, M. Lemmerer, C. J. Teskey, P. Adler, D. Kaiser, B. Maryasin, L. González, N. Maulide, *J. Am. Chem. Soc.* **2019**, *141*, 18437–18443.
[5] Y. Zheng, L. Andna, O. Bistri, L. Miesch, *Org. Lett.* **2020**, *22*, 6771–6775.
[6] X. Zhou, L. Guo, H. Zhang, R. Y. Xia, C. Yang, W. Xia, *Adv. Synth. Catal.* **2022**, *364*, 1526–1531.
[7] J. Iqbal, A.-U. -Rehman, M. A. Abbasi, S. Z. Siddiqui, S. Rasool, M. Rehan, S. A. Ali Shah, *Asian J. Chem.* **2017**, *29*, 1901–1906.
[8] R. J. Armstrong, W. Niwetmarin, V. K. Aggarwal, *Org. Lett.* **2017**, *19*, 2762–2765.
[9] M. E. Kieffer, K. V. Chuang, S. E. Reisman, *Chem. Sci.* **2012**, *3*, 3170–3174.
[10] Z. Pan, L. Shen, D. Song, Z. Xie, F. Ling, W. Zhong, *Journal of Organic Chemistry* **2018**, *83*, 11502–11509.
[11] P. Pracht, F. Bohle, S. Grimme, *Phys. Chem. Chem. Phys.* **2020**, *22*, 7169–7192.
[12] S. Grimme, *J. Chem. Theory Comput.* **2019**, *15*, 2847–2862.
[13] J. P. Perdew, K. Burke, M. Ernzerhof, *Phys. Rev. Lett.* **1996**, *77*, 3865–3868.
[14] J. P. Perdew, K. Burke, M. Ernzerhof, *Phys. Rev. Lett.* **1997**, *78*, 1396–1396.
[15] C. Adamo, V. Barone, *J. Chem. Phys.* **1999**, *110*, 6158–6170.
[16] S. Grimme, J. Antony, S. Ehrlich, H. Krieg, *J. Chem. Phys.* **2010**, *132*, 154104.
[17] S. Grimme, S. Ehrlich, L. Goerigk, *J. Comput. Chem.* **2011**, *32*, 1456–1465.
[18] F. Weigend, *Phys. Chem. Chem. Phys.* **2006**, *8*, 1057–1065.
[19] F. Weigend, R. Ahlrichs, *Phys. Chem. Chem. Phys.* **2005**, *7*, 3297–3305.
[20] Gaussian 16, Revision C. 01, M. J. Frisch, G. W. Trucks, H. B. Schlegel, G. E. Scuseria, M. A. Robb, J. R. Cheeseman, G. Scalmani, V. Barone, G. A. Petersson, H. Nakatsuji, X. Li, M. Caricato, A. V. Marenich, J. Bloino, B. G. Janesko, R. Gomperts, B. Mennucci, H. P. Hratchian, J. V. Ortiz, A. F. Izmaylov, J. L. Sonnenberg, D. Williams-Young, F. Ding, F. Lipparini, F. Egidi, J. Goings, B. Peng, A. Petrone, T. Henderson, D. Ranasinghe, V. G. Zakrzewski, J. Gao, N. Rega, G. Zheng, W. Liang, M. Hada, M. Ehara, K. Toyota, R. Fukuda, J. Hasegawa, M. Ishida, T. Nakajima, Y. Honda, O. Kitao, H. Nakai, T. Vreven, K. Throssell, J. A. Montgomery, J. E. Peralta, F. Ogliaro, M. J. Bearpark, J. J. Heyd, E. N. Brothers, K. N. Kudin, V. N. Staroverov, T. A. Keith, R. Kobayashi, J. Normand, K. Raghavachari, A. P. Rendell, J. C. Burant, S. S. Iyengar, J. Tomasi, M. Cossi, J. M. Millam, M. Klene, C. Adamo, R. Cammi, J. W. Ochterski, R. L. Martin, K. Morokuma, O. Farkas, J. B. Foresman, and D. J. Fox, Gaussian, Inc. , Wallin, **n.d.**
[21] E. Cancès, B. Mennucci, J. Tomasi, *J. Chem. Phys.* **1997**, *107*, 3032–3041.
[22] A. V. Marenich, C. J. Cramer, D. G. Truhlar, *J. Phys. Chem. B* **2009**, *113*, 6378–6396.
[23] A. D. Becke, *J. Chem. Phys.* **1993**, *98*, 5648–5652.

8. NMR Spectra

2,4,6-Tris(trifluoromethyl)benzenesulfonyl chloride (5)

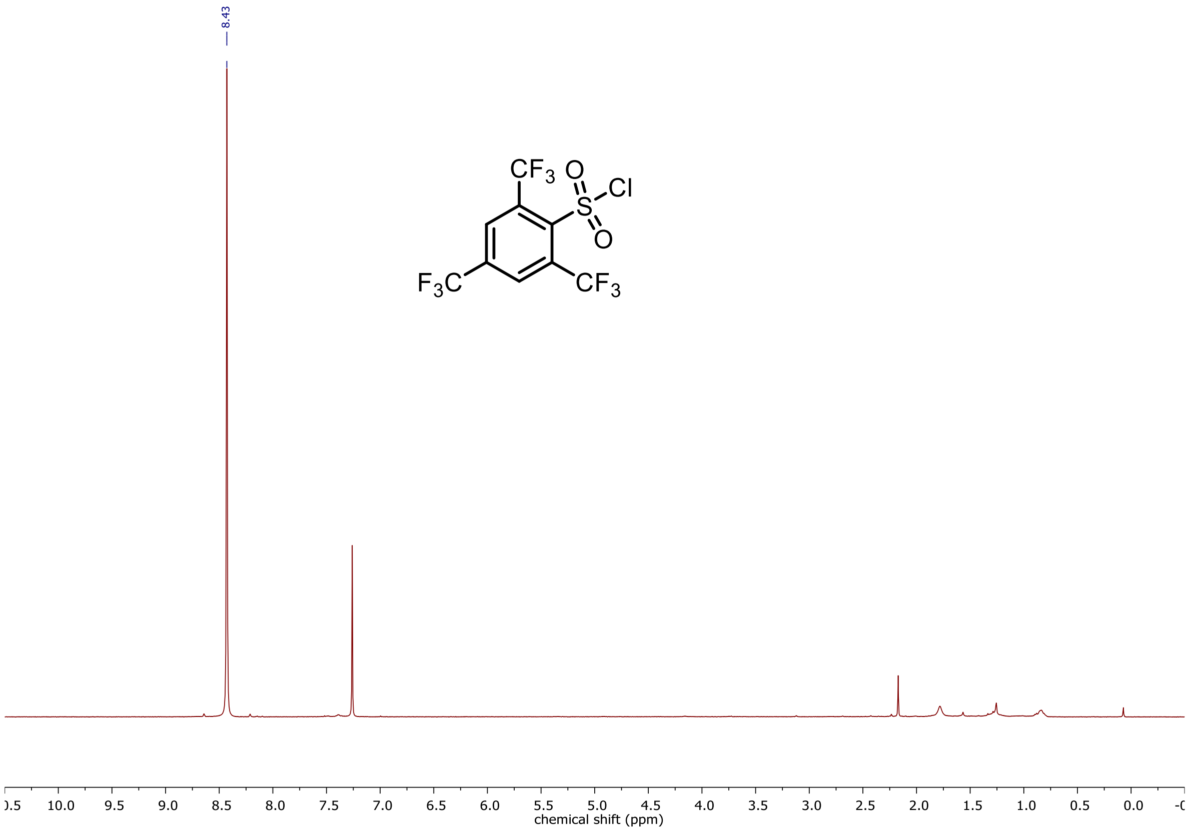

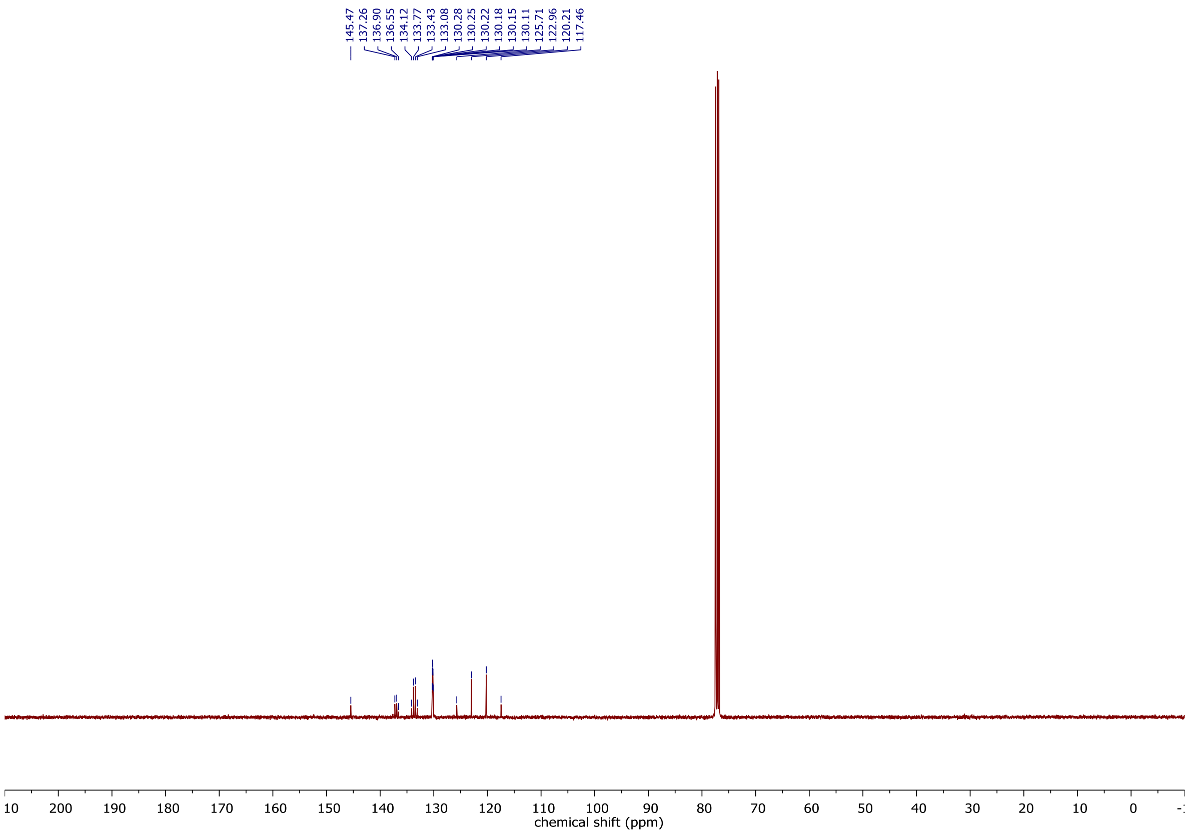

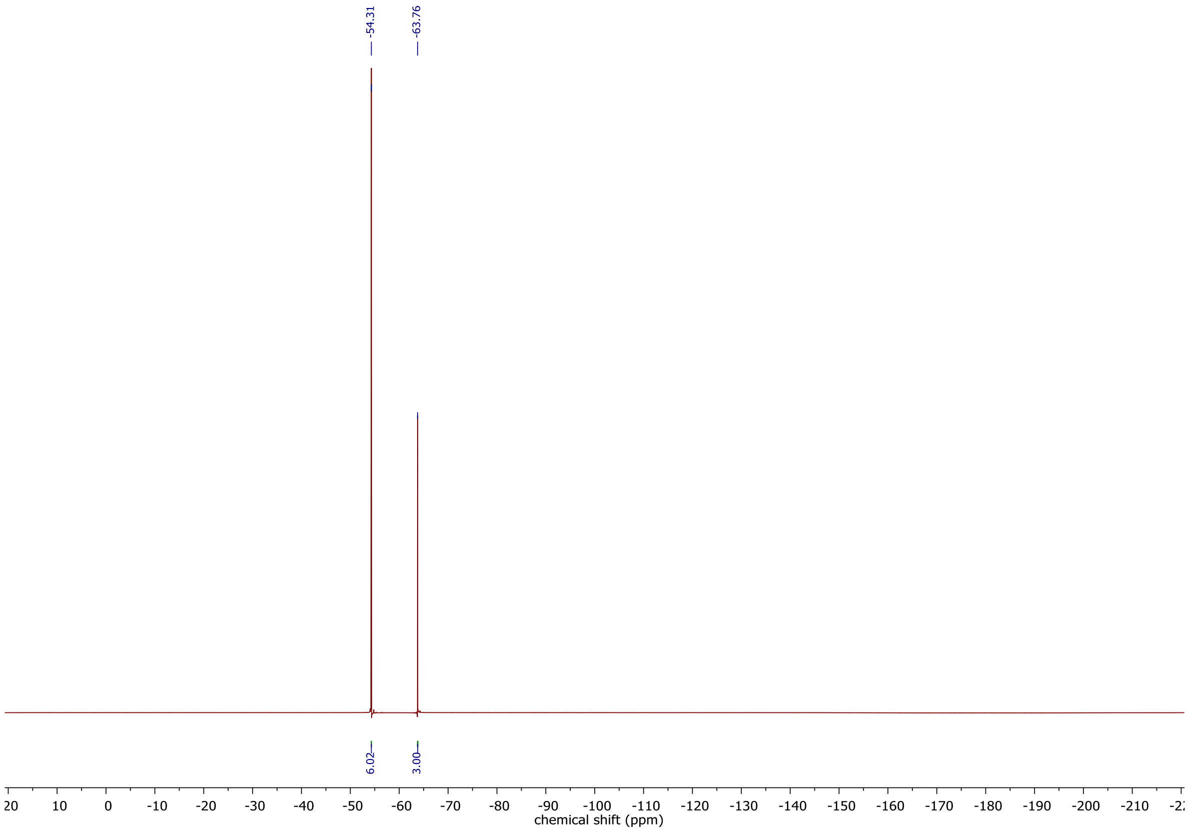

-54.31
-63.76
6.02
3.00
20
10
0
-10
-20
-30
-40
-50
-60
-70
-80
-90
-100
-110
-120
-130
-140
-150
-160
-170
-180
-190
-200
-210
chemical shift (ppm)

Butyl (*R*)-2-(4-(4-(aminomethyl)-2-fluorophenoxy)phenoxy)propanoate (1p)

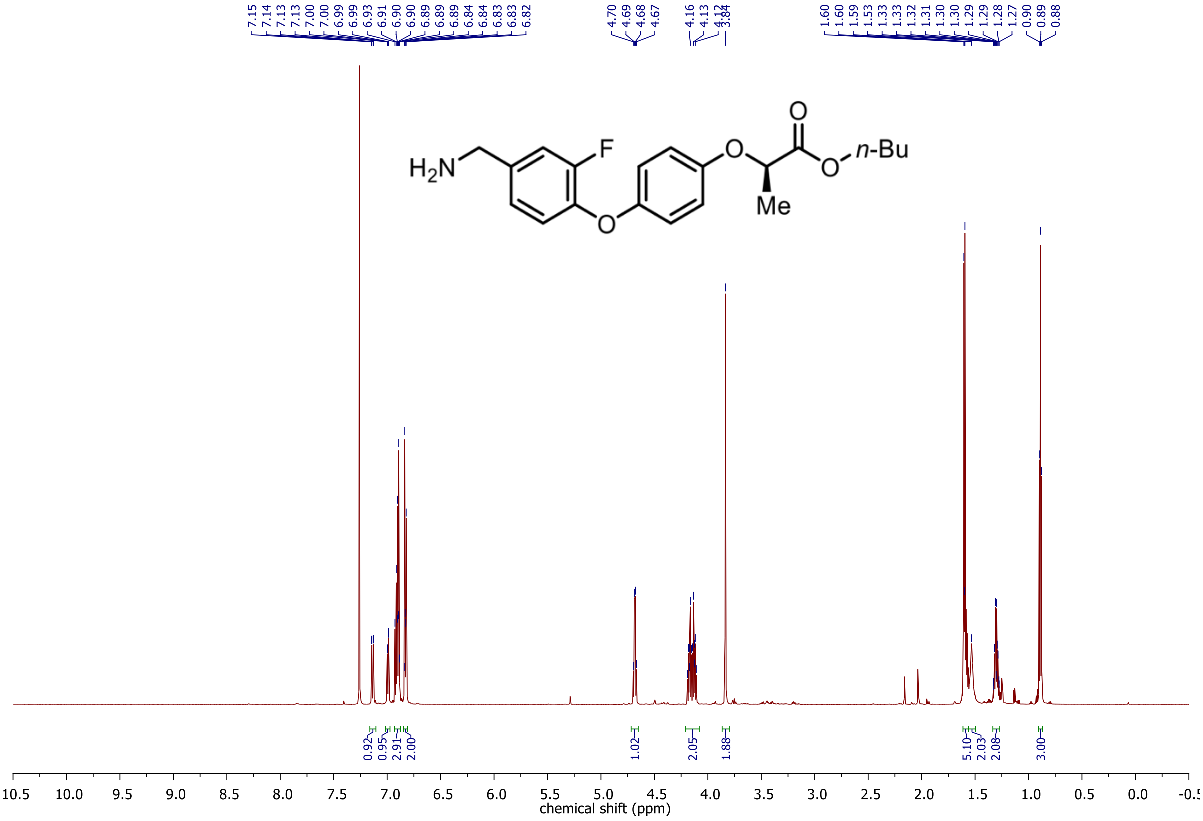

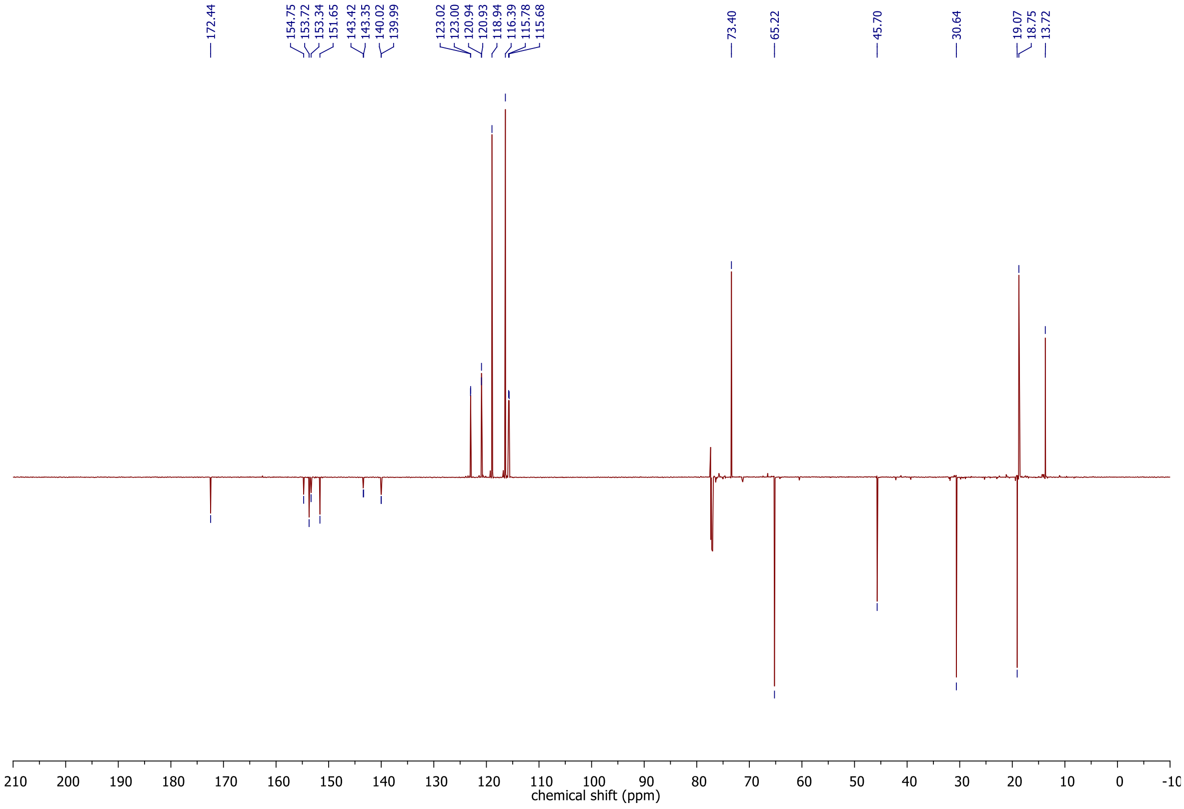

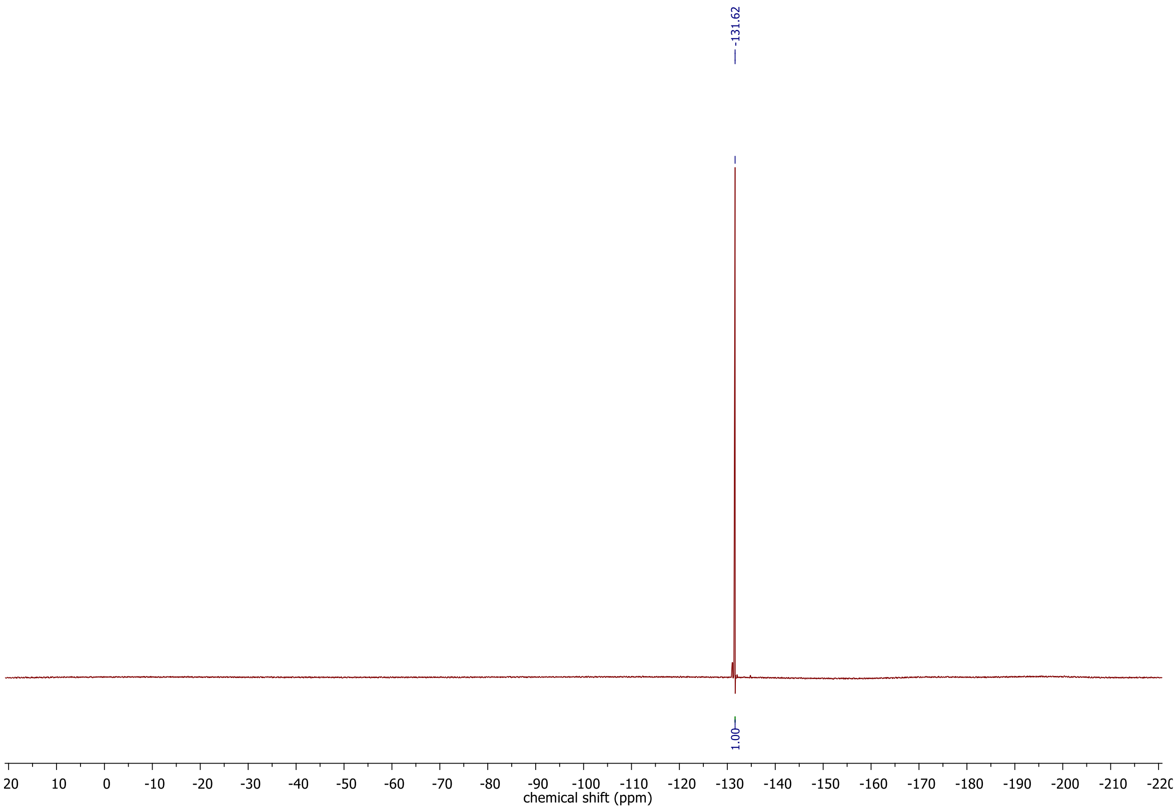

-131.62
1.00
20 10 0 -10 -20 -30 -40 -50 -60 -70 -80 -90 -100 -110 -120 -130 -140 -150 -160 -170 -180 -190 -200 -210 -220
chemical shift (ppm)

2,4,6-tris(Trifluoromethyl)benzenesulfonamide (6)

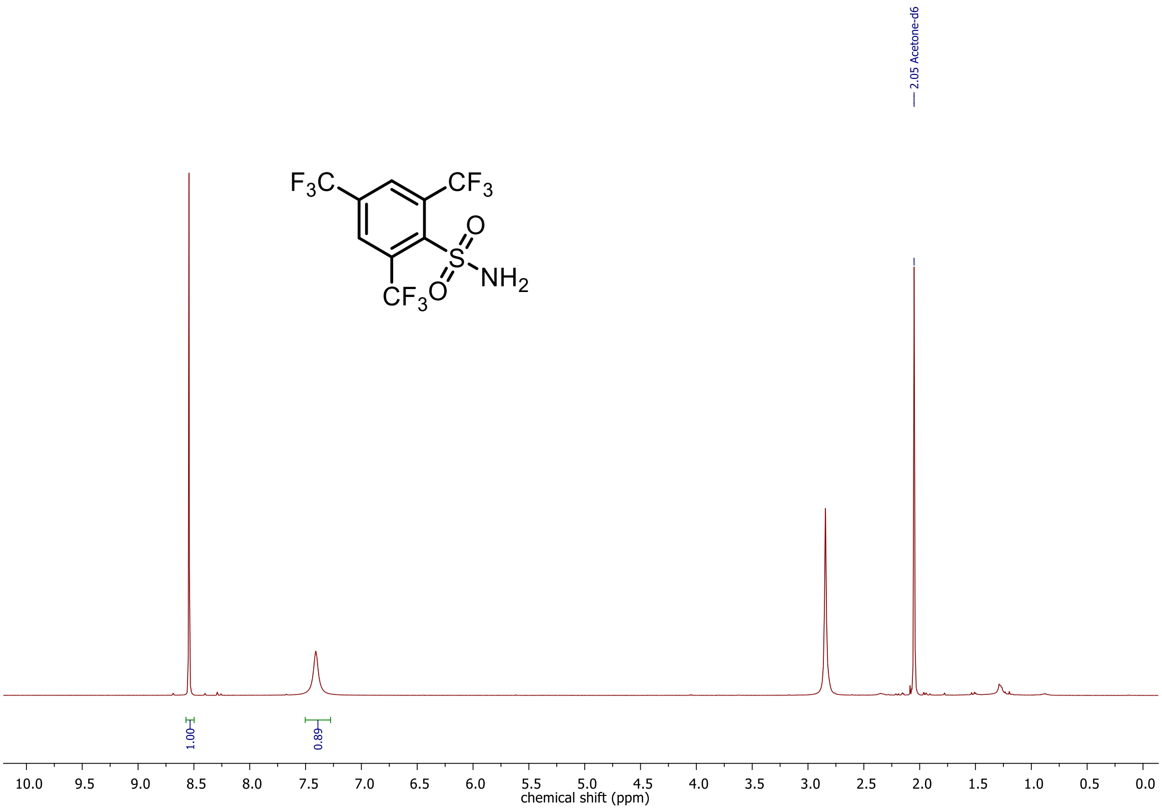

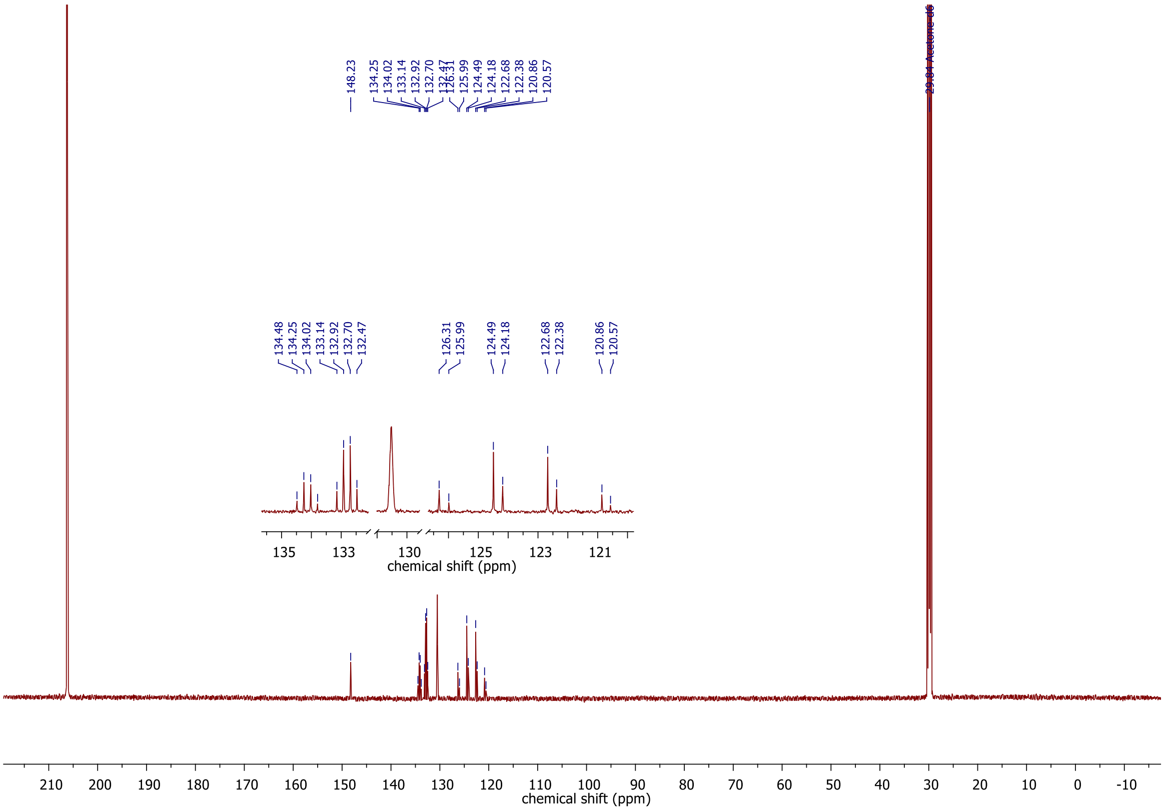

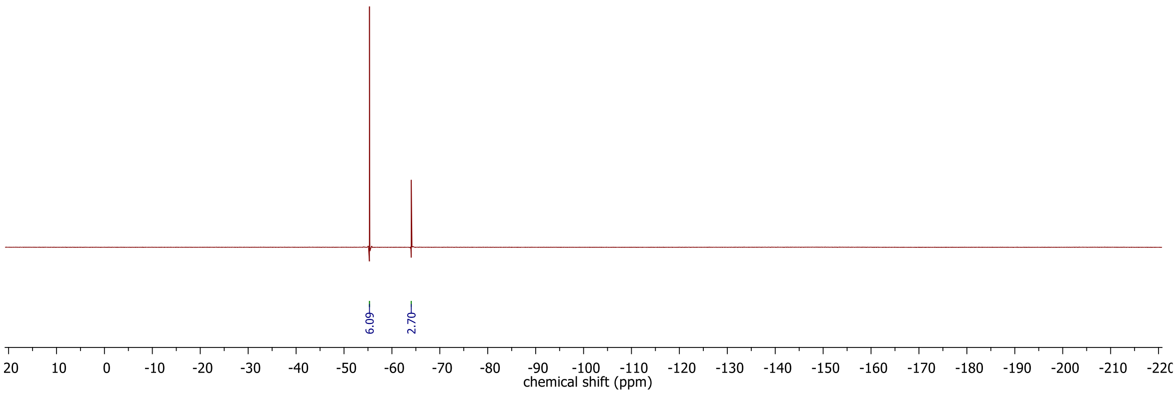
6.09
2.70
20 10 0 -10 -20 -30 -40 -50 -60 -70 -80 -90 -100 -110 -120 -130 -140 -150 -160 -170 -180 -190 -200 -210 -220
chemical shift (ppm)

***N*-(Cyclohexyl)-2,4,6-tris(trifluoromethyl)benzenesulfonamide (2a)**

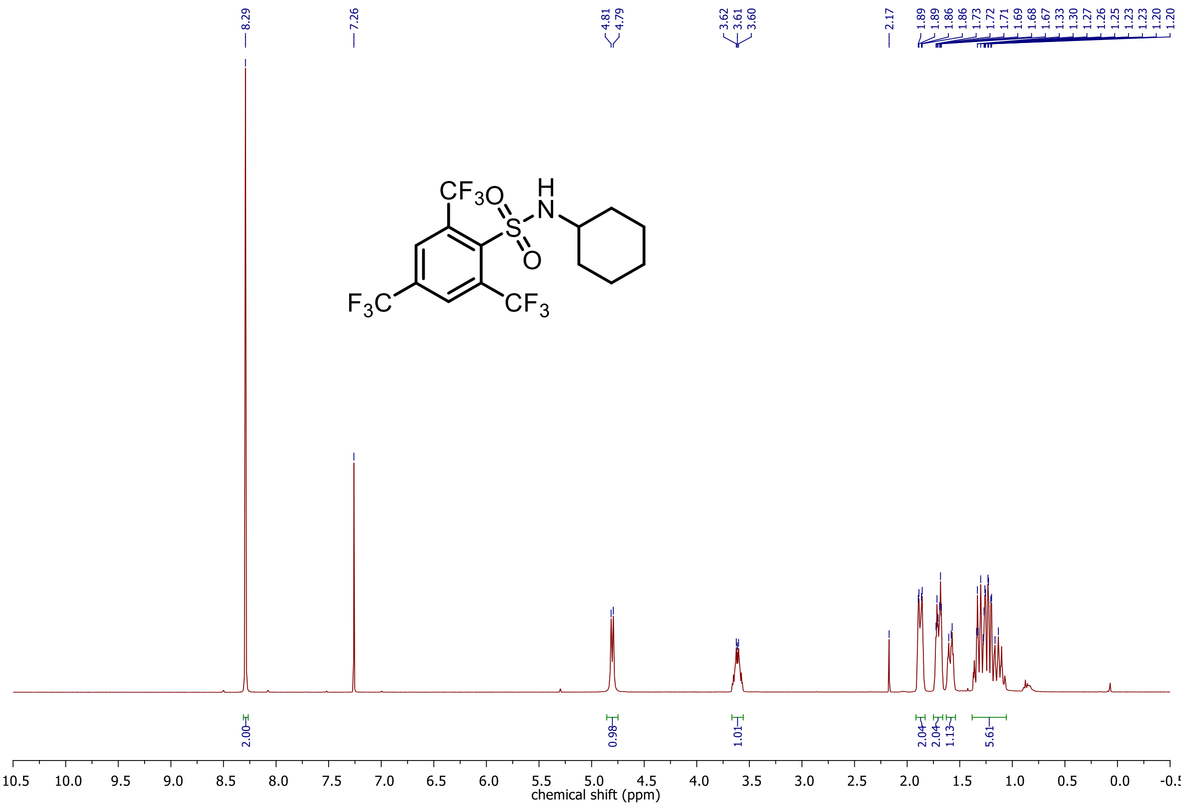

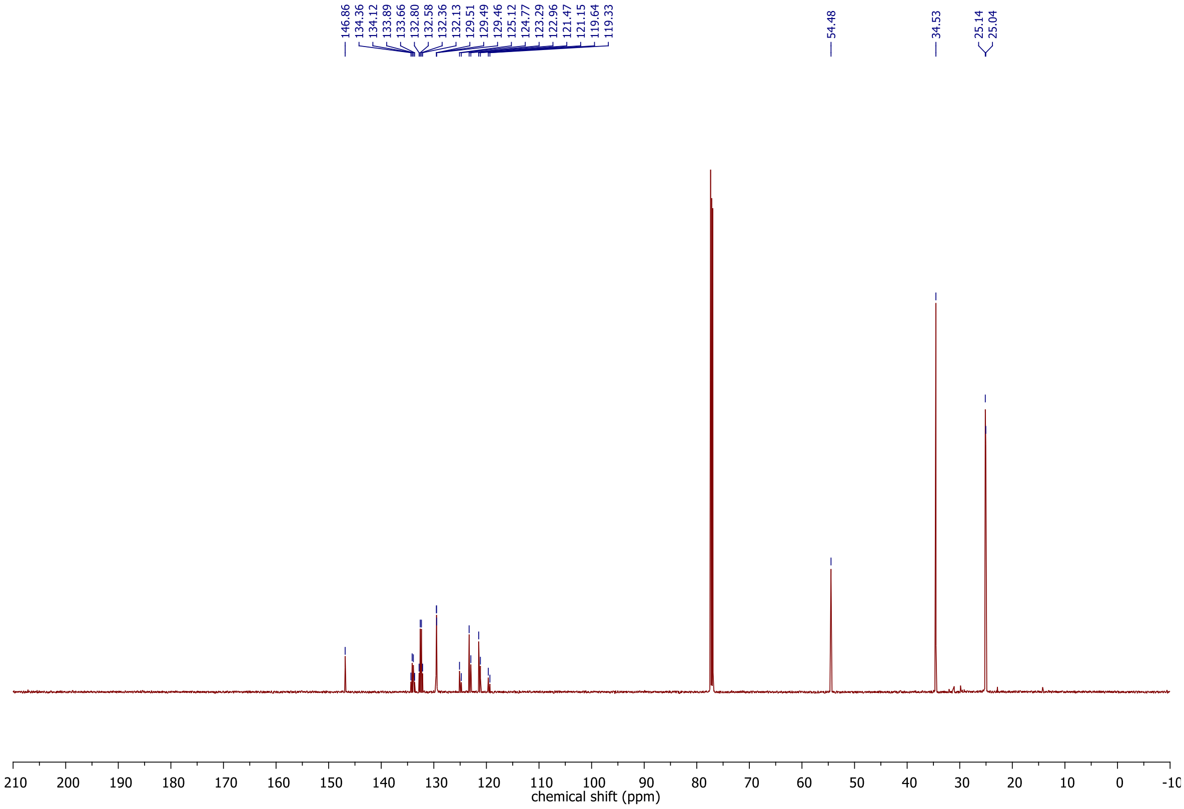

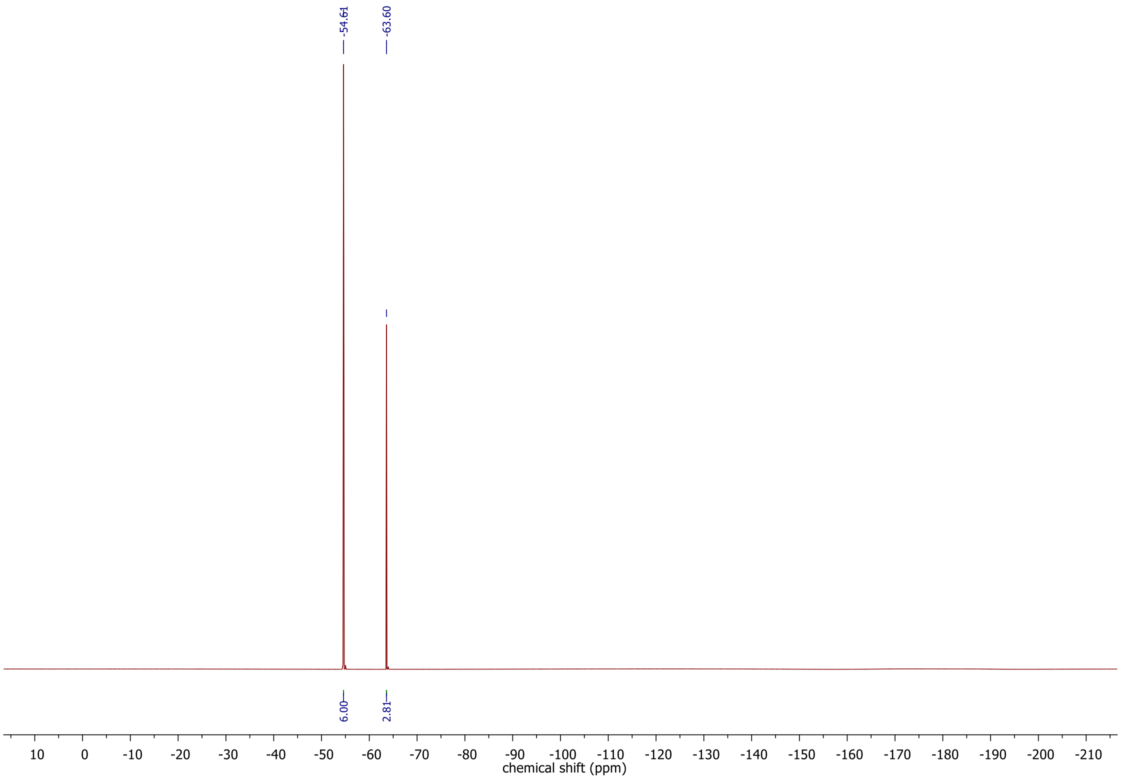
-54.61
-63.60
6.00
2.81
10
0
-10
-20
-30
-40
-50
-60
-70
-80
-90
-100
-110
-120
-130
-140
-150
-160
-170
-180
-190
-200
-210
chemical shift (ppm)

N-Pentyl-2,4,6-tris(trifluoromethyl)benzenesulfonamide (2b)

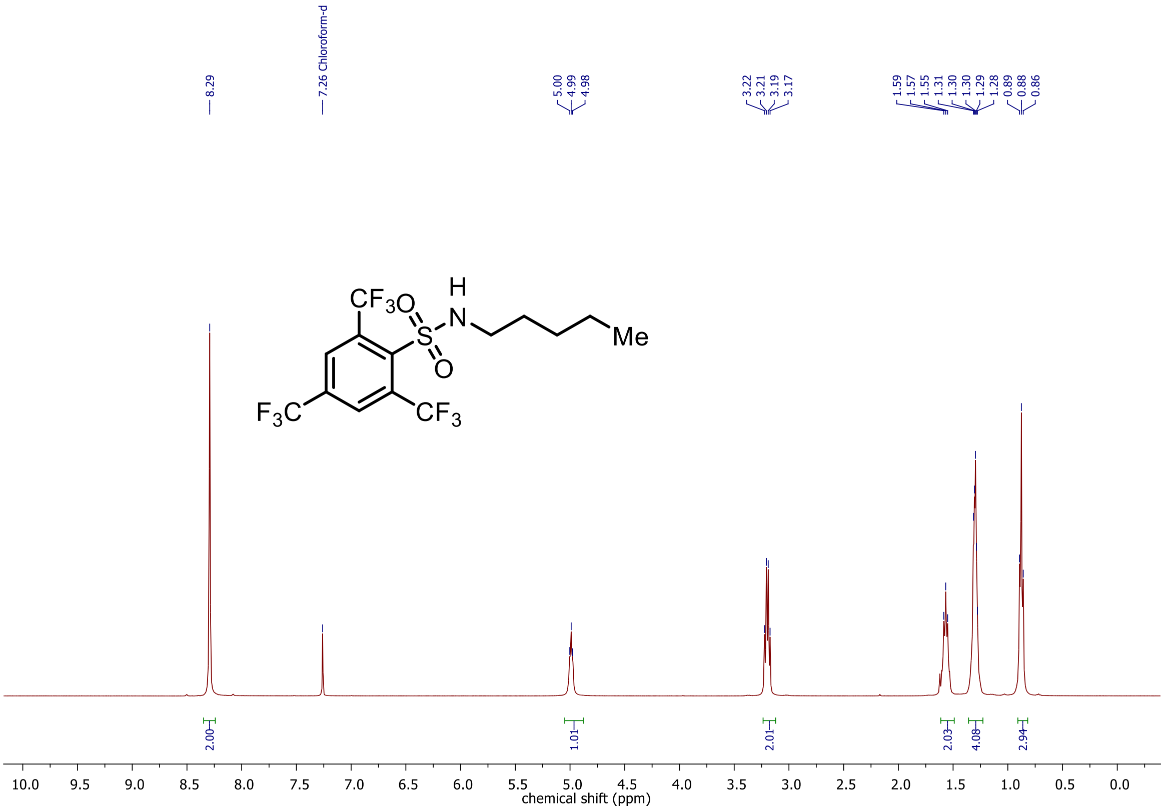

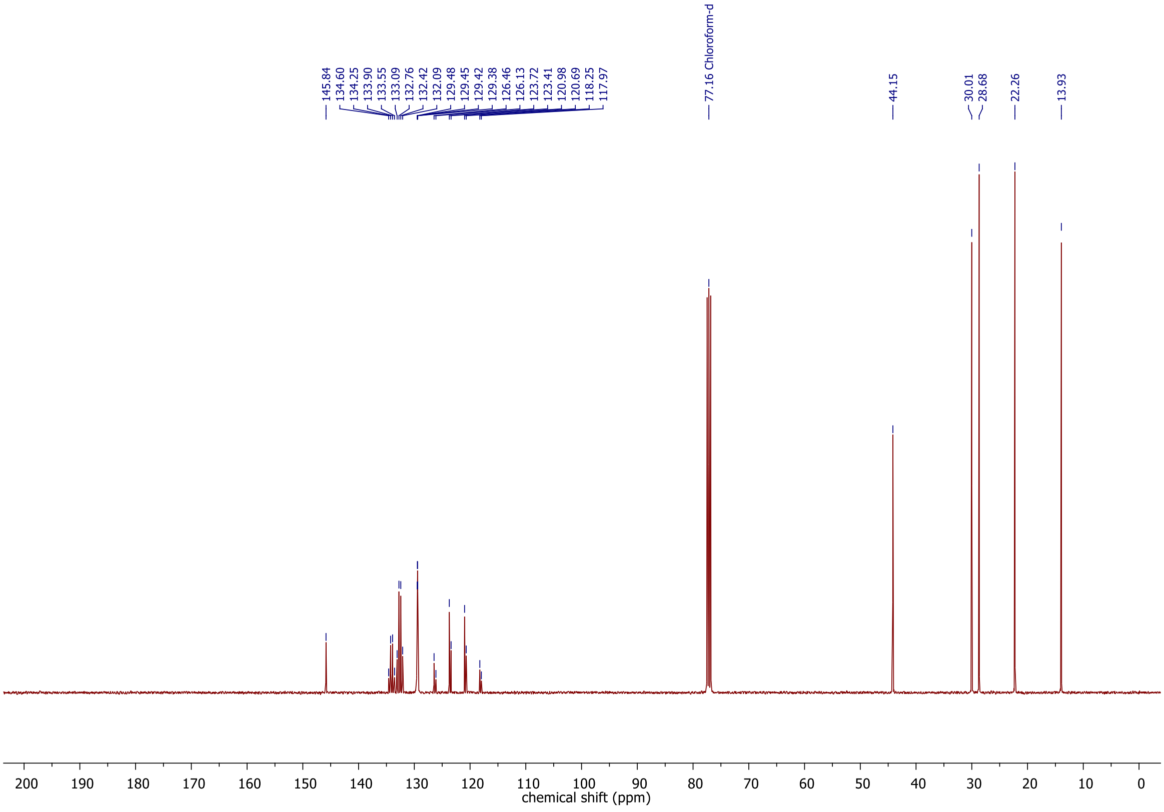

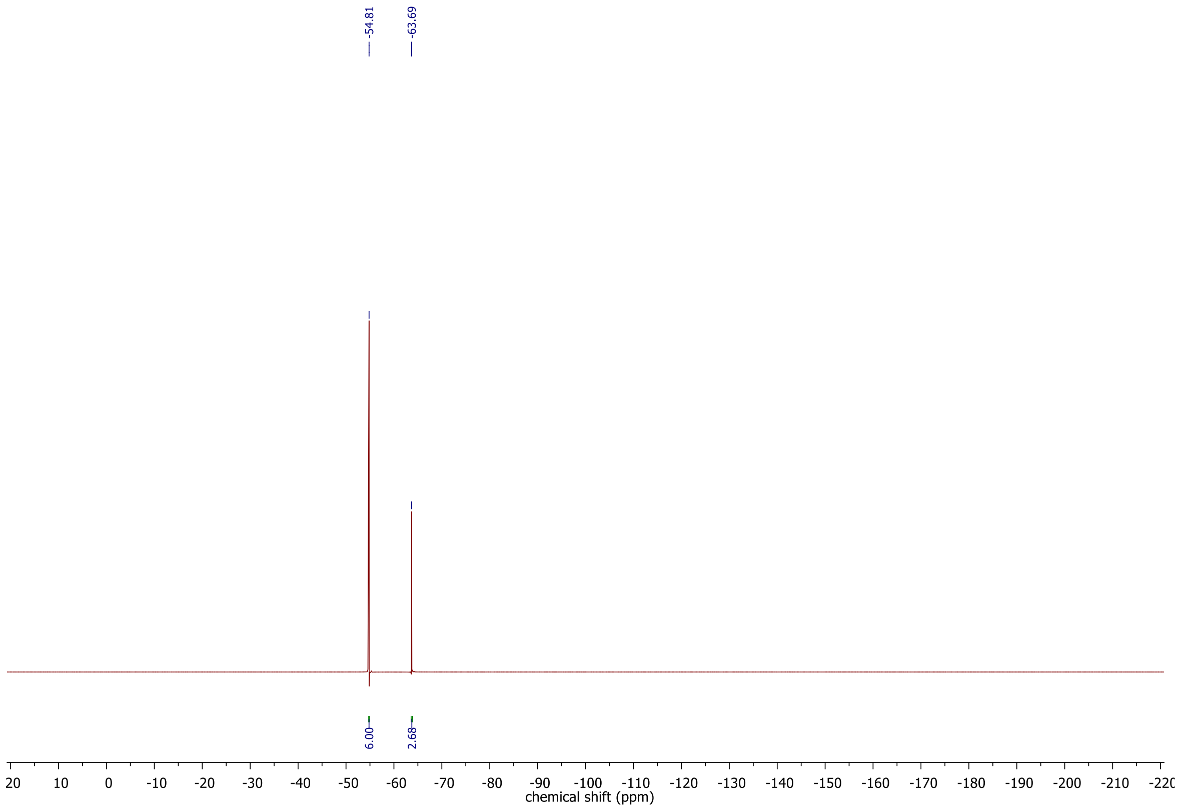

-54.81
-63.69
6.00
2.68
20 10 0 -10 -20 -30 -40 -50 -60 -70 -80 -90 -100 -110 -120 -130 -140 -150 -160 -170 -180 -190 -200 -210 -220
chemical shift (ppm)

N-(But-3-en-1-yl)-2,4,6-tris(trifluoromethyl)benzenesulfonamide (2c)

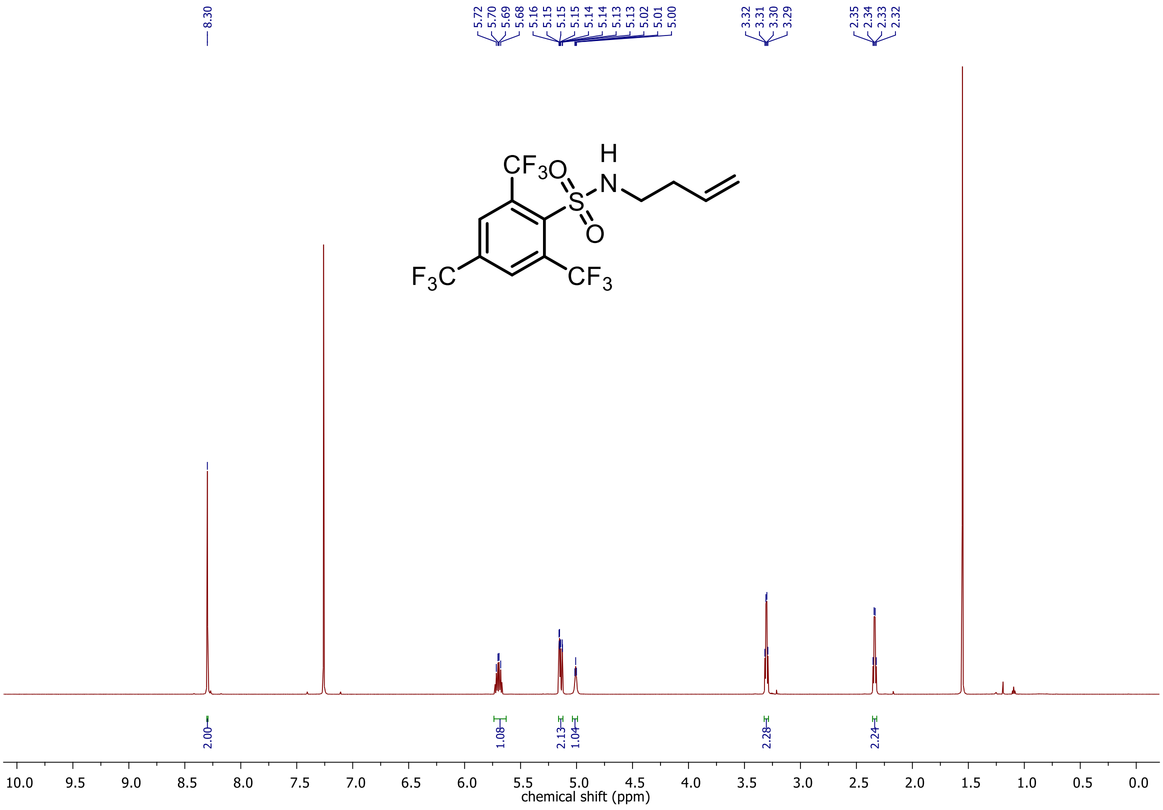

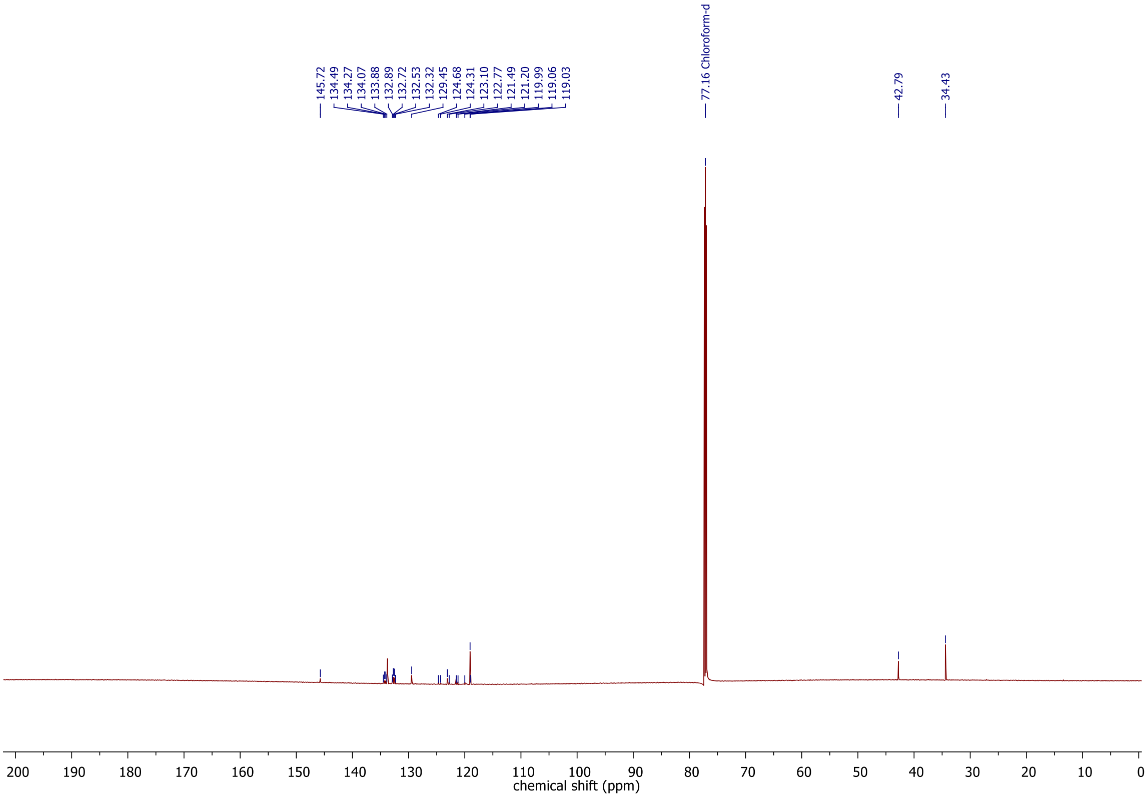

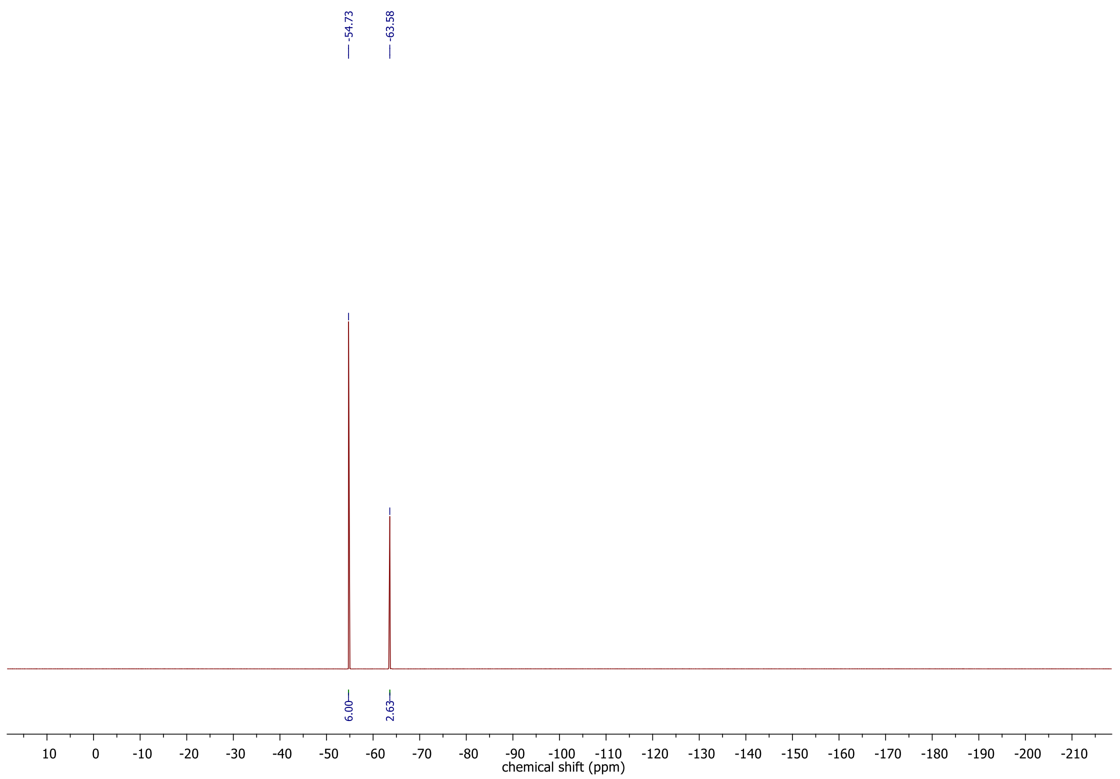
-54.73
-63.58
6.00
2.63
10 0 -10 -20 -30 -40 -50 -60 -70 -80 -90 -100 -110 -120 -130 -140 -150 -160 -170 -180 -190 -200 -210
chemical shift (ppm)

N-(((1S,2R,5S)-6,6-Dimethylbicyclo[3.1.1]heptan-2-yl)methyl)-2,4,6-tris(trifluoromethyl)benzenesulfonamide (2d)

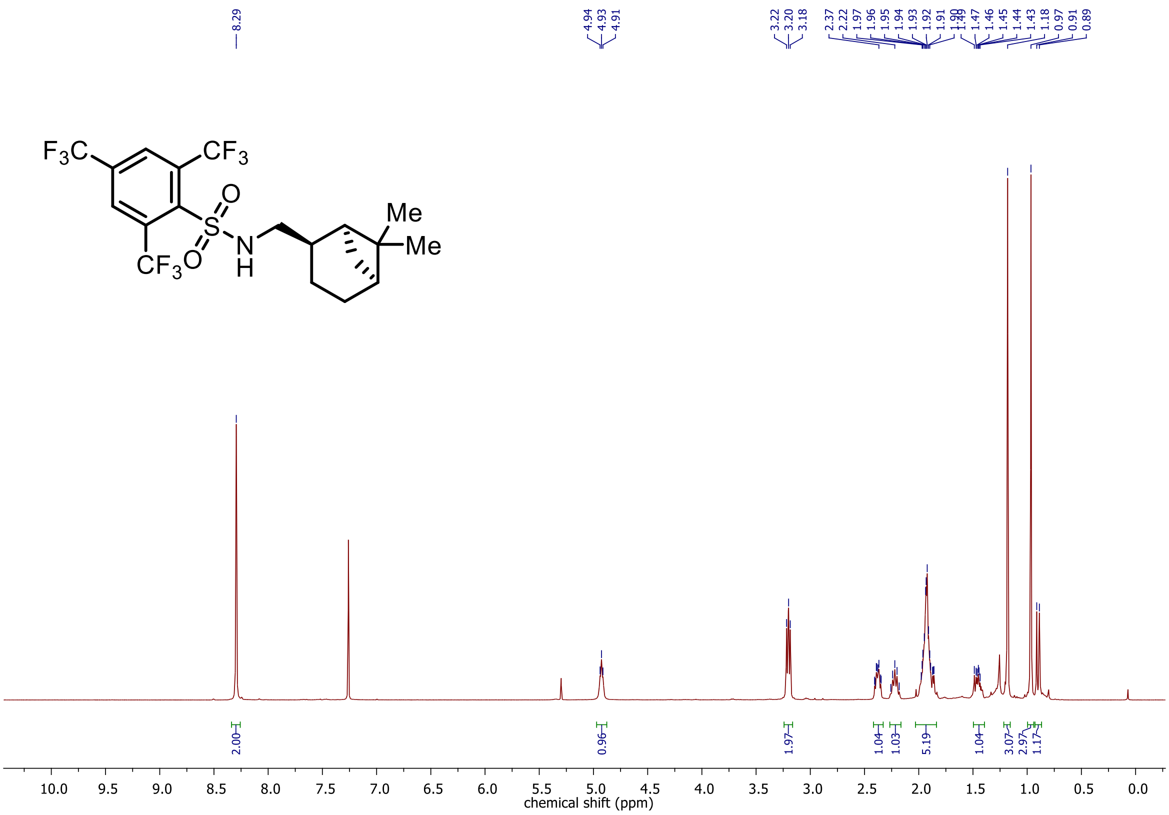

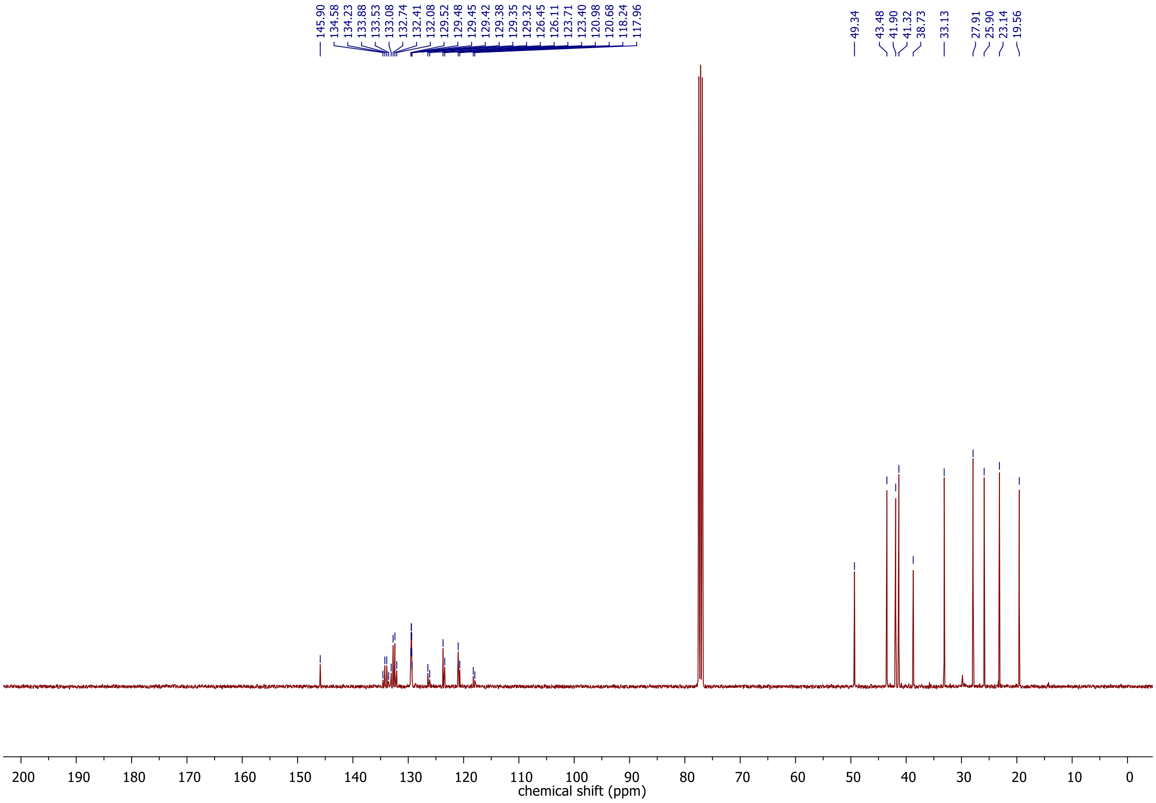

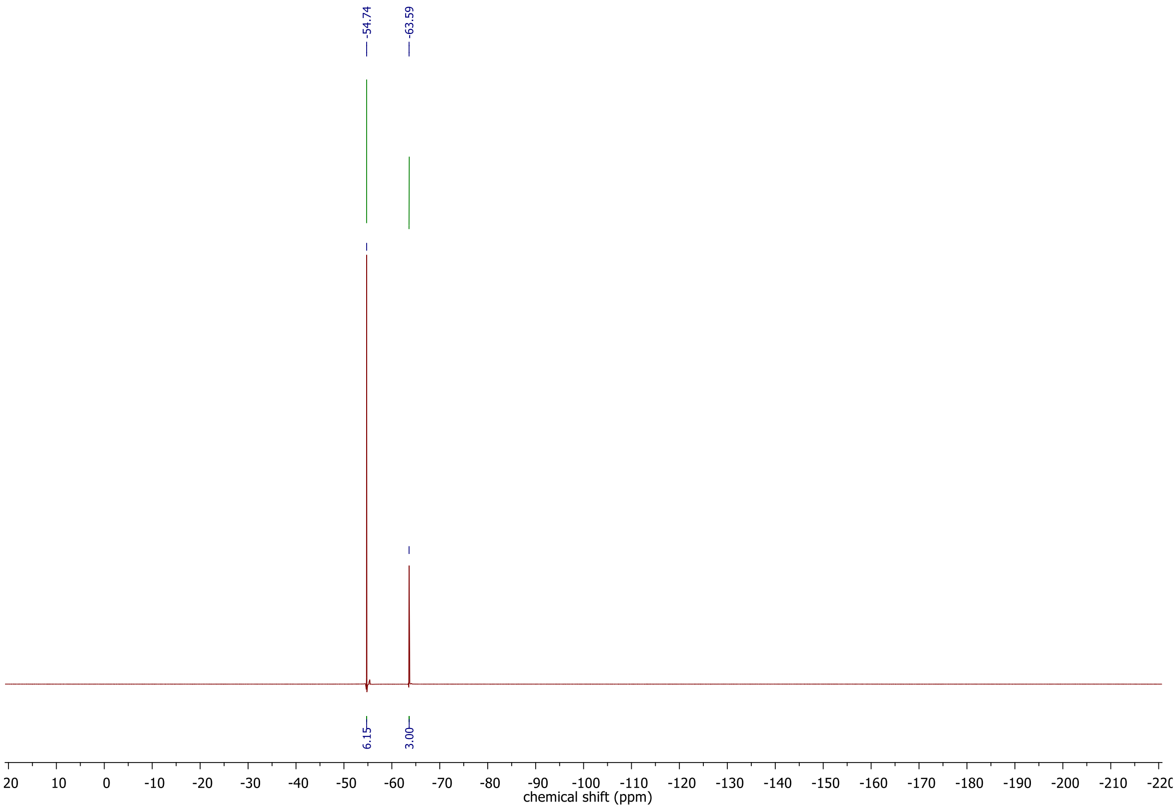
-54.74
-63.59
6.15
3.00
20 10 0 -10 -20 -30 -40 -50 -60 -70 -80 -90 -100 -110 -120 -130 -140 -150 -160 -170 -180 -190 -200 -210 -220
chemical shift (ppm)

_N_-(3,4-Dimethoxyphenethyl)-2,4,6-tris(trifluoromethyl)benzenesulfonamide (2e)

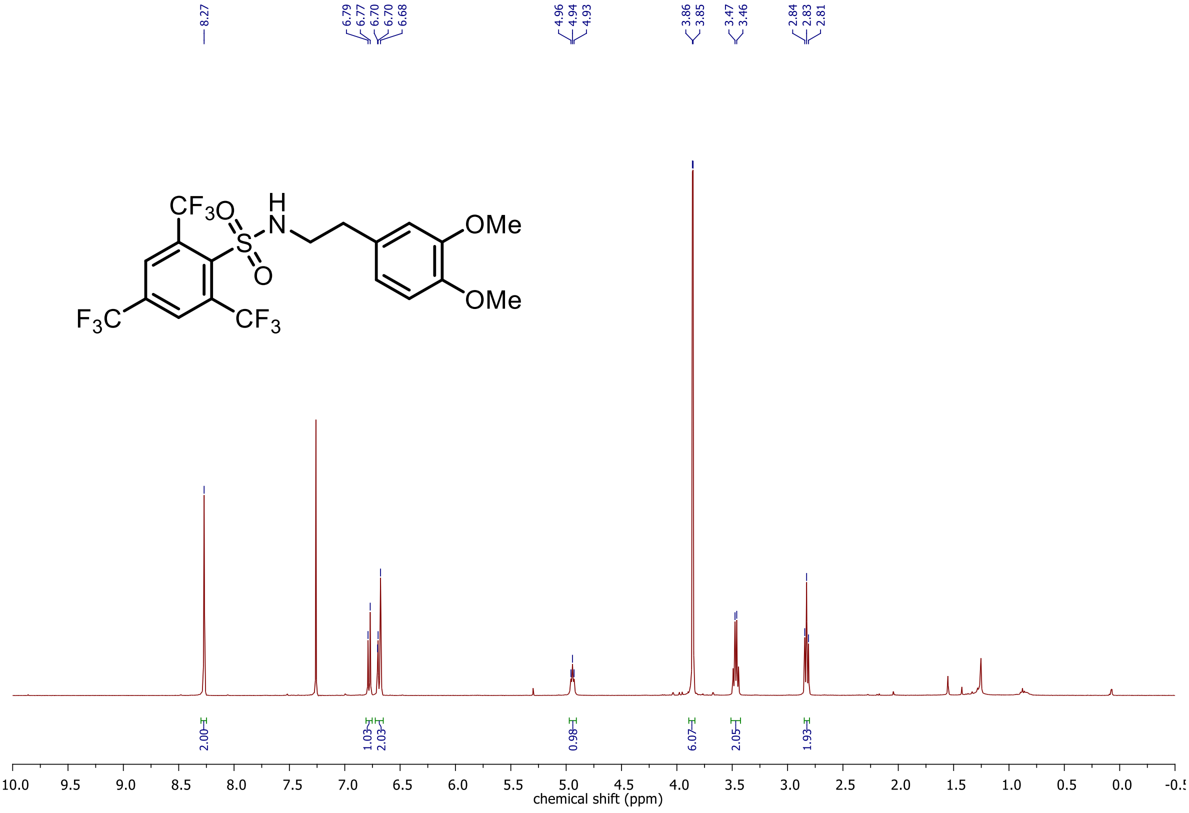

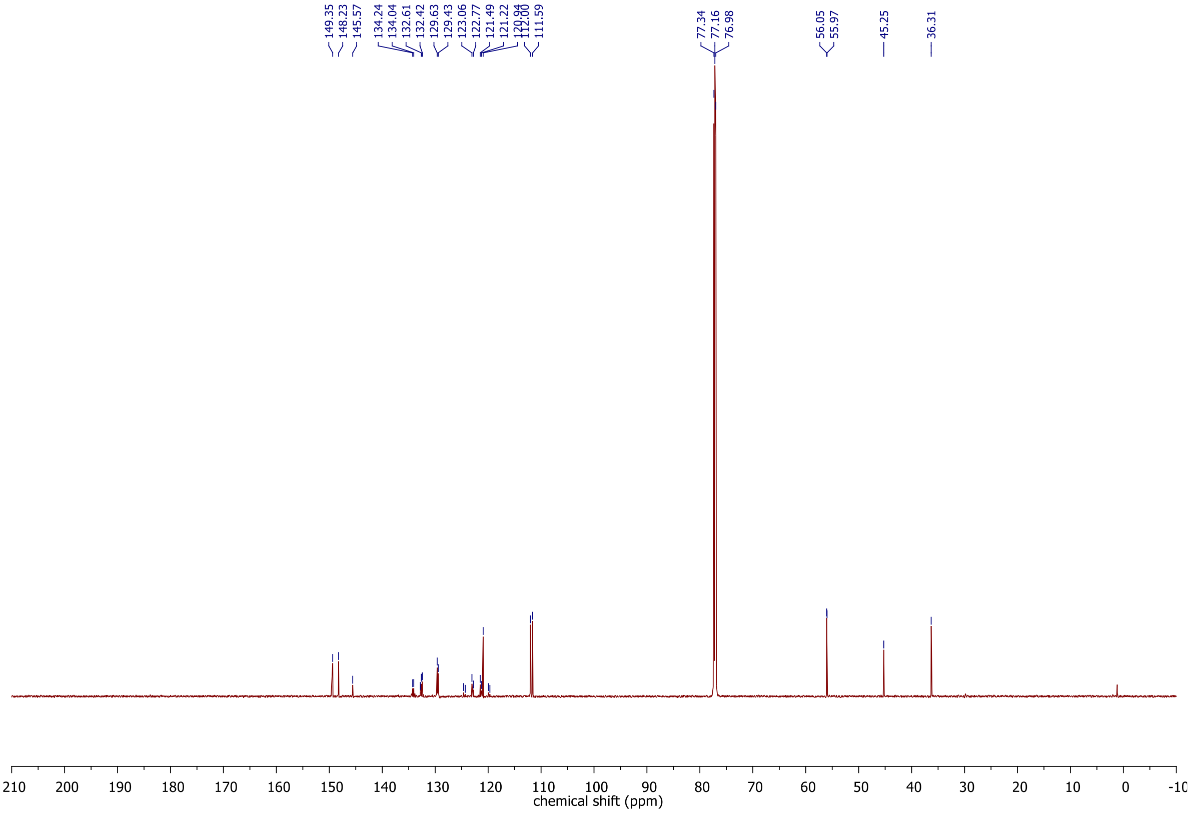

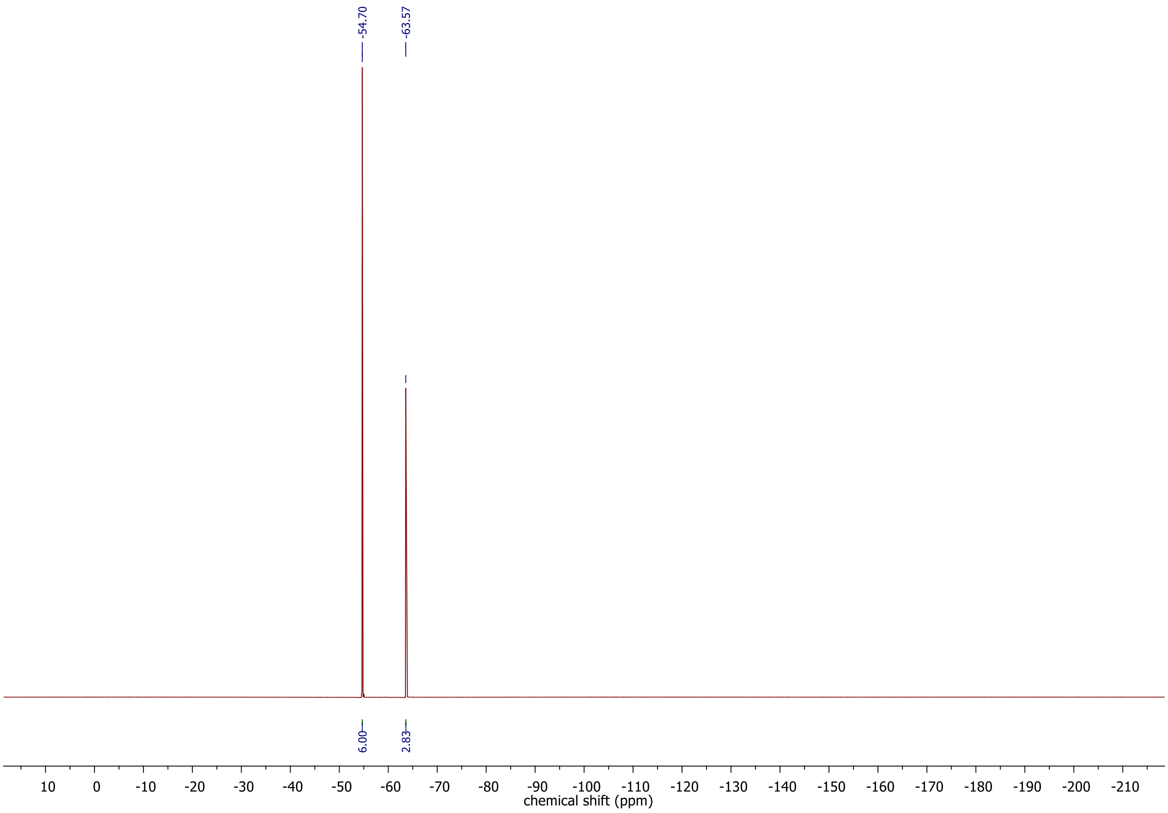

-54.70
-63.57
6.00
2.83
10
0
-10
-20
-30
-40
-50
-60
-70
-80
-90
-100
-110
-120
-130
-140
-150
-160
-170
-180
-190
-200
-210
chemical shift (ppm)

***N*-[3-(2-Methylpiperidin-1-yl)propyl]-2,4,6-tris(trifluoromethyl)benzenesulfonamide (2f)**

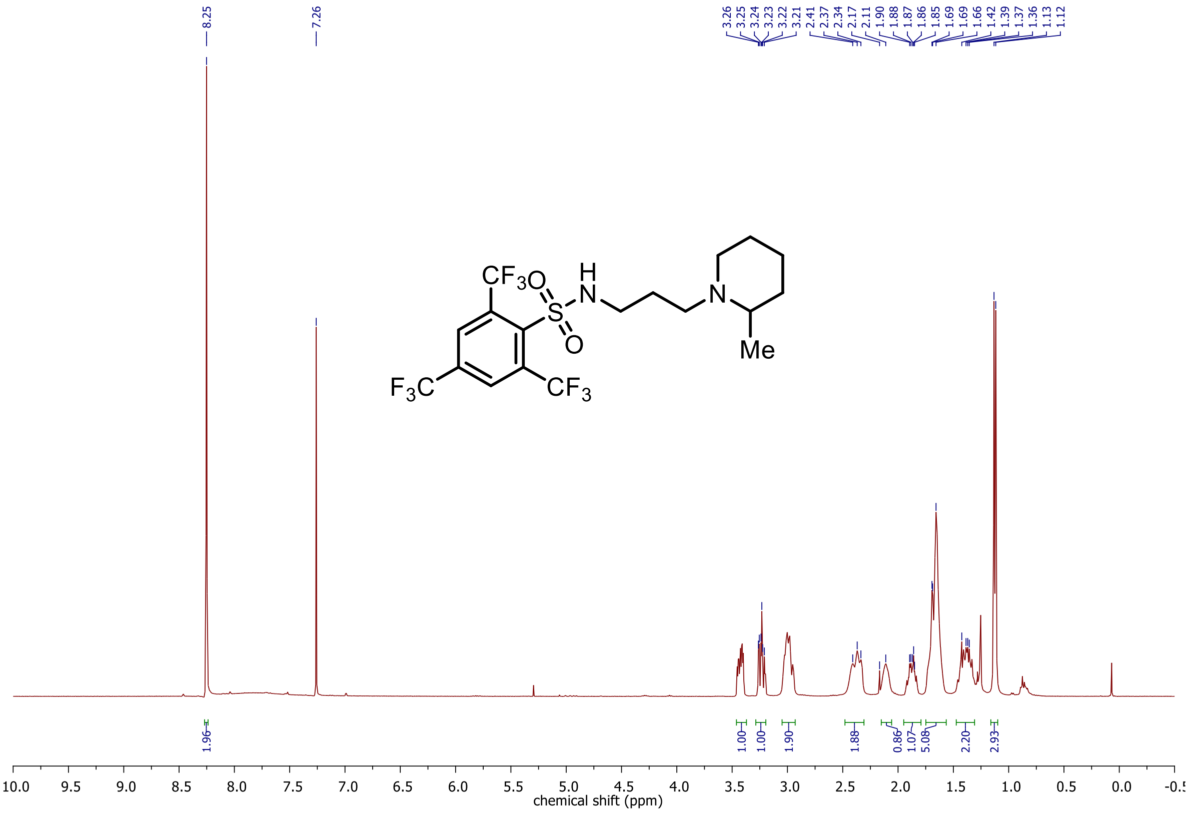

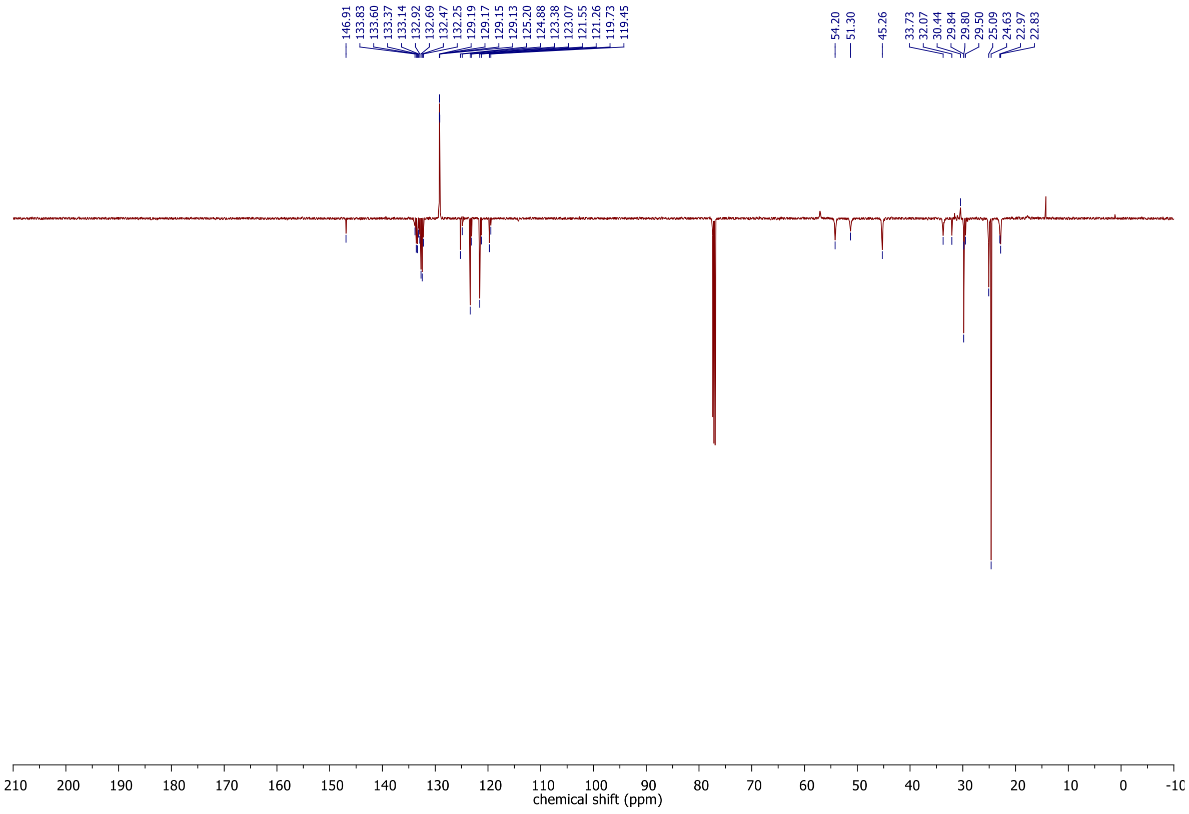

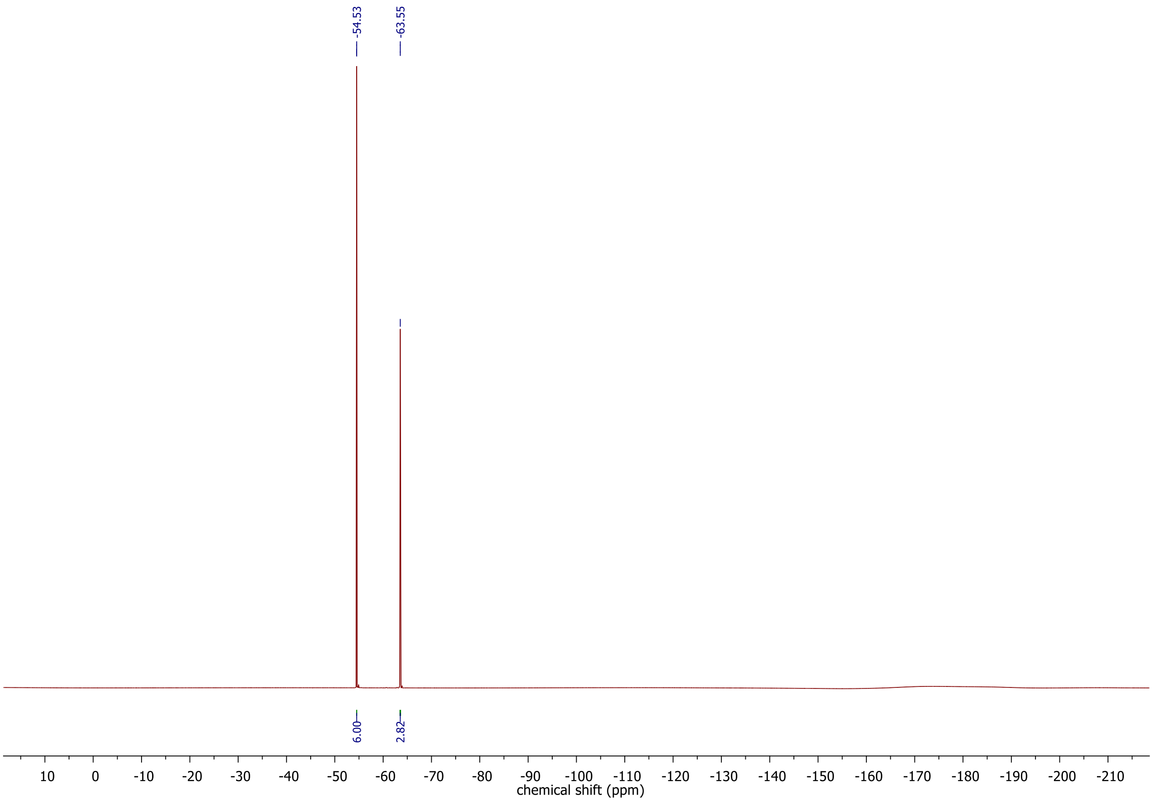

-54.53
-63.55
6.00
2.82
10 0 -10 -20 -30 -40 -50 -60 -70 -80 -90 -100 -110 -120 -130 -140 -150 -160 -170 -180 -190 -200 -210
chemical shift (ppm)

tert-Butyl [3-((2,4,6-tris(trifluoromethyl)phenyl)sulfonamido)propyl]carbamate (2g)

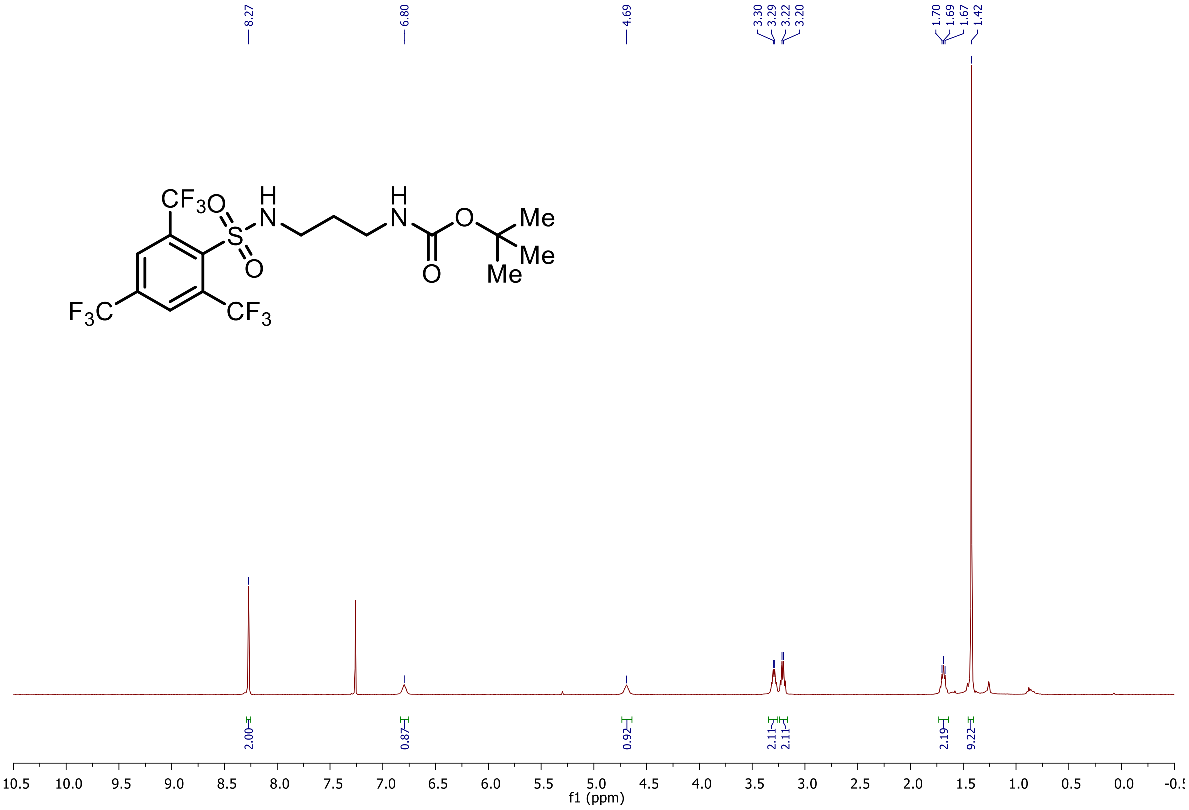

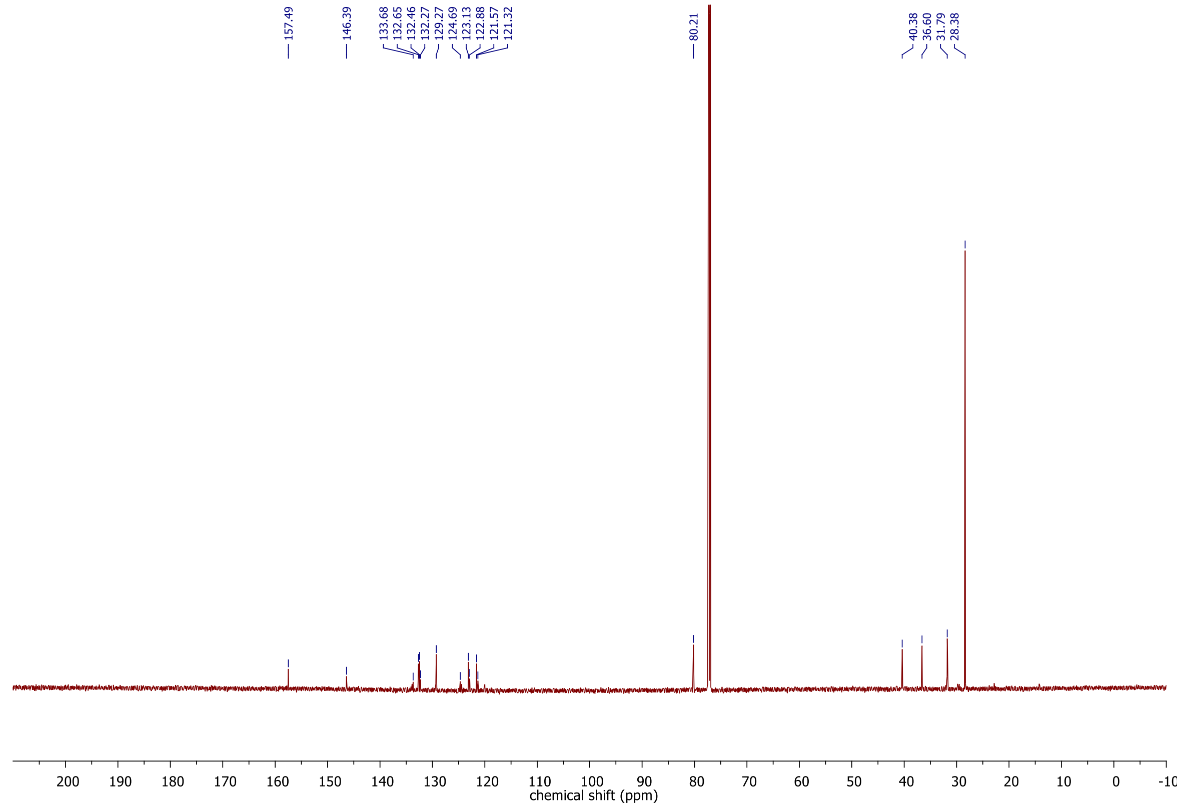

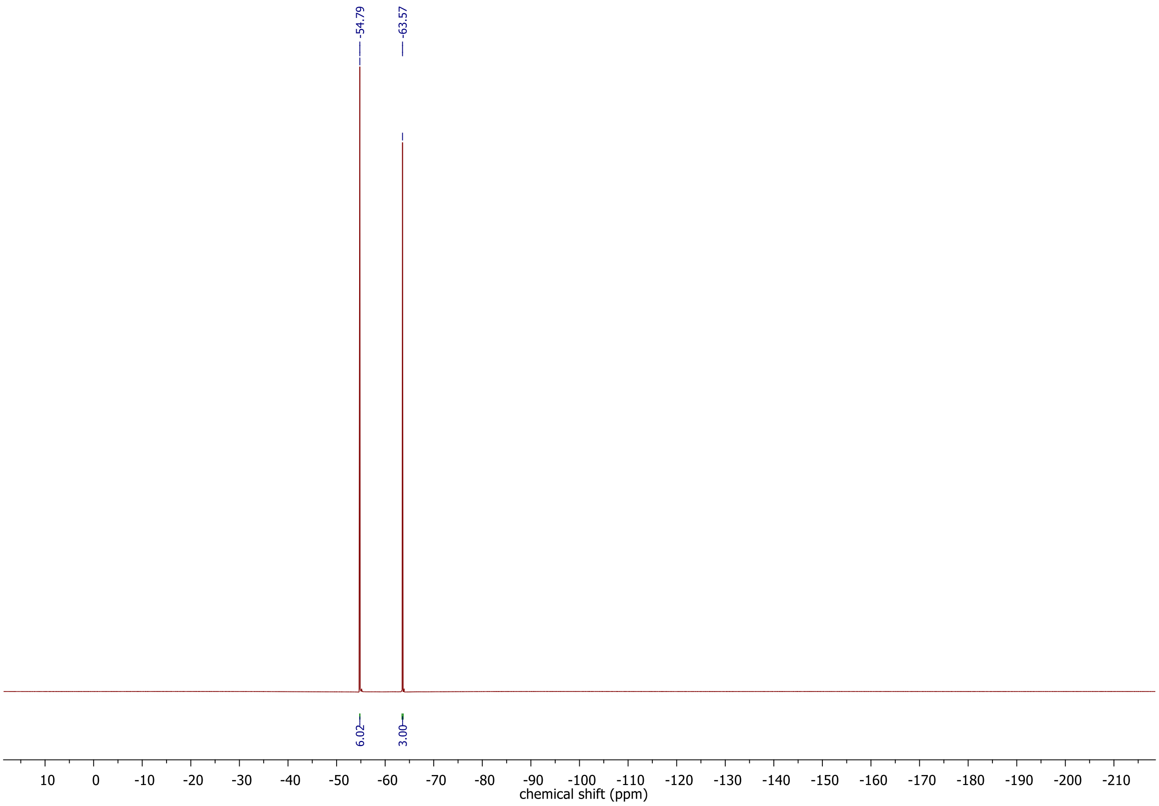

-54.79
-63.57
6.02
3.00
10 0 -10 -20 -30 -40 -50 -60 -70 -80 -90 -100 -110 -120 -130 -140 -150 -160 -170 -180 -190 -200 -210
chemical shift (ppm)

_N_-[2-(1_H_-Indol-3-yl)ethyl]-2,4,6-tris(trifluoromethyl)benzenesulfonamide (2h)

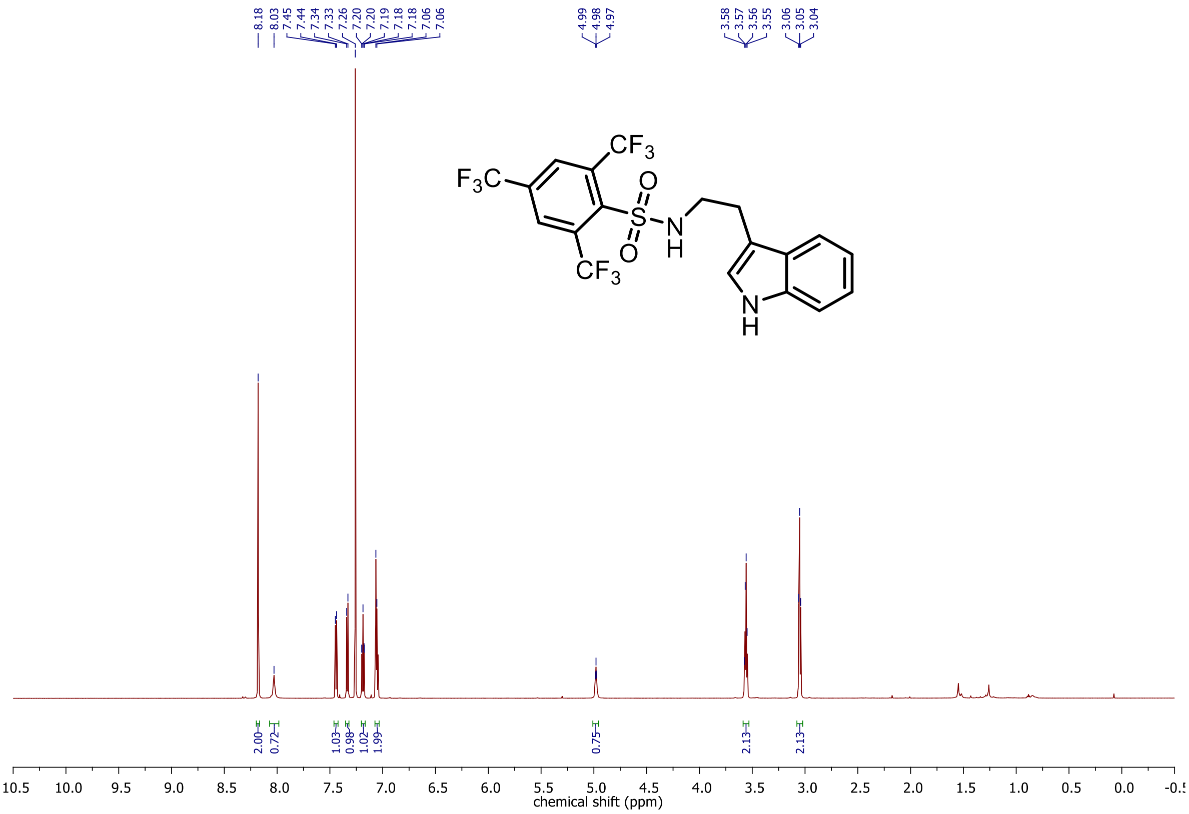

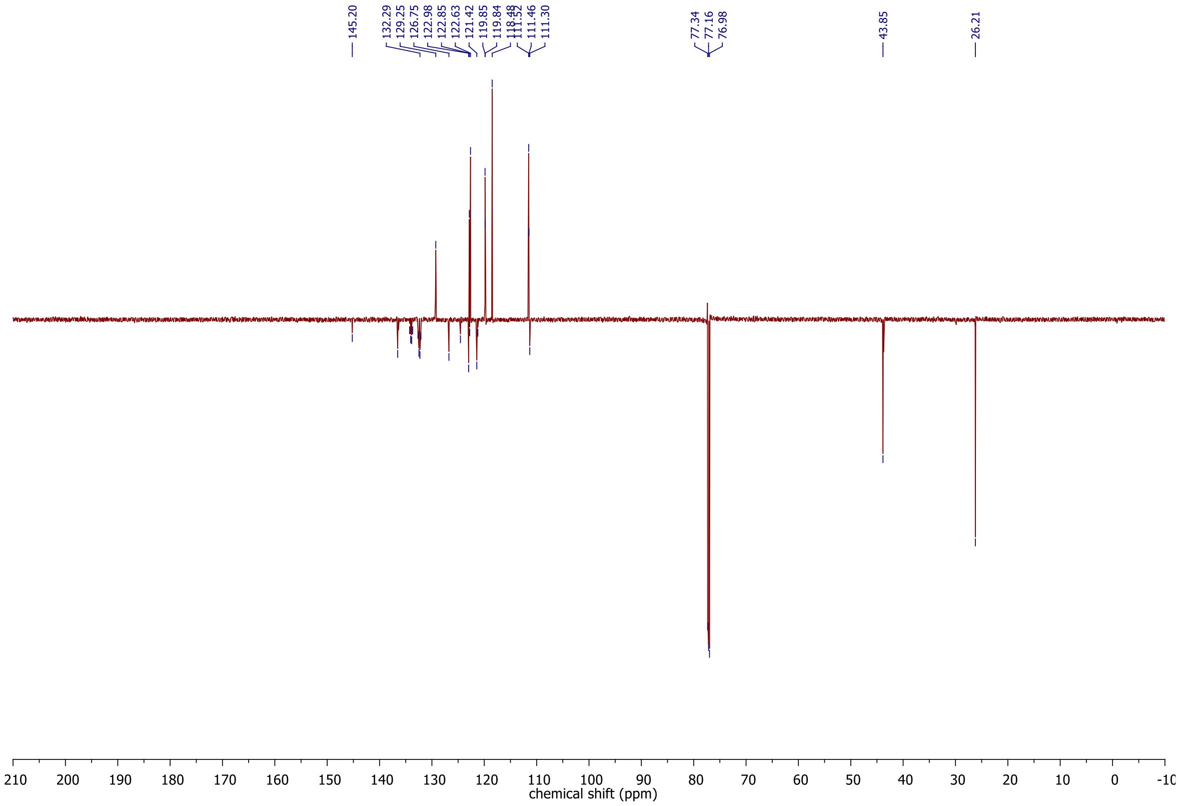

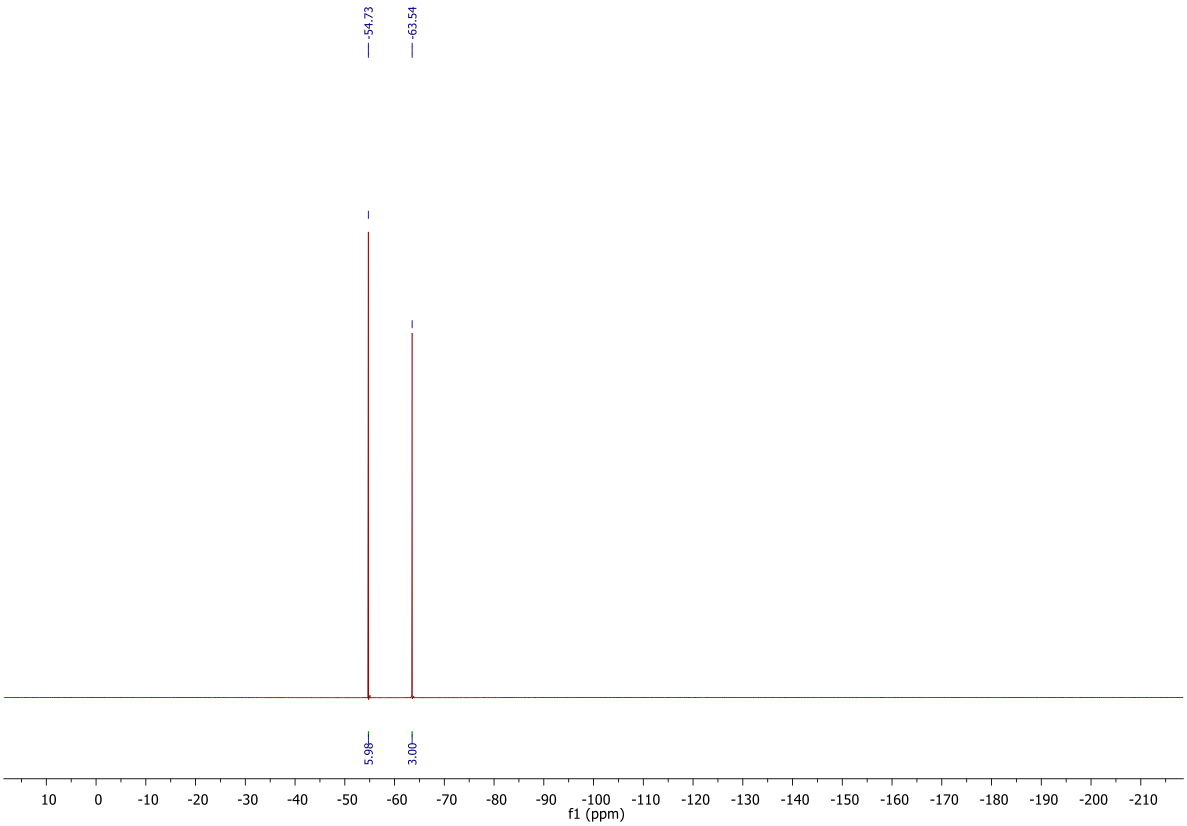

-54.73
-63.54
5.98
3.00
10 0 -10 -20 -30 -40 -50 -60 -70 -80 -90 -100 -110 -120 -130 -140 -150 -160 -170 -180 -190 -200 -210
f1 (ppm)

N-(Pyridin-2-ylmethyl)-2,4,6-tris(trifluoromethyl)benzenesulfonamide (2i)

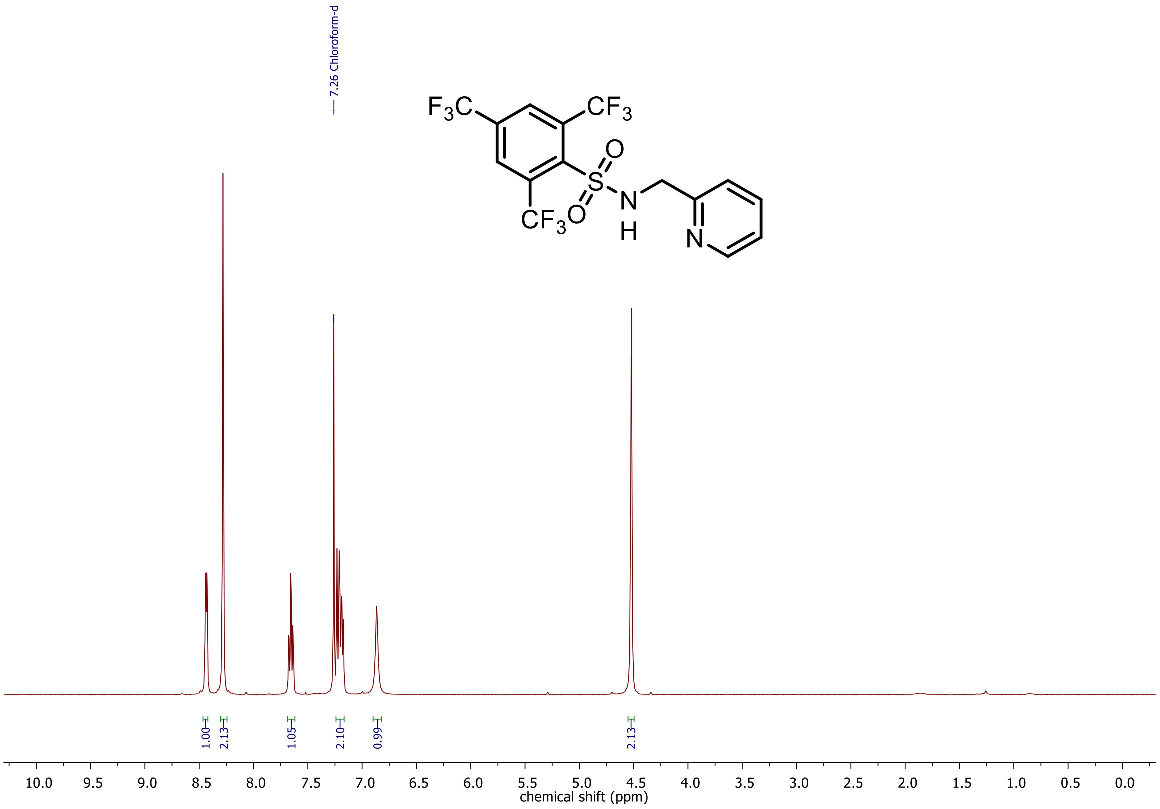

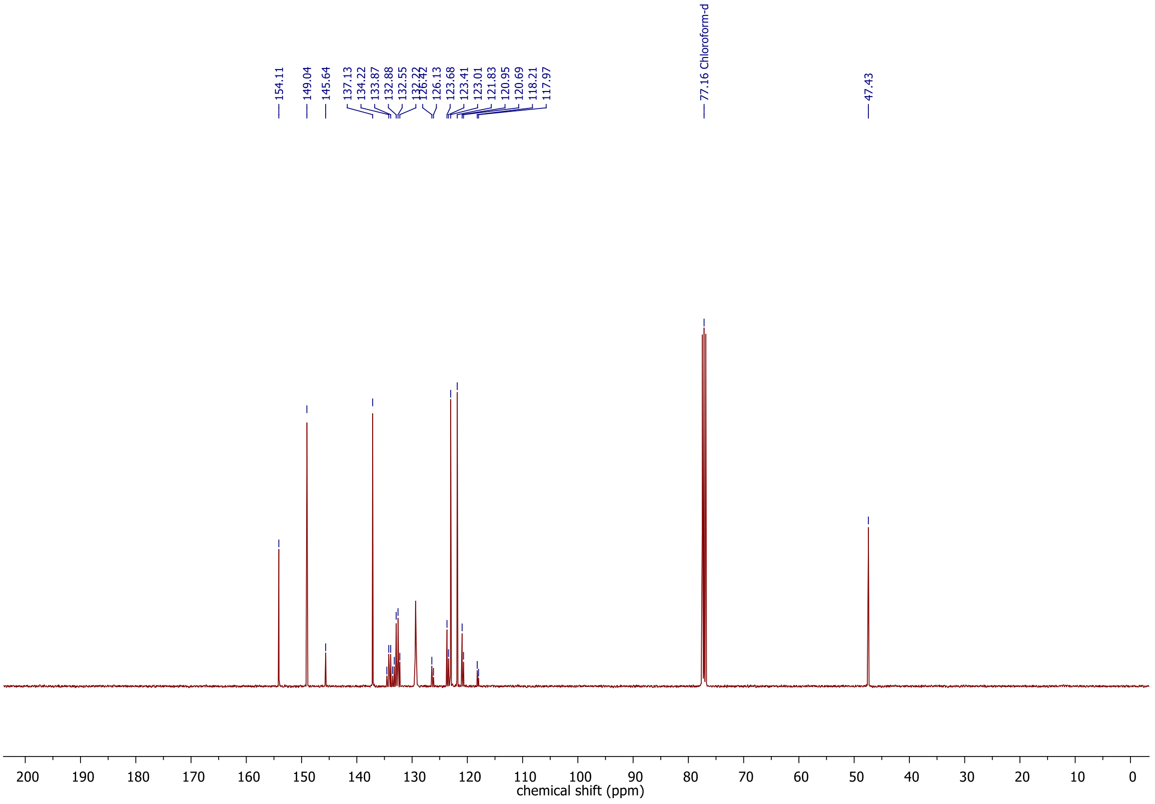

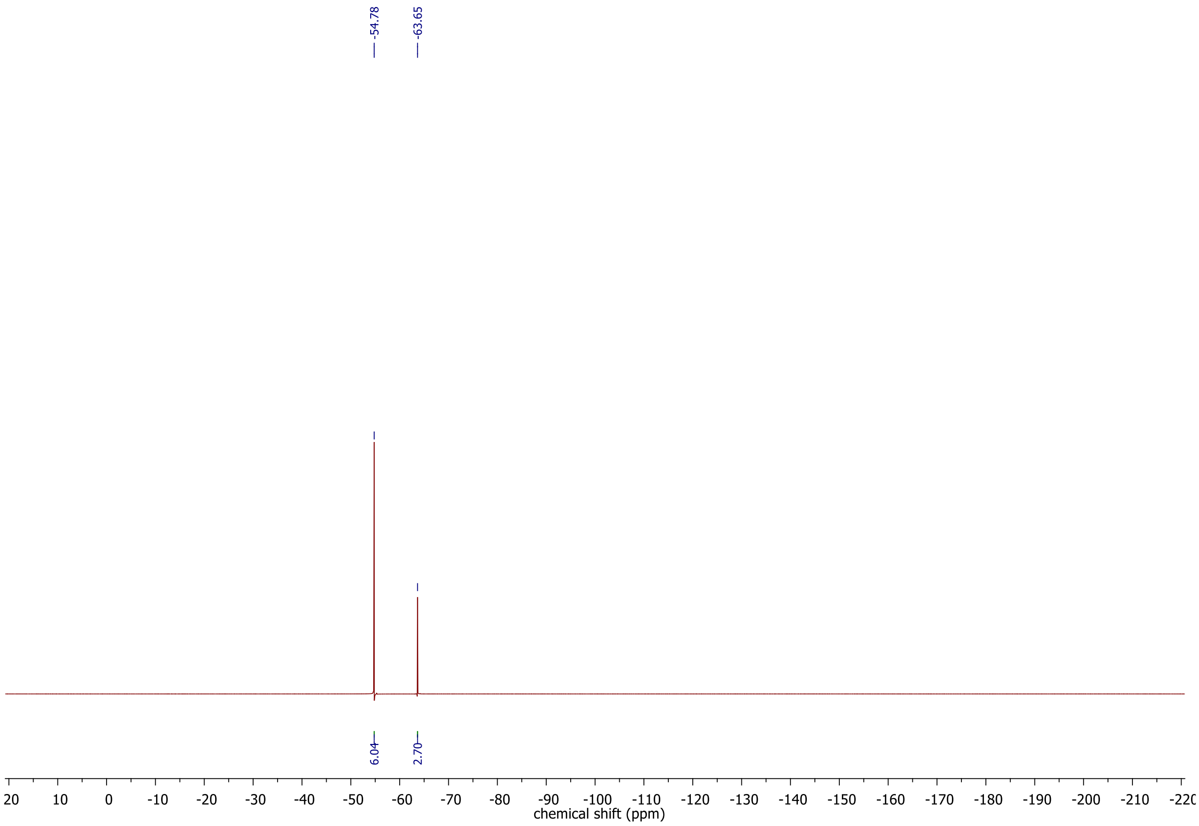
-54.78
-63.65
6.04
2.70
20
10
0
-10
-20
-30
-40
-50
-60
-70
-80
-90
-100
-110
-120
-130
-140
-150
-160
-170
-180
-190
-200
-210
-220
chemical shift (ppm)

***N*-(Furan-2-ylmethyl)-2,4,6-tris(trifluoromethyl)benzenesulfonamide (2j)**

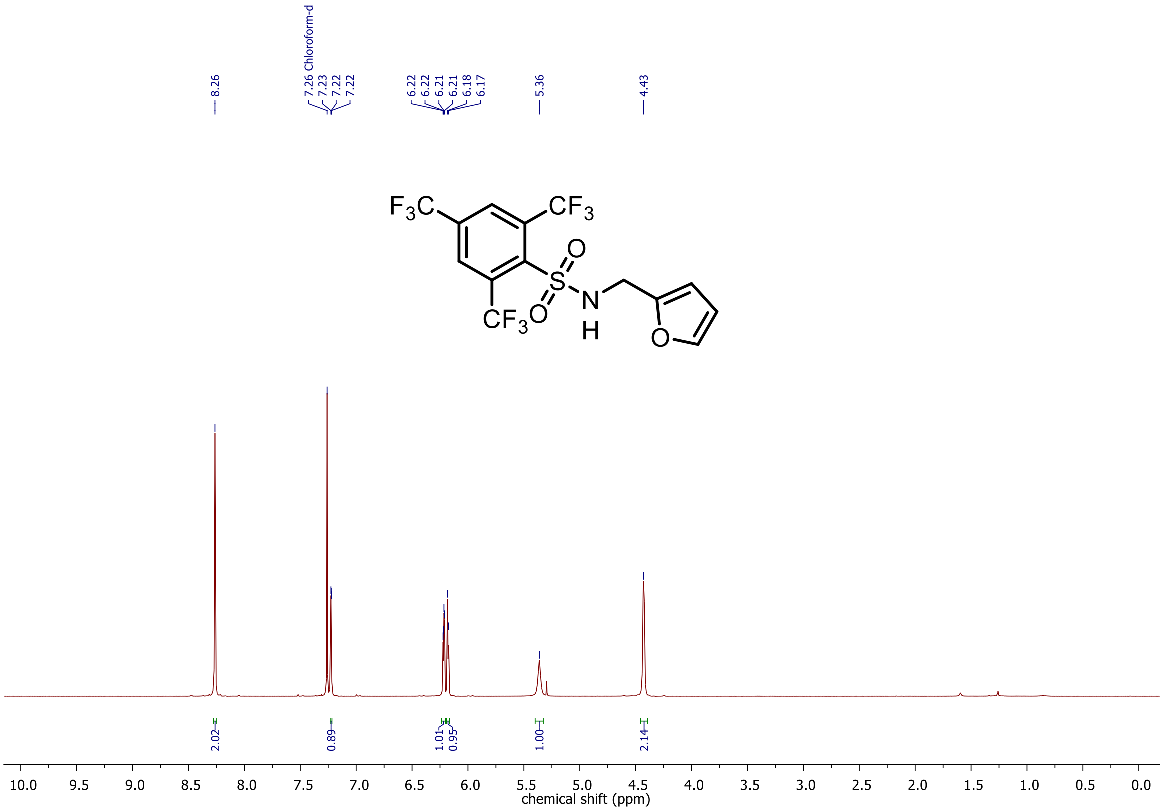

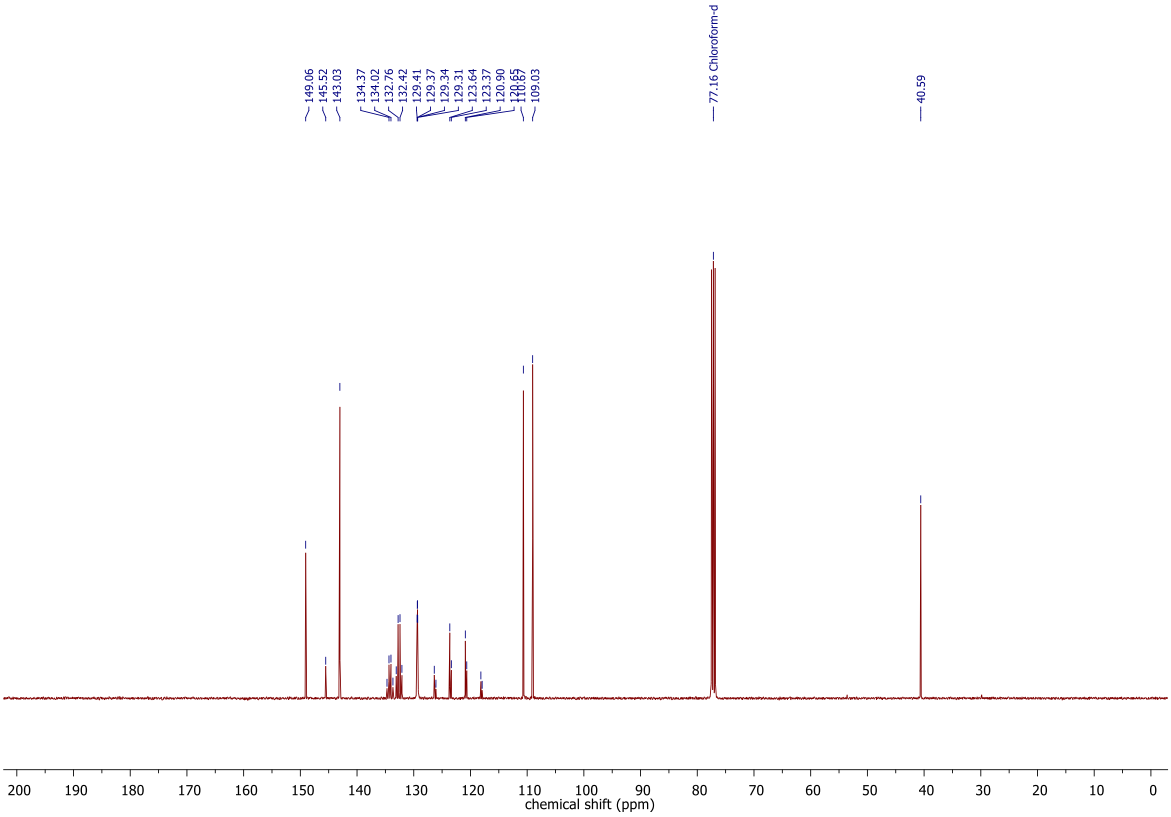

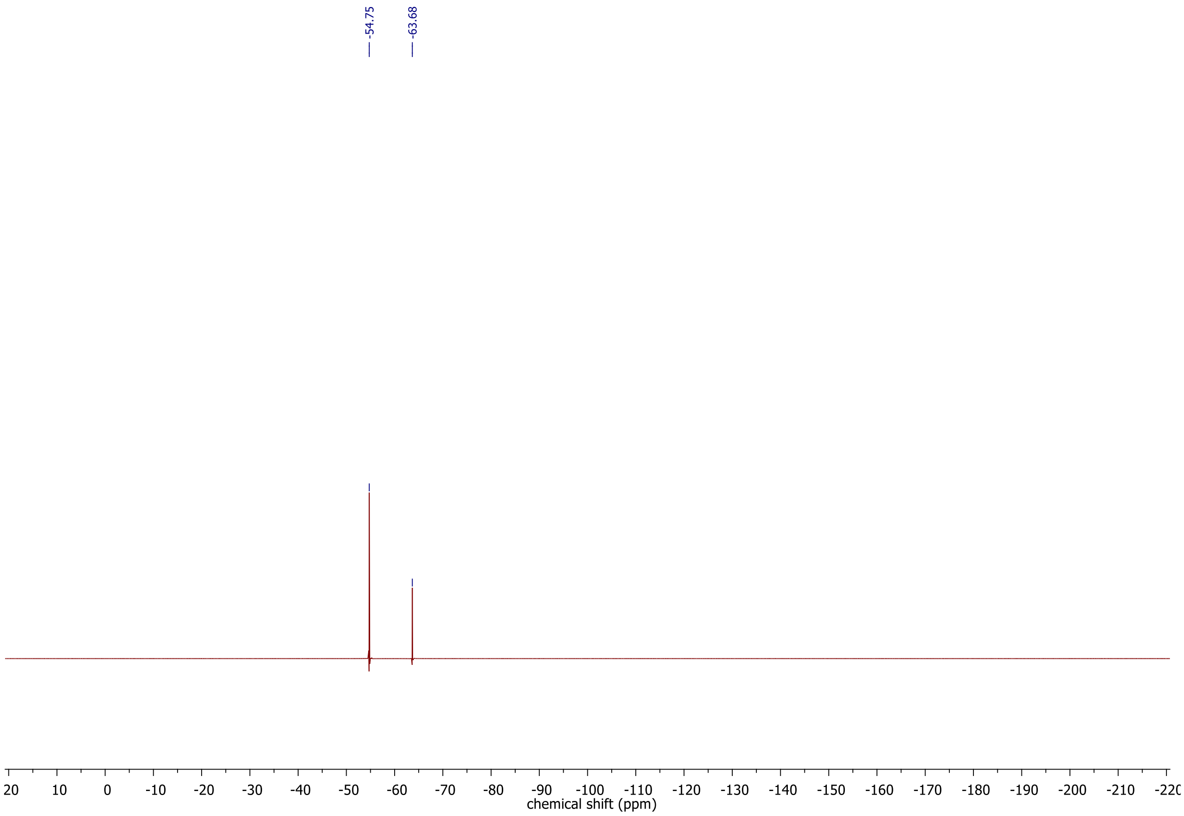

-54.75
-63.68
20 10 0 -10 -20 -30 -40 -50 -60 -70 -80 -90 -100 -110 -120 -130 -140 -150 -160 -170 -180 -190 -200 -210 -220
chemical shift (ppm)

N-(Thiophen-2-ylmethyl)-2,4,6-tris(trifluoromethyl)benzenesulfonamide (2k)

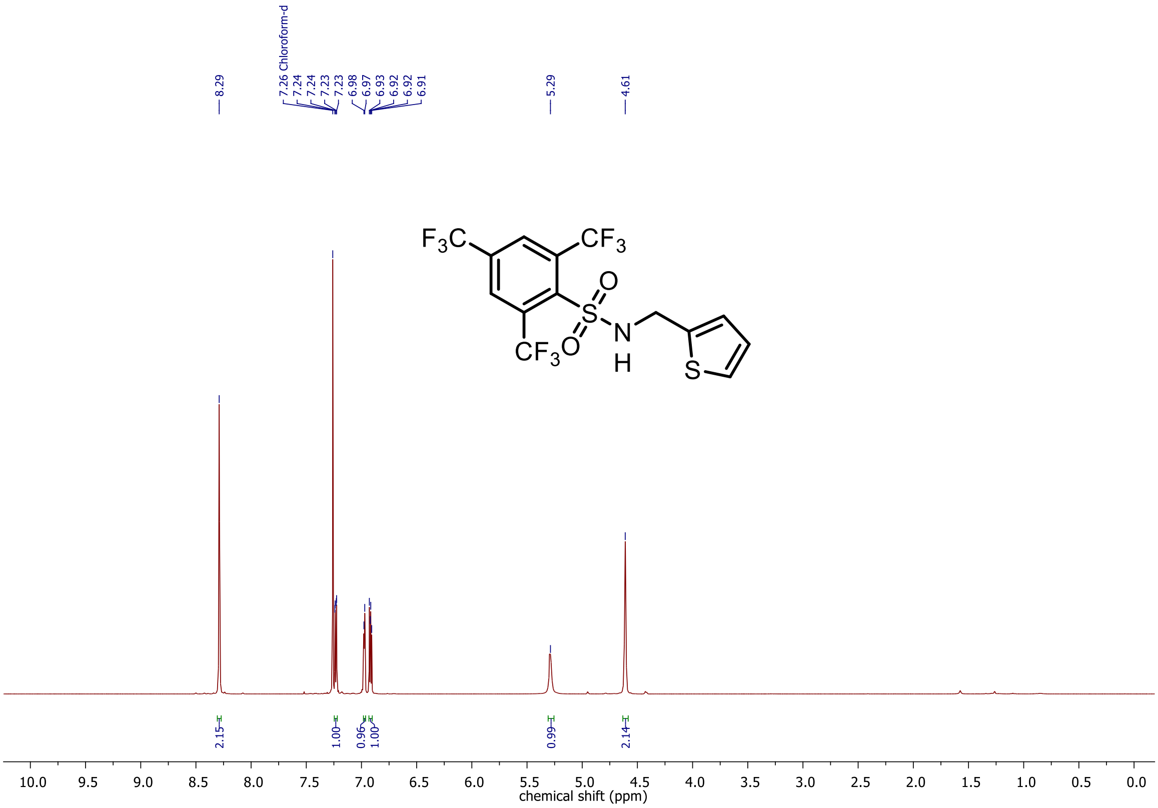

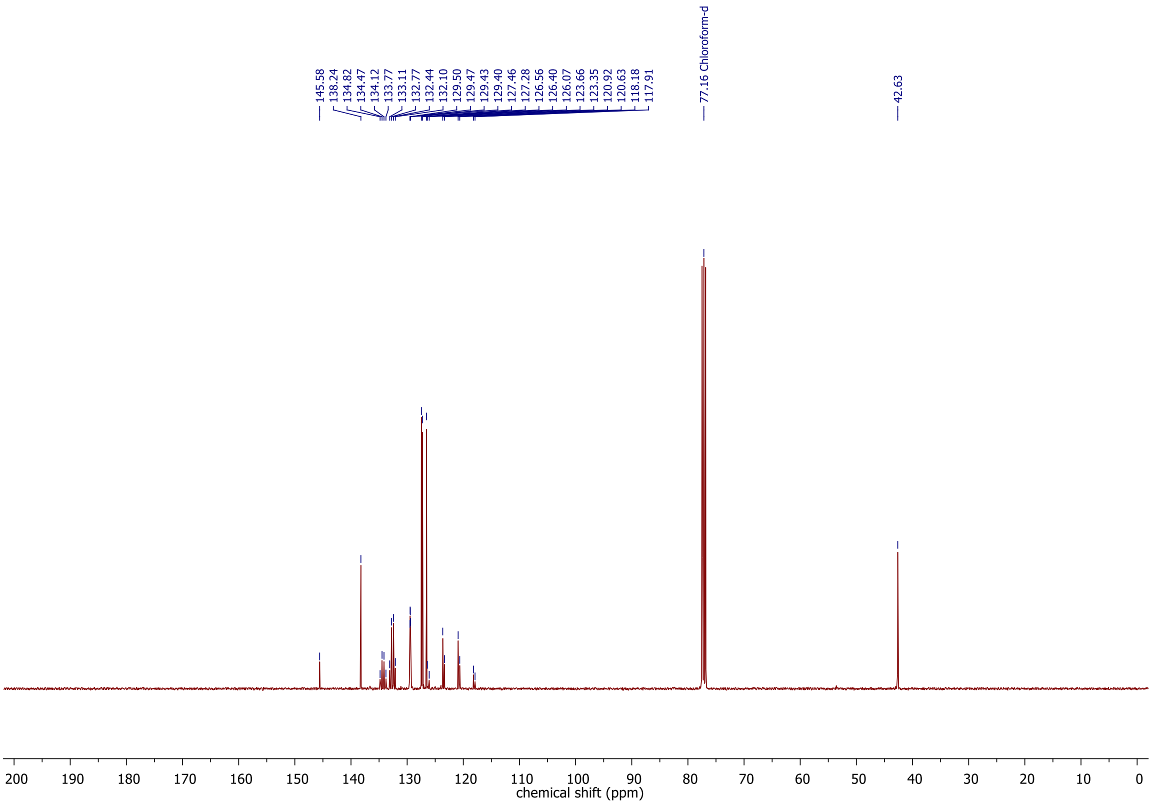

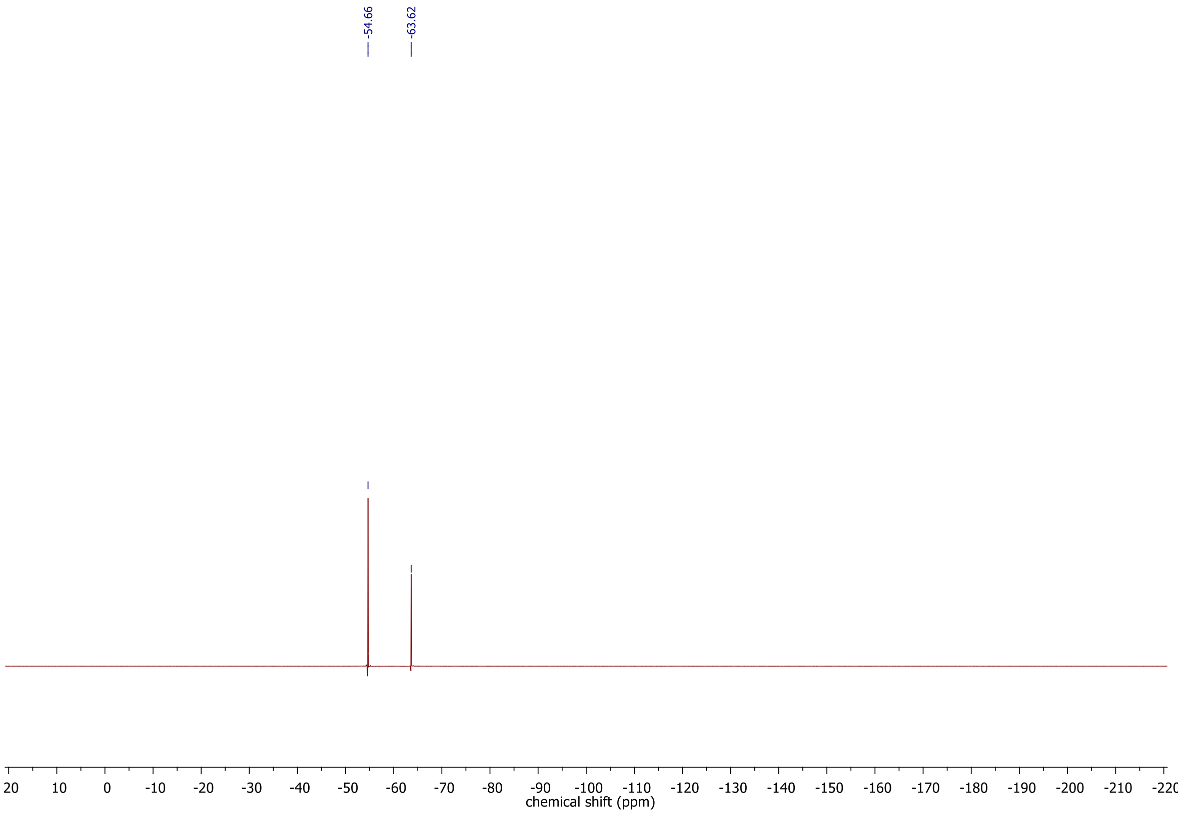
-54.66
-63.62
20
10
0
-10
-20
-30
-40
-50
-60
-70
-80
-90
-100
-110
-120
-130
-140
-150
-160
-170
-180
-190
-200
-210
-220
chemical shift (ppm)

N-Benzyl-2,4,6-tris(trifluoromethyl)benzenesulfonamide (2l)

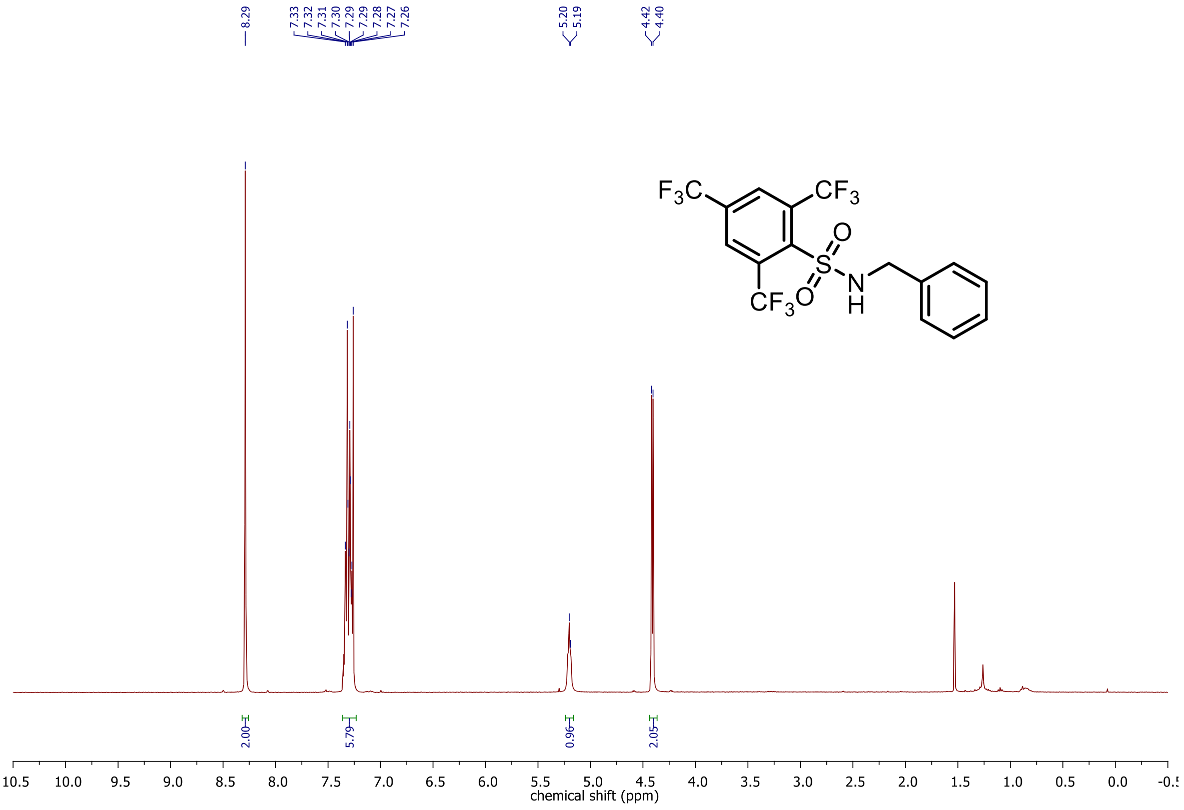

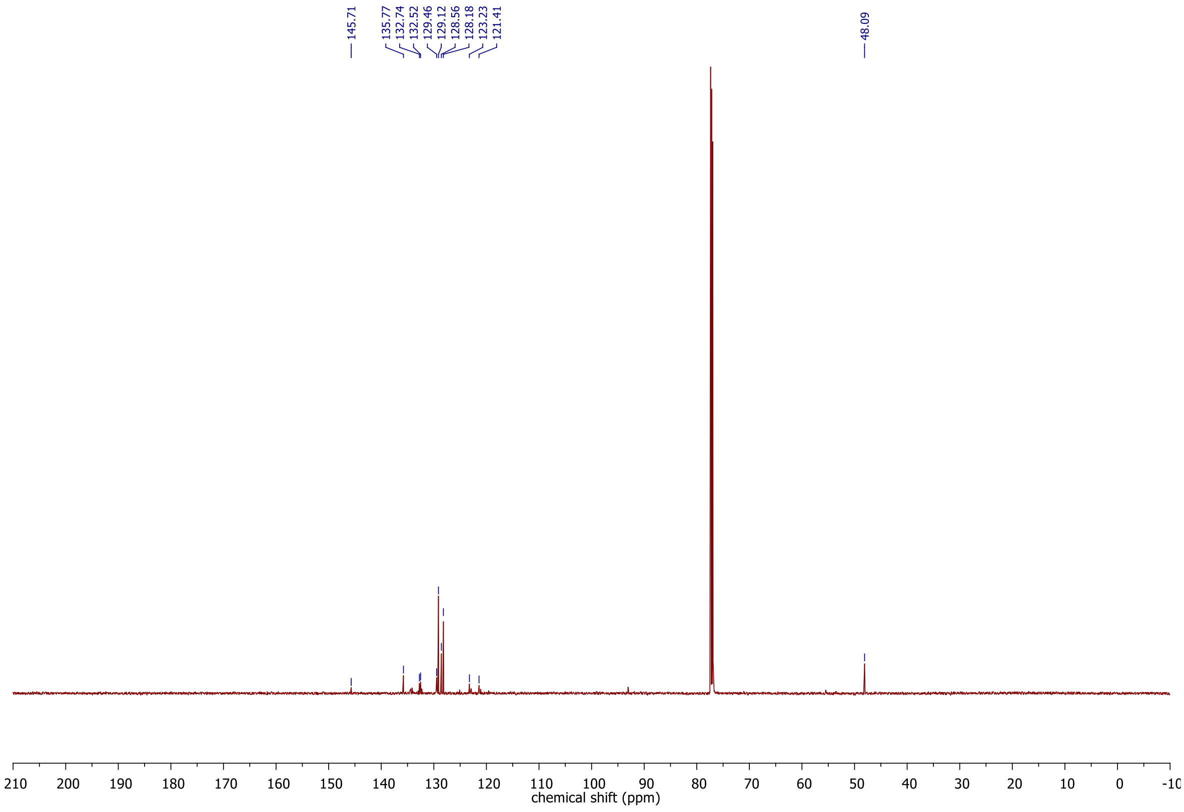

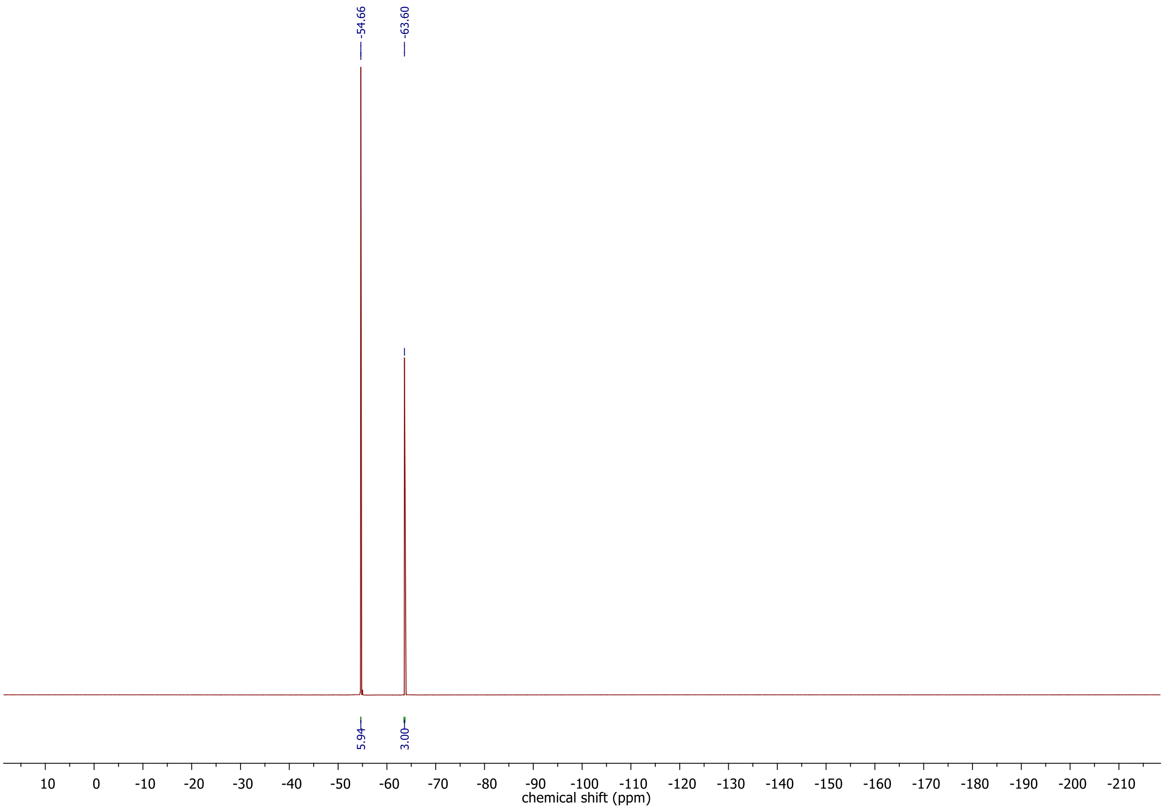
-54.66
-63.60
5.94
3.00
10 0 -10 -20 -30 -40 -50 -60 -70 -80 -90 -100 -110 -120 -130 -140 -150 -160 -170 -180 -190 -200 -210
chemical shift (ppm)

N-(4-Cyanobenzyl)-2,4,6-tris(trifluoromethyl)benzenesulfonamide (2m)

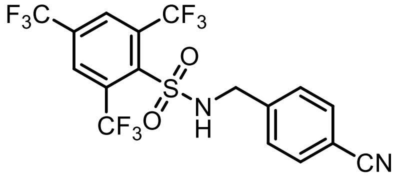

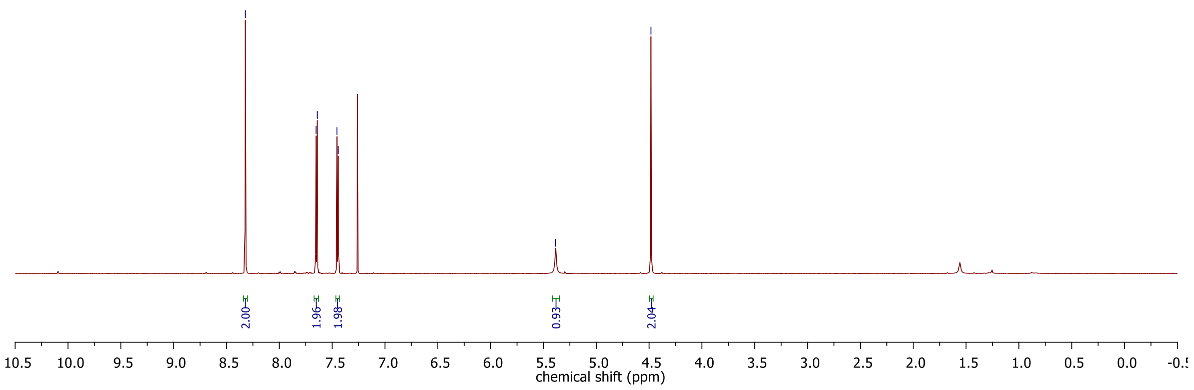

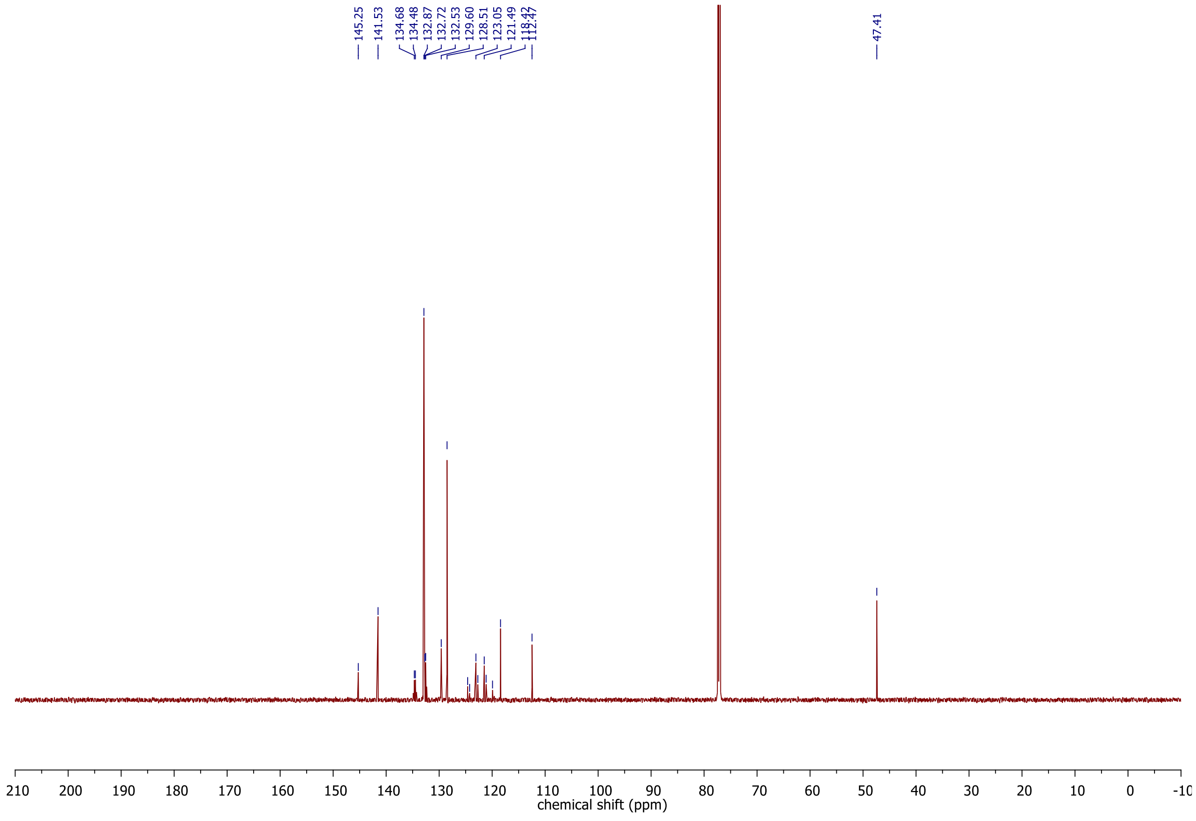

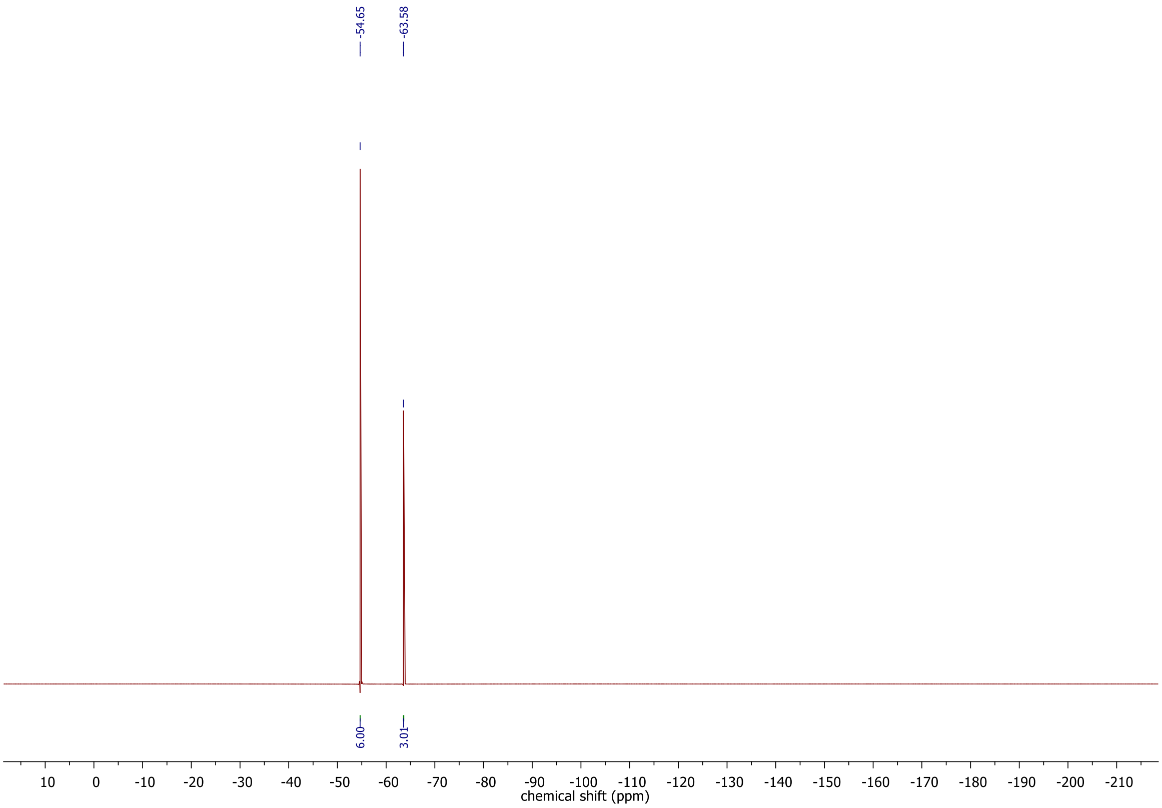

-54.65
-63.58
6.00
3.01
10
0
-10
-20
-30
-40
-50
-60
-70
-80
-90
-100
-110
-120
-130
-140
-150
-160
-170
-180
-190
-200
-210
chemical shift (ppm)

N-(4-Methoxybenzyl)-2,4,6-tris(trifluoromethyl)benzenesulfonamide (2n)

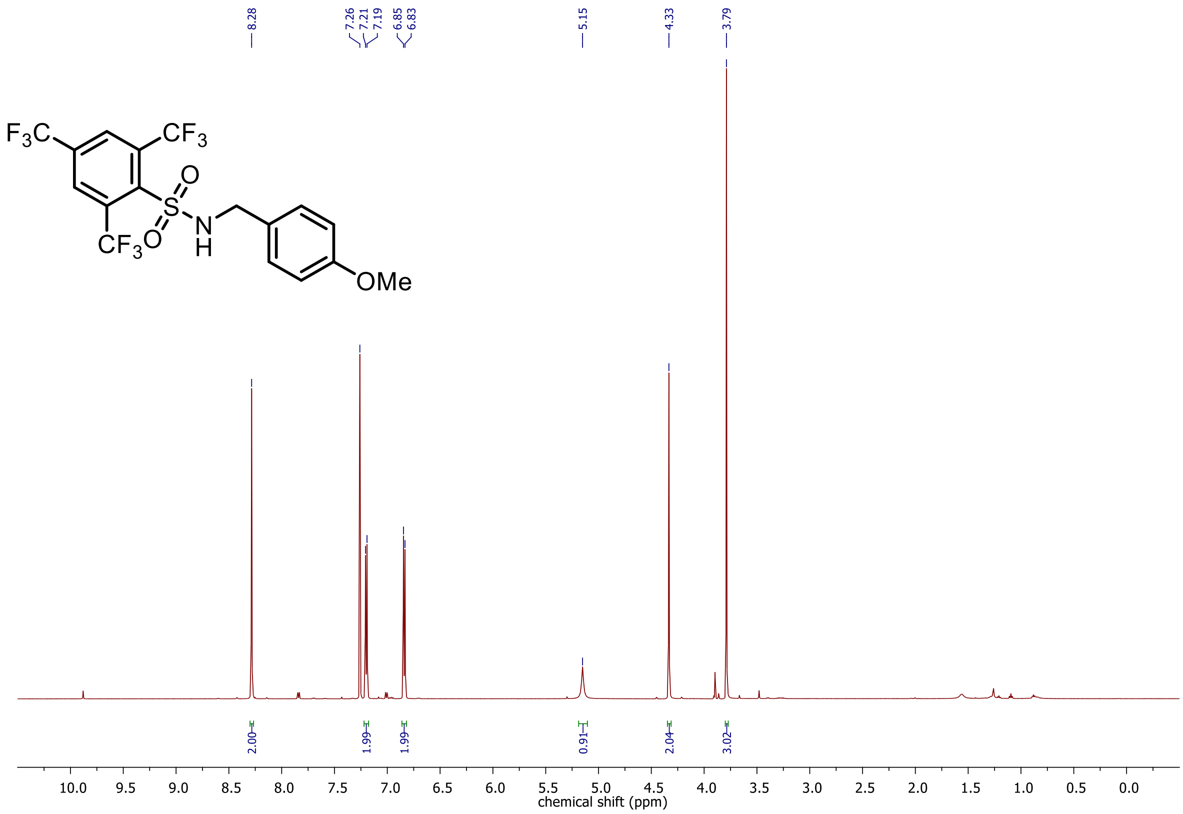

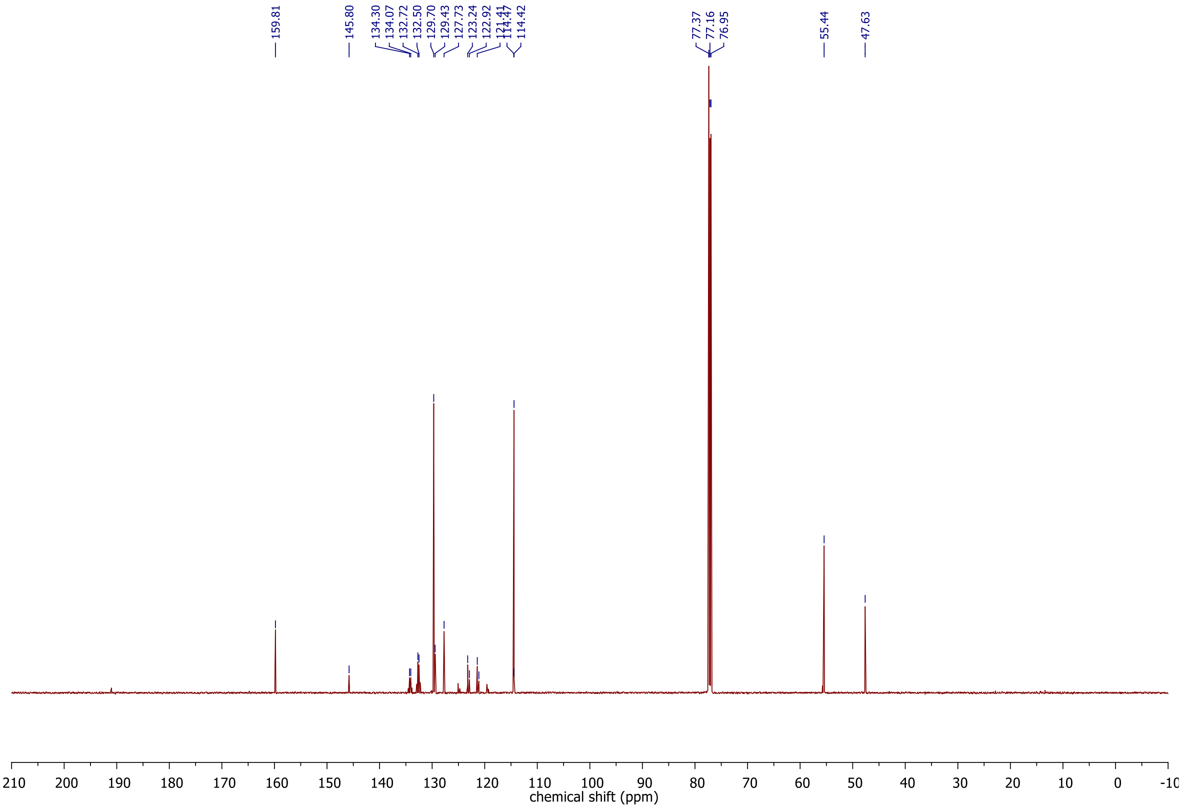

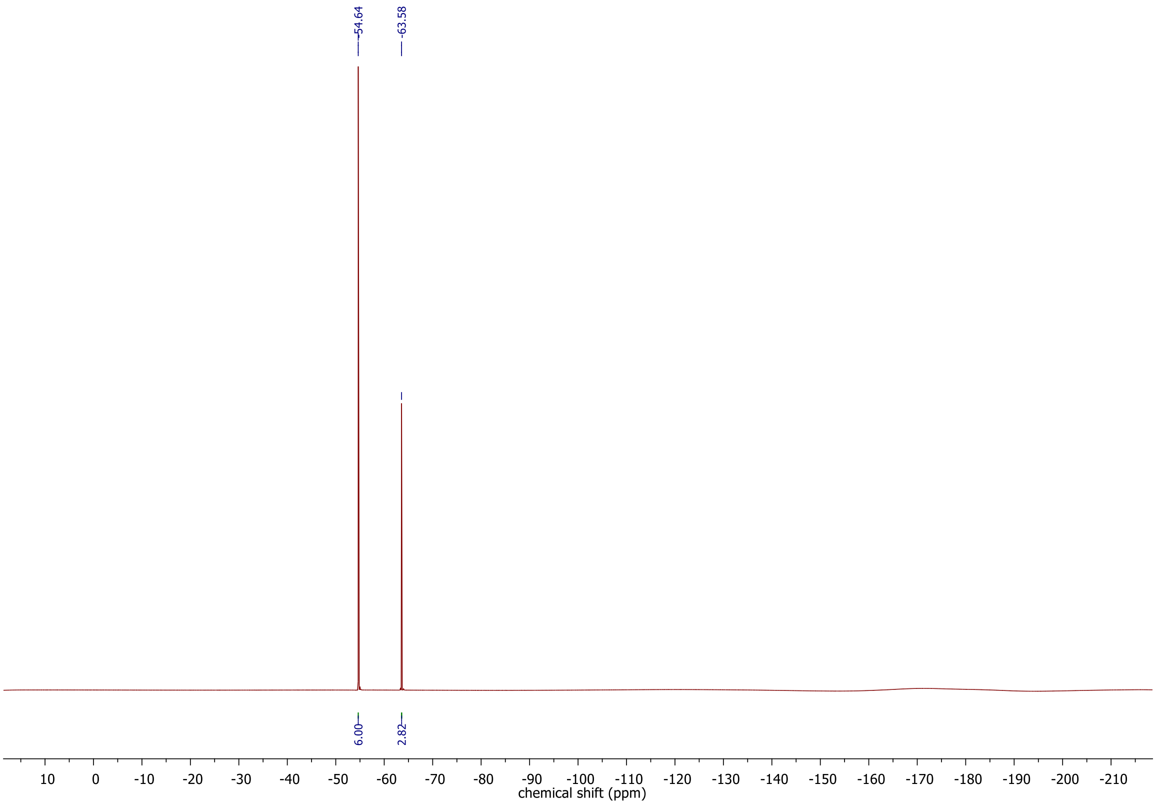

-54.64
-63.58
6.00
2.82
10 0 -10 -20 -30 -40 -50 -60 -70 -80 -90 -100 -110 -120 -130 -140 -150 -160 -170 -180 -190 -200 -210
chemical shift (ppm)

(*R*)-*N*-(1-(Naphthalen-1-yl)ethyl)-2,4,6-tris(trifluoromethyl)benzenesulfonamide (2o)

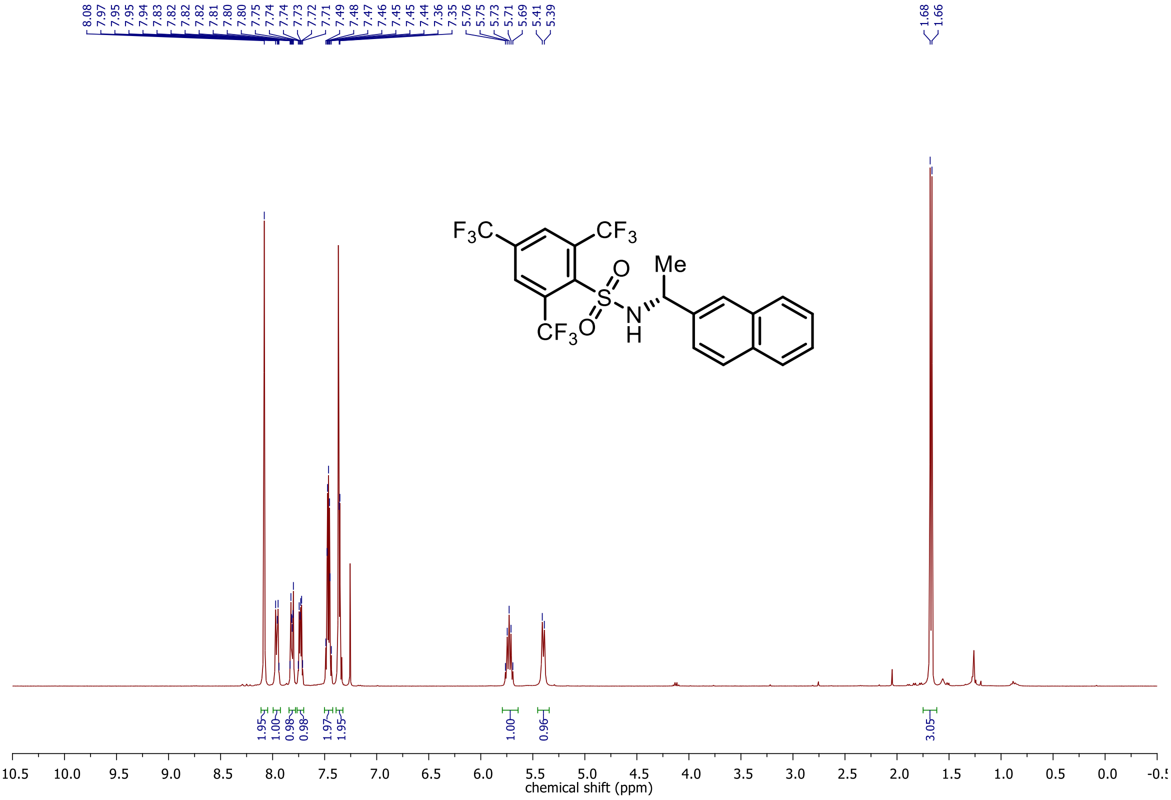

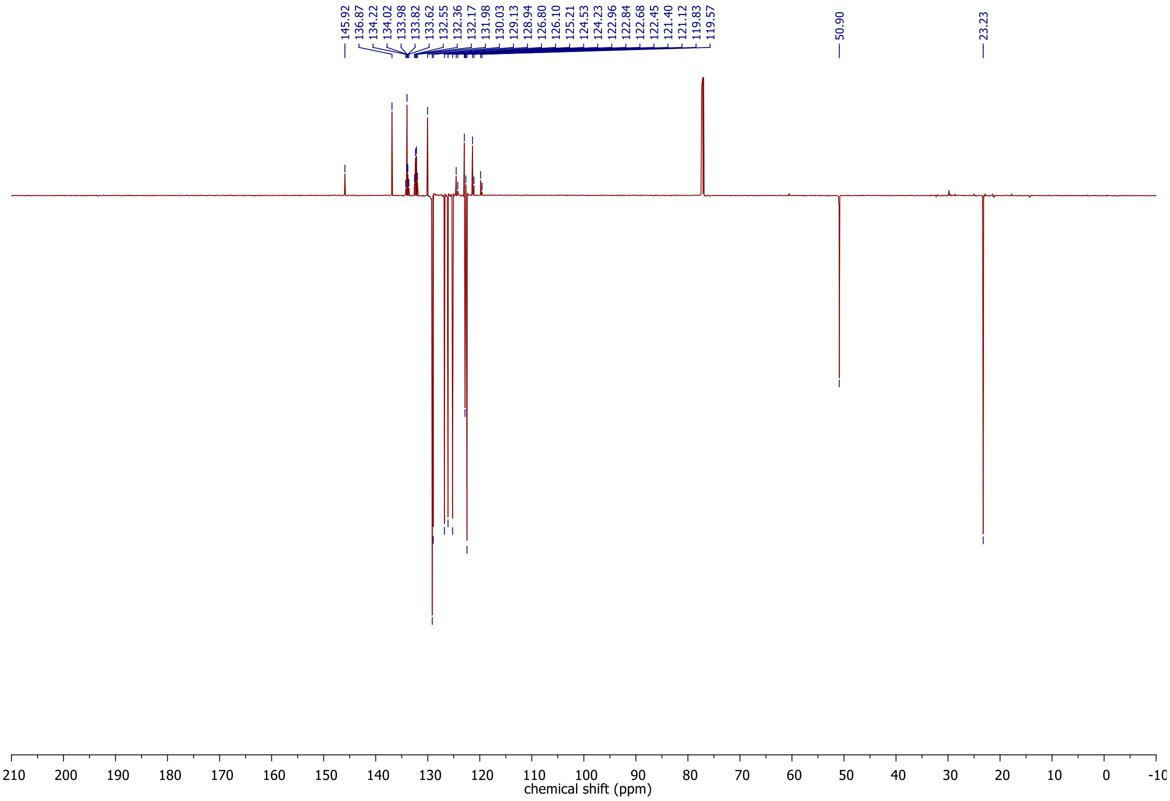

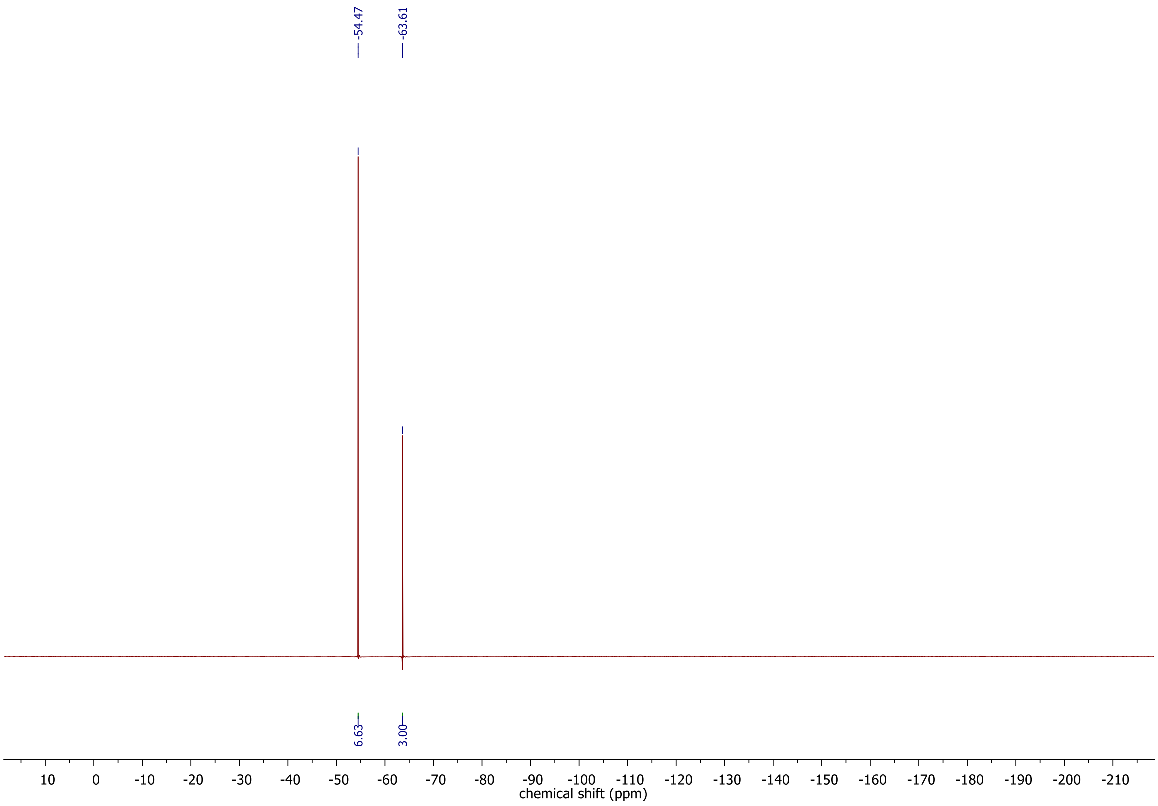

-54.47
-63.61
6.63
3.00
10
0
-10
-20
-30
-40
-50
-60
-70
-80
-90
-100
-110
-120
-130
-140
-150
-160
-170
-180
-190
-200
-210
chemical shift (ppm)

Butyl (*R*)-2-(4-(2-fluoro-4-(((2,4,6-tris(trifluoromethyl)phenyl)sulfonamido)methyl)phenoxy)phenoxy)propanoate (2p)

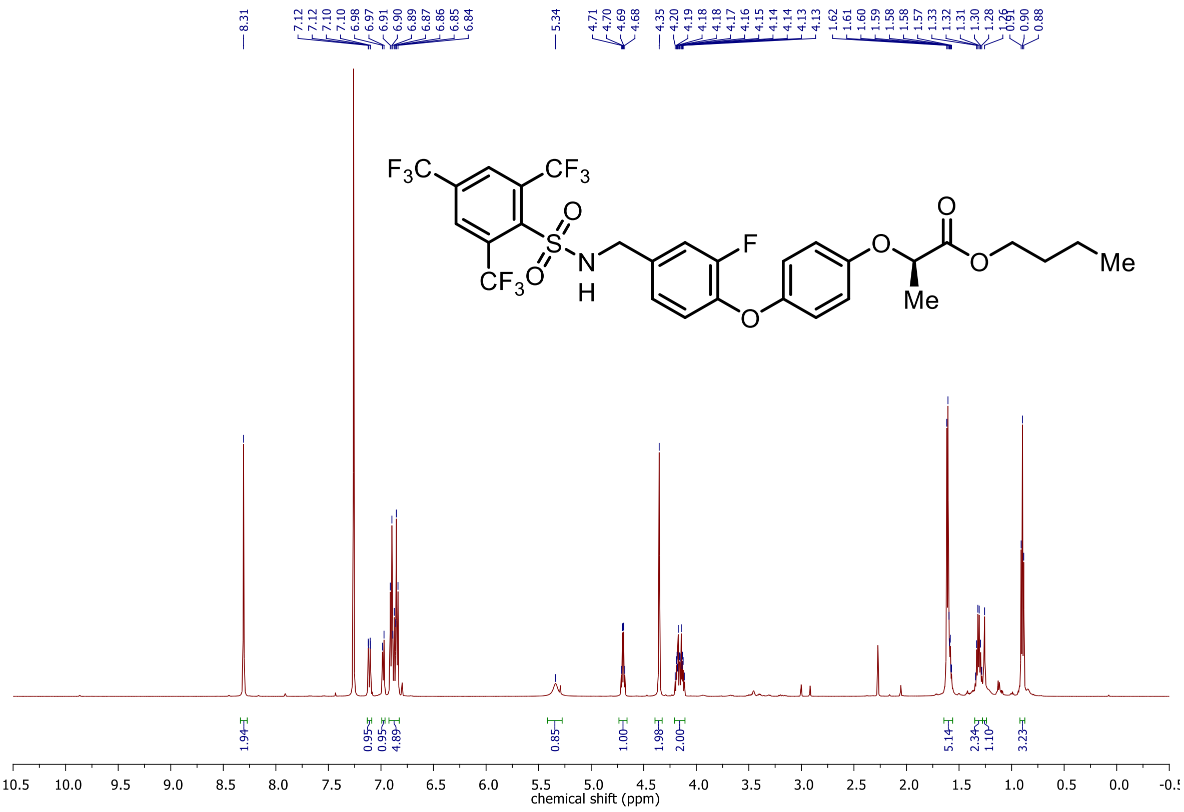

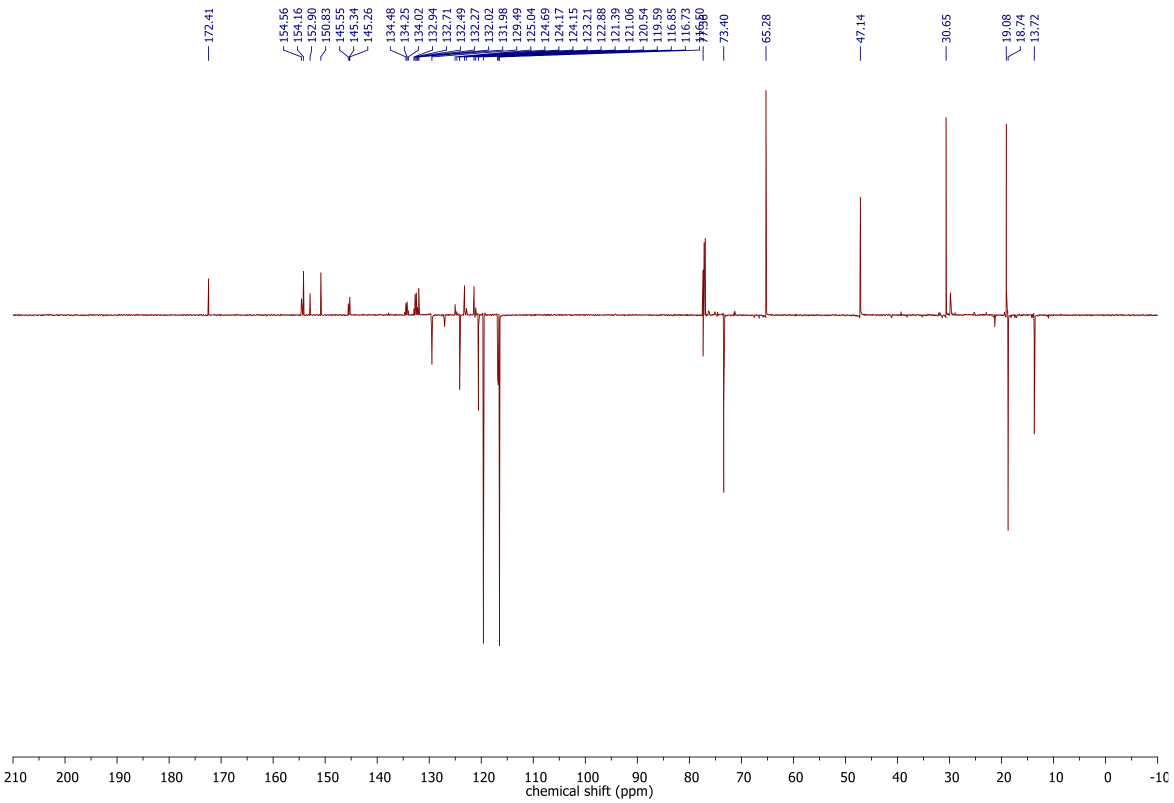

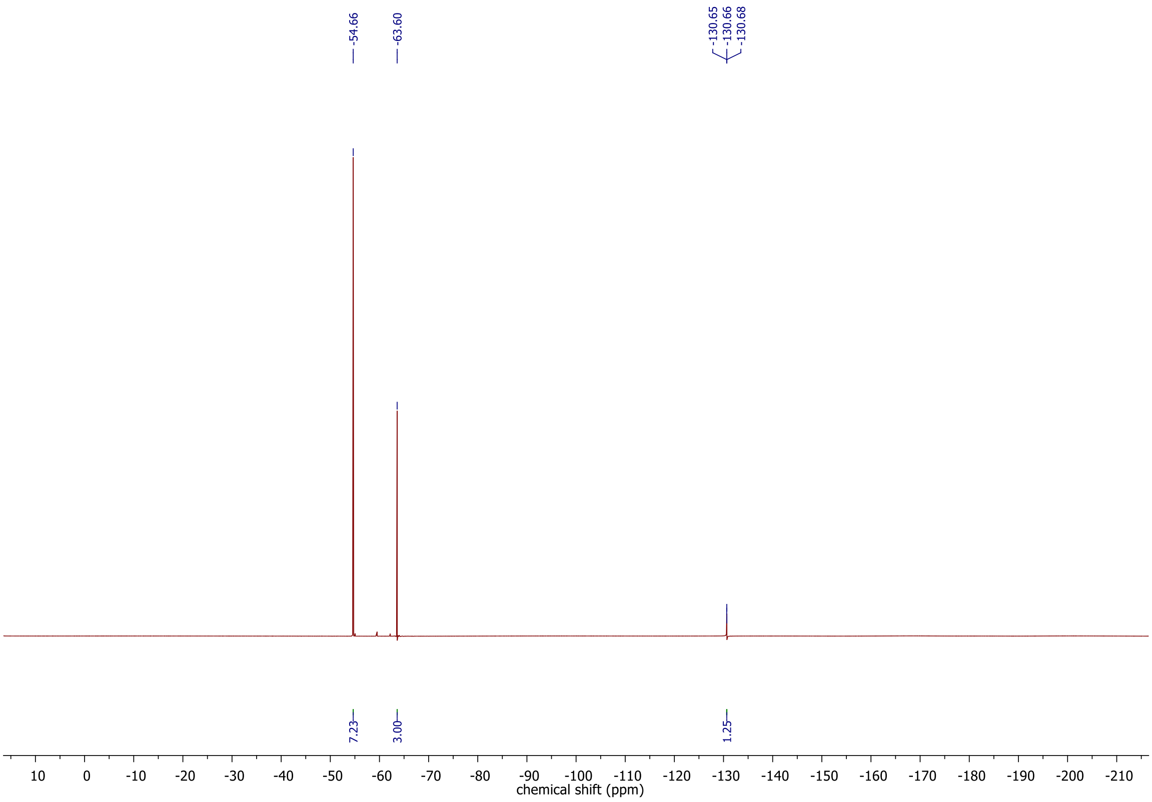
-54.66
-63.60
-130.65
-130.66
-130.68
7.23
3.00
1.25
10 0 -10 -20 -30 -40 -50 -60 -70 -80 -90 -100 -110 -120 -130 -140 -150 -160 -170 -180 -190 -200 -210
chemical shift (ppm)

***N*-(*tert*-Butyl)-2,4,6-tris(trifluoromethyl)benzenesulfonamide (2q)**

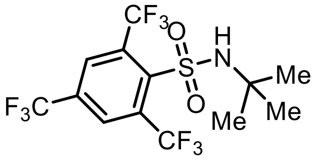

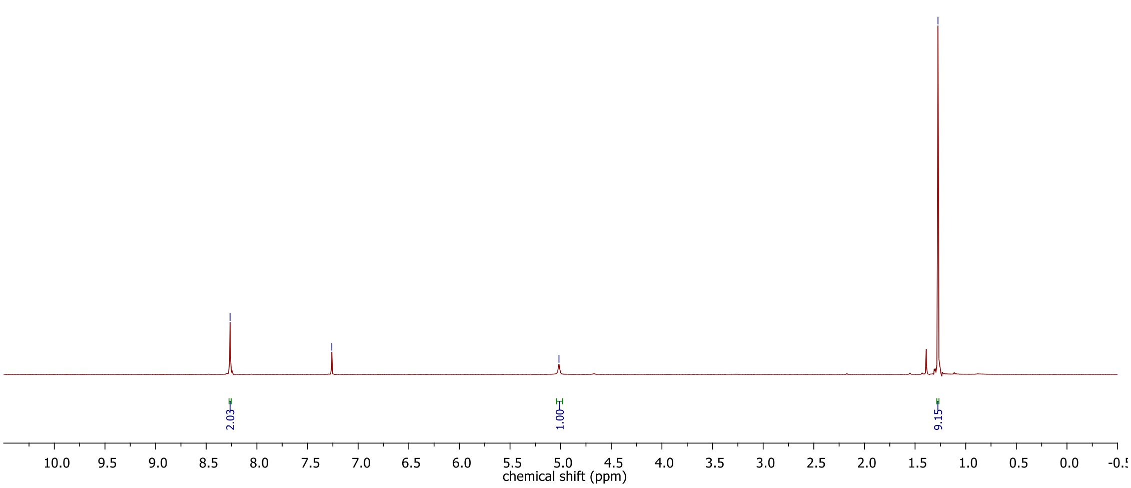

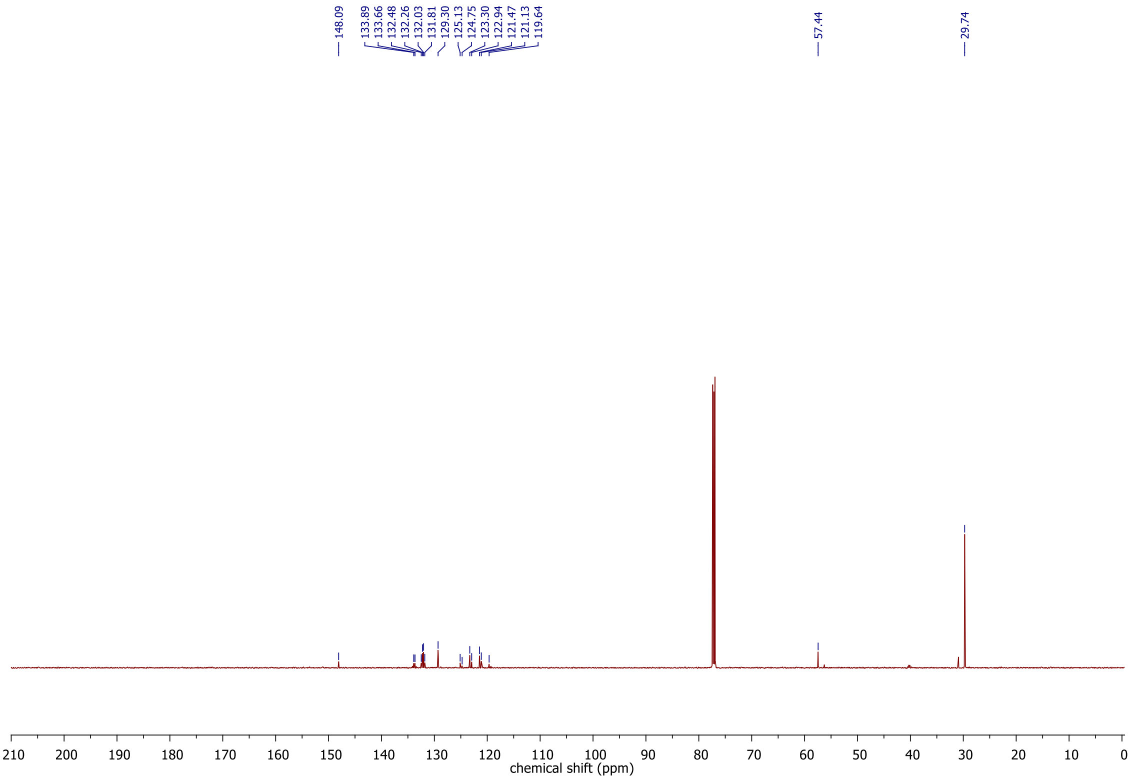

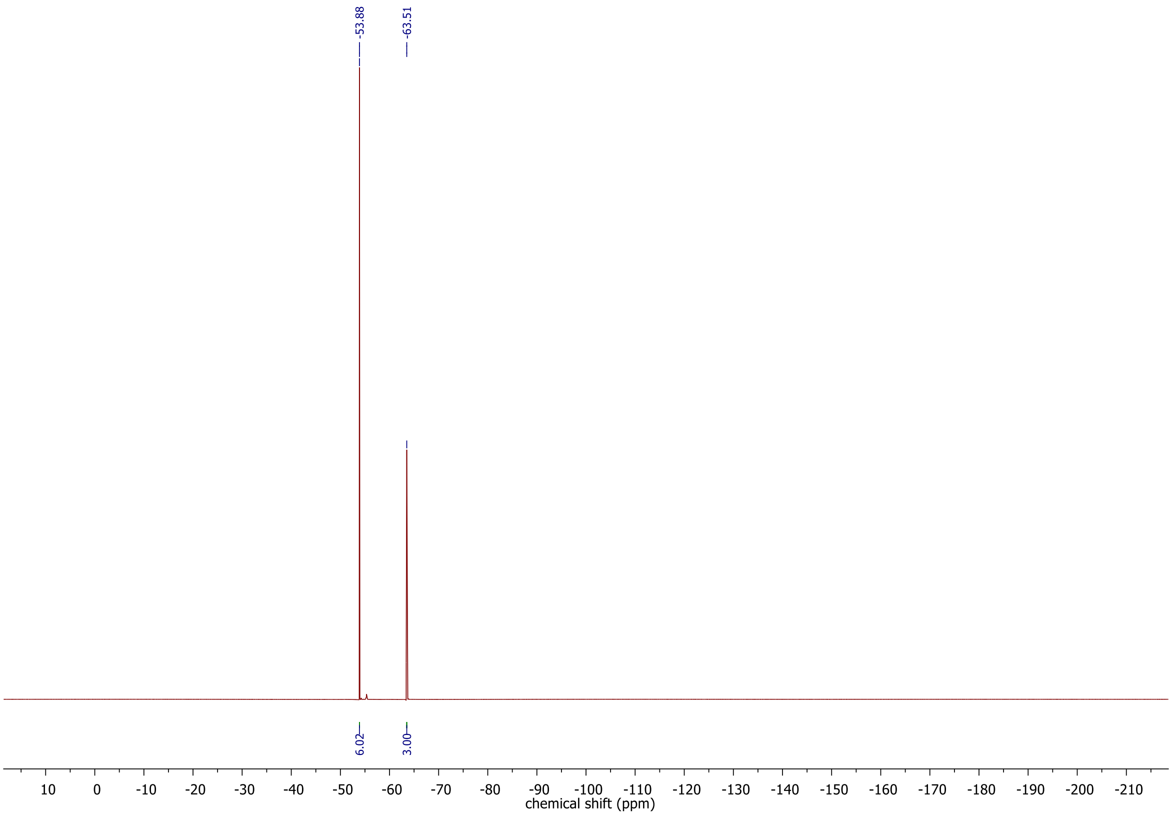

-53.88
-63.51
6.02
3.00
10
0
-10
-20
-30
-40
-50
-60
-70
-80
-90
-100
-110
-120
-130
-140
-150
-160
-170
-180
-190
-200
-210
chemical shift (ppm)

***N*-((1*S*,2*R*,5*S*)-2-Isopropyl-5-methylcyclohexyl)-2,4,6-tris(trifluoromethyl)benzenesulfonamide (2r)**

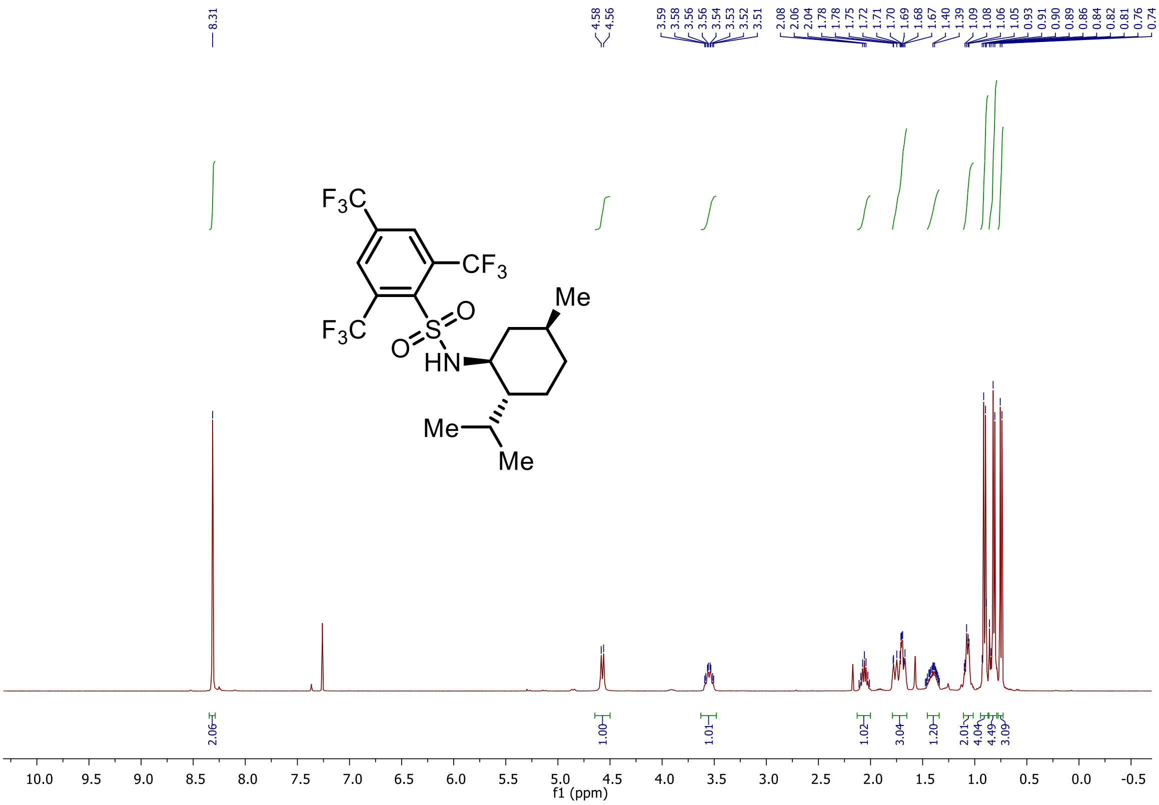

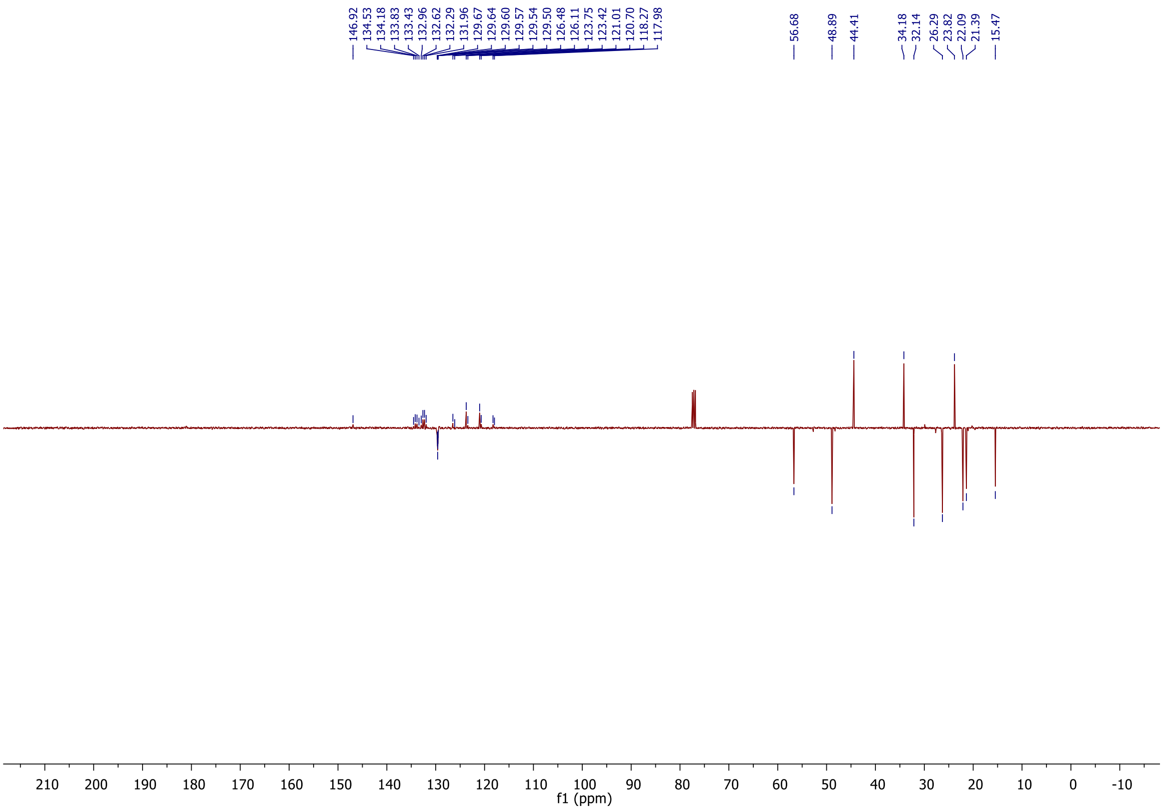

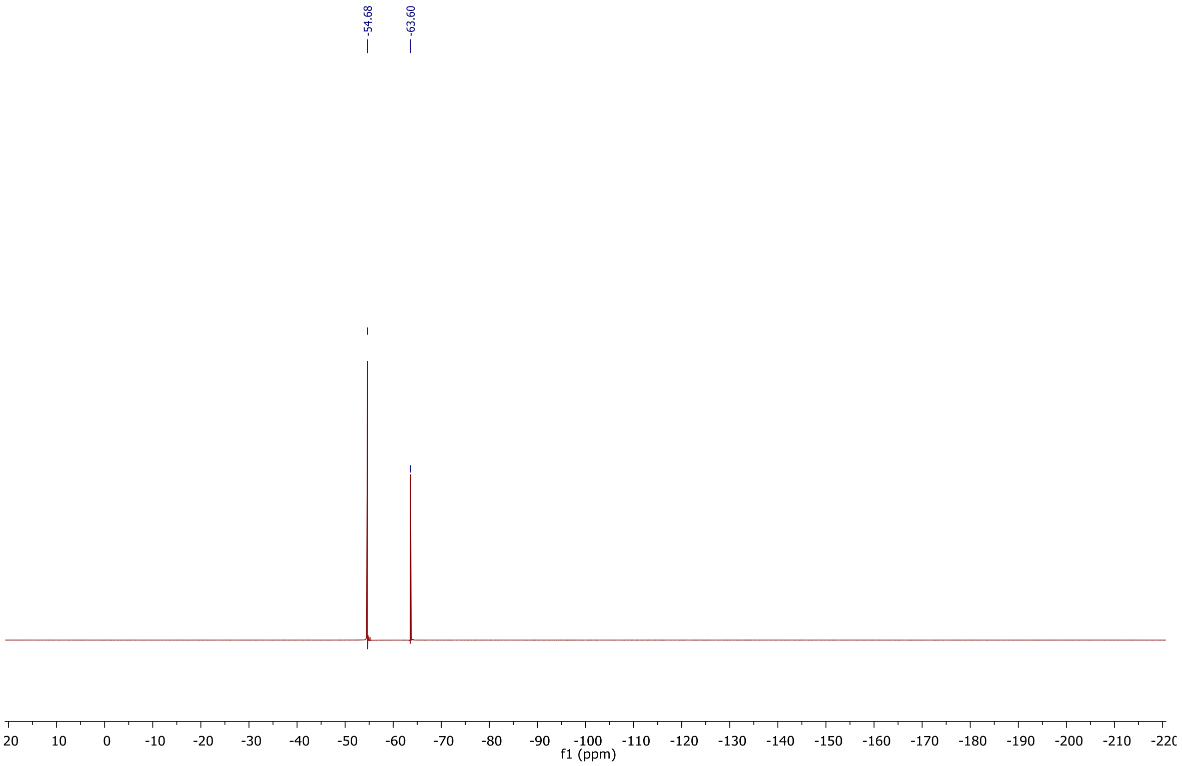

-54.68
-63.60
20
10
0
-10
-20
-30
-40
-50
-60
-70
-80
-90
-100
-110
-120
-130
-140
-150
-160
-170
-180
-190
-200
-210
-220
f1 (ppm)

N-(4-Methoxyphenyl)-2,4,6-tris(trifluoromethyl)benzenesulfonamide (2s)

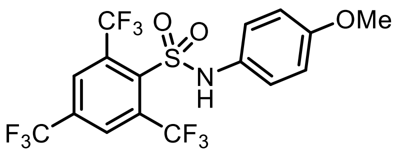

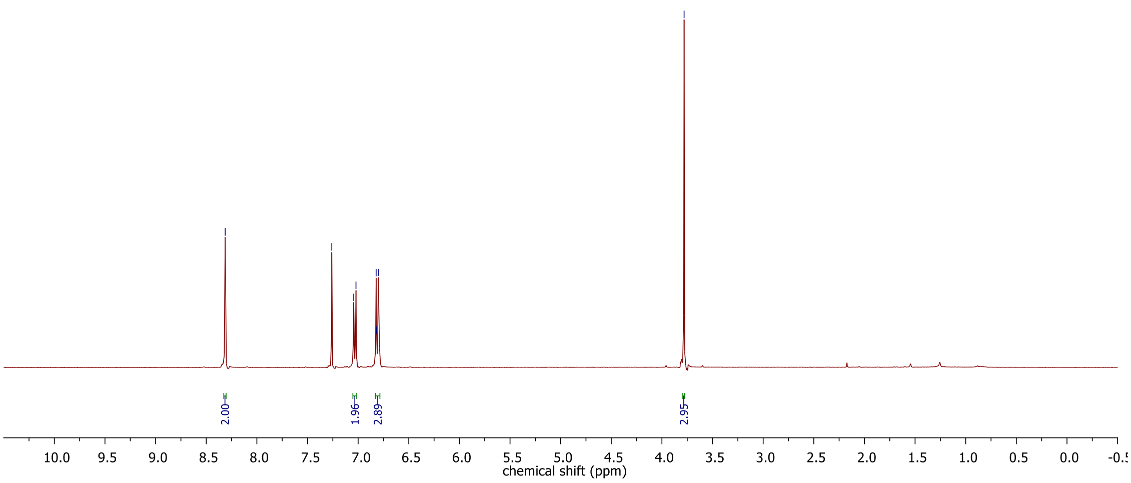

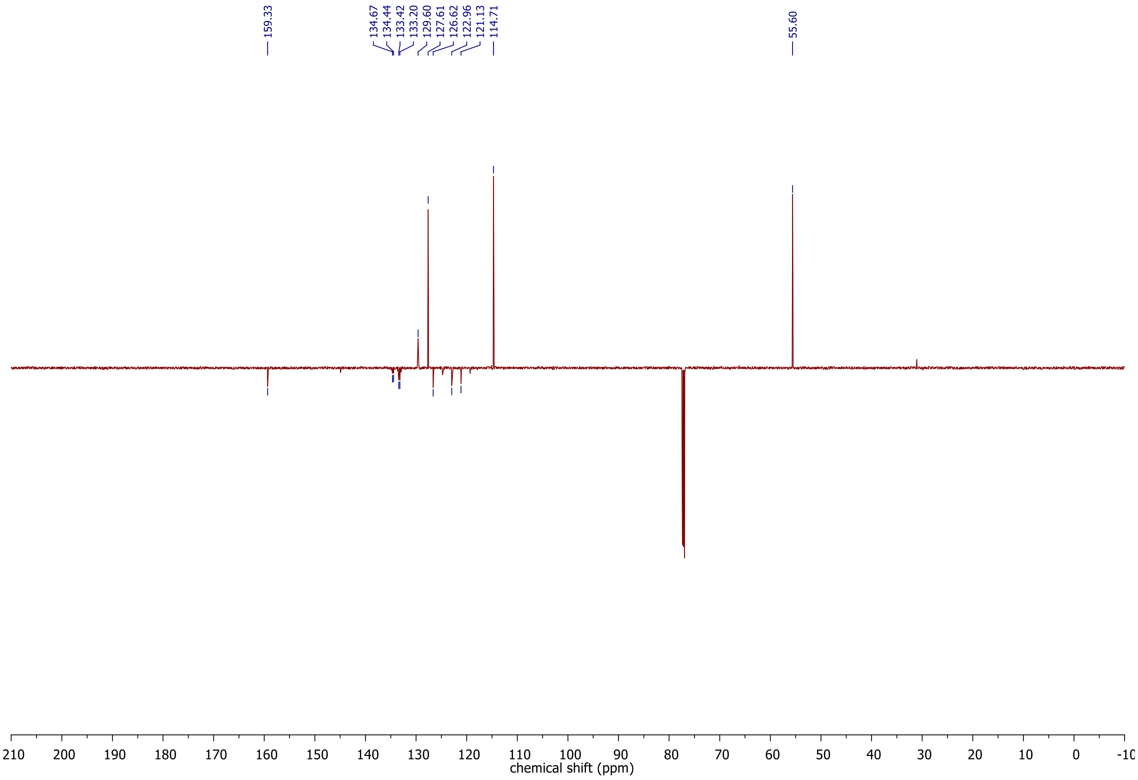

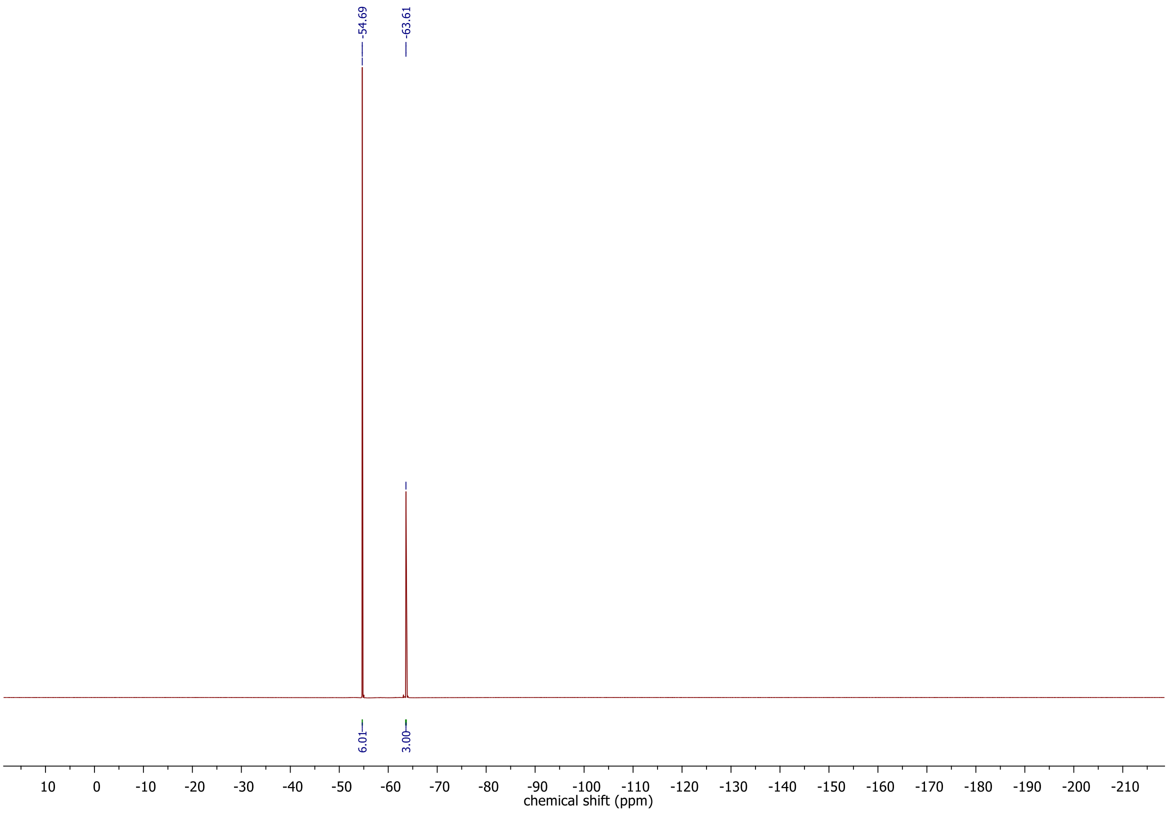
-54.69
-63.61
6.01
3.00
10 0 -10 -20 -30 -40 -50 -60 -70 -80 -90 -100 -110 -120 -130 -140 -150 -160 -170 -180 -190 -200 -210
chemical shift (ppm)

2,4,6-Tris(trifluoromethyl)-*N*-(3,4,5-trimethoxyphenyl)benzenesulfonamide (2t)

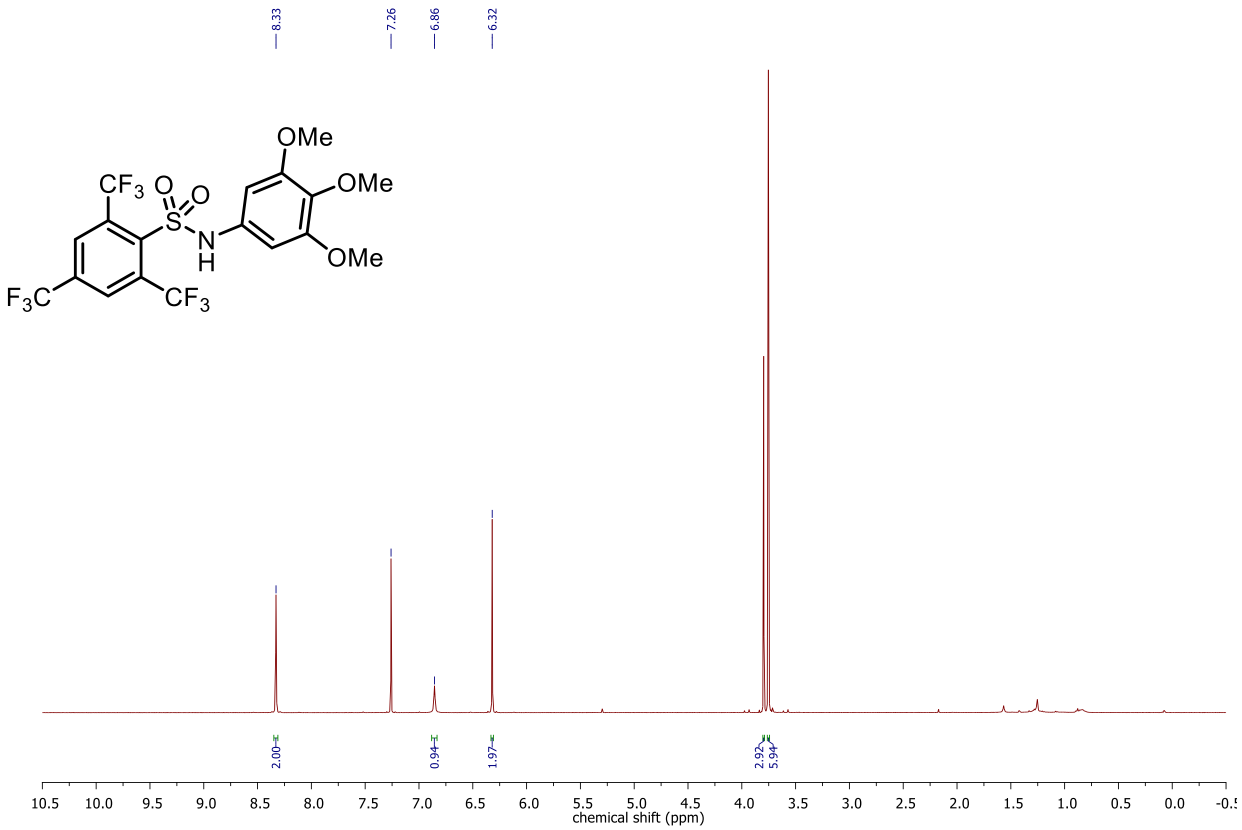

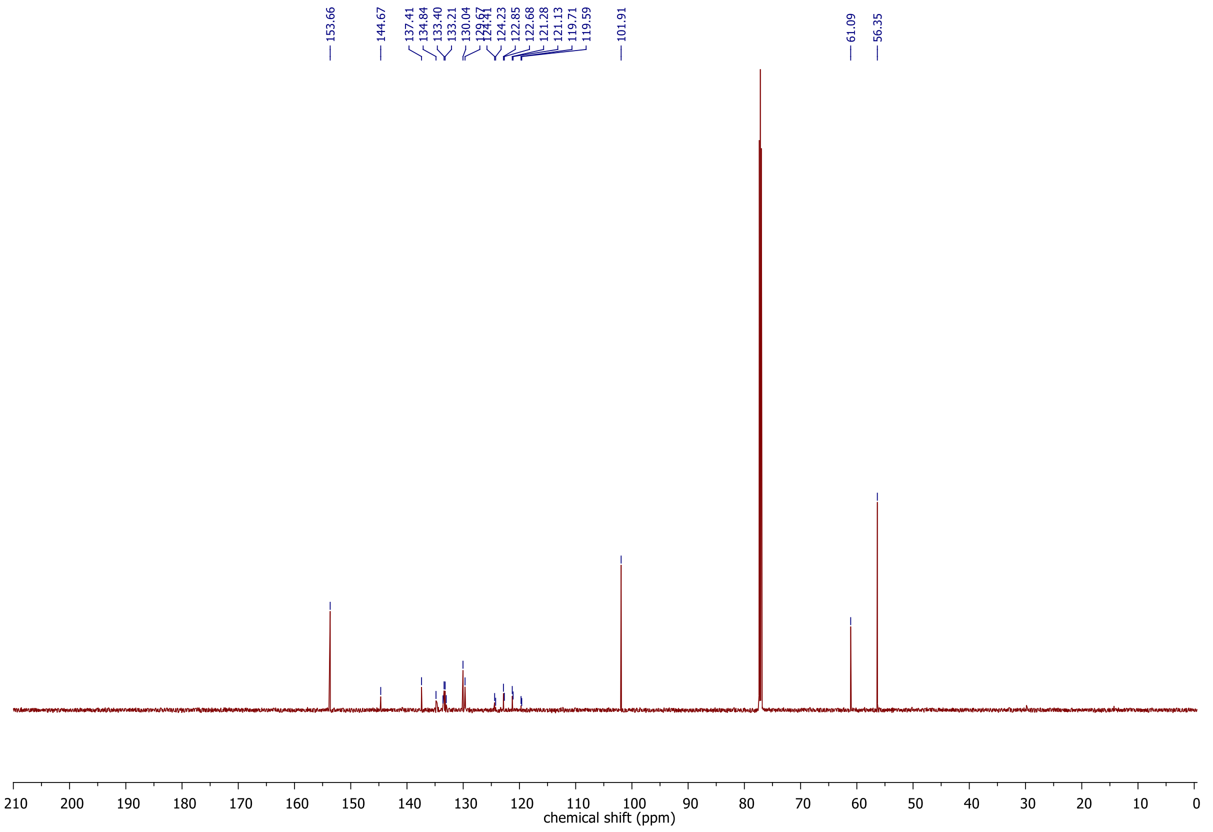

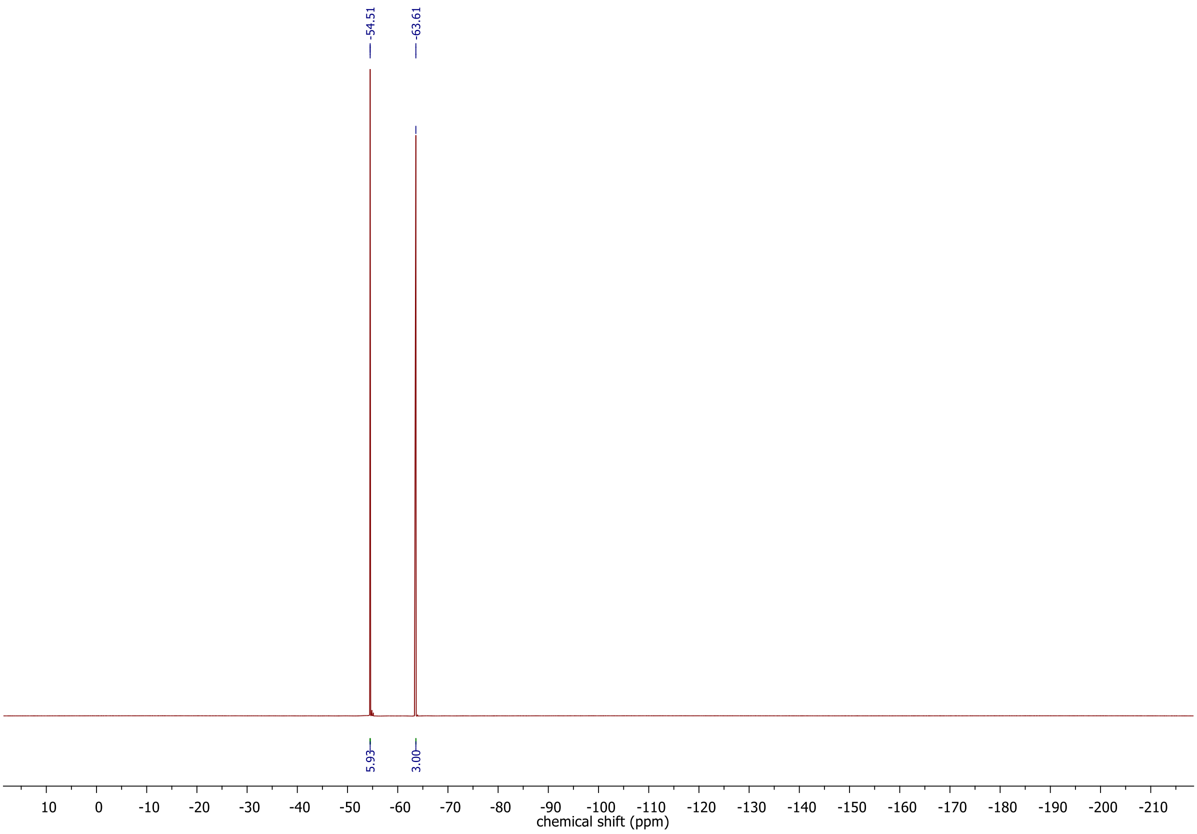

-54.51
-63.61
5.93
3.00
10 0 -10 -20 -30 -40 -50 -60 -70 -80 -90 -100 -110 -120 -130 -140 -150 -160 -170 -180 -190 -200 -210
chemical shift (ppm)

N-(1H-Indol-5-yl)-2,4,6-tris(trifluoromethyl)benzenesulfonamide (2u)

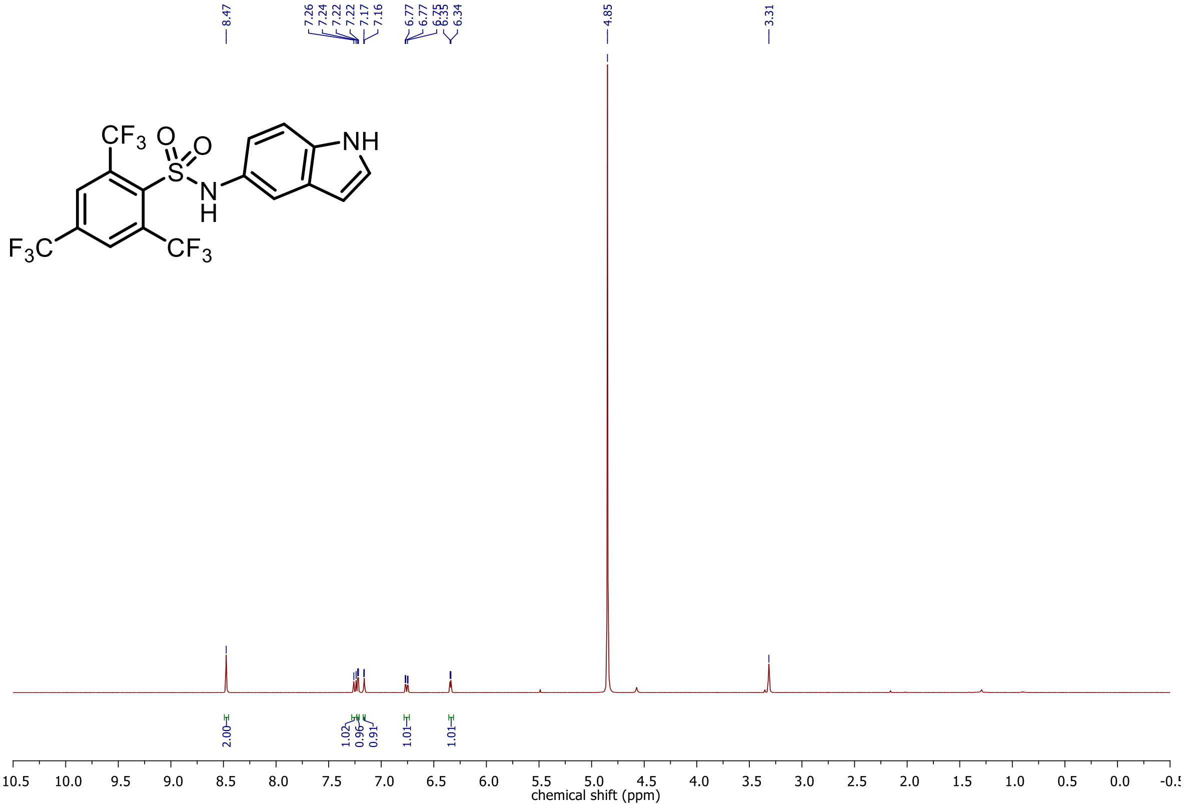

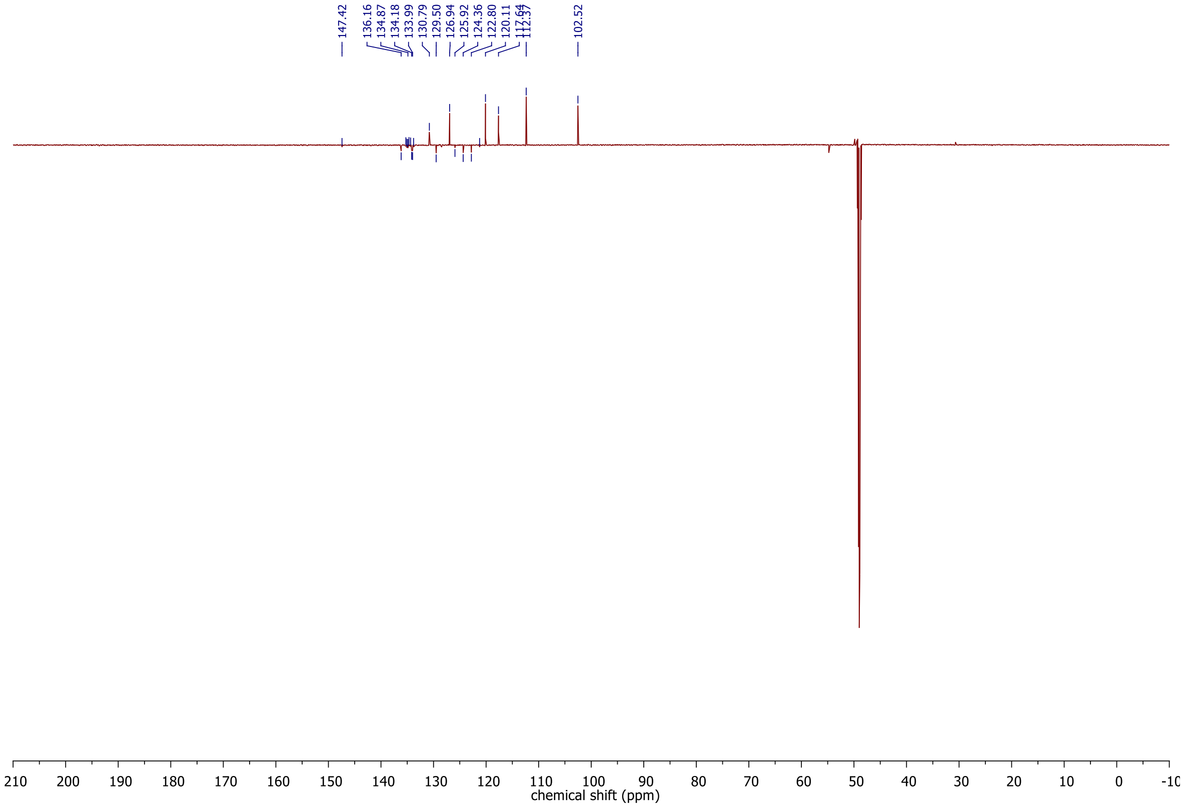

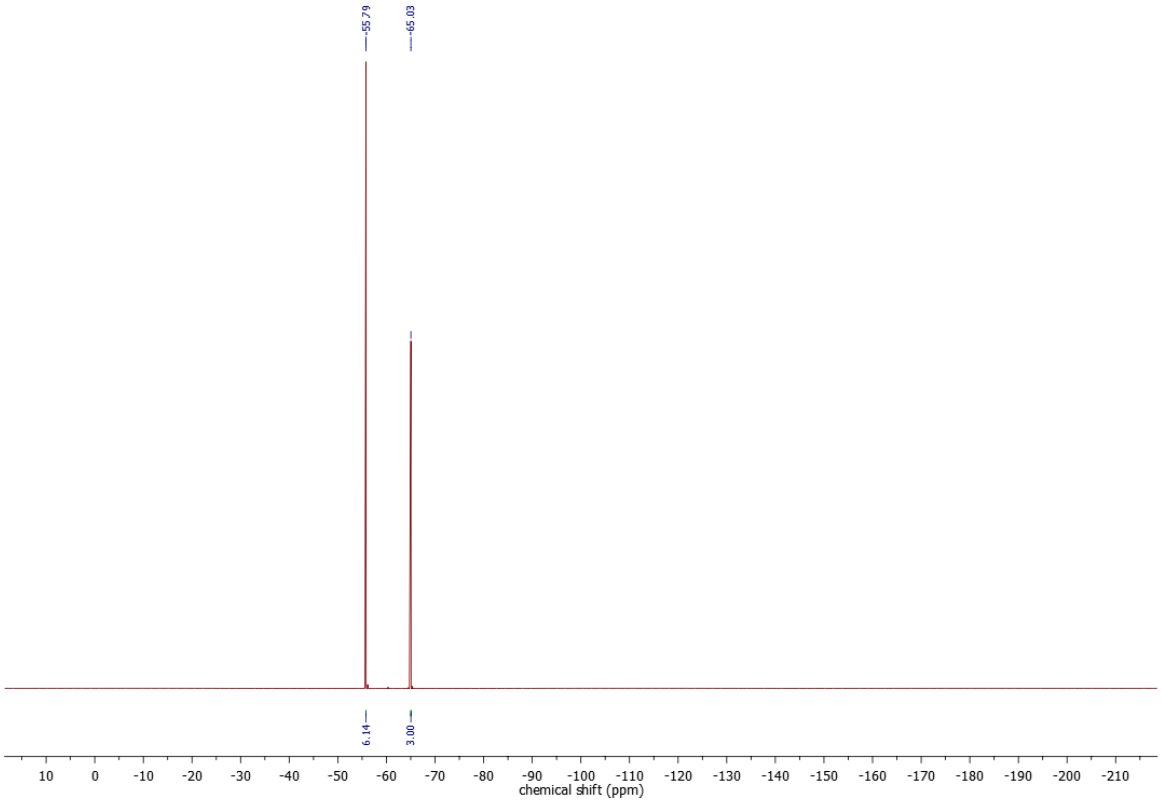

-55.79
-65.03
6.14
3.00
10 0 -10 -20 -30 -40 -50 -60 -70 -80 -90 -100 -110 -120 -130 -140 -150 -160 -170 -180 -190 -200 -210
chemical shift (ppm)

Methyl ((2,4,6-tris(trifluoromethyl)phenyl)sulfonyl)-*L*-isoleucinate (2v)

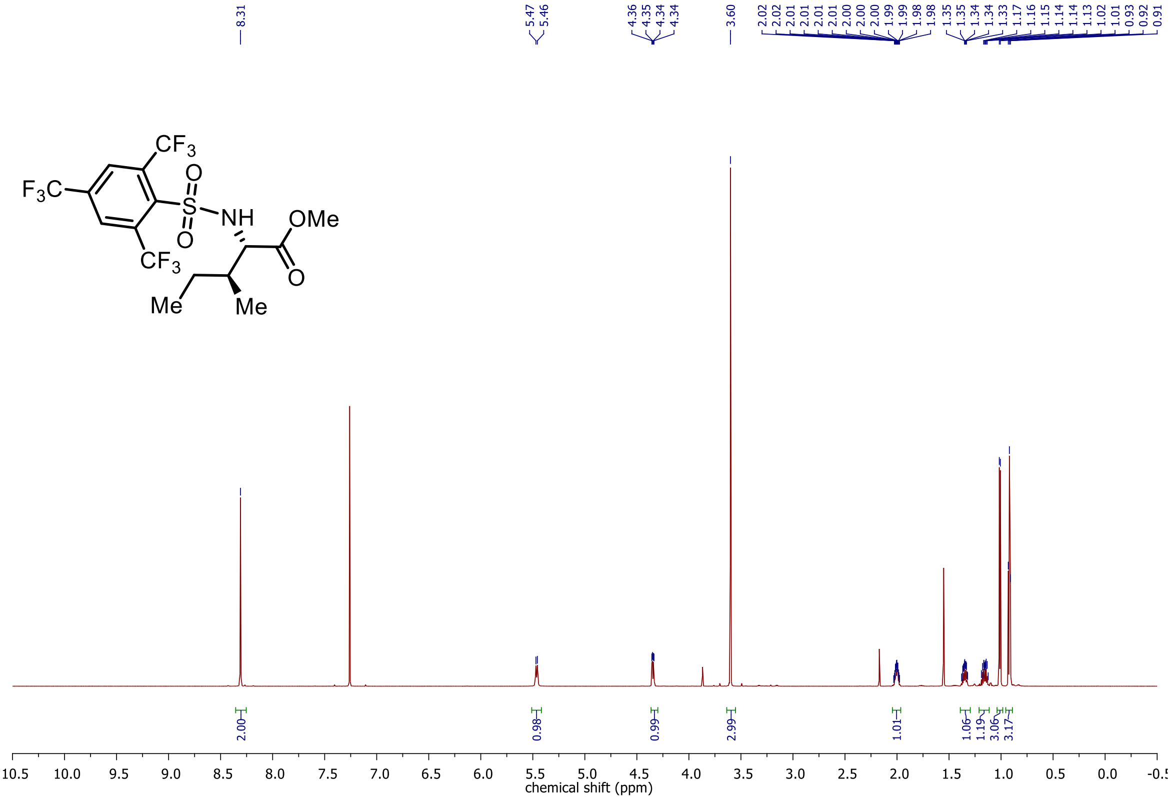

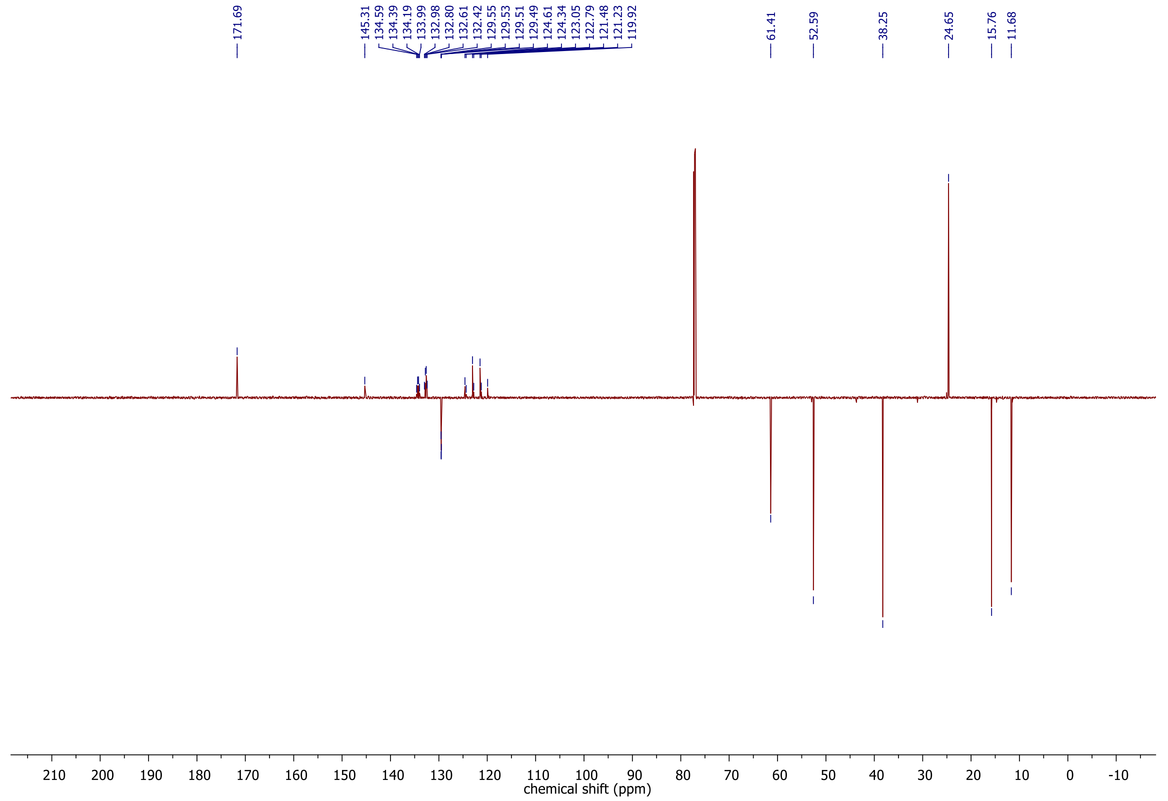

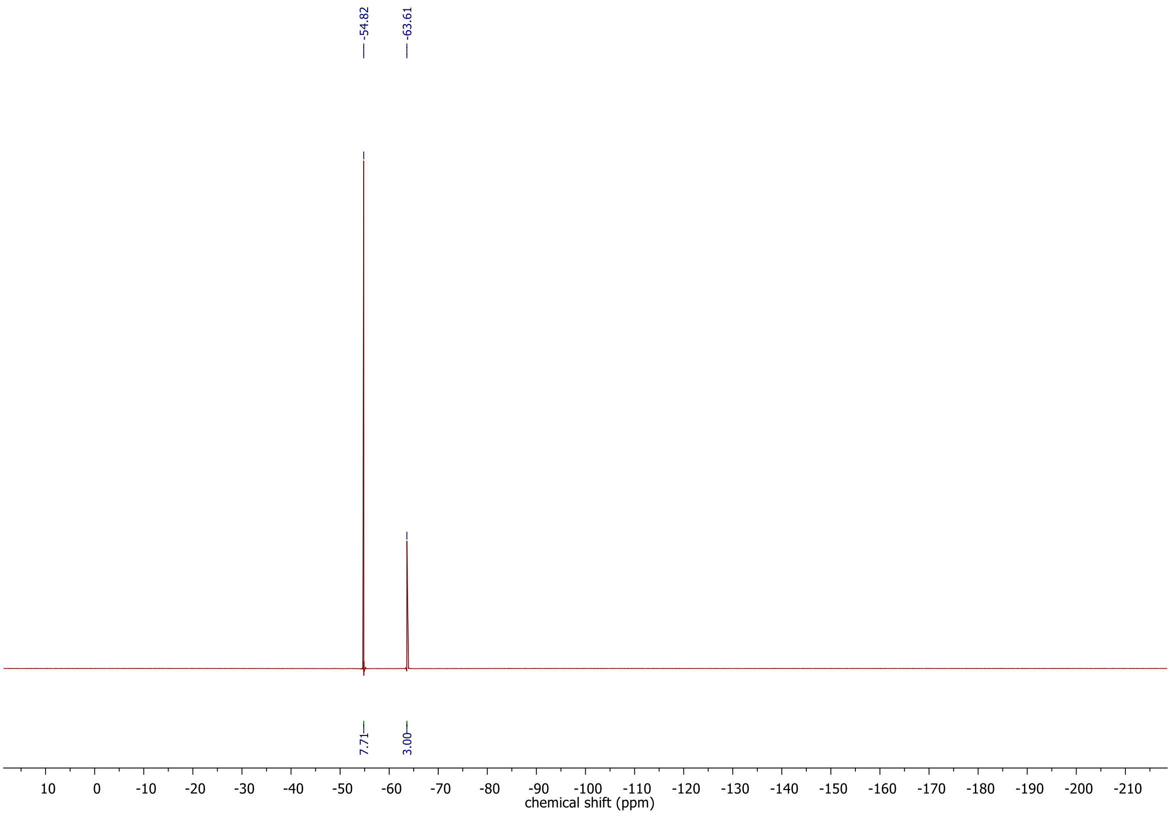
-54.82
-63.61
7.71
3.00
10 0 -10 -20 -30 -40 -50 -60 -70 -80 -90 -100 -110 -120 -130 -140 -150 -160 -170 -180 -190 -200 -210
chemical shift (ppm)

Benzyl ((2,4,6-tris(trifluoromethyl)phenyl)sulfonyl)-L-serinate (2w)

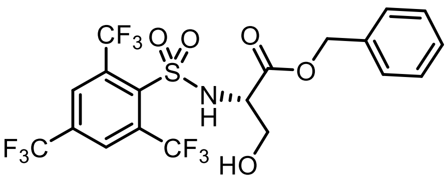

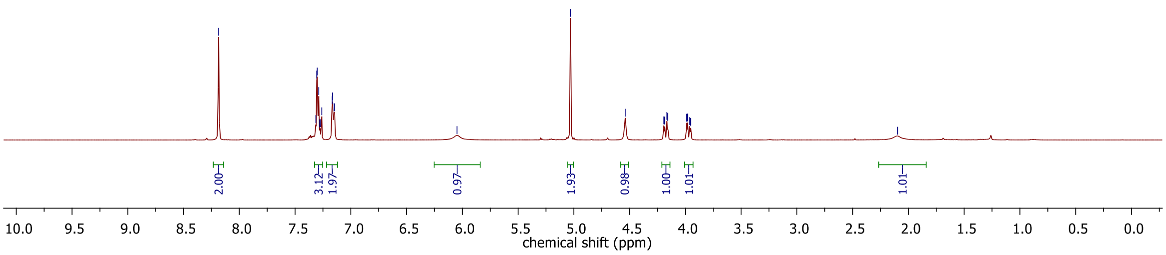

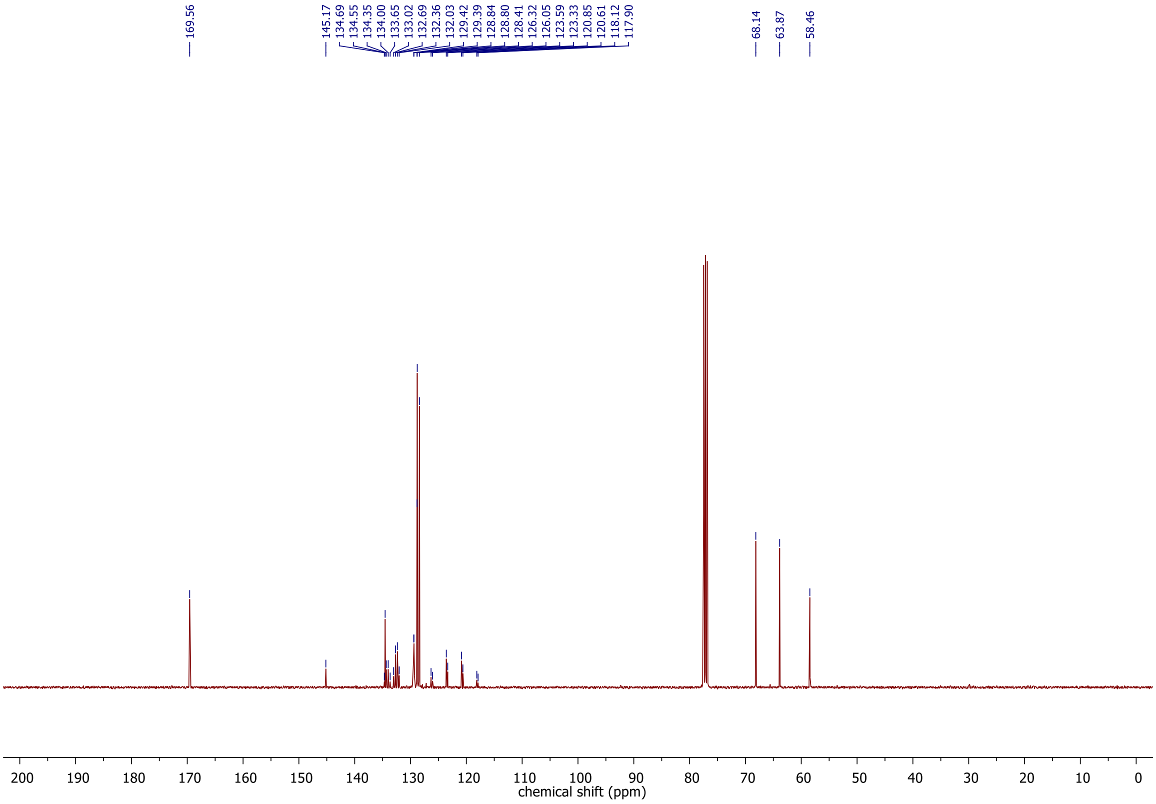

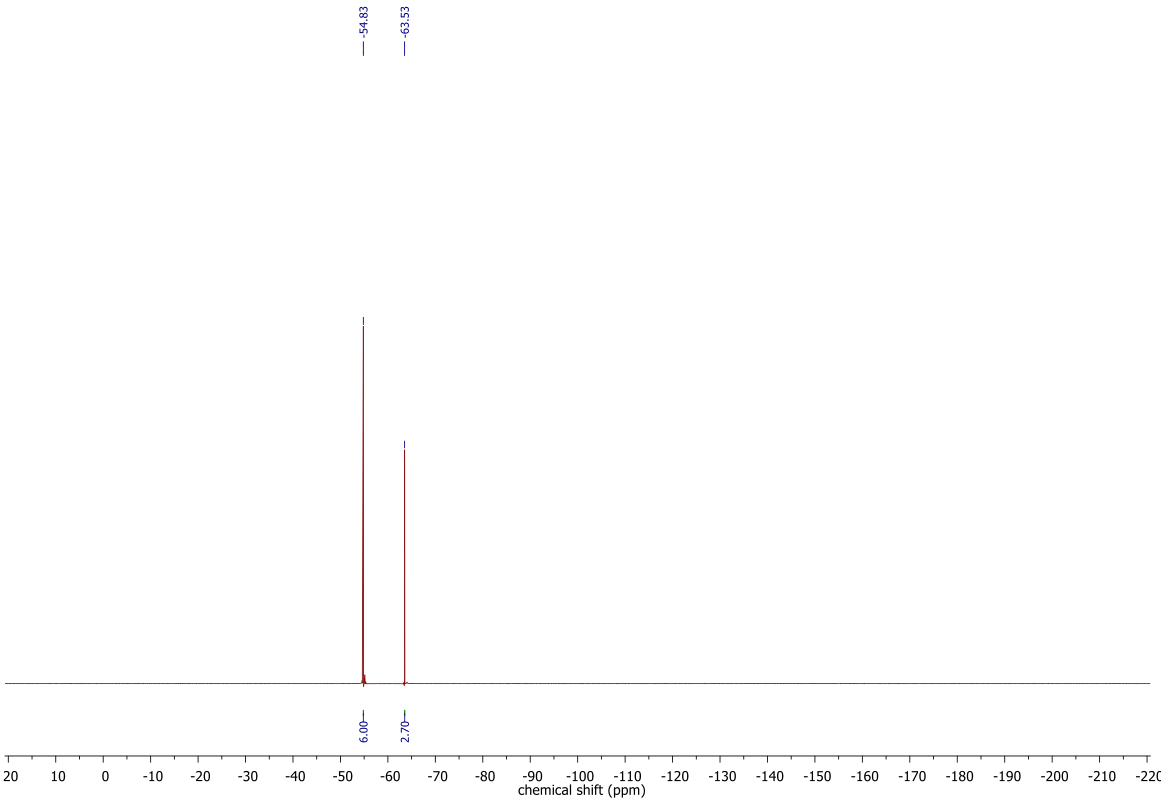
-54.83
-63.53
6.00
2.70
20 10 0 -10 -20 -30 -40 -50 -60 -70 -80 -90 -100 -110 -120 -130 -140 -150 -160 -170 -180 -190 -200 -210 -220
chemical shift (ppm)

Methyl ((2,4,6-tris(trifluoromethyl)phenyl)sulfonyl)-*L*-serinate (2x)

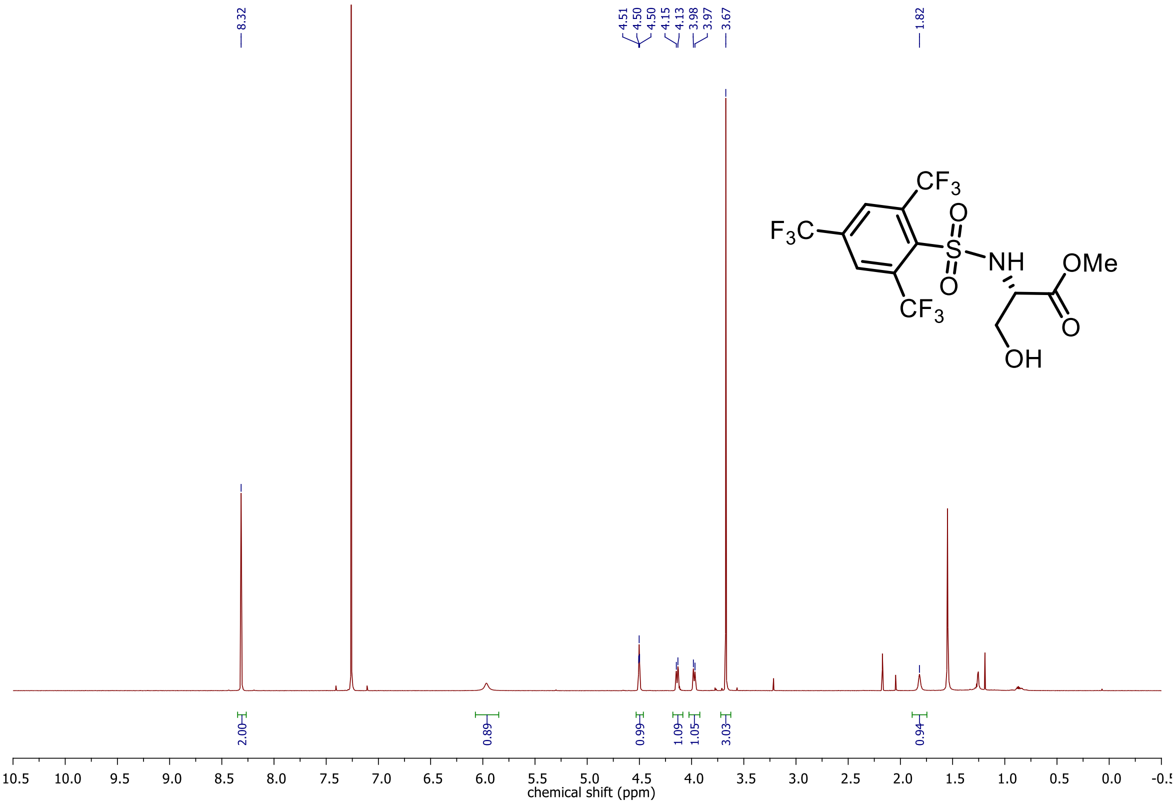

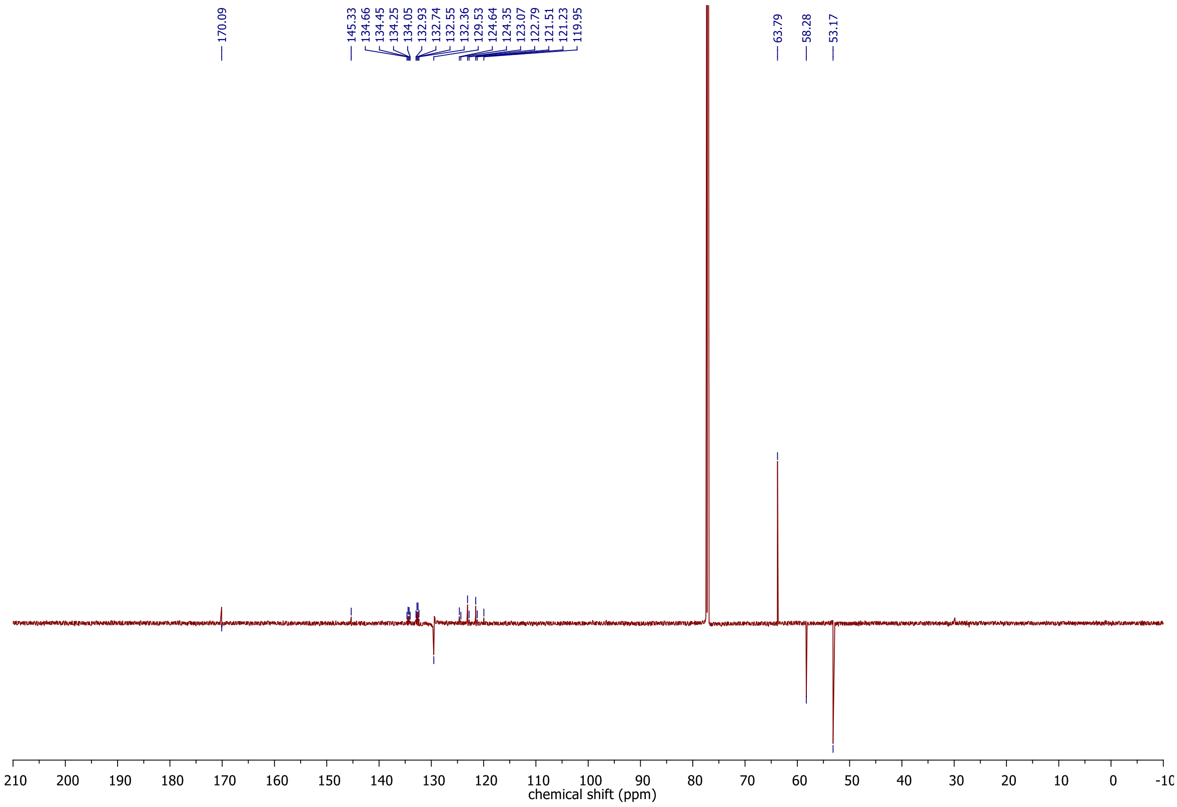

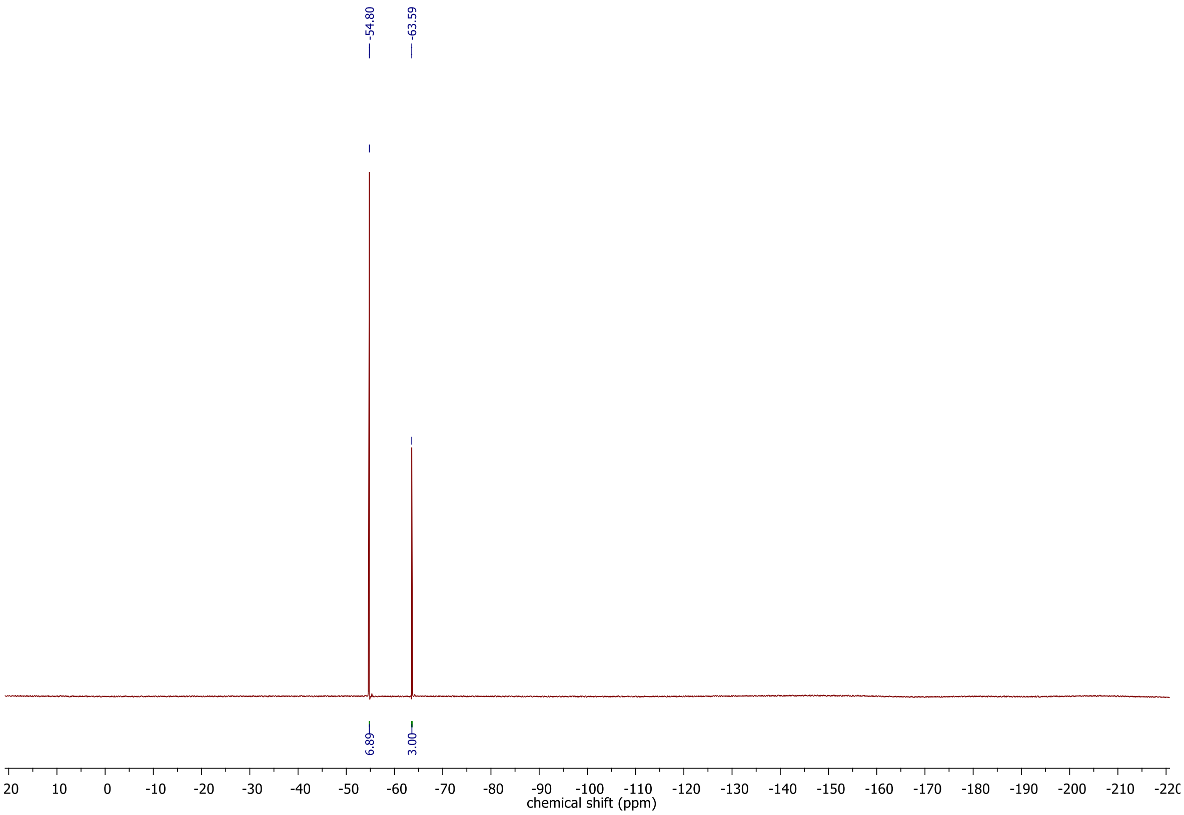
-54.80
-63.59
6.89
3.00
20
10
0
-10
-20
-30
-40
-50
-60
-70
-80
-90
-100
-110
-120
-130
-140
-150
-160
-170
-180
-190
-200
-210
-220
chemical shift (ppm)

Ethyl 1-((2,4,6-tris(trifluoromethyl)phenyl)sulfonyl)piperidine-4-carboxylate (2y)

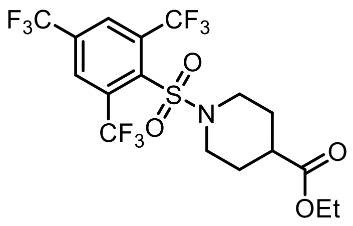

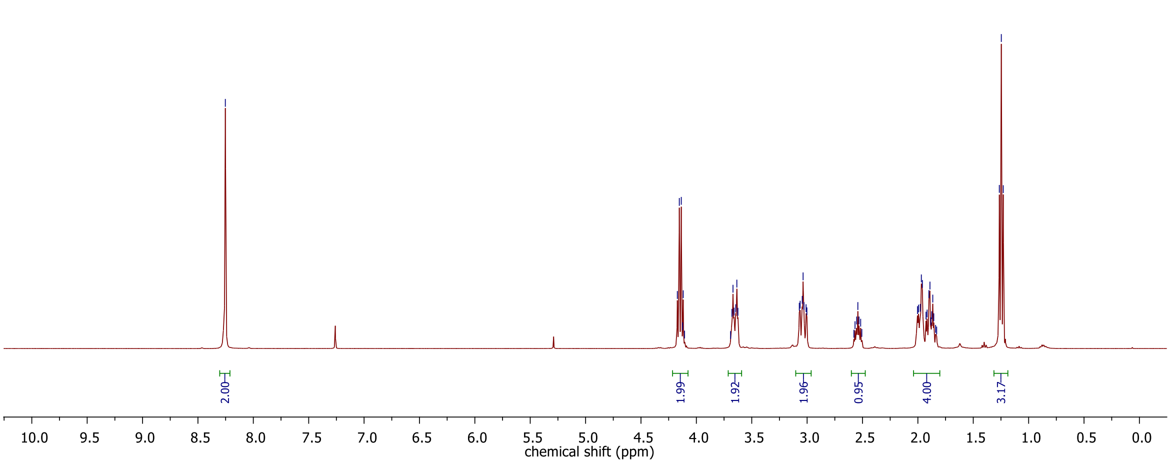

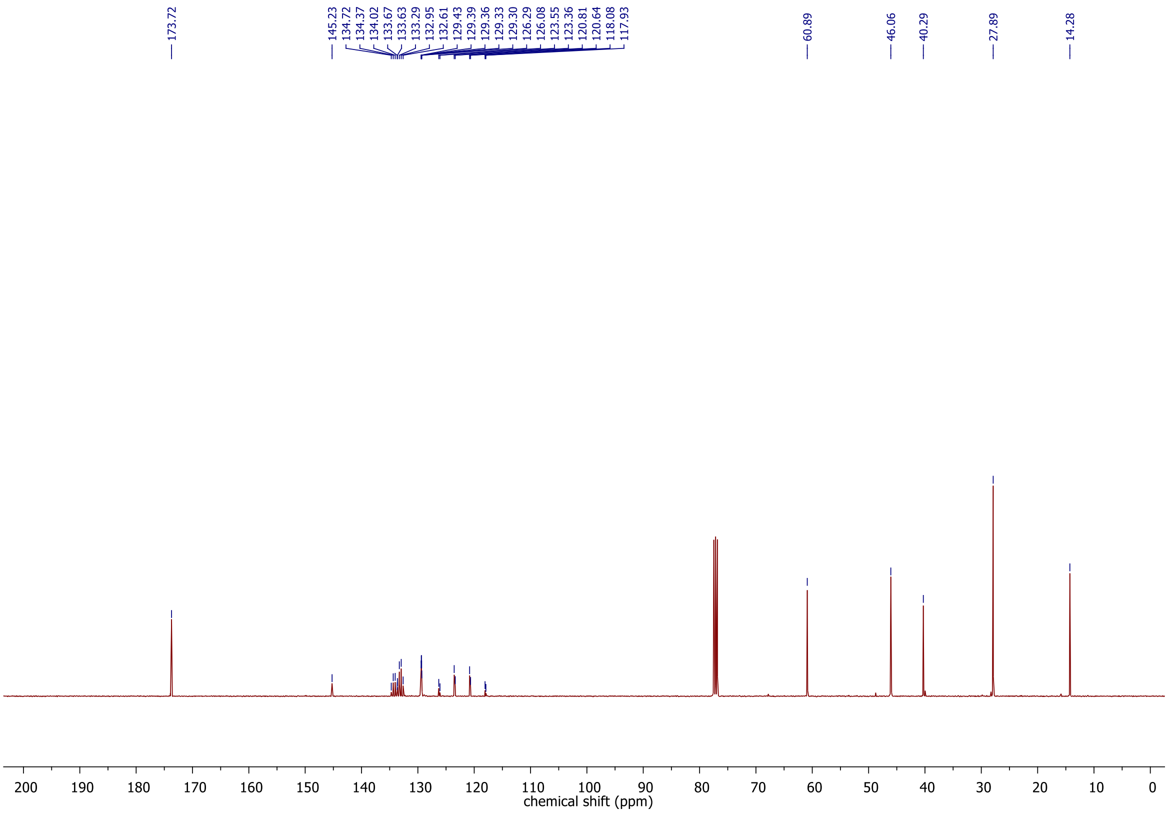

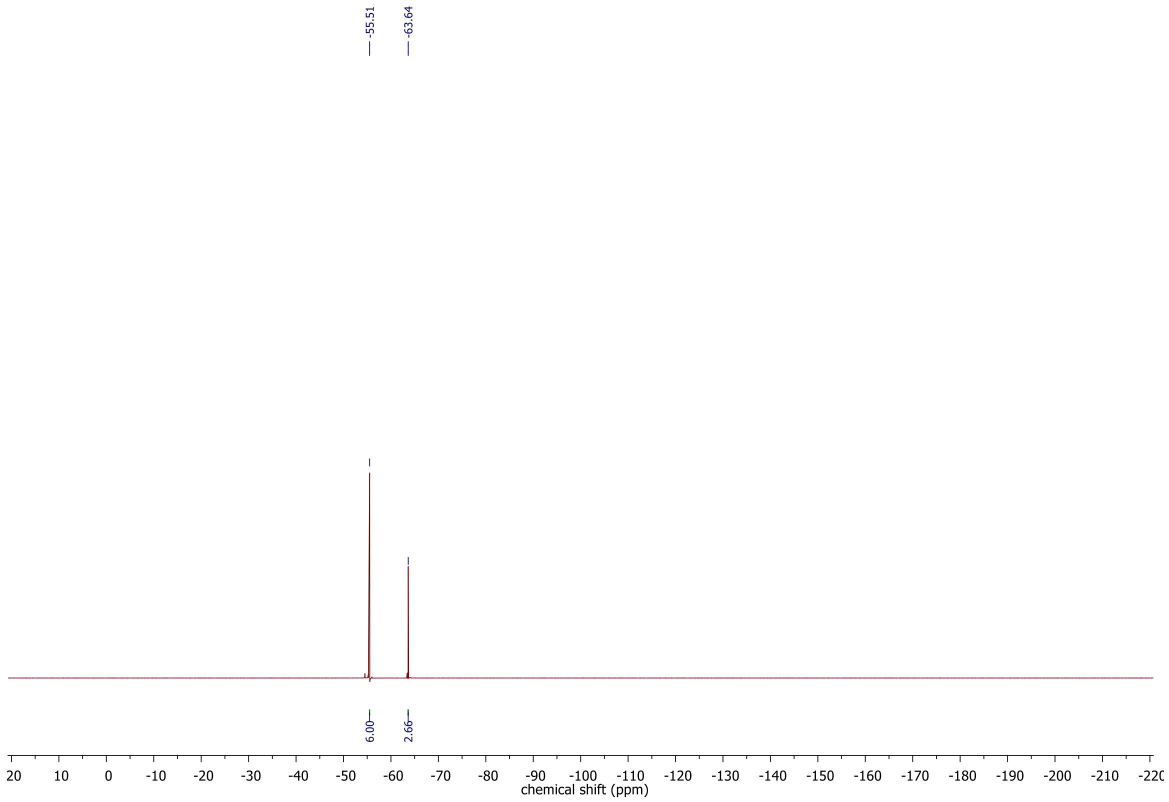

-55.51
-63.64
6.00
2.66
20 10 0 -10 -20 -30 -40 -50 -60 -70 -80 -90 -100 -110 -120 -130 -140 -150 -160 -170 -180 -190 -200 -210 -220
chemical shift (ppm)

_N_-Methoxy-_N_-methyl-1-((2,4,6-tris(trifluoromethyl)phenyl)sulfonyl)piperidine-4-carboxamide (2z)

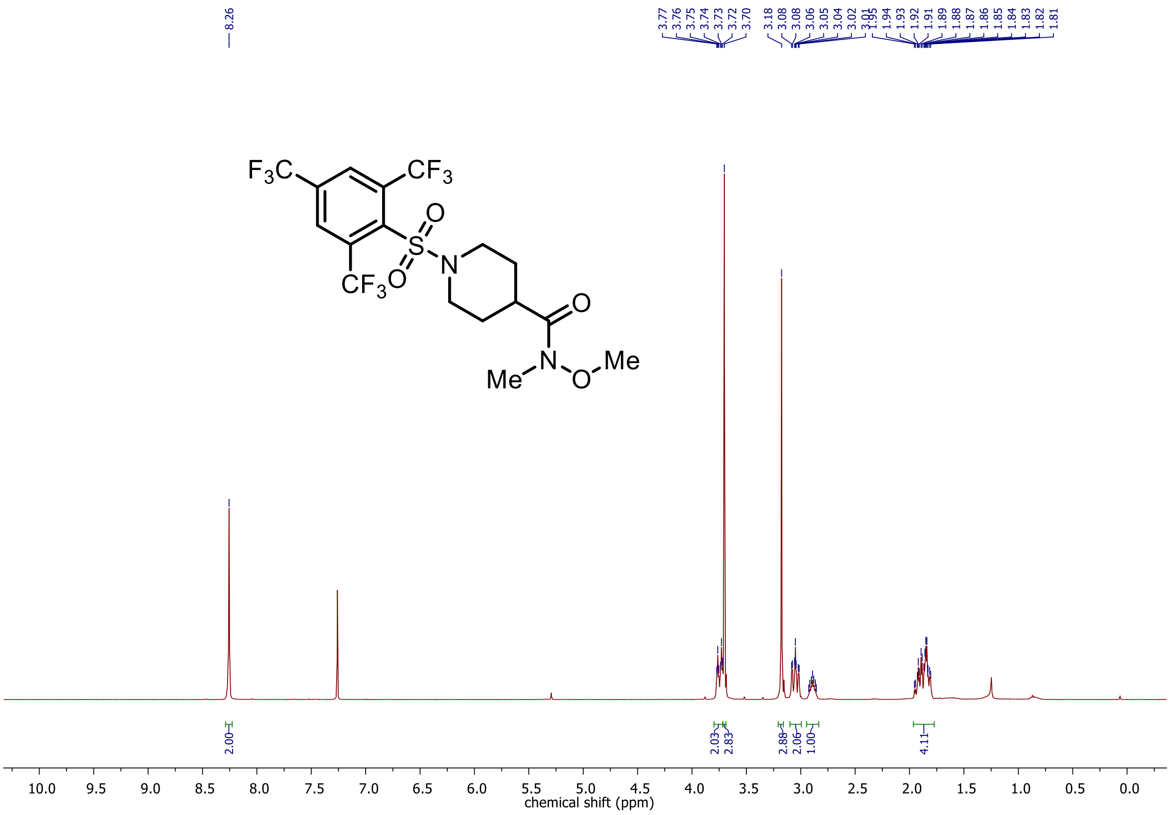

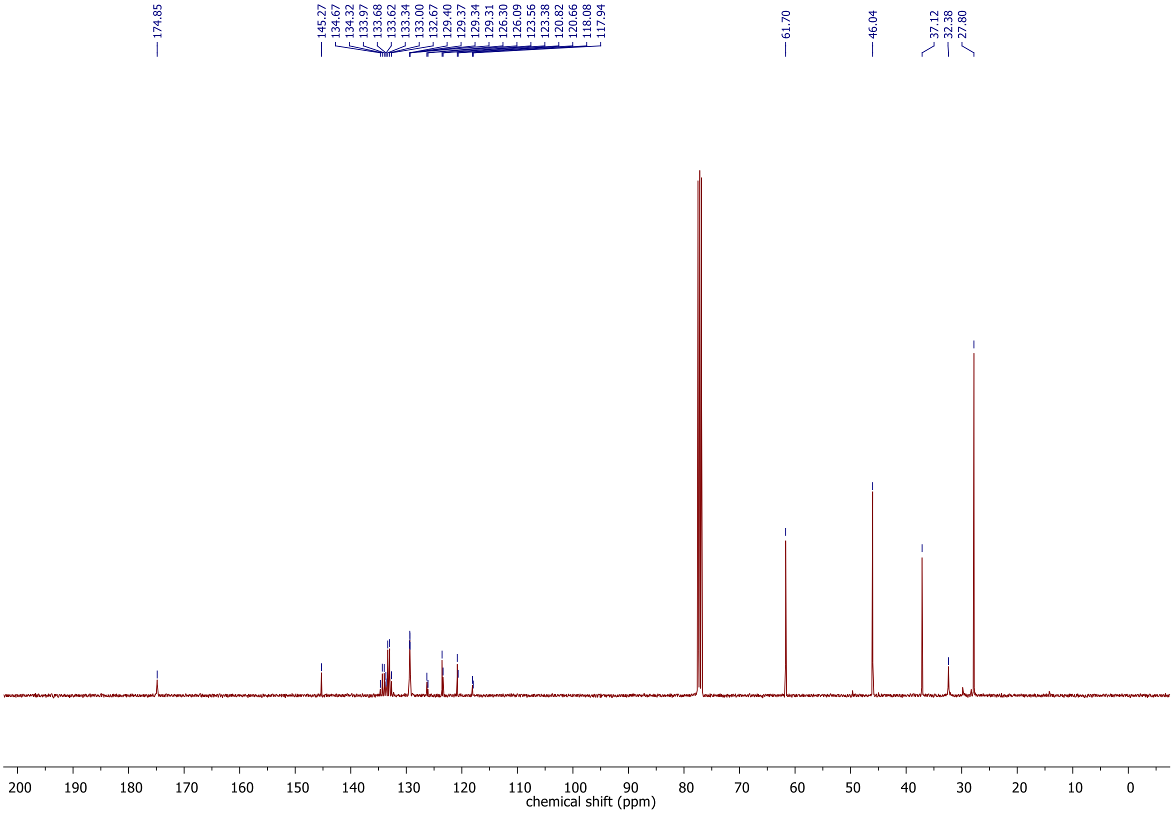

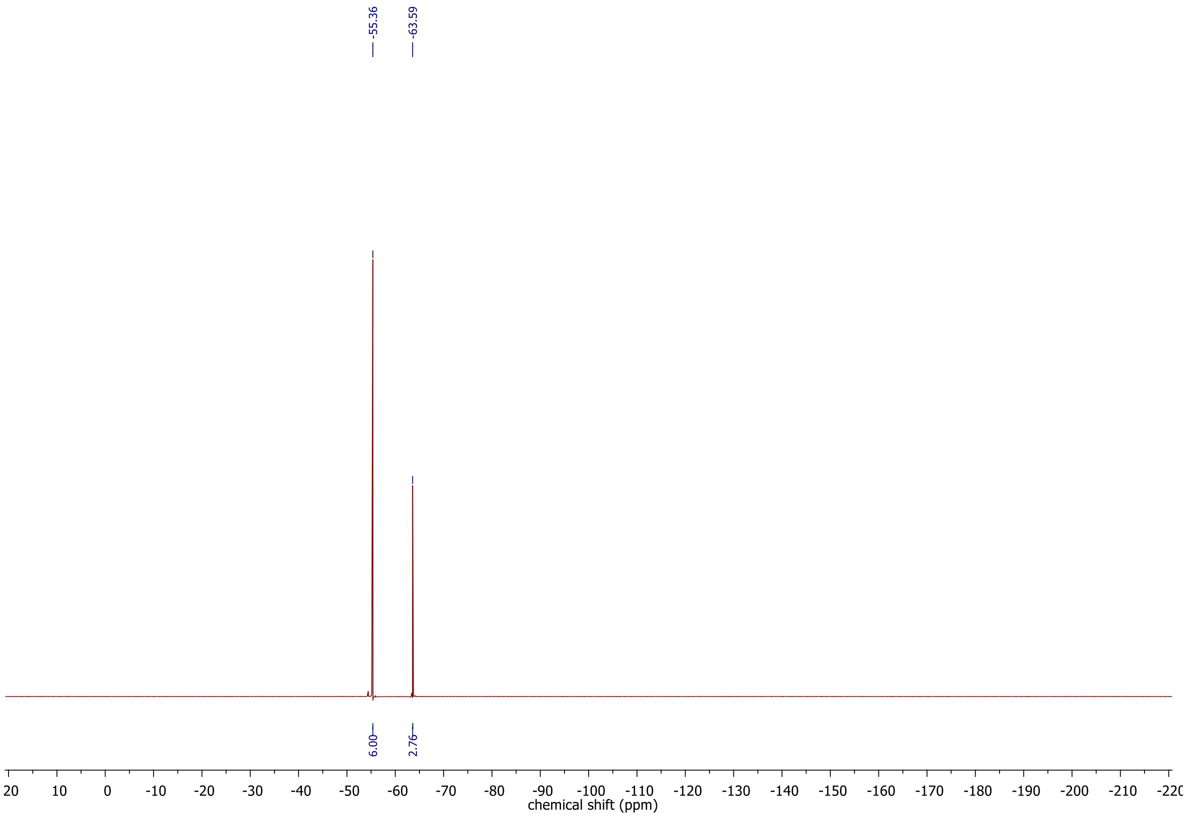

-55.36
-63.59
6.00
2.76
20 10 0 -10 -20 -30 -40 -50 -60 -70 -80 -90 -100 -110 -120 -130 -140 -150 -160 -170 -180 -190 -200 -210 -220
chemical shift (ppm)

4-Bromo-1-((2,4,6-tris(trifluoromethyl)phenyl)sulfonyl)piperidine (2aa)

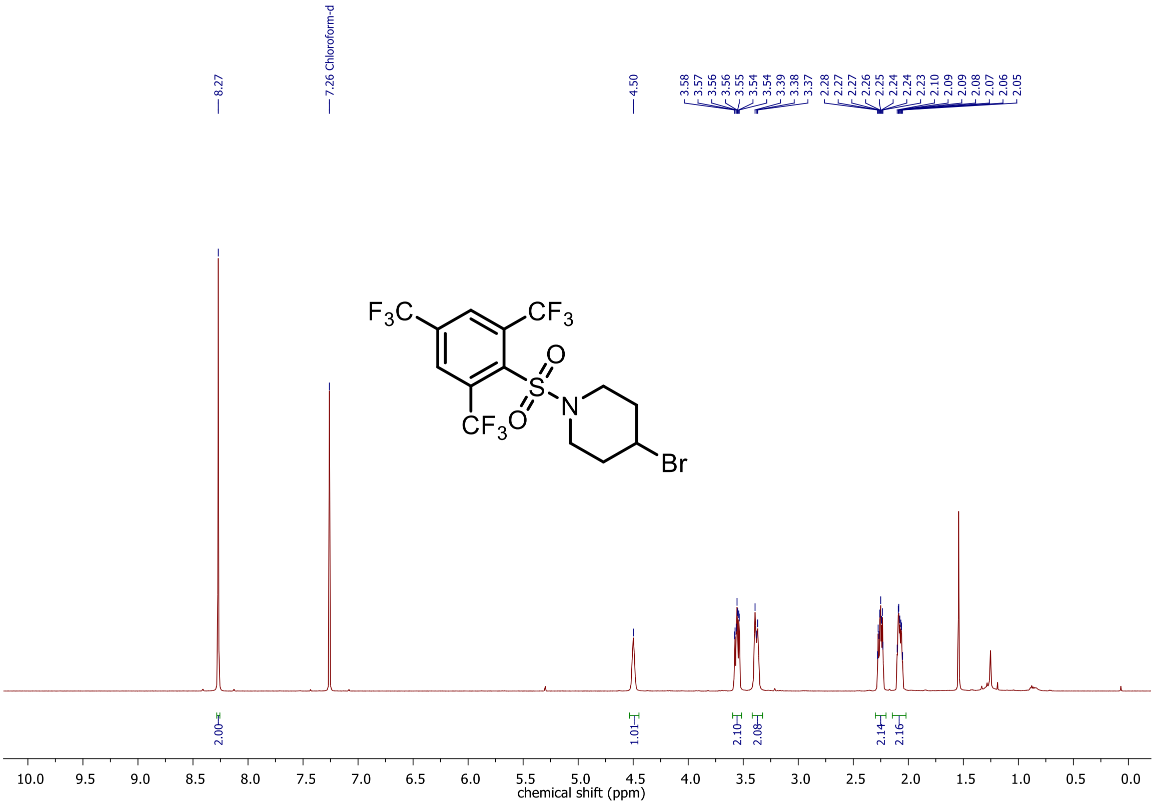

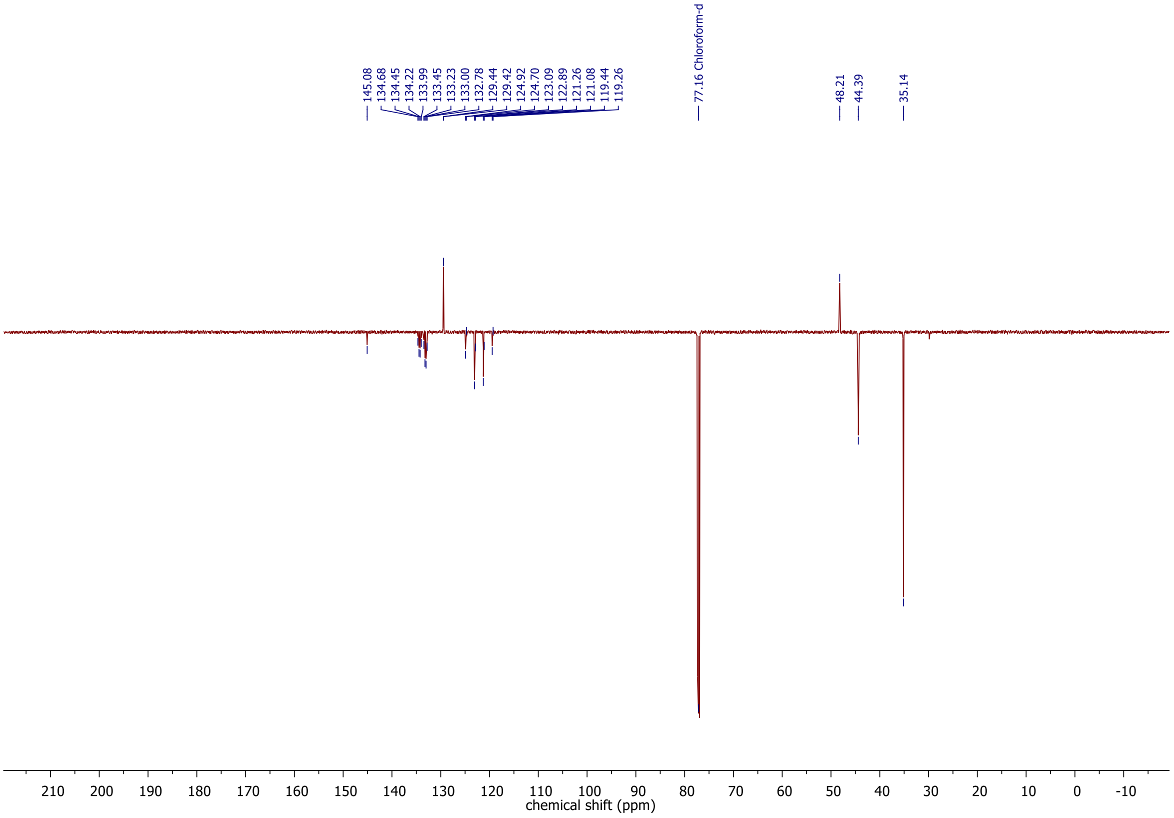

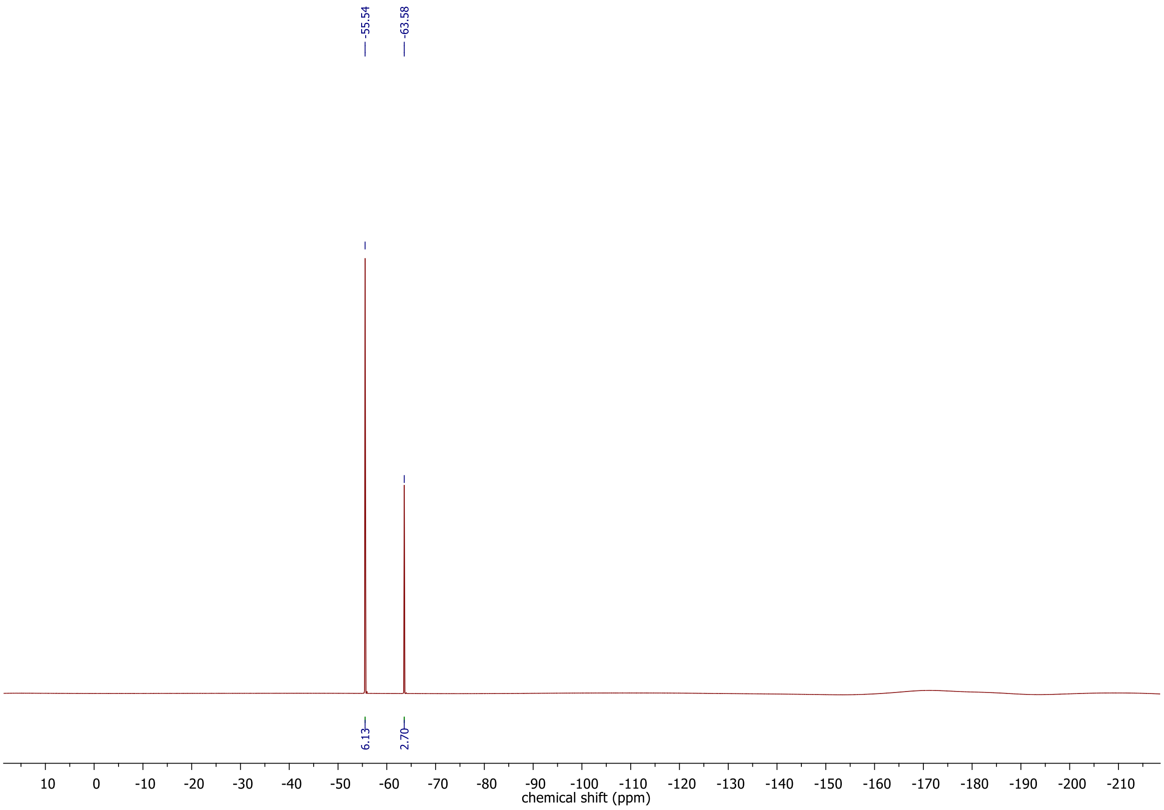
-55.54
-63.58
6.13
2.70
10 0 -10 -20 -30 -40 -50 -60 -70 -80 -90 -100 -110 -120 -130 -140 -150 -160 -170 -180 -190 -200 -210
chemical shift (ppm)

4-(4,4,5,5-Tetramethyl-1,3,2-dioxaborolan-2-yl)-1-((2,4,6 tris(trifluoromethyl)phenyl)sulfonyl)piperidine (2ab)

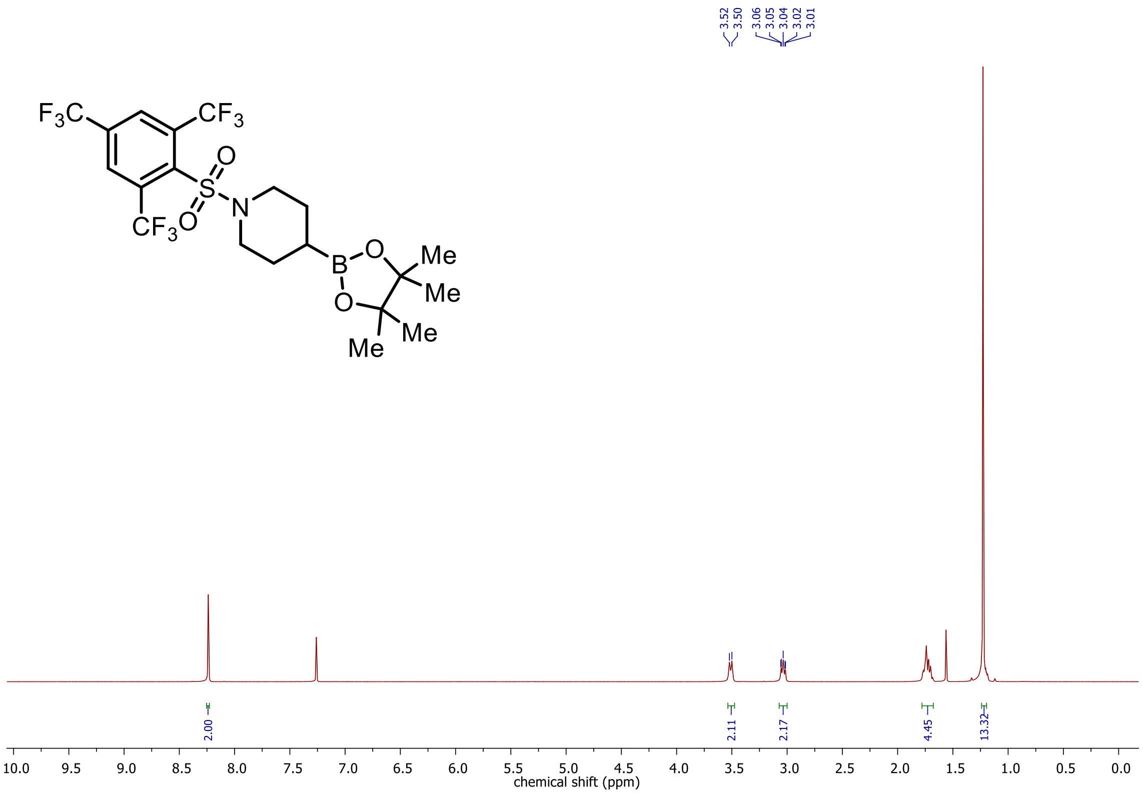

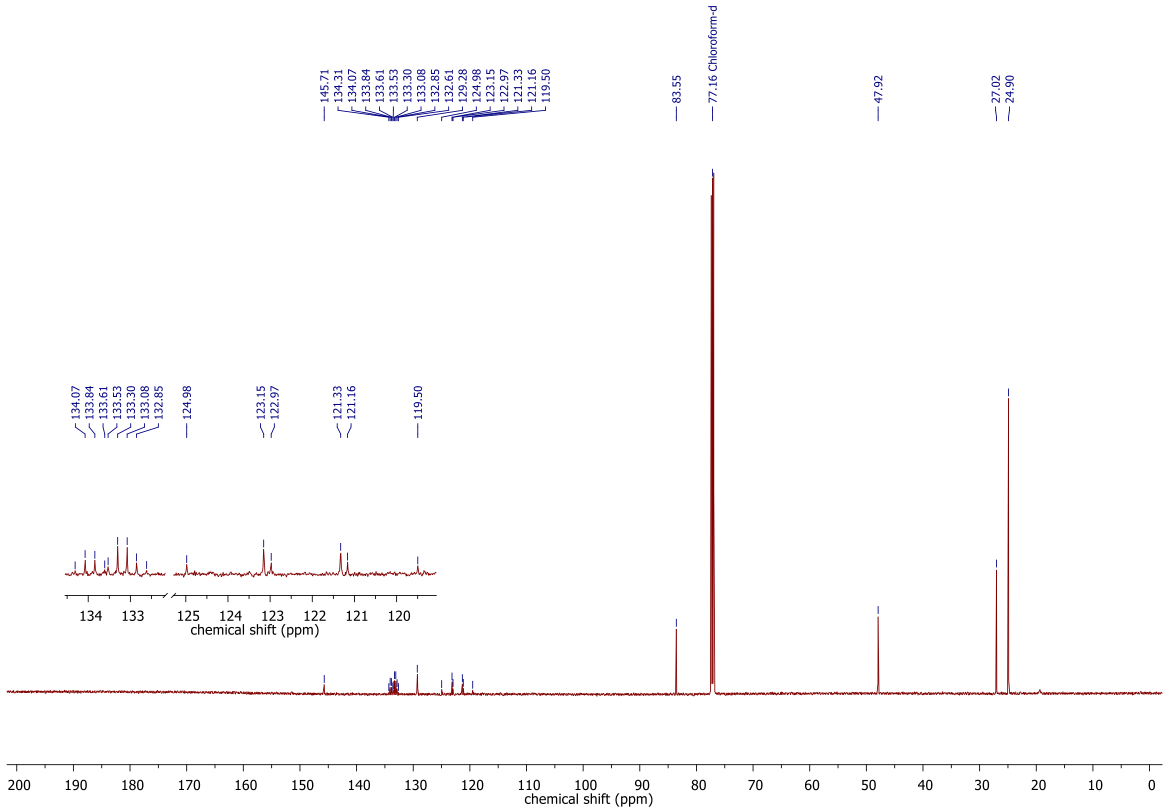

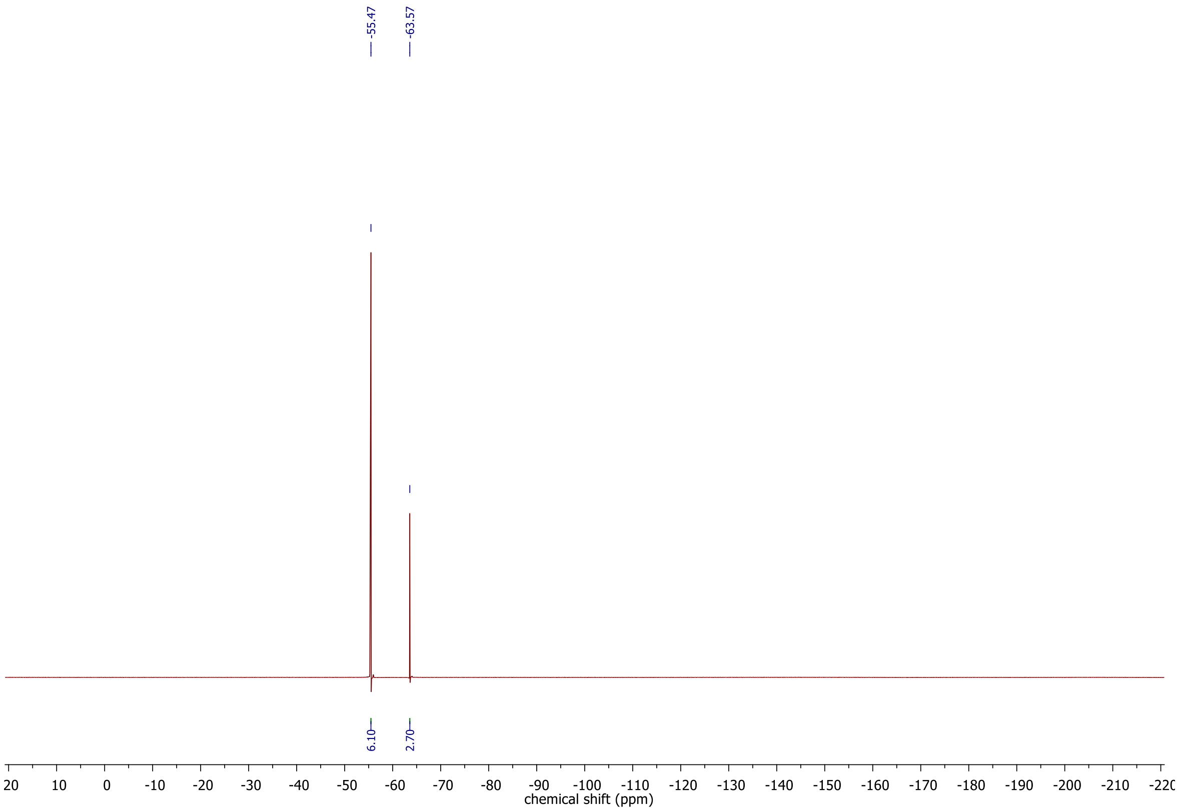
-55.47
-63.57
6.10
2.70
20 10 0 -10 -20 -30 -40 -50 -60 -70 -80 -90 -100 -110 -120 -130 -140 -150 -160 -170 -180 -190 -200 -210 -220
chemical shift (ppm)

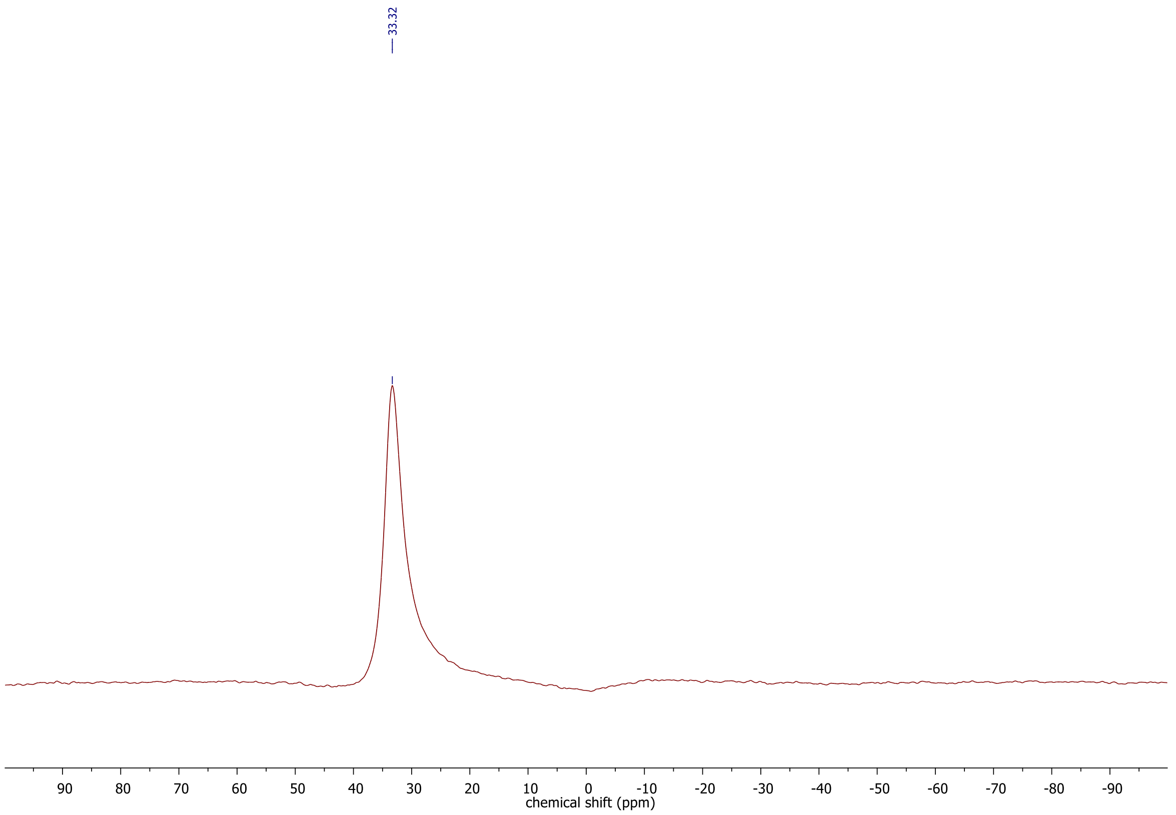
33.32
90 80 70 60 50 40 30 20 10 0 -10 -20 -30 -40 -50 -60 -70 -80 -90
chemical shift (ppm)

1-(4-Nitrophenyl)-4-((2,4,6-tris(trifluoromethyl)phenyl)sulfonyl)piperazine (2ac)

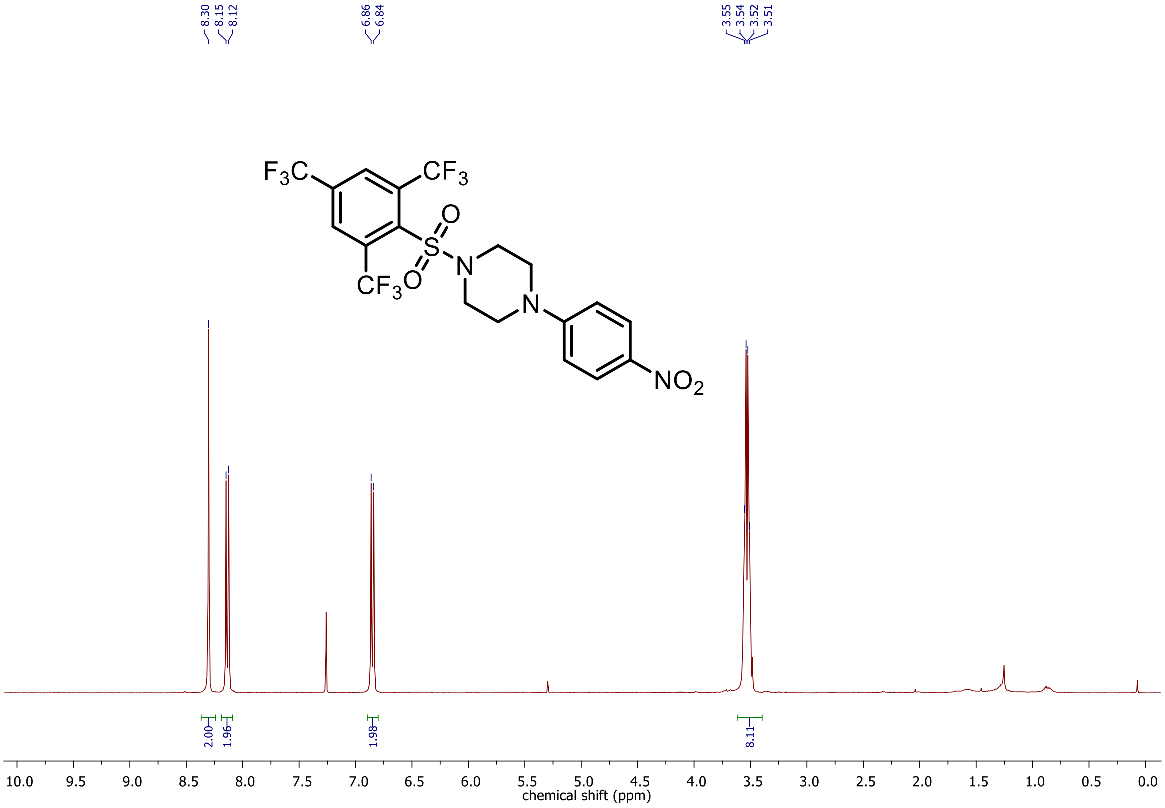

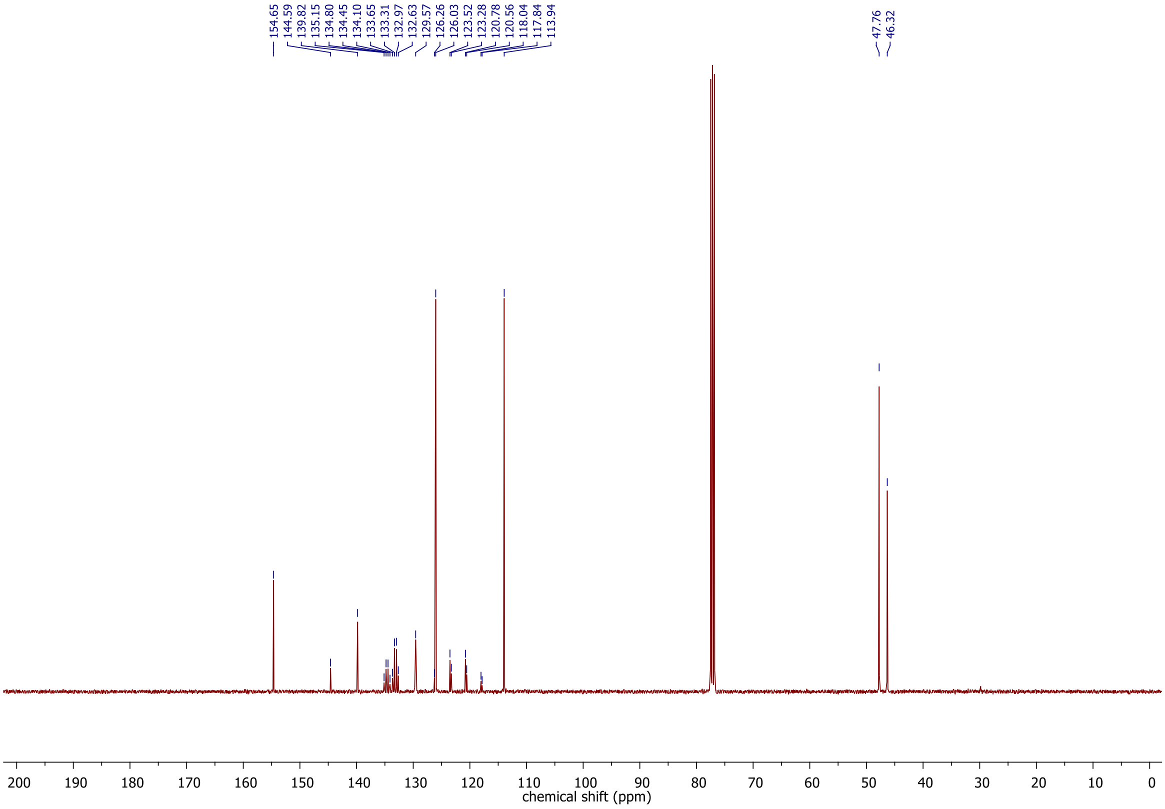

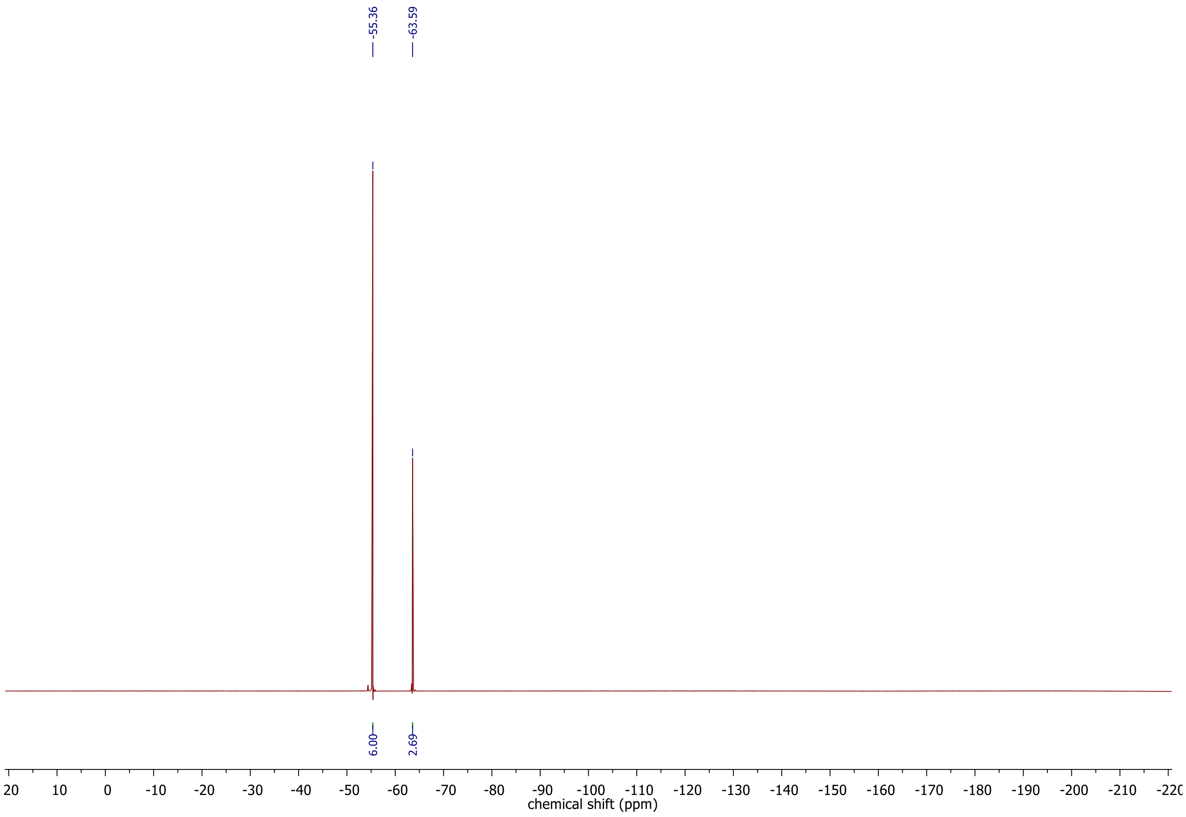
-55.36
-63.59
6.00
2.69
20 10 0 -10 -20 -30 -40 -50 -60 -70 -80 -90 -100 -110 -120 -130 -140 -150 -160 -170 -180 -190 -200 -210 -220
chemical shift (ppm)

1-(4-(4,4,5,5-Tetramethyl-1,3,2-dioxaborolan-2-yl)pyridin-2-yl)-4-((2,4,6-tris(trifluoromethyl)phenyl)sulfonyl)piperazine (2ad)

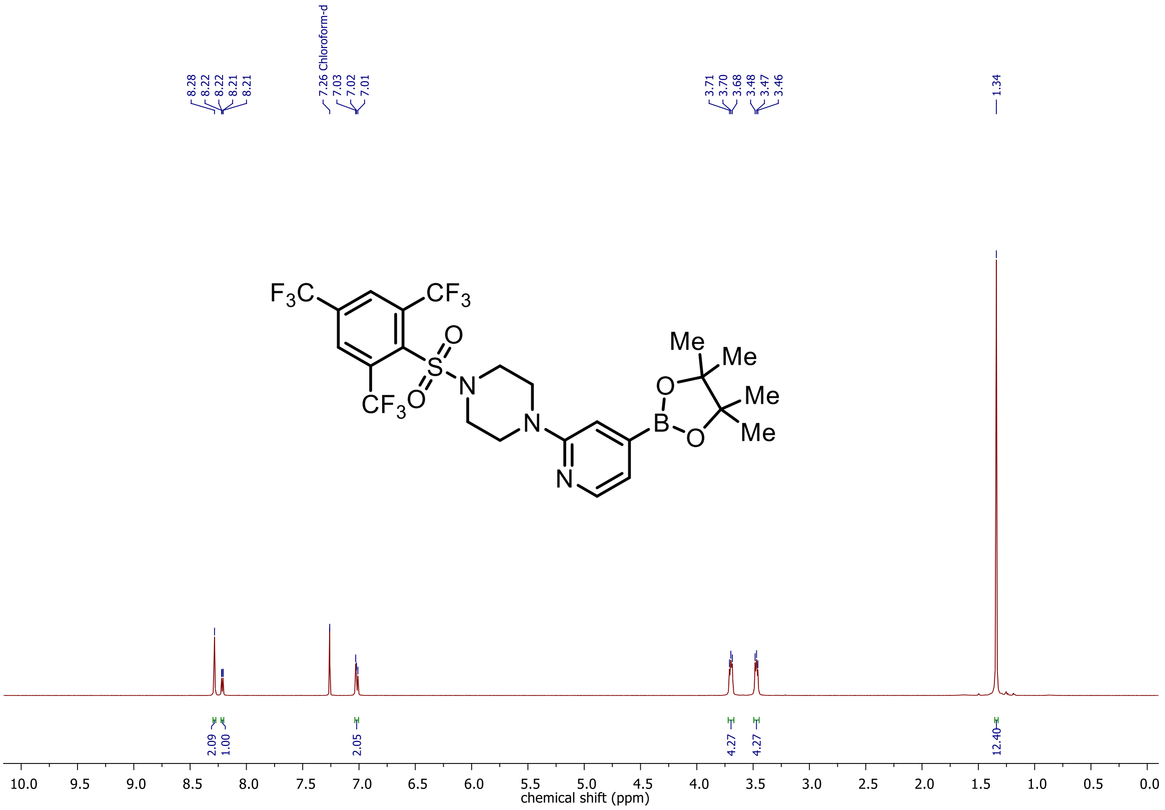

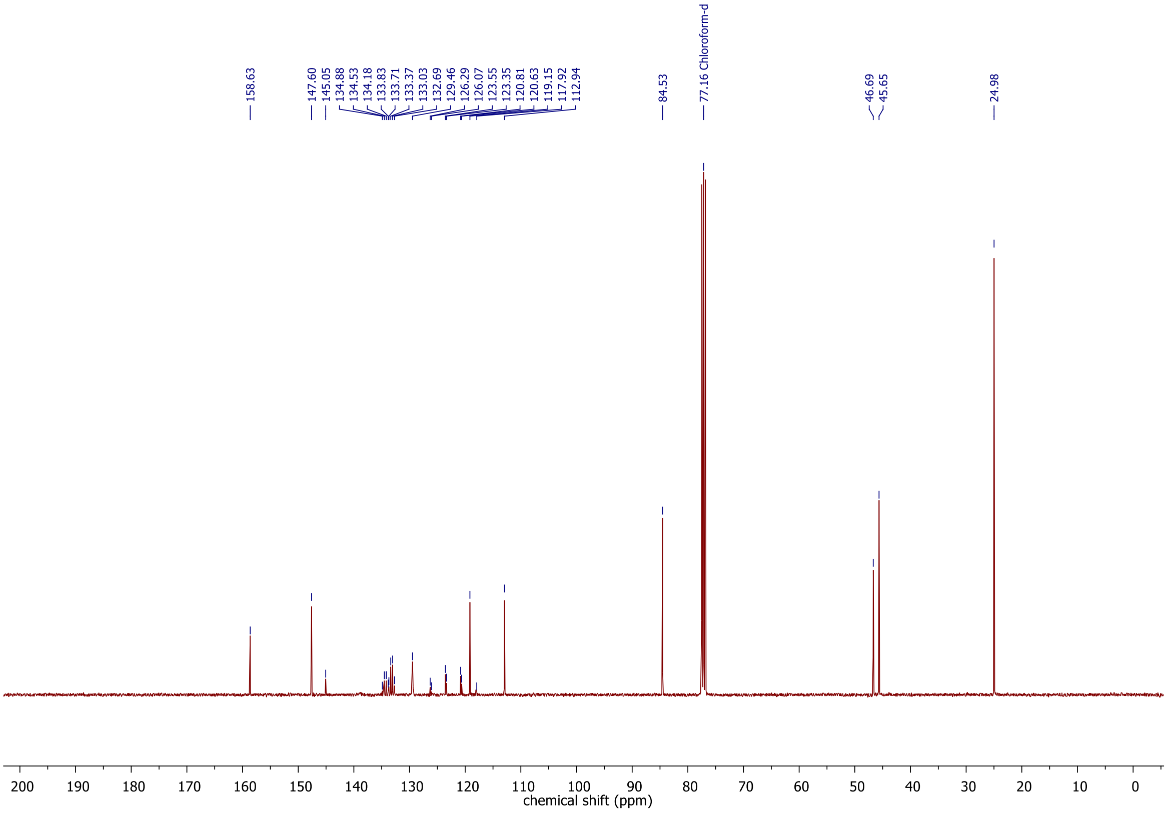

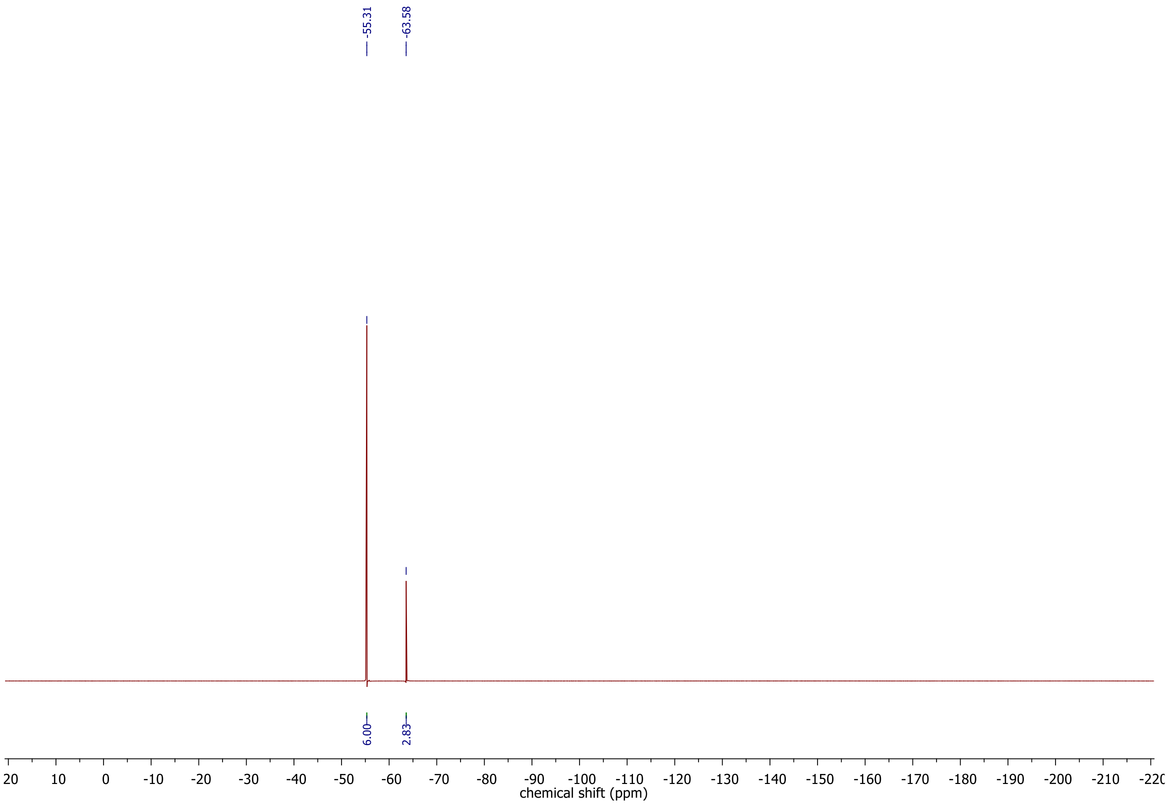

-55.31
-63.58
6.00
2.83
20 10 0 -10 -20 -30 -40 -50 -60 -70 -80 -90 -100 -110 -120 -130 -140 -150 -160 -170 -180 -190 -200 -210 -220
chemical shift (ppm)

N-(3,4-Dimethoxyphenethyl)-N-methyl-2,4,6-tris(trifluoromethyl)benzenesulfonamide (2ae)

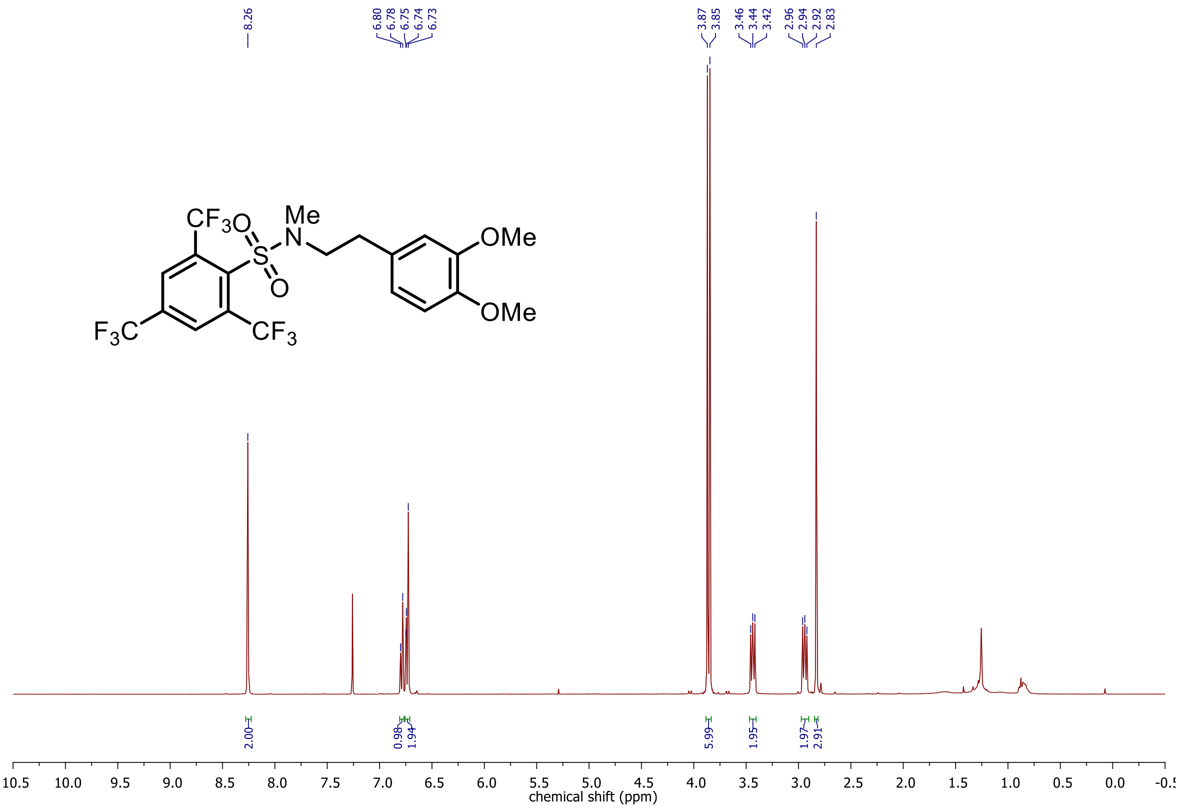

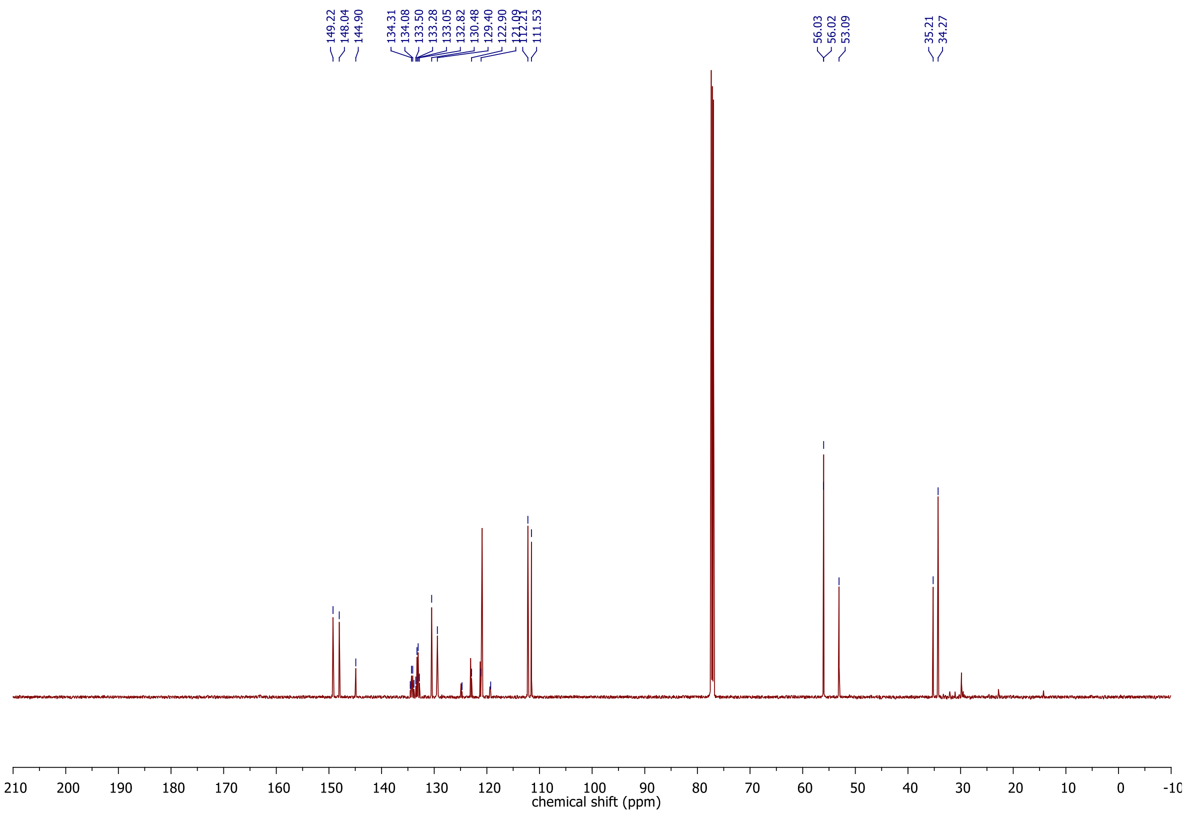

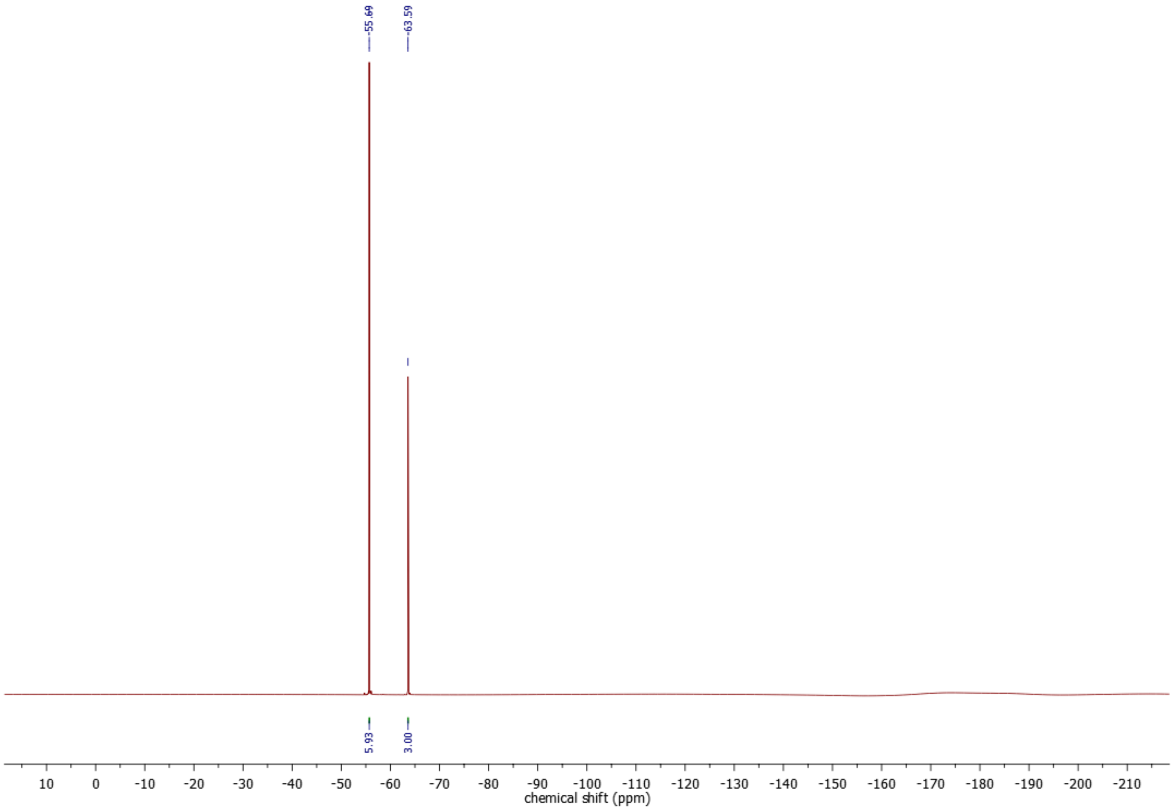

-55.69
-63.59
5.93
3.00
10 0 -10 -20 -30 -40 -50 -60 -70 -80 -90 -100 -110 -120 -130 -140 -150 -160 -170 -180 -190 -200 -210
chemical shift (ppm)

N-(3-(10,11-Dihydro-5H-dibenzo[b,f]azepin-5-yl)propyl)-N-methyl-2,4,6-tris(trifluoromethyl)benzenesulfonamide (2af)

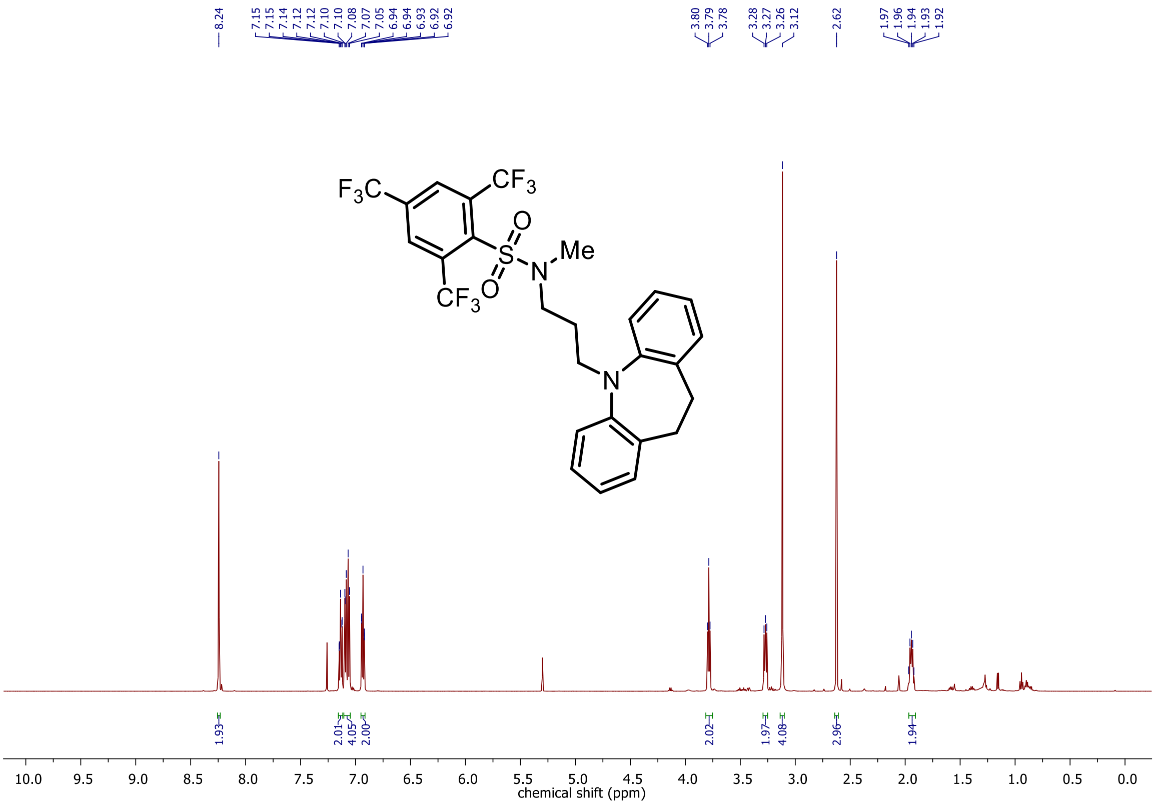

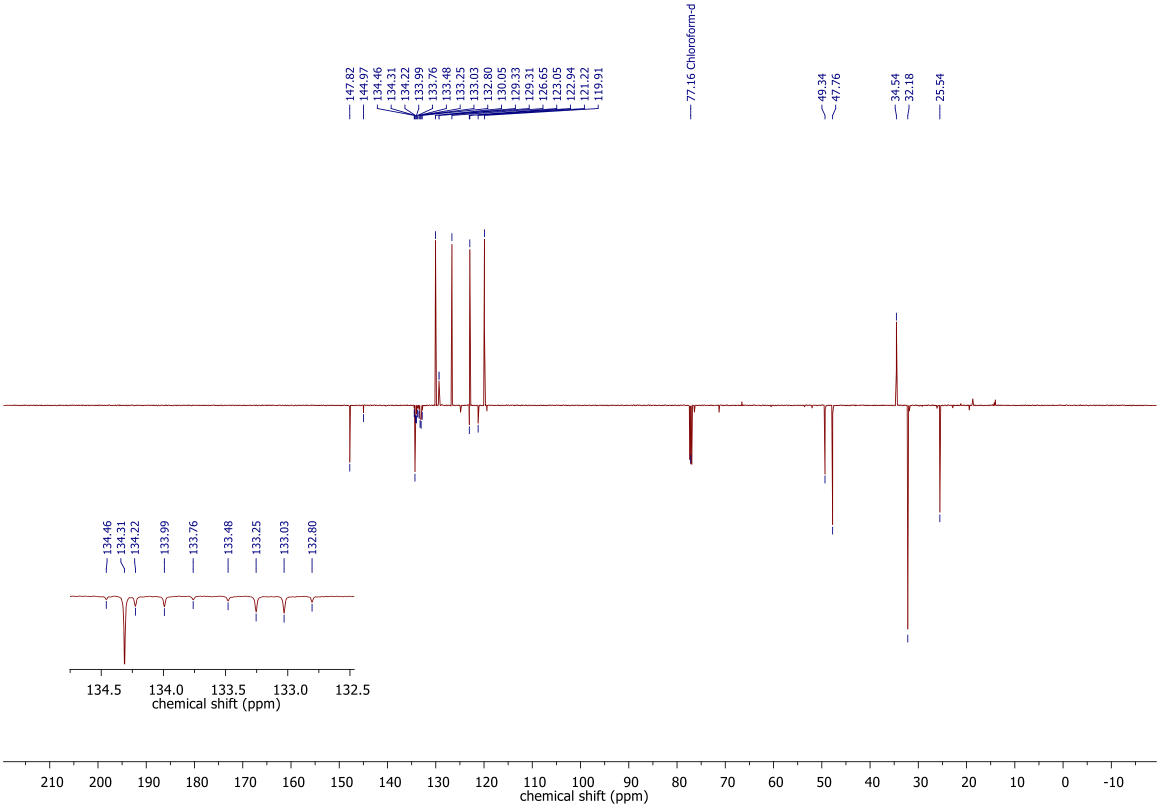

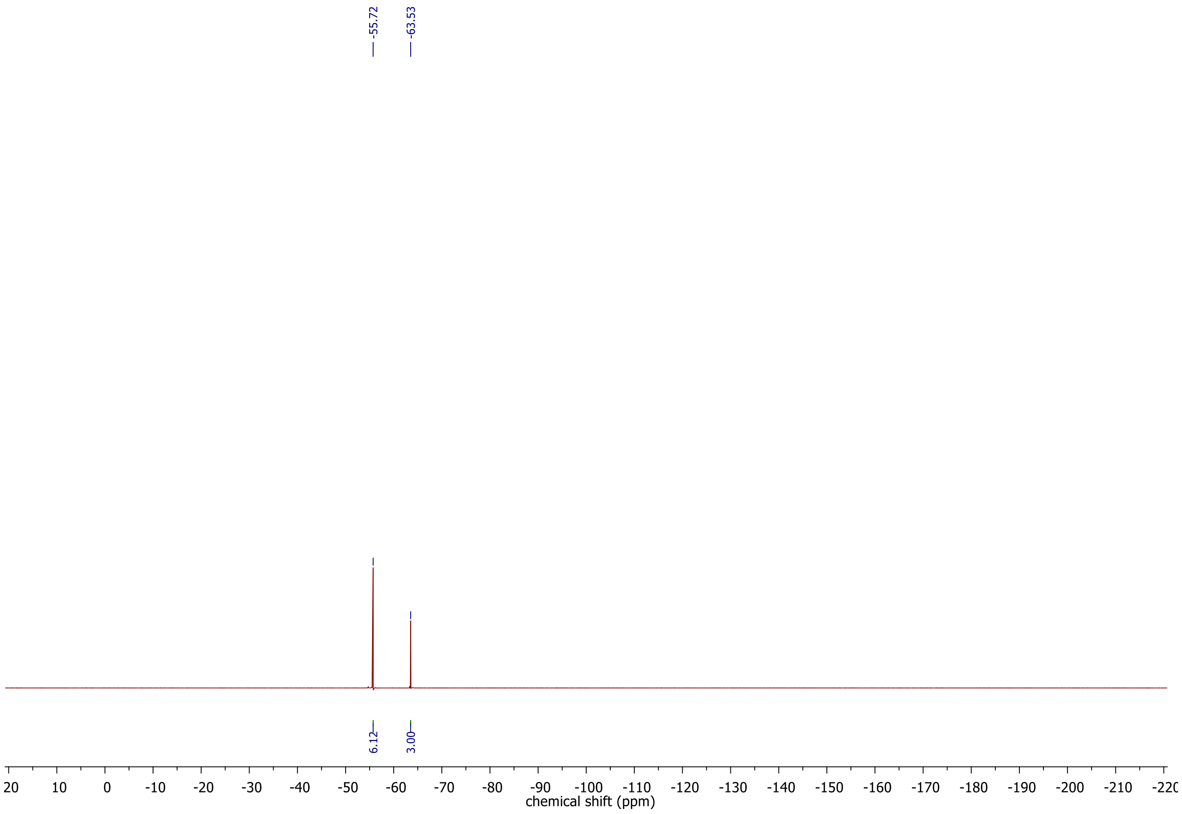

-55.72
-63.53
6.12
3.00
20
10
0
-10
-20
-30
-40
-50
-60
-70
-80
-90
-100
-110
-120
-130
-140
-150
-160
-170
-180
-190
-200
-210
-220
chemical shift (ppm)

(*S*)-*N*-methyl-*N*-(3-(naphthalen-1-yloxy)-3-(thiophen-2-yl)propyl)-2,4,6 tris(trifluoromethyl)benzenesulfonamide (2ag)

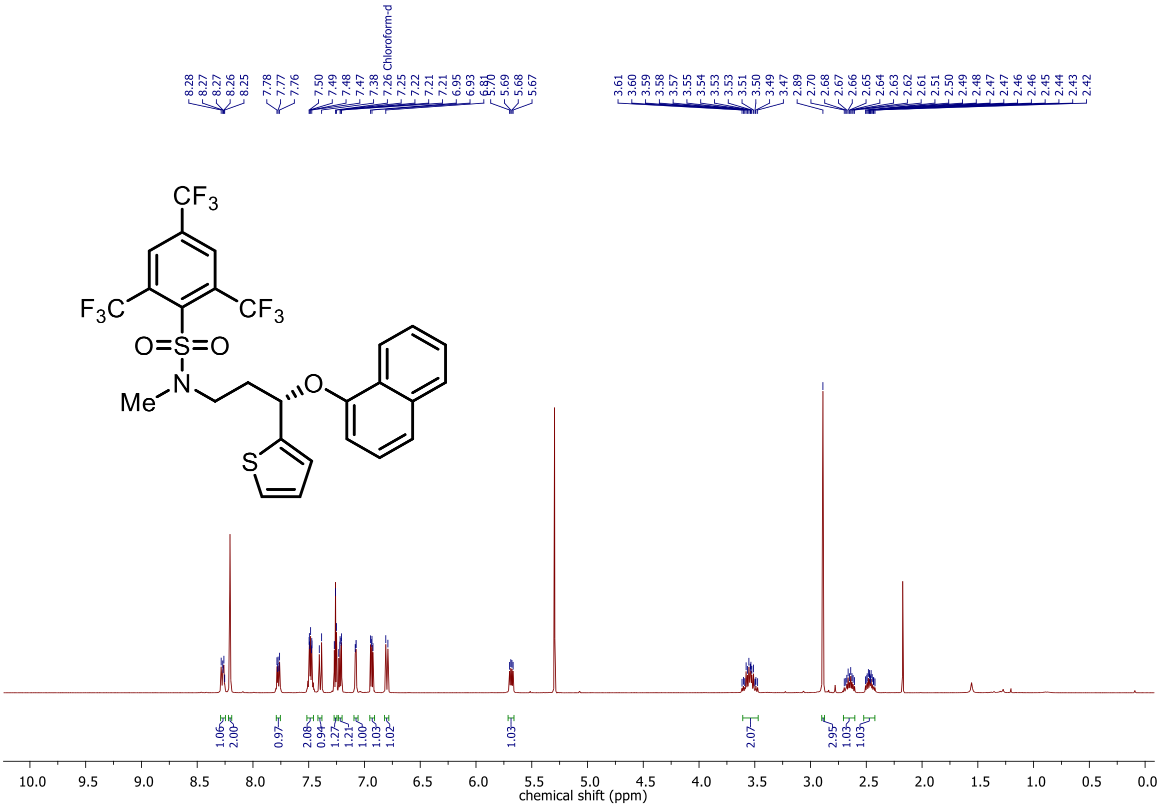

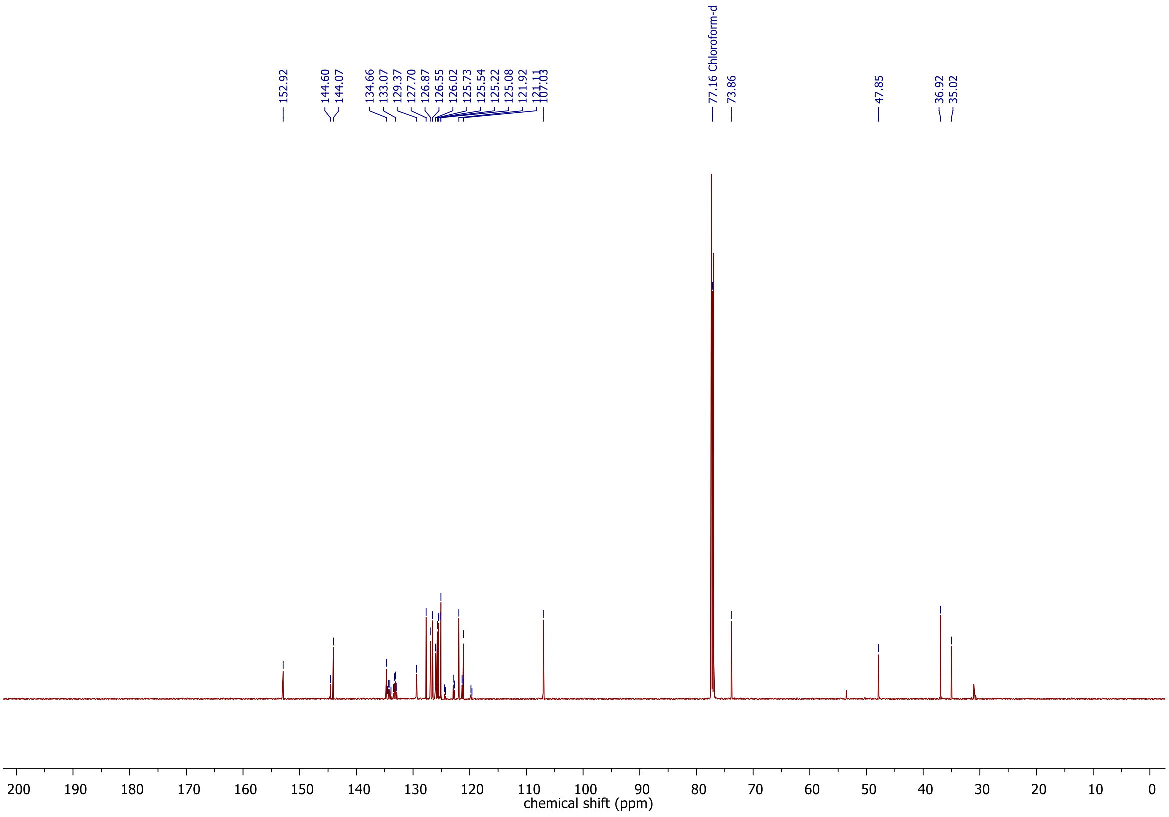

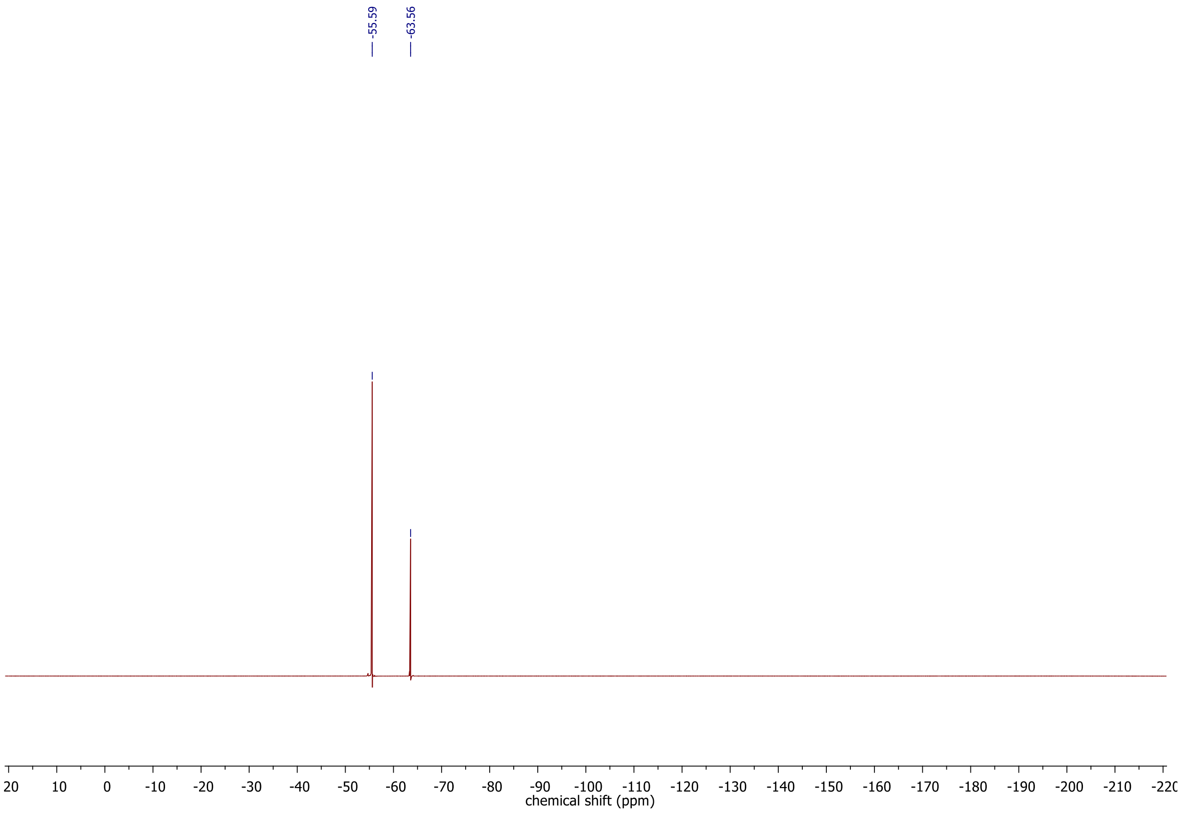

-55.59
-63.56
20 10 0 -10 -20 -30 -40 -50 -60 -70 -80 -90 -100 -110 -120 -130 -140 -150 -160 -170 -180 -190 -200 -210 -220
chemical shift (ppm)

***tert*-Butyl ((2,4,6-(tristrifluoromethyl)phenyl)sulfonyl]-*L*-prolinate (2ah)**

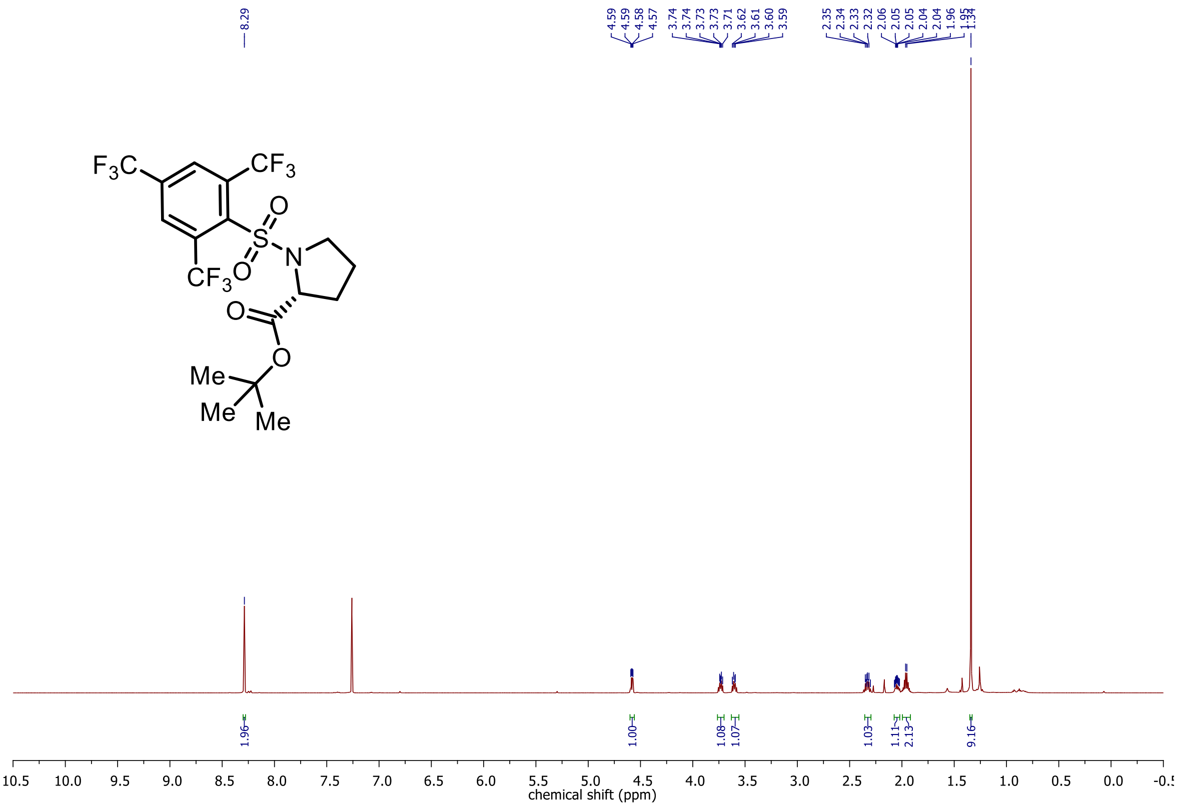

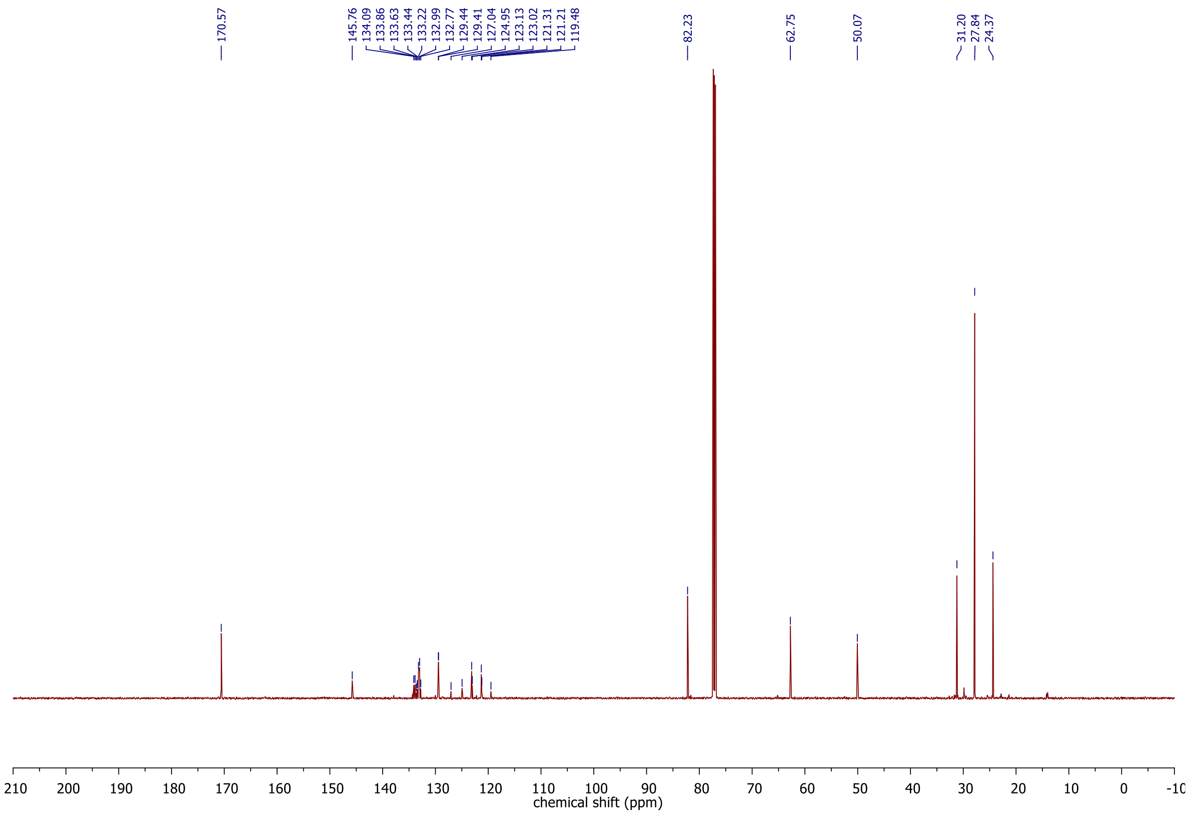

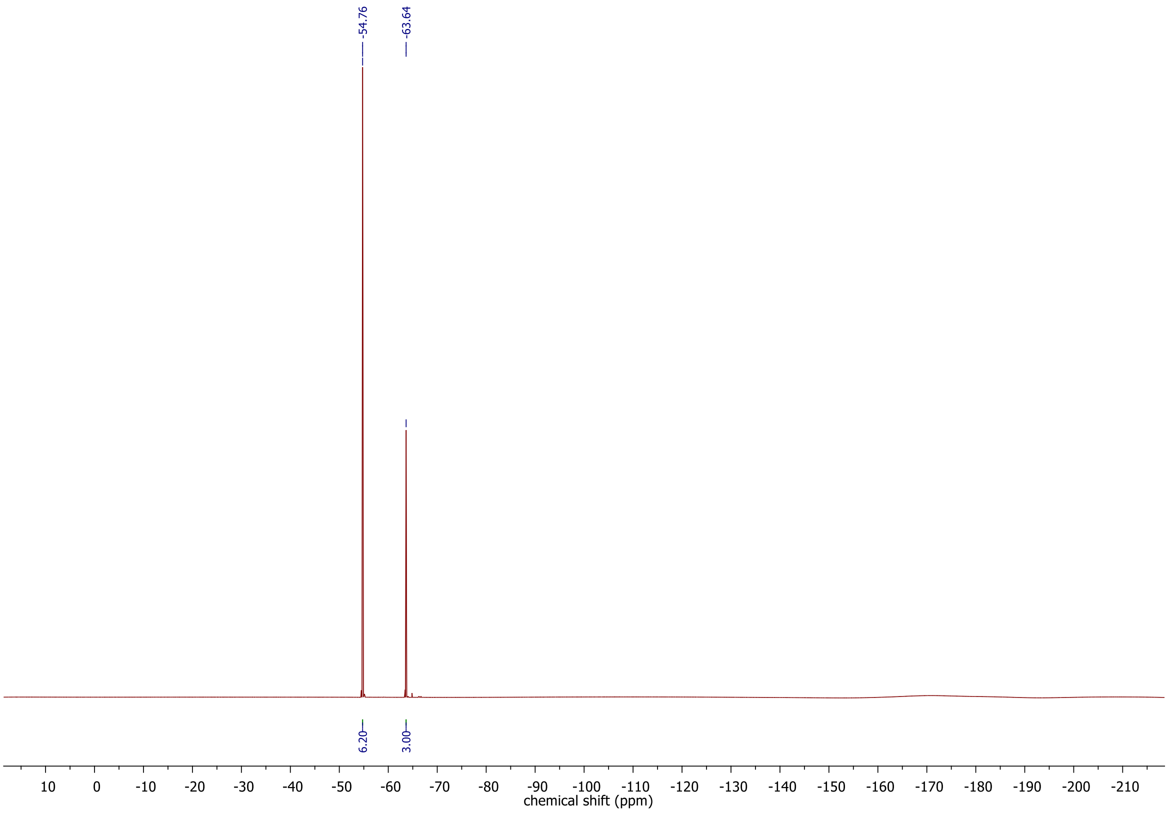
-54.76
-63.64
6.20
3.00
10
0
-10
-20
-30
-40
-50
-60
-70
-80
-90
-100
-110
-120
-130
-140
-150
-160
-170
-180
-190
-200
-210
chemical shift (ppm)

8-((2,4,6-Tris(trifluoromethyl)phenyl)sulfonyl)-8-azabicyclo[3.2.1]octan-3-one (2ai)

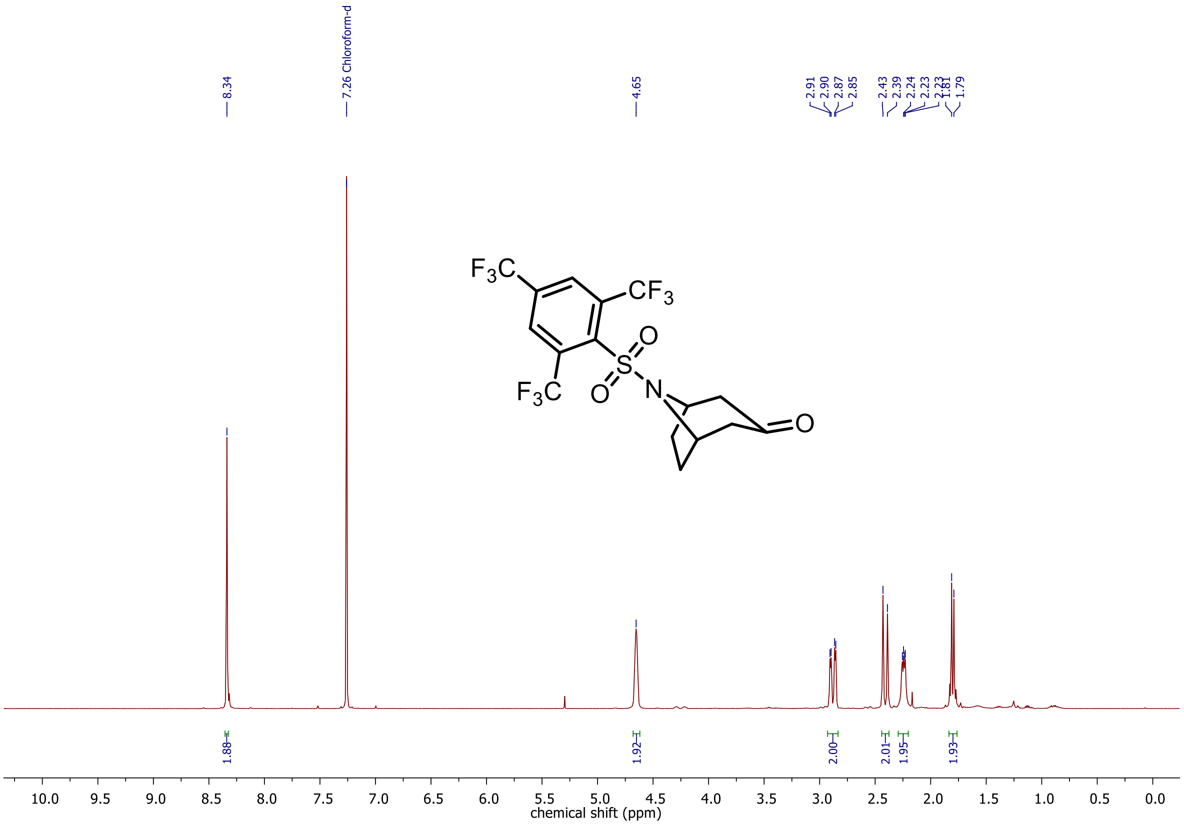

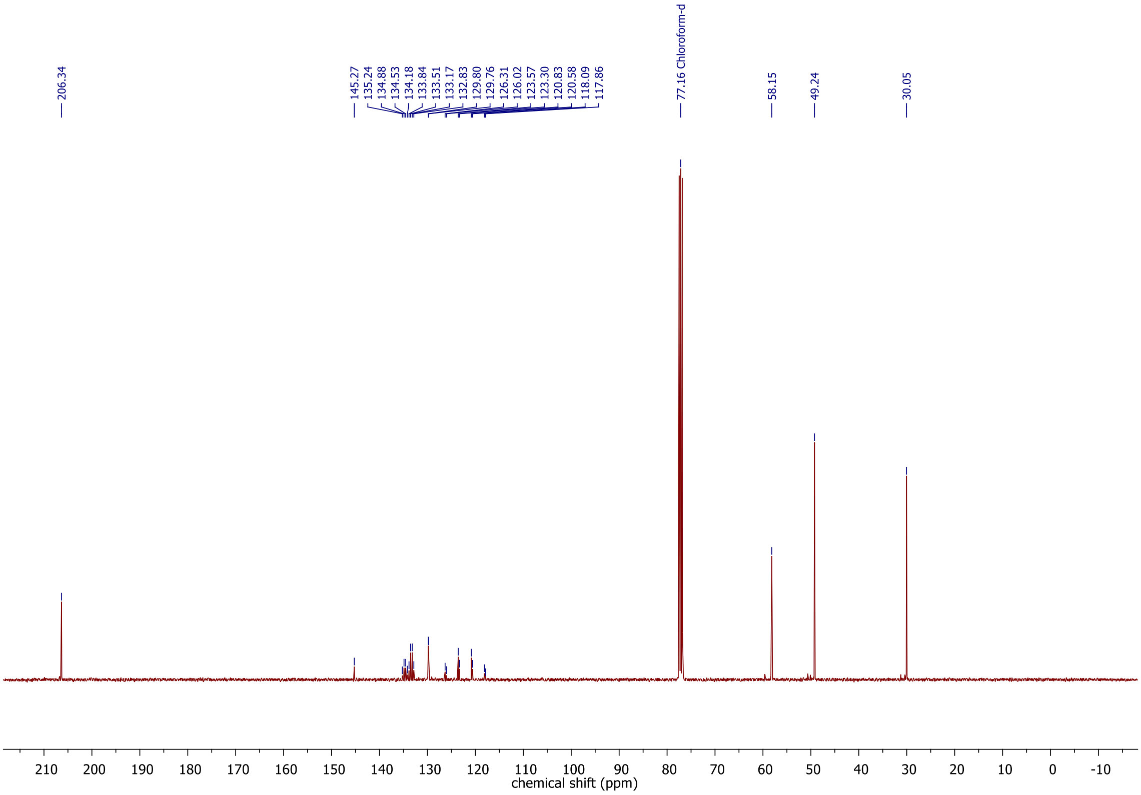

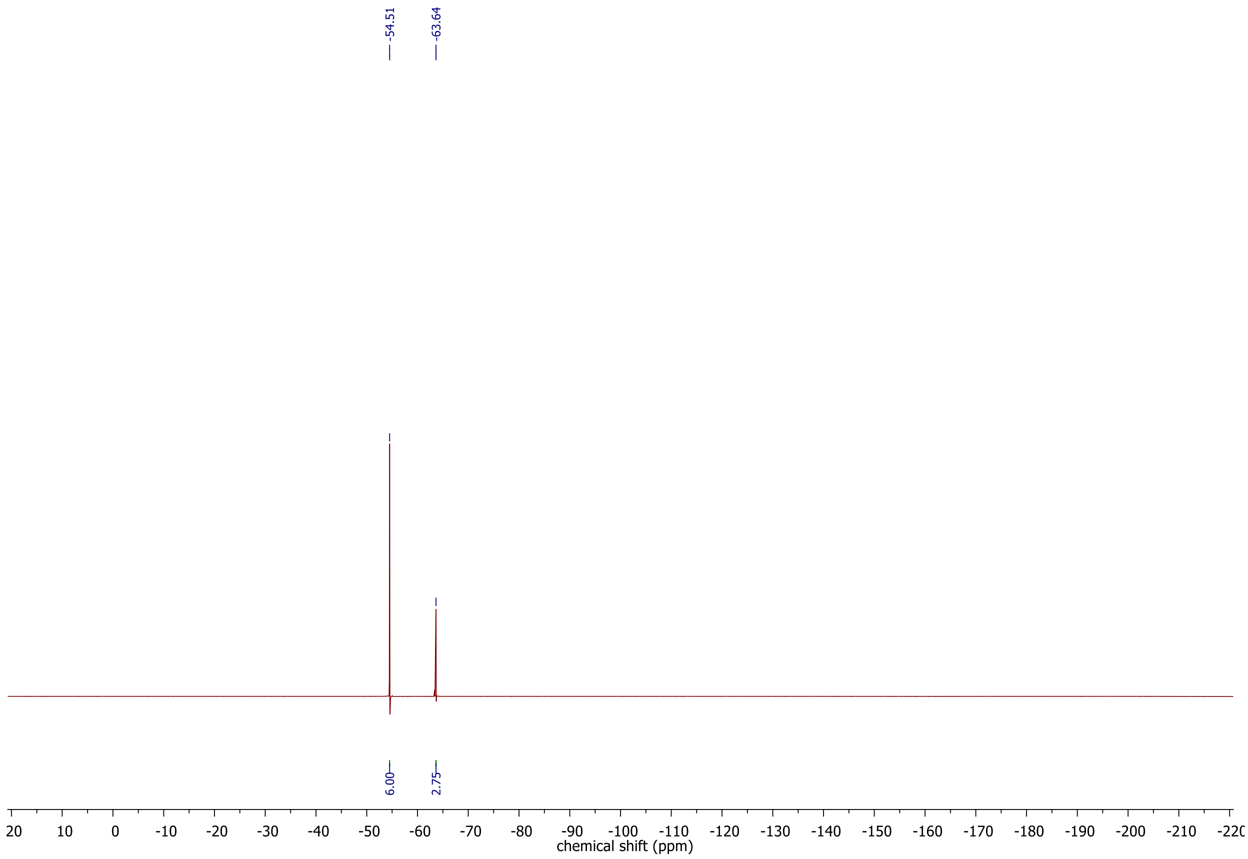

-54.51
-63.64
6.00
2.75
20 10 0 -10 -20 -30 -40 -50 -60 -70 -80 -90 -100 -110 -120 -130 -140 -150 -160 -170 -180 -190 -200 -210 -220
chemical shift (ppm)

2-Chloro-11-(4-((2,4,6-tris(trifluoromethyl)phenyl)sulfonyl)piperazin-1-yl)dibenzo[b,f][1,4]oxazepane (2aj)

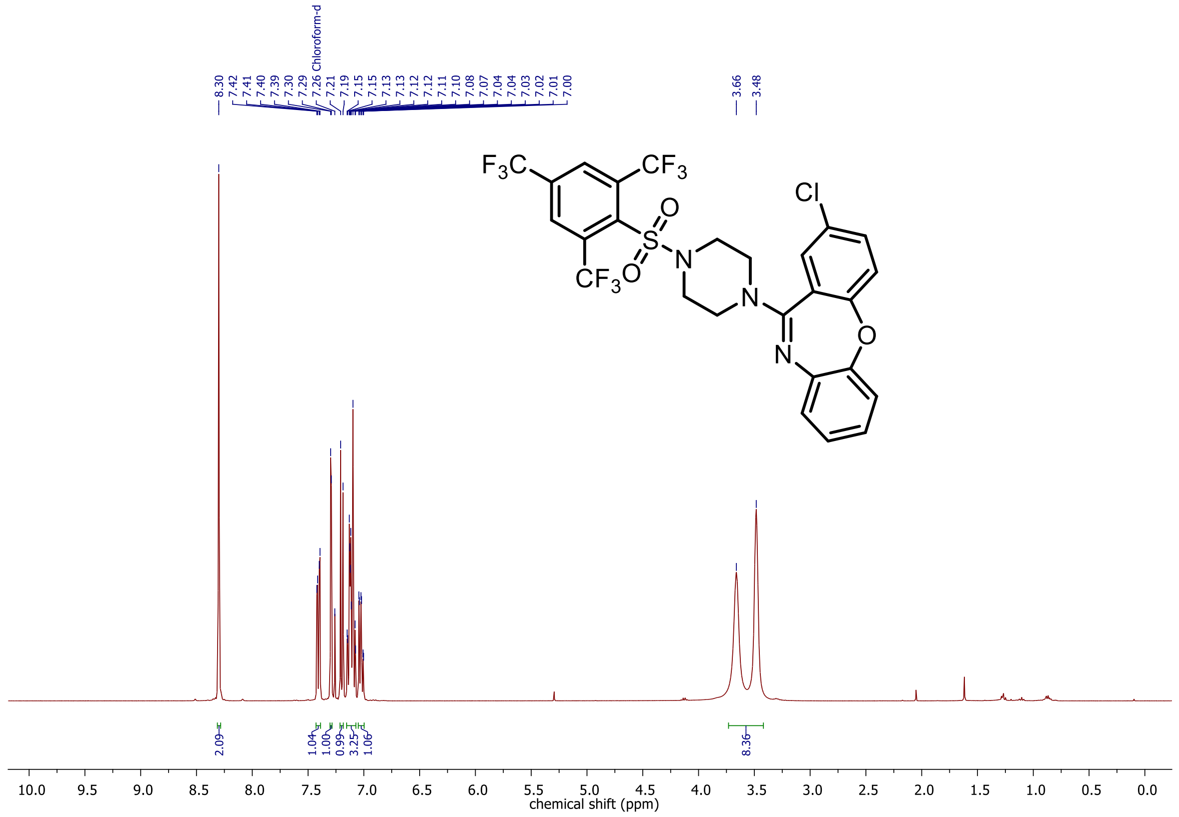

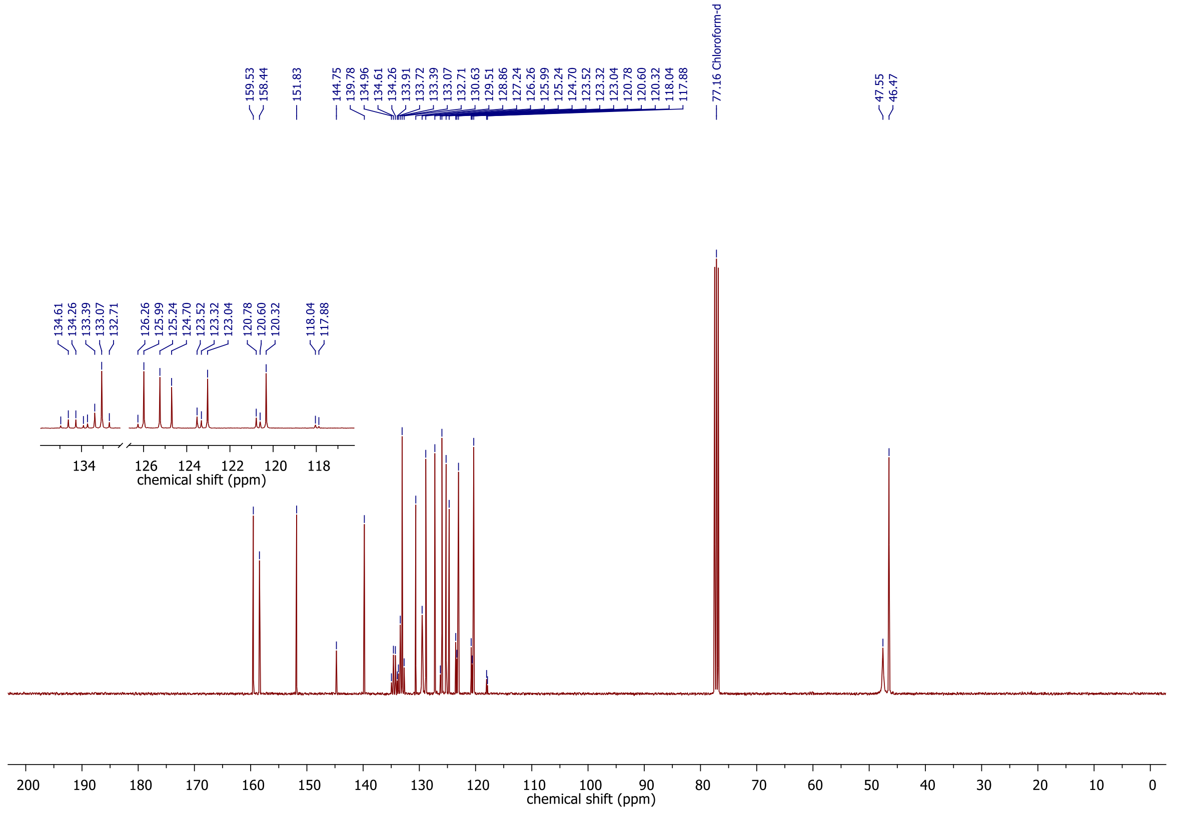

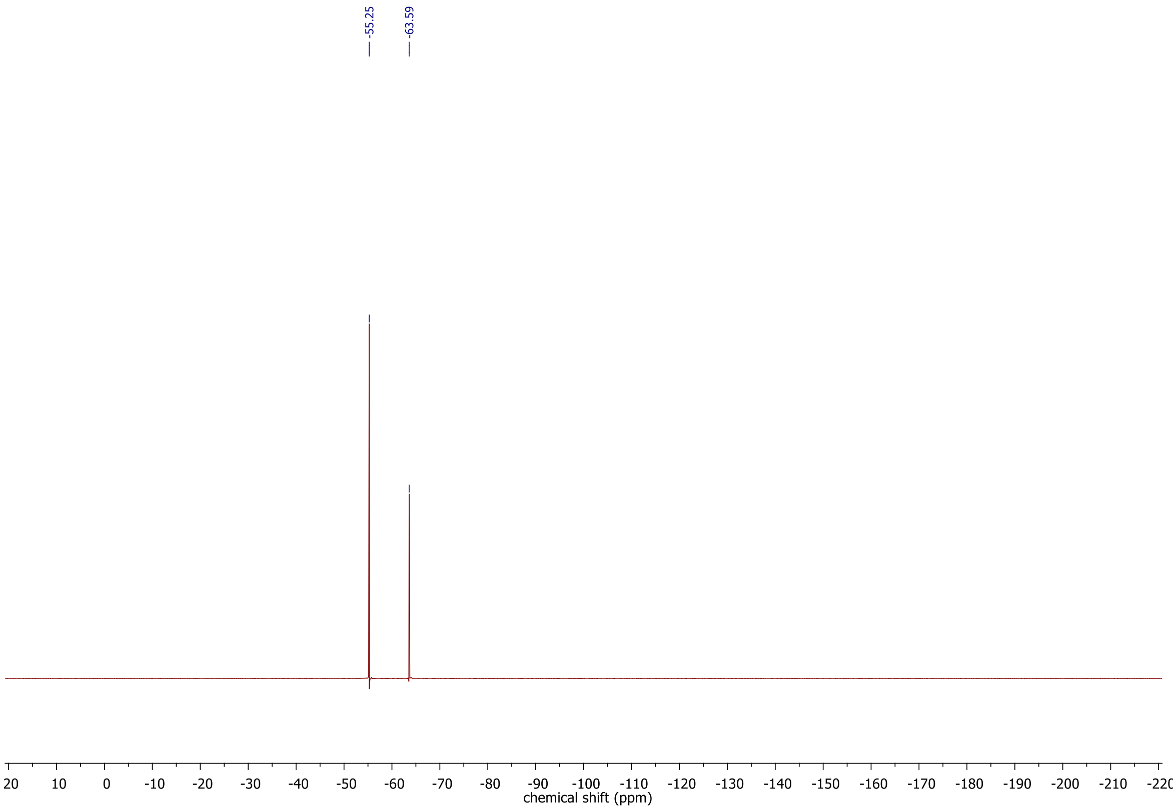
-55.25
-63.59
20 10 0 -10 -20 -30 -40 -50 -60 -70 -80 -90 -100 -110 -120 -130 -140 -150 -160 -170 -180 -190 -200 -210 -220
chemical shift (ppm)

(3*S*,4*R*)-3-((Benzo[d][1,3]dioxol-5-yloxy)methyl)-4-(4-fluorophenyl)-1-((2,4,6-tris(trifluoromethyl)phenyl)sulfonyl)piperidine (2ak)

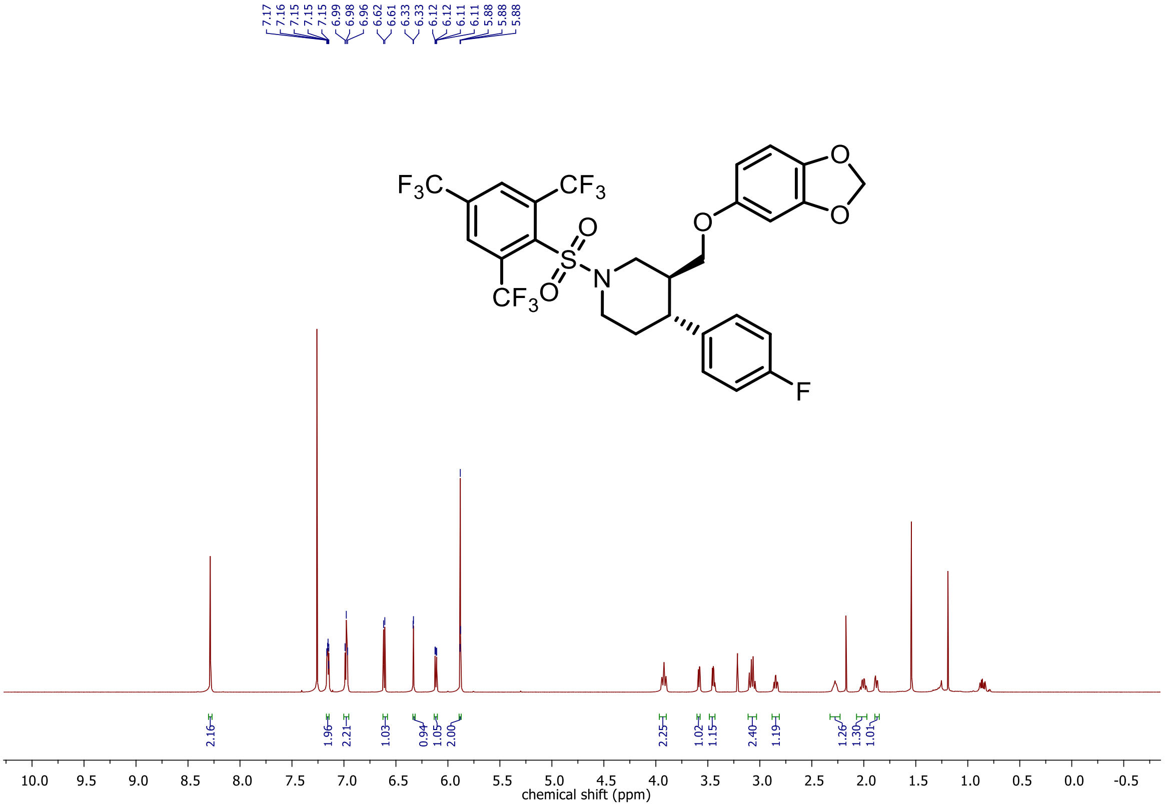

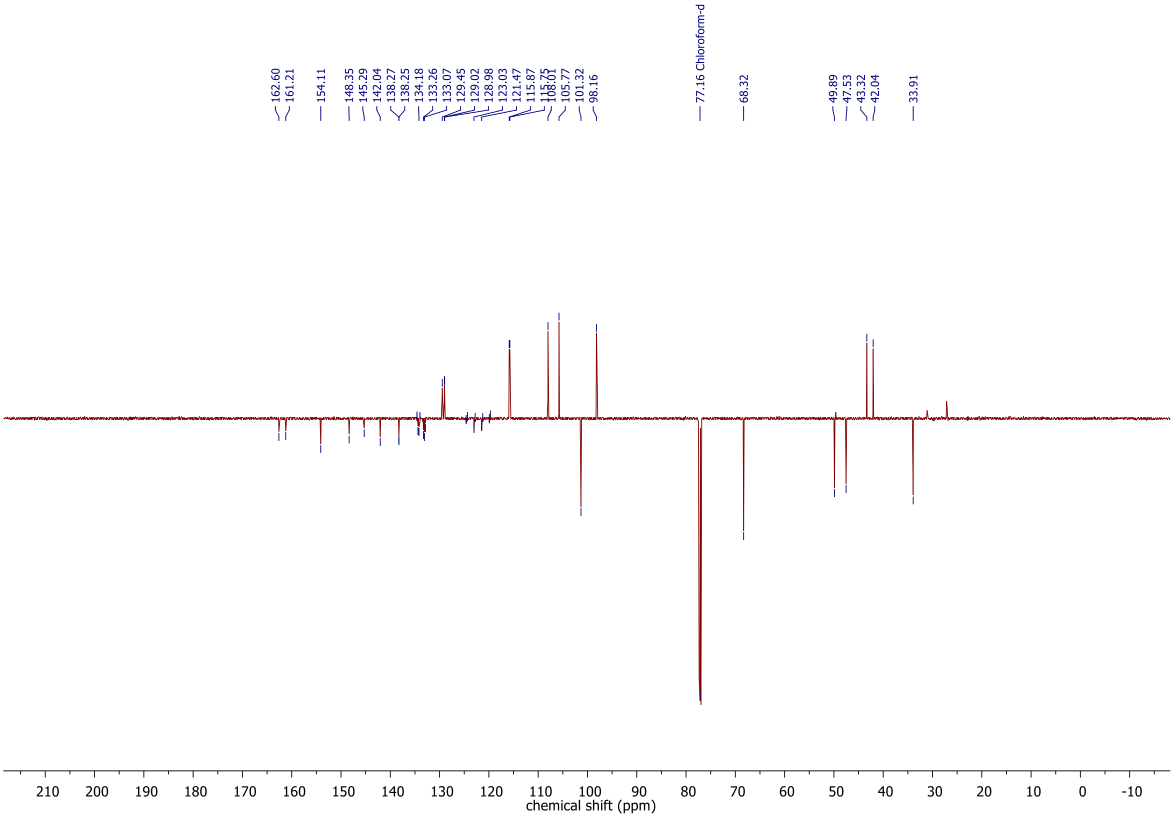

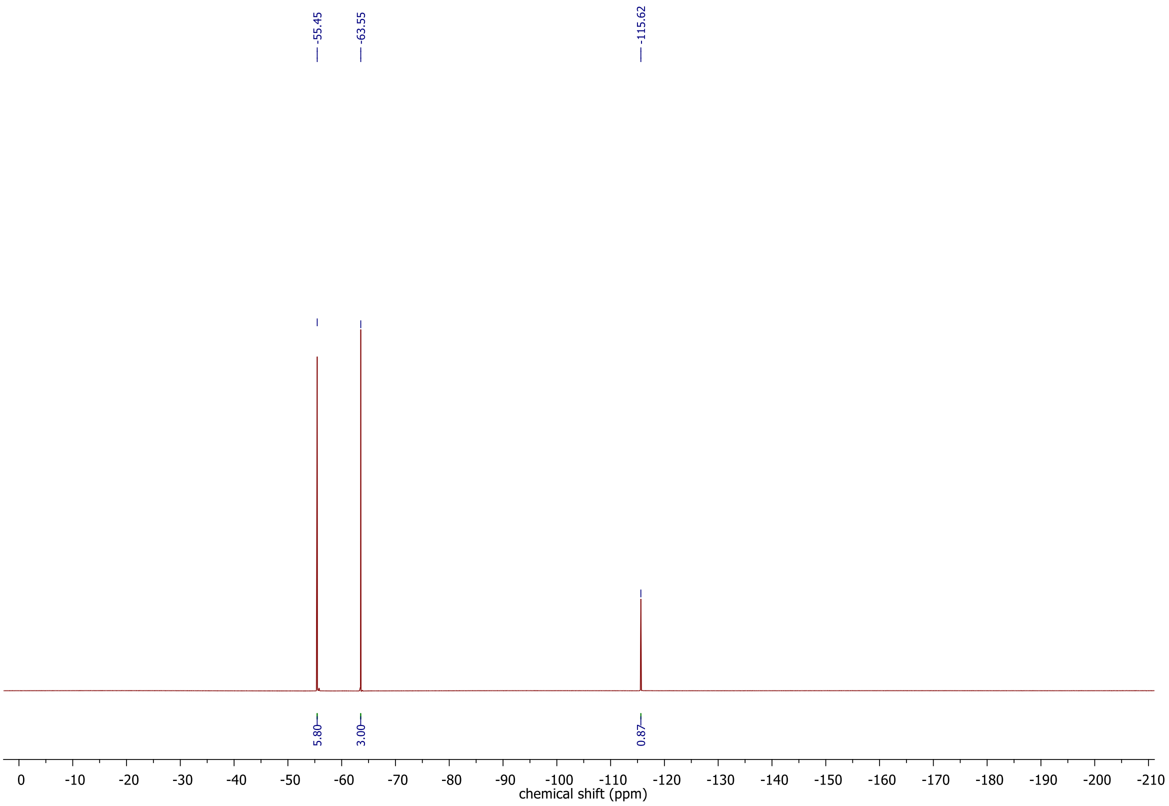

-55.45
-63.55
-115.62
5.80
3.00
0.87
0 -10 -20 -30 -40 -50 -60 -70 -80 -90 -100 -110 -120 -130 -140 -150 -160 -170 -180 -190 -200 -210
chemical shift (ppm)

8-Chloro-11-(1-((2,4,6-tris(trifluoromethyl)phenyl)sulfonyl)piperidin-4-ylidene)-6,11-dihydro-5H-benzo[5,6]cyclohepta[1,2-b]pyridine (2al)

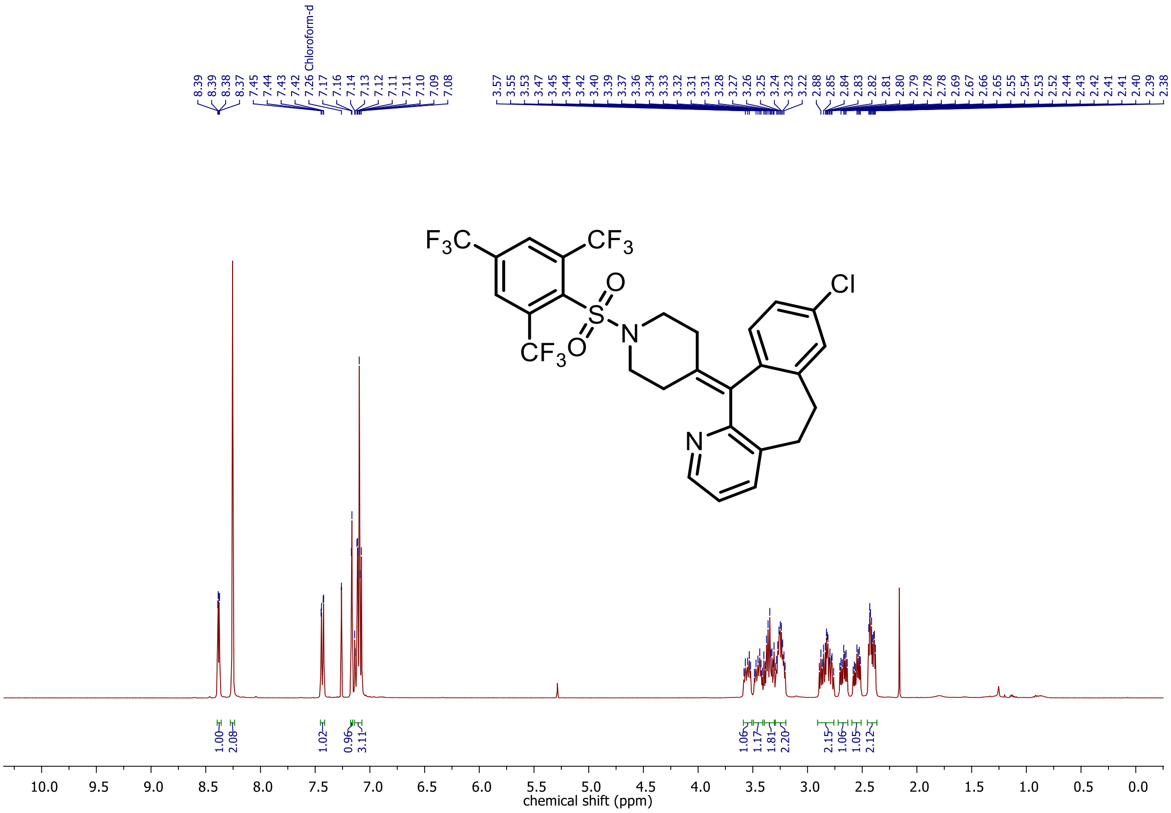

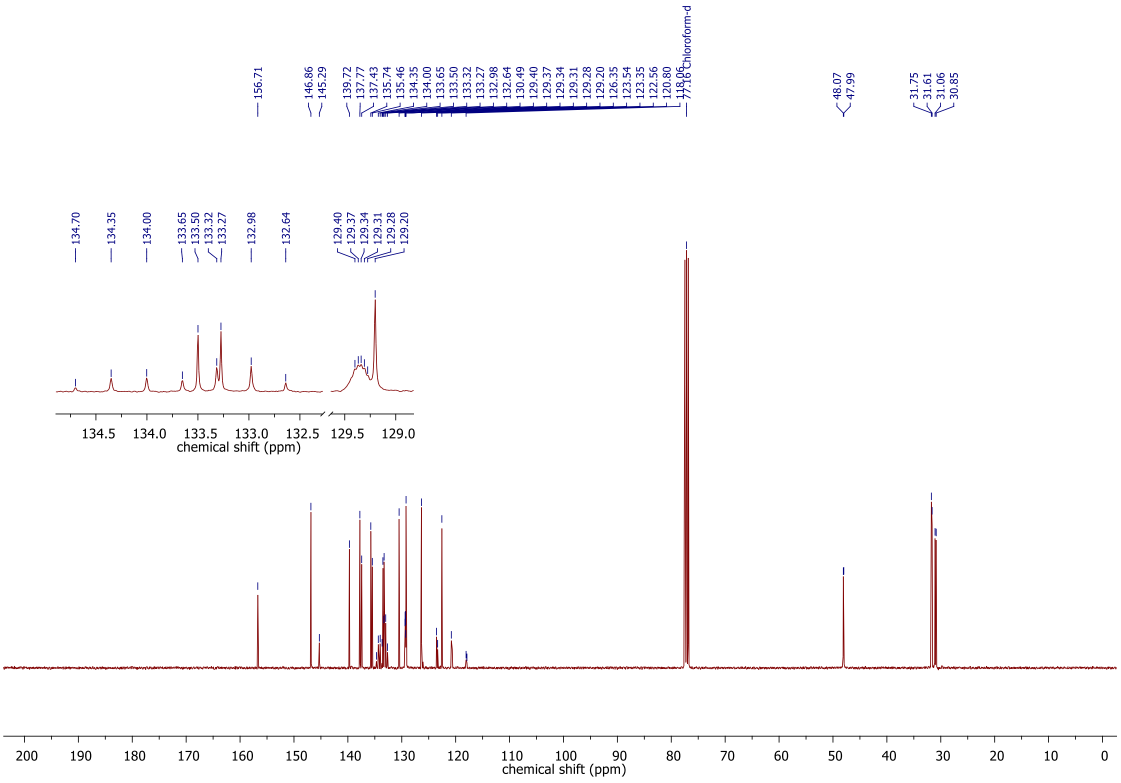

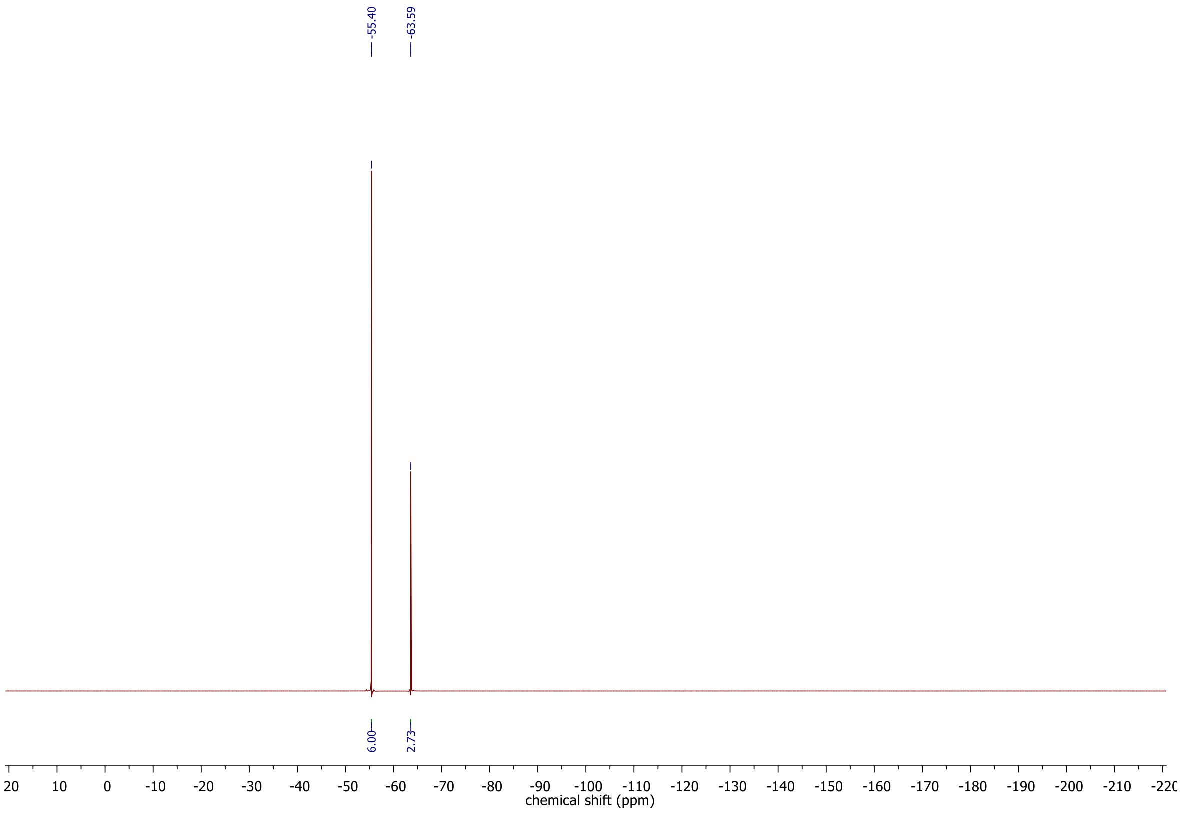
-55.40
-63.59
6.00
2.73
20 10 0 -10 -20 -30 -40 -50 -60 -70 -80 -90 -100 -110 -120 -130 -140 -150 -160 -170 -180 -190 -200 -210 -220
chemical shift (ppm)

Mentylamine (1r)

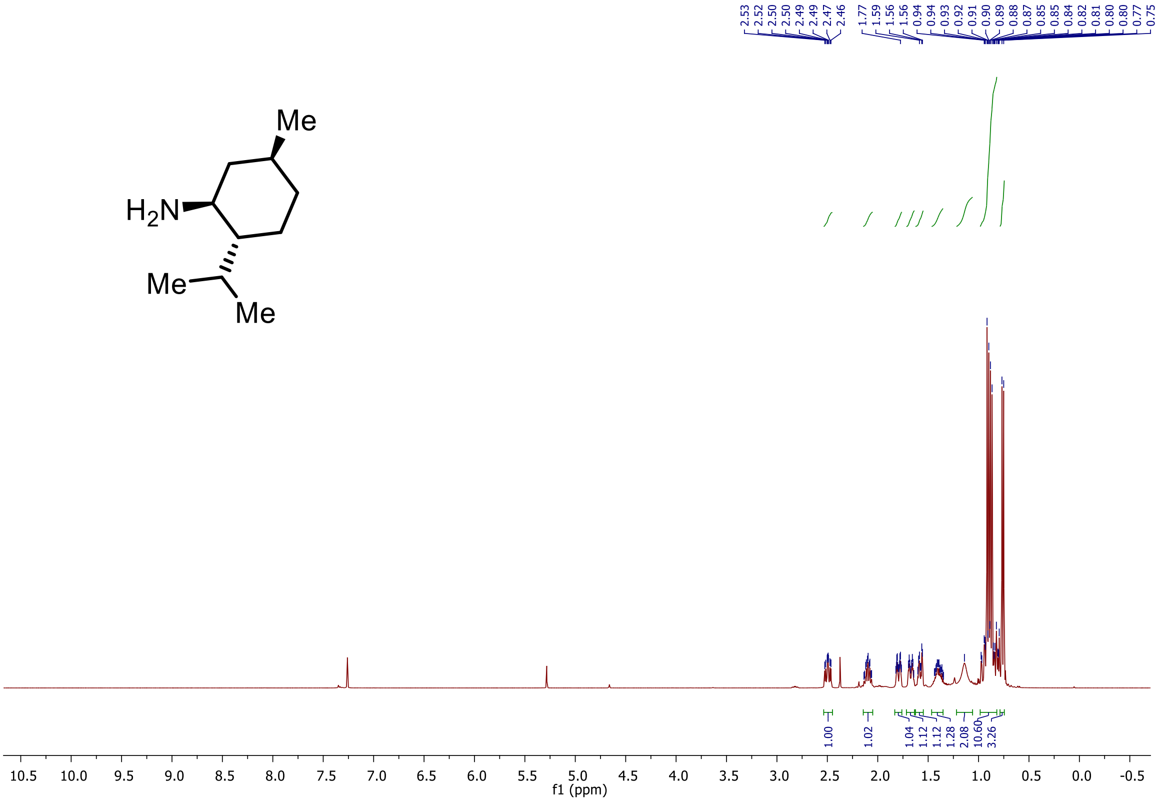

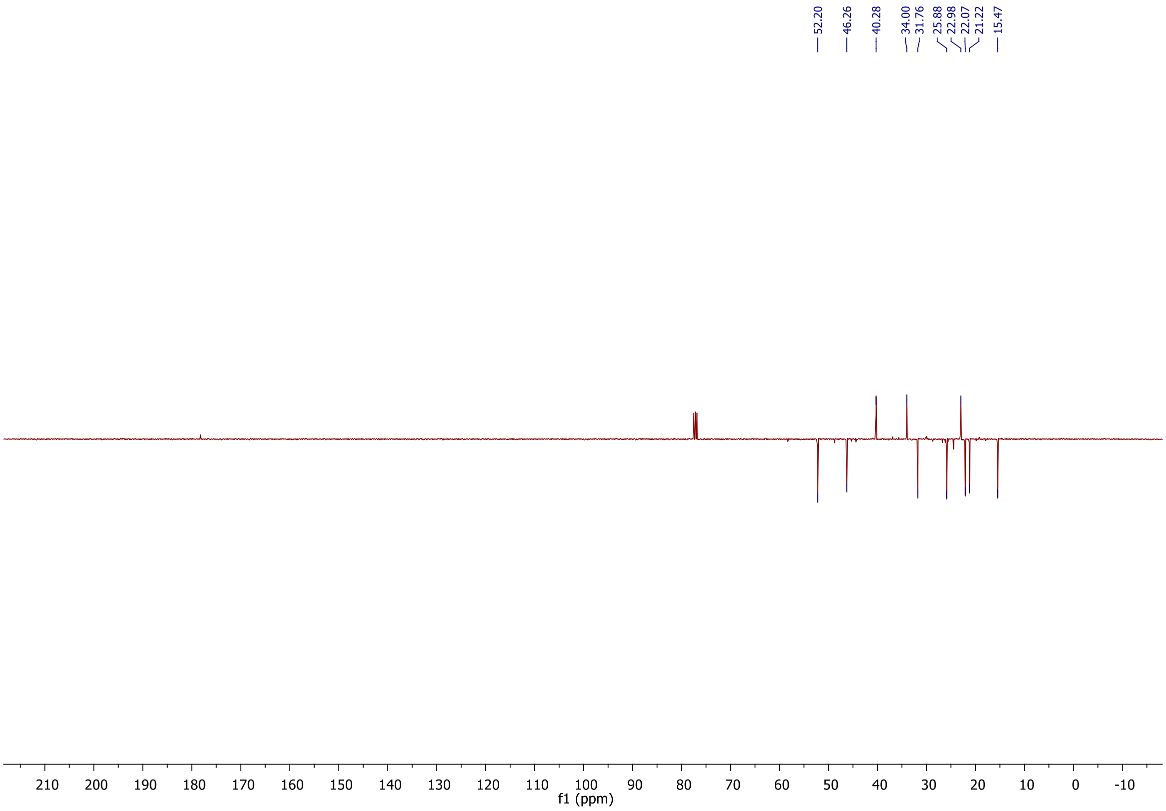

Paroxetine (1ak)

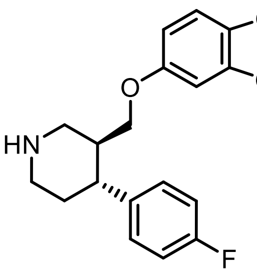

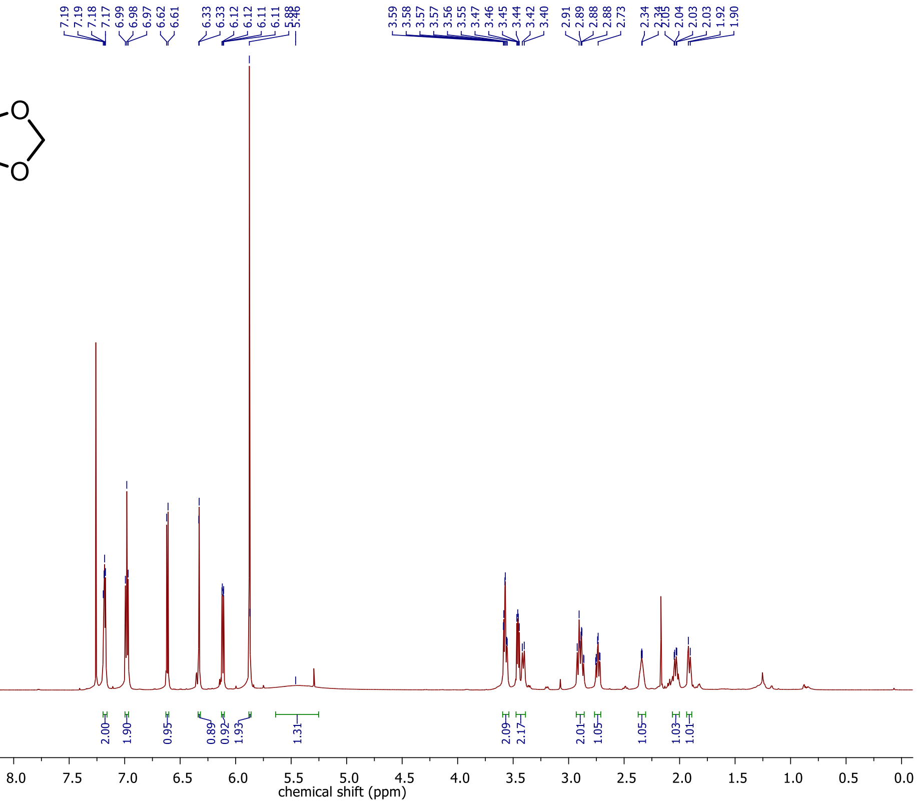

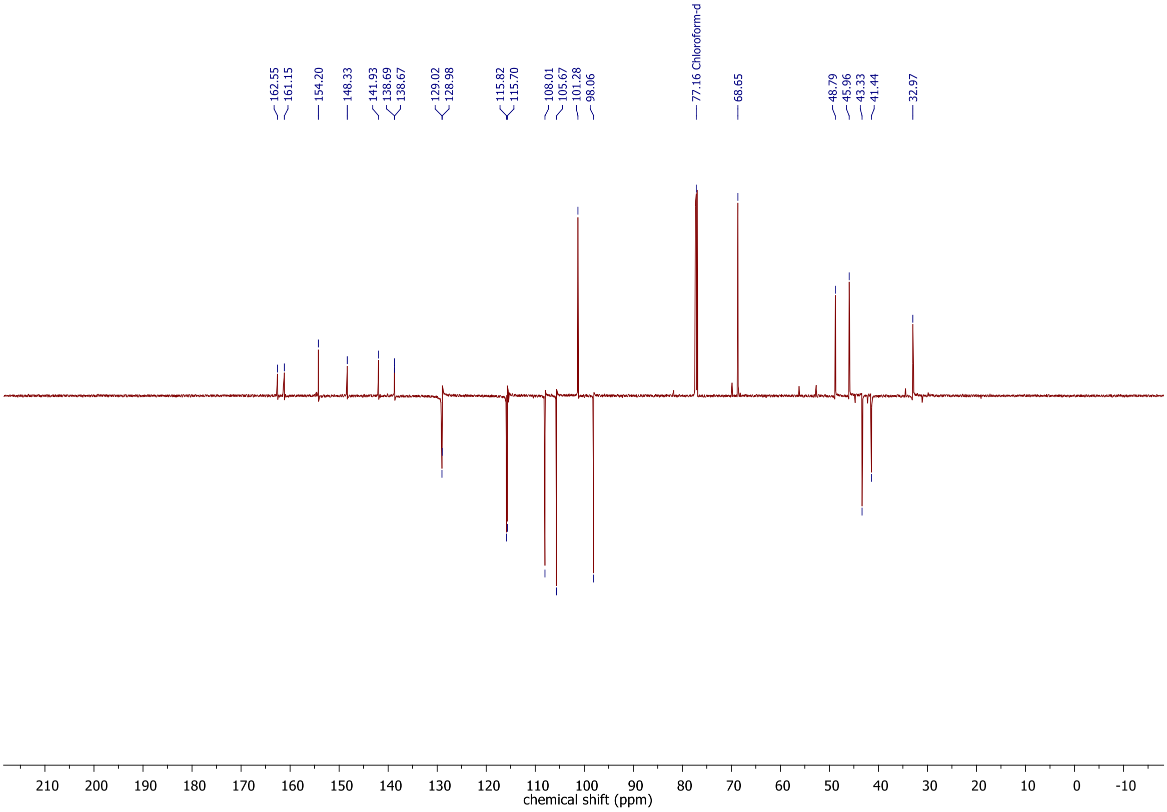

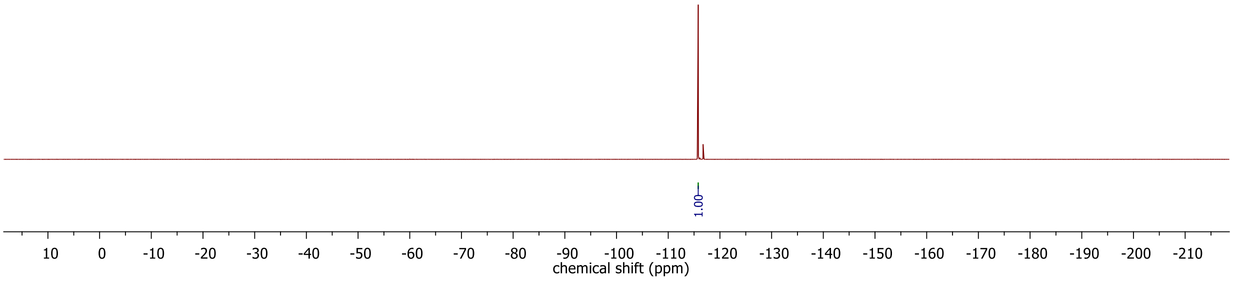
1.00
10
0
-10
-20
-30
-40
-50
-60
-70
-80
-90
-100
-110
-120
-130
-140
-150
-160
-170
-180
-190
-200
-210
chemical shift (ppm)

Phenyl(2,4,6-tris(trifluoromethyl)phenyl)sulfane (3)

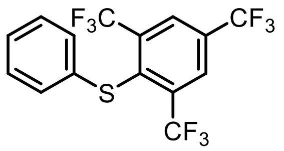

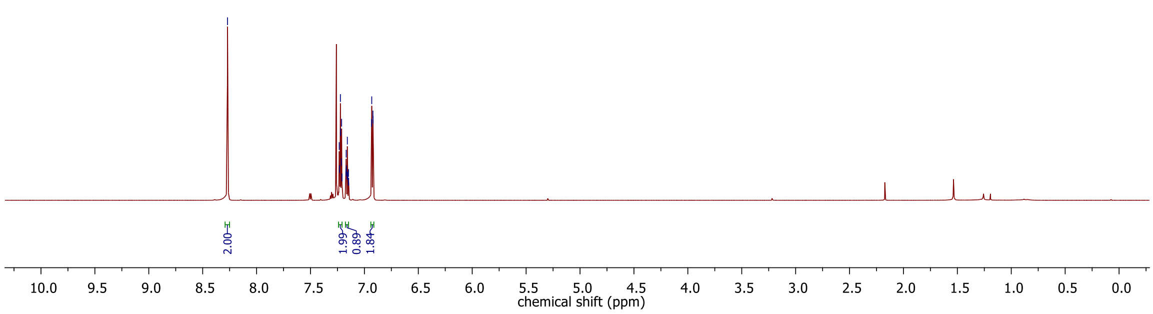

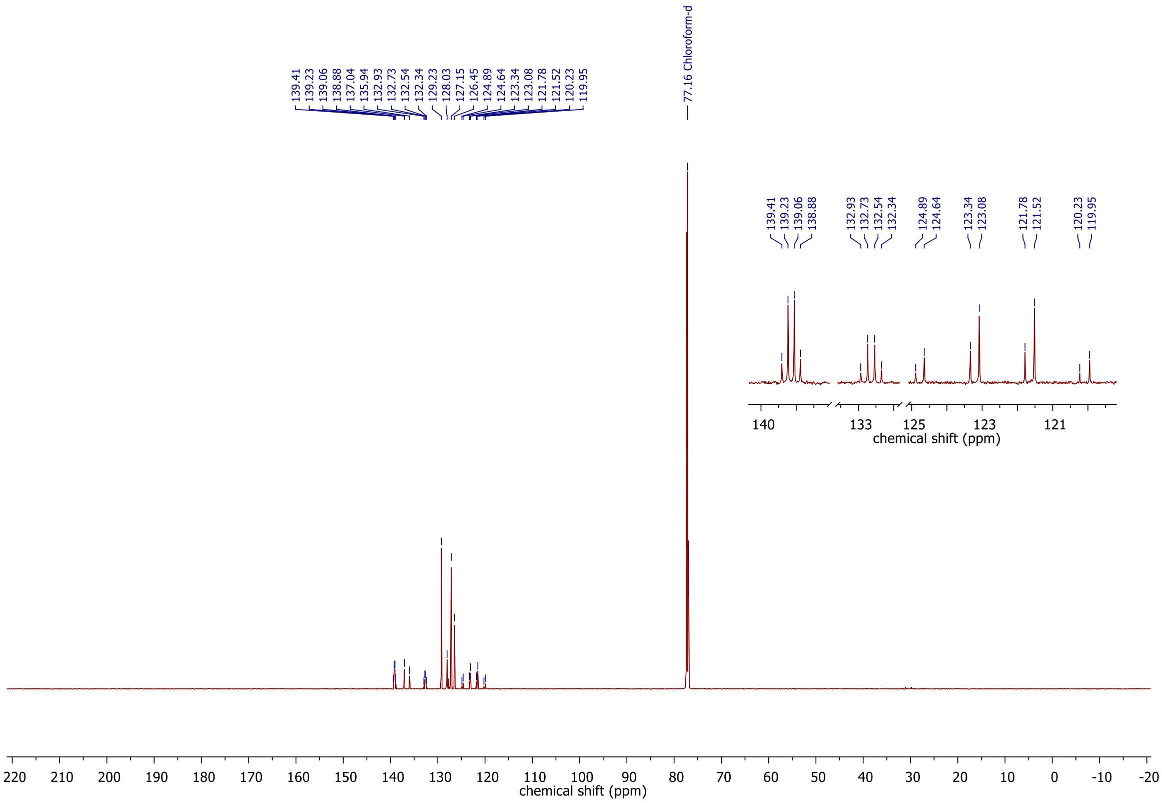

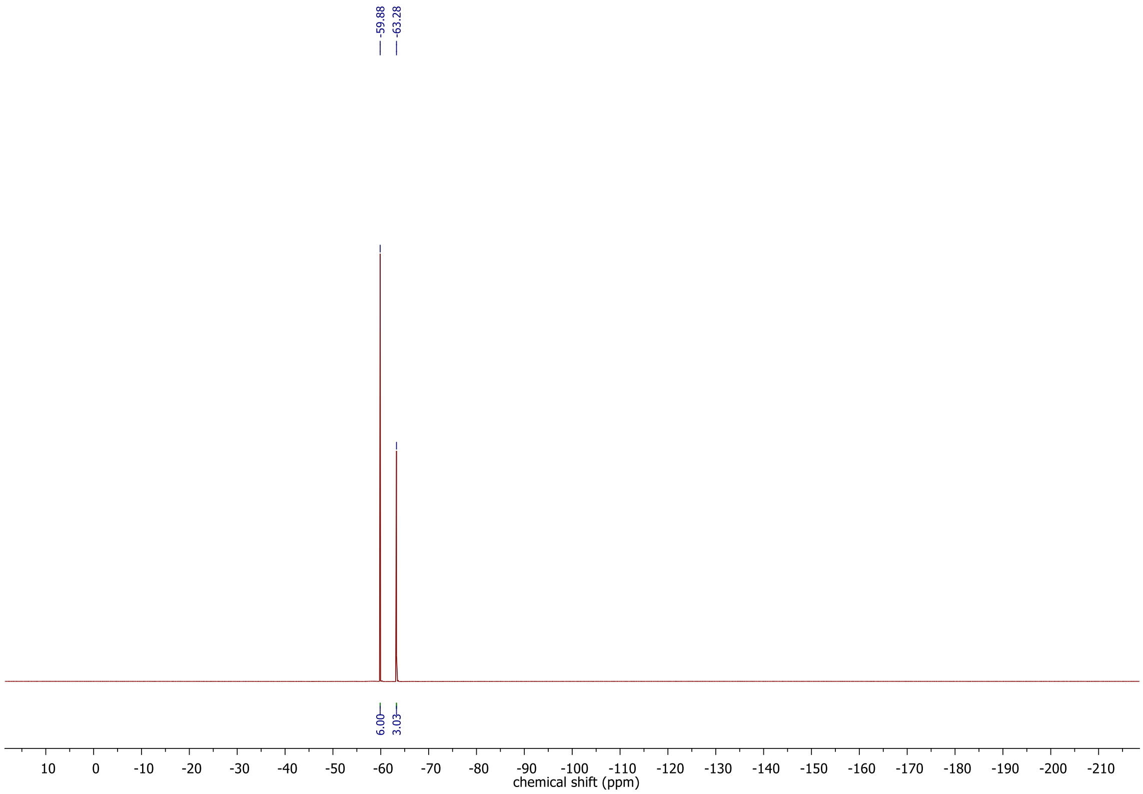
-59.88
-63.28
6.00
3.03
10 0 -10 -20 -30 -40 -50 -60 -70 -80 -90 -100 -110 -120 -130 -140 -150 -160 -170 -180 -190 -200 -210
chemical shift (ppm)

N-(1-oxo-4-phenyl-1-(pyrrolidin-1-yl)butan-2-yl)-2,4,6-tris(trifluoromethyl)benzenesulfonamide (8)

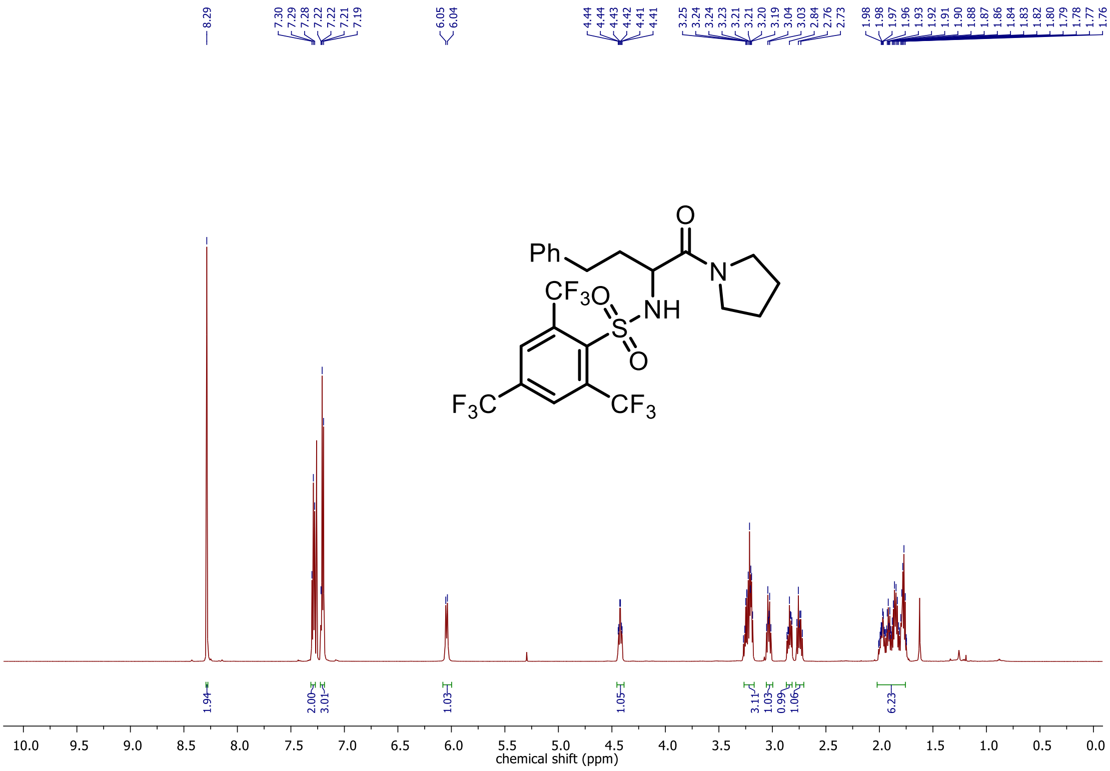

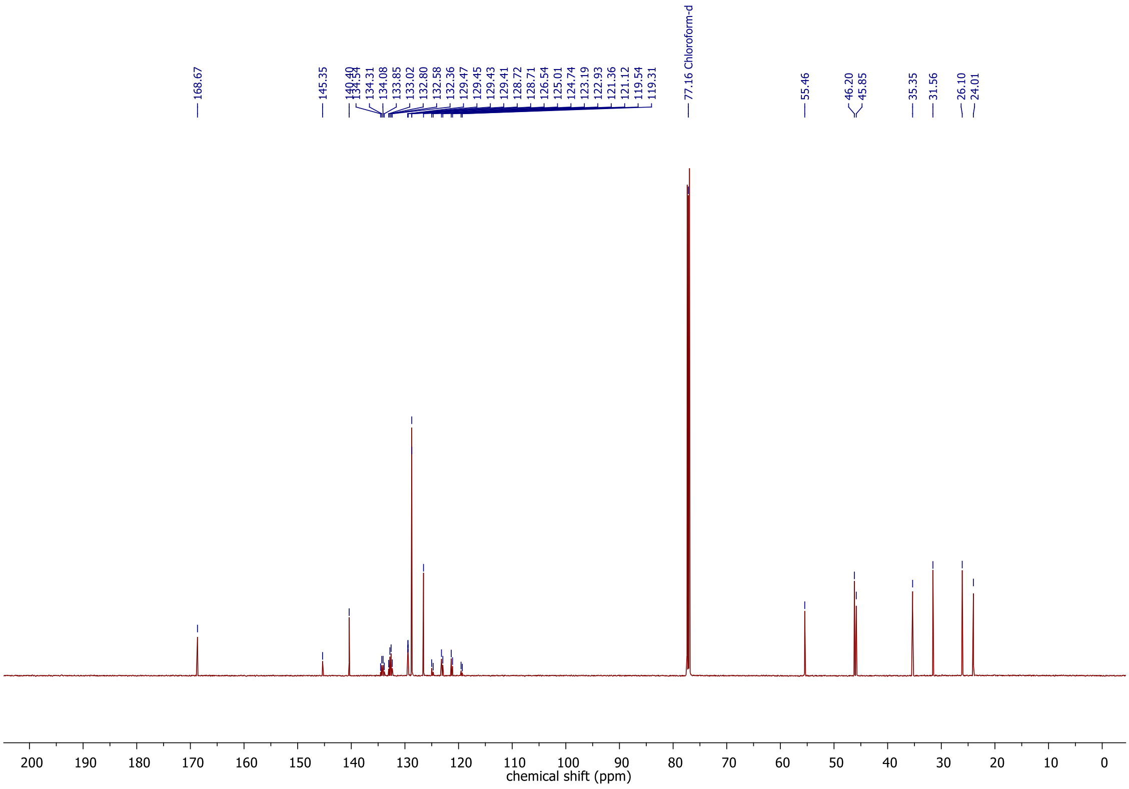

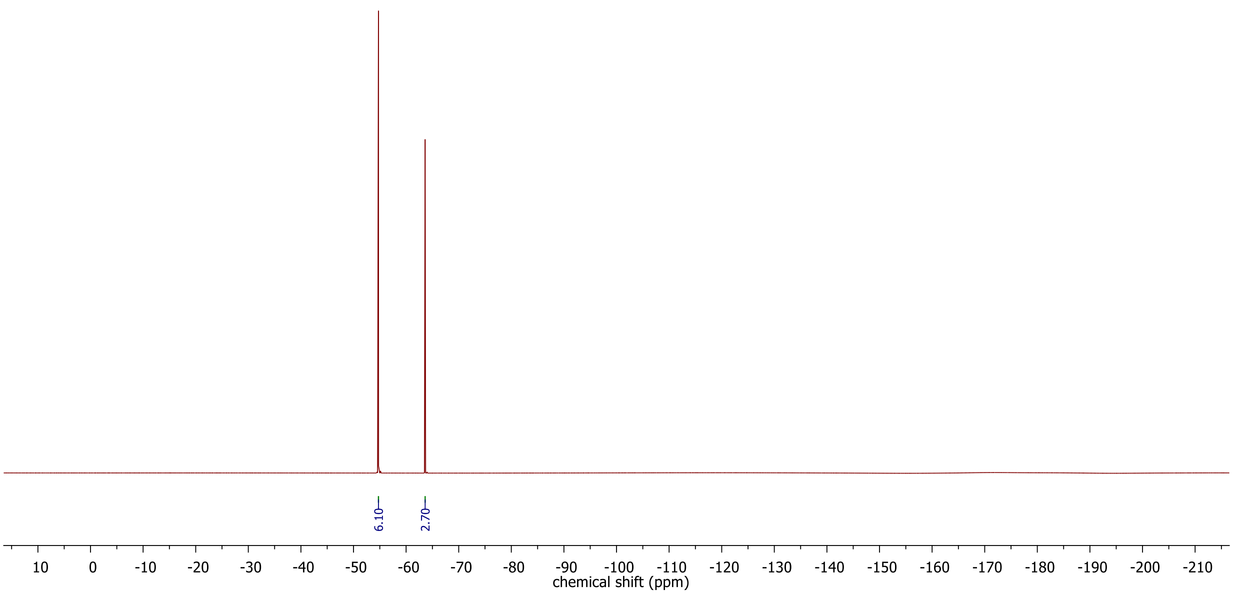
6.10
2.70
10 0 -10 -20 -30 -40 -50 -60 -70 -80 -90 -100 -110 -120 -130 -140 -150 -160 -170 -180 -190 -200 -210
chemical shift (ppm)

N-(3-Aminopropyl)-2,4,6-tris(trifluoromethyl)benzenesulfonamide (15)

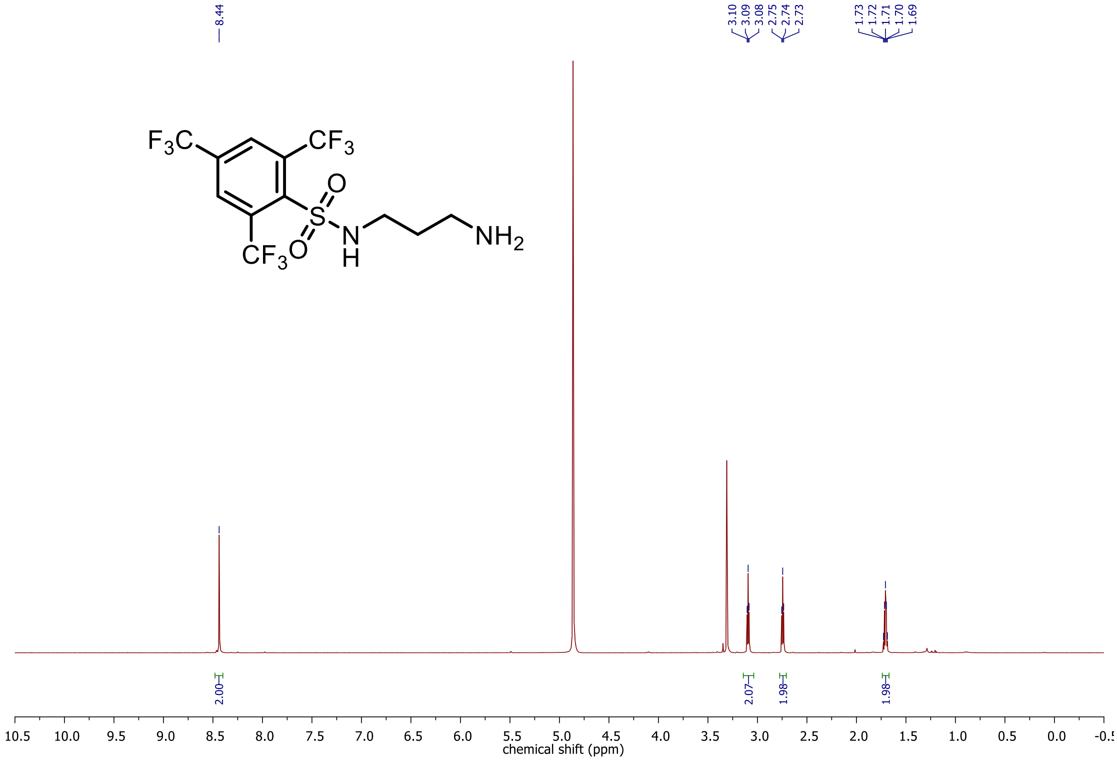

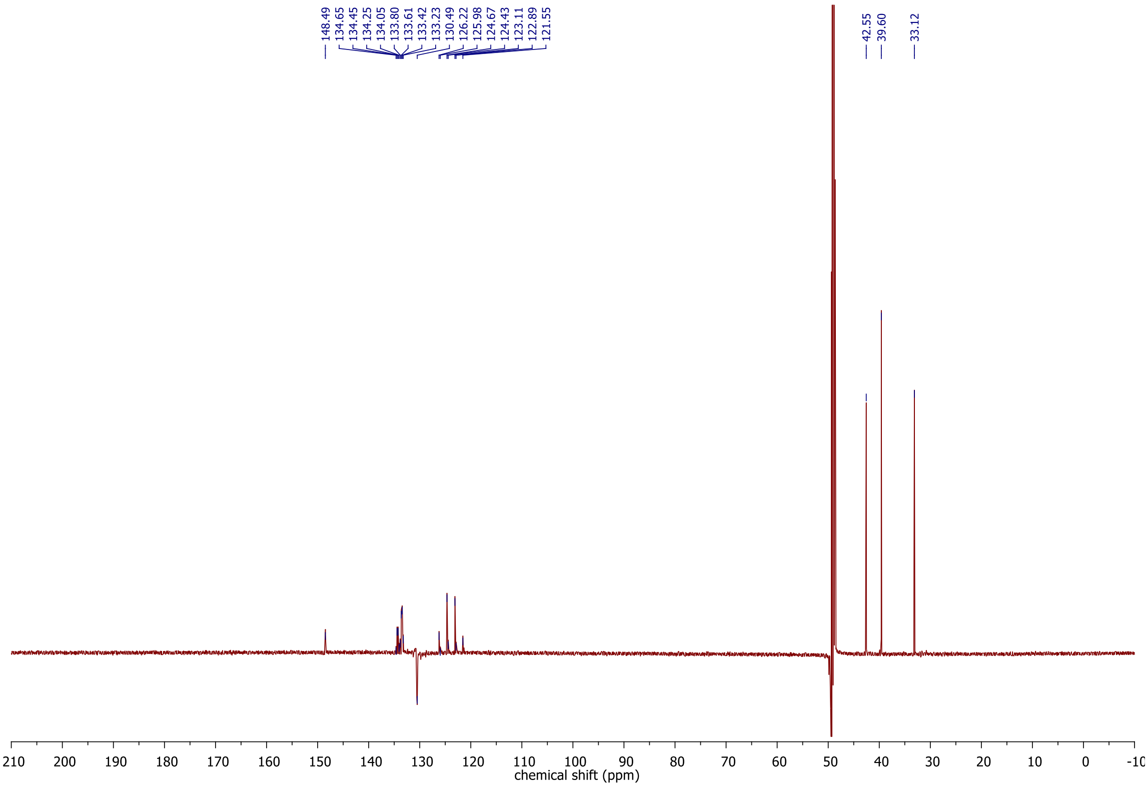

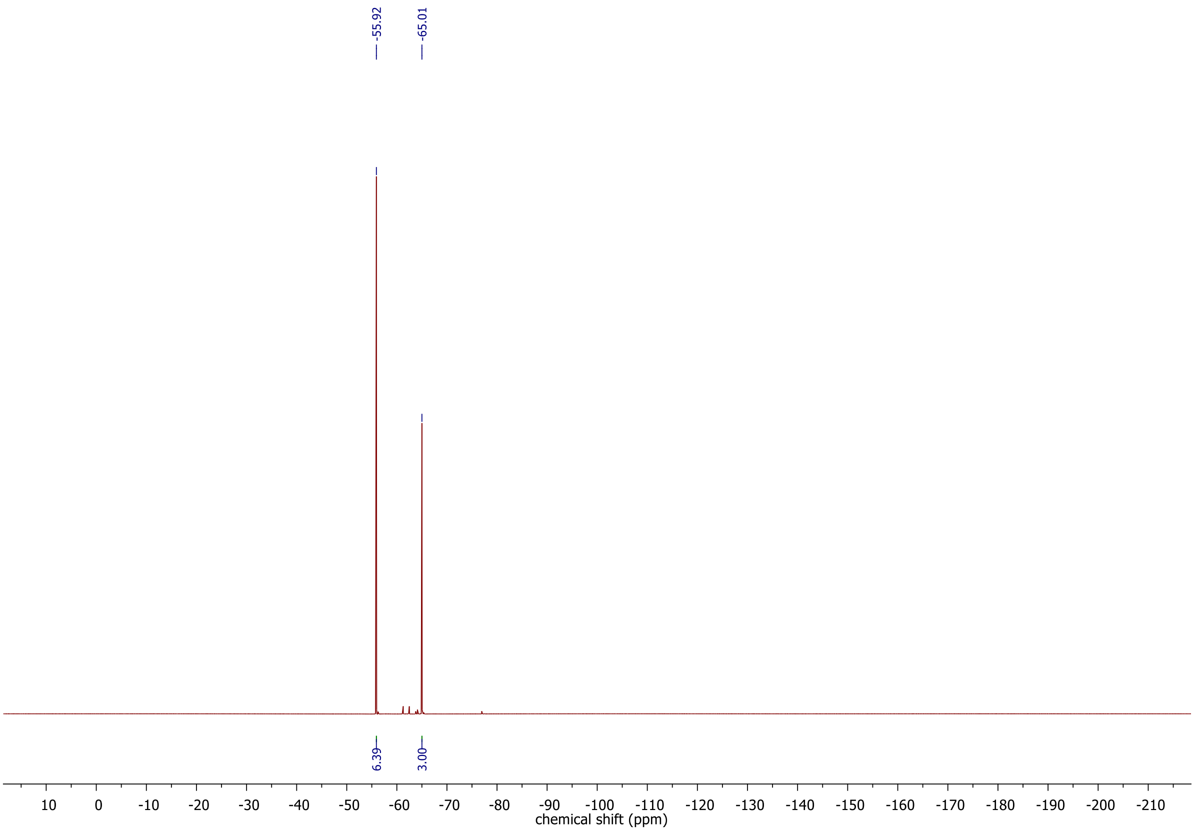

-55.92
-65.01
6.39
3.00
10
0
-10
-20
-30
-40
-50
-60
-70
-80
-90
-100
-110
-120
-130
-140
-150
-160
-170
-180
-190
-200
-210
chemical shift (ppm)

***N*-(3-((4-Nitrophenyl)sulfonamido)propyl)-2,4,6-tris(trifluoromethyl)benzenesulfonamide (12)**

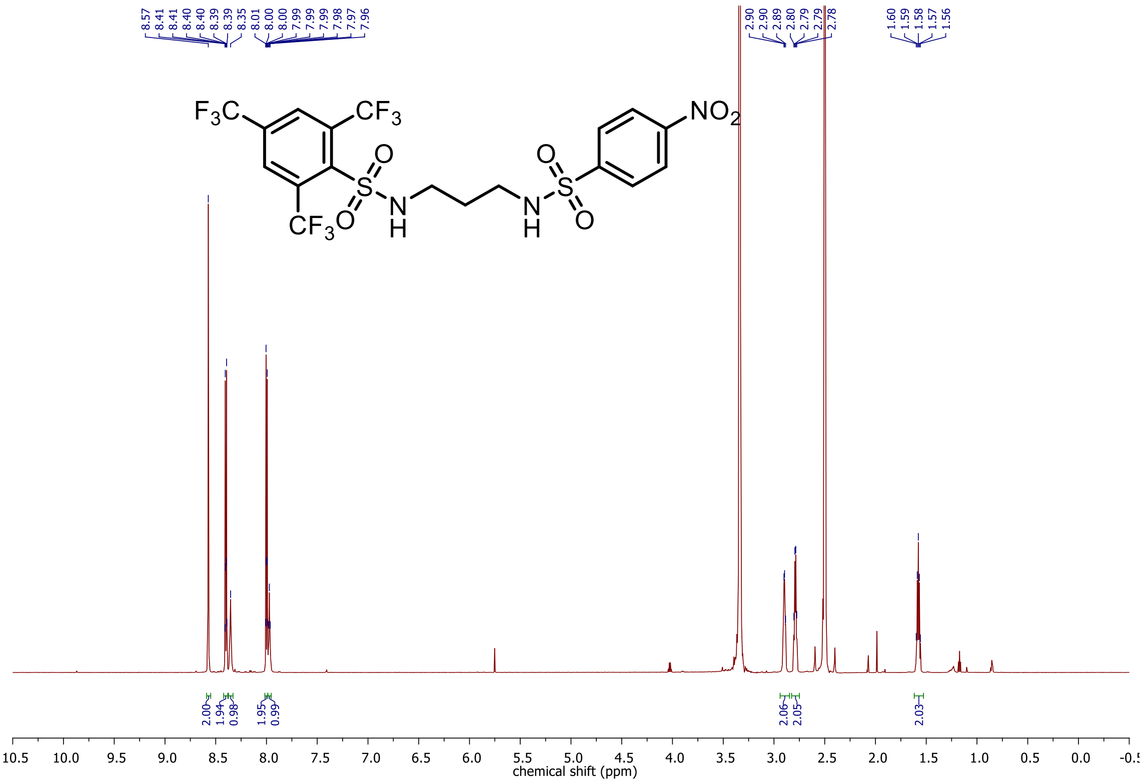

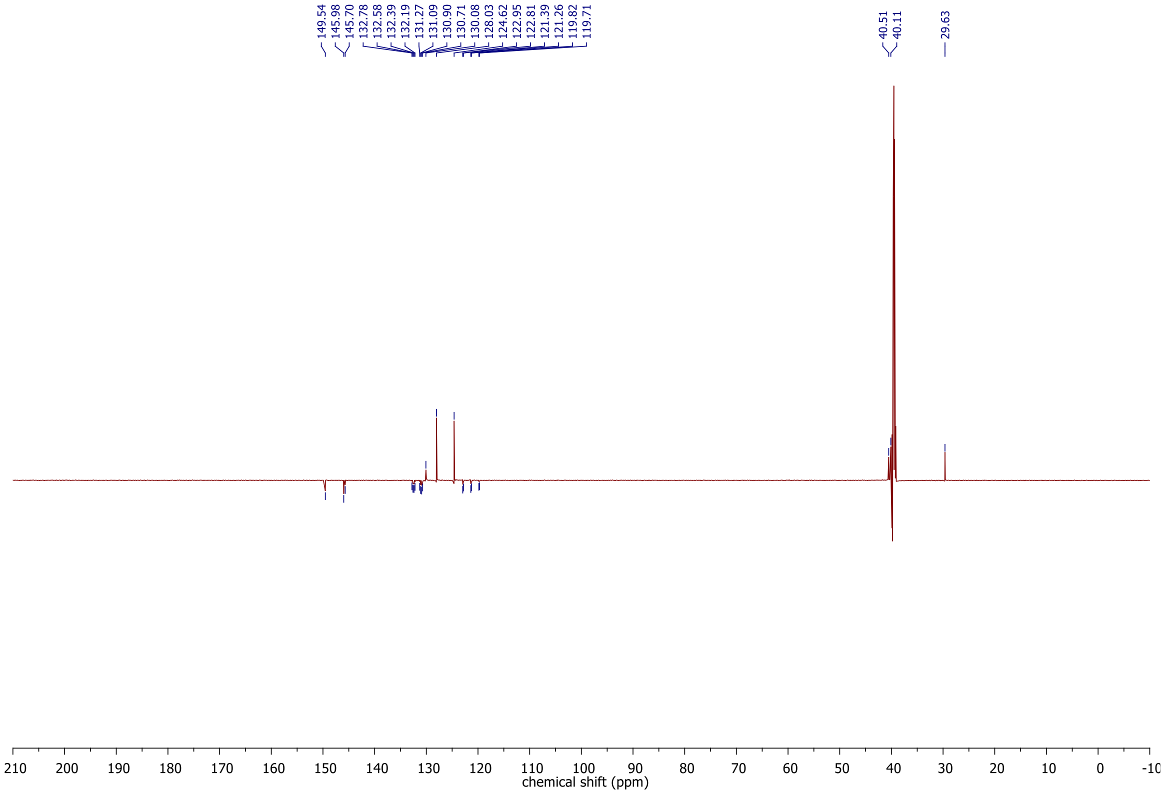

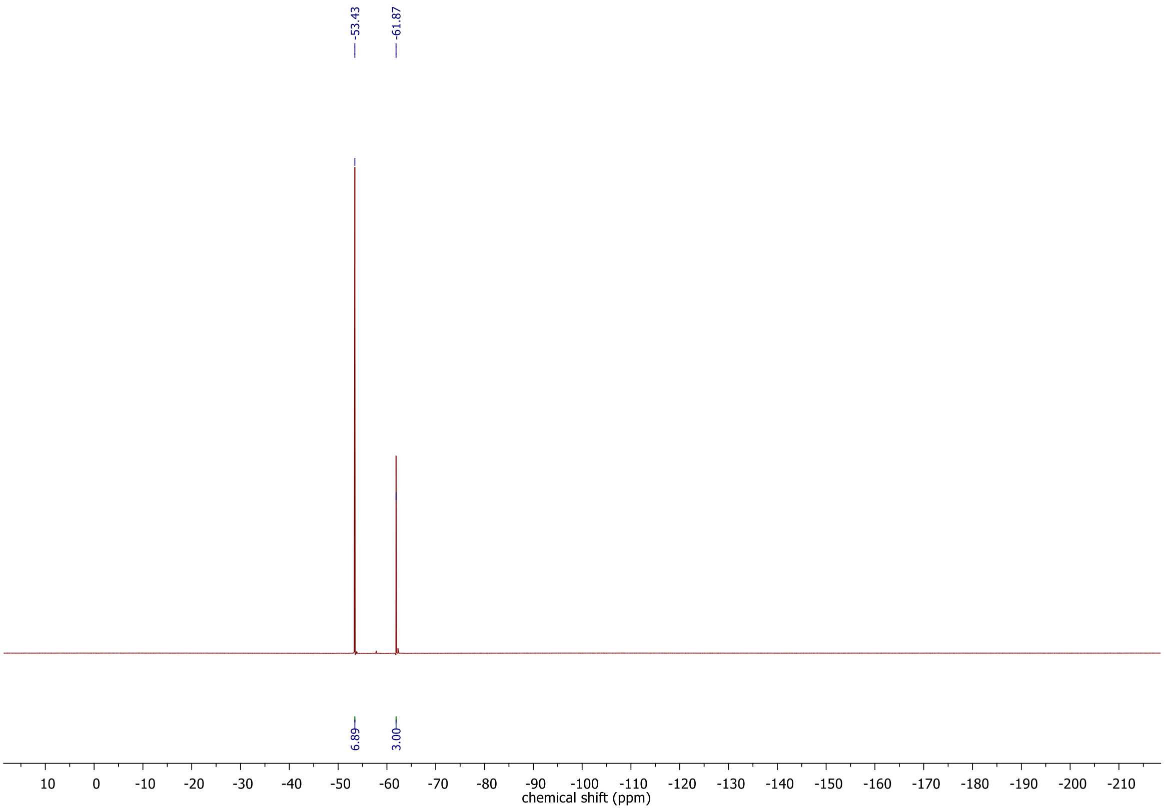
-53.43
-61.87
6.89
3.00
10 0 -10 -20 -30 -40 -50 -60 -70 -80 -90 -100 -110 -120 -130 -140 -150 -160 -170 -180 -190 -200 -210
chemical shift (ppm)

_N_-(3-((4-Cyanophenyl)sulfonamido)propyl)-2,4,6-tris(trifluoromethyl)benzenesulfonamide (13)

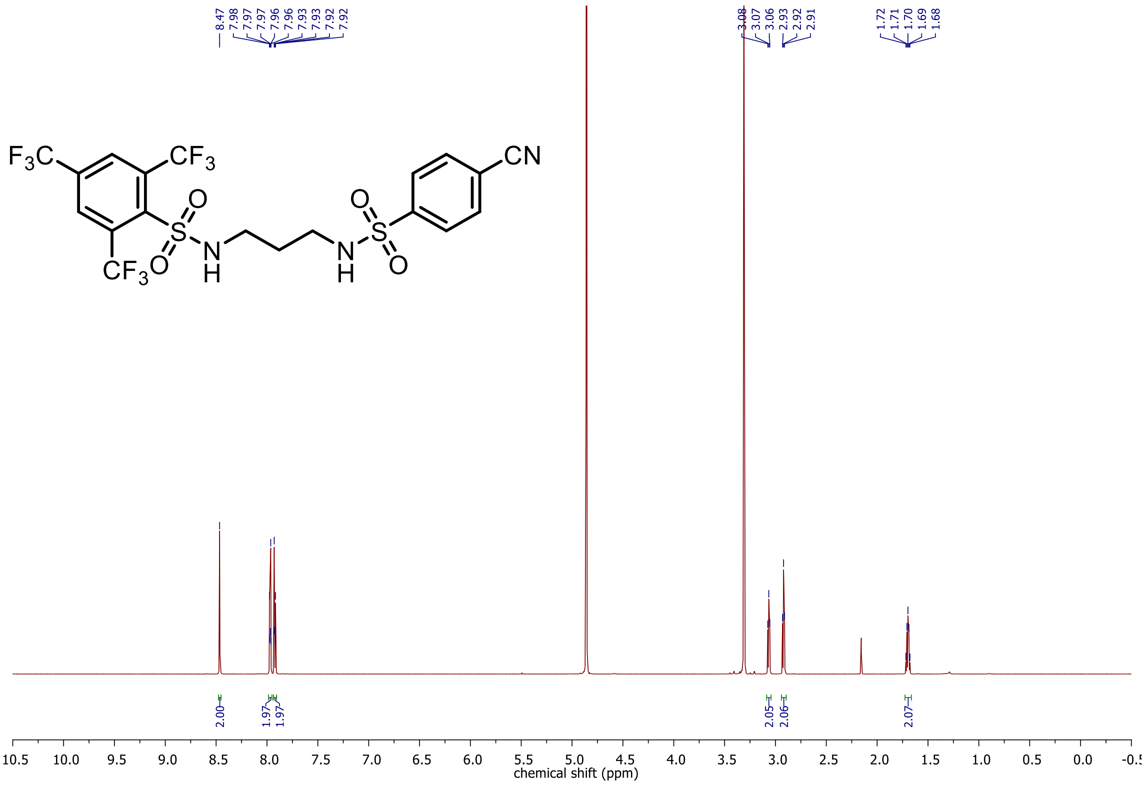

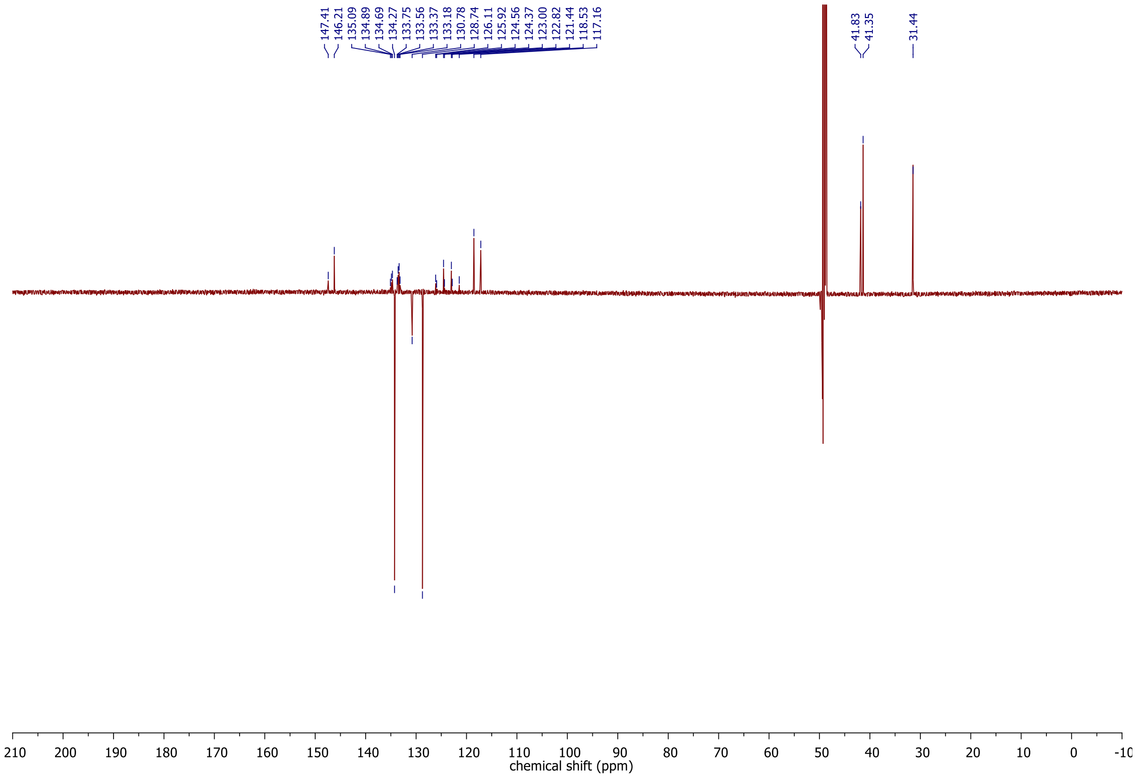

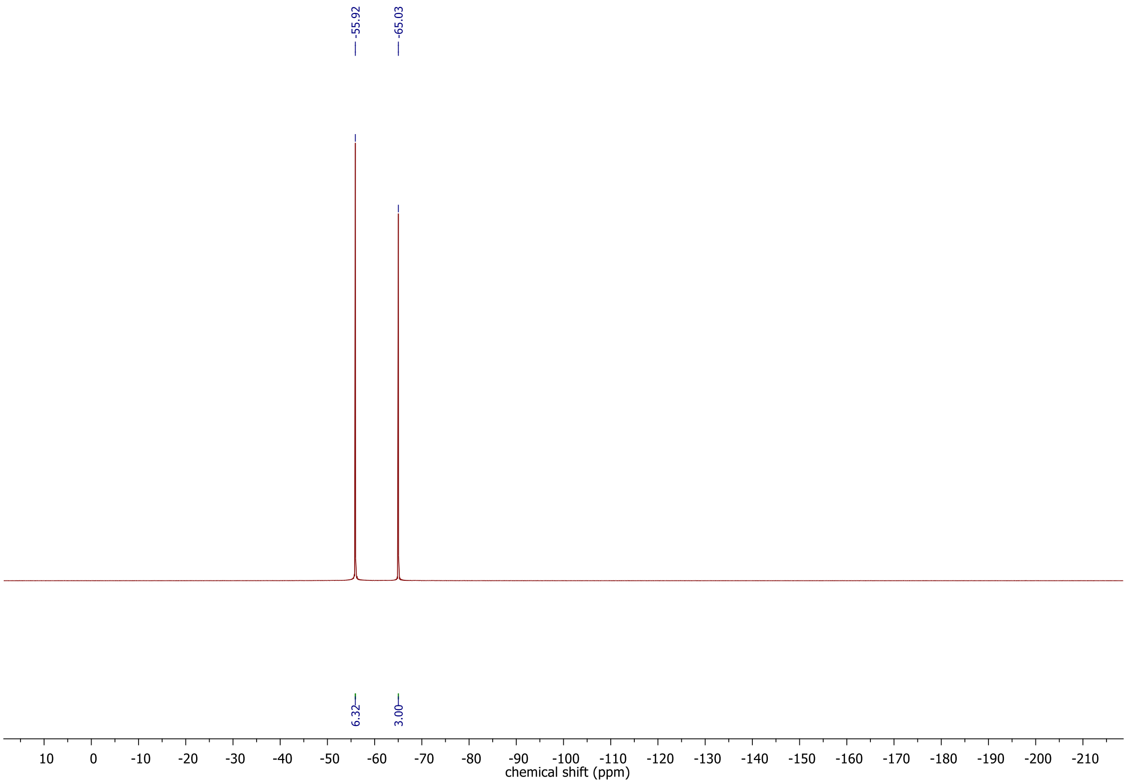

-55.92
-65.03
6.32
3.00
10 0 -10 -20 -30 -40 -50 -60 -70 -80 -90 -100 -110 -120 -130 -140 -150 -160 -170 -180 -190 -200 -210
chemical shift (ppm)

_N_-(3-((4-Methylphenyl)sulfonamido)propyl)-2,4,6-tris(trifluoromethyl)benzenesulfonamide (14)

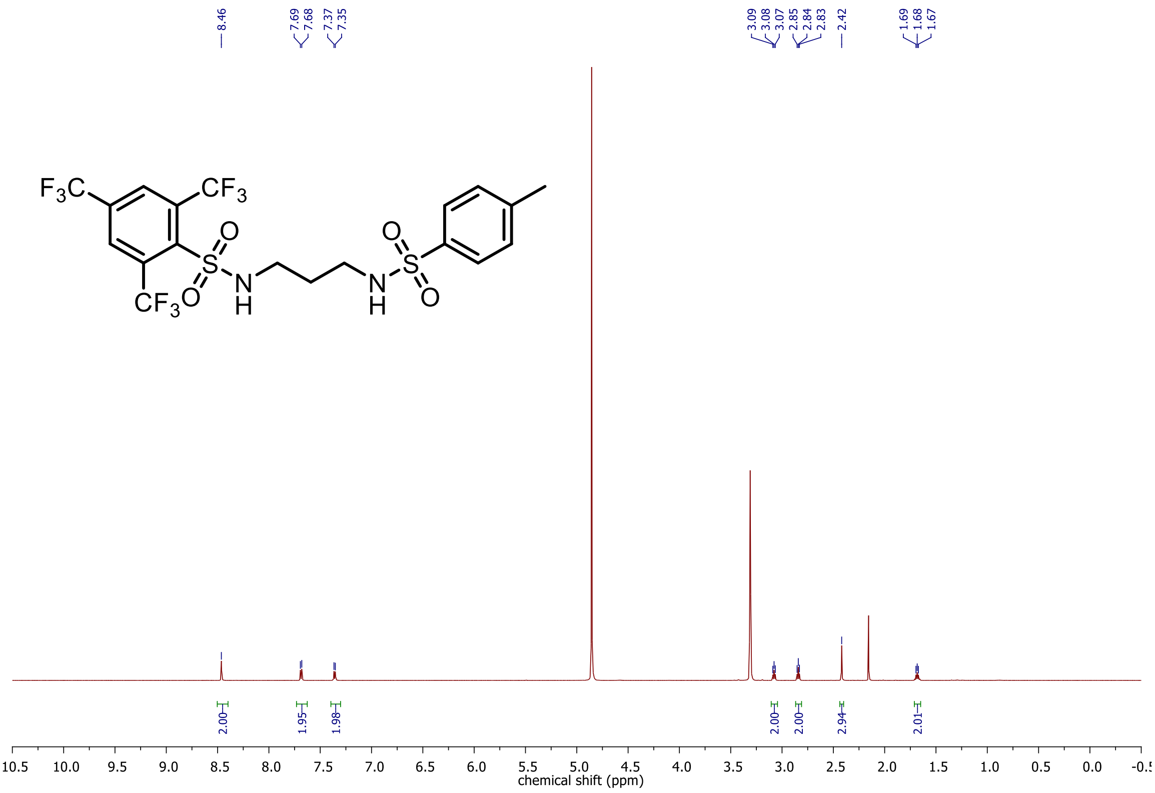

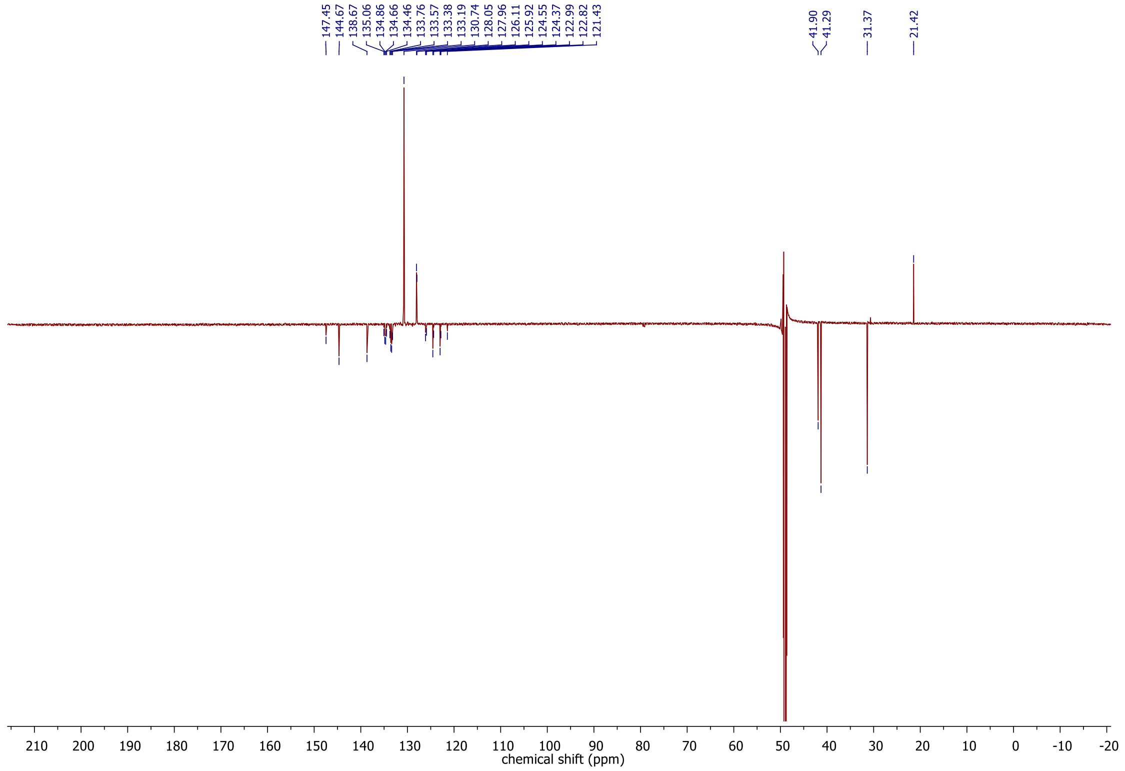

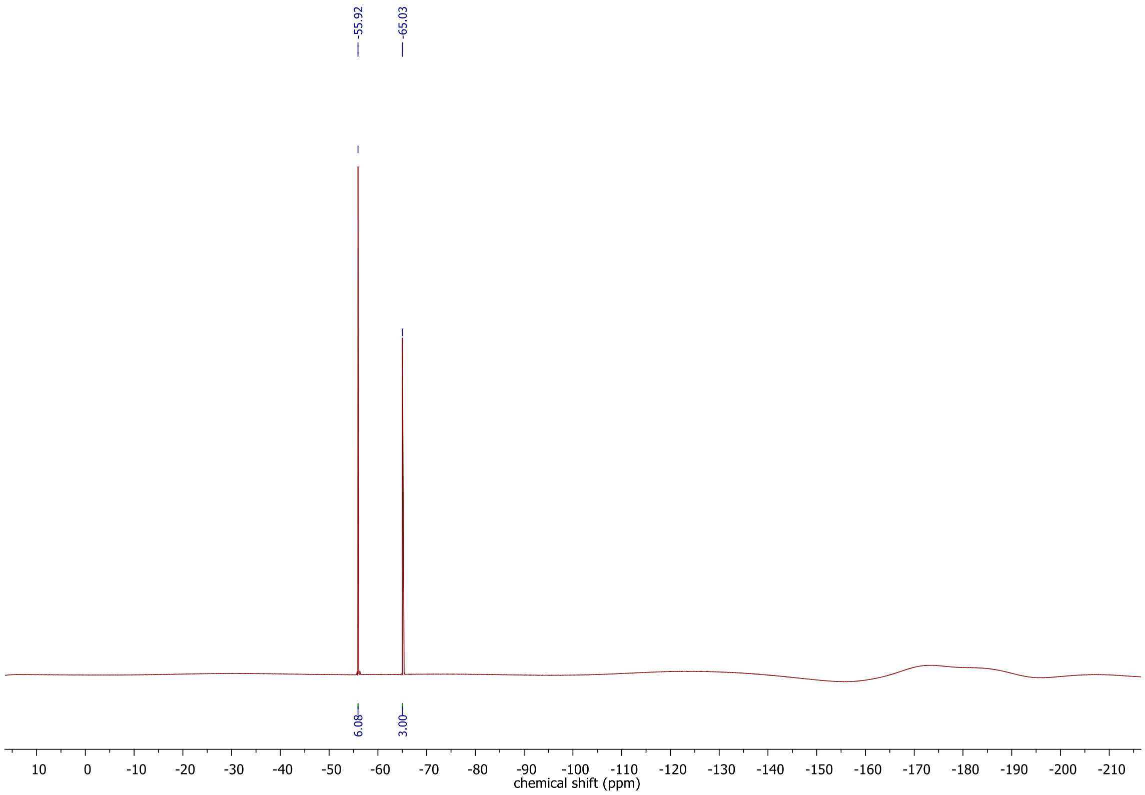
-55.92
-65.03
6.08
3.00
10 0 -10 -20 -30 -40 -50 -60 -70 -80 -90 -100 -110 -120 -130 -140 -150 -160 -170 -180 -190 -200 -210
chemical shift (ppm)

***N*-(3-Aminopropyl)-4-nitrobenzenesulfonamide (16)**

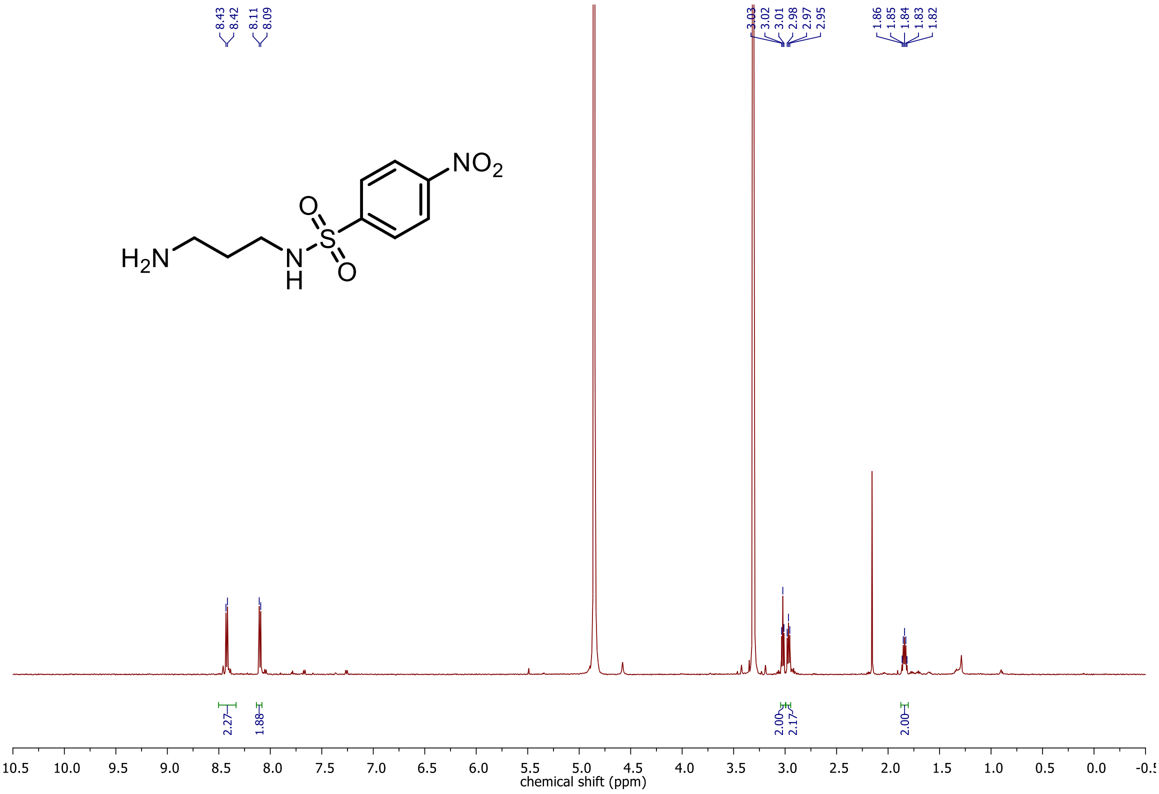

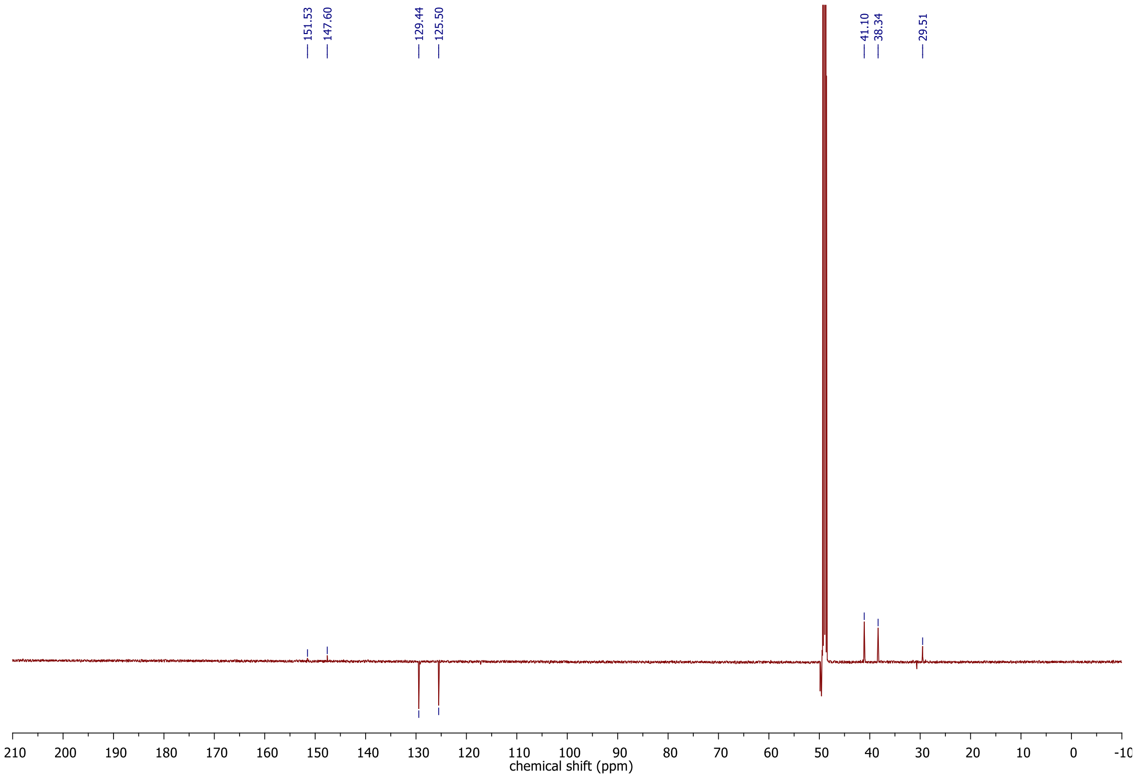

N-(3-Aminopropyl)-4-cyanobenzenesulfonamide (17)

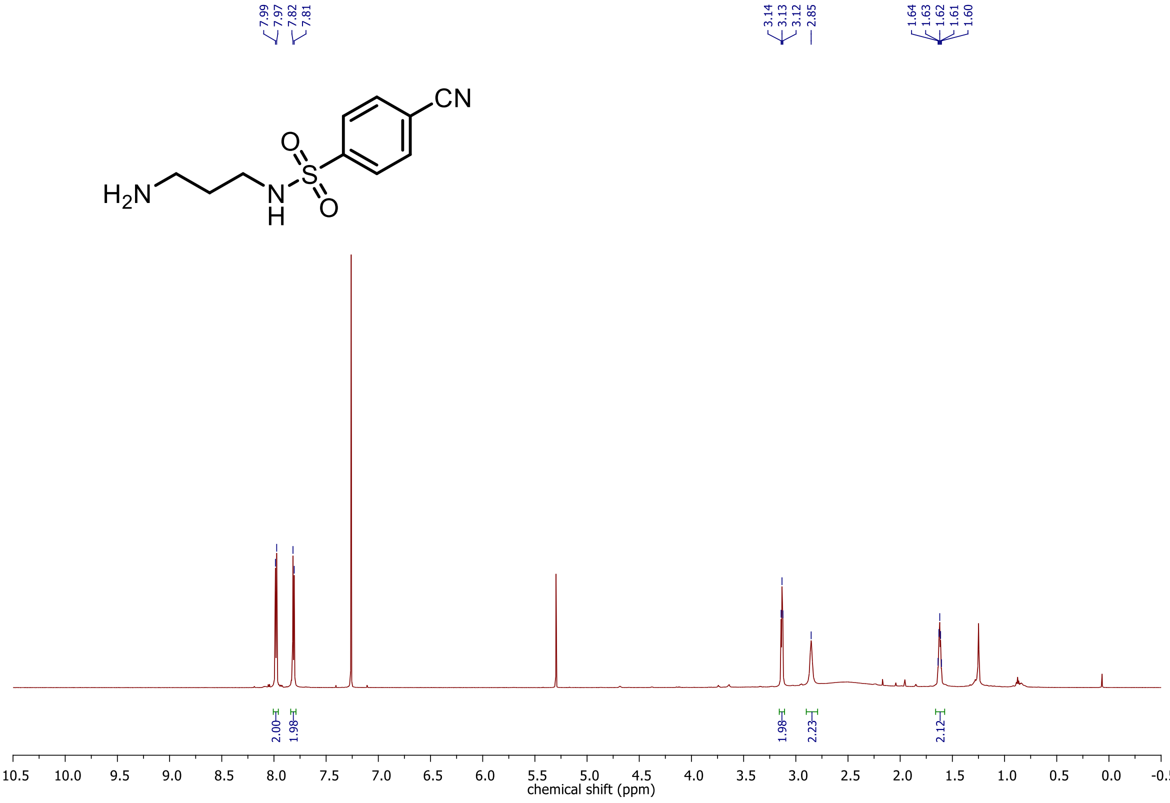

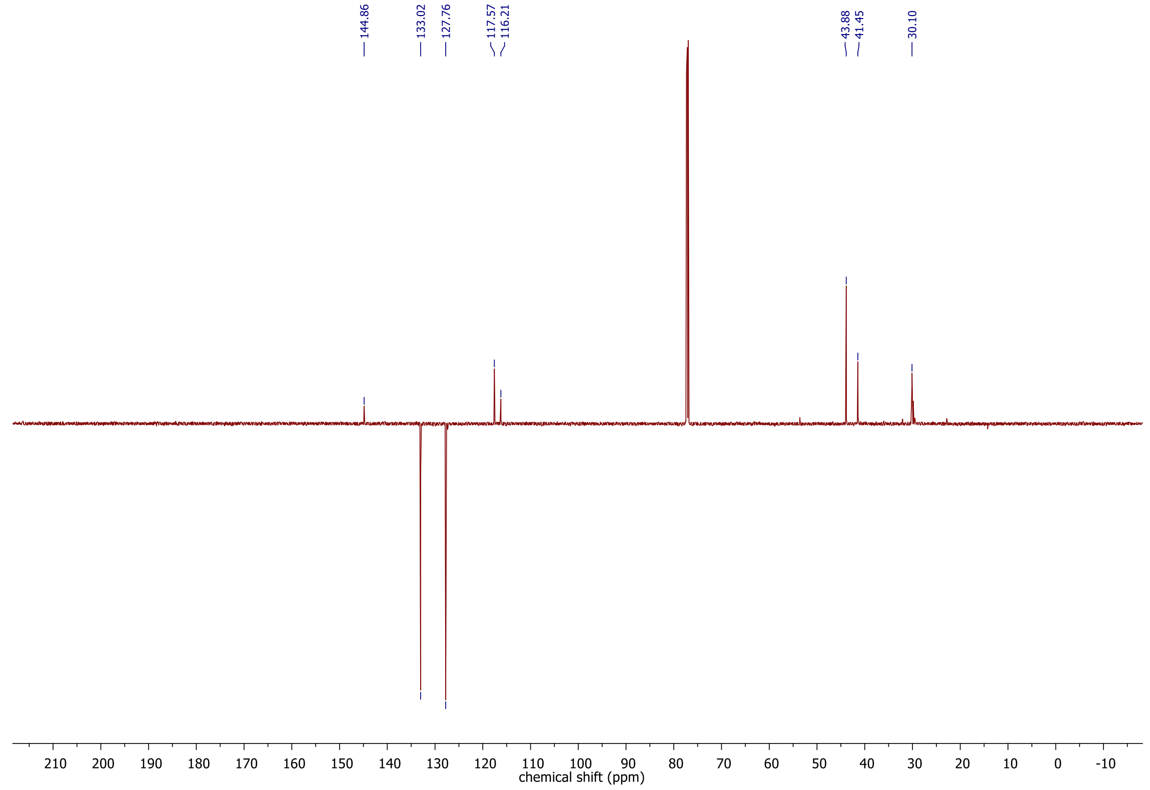

N-methoxy-N-methyl-1-((4-nitrophenyl)sulfonyl)piperidine-4-carboxamide (19)

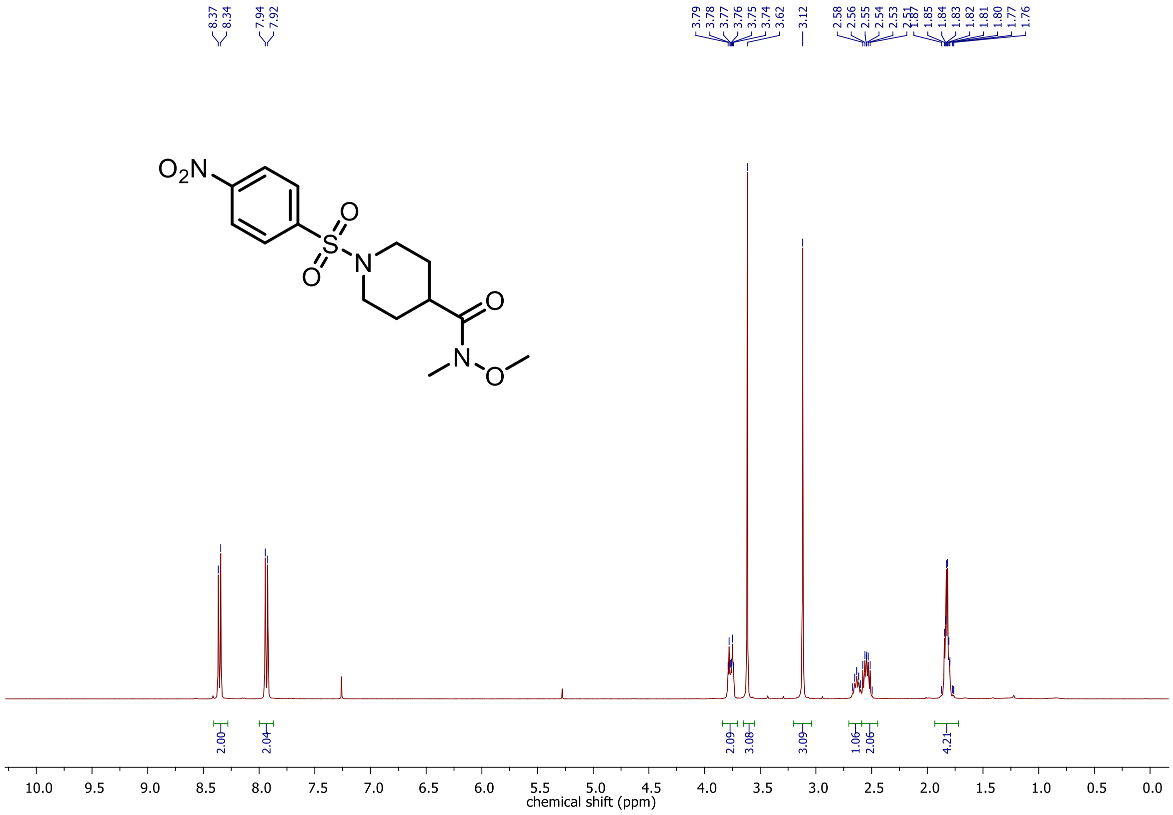

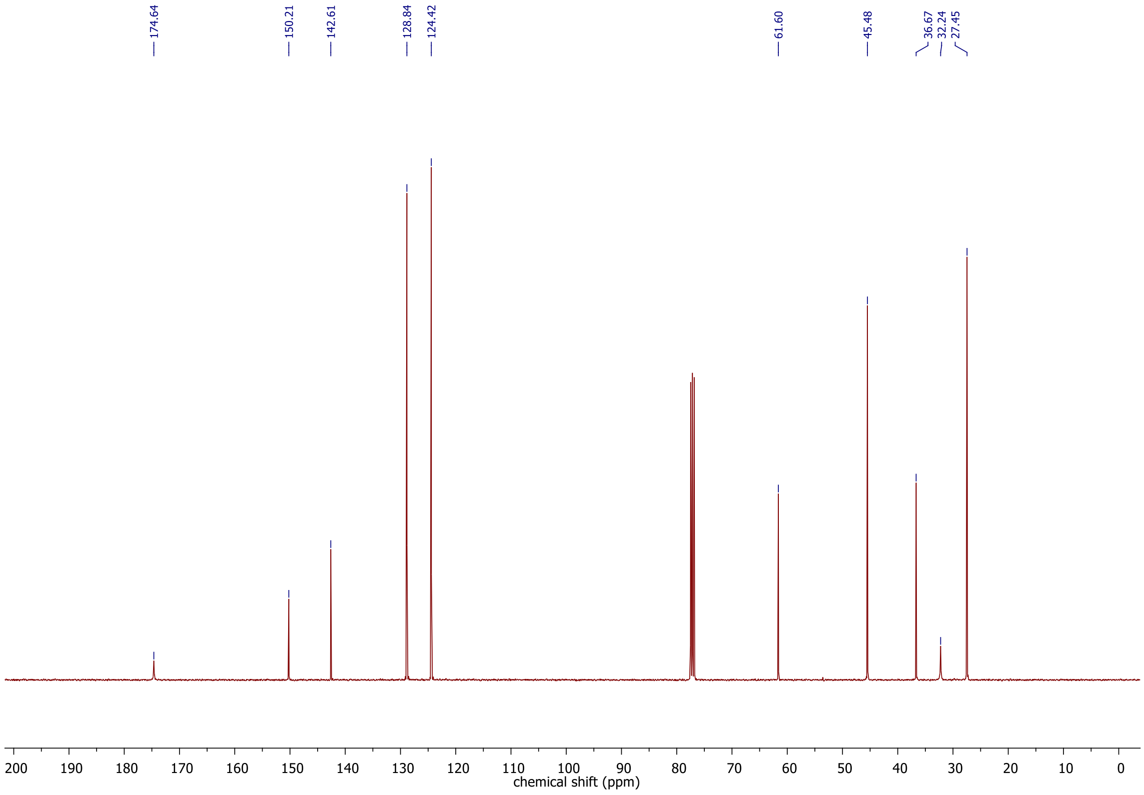

1-((4-Cyanophenyl)sulfonyl)-N-methoxy-N-methylpiperidine-4-carboxamide (20)

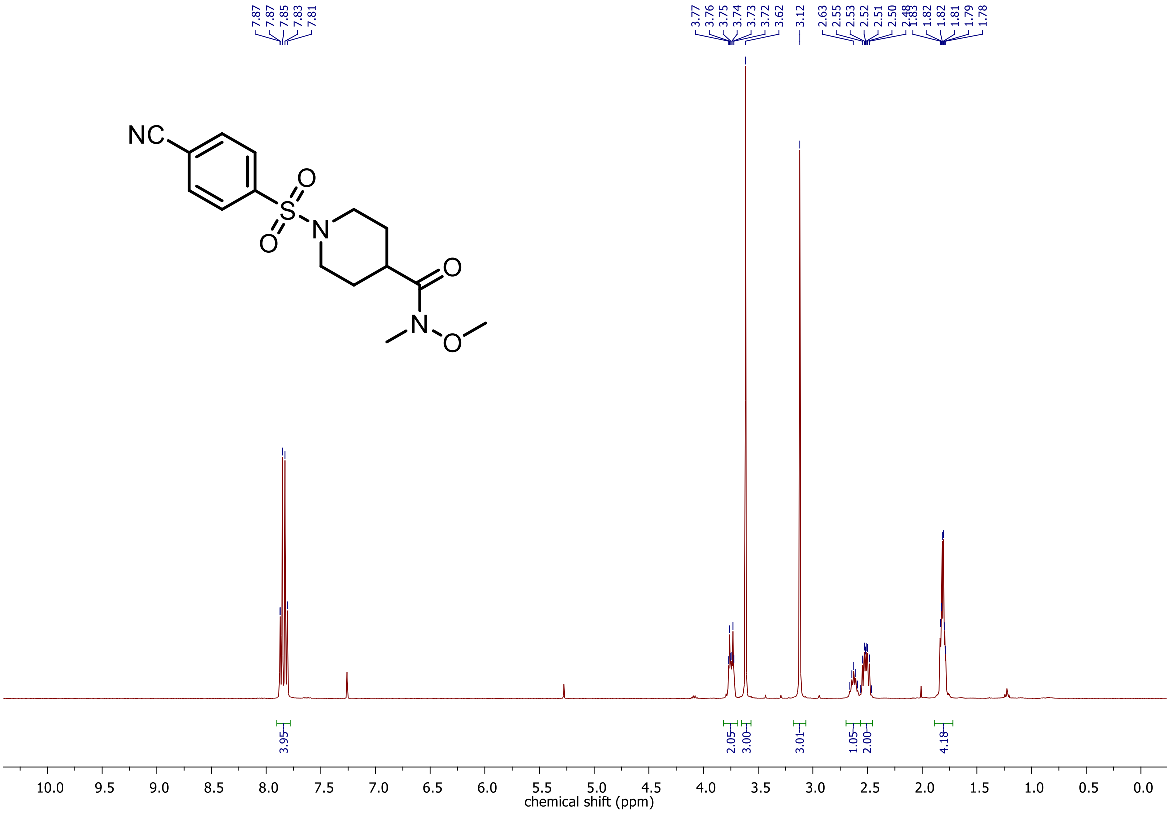

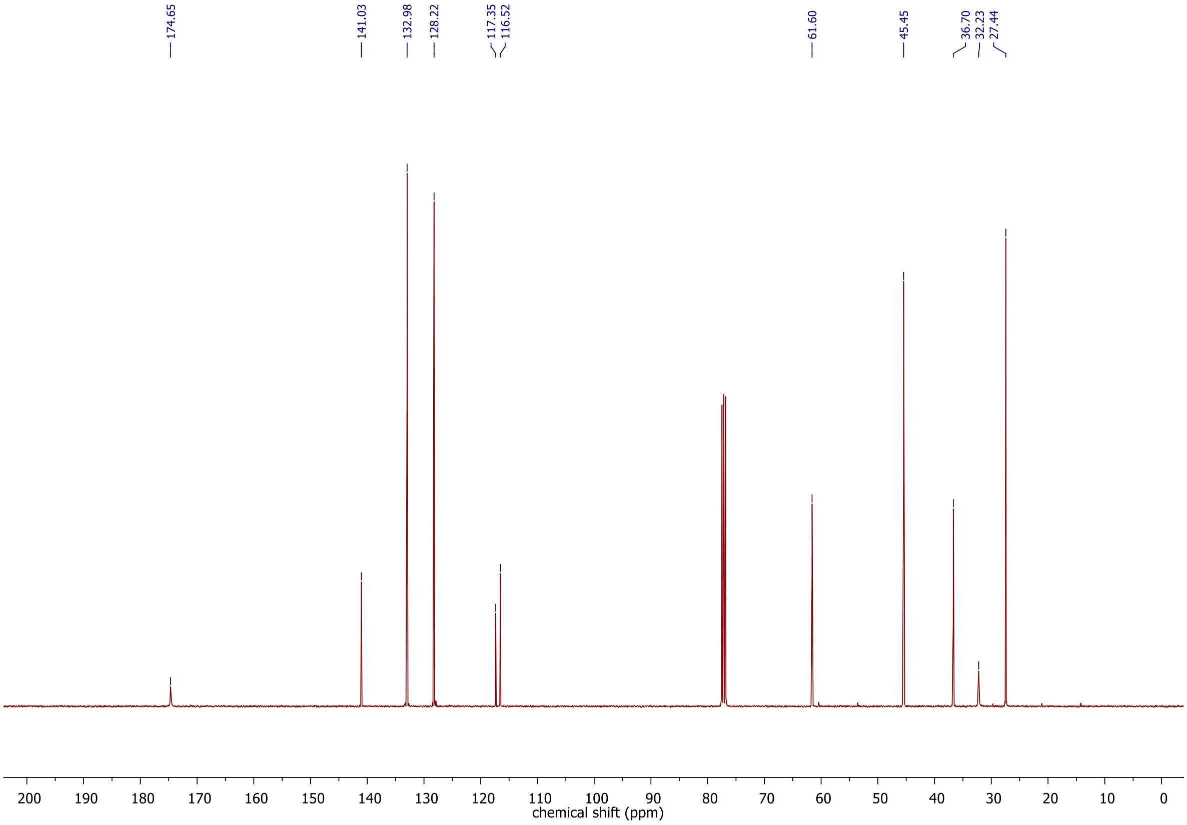

N-methoxy-N-methyl-1-tosylpiperidine-4-carboxamide (21)

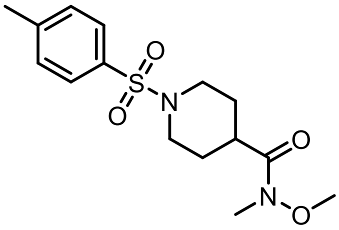

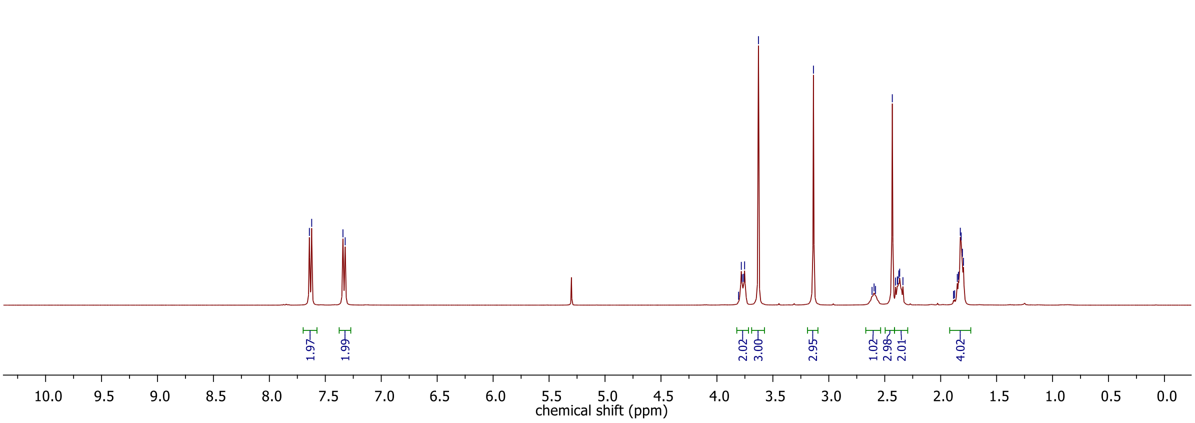

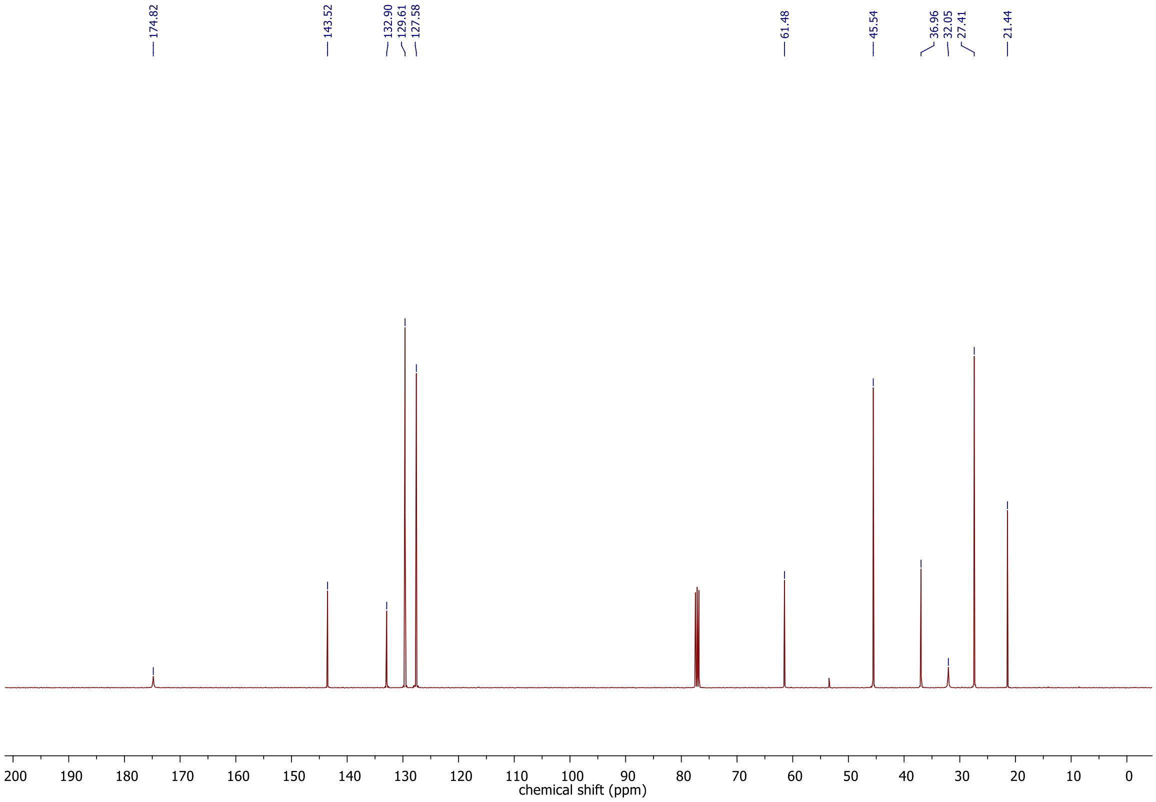

Phenyl(1-((2,4,6-tris(trifluoromethyl)phenyl)sulfonyl)piperidin-4-yl)methanone (23)

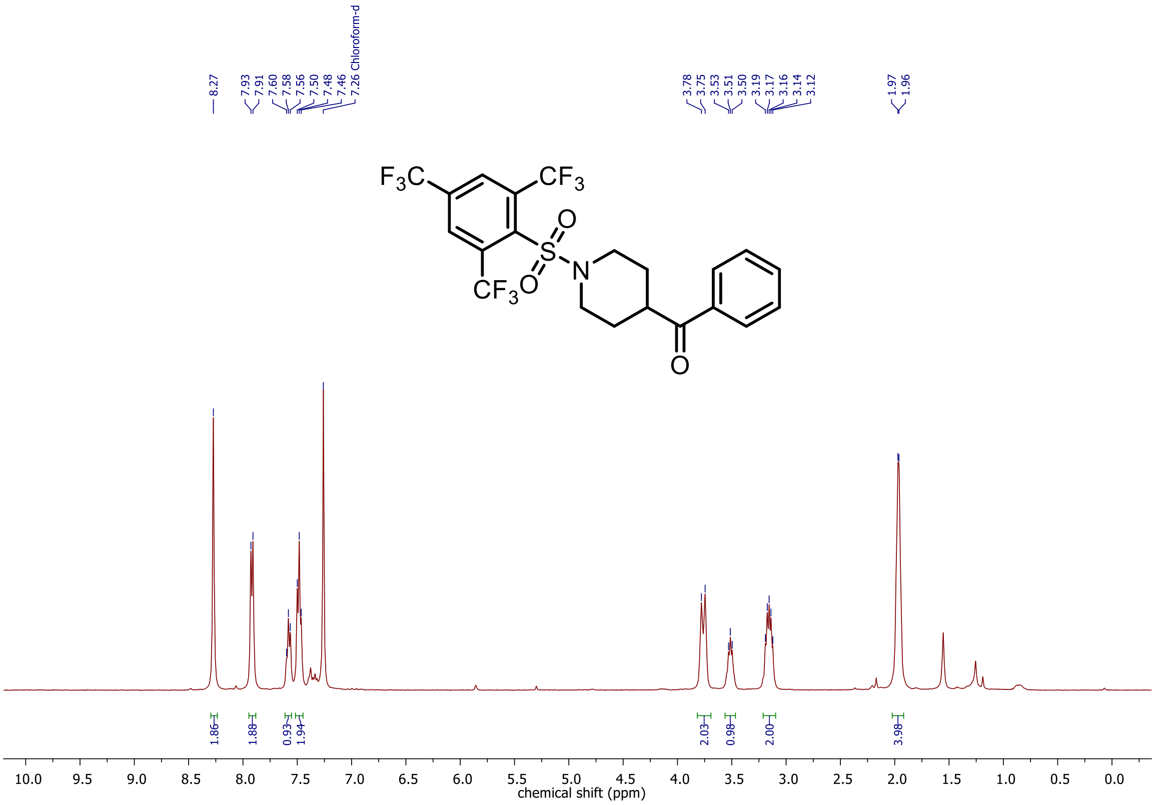

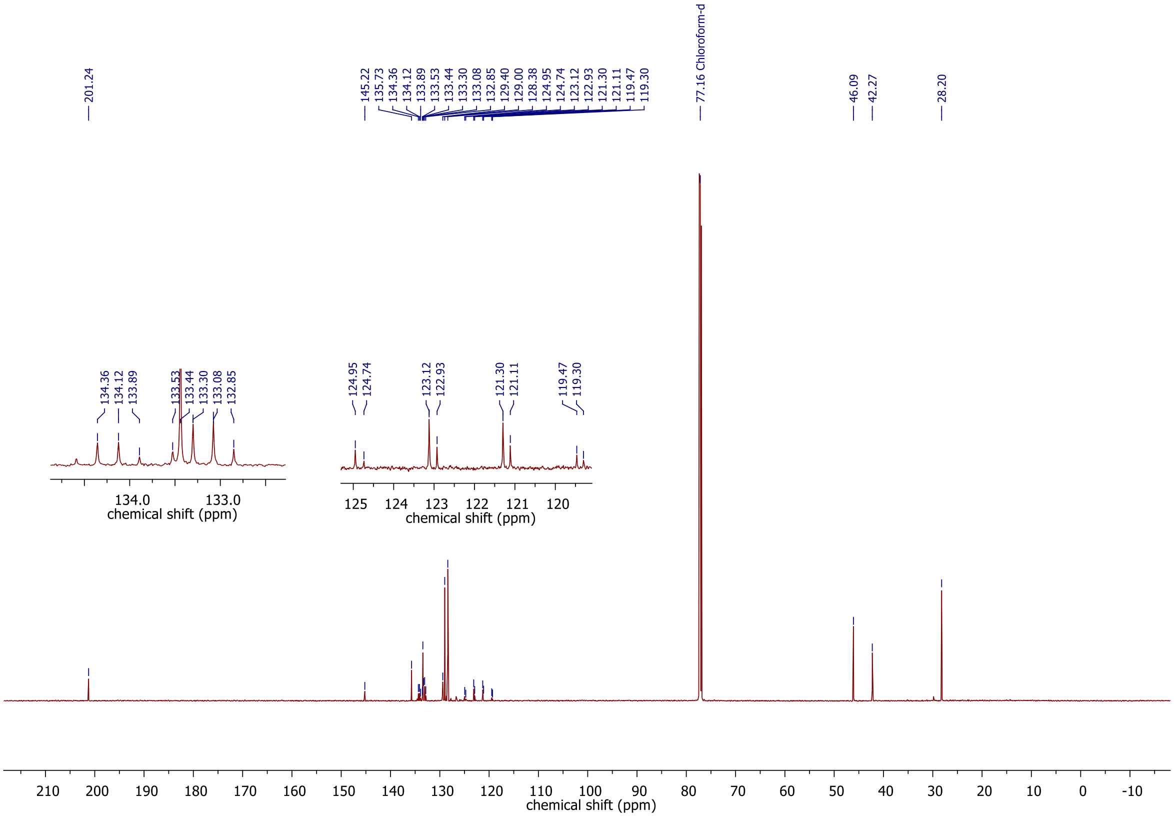

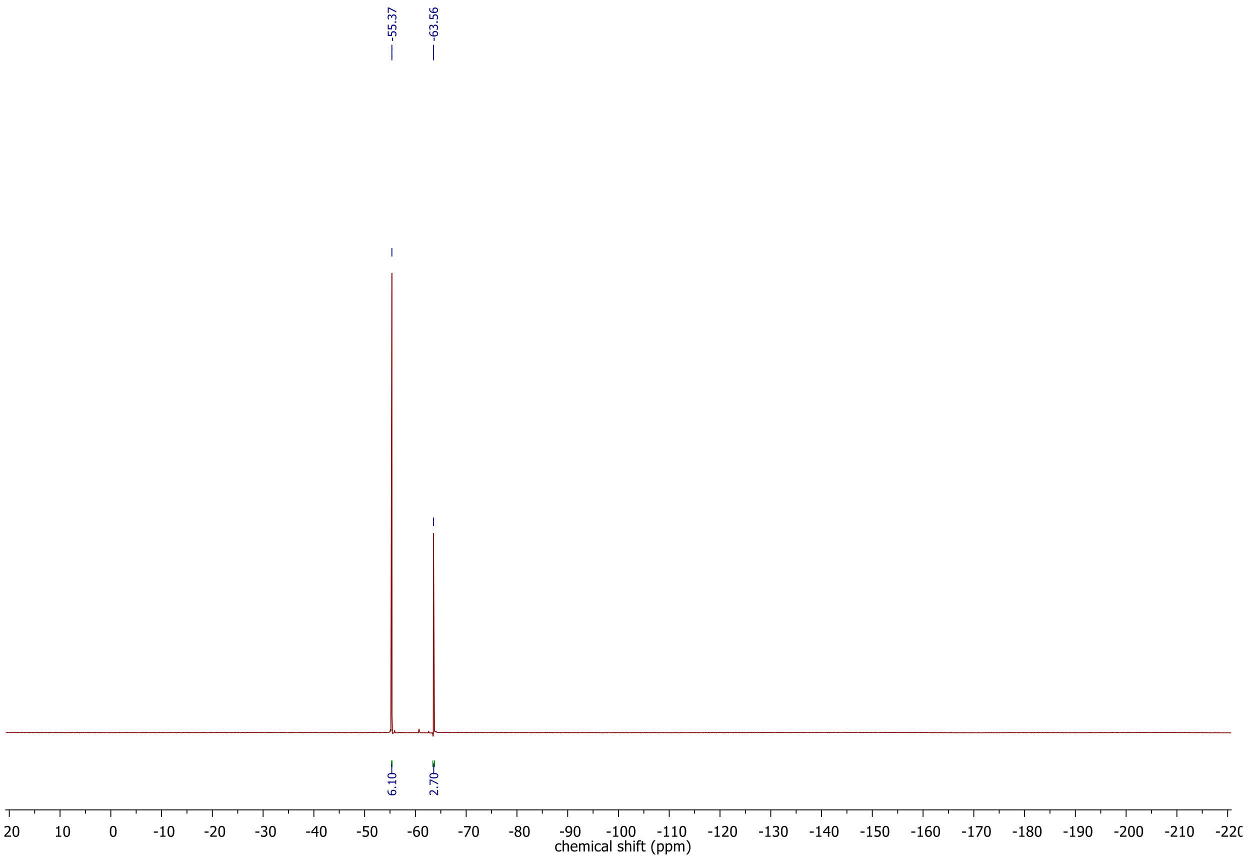
-55.37
-63.56
6.10
2.70
20 10 0 -10 -20 -30 -40 -50 -60 -70 -80 -90 -100 -110 -120 -130 -140 -150 -160 -170 -180 -190 -200 -210 -220
chemical shift (ppm)

Phenyl(1-((4-(phenylamino)phenyl)sulfonyl)piperidin-4-yl)methanone (24’)

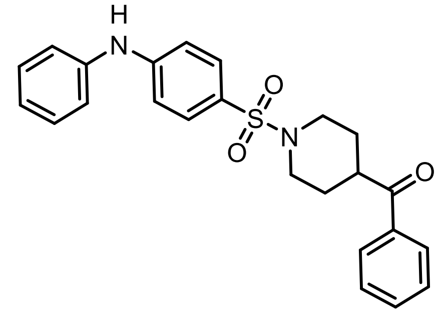

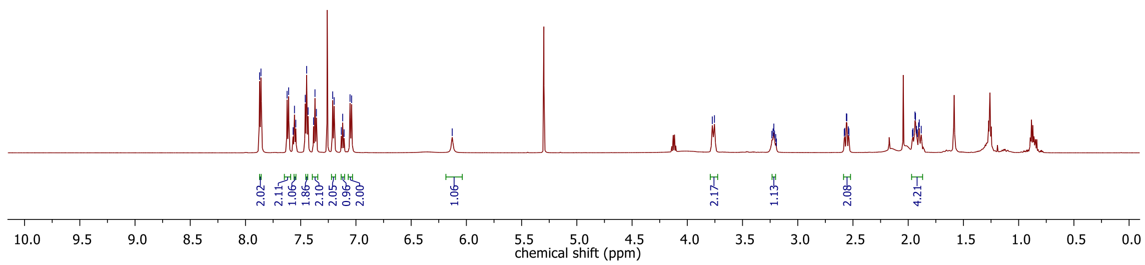

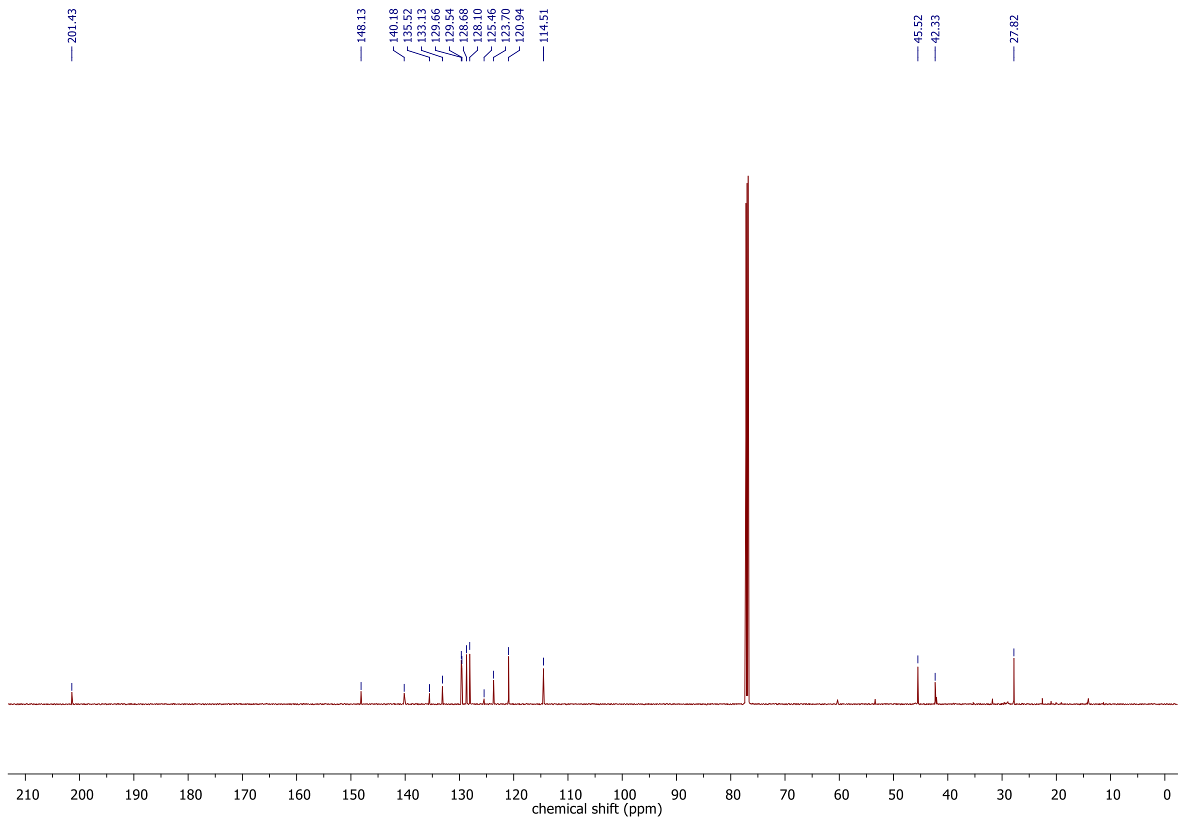

N-methoxy-N-methyl-1-((4-(phenylamino)phenyl)sulfonyl)piperidine-4-carboxamide (24'')

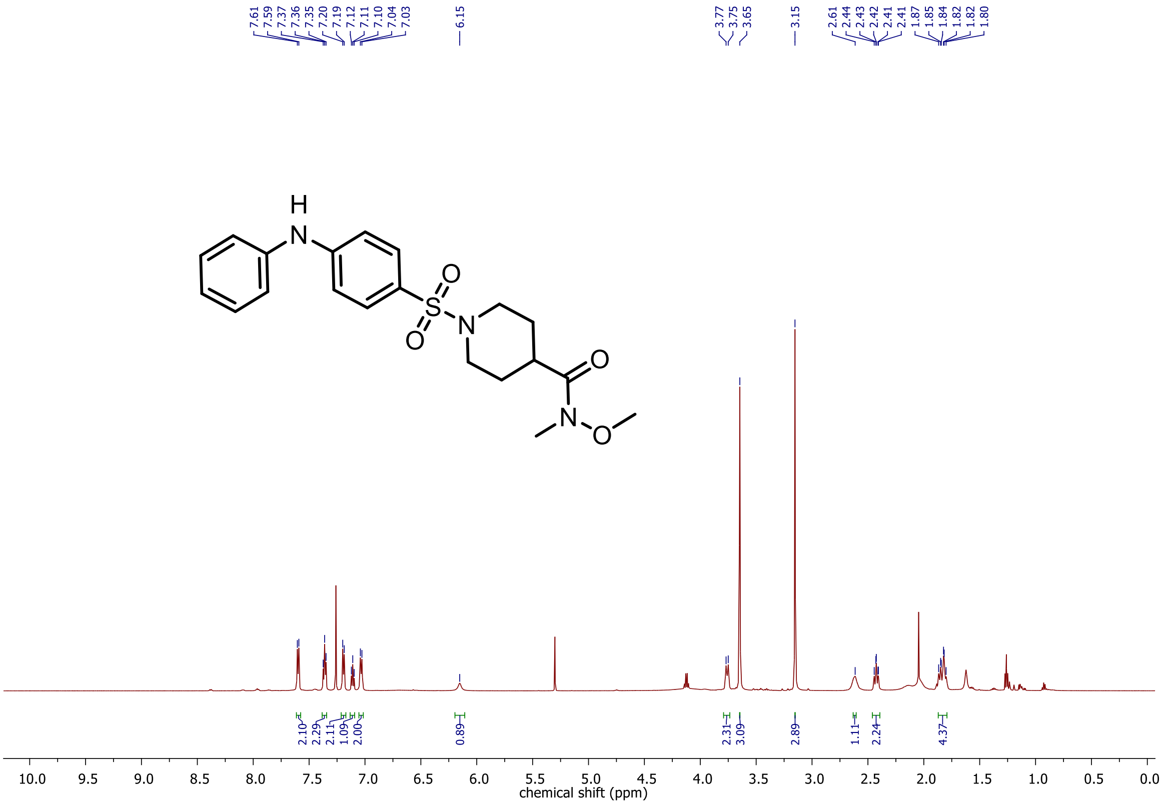

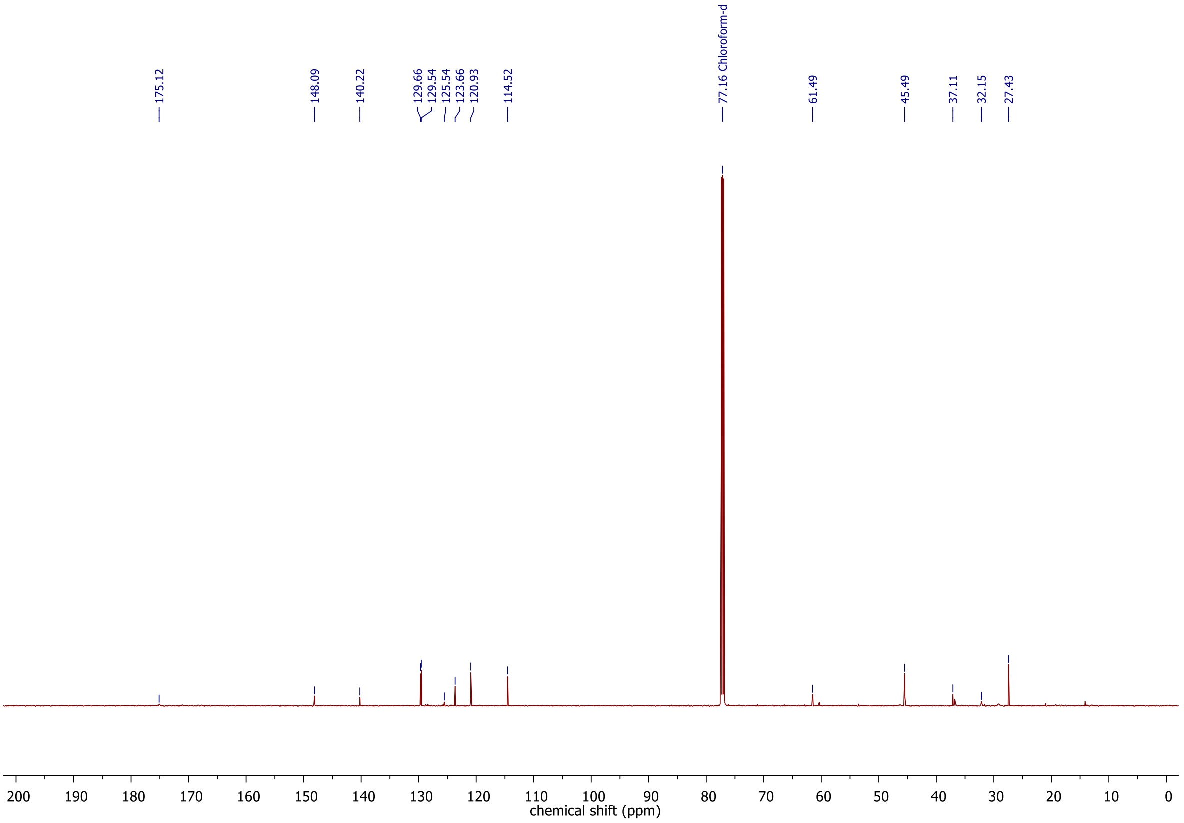

4-((4-Benzoylpiperidin-1-yl)sulfonyl)benzonitrile (25)

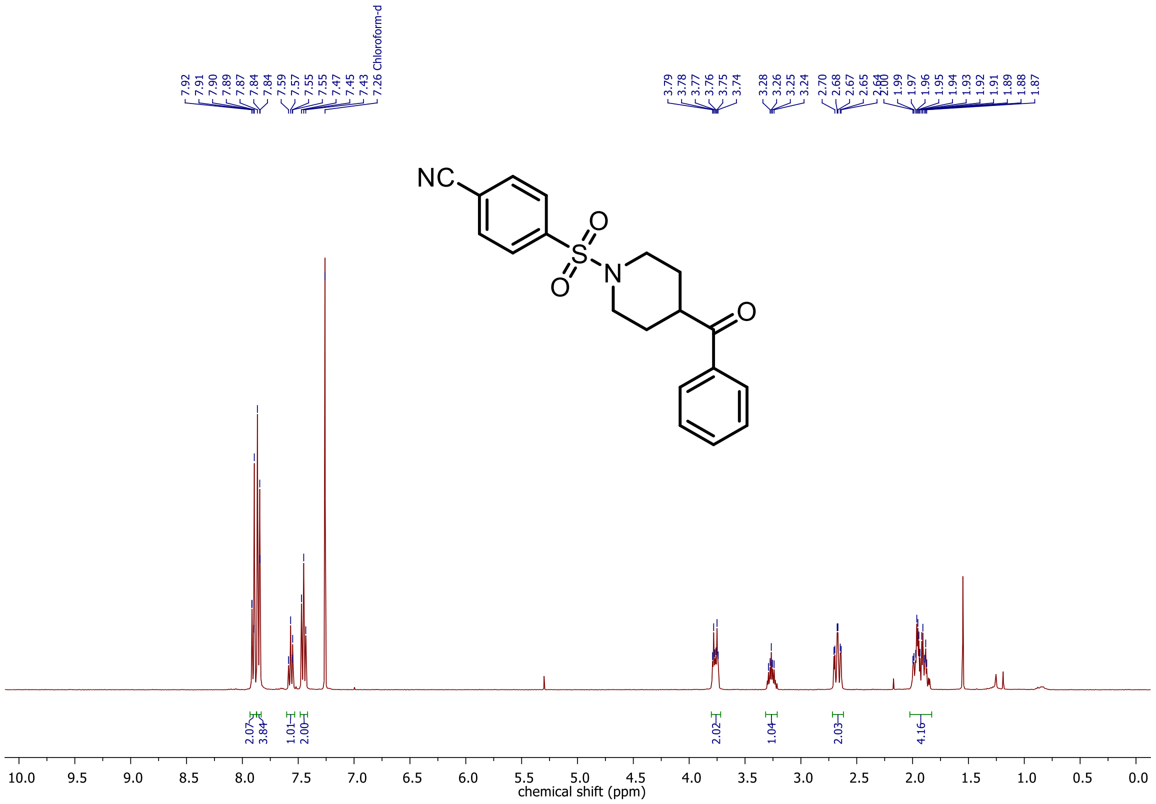

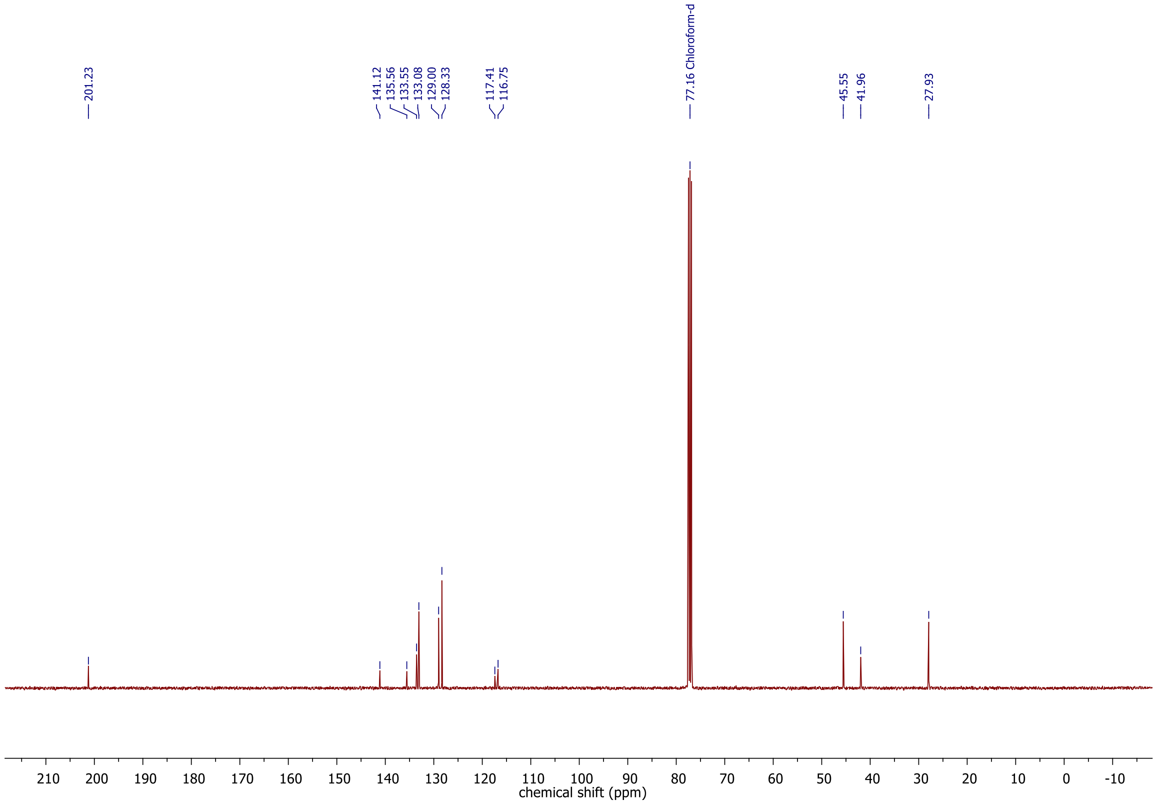

(1-((4-Benzoylphenyl)sulfonyl)piperidin-4-yl)(phenyl)methanone (25’)

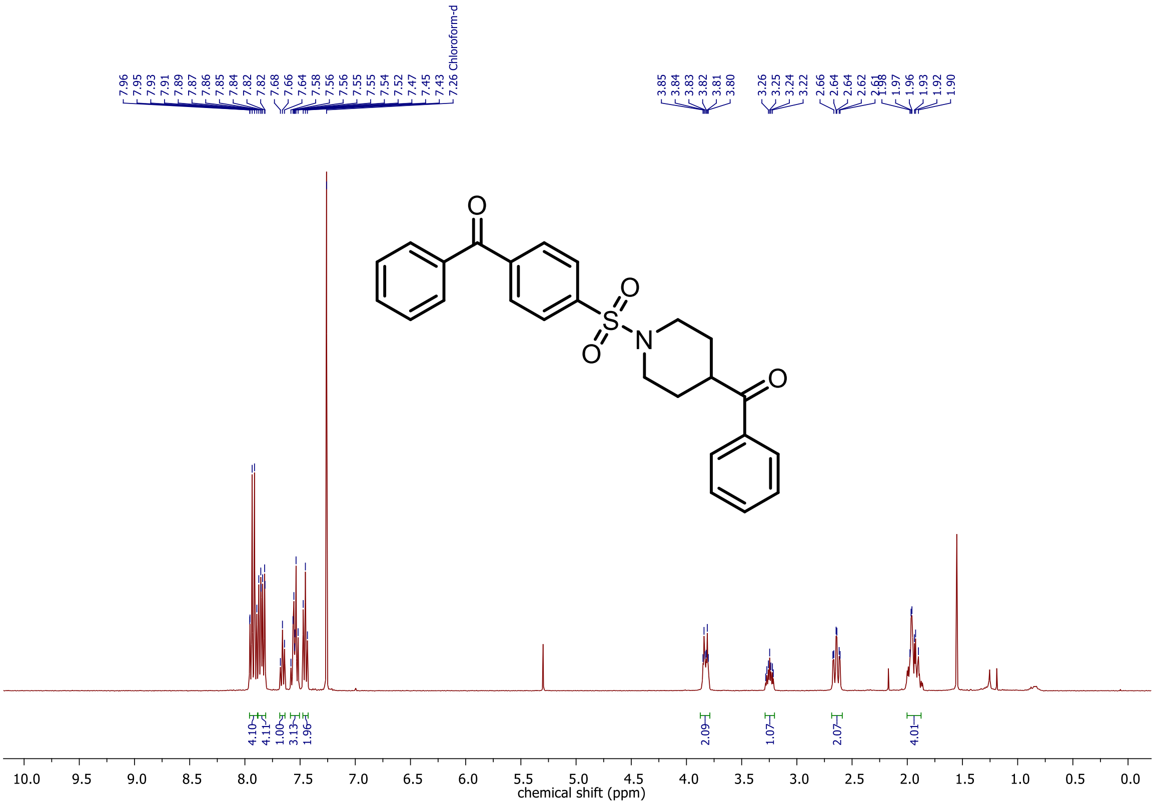

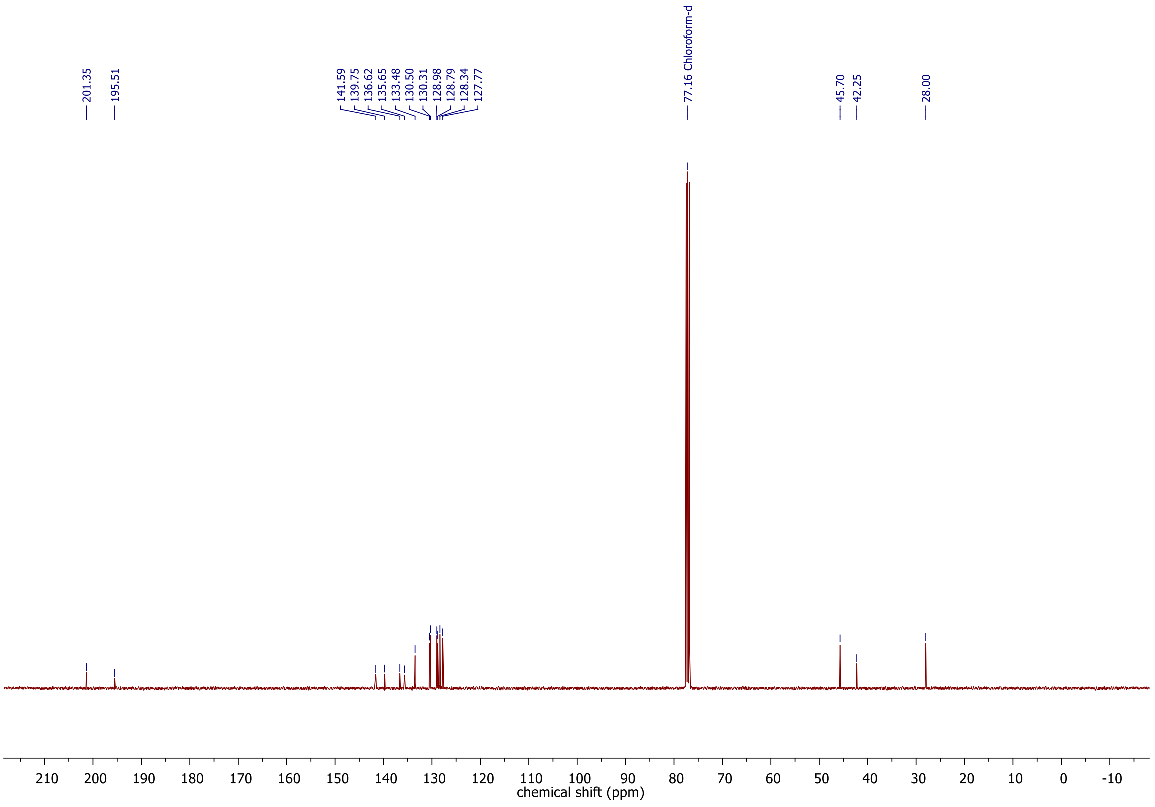

Phenyl(1-tosylpiperidin-4-yl)methanone (26)

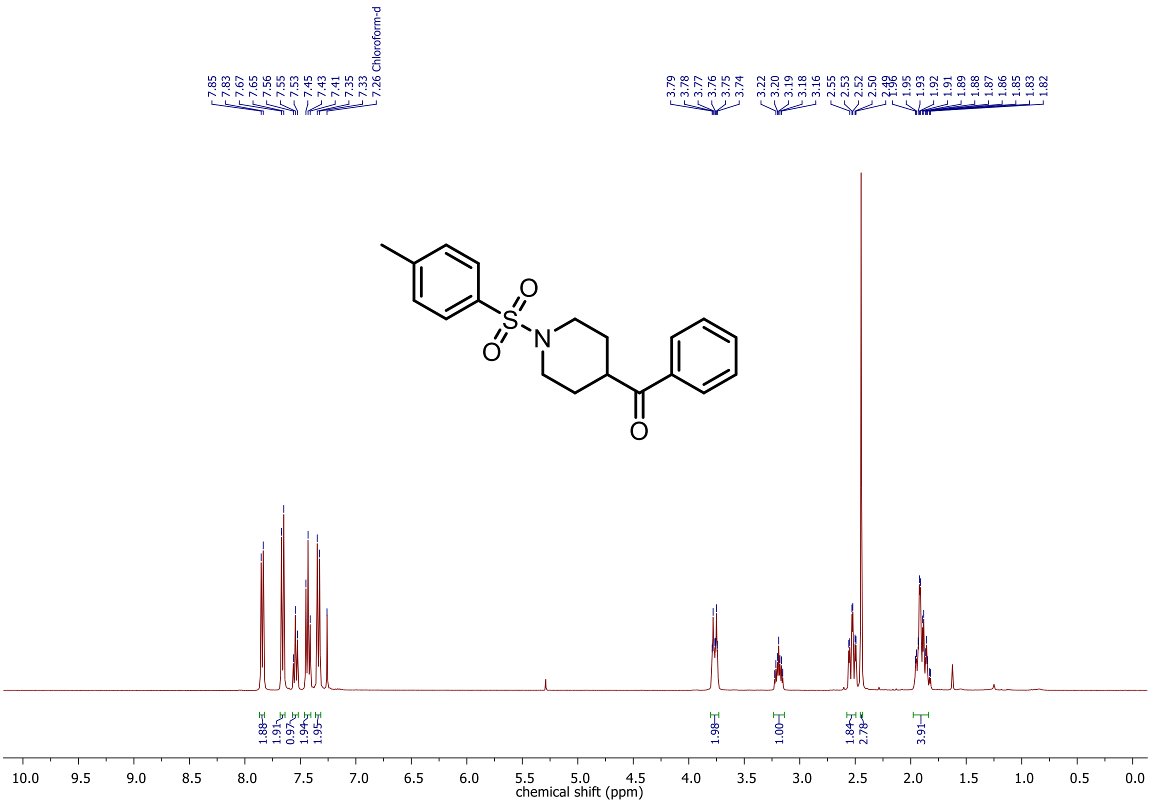

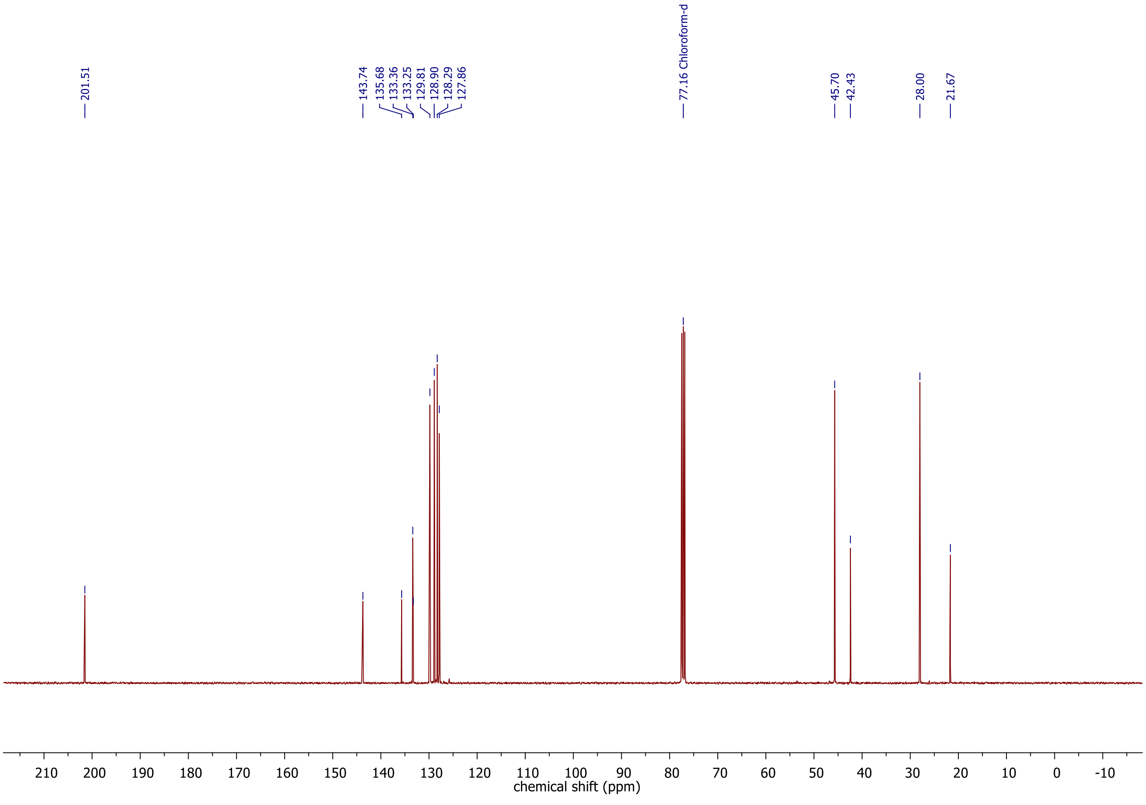

Phenyl(1-tosylpiperidin-4-yl)methanone (26’)

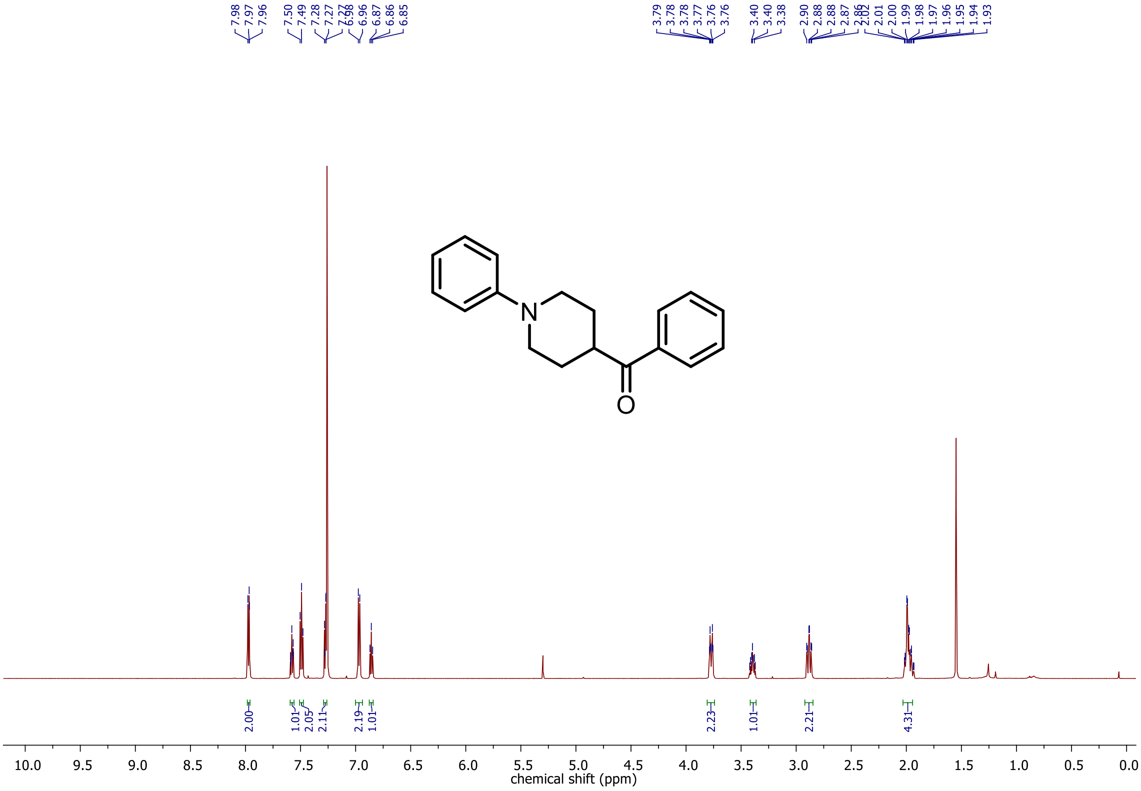

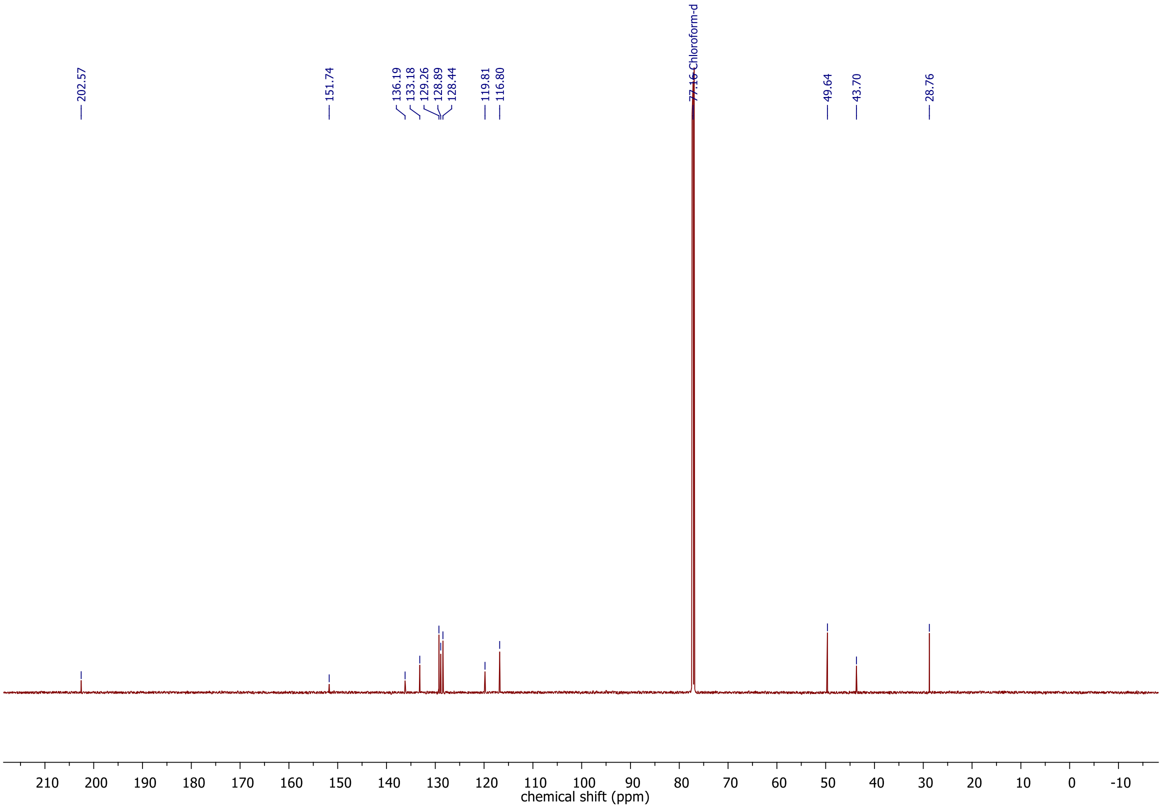

1-(1-((2,4,6-Tris(trifluoromethyl)phenyl)sulfonyl)piperidin-4-yl)ethan-1-one (28)

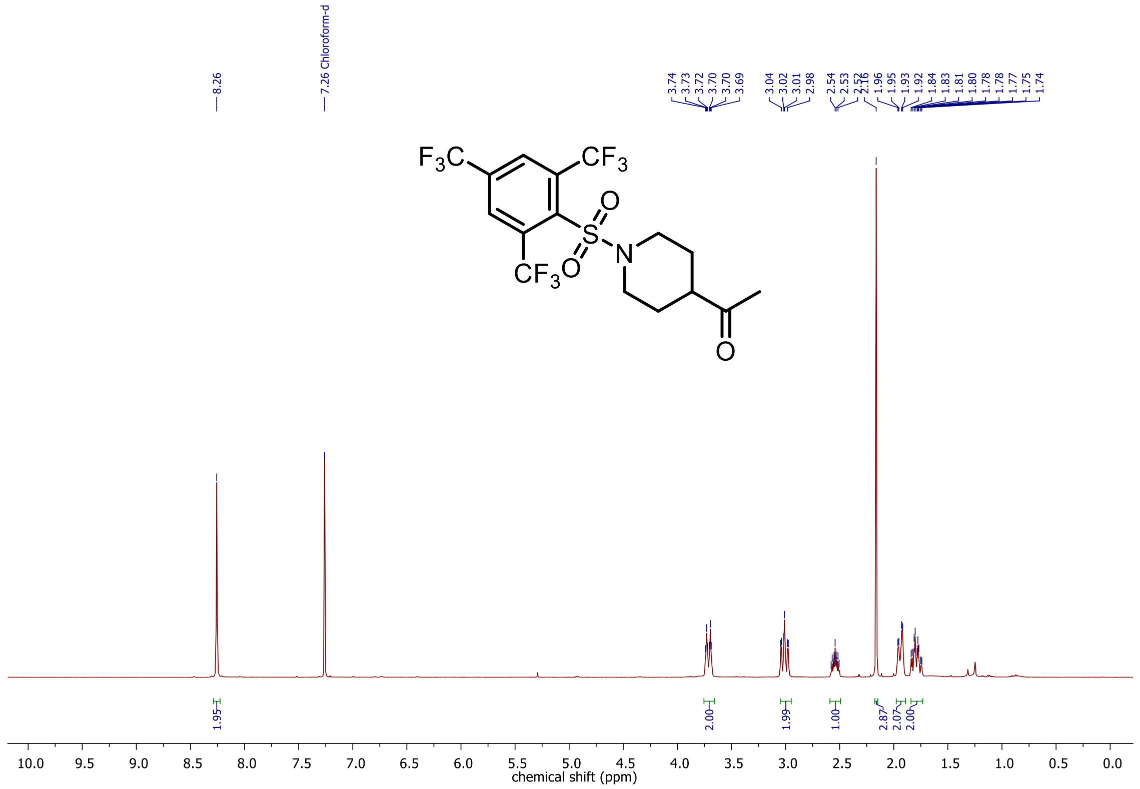

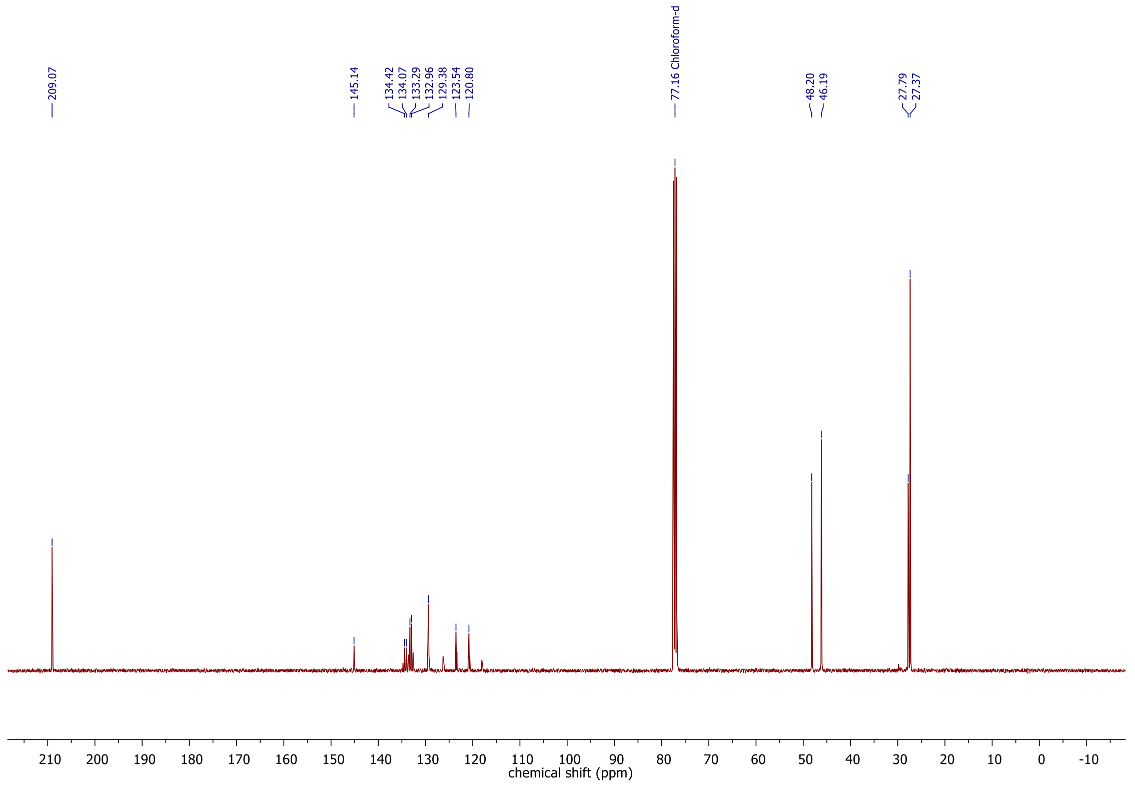

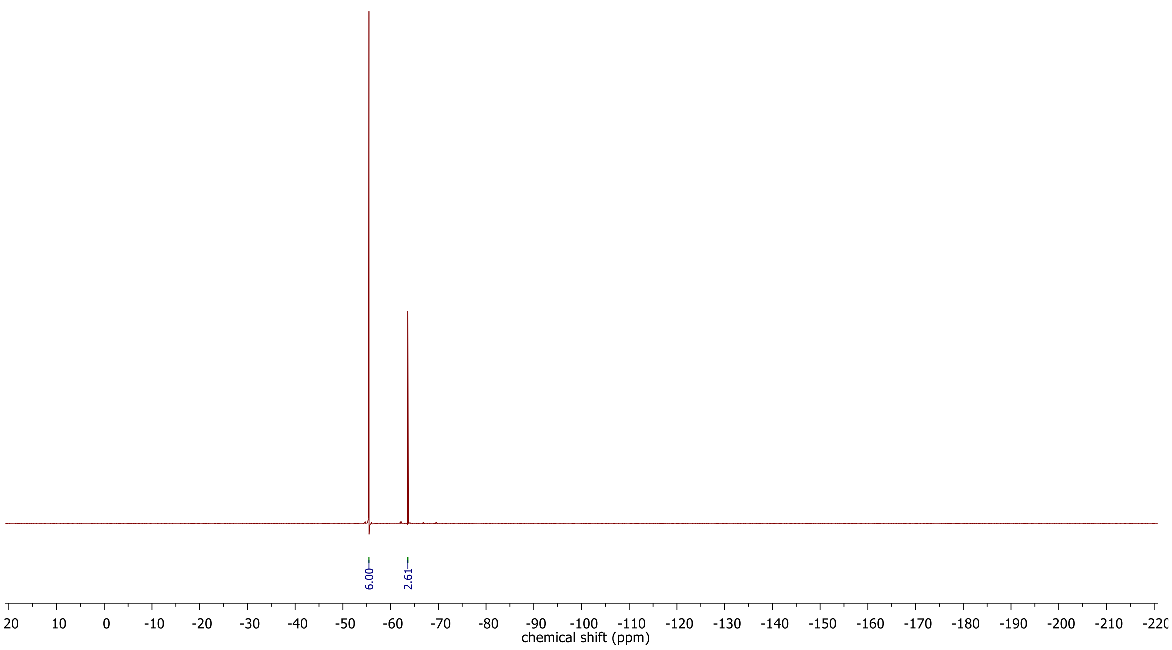
6.00
2.61
20 10 0 -10 -20 -30 -40 -50 -60 -70 -80 -90 -100 -110 -120 -130 -140 -150 -160 -170 -180 -190 -200 -210 -220
chemical shift (ppm)

1-(4-Nitrophenyl)-4-((4-nitrophenyl)sulfonyl)piperazine (29)

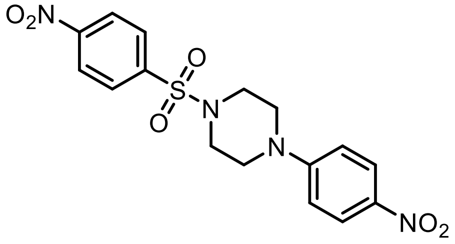

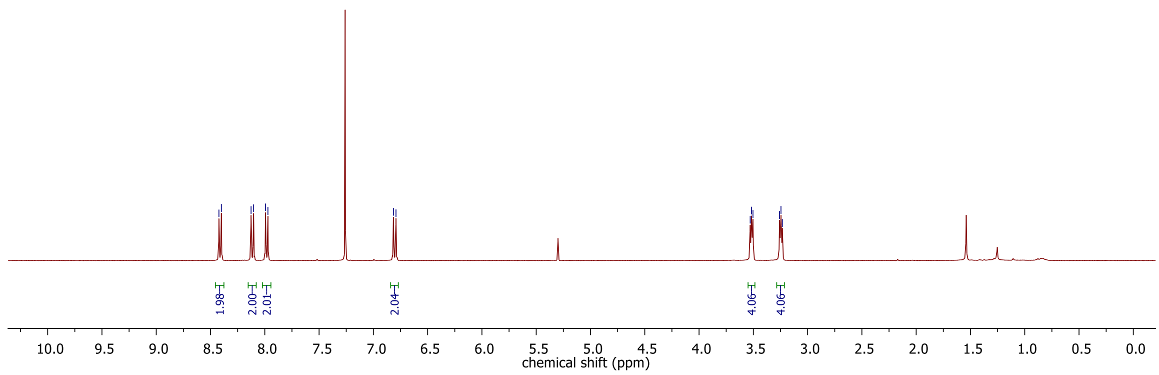

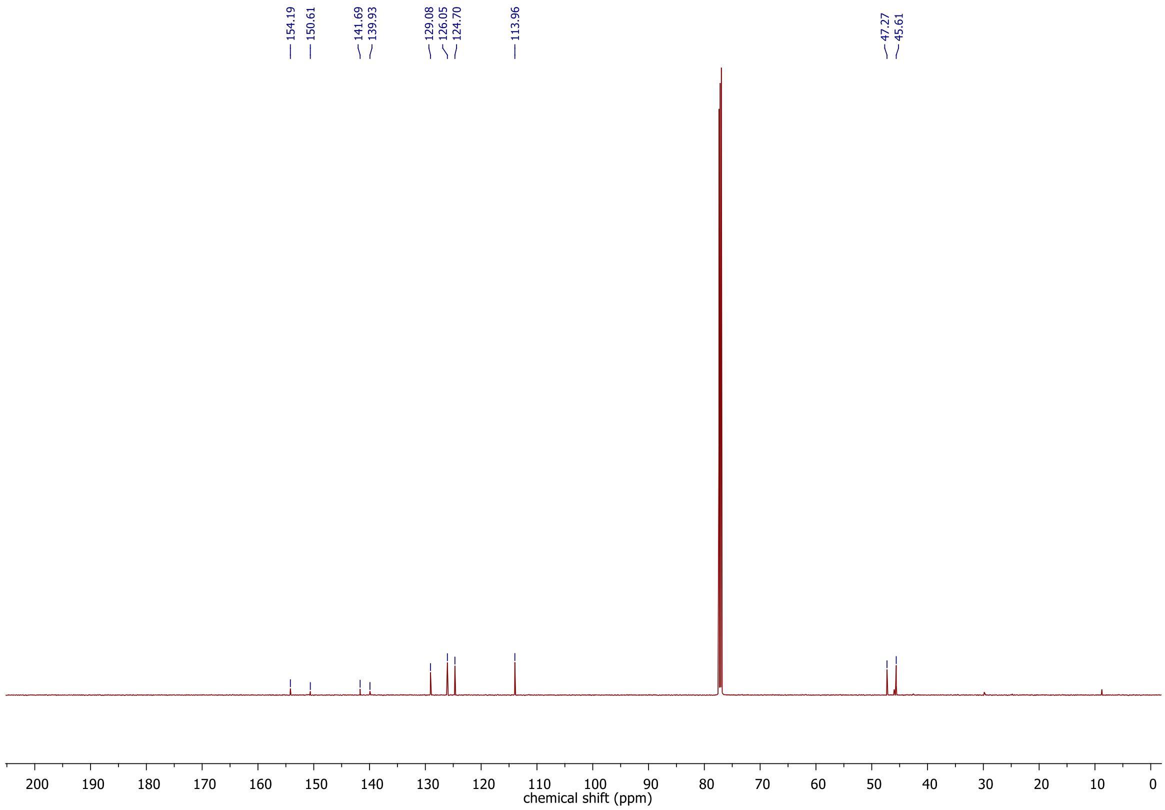

4-((4-(4-Nitrophenyl)piperazin-1-yl)sulfonyl)benzonitrile (30)

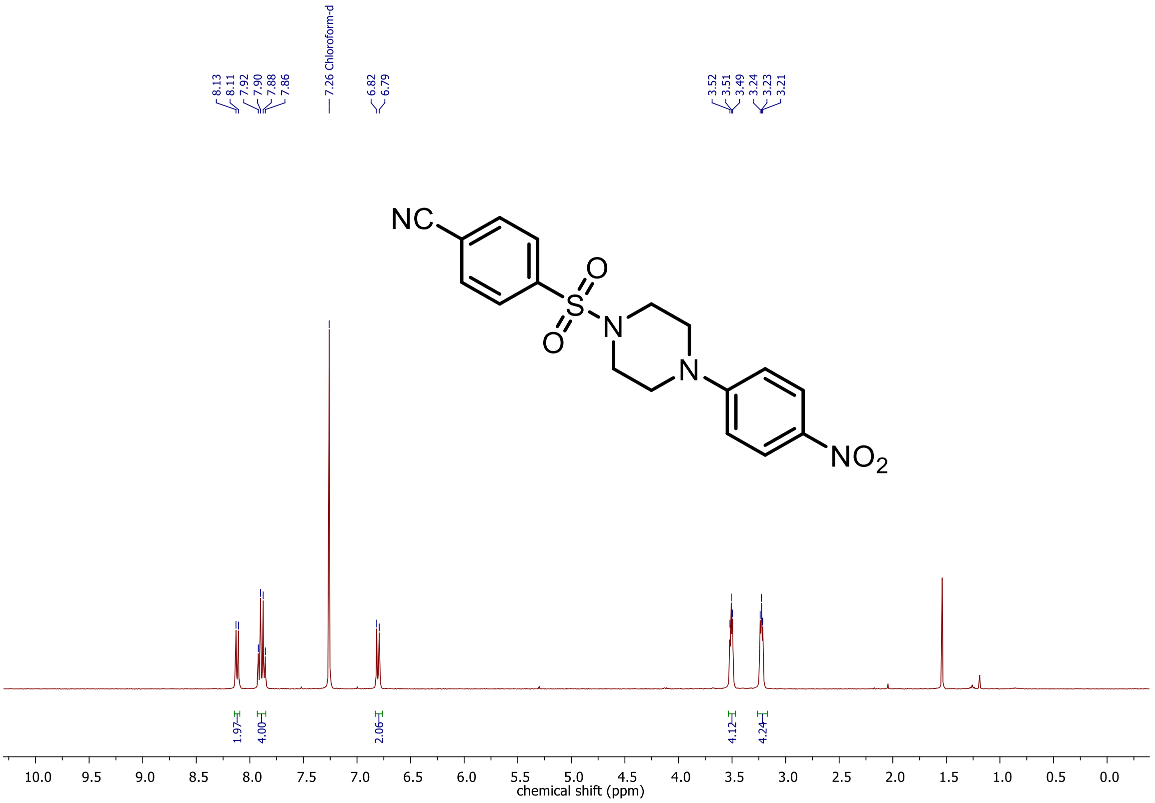

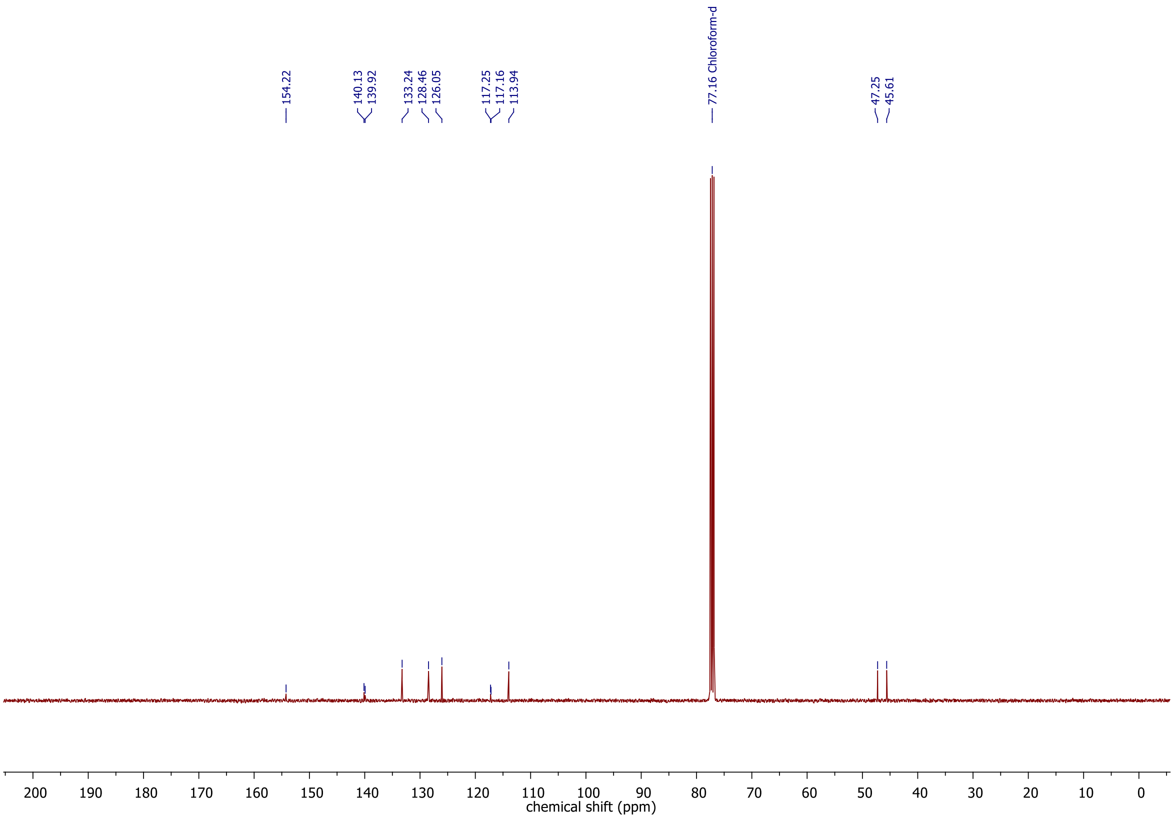

4-(4-((2,4,6-tris(trifluoromethyl)phenyl)sulfonyl)piperazin-1-yl)aniline (31)

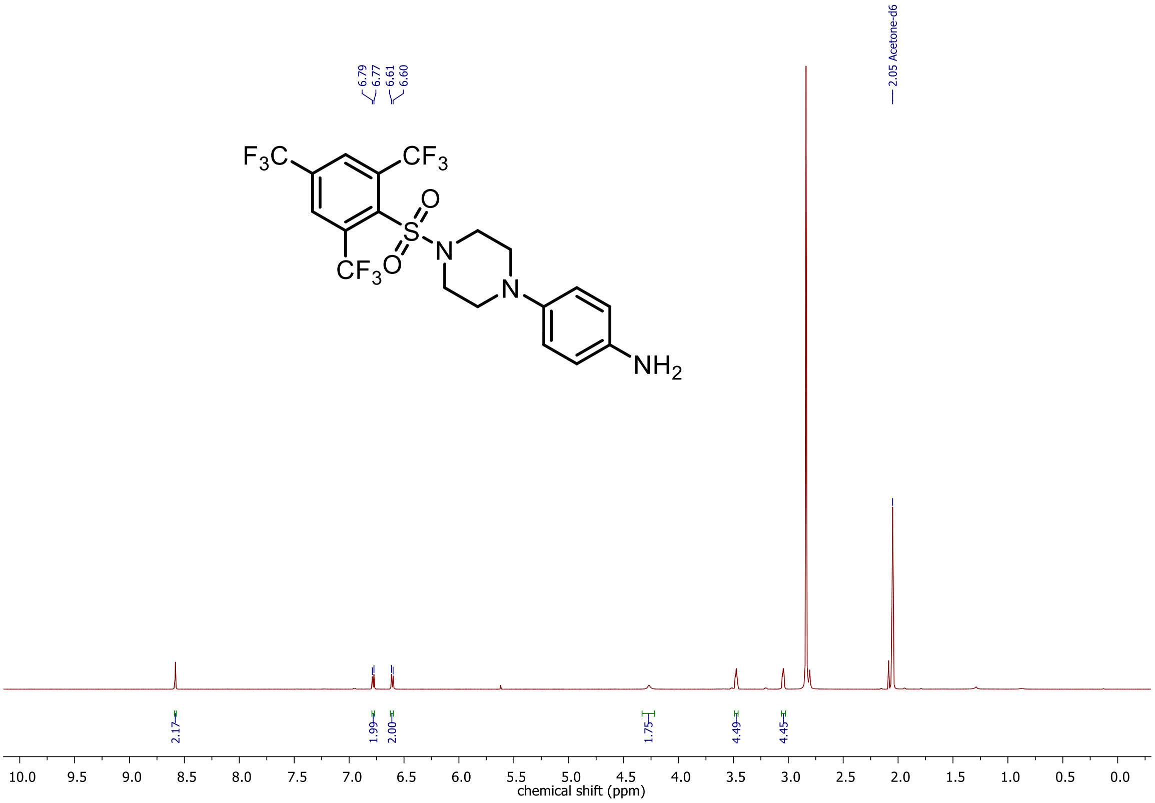

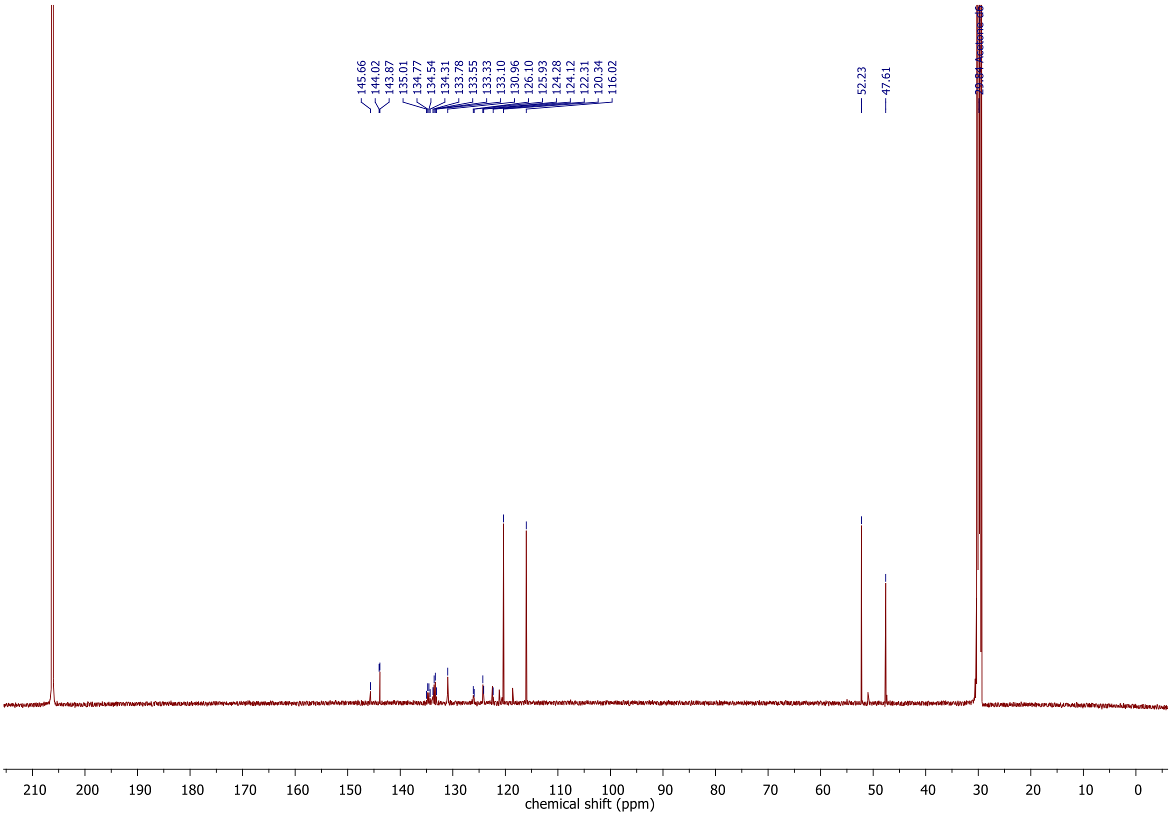

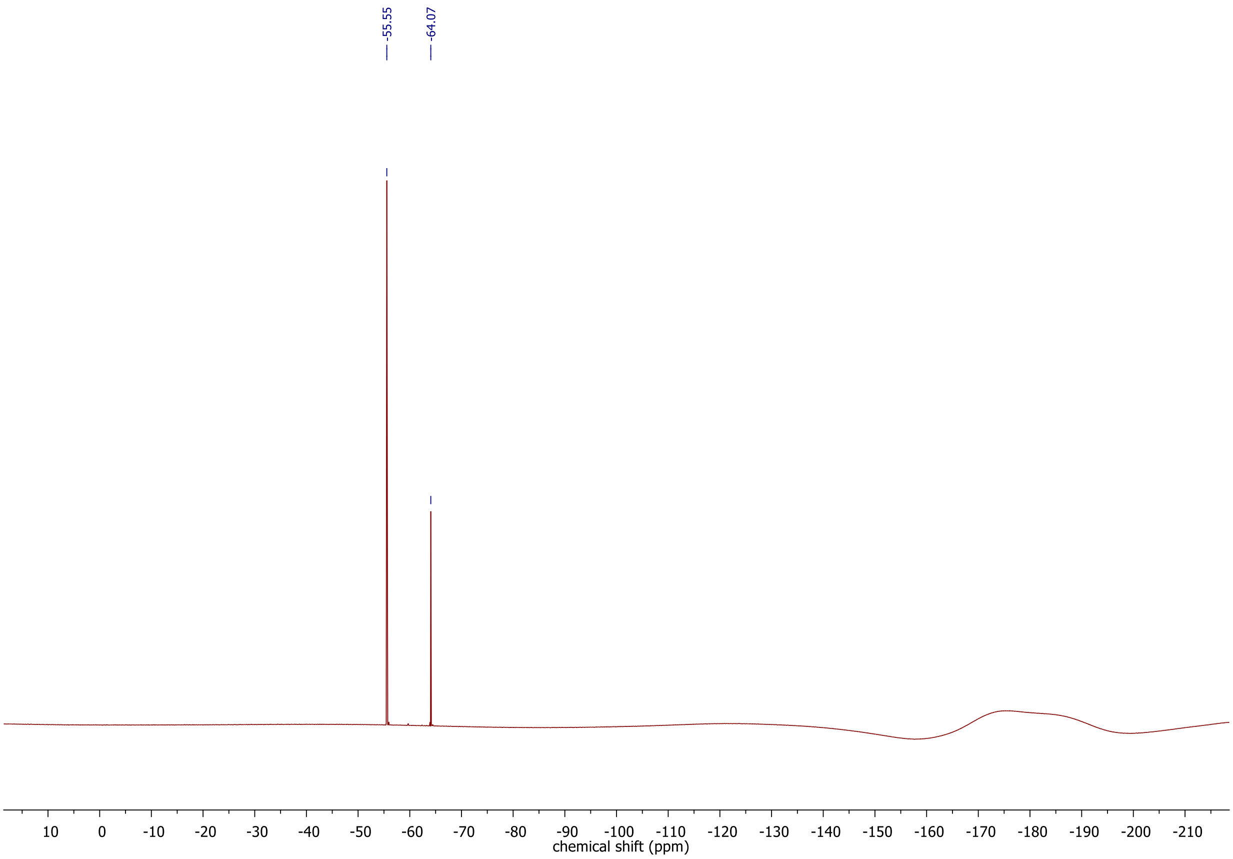
-55.55
-64.07
10
0
-10
-20
-30
-40
-50
-60
-70
-80
-90
-100
-110
-120
-130
-140
-150
-160
-170
-180
-190
-200
-210
chemical shift (ppm)

4-(4-Tosylpiperazin-1-yl)aniline (33’)

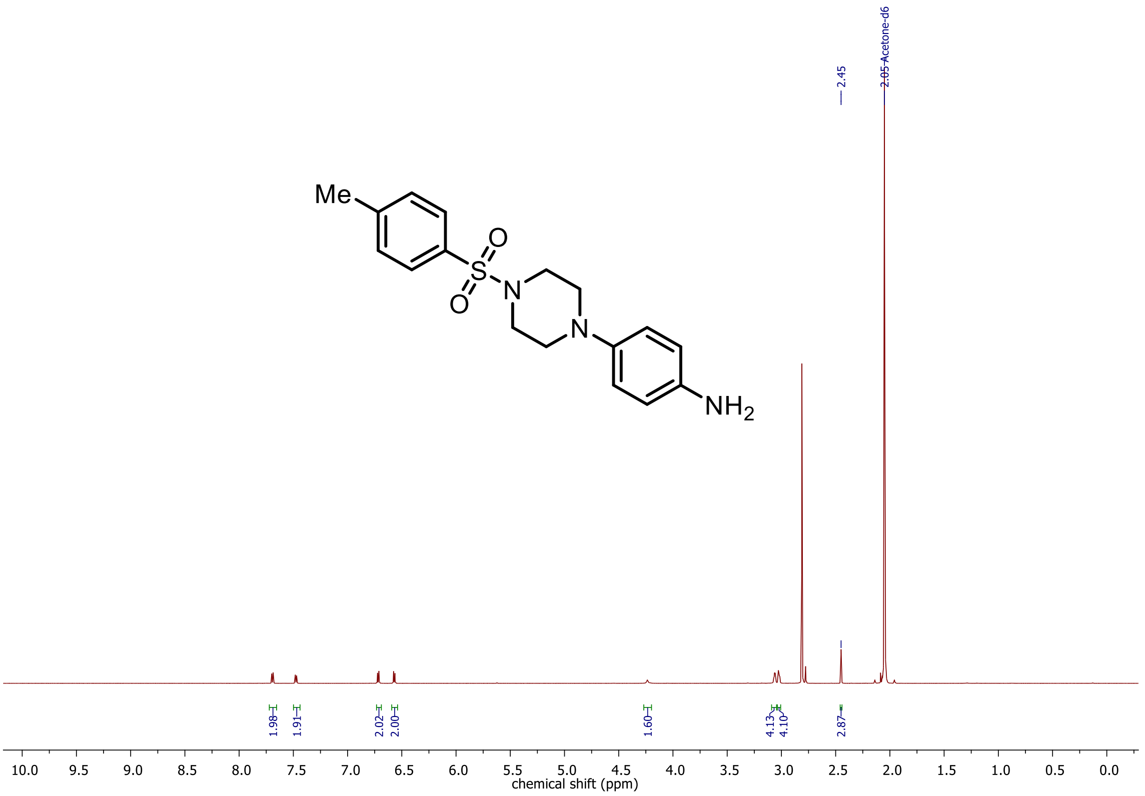

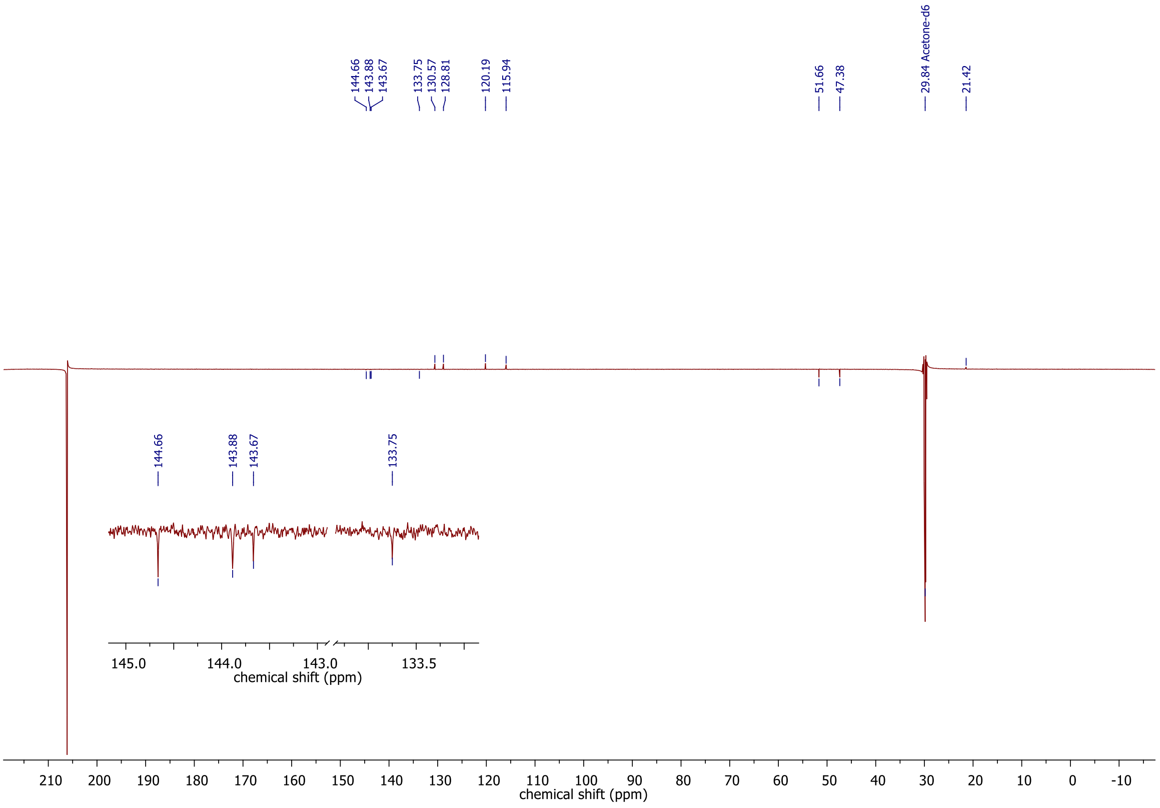

Ethyl 1-((4-nitrophenyl)sulfonyl)piperidine-4-carboxylate (34)

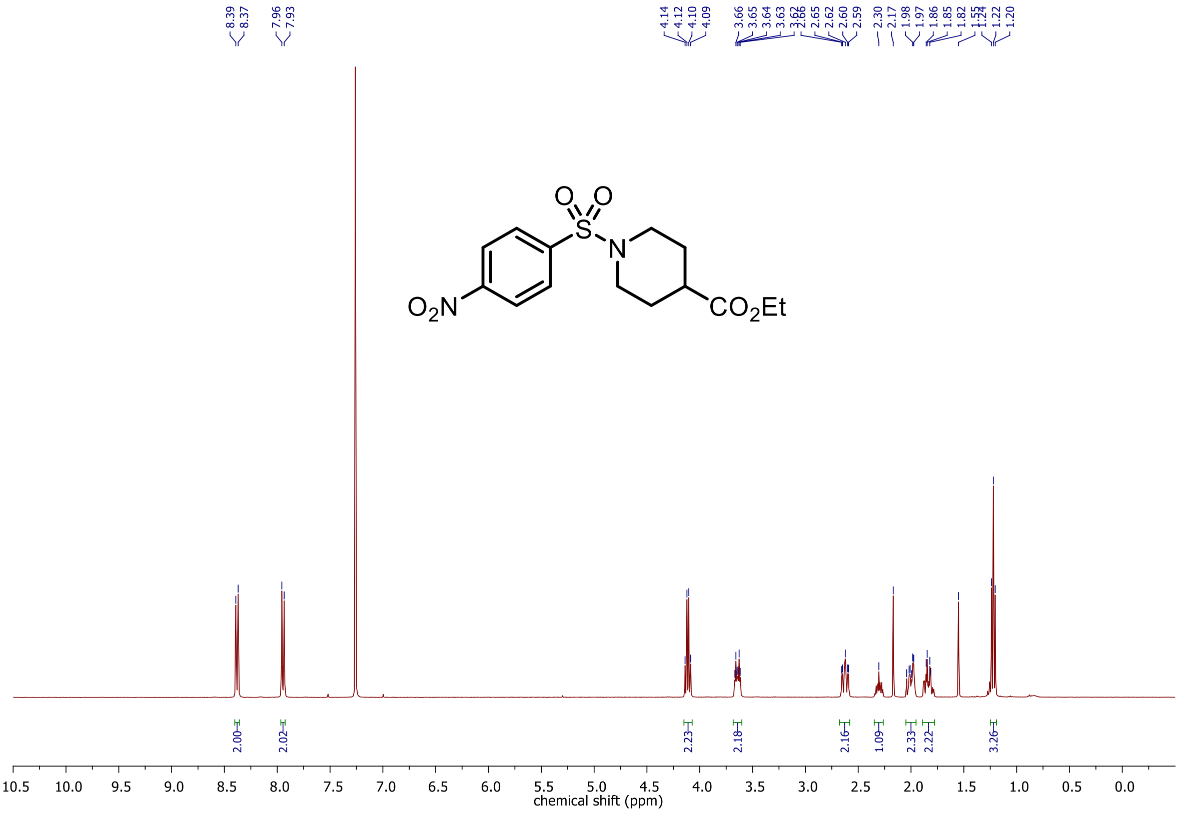

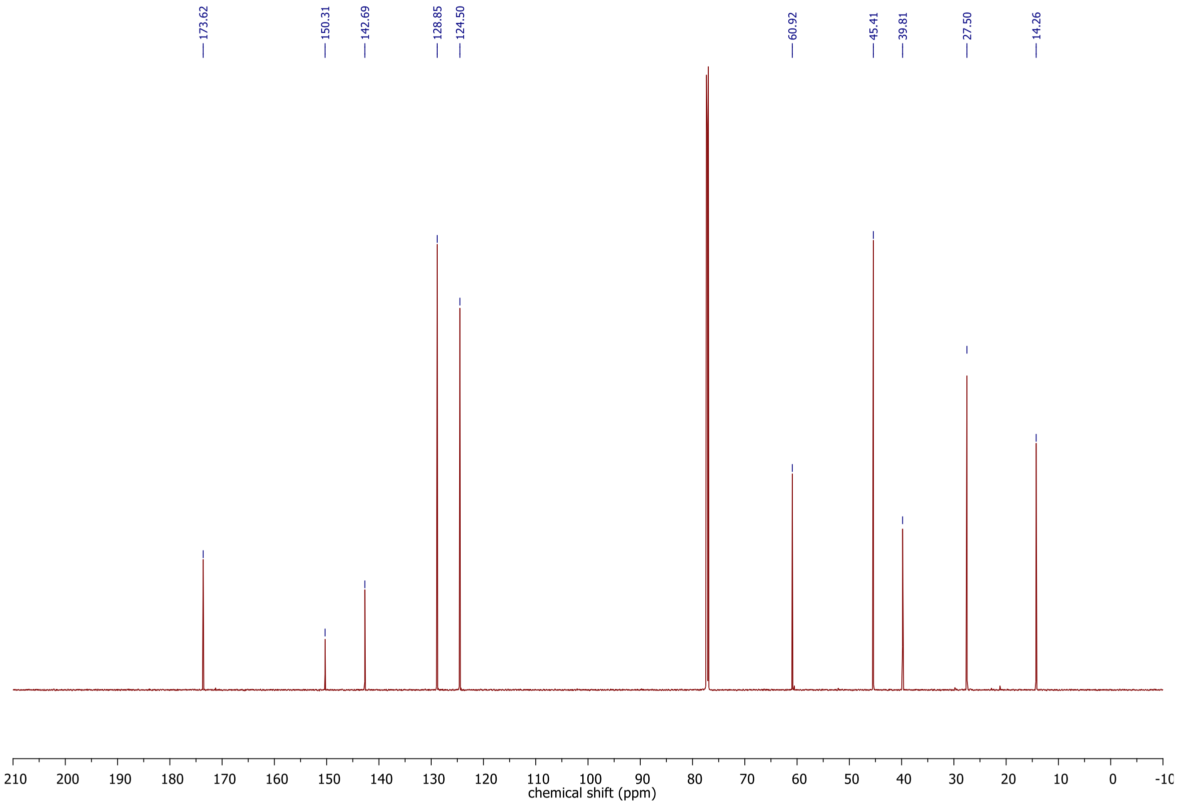

Ethyl 1-((4-cyanophenyl)sulfonyl)piperidine-4-carboxylate (35)

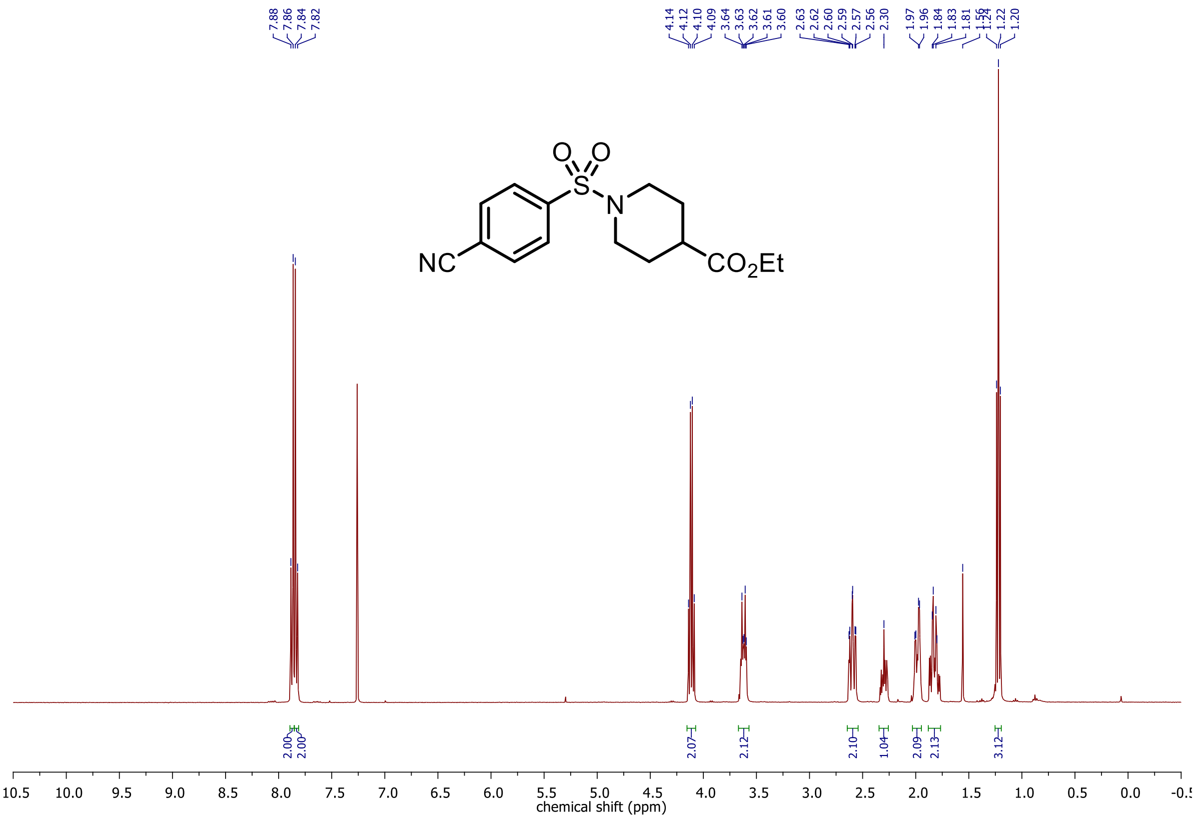

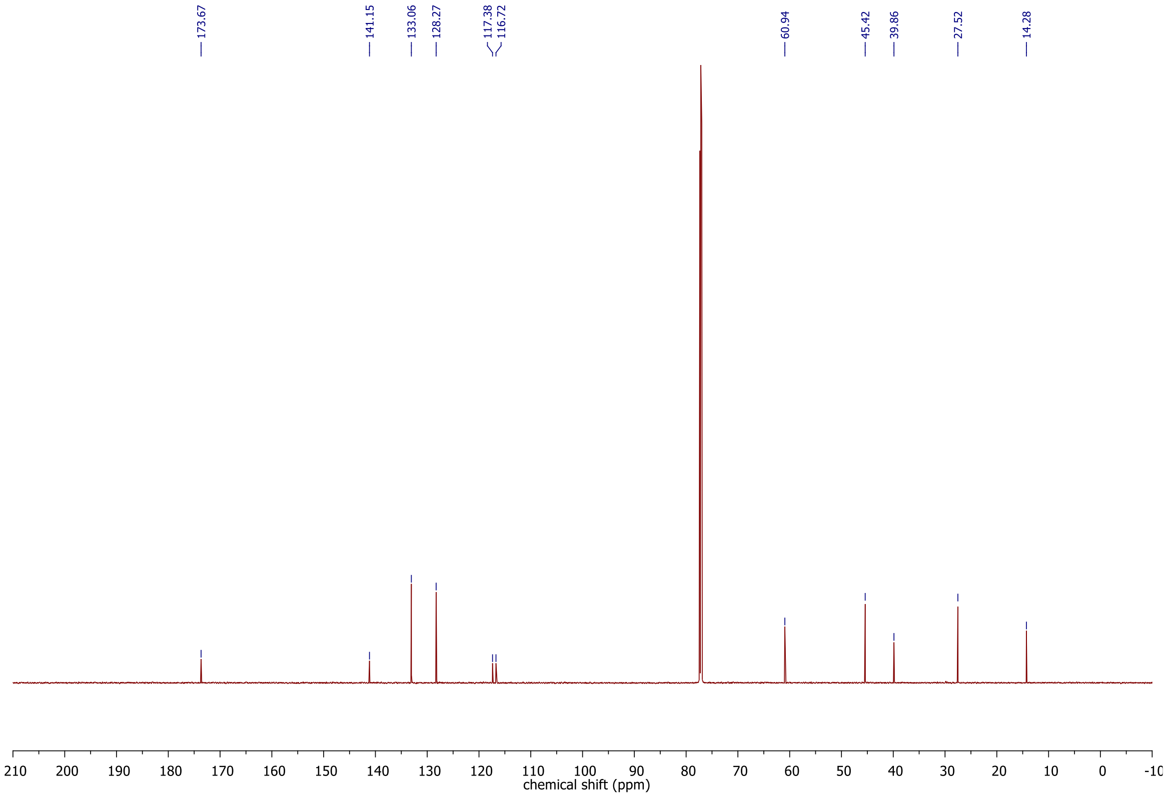

Ethyl 1-tosylpiperidine-4-carboxylate (36)

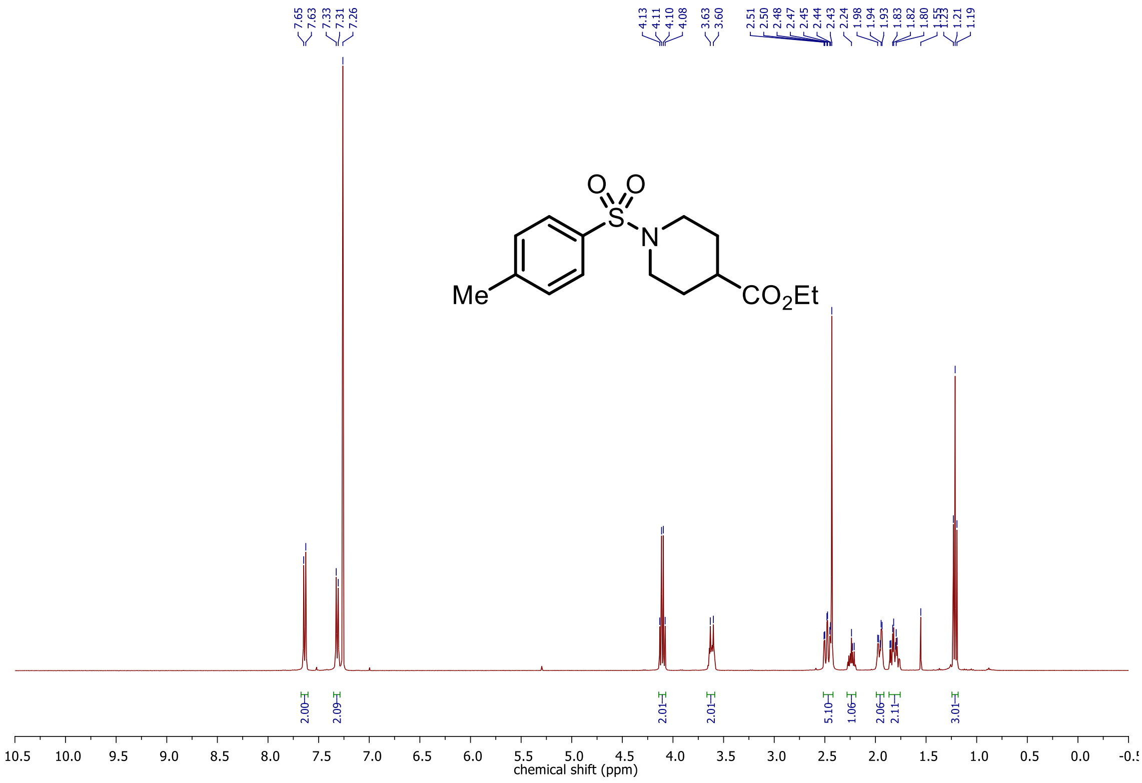

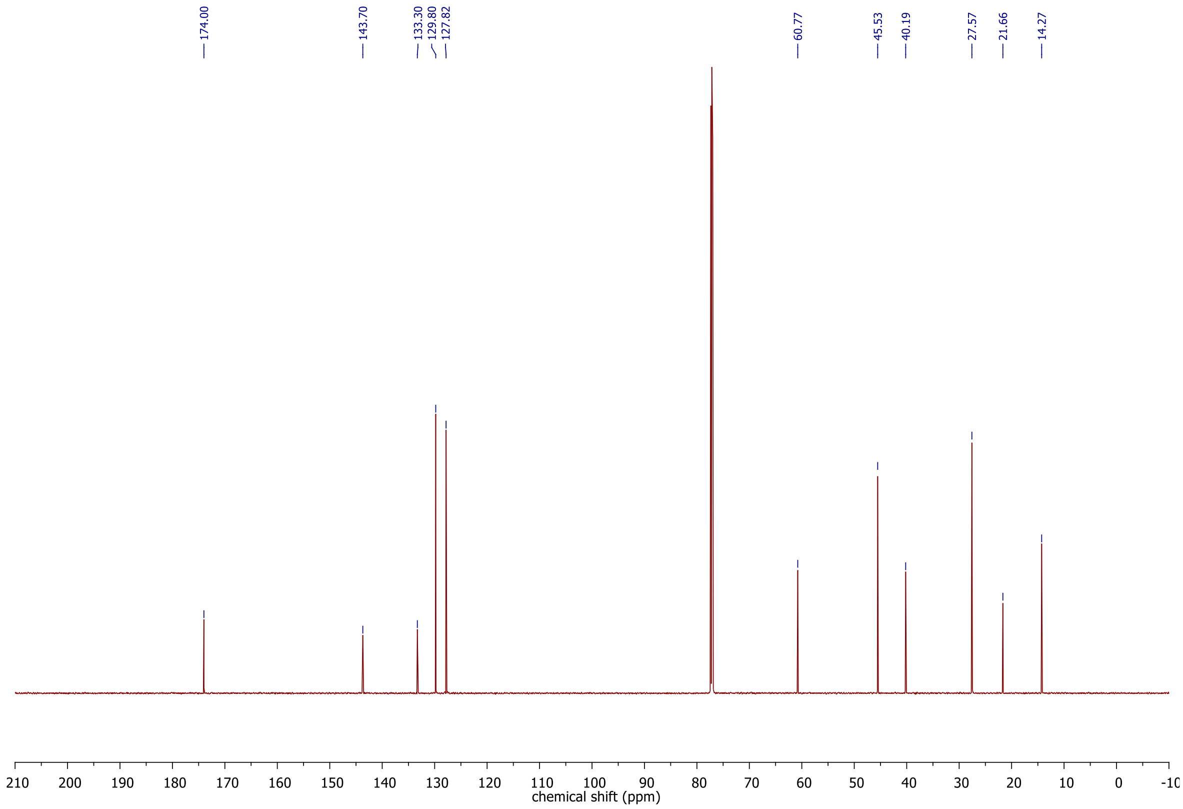

[1-((2,4,6-tris(Trifluoromethyl)phenyl)sulfonyl)piperidin-4-yl]methanol (37)

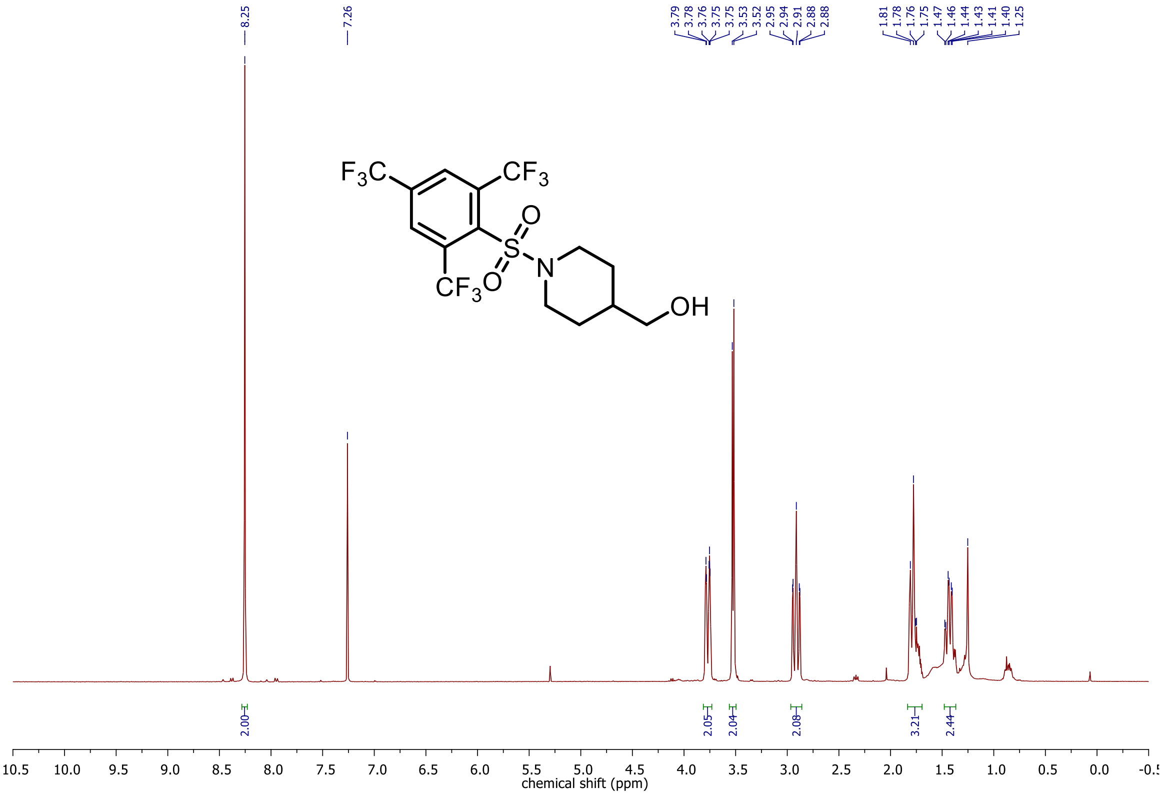

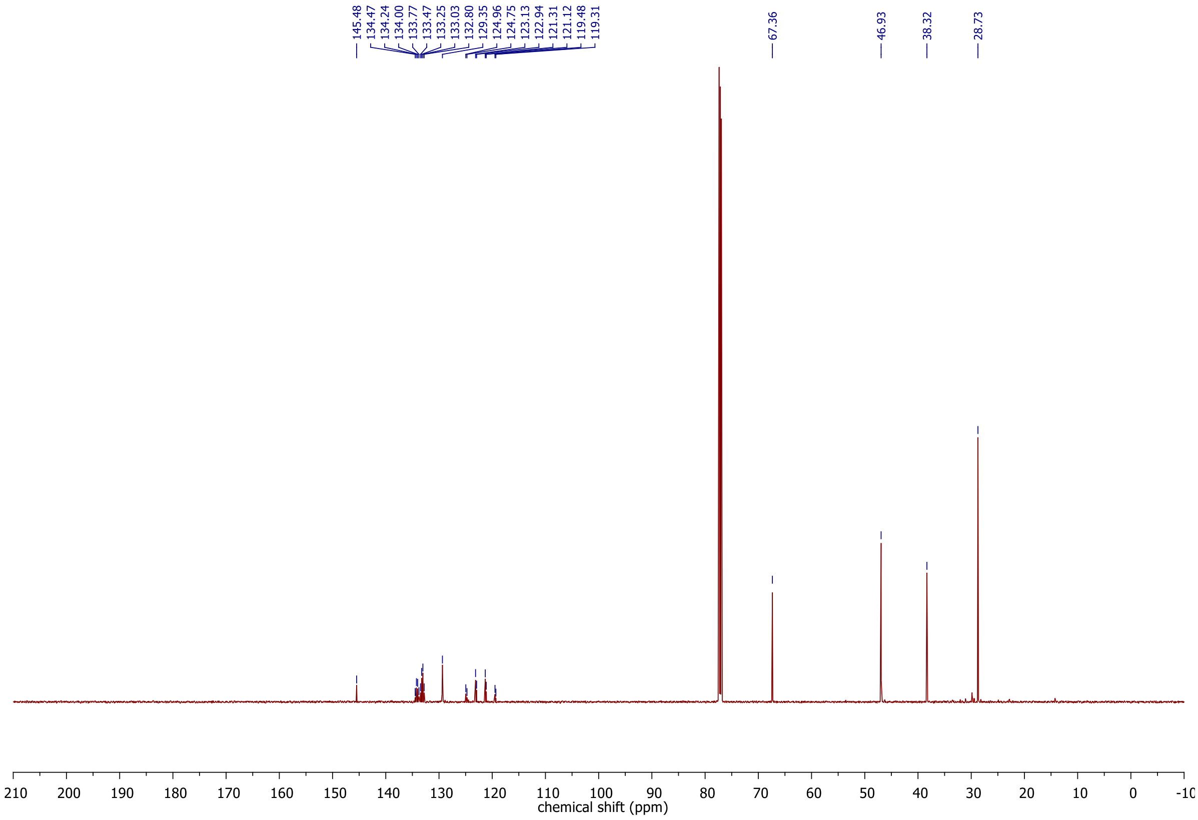

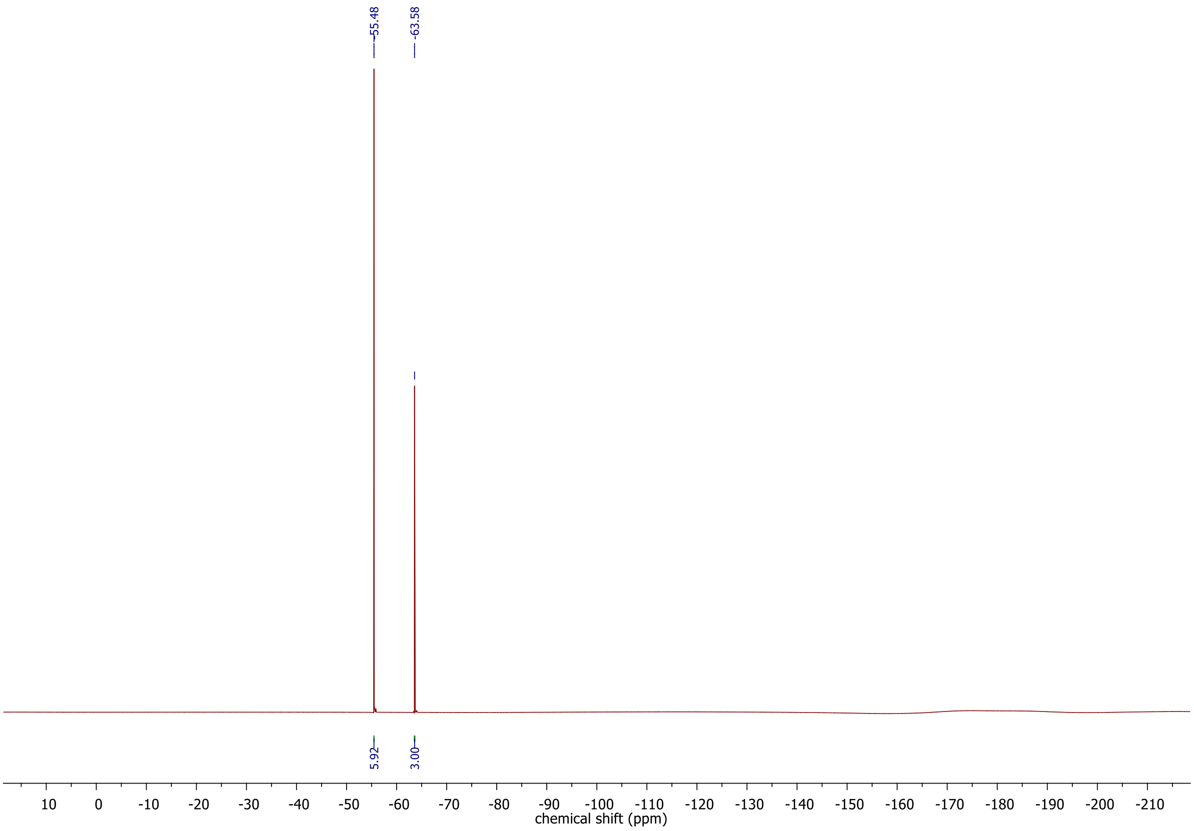

-55.48
-63.58
5.92
3.00
10
0
-10
-20
-30
-40
-50
-60
-70
-80
-90
-100
-110
-120
-130
-140
-150
-160
-170
-180
-190
-200
-210
chemical shift (ppm)

[1-((4-Nitrophenyl)sulfonyl)piperidin-4-yl]methanol (38)

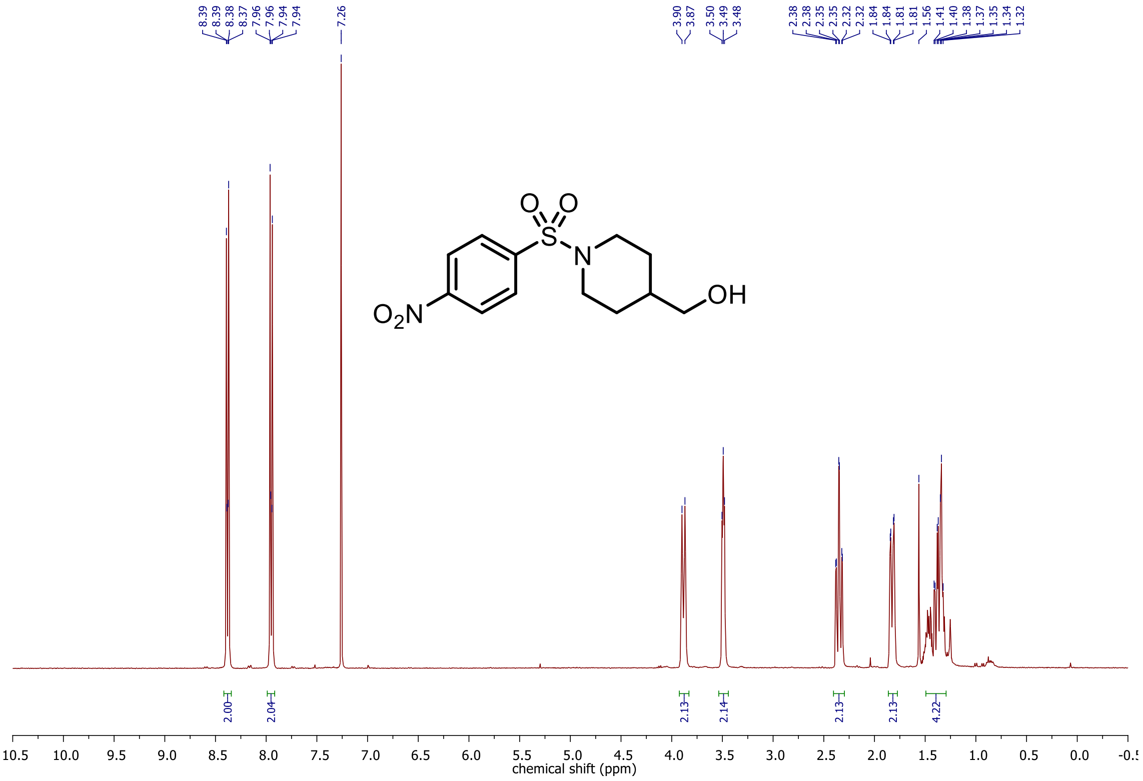

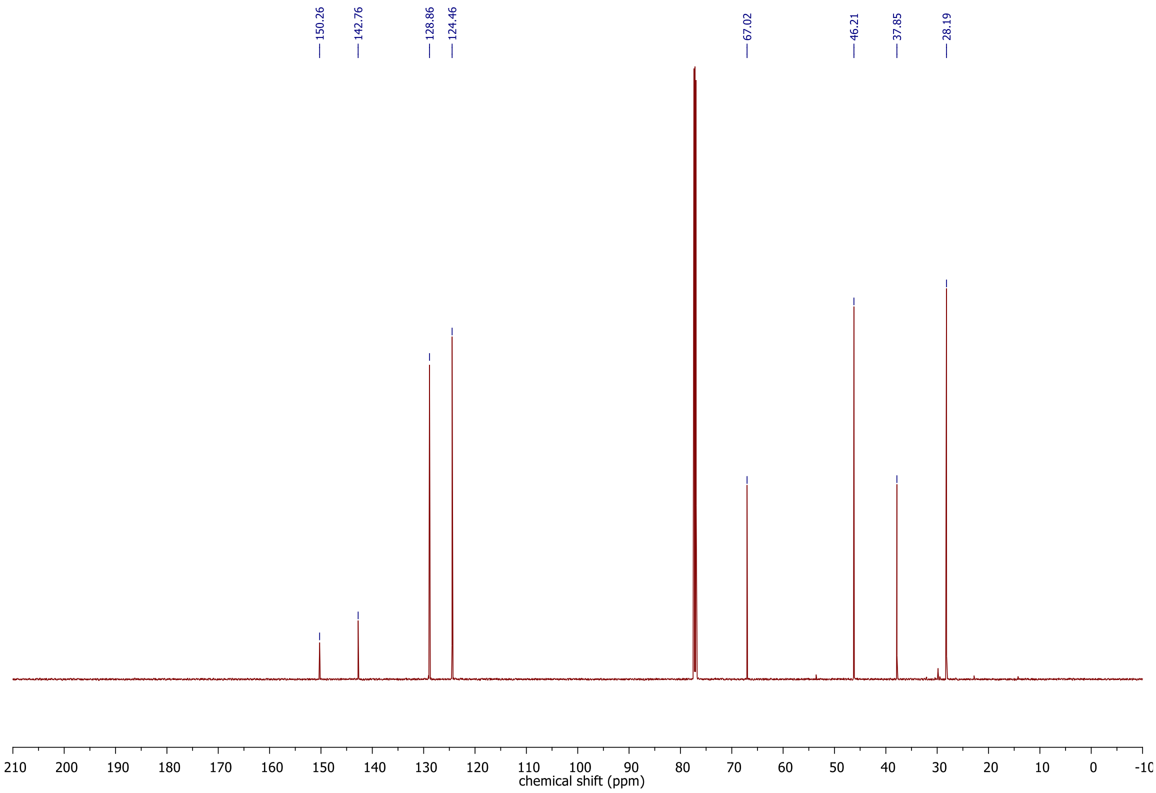

4-[(4-(Hydroxymethyl)piperidin-1-yl)sulfonyl]benzaldehyde (39’)

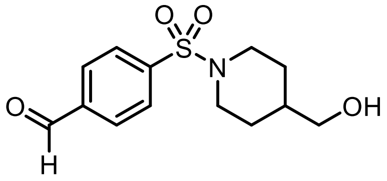

with impurities

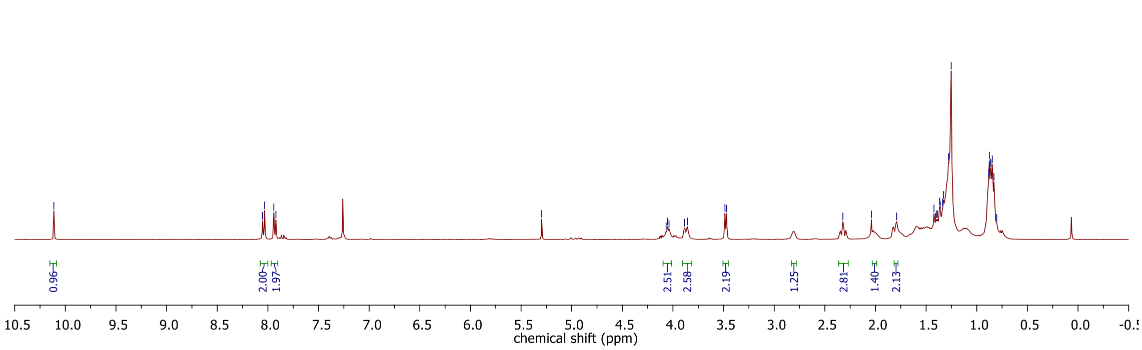

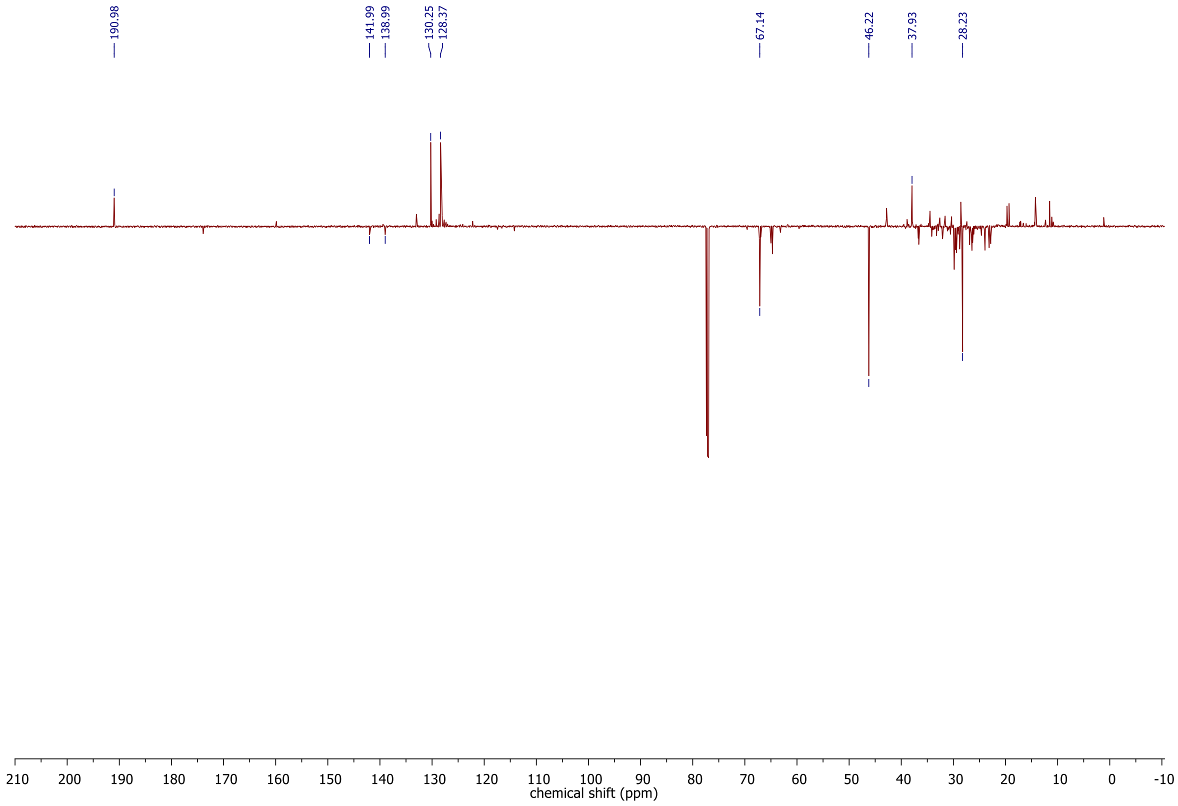

[1-((4-Methylphenyl)sulfonyl)piperidin-4-yl]methanol (40)

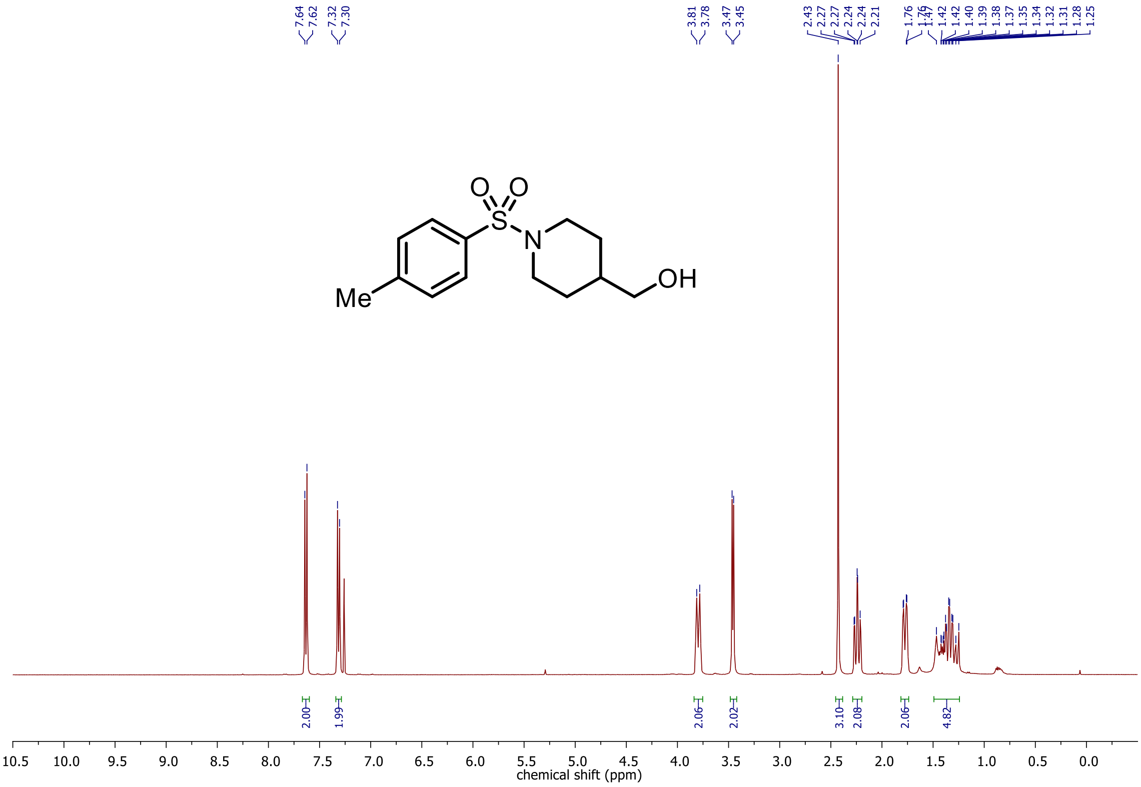

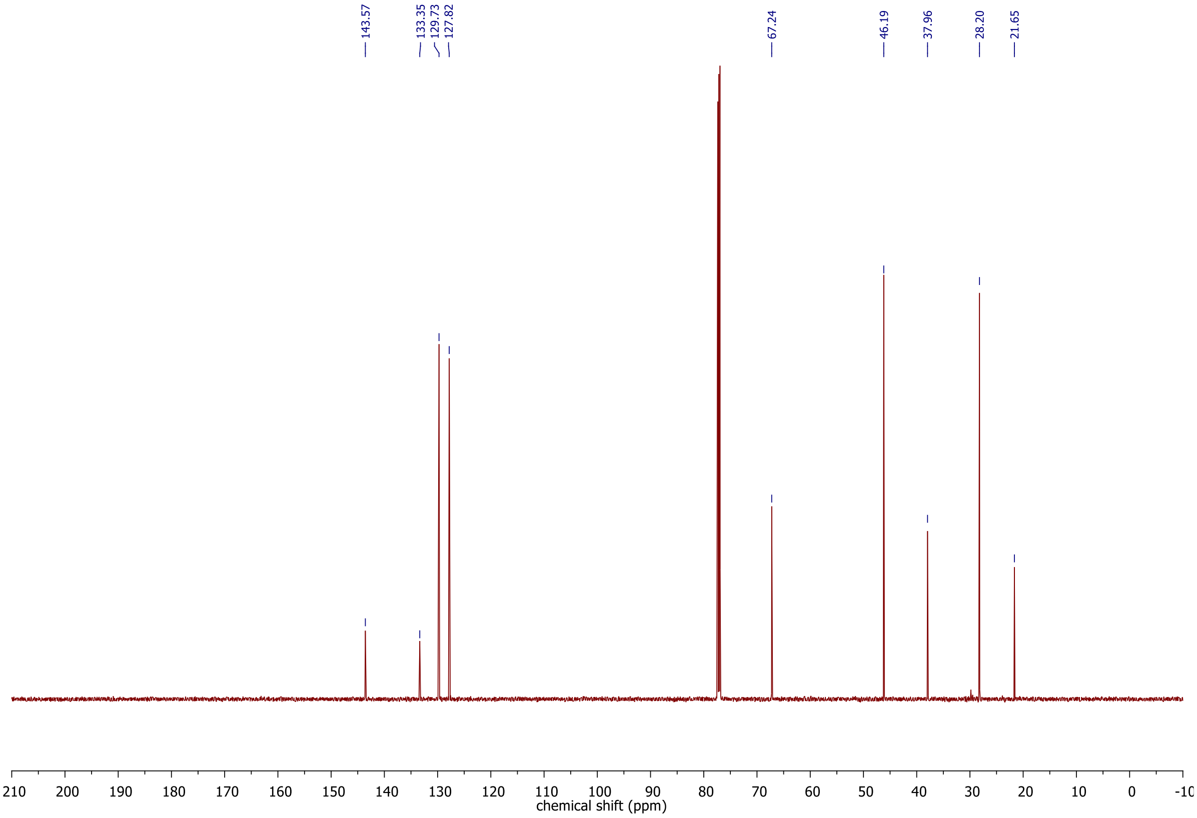

1-((2,4,6-Tris(trifluoromethyl)phenyl)sulfonyl)-4-vinylpiperidine (41)

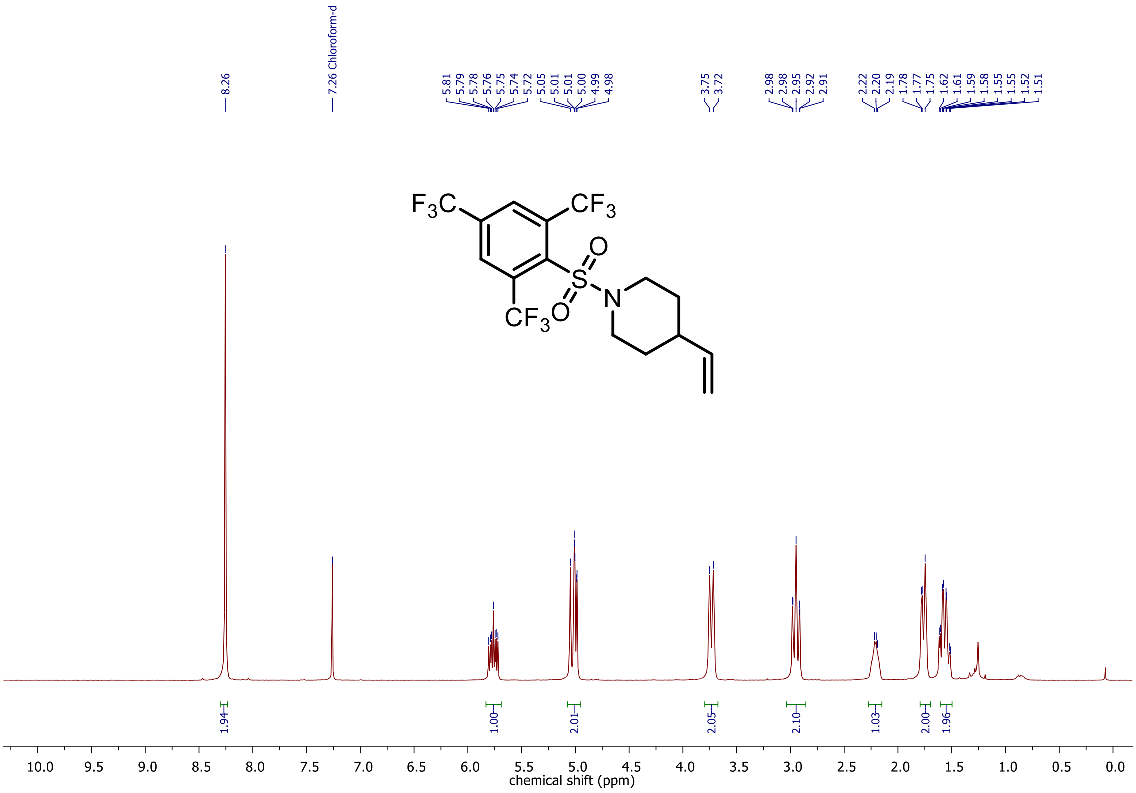

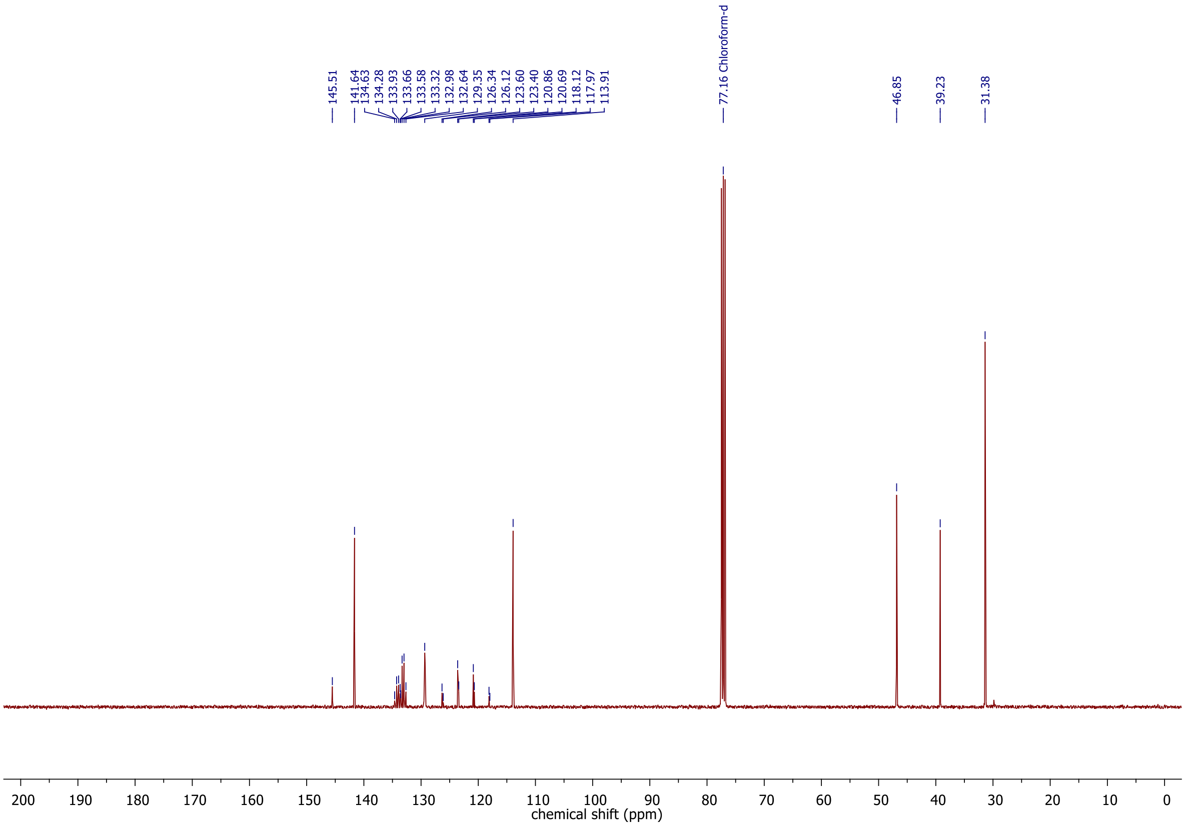

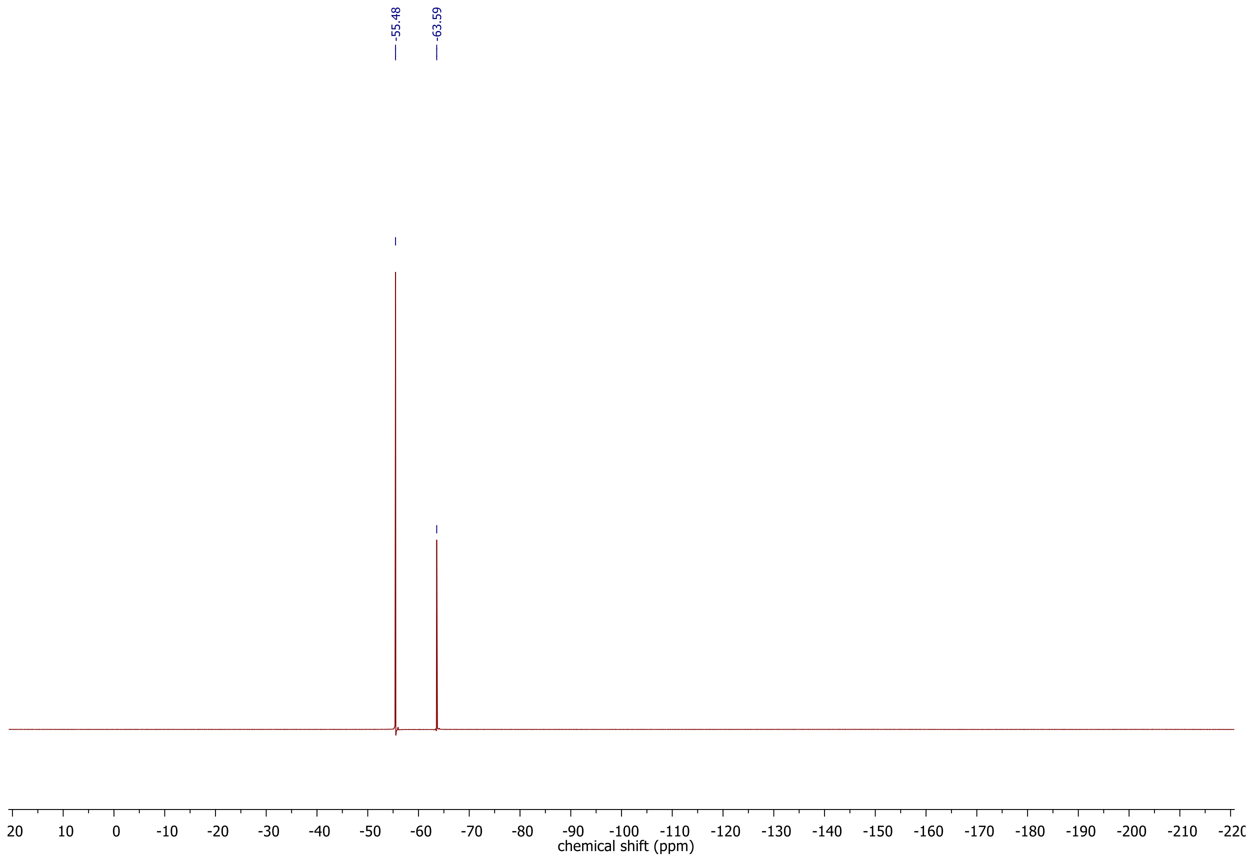
-55.48
-63.59
20 10 0 -10 -20 -30 -40 -50 -60 -70 -80 -90 -100 -110 -120 -130 -140 -150 -160 -170 -180 -190 -200 -210 -220
chemical shift (ppm)

4-Ethyl-1-((2,4,6-tris(trifluoromethyl)phenyl)sulfonyl)piperidine (42)

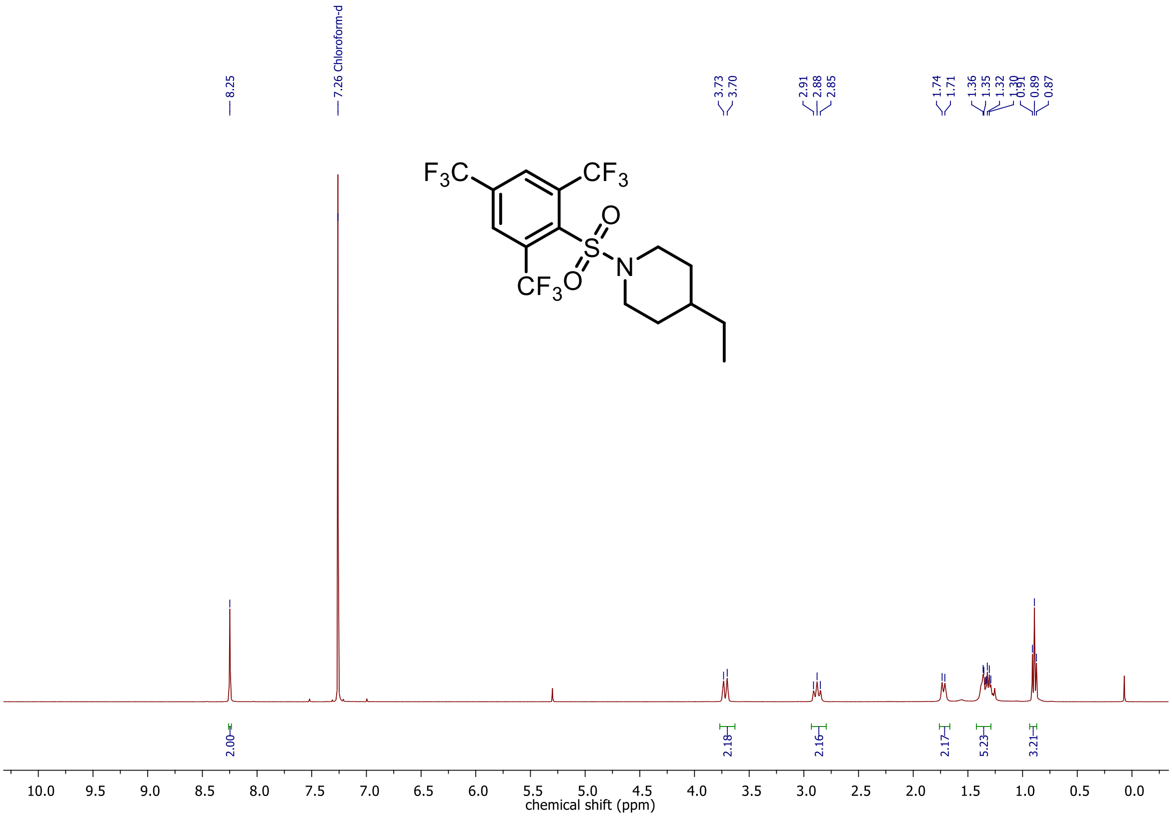

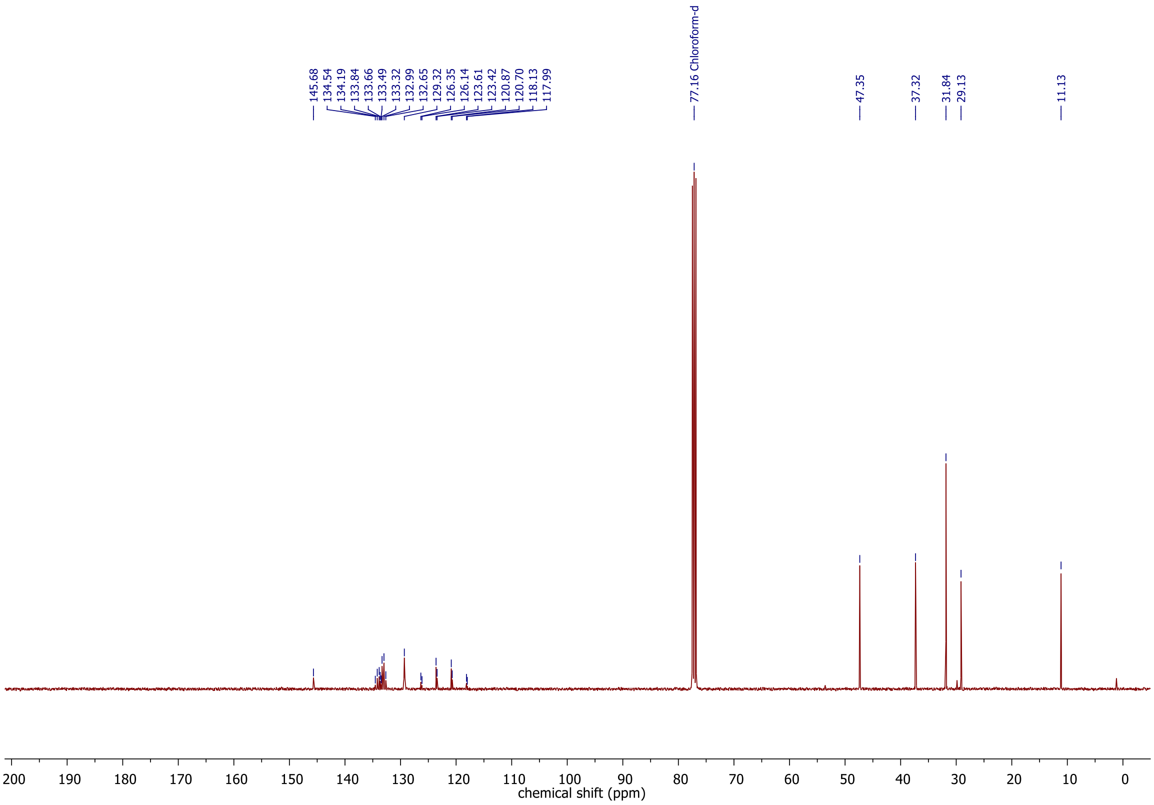

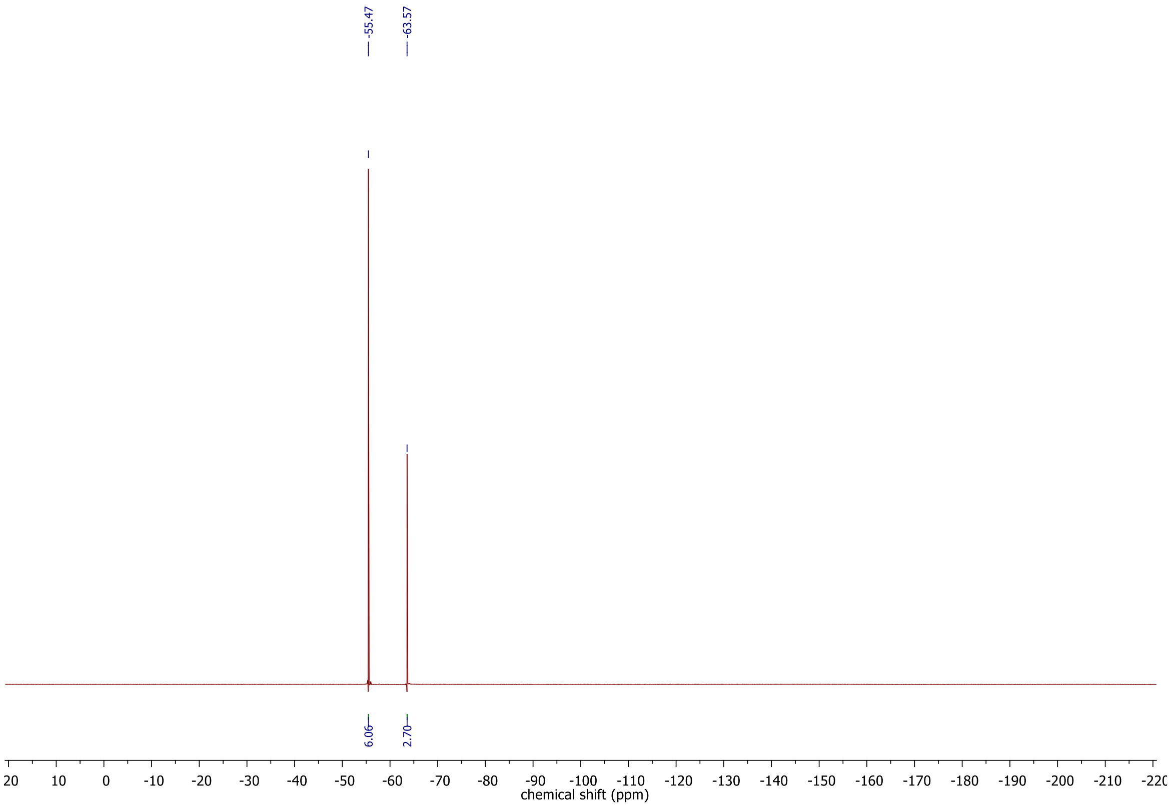

-55.47
-63.57
6.06
2.70
20 10 0 -10 -20 -30 -40 -50 -60 -70 -80 -90 -100 -110 -120 -130 -140 -150 -160 -170 -180 -190 -200 -210 -220
chemical shift (ppm)

***N*-[2-(1*H*-Indol-3-yl)ethyl]-*N*-benzyl-2,4,6-tris(trifluoromethyl)benzenesulfonamide (43)**

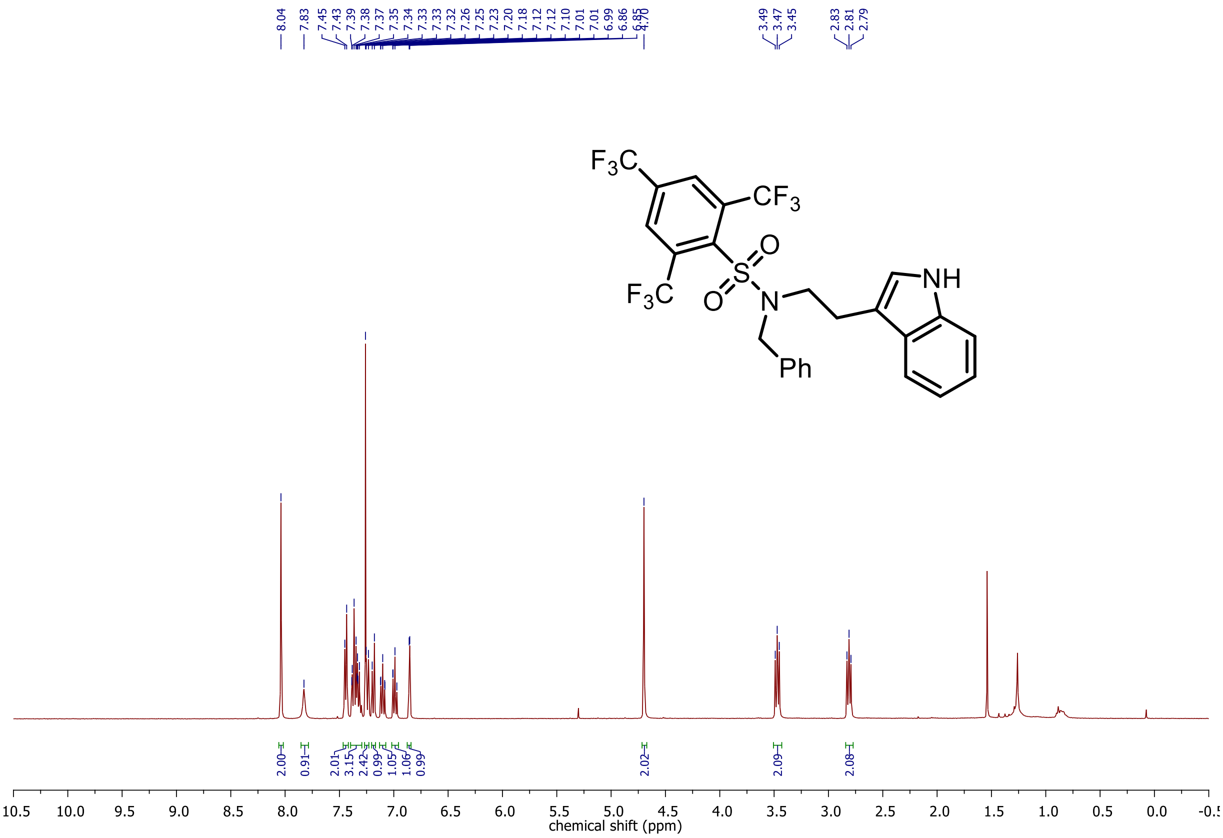

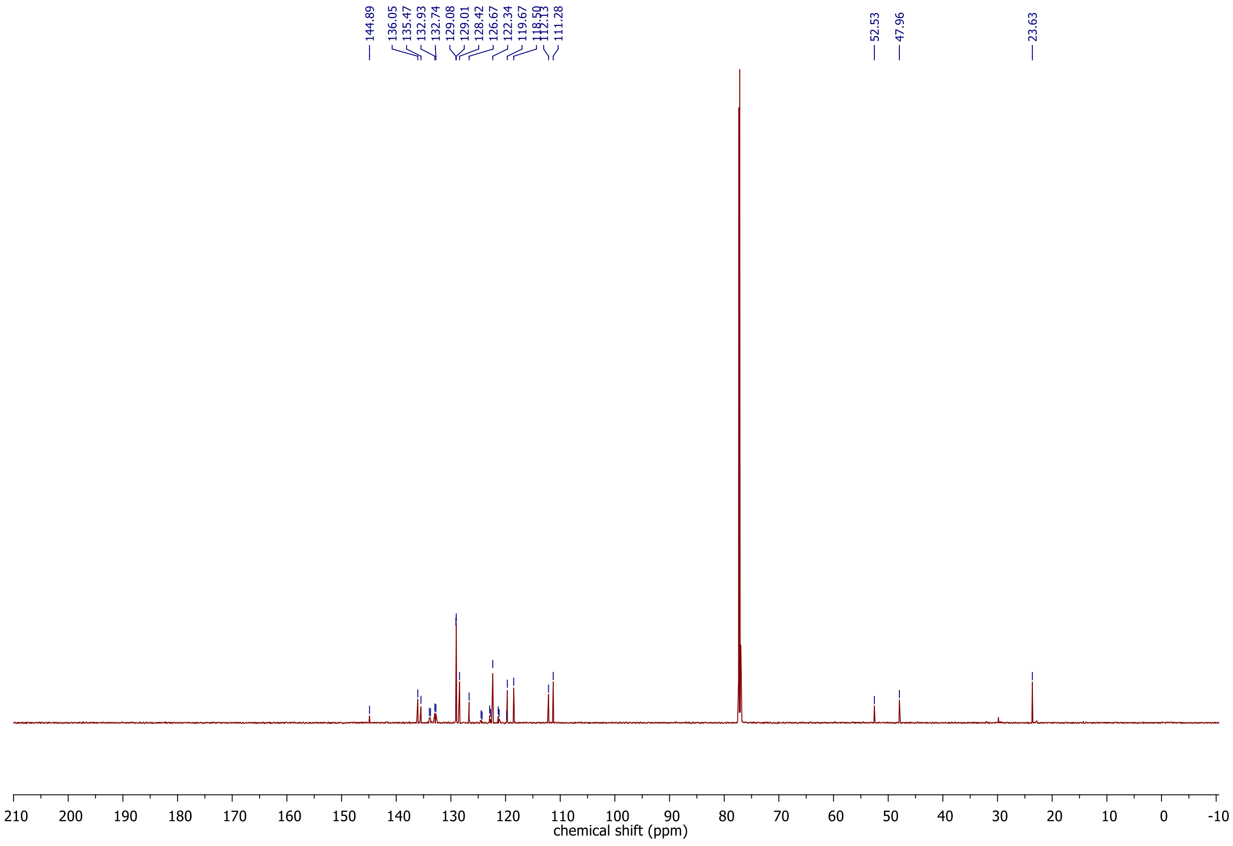

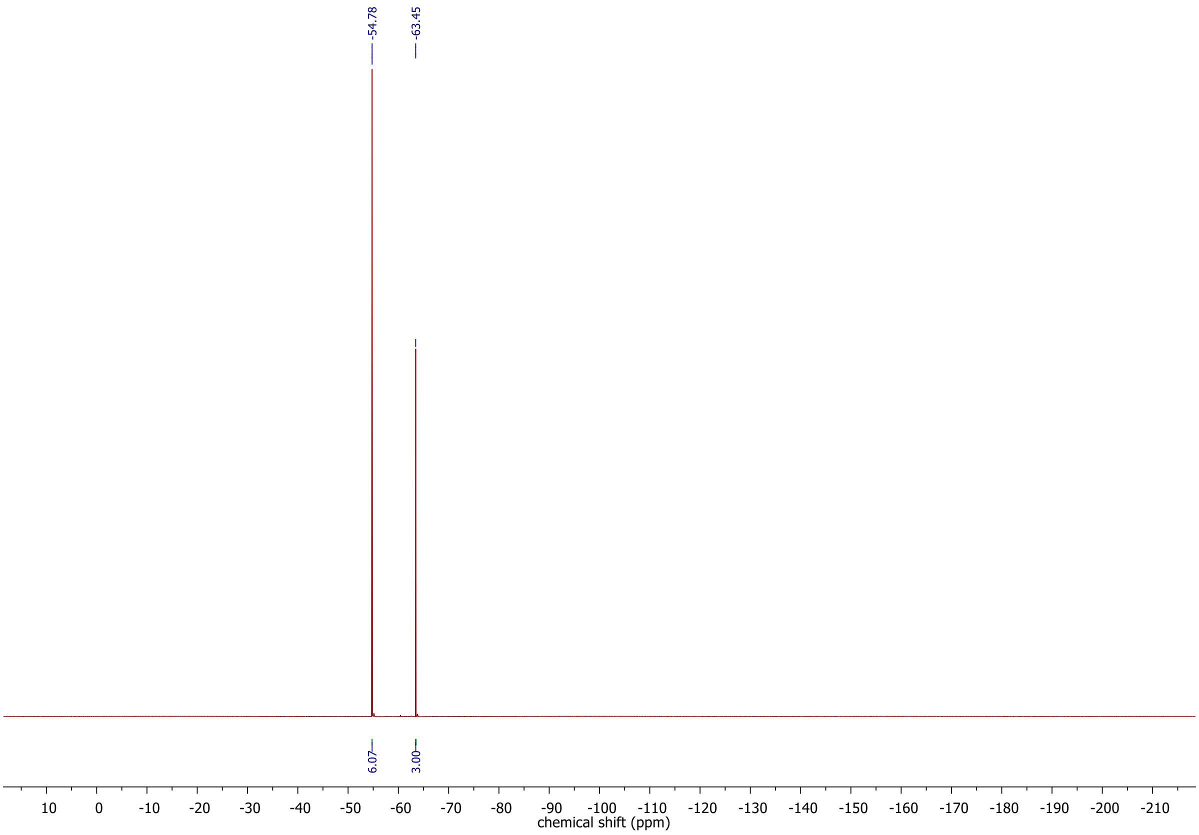
-54.78
-63.45
6.07
3.00
10 0 -10 -20 -30 -40 -50 -60 -70 -80 -90 -100 -110 -120 -130 -140 -150 -160 -170 -180 -190 -200 -210
chemical shift (ppm)

N-[2-(1*H*-Indol-3-yl)ethyl]-*N*-allyl-2,4,6-tris(trifluoromethyl)benzenesulfonamide (44)

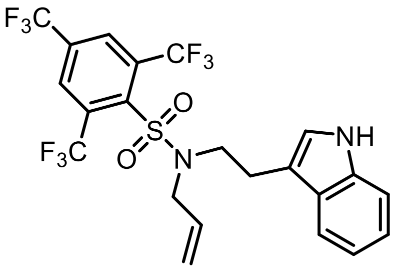

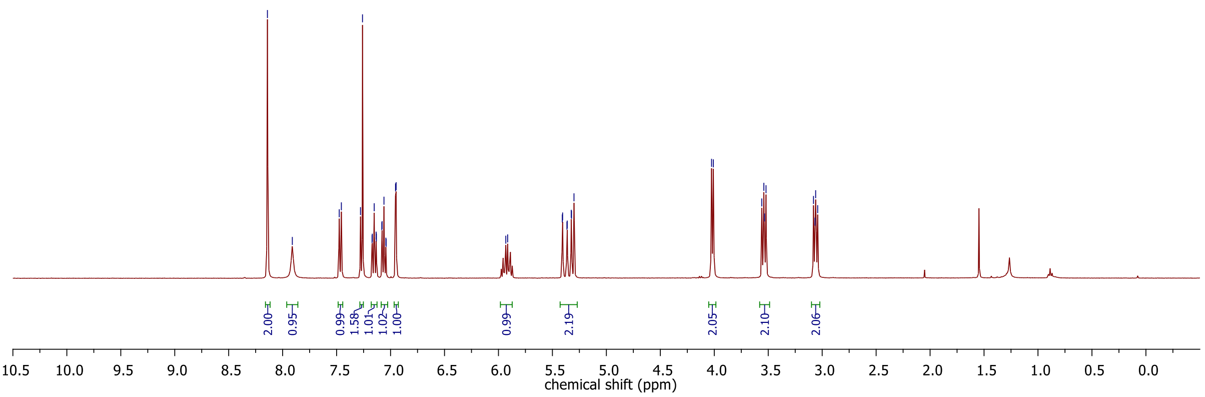

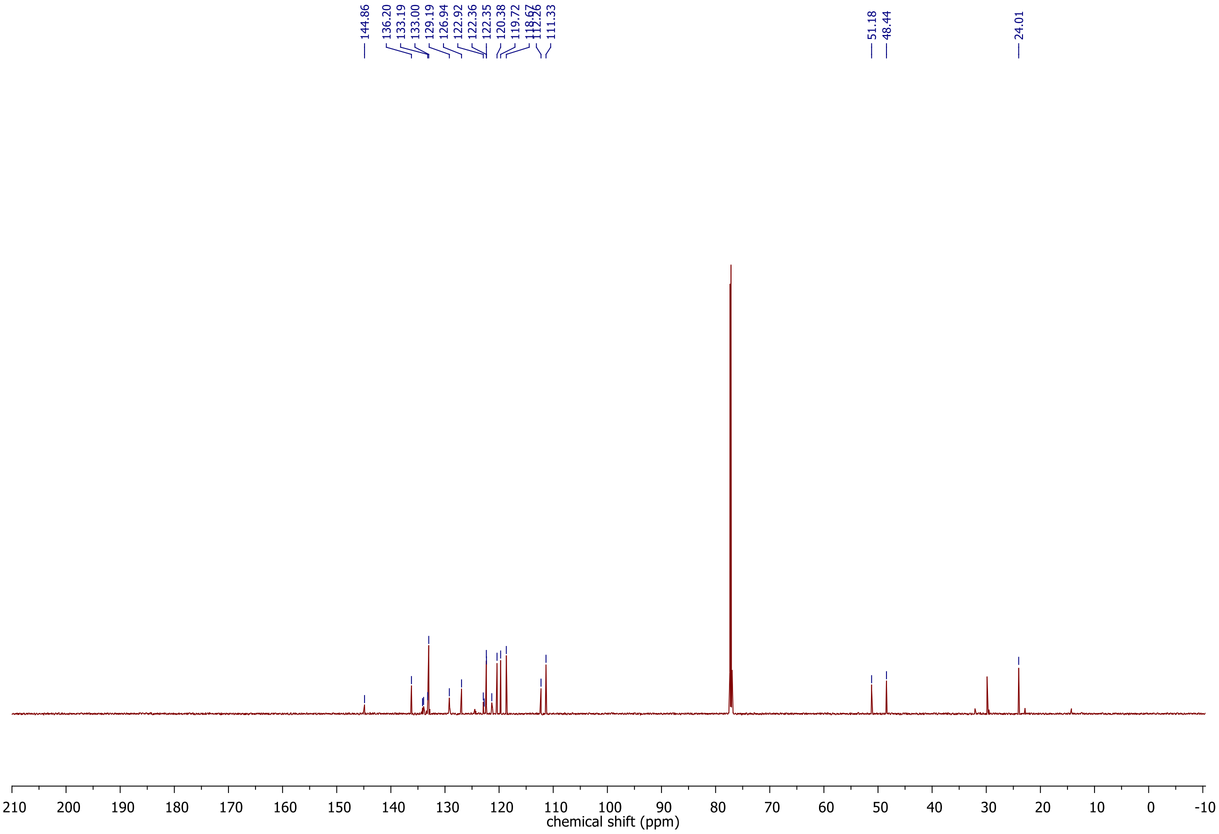

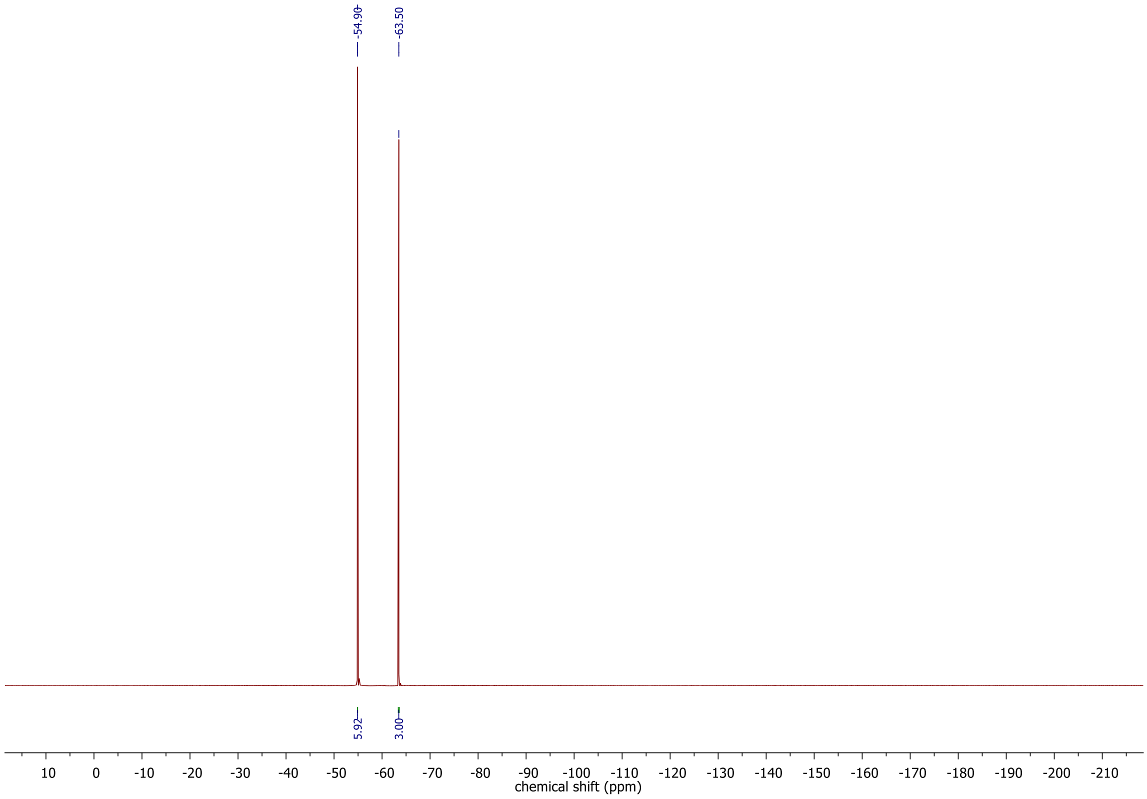
-54.90
-63.50
5.92
3.00
10
0
-10
-20
-30
-40
-50
-60
-70
-80
-90
-100
-110
-120
-130
-140
-150
-160
-170
-180
-190
-200
-210
chemical shift (ppm)

***N*-[2-(1*H*-Indol-3-yl)ethyl]-*N*-(cyanomethyl)-2,4,6-tris(trifluoromethyl)benzenesulfonamide (45)**

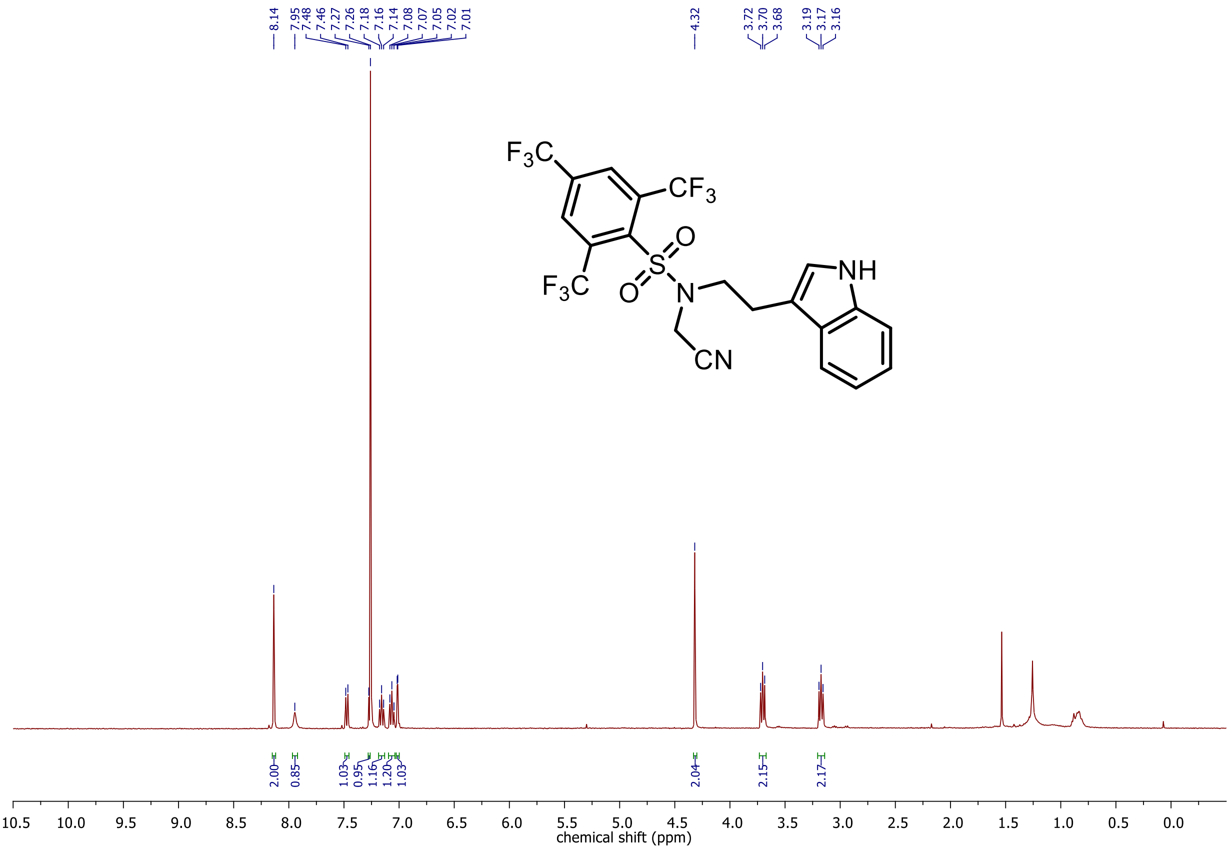

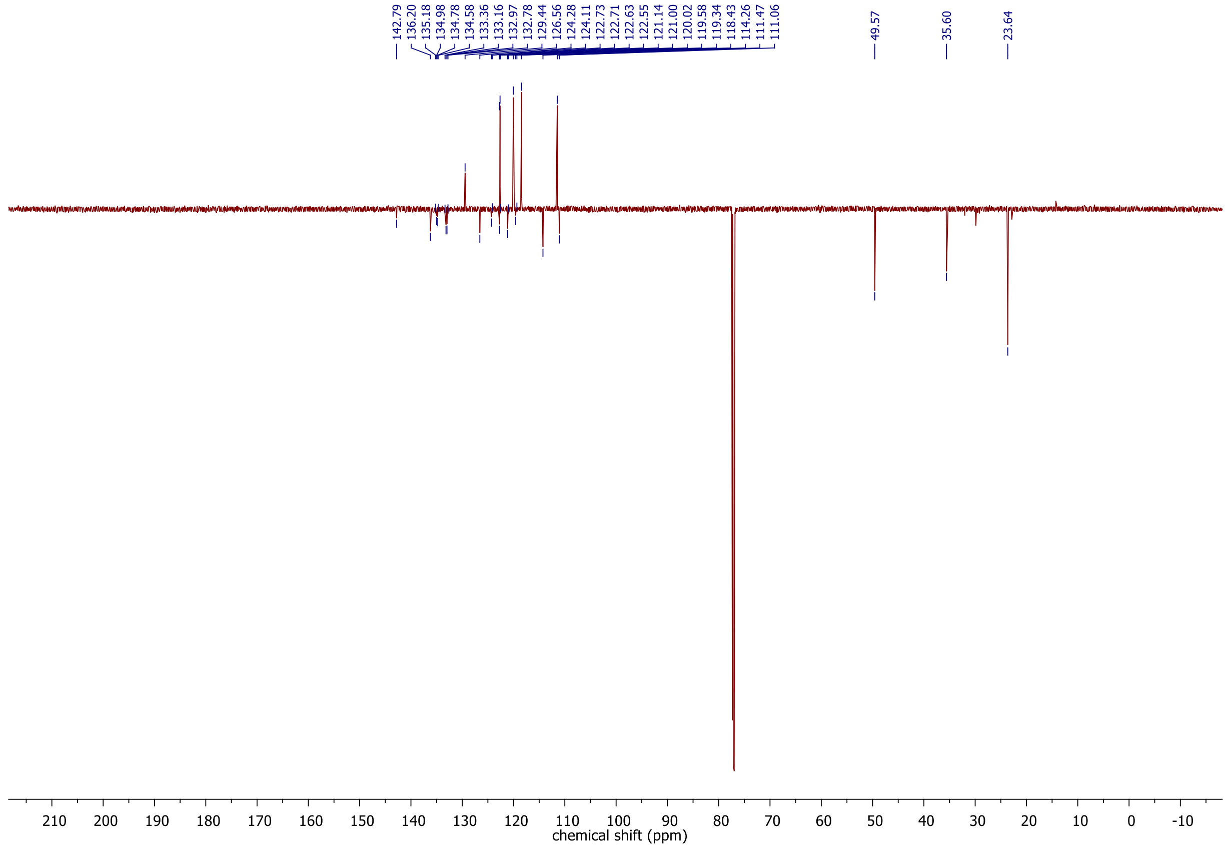

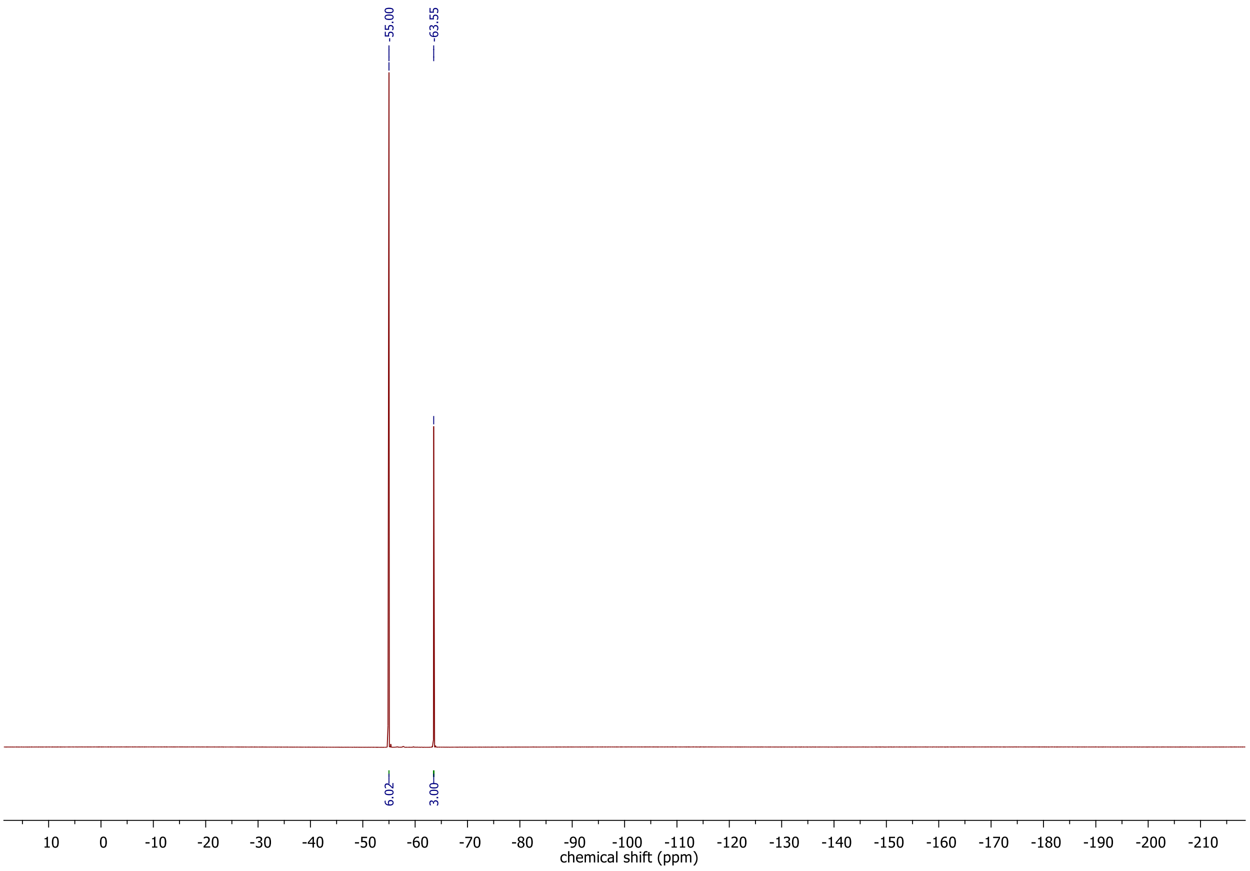
-55.00
-63.55
6.02
3.00
10
0
-10
-20
-30
-40
-50
-60
-70
-80
-90
-100
-110
-120
-130
-140
-150
-160
-170
-180
-190
-200
-210
chemical shift (ppm)

N-[2-(1H-Indol-3-yl)ethyl]-N-propyl-2,4,6-tris(trifluoromethyl)benzenesulfonamide (46)

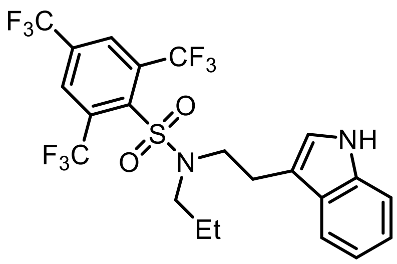

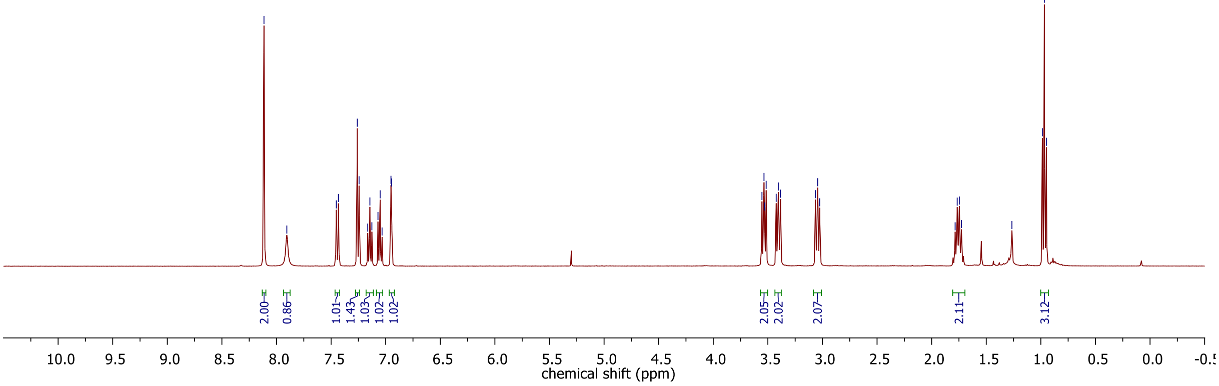

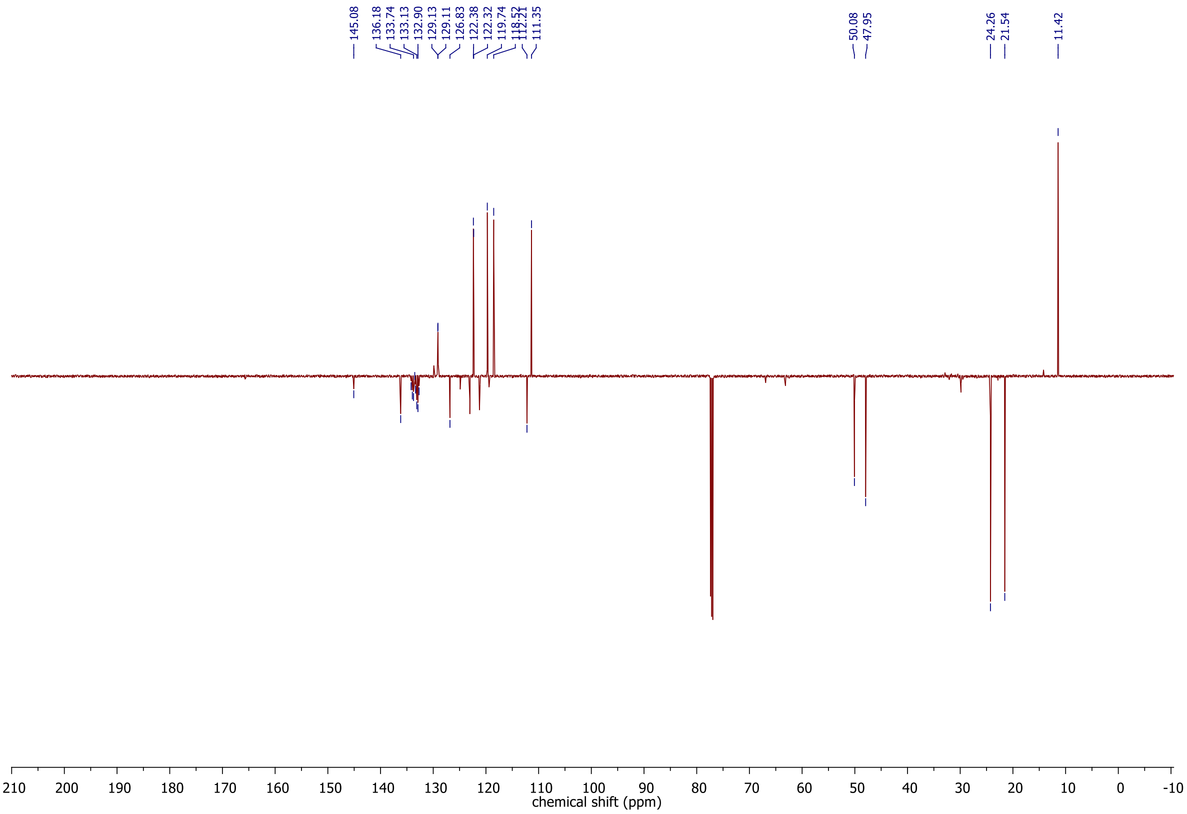

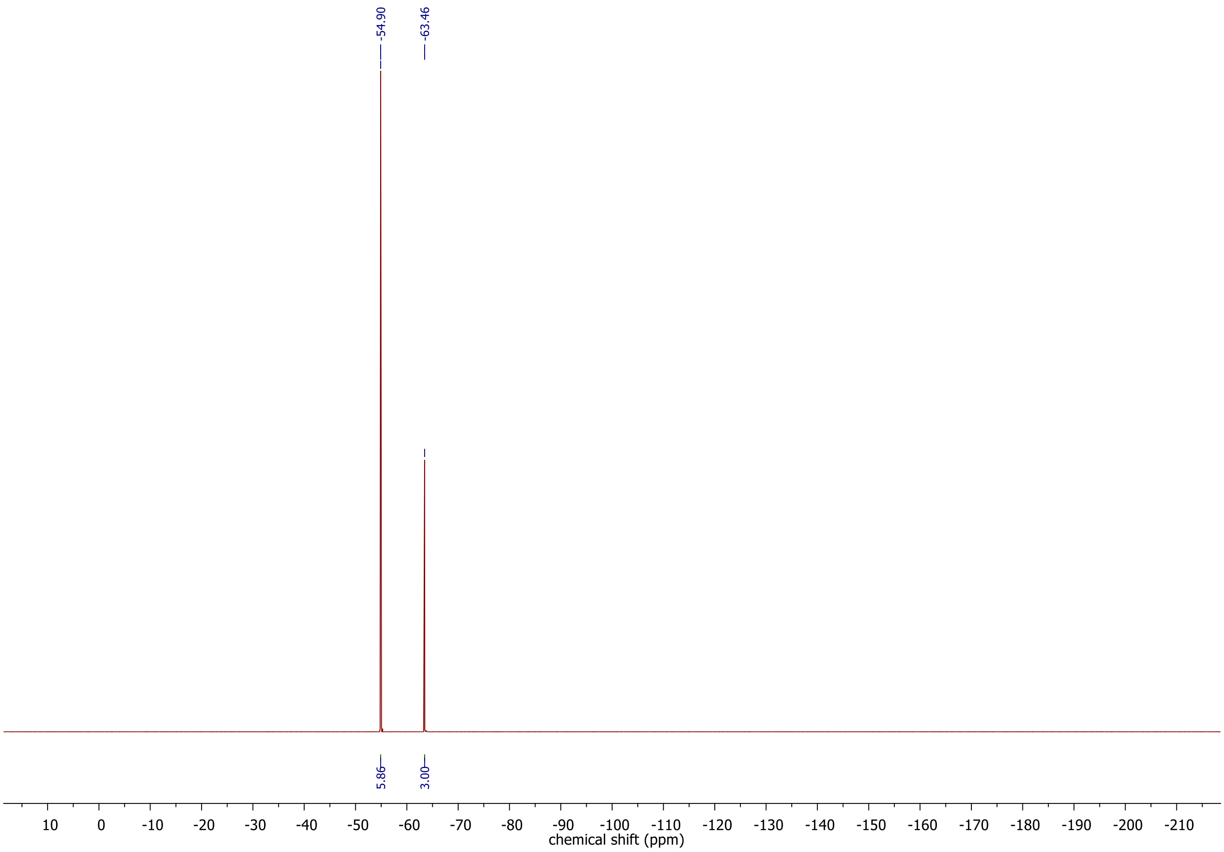

-54.90
-63.46
5.86
3.00
10 0 -10 -20 -30 -40 -50 -60 -70 -80 -90 -100 -110 -120 -130 -140 -150 -160 -170 -180 -190 -200 -210
chemical shift (ppm)

N-(p-Tolyl)-2,4,6-tris(trifluoromethyl)benzenesulfonamide (47)

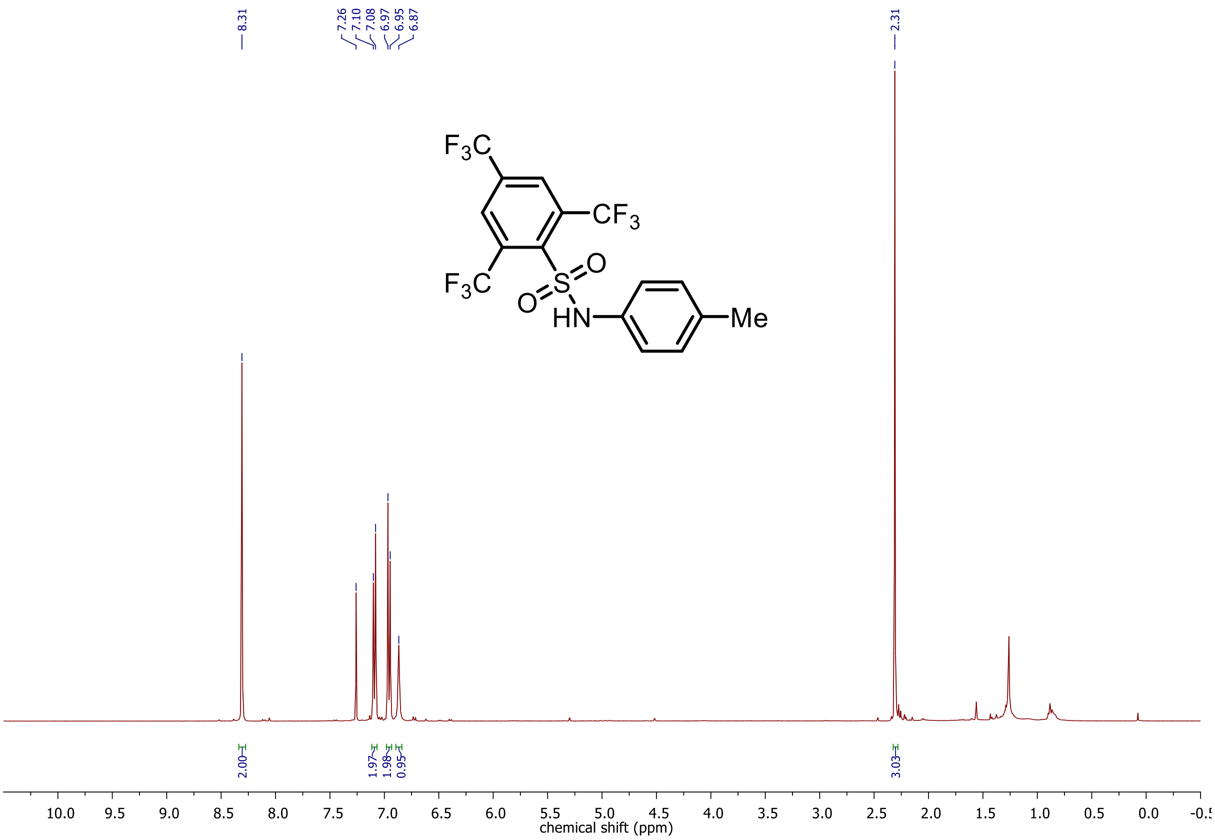

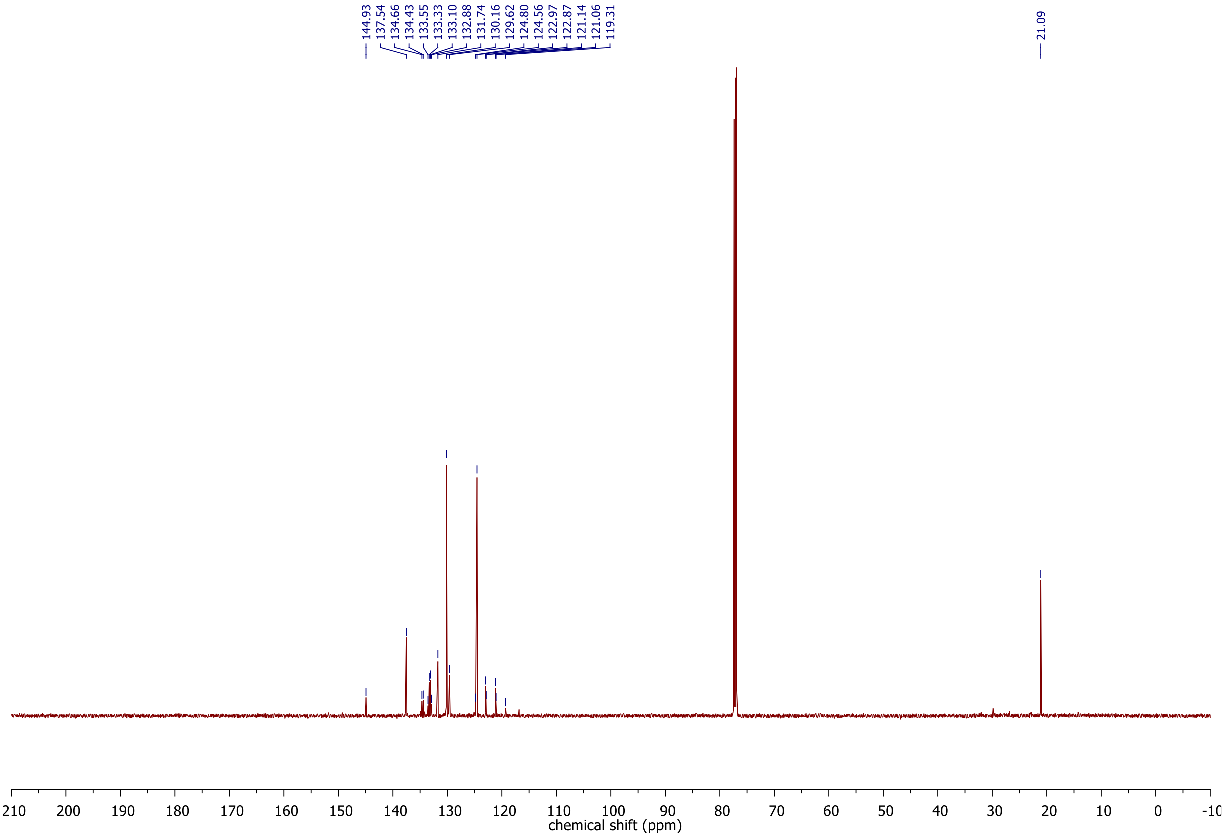

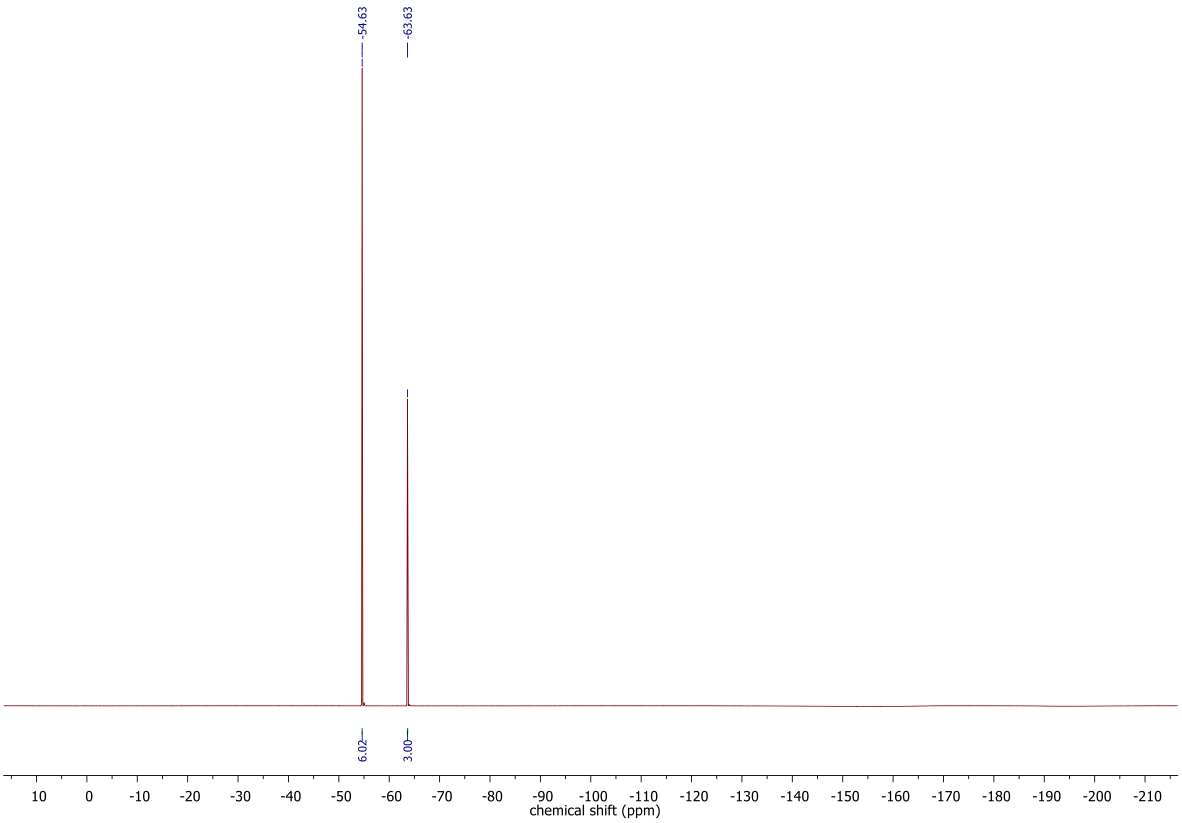

-54.63
-63.63
6.02
3.00
10
0
-10
-20
-30
-40
-50
-60
-70
-80
-90
-100
-110
-120
-130
-140
-150
-160
-170
-180
-190
-200
-210
chemical shift (ppm)

N-(3,4-Dimethoxyphenethyl)-N-phenyl-2,4,6-tris(trifluoromethyl)benzenesulfonamide (48)

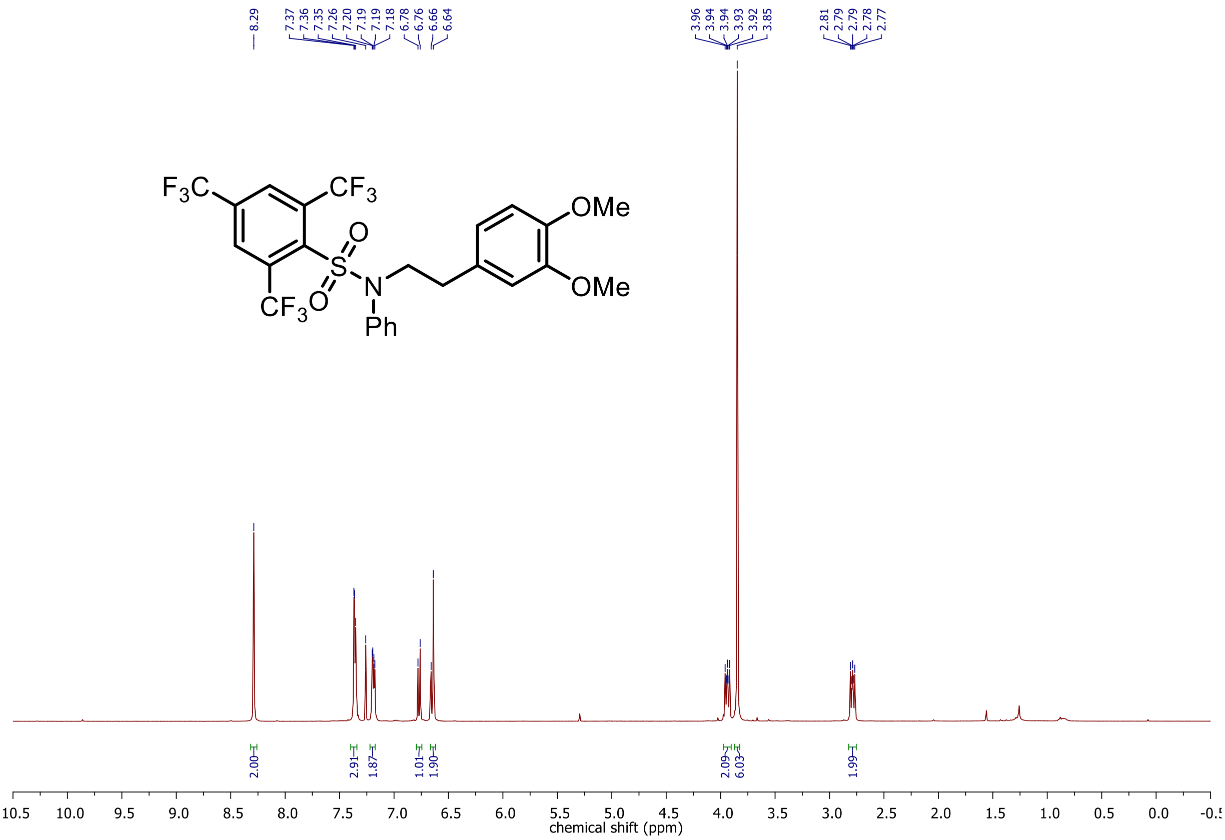

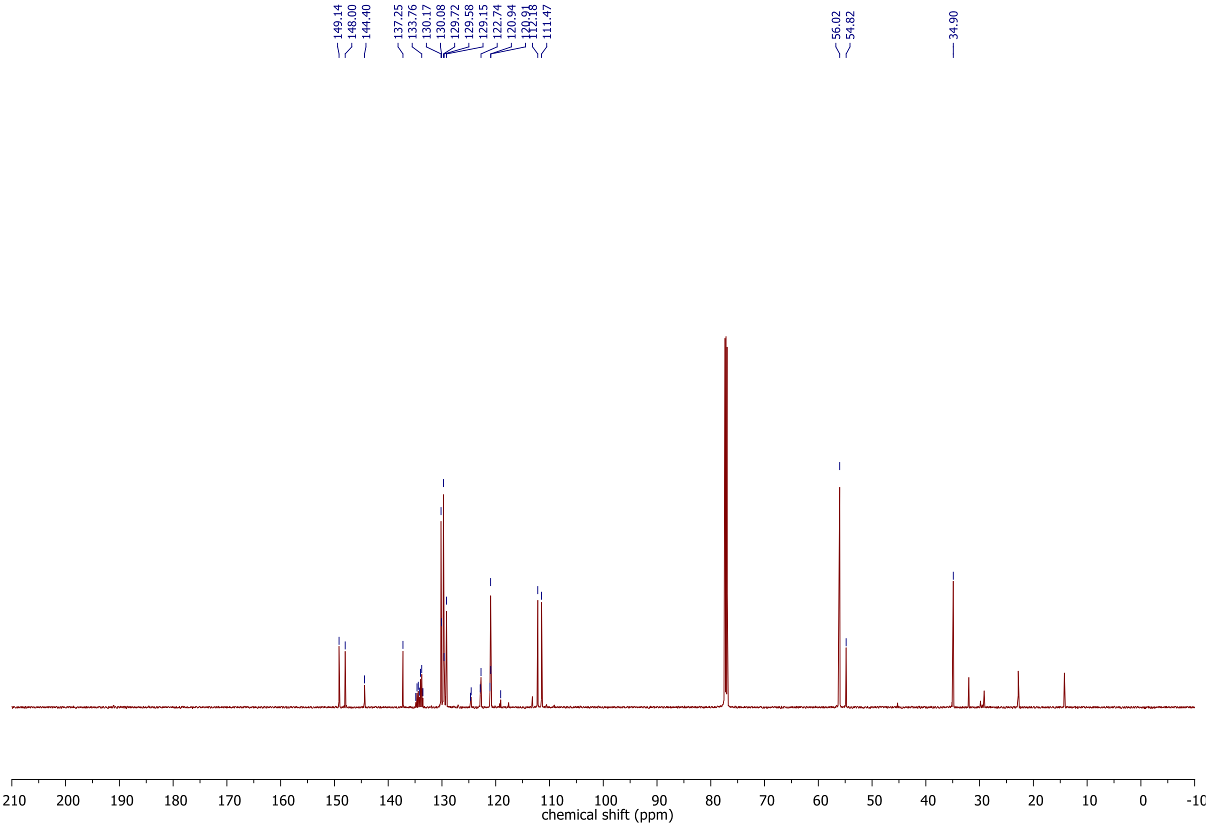

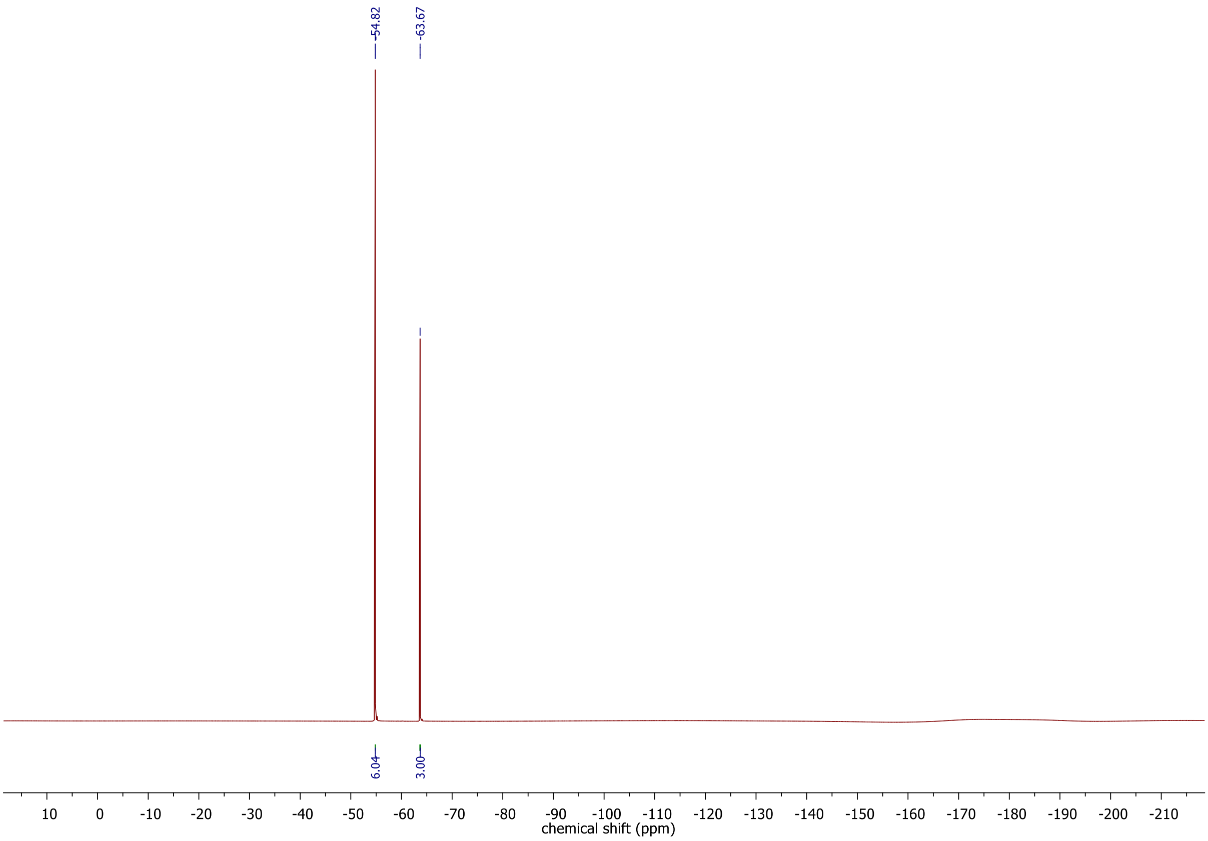

-54.82
-63.67
6.04
3.00
10
0
-10
-20
-30
-40
-50
-60
-70
-80
-90
-100
-110
-120
-130
-140
-150
-160
-170
-180
-190
-200
-210
chemical shift (ppm)

***N*-(2-(1-Phenyl-1*H*-indol-3-yl)ethyl)-2,4,6-tris(trifluoromethyl)benzenesulfonamide (49)**

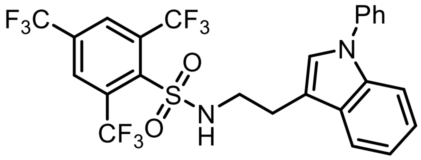

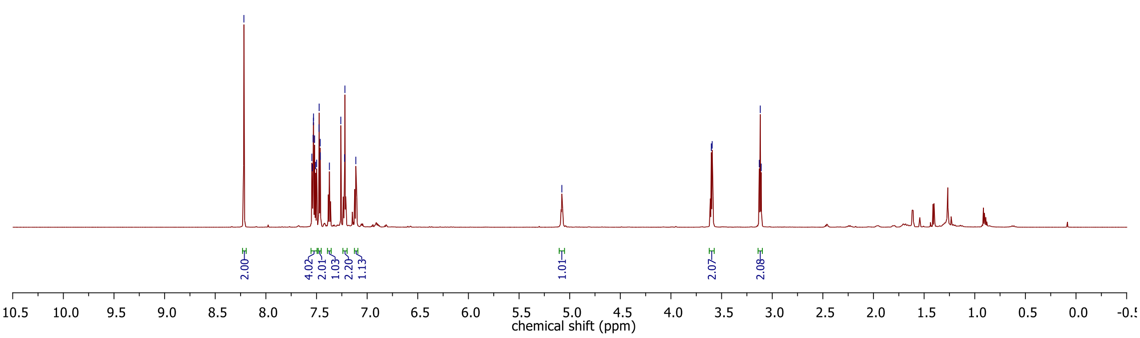

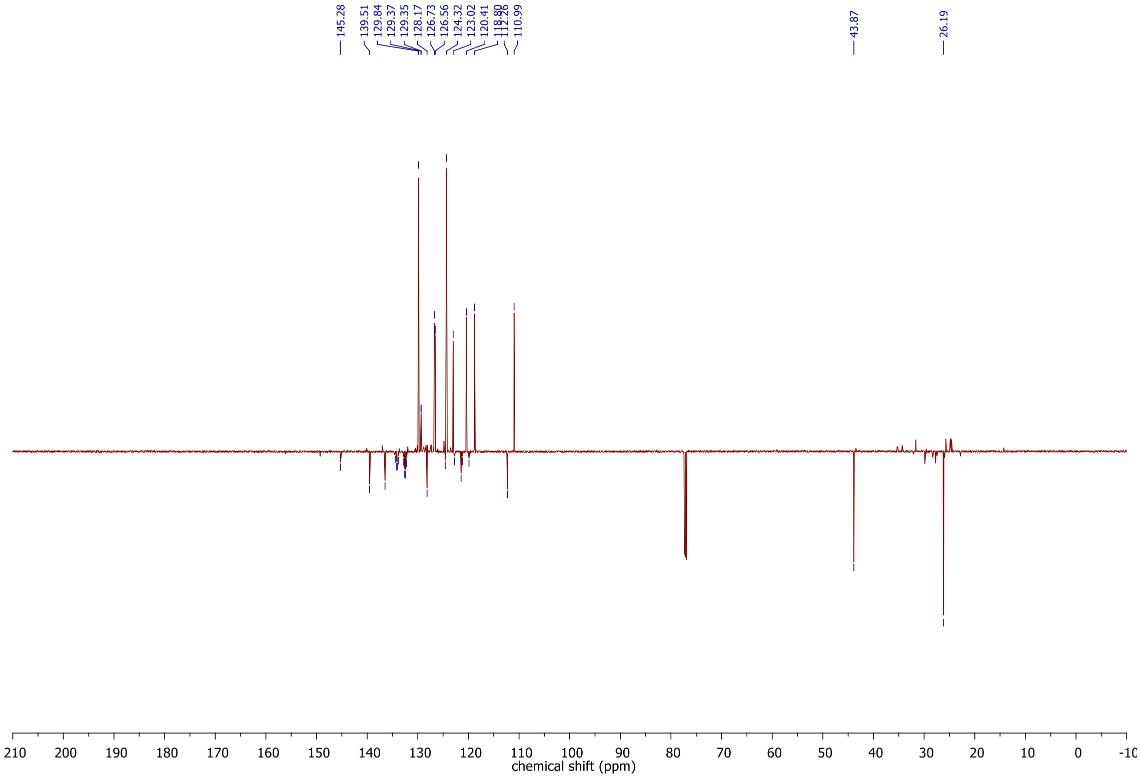

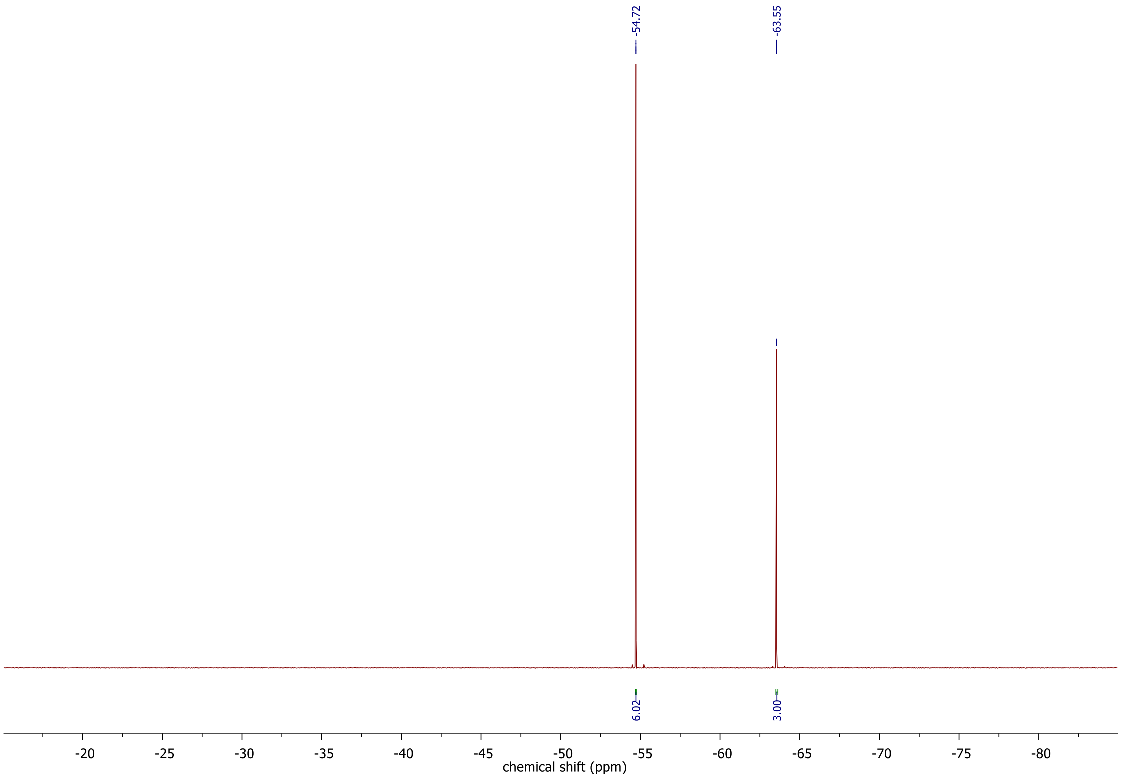
-54.72
-63.55
6.02
3.00
-20
-25
-30
-35
-40
-45
-50
-55
-60
-65
-70
-75
-80
chemical shift (ppm)

Benzyl (S)-1-((2,4,6-tris(trifluoromethyl)phenyl)sulfonyl)aziridine-2-carboxylate (50)

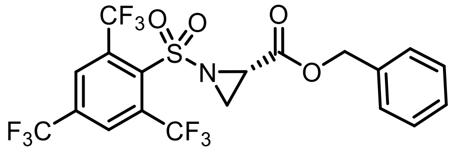

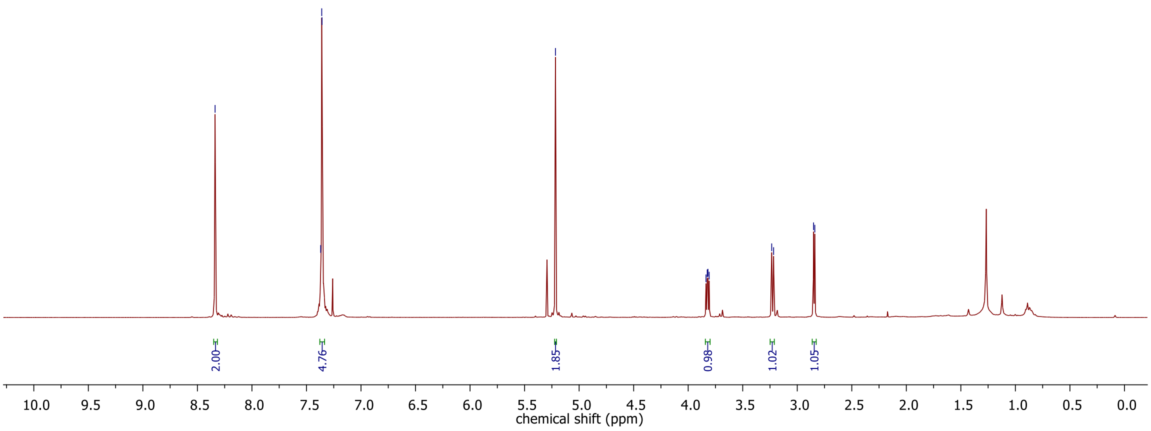

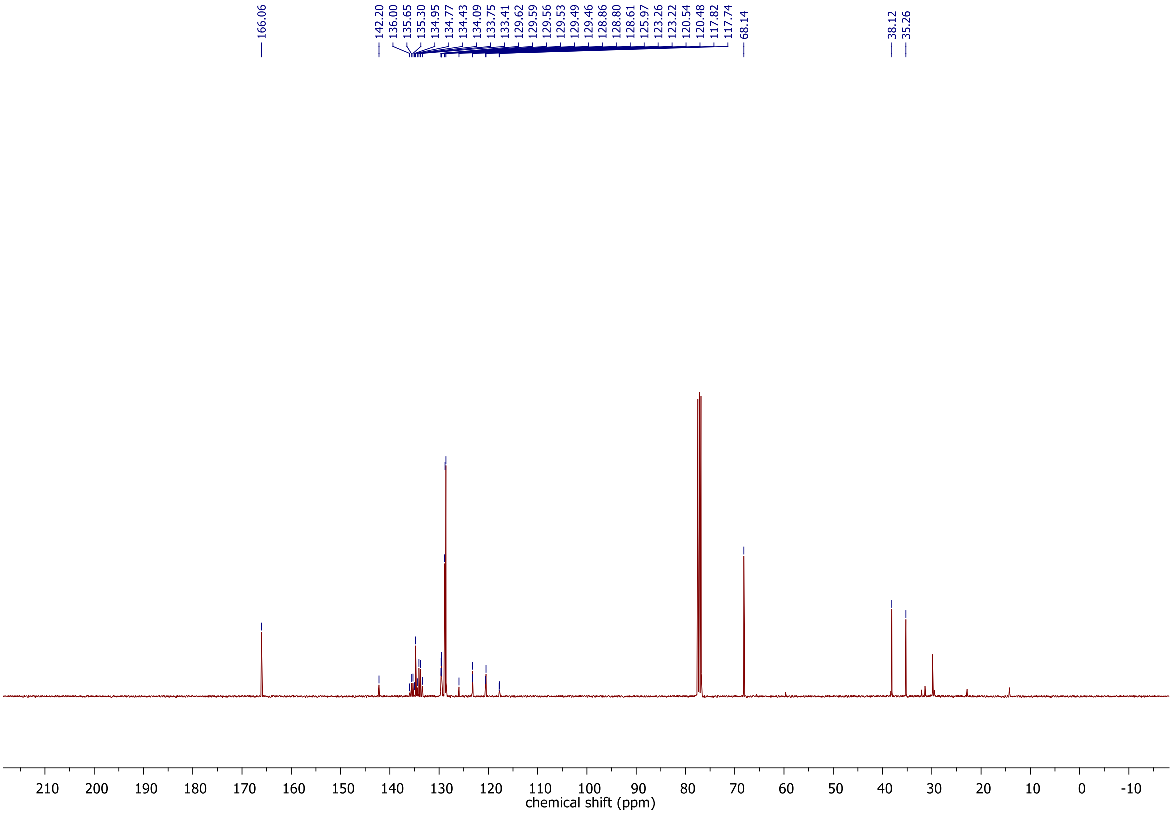

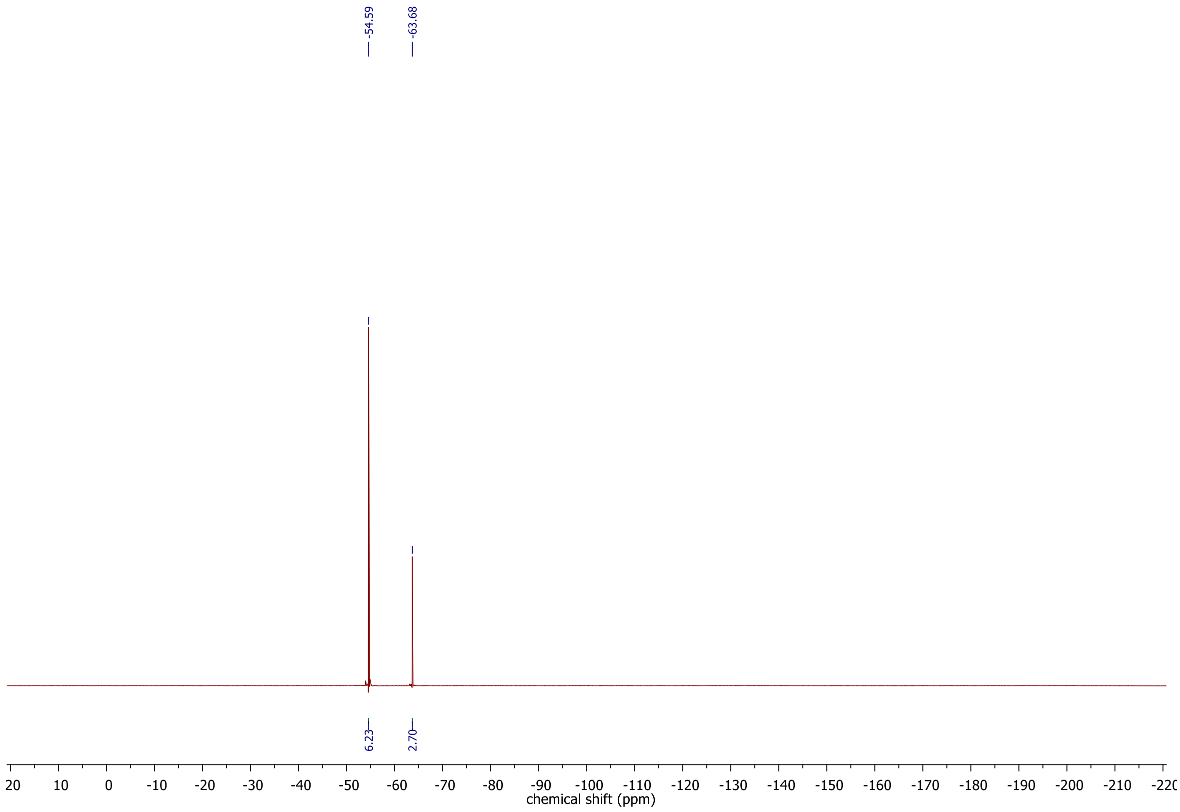
-54.59
-63.68
6.23
2.70
20 10 0 -10 -20 -30 -40 -50 -60 -70 -80 -90 -100 -110 -120 -130 -140 -150 -160 -170 -180 -190 -200 -210 -220
chemical shift (ppm)

Methyl *N*-(4-methoxybenzyl)-*N*-((2,4,6-tris(trifluoromethyl)phenyl)sulfonyl)-*D*-alaninate (51)

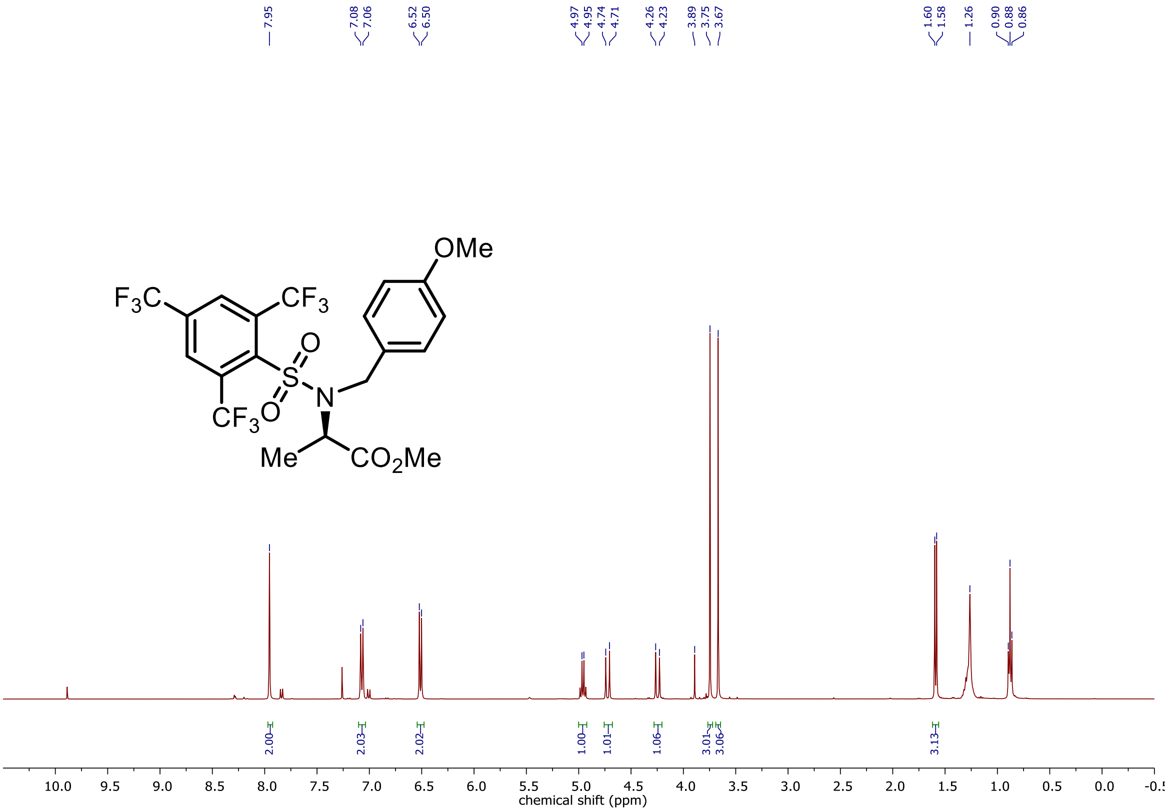

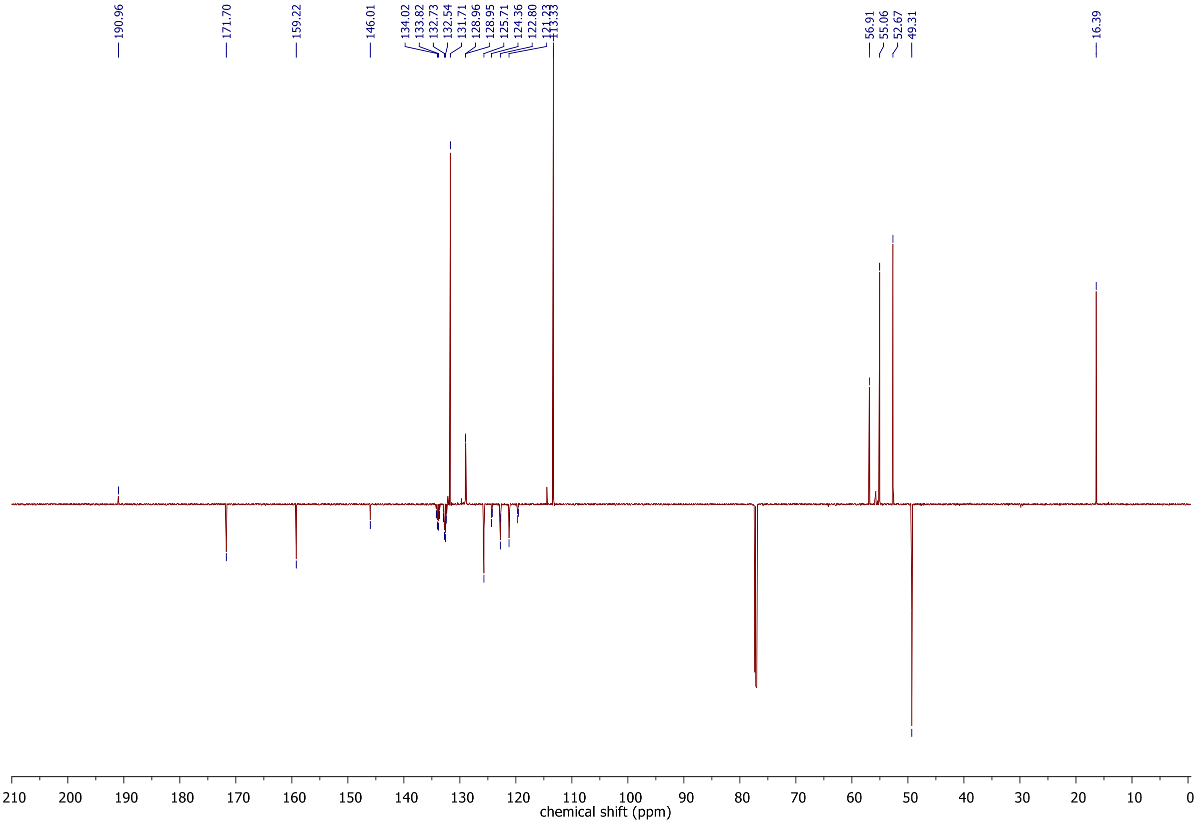

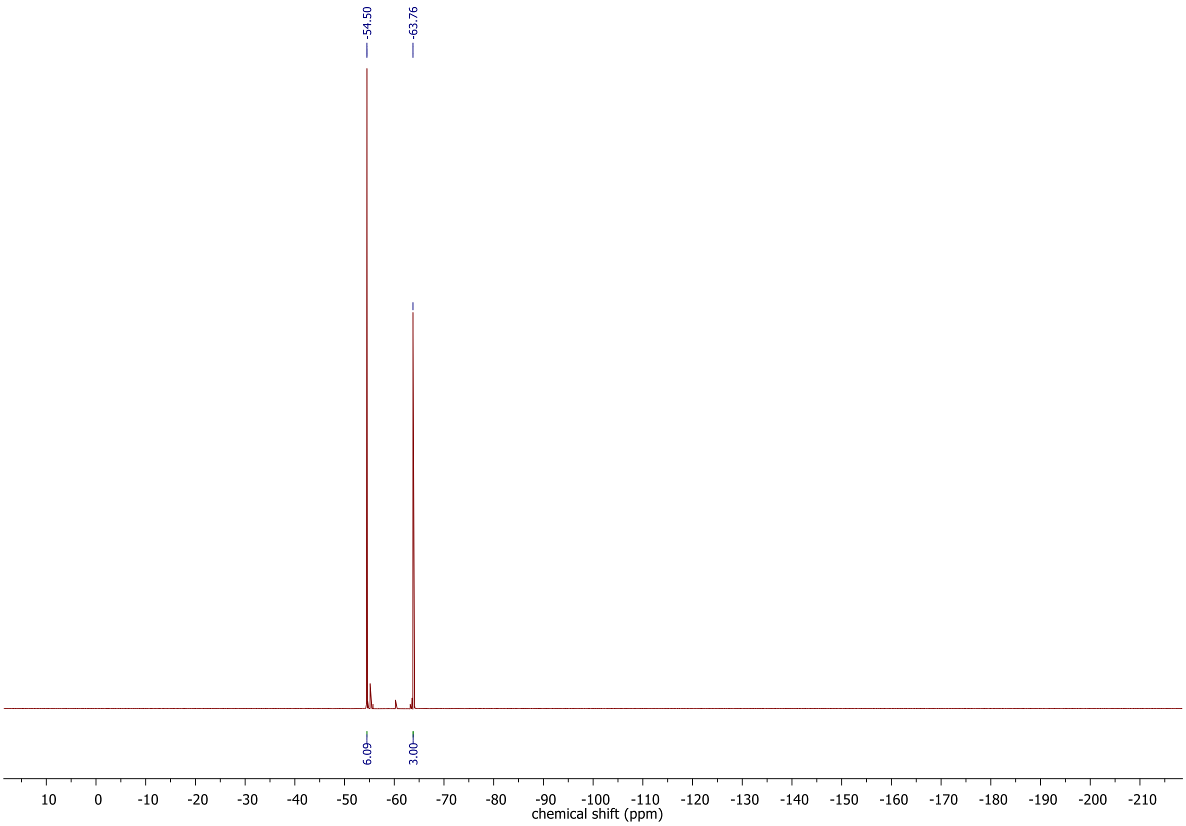
-54.50
-63.76
6.09
3.00
10 0 -10 -20 -30 -40 -50 -60 -70 -80 -90 -100 -110 -120 -130 -140 -150 -160 -170 -180 -190 -200 -210
chemical shift (ppm)

(*R*)-*N*-(1-(naphthalen-1-yl)ethyl)-2,4,6-tris(trifluoromethyl)-*N*-(3-(3-(trifluoromethyl)phenyl)propyl)benzenesulfonamide (52)

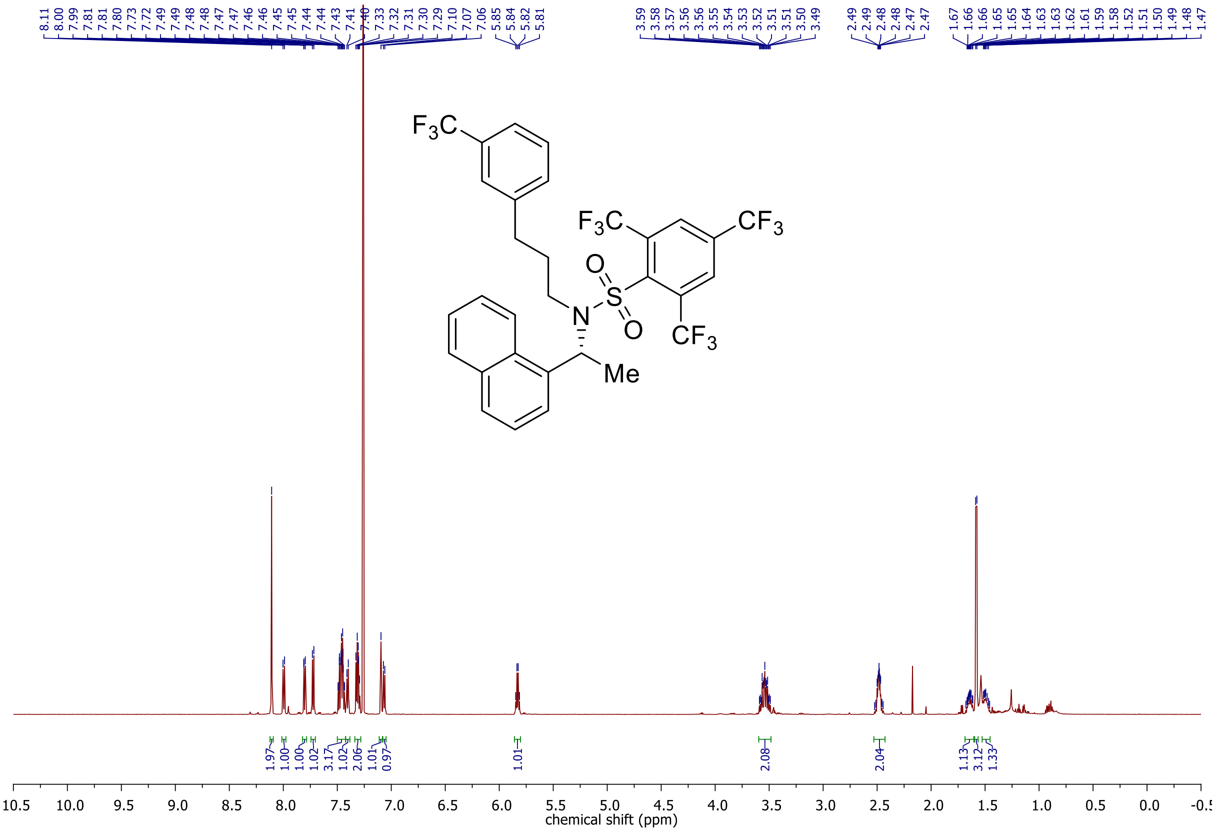

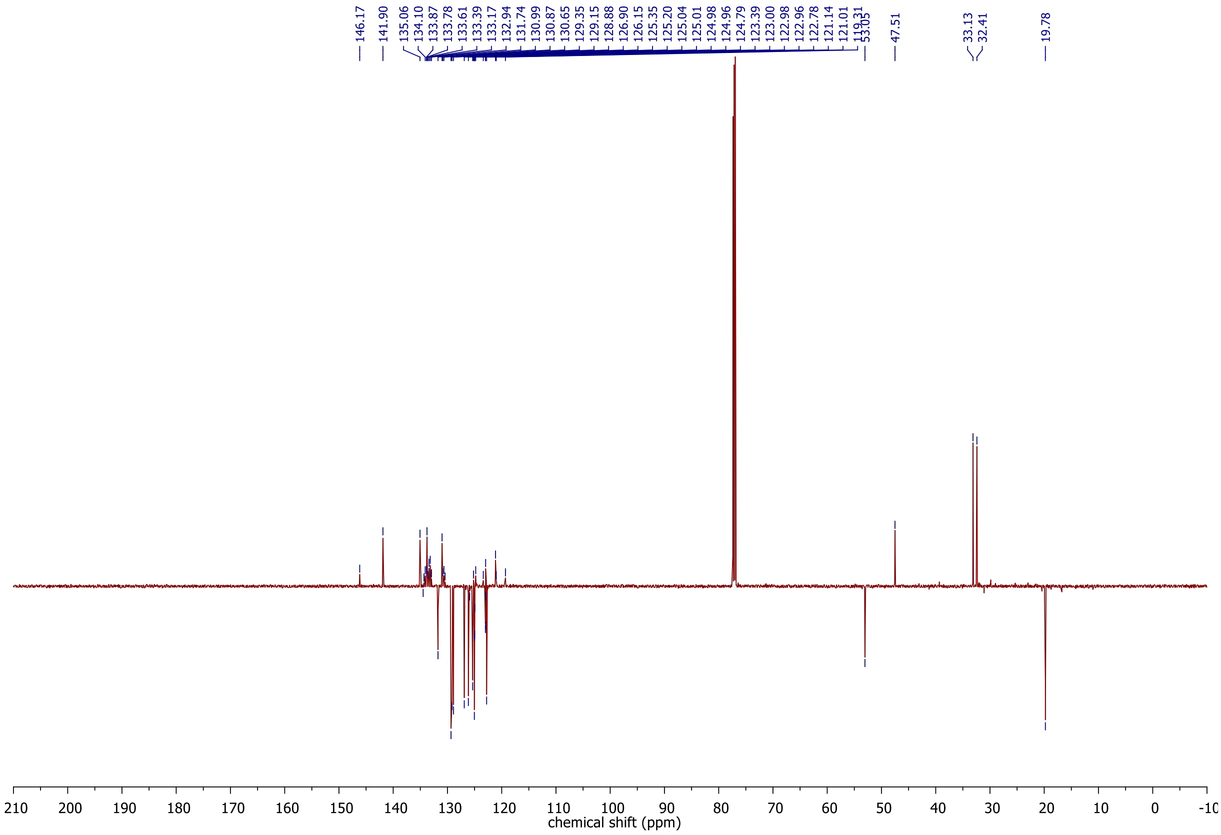

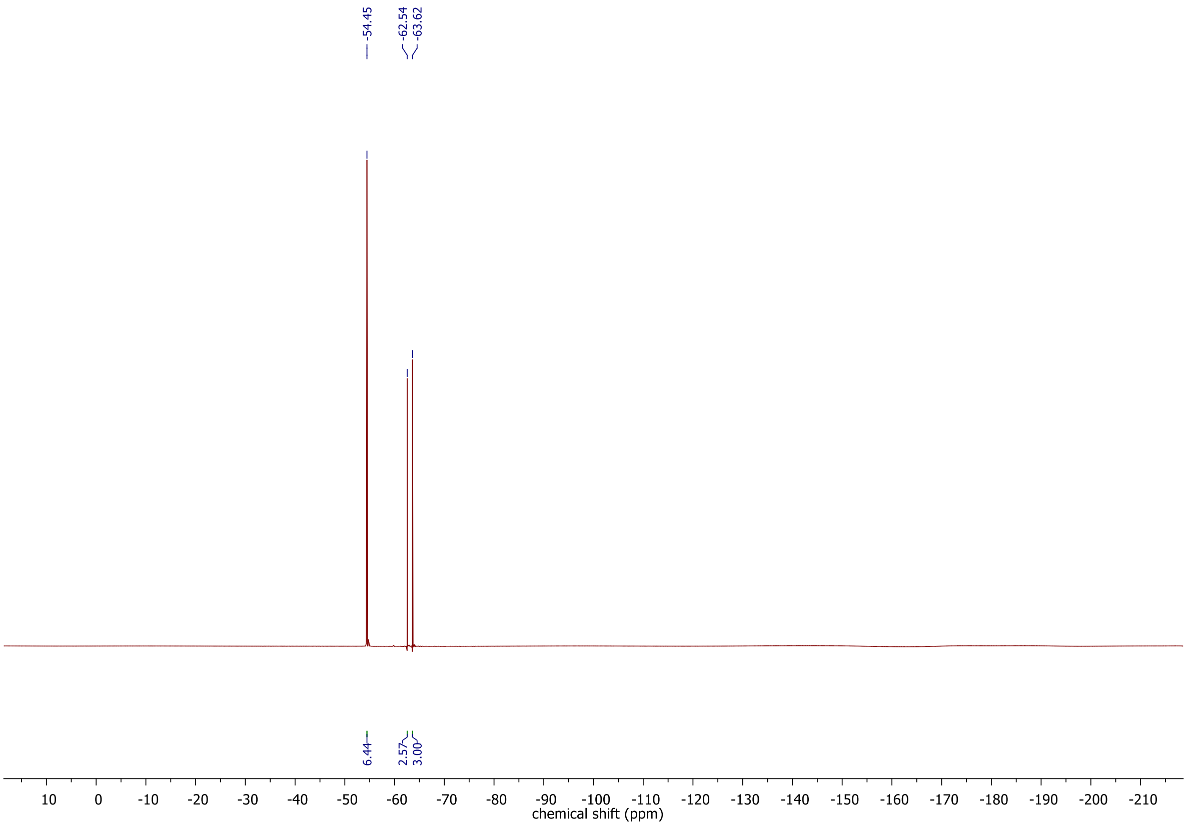

-54.45
-62.54
-63.62
6.44
2.57
3.00
10 0 -10 -20 -30 -40 -50 -60 -70 -80 -90 -100 -110 -120 -130 -140 -150 -160 -170 -180 -190 -200 -210
chemical shift (ppm)

9. X-Ray Crystallographic Data

The X-ray intensity data was measured on Bruker D8 Venture diffractometer equipped with multilayer monochromator, Mo K/α INCOATEC micro focus sealed tube and Oxford cooling system. The structure was solved by Direct Methods. Non-hydrogen atoms were refined with anisotropic displacement parameters. Hydrogen atoms were inserted at calculated positions and refined with riding model. The following software was used: Bruker SAINT software package[i] using a narrow-frame algorithm for frame integration, SADABS[ii] for absorption correction, OLEX2[iii] for structure solution, refinement, molecular diagrams and graphical user-interface, Shelxle[iv] for refinement and graphical user-interface SHELXS-2015[v] for structure solution, SHELXL-2015[vi] for refinement, Platon[vii] for symmetry check. Experimental data and CCDC-Codes Experimental data available online: http://www.ccdc.cam.ac.uk/conts/retrieving.html.

Listing of measured compounds:

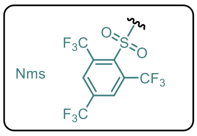

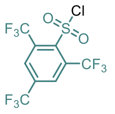

5

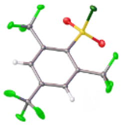

CCDC2244507

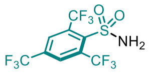

6

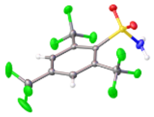

CCDC2244503

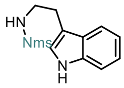

2h

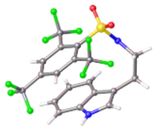

CCDC2244508

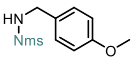

2n

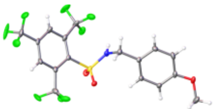

CCDC2244501

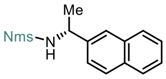

2o

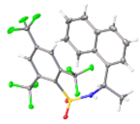

CCDC2244506

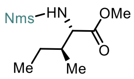

2v

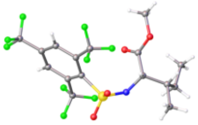

CCDC2244505

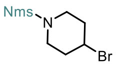

2aa

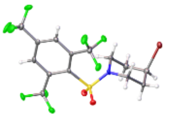

CCDC2244499

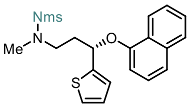

2ag

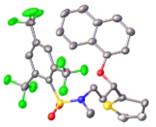

CCDC2244504

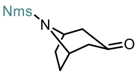

2ai

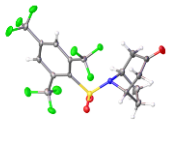

CCDC2244500

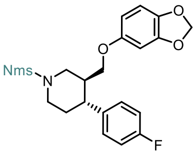

2ak

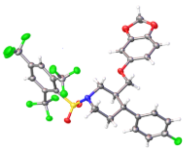

CCDC2244698

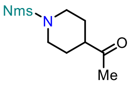

28

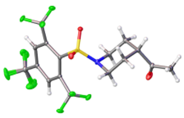

CCDC2244498

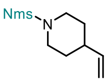

41

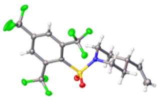

CCDC2244502

References:

[i] Bruker SAINT v8.38B Copyright © 2005-2019 Bruker AXS

[ii] Sheldrick, G. M. (1996). SADABS. University of Göttingen, Germany.

[iii] Dolomanov, O.V., Bourhis, L.J., Gildea, R.J, Howard, J.A.K. & Puschmann, H. , OLEX2, (2009), J. Appl. Cryst. 42, 339-341.

[iv] C. B. Huebschle, G. M. Sheldrick and B. Dittrich, ShelXle: a Qt graphical user interface for SHELXL, J. Appl. Cryst., 44, (2011) 1281-1284.

[v] Sheldrick, G. M. (2015). SHELXS v 2016/4 University of Göttingen, Germany.

[vi] Sheldrick, G. M. (2015). SHELXL v 2016/4 University of Göttingen, Germany.

[vii] A. L. Spek, Acta Cryst. 2009, D65, 148-155.
